# Enantioselective Synthesis of Sealutomicin C

**DOI:** 10.1021/jacs.4c02969

**Published:** 2024-06-17

**Authors:** Stuart M. Astle, Sean Guggiari, James R. Frost, Hamish B. Hepburn, David J. Klauber, Kirsten E. Christensen, Jonathan W. Burton

**Affiliations:** † Chemistry Research Laboratory, University of Oxford, 12 Mansfield Road, Oxford OX1 3TA, U.K.; ‡ UCB Pharma, 216 Bath Road, Slough, Berkshire SL1 3WE, U.K.; § Vertex Pharmaceuticals, 86-88 Jubilee Avenue Milton Park, Abingdon OX14 4RW, U.K.; ∥ Chemical Development, Pharmaceutical Technology & Development, Operations, AstraZeneca, Macclesfield SK10 2NA, U.K.

## Abstract

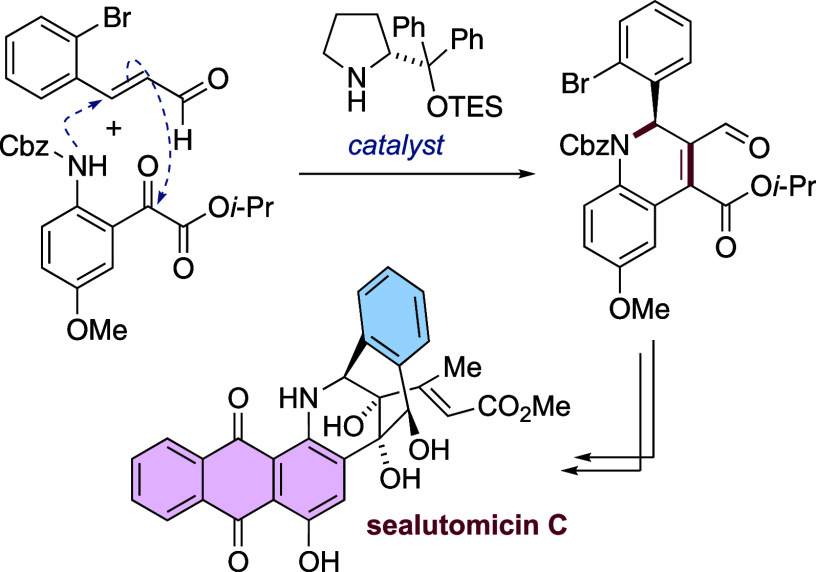

The sealutomicins are a family of anthraquinone antibiotics featuring an enediyne (sealutomicin A) or Bergman-cyclized aromatic ring (sealutomicins B–D). Herein we report the development of an enantioselective organocatalytic method for the synthesis of dihydroquinolines and the use of the developed method in the total synthesis of sealutomicin C which features a transannular cyclization of an aryllithium onto a γ-lactone as a second key step.

## Introduction

**Figure 1:**
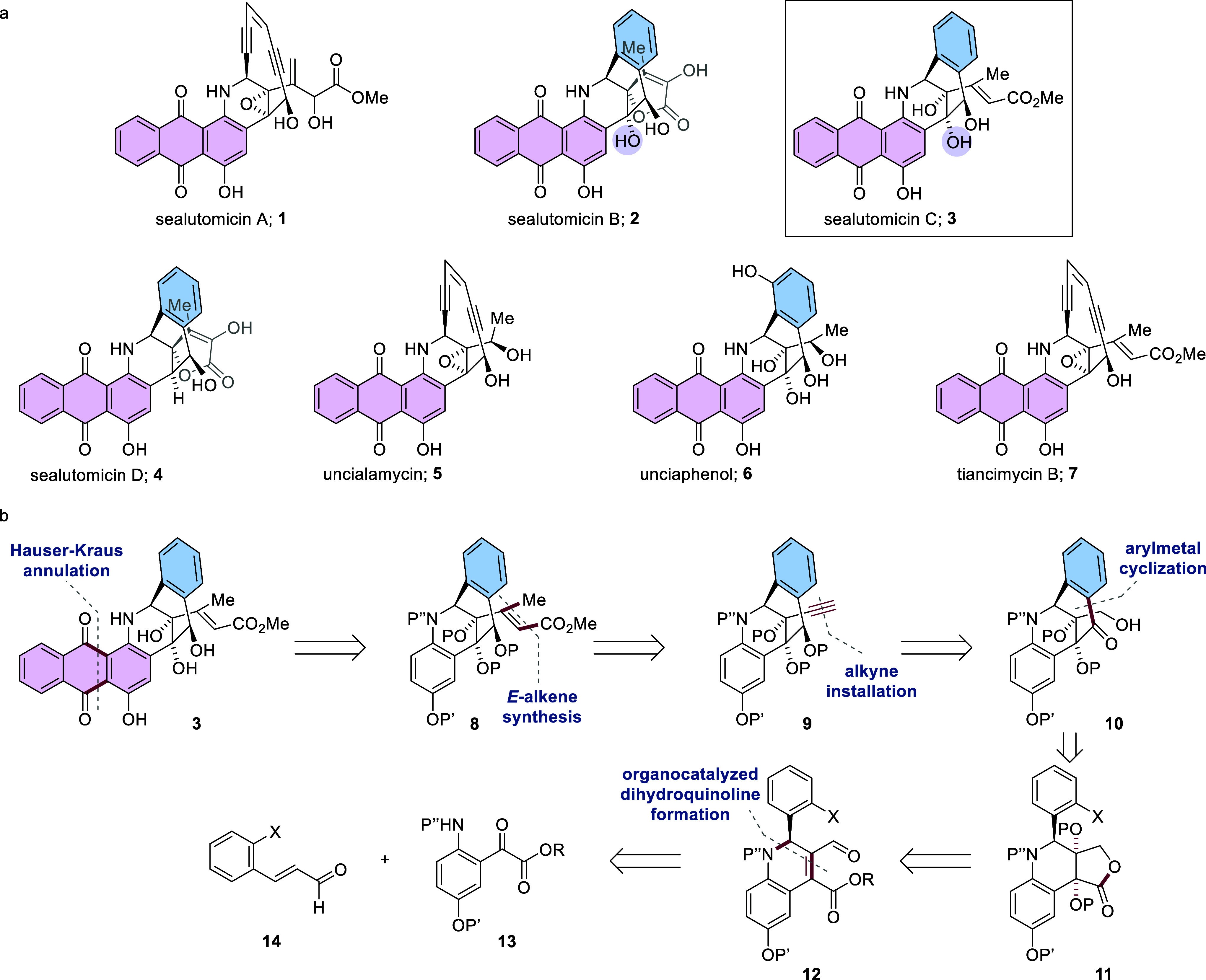
(a) Sealutomicin family of natural products, along with uncialamycin, unciaphenol, and tiancimycin B. (b) Retrosynthetic analysis of sealutomicin C.

A recent report from Igarashi, Sawa, and co-workers described the isolation of four novel anthraquinone-fused natural products, the sealutomicins A–D **1**–**4**, from fermentation of the marine actinomycete *Nonomuraea* sp. MM565M-173N2 (Figure 1a).^1^ In addition to the anthracene-9,10-dione motif shared by all four natural products, sealutomicin A **1** contains a bicyclo[7.3.1]-tridecadiynene core, placing it in the anthraquinone-fused enediyne (AFE) subfamily of natural products which includes dynemicin A, tiancimycin A, yangpumicin A, and uncialamycin **5**.^2−6^ AFEs have attracted considerable attention from the synthetic and medicinal chemistry communities owing to their potent antibiotic and antitumor activities, with notable contributions coming from the Myers, Danishefsky, and Nicolaou groups,^7−15^ including the synthesis of numerous analogues to develop structure–activity relationships.^7,8,13,14^ In contrast, sealutomicins B–D **2**–**4** all possess a bridging aryl ring in place of the enediyne core, proposed to be formed biosynthetically via Bergman cyclization of an enediyne precursor, such as sealutomicin A **1**. Such reactivity has previously been implicated in the biosynthesis of the natural product unciaphenol **6** from its enediyne precursor uncialamycin **5**.^16^ In both cases, aryl ring formation is thought to be preceded by *syn*-hydrolysis of an epoxide unit, bringing the two alkyne units close enough together to trigger the cyclization. Igarashi, Sawa, and co-workers note in the isolation paper that sealutomicin C **3** may be identical to a compound isolated by Shen and co-workers, namely, the Bergman cyclization product of tiancimycin B **7**.^17^

The sealutomicins **1**–**4** all display in vitro antibacterial activity against Gram-positive bacteria with sealutomicin A **1** being more potent than the cycloaromatized sealutomicins B–D **2**–**4**. Sealutomicin A **1** also shows similar effects against Gram-negative bacteria. The antibacterial activity of sealutomicin A **1** is proposed to arise from the ability of the enediyne warhead to form a benzenoid biradical that triggers bacterial DNA scission via hydrogen atom abstraction from the DNA backbone, in the same manner as other AFEs such as uncialamycin **5** and dynemicin A. Sealutomicins B–D all lack this key enediyne motif capable of inducing DNA damage, and as such, the mechanism of action by which they exert their antimicrobial effects is currently unknown. Sealutomicins B–D **2**–**4** share strong structural similarities with the natural product unciaphenol **6**, which was found to display in vitro anti-HIV activity against both wild-type and antiretroviral-resistant strains of HIV.^16^ This raises the possibility that sealutomicins B–D **2**–**4** may also display similar antiviral properties. In light of the biological activities and intriguing chemical structures, we sought to develop a synthetic route to the sealutomicins. Herein, we report the development of an organocatalytic enantioselective dihydroquinoline synthesis which, along with a transannular cyclization of an aryl lithium onto a γ-lactone, feature as key steps in our total synthesis of sealutomicin C **3**. Additionally, our successful synthesis of **3** demonstrates that the Bergman cyclization product of tiancimycin B, isolated by Shen and co-workers,^17^ and sealutomicin C **3** have the same structure.

## Results and Discussion

Our strategy for the synthesis of sealutomicin C **3** is shown retrosynthetically in Figure 1b. We reasoned that the anthraquinone moiety of sealutomicin C **3** could be installed via a Hauser–Kraus annulation on an iminoquinone formed from oxidation of an alkoxy aniline such as **8**, as previously utilized by Nicolaou et al. in the syntheses of uncialamycin **5**,^11−13^ leading to a protected triol derivative from which **3** could be formed on global deprotection.

The α,β-unsaturated ester functionality in **8** was to be introduced from terminal alkyne **9**, which itself would be derived ultimately from ketone **10**. The key disconnection in our proposed route to sealutomicin C was to be a 6-*exo*-*trig* cyclization of an arylmetal derived from **11** (X = halide) onto a γ-lactone to yield ketone **10**. The initial aim was to conduct a lithium–halogen exchange reaction of the bromide **11** (X = Br) given the precedent for lithium–halogen exchange to outcompete addition of alkyllithium reagents to carbonyl groups.^18−20^ The key cyclization substrate was to be prepared from the dihydroquinoline **12**, which we envisaged would be synthesized by condensation of a cinnamaldehyde **14** and an aniline **13** bearing an α-ketoester under enantioselective organocatalysis.

Numerous methods for the enantioselective synthesis of 1,2-dihydroquinolines have been reported including kinetic resolution of 1,2-dihydroquinolines;^21−26^ additions to quinolines and 1,2-dihydroquinolines and their derivatives;^27−36^ and direct enantioselective synthesis of 1,2-dihydroquinolines.^37−40^ Two early examples involving 1,2-dihydroquinoline synthesis, from the groups of Wang and Córdova et al., utilized diarylprolinol silyl ether catalysts to effect an aza-Michael/aldol cascade coupling of 2-aminobenzaldehydes with cinnamaldehydes in order to access aryl-substituted 1,2-dihydroquinolines in high yields and >90% ee.^37,38^ We sought to adapt these methods to the synthesis of a variety of dihydroquinolines **18** by replacing the 2-aminobenzaldehyde component with the analogous α-ketoester **15** (Table 1). To investigate the proposed modified organocatalytic cascade, representative α-ketoesters **15** were readily prepared from the analogous isatins in two steps (see the Supporting Information, p S3). Using (*E*)-cinnamaldehyde **16a** and α-ketoester **15a**, the conditions of Wang et al.,^38^ namely, catalyst (*S*)-**17a** in DCE with 4 Å molecular sieves and sodium acetate as additive, gave poor conversion of reactants to a mixture of the desired 1,2-dihydroquinoline (−)-**18a** (structure confirmed by single-crystal X-ray diffraction—see the Supporting Information, p S52 and CIF) and a single diastereomer of aldol product (−)-**19a** [Table 1, entry 1, the configuration of (±)-**19a** was assigned from ^1^H–^1^H NMR coupling constant and ^1^H–^1^H NOESY analysis—see the Supporting Information, pp S16–S17 and p S116]. Pleasingly, (−)-**18a** was isolated in high enantiomeric excess, and dehydration of (−)-**19a** under the reaction conditions both with and without chiral catalyst (*S*)-**17a** provided (−)-**18a** with similarly high enantiomeric excess.

**Table 1:**
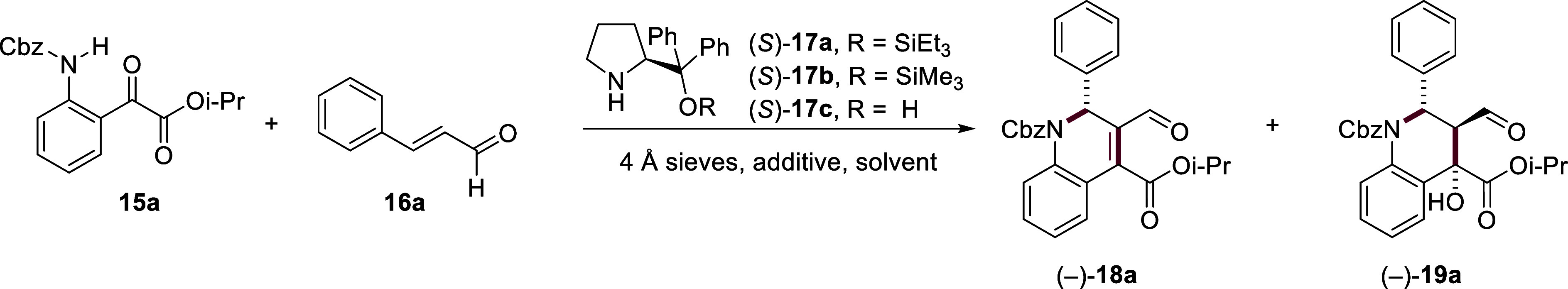
Optimization of the Dihydroquinoline Synthesis

entry^a^	catalyst	additive	solvent	yield of (−)-18a/(−)-19a (%)^b^	ee of (−)-18a (%)^c^
1	**17a**	NaOAc	ClCH_2_CH_2_Cl	8/10	98
2	**17b**	NaOAc	ClCH_2_CH_2_Cl	12/13	88
3	**17a**	none	ClCH_2_CH_2_Cl	9/6	96
4	**17a**	PhCO_2_H	ClCH_2_CH_2_Cl	98/0	97
5^d^	**17a**	PhCO_2_H	ClCH_2_CH_2_Cl	23/42	99
6^e^	**17a**	PhCO_2_H	ClCH_2_CH_2_Cl	91/0	97
7^e^	**17b**	PhCO_2_H	ClCH_2_CH_2_Cl	83/0	99
8^e^	**17c**	PhCO_2_H	ClCH_2_CH_2_Cl	<5/0	n.d.
9^e^	**17a**	PhCO_2_H	CH_2_Cl_2_	98/0	99
10^e^	**17a**	PhCO_2_H	PhCH_3_	97/0	98
11^e^	**17a**	PhCO_2_H	THF	43/24	98
12^f^	**17a**	PhCO_2_H	CH_2_Cl_2_	92/0	98^g^

a General conditions: **15a** (3.0 equiv), **16a** (1.0 equiv, 0.15 mmol), (*S*)-**17a** (20 mol %), additive (50 mol %), 4 Å MS (75 mg), solvent (0.5 M), RT.

b Isolated yield based on cinnamaldehyde starting material.

c ee determined by chiral HPLC.

d Molecular sieves omitted.

e **15a** (2.0 equiv).

f **15a** (2.0 equiv), **16a** (1.0 equiv, 3.0 mmol), (*R*)-**17a** 20 mol %, in place of (*S*)-**17a**, PhCO_2_H (50 mol %), 4 Å MS (1.0 g), CH_2_Cl_2_ (0.5 M).

g (+)-**18a** [enantiomer of (−)-**18a** formed as using (*R*)-**17a**].

It was found that the additive played a vital role in the catalyst turnover, with benzoic acid in place of sodium acetate resulting in complete consumption of **16a** without accumulation of **19a**, while requiring a lower excess of **15a** (Table 1, entries 3, 4, and 6). Meanwhile, the absence of molecular sieves considerably reduced the turnover (Table 1, entry 5). While the TMS-protected diphenylprolinol catalyst (*S*)-**17b** gave the product (−)-**18a** with notably lower enantiomeric excess in the presence of a basic additive (Table 1, entry 2), with an acidic additive, the product was formed with similar enantiomeric excess as when using catalyst (*S*)-**17a** (Table 1, entries 6 and 7). Catalyst (*S*)-**17c** bearing a free alcohol resulted in an almost complete lack of reactivity (Table 1, entry 8). With catalyst (*S*)-**17a**, the reaction worked similarly well in other chlorinated solvents as well as toluene, but more polar solvents resulted in lower yields and incomplete dehydration to give (−)-**18a** (Table 1, entries 9–11). Reduction in the loading of molecular sieves was possible and resulted in the optimal reaction conditions which were readily performed on an up to 3 mmol scale (Table 1, entry 12).

**Chart 1:**
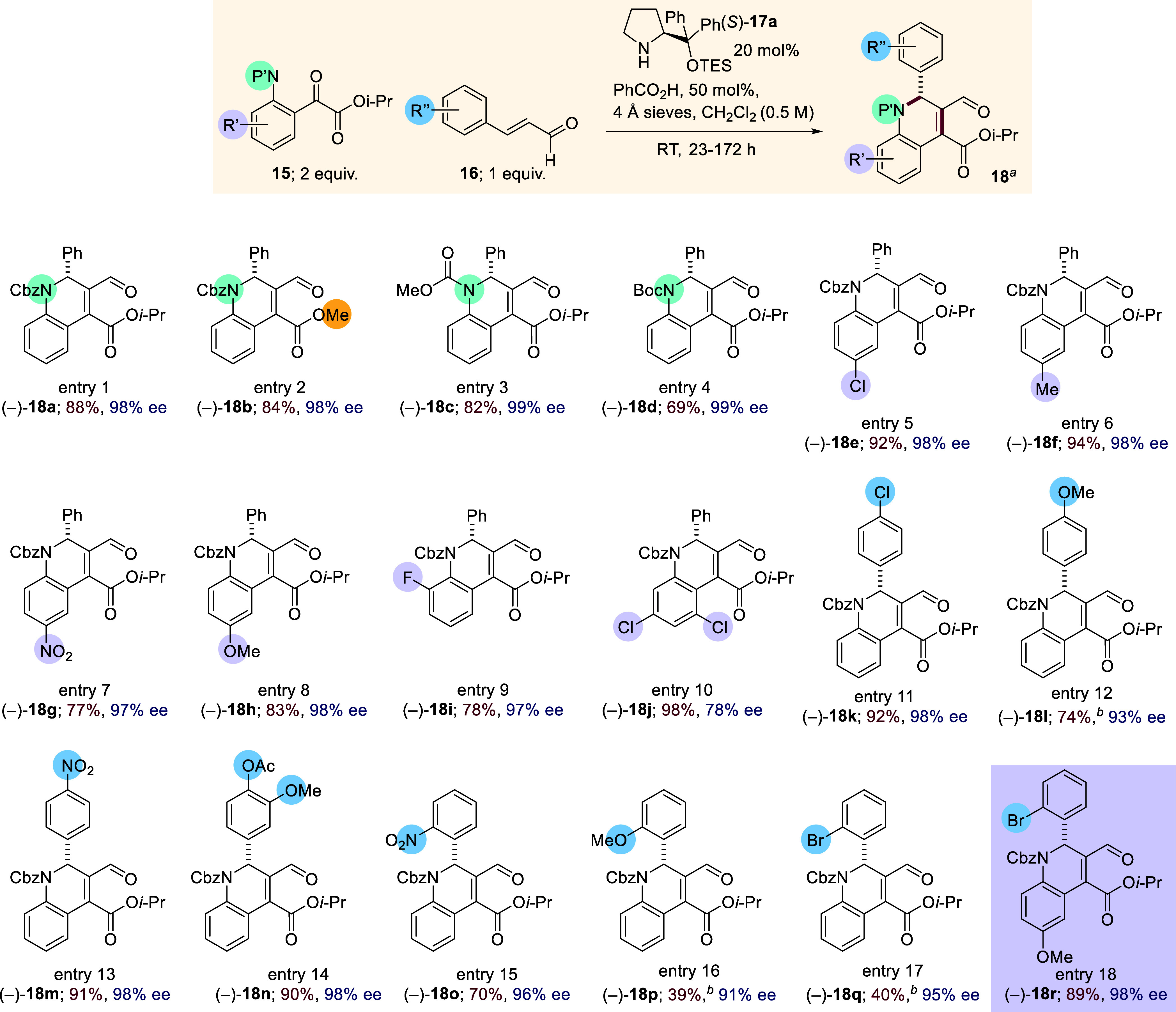
Substrate Scope for Enantioselective Organocatalytic Dihydroquinoline Synthesis

Having established optimized conditions for the modified dihydroquinoline synthesis, using unsubstituted α-ketoester **15a** and cinnamaldehyde **16a**, the scope of the method was investigated, using catalyst (*S*)-**17a** derived from l-proline. The scope included variation of the aniline protecting group and substitution on the aromatic rings of both aniline and cinnamaldehyde. Carbamate protection of the aniline nitrogen atom was well-tolerated with both Boc and methyl carbonate groups giving the desired products in good yields and enantiomeric excesses with cinnamaldehyde **16a** (Chart 1, entries 1–4, (−)-**18a**–**d**, 69–88% yield, 98–99% ee). Using Cbz-protected anilines, a brief scope of the substitution on the aniline ring was investigated with numerous substituents being well-tolerated including 4-chloro, 4-methyl, 4-nitro, and 4-methoxy [Chart 1, entries 5–8, (−)-**18e**–**h**, yields 77–94%, ee, 97–98%]. 2-Fluoro- and 3,5-dichloro-substituted anilines gave the corresponding products (−)-**18i**, 78% yield, 97% ee and (−)-**18j**, 98% yield, 78% ee, respectively (Chart 1, entries 9 and 10). Substitution on the aromatic ring of the cinnamaldehyde was also accommodated. 4-Chloro-, methoxy-, and nitro-substituted cinnamaldehydes gave the corresponding dihydroquinolines in good yields and enantiomeric excesses [Chart 1, entries 11–13, (−)-**18k**–**m** 74–92% yield, 93–98% ee], with 3,4-disubstitution also tolerated giving (−)-**18n** in 90% yield and 98% ee (Chart 1, entry 14). 2-Substitution on the aromatic ring of the cinnamaldehyde gave the dihydroquinolines in high enantiomeric excesses (91–96%) and yields ranging from 39 to 70% [Chart 1, entries 15–17, (−)-**18o**–**q**]. Importantly, for the synthesis of sealutomicin C (**3**), dihydroquinoline (−)-**18r**, bearing the necessary functionality on both aromatic rings for the synthesis of the natural product, could be readily formed in 89% yield and 98% ee (Chart 1, entry 18). Overall, this methodology provided the desired dihydroquinoline products in good yields and with high enantiomeric excesses.

a Yield based on cinnamaldehyde starting material. b Incomplete conversion to a dihydroquinoline product.

The absolute configuration of dihydroquinoline (−)-**18k** was determined to be (*R*) by single-crystal X-ray diffraction (see the Supporting Information, p S53, and CIF), with the configuration of all other products assigned by analogy. Having identified **18r** as having the appropriate core from which to construct sealutomicin C **3**, we now embarked on our synthesis of the natural product.

**Scheme 1:**
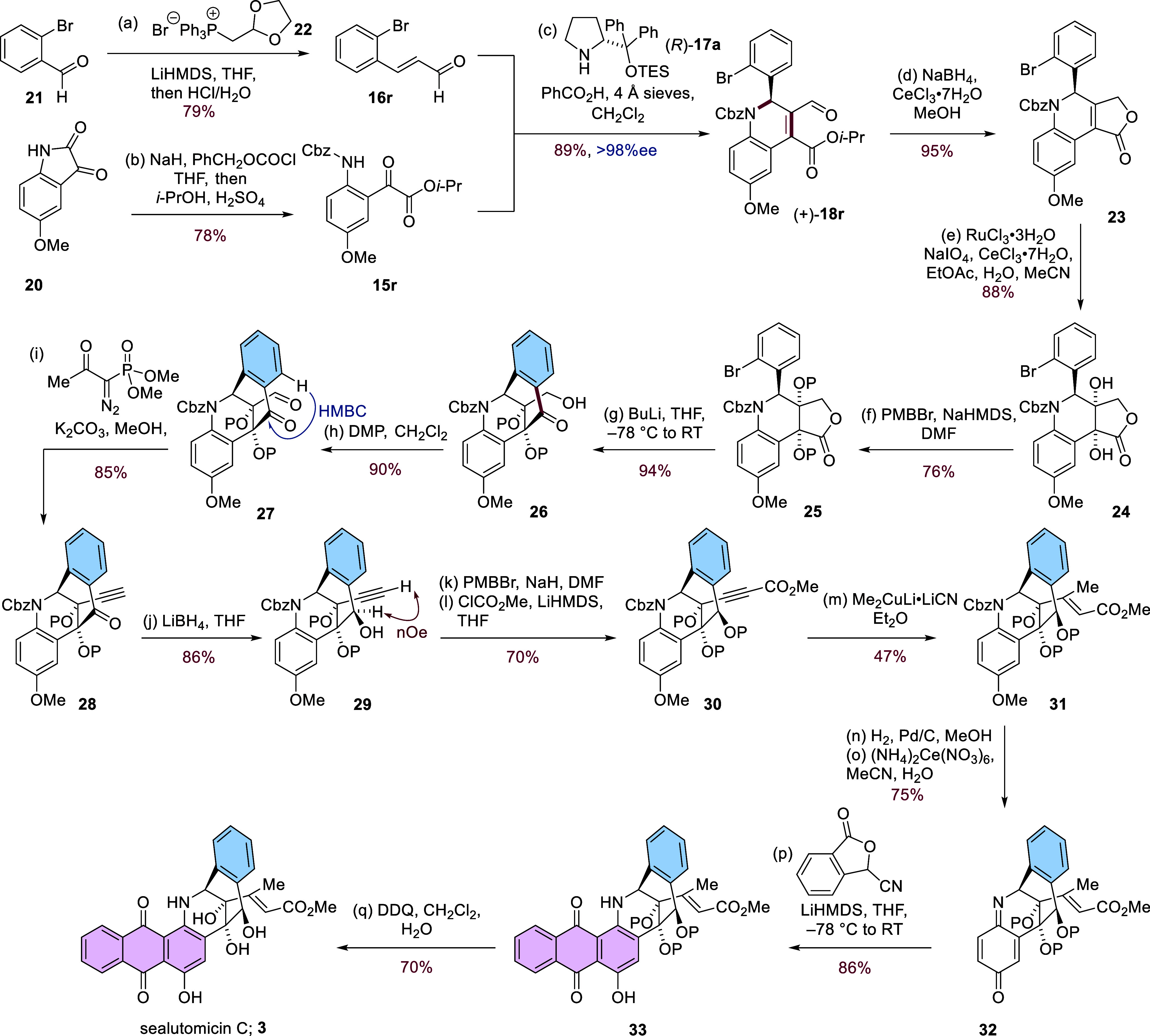
Total Synthesis of Sealutomicin C P = 4-methoxybenzyl. Reagents and conditions: (a) (1,3-dioxolan-2-ylmethyl)triphenylphosphonium bromide **22**, LiHMDS (1.0 M in THF), THF, 0 °C then RT 15 min, then aldehyde **21** was added in THF, refluxed for 40 h, then 2 M HCl was added at RT, 15 h, 79%; (b) NaH, THF, 0 °C, RT 30 min, then benzyl chloroformate was added, RT 3 h, workup, *i*-PrOH, H_2_SO_4_, refluxed, 61 h, 78%; (c) (*R*)-2-{[(triethylsilyl)oxy]diphenylmethyl} pyrrolidine (*R*)-**17a** (20 mol %), 2-bromocinnamaldehyde **16r** (1 equiv), isopropyl 2-(2-{[(benzyloxy)carbonyl]amino}-5-methoxyphenyl)-2-oxoacetate **15r** (2.0 equiv), 4 Å molecular sieves, RT, 72 h, 89%, 98% ee; (d) NaBH_4_, CeCl_3_·7H_2_O, MeOH, RT, 1 h, 95%; (e) NaIO_4_, CeCl_3_·7H_2_O, water, 35 °C, 10 min, then cooled to 0 °C, then EtOAc, MeCN, and RuCl_3_·3H_2_O were added, then γ-lactone **23** was added, 0 °C, 10 min, 88%; (f) NaHMDS (1.0 M in THF, 3.0 equiv), DMF, 0 °C, 30 min, then 4-methoxybenzyl bromide was added, RT, 12 h, 76%; (g) *n*-BuLi, (1.6 M in hexane), THF, −78 °C, 5 min, then warmed to RT, 30 min, 94%; (h) Dess–Martin periodinane, NaHCO_3_, CH_2_Cl_2_, 0 °C then RT, 1 h, 90%; (i) dimethyl (1-diazo-2-oxopropyl)phosphonate, MeOH, RT, 20 h, 85%; (j) LiBH_4_, THF, 0 °C, then RT, 18 h, 86%; (k) NaH, DMF, 0 °C, then RT, 15 min, then 4-methoxybenzyl bromide was added, 14 h, 87%; (l) LiHMDS (1.0 M in toluene), THF, HMPA, 15 min, −78 °C, then methyl chloroformate was added, −78 °C, 2 h, 80%; (m) Cu(I)CN, MeLi (1.7 M in Et_2_O), Et_2_O, −78 °C, then propiolate ester **30** was added in Et_2_O at 0 °C, 3h, 47%; (n) H_2_, Pd/C, MeOH, EtOAc, RT, 3 h, 89%; (o) (NH_4_)_2_Ce(NO_3_)_6_, MeCN, water, RT, 15 min, 84%; (p) 3-cyanophthalide, LiHMDS (1.0 M in toluene), THF, −78 °C, 15 min, then iminoquinone **32** was added in THF, −78 °C to RT, 15 min, 86%; (q) DDQ, CH_2_Cl_2_, water, RT, 16 h, 70%.

The α-ketoester **15r** and bromocinnamaldehyde **16r** required for the key catalytic enantioselective dihydroquinoline synthesis were both prepared on a scale in a single step from cheap, commercially available starting materials. Carbamate protection of 5-methoxyisatin **20** followed by alcoholysis in acidic isopropanol gave **15r** in 78% yield, while Wittig homologation of 2-bromobenzaldehyde **21** using the ylide derived from **22** followed by subsequent acetal hydrolysis gave **16r** in 79% yield (Scheme 1).^41,42^ Under the optimized organocatalytic conditions, using (*R*)-**17a** as the catalyst, dihydroquinoline (+)-**18r** was obtained in 89% on a 7 g scale with an enantiomeric excess of >98%. Subsequent Luche reduction of (+)-**18r** gave α,β-unsaturated lactone **23**.^43^ It was found that the addition of CeCl_3_·7H_2_O was crucial for suppressing unwanted over-reduction to the corresponding saturated lactone. With lactone **23** in hand, we turned our attention to the installation of the 1,2-*syn* diol. Ruthenium(VIII)-catalyzed dihydroxylation of **23** gave the *syn*-diol **24** as a single diastereomer in 88% yield, which was assigned as the (*R*,*R*) diastereomer in the expectation that dihydroxylation had occurred from the less hindered face of the fully substituted alkene (vide infra).^44^ Protection of **24** as the *bis*-paramethoxybenzyl (PMB) ether **25** under basic conditions now set the stage for the crucial cyclization to form the bridged bicyclic core of sealutomicin C. Treatment of bromide **25** in THF with a slight excess of *n*-butyllithium at −78 °C and subsequent warming to room temperature gave the desired ketone **26** in 94% yield on a 1 g scale. By ^1^H NMR, the ketone **26** was formed as a mixture with the corresponding lactol, resulting in a complex spectrum; however, simple oxidation of **26** with the Dess–Martin periodinane gave the aldehyde **27** with a much simplified ^1^H NMR spectrum. Aldehyde **27** showed a ^1^H–^13^C HMBC correlation between the indicated aromatic proton (δ_H_ 7.88 ppm) and the ketone carbonyl carbon (δ_C_ 190 ppm) demonstrating that successful cyclization had occurred which also demonstrated that dihydroxylation had indeed occurred on the less hindered face of the α,β-unsaturated-γ-lactone **23**. The aldehyde **27** was readily converted into the terminal acetylene **28** under standard conditions in the presence of the aromatic ketone.^45,46^ Reduction of the aromatic ketone **28** with lithium borohydride gave the corresponding secondary alcohol **29** as a single diastereomer whose configuration was tentatively assigned by ^1^H–^1^H NOESY analysis and later confirmed by ^1^H–^1^H ROESY analysis of anthraquinone **33**. Following protection of the benzylic alcohol as its PMB ether, the terminal alkyne was carboxylated under basic conditions with methyl chloroformate to give alkynyl ester **30**. Exposure of propargylic ester **30** to an excess of the organocuprate derived from methyllithium and copper(I) cyanide at 0 °C gave alkenyl ester **31** in 47% yield with the double bond configuration assigned through ^1^H–^1^H NOESY and ROESY experiments on later synthetic intermediates. Our endgame strategy centered around the conversion of the *p*-methoxyaniline motif of **31** into a suitable electrophile which could be used in the anthraquinone-forming Hauser–Kraus annulation.^47−49^ To this end, hydrogenolysis using palladium on carbon enabled selective cleavage of the benzyloxycarbamate protecting group to give the corresponding aniline, which on treatment with cerium(IV) ammonium nitrate (CAN) gave iminoquinone **32** in 75% yield over two steps.^50^ Treatment of **32** with the anion generated from 3-cyanophthalide and LiHMDS resulted in rapid iminoquinone consumption, giving anthraquinone **33** in 86% yield; ^1^H–^1^H ROESY analysis of **33** was entirely in keeping with the depicted configuration. Attempts to remove the three PMB ether groups from **33** using Lewis-acidic boron trichloride dimethyl sulfide complex^51^ led to degradation of the starting material which also occurred when attempting the deprotection with CAN. Global deprotection was ultimately achieved upon treatment of **33** with excess 2,3-dichloro-5,6-dicyano-1,4-benzoquinone (DDQ), yielding sealutomicin C **3** in 70% yield. The ^1^H and ^13^C NMR data (DMSO-*d*_6_) for the synthetic sealutomicin C were in good agreement with those reported for the natural product.^1^ Additionally, the circular dichroism spectrum of our synthetic material matched closely with that of the isolated natural product, indicating that the natural enantiomer of sealutomicin C had been synthesized. The ^1^H and ^13^C NMR spectra (acetone-*d*_6_) for synthetic sealutomicin C were in agreement with those reported by Shen and co-workers for the Bergman cyclization product of tiancimycin B **7**, indicating that sealutomicin C and cycloaromatized tiancimycin B have the same structure.

## Conclusions

In conclusion, we have developed the first enantioselective synthesis of sealutomicin C **3** and demonstrated that it has the same structure as the Bergman cyclization product of tiancimycin B. The synthesis proceeded in 16 steps (longest linear sequence) from 5-methoxy isatin **20** and required the development of a methodology for the organocatalytic enantioselective synthesis of dihydroquinolines from α-ketoesters and cinnamaldehydes, as well as the use of a key intramolecular cyclization of an aryllithium onto a γ-lactone. Work toward the total synthesis of other members of the sealutomicins is ongoing.

## Supporting Information Available

Experimental procedures, characterization data, NMR spectra of reported compounds, and CIF files (PDF)
