# Supplementary material for: Enantioselective Synthesis of Sealutomicin C
Source: J Am Chem Soc. 2024 Jun 17;146(26):17757–64. doi: 10.1021/jacs.4c02969 (PMC11228992; doi:10.1021/jacs.4c02969)
Supplement: Supplementary file 1 — ja4c02969_si_001.pdf [file ja4c02969_si_001.pdf]

Supporting Information Experimental Methods and Data

Enantioselective synthesis of sealutomicin C

Stuart M. Astle,[1] Sean Guggiari,[1] James R. Frost,[2] Hamish B. Hepburn,[3] David J. Klauber,[4] Kirsten E. Christensen[1] and Jonathan W. Burton[1]*

[1]Chemistry Research Laboratory, University of Oxford, 12 Mansfield Road, Oxford, OX1 3TA, UK.

[2]UCB Pharma, 216 Bath Road, Slough, Berkshire, SL1 3WE, UK.

[3]Vertex Pharmaceuticals, Abingdon OX14 4RW, U.K.

[4]Chemical Development, Pharmaceutical Technology & Development, Operations, AstraZeneca, Macclesfield, SK10 2NA, UK.

E-mail: jonathan.burton@chem.ox.ac.uk; homepage: https://burton.web.ox.ac.uk

Table of Contents

General Experimental....2

General Procedures....3

Experimental Data....3

Synthesis of α-ketoester substrates **15a** – **15g**, **15i** – **15j** and **15r**....6

Dihydroquinoline synthesis....16

Total Synthesis of Sealutomicin C....29

Circular Dichroism....44

NMR Data Comparison for Synthetic and Natural Sealutomicin C....45

Single Crystal X-ray Diffraction....51

References....54

NMR Spectra....55

HPLC Data....213

General Experimental

^{1}H, ^{13}C and ^{19}F NMR spectra were recorded on Bruker NEO600 (600/151 MHz), Bruker AV 500 (500/125 MHz) and Bruker AV 400 (400/101/377 MHz) spectrometers. Proton, carbon and fluorine chemical shifts are quoted in ppm with ^{1}H, ^{13}C NMR spectra referenced to residual protonated solvent; ^{19}F NMR spectra were referenced relative to $CFCl_3$. Resonances are described as s (singlet), d (doublet), t (triplet), q (quartet), m (multiplet), br (broad), dd (double doublet) and so on. Coupling constants (J) are given in Hz and are rounded to the nearest 0.1 Hz. A number of compounds contain a Cbz protecting group hence broad resonances are frequently observed in both the ^{1}H and ^{13}C NMR spectra due to rotamers.

High resolution mass spectra were recorded by the mass spectrometry staff at the Chemistry Research Laboratory, University of Oxford, using a Bruker Daltronics microTOF spectrometer (ES) or a Micromass GCT (FI). *m/z* values are reported in Daltons. High resolution values are calculated to four decimal places from the molecular formula, all found values being within a tolerance of 5.0 ppm.

Infrared spectra were recorded on a Bruker Tensor 27 Fourier Transform spectrometer using diamond ATR. Absorption maxima (ν_{max}) are described as strong, medium, weak and broad and are quoted in wavenumbers (cm^{-1}).

Optical rotations were measured using a Perkin-Elmer 241 polarimeter in a cell of 1.0 dm path length.

Circular dichroism was measured on an Cirus Applied Photophysics Chirascan^{TM} Spectrophotometer from 210 to 700 nm with a step size of 0.5 nm and a time per point of 1 s.

HPLC analysis was performed using an Agilent 1200 machine with a Chiralcel OD chiral column (4.6 mm × 250 mm) and a Chiralcel AD-H chiral column (4.6 mm × 250 mm). Compounds were detected using wavelengths of 250 nm and 254 nm, with integrations taken from the 254 nm trace. Racemic samples of the chiral dhihydroquinolines were obtained from using pyrrolidine as the catalyst and scalemic samples were prepared or by mixing approximately equal quantities of each enantiomer, prepared *via* identical reaction conditions but with opposite enantiomers of the catalyst.

TLC was performed on Merck DC-Alufolien 60 F254 0.2 mm precoated plates and visualised using an acidic vanillin or basic potassium permanganate dip. Retention factors (R_f) are reported with the solvent system used in parentheses. Flash column chromatography was performed on Merck 60 silica (particle size 40– 63 µm, pore diameter 60 Å) and the solvent system used is recorded in parentheses.

All non-aqueous reactions were carried out in flame-dried glassware under an inert atmosphere of argon or nitrogen and employing standard techniques for handling air-sensitive materials. Solvents and commercially available reagents were dried and purified before use, as appropriate.

Compounds were named using ChemDraw™ and the assignments of NMR spectra were made using MestReNova. ™ The numbering of atoms in the reported data and on the NMR spectra do not necessarily correspond to the IUPAC compound name.

General Procedures

General Procedure A - Optimised organocatalytic cyclisation conditions.

To (*S*)-2-(diphenyl((triethylsilyl)oxy)methyl)pyrrolidine (**(*S*)-17a**, 11.0 mg, 0.03 mmol, 0.2 equiv.) in CH_2Cl_2 (0.15 mL) were added sequentially α,β-unsaturated aldehyde (0.15 mmol, 1.0 equiv.), benzoic acid (9.2 mg, 0.08 mmol, 0.5 equiv.), freshly activated 4 Å molecular sieves (ground) (50 mg), and then keto ester (0.30 mmol, 2.0 equiv.) in CH_2Cl_2 (0.15 mL), then the mixture stirred at room temperature for the stated time. Additional CH_2Cl_2 (0.15 mL) was added after 24 h to compensate for solvent loss. The reaction was filtered and the filter cake washed with ethyl acetate. The filtrate was washed sequentially with sat. aq. sodium bicarbonate solution, hydrochloric acid (2 M), and brine. The organic portion was dried (Na_2SO_4) and concentrated *in vacuo*. The residue was purified by column chromatography to give the title product.

General Procedure B – Cbz protection of isatin derivatives

To the isatin (1.0 equiv.) in tetrahydrofuran at 0 °C was added triethylamine (1.2 equiv.) followed by dropwise benzyl chloroformate (95%, 1.2 equiv.). The resultant solution was stirred at room temperature for the stated time. The reaction was concentrated *in vacuo*, then water was added and the solid was collected by filtration, washing with water, to give the title product.

General Procedure C – Solvolysis of Cbz-protected isatin derivatives with 2-propanol.

Cbz-protected isatin (1.0 equiv.) was dissolved in 2-propanol and heated at reflux for the stated time. The solution was allowed to cool to room temperature and concentrated *in vacuo*. The residue was purified by column chromatography (SiO_2, eluent load) to give the title product.

Experimental Data

(*S*)-Diphenyl(pyrrolidin-2-yl)methanol: (*S*)-17c

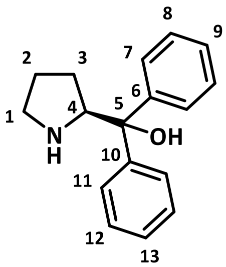

Prepared according to the procedures and Boeckman.[1]

To sodium hydroxide (3.8 g, 95.5 mmol, 1.1 equiv.) in water (95 mL) were added sequentially (*S*)-proline (10.0 g, 86.9 mmol, 1.0 equiv.), and *tert*-butyl alcohol (65 mL). To this solution was added di-*tert*-butyl dicarbonate (19.0 g, 86.9 mmol, 1.0 equiv.) over 20 min and the solution stirred at room temperature for 18 h. The reaction was extracted with pentane (× 2), and then the combined organic portions were extracted with sat. aq. sodium bicarbonate solution (× 3). The combined aqueous fractions were acidified to pH 1 with concentrated hydrochloric acid, then were extracted with diethyl ether (× 4). The combined ethereal portions were washed sequentially with water and brine, dried (Na_2SO_4) and concentrated *in vacuo* to yield a white powdery solid that was used without further purification. The product (14.3 g, 67.4 mmol, 1.0 equiv.) was dissolved in *N*,*N*-dimethylformamide (170 mL) and cooled to 0 °C. Potassium carbonate (10.2 g, 74.1 mmol, 1.1 equiv.) was added and the mixture stirred at 0 °C for 15 min. To this was added iodomethane (9.2 mL, 148.0 mmol, 2.2 equiv.) then the reaction was allowed to warm to room temperature and stirred for 24 h. Water (100 mL) was added and the reaction extracted with diethyl ether (× 4). The combined organic portions were washed sequentially with water (50 mL × 5) and brine, dried (Na_2SO_4) and concentrated *in vacuo* to yield a yellow oil that was used without further purification. Phenylmagnesium bromide (2.7 M diethyl ether solution) (61 mL, 165.7 mmol, 2.5 equiv.) and tetrahydrofuran (100 mL) were added to a flame-dried three-necked round-bottomed flask equipped with a Friedrich's condenser and a pressure-equalising dropping funnel. The previous product (15.2 g, 66.3 mmol, 1.0 equiv.) was dissolved in tetrahydrofuran (130 mL) and transferred to the dropping funnel. This solution was

added dropwise at RT to the Grignard reagent over 45 min then the reaction stirred for 4 h at RT. The solution was cooled to 0 °C and sat. aq. ammonium chloride solution (100 mL) added *via* the dropping funnel over 5 min. Water (100 mL) was added and the layers separated. The aqueous portion was extracted with diethyl ether (100 mL × 3) and the combined organic fractions were dried (Na_2SO_4) and concentrated *in vacuo* to yield a pale yellow solid that was used without further purification. The product (23.2 g, 65.6 mmol, 1.0 equiv.) was dissolved in ethanol (350 mL). Sodium hydroxide (26.3 g, 656.0 mmol, 10.0 equiv.) was added and the reaction heated at reflux for 105 min before being allowed to cool to room temperature. The reaction was concentrated *in vacuo* then water (130 mL) and diethyl ether (130 mL)were added and the layers separated. The aqueous phase was extracted with diethyl ether (130 mL × 2) then the combined organic portions were washed sequentially with water (130 mL) and brine (130 mL). The organic fraction was dried (Na_2SO_4) and concentrated *in vacuo*. The residue was dissolved in boiling hexane (130 mL) and activated charcoal (1.6 g) was added. After hot filtration of the mixture, with the filter funnel kept hot by boiling hexane (20 mL) in the receiving conical flask, the solution was allowed to cool to room temperature and left until crystallisation occurred. The flask was cooled to 0 °C, the solvent decanted, and the crystals washed with ice cold hexane (20 mL × 3) and dried *in vacuo* to give the title compound as a white crystalline solid (8.9 g). The filtrate was concentrated *in vacuo*, dissolved in boiling hexane (50 mL), and cooled to yield a second batch of crystals (4.0 g) to give a combined yield (12.9 g, 51.0 mmol, 59% from L-proline).

R_f: 0.1 (100% ethyl acetate).

m.p.: 76.0 – 78.0 °C

ν_{max}/cm^{-1} (thin film): 3351 (br), 3085, 3058, 3026, 2969, 2946, 2871, 1598

δ_H(500 MHz, $CDCl_3$): 7.63 – 7.57 (2H, m, 2 x Ar**H**), 7.56 – 7.49 (2H, m, 2 x Ar**H**), 7.35 – 7.26 (4H, m, 4 x Ar**H**), 7.21 – 7.14 (2H, m, 2 x Ar**H**), 4.27 (1H, t, *J* = 7.5 Hz, C(4)**H**), 3.04 (1H, ddd, *J* = 9.0, 6.5, 5.0 Hz, C(1)**H**H), 2.95 (1H, dt, *J* = 9.0, 7.5 Hz, C(1)H**H'**), 1.82 – 1.54 (4H, m, C(2)$\mathbf{H_2}$, C(3)$\mathbf{H_2}$)

δ_C(126 MHz, $CDCl_3$): 148.3 (**Ar**), 145.5 (**Ar**), 128.3 (**Ar**), 128.1 (**Ar**), 126.6 (**Ar**), 126.4 (**Ar**), 126.0 (**Ar**), 125.6 (**Ar**), 77.2 (**C**(5)), 64.6 (**C**(4)), 46.9 (**C**(1)), 26.4 (**C**(2)), 25.6 (**C**(3))

$[\alpha]_D^{25}$ = –68.7 (c = 1.0, $CHCl_3$); lit.[2] $[\alpha]_D^{25}$ = –69 (c = 1.0, $CHCl_3$)

Data in accordance with literature.[1]

A sample of the enantiomer, (*R*)-diphenyl(pyrrolidin-2-yl)methanol (**(*R*)-16c** 5.20 g, 20.3 mmol, 47%), was prepared as above using (*R*)-proline as starting material.

$[\alpha]_D^{25}$ = +71.0 (c = 1.0, $CHCl_3$); lit.[3] $[\alpha]_D^{25}$ = +67.9 (c = 3.4, $CHCl_3$)

All other characterisation identical.

(*S*)-2-(Diphenyl((triethylsilyl)oxy)methyl)pyrrolidine: (*S*)-17a

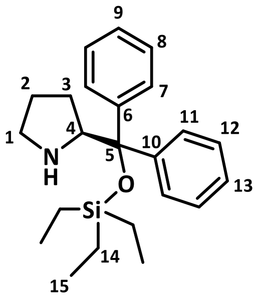

Prepared according to the procedure of Hayashi[4]

To a solution of (*S*)-diphenyl(pyrrolidin-2-yl)methanol (**(*S*)-17c**, 2.00 g, 7.90 mmol, 1.0 equiv.) in dichloromethane (35 mL) at –78 °C was added triethylamine (2.0 mL, 14.2 mmol, 1.8 equiv.) and trimethylsilyl triflate (3.2 mL, 14.2 mmol, 1.8 equiv.). The resultant solution was warmed to RT and stirred for 4 h then quenched by dropwise addition of sat. aq. sodium bicarbonate (40 mL). The layers were separated and the aqueous layer was extracted with dichloromethane (x 3). The combined organic layers were dried over sodium sulfate, concentrated *in vacuo*

and purified by flash column chromatography (10% to 60% diethyl ether in pentane) to give the title compound as a colourless oil (2.46 g, 6.72 mmol, 85%).

R_f: 0.3 (60% diethyl ether in pentane).

ν_{max}/cm^{-1} (thin film): 2956 (br, m), 2910 (m), 2876 (m), 1492 (m), 1446 (m), 1071 (m), 739 (m), 701 (s).

δ_H(600 MHz, $CDCl_3$): 7.52 – 7.48 (2H, m, 2 x Ar**H**), 7.41 – 7.36 (2H, m, 2 x Ar**H**), 7.32 – 7.22 (6H, m, 6 x Ar**H**), 4.04 (1H, t, *J* = 7.5 Hz, C(4)**H**), 2.85 (1H, ddd, *J* = 10.0, 7.5, 6.0 Hz, C(1)**H**H'), 2.72 (1H, ddd, *J* = 10.0, 7.5, 6.0 Hz, C(1)H**H'**), 1.80 (1H, br s, N**H**), 1.65 – 1.51 (3H, m, C(2)**HH'**, C(3)**H**H), 1.32 – 1.23 (1H, m, C(3)H**H'**), 0.88 (9H, t, *J* = 8.0 Hz, 3 x C(15)**H₃**), 0.38 (6H, q, *J* = 8.0 Hz, 3 x C(14)**H₂**).

δ_C(151 MHz, $CDCl_3$): 146.8 (**Ar**), 145.6 (**Ar**), 129.0 (**Ar**), 128.2 (**Ar**), 127.7 (**Ar**), 127.5 (**Ar**), 127.1 (**Ar**), 127.1 (**Ar**), 83.1 (**C**(5)), 65.8 (**C**(4)), 47.4 (**C**(1)), 27.9 (**C**(3)), 25.3 (**C**(2)), 7.4 (**C**(15)), 6.7 (**C**(14)).

m/z HRMS (ESI$^+$) found 368.2403; $C_{23}H_{34}NO_9Si^+$ (M+H$^+$) requires 368.2404.

$[\alpha]_D^{25}$ = −30.9 (c = 0.54, $CHCl_3$)); lit.[4] $[\alpha]_D^{30}$ = −48.4 (c = 0.097 $CHCl_3$); lit.[5] $[\alpha]_D^{30}$ = −29.6 (c = 1.0, $CHCl_3$).

Data in accordance with literature.[4]

A sample of the enantiomer, (*R*)-2-(diphenyl((triethylsilyl)oxy)methyl)pyrrolidine (**(*R*)-17a**, 2.97 g, 8.09 mmol, 82%), was prepared as above using (*R*)-diphenyl(pyrrolidin-2-yl)methanol (**(*R*)-17c**) as starting material.

$[\alpha]_D^{25}$ = +31.1 (c = 0.62, $CHCl_3$)

All other characterisation identical.

(*S*)-2-(Diphenyl((trimethylsilyl)oxy)methyl)pyrrolidine: (*S*)-17b

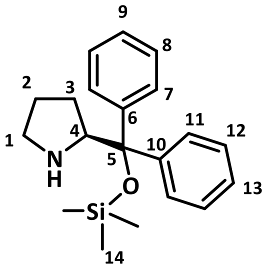

Prepared according to the procedure of Boeckman[1]

To (*S*)-diphenyl(pyrrolidin-2-yl)methanol (**(*S*)-17c**, 1.0 g, 3.9 mmol, 1.0 equiv.) in CH_2Cl_2 (20 mL) at −78 °C was added triethylamine (0.8 mL, 5.5 mmol, 1.4 equiv.) followed by dropwise trimethylsilyl triflate (1.0 mL, 5.5 mmol, 1.4 equiv.) over 10 min and the mixture allowed to warm to room temperature over 2 h. The reaction was stirred for a further 2 h. To the solution was added sequentially sat. aq. sodium bicarbonate solution (6 mL) and water (6 mL) then the mixture was extracted with CH_2Cl_2 (× 3). The combined organic portions were dried (Na_2SO_4) and concentrated *in vacuo*. The residue was purified by flash column chromatography (30% to 70% diethyl ether in pentane) to give the title compound as a viscous yellow oil (940 mg, 2.9 mmol, 73%).

R_f: 0.1 (100% ethyl acetate).

ν_{max}/cm^{-1} (thin film): 3086, 3059, 3025, 2954, 2874, 1599

δ_H(500 MHz, $CDCl_3$): 7.48 – 7.44 (2H, m, 2 x Ar**H**), 7.38 – 7.32 (2H, m, 2 x Ar**H**), 7.30 – 7.18 (6H, m 6 x Ar**H**), 4.03 (1H, t, *J* = 7.5 Hz, C(4)**H**), 2.90 – 2.82 (1H, m, C(1)**H**H'), 2.82 – 2.75 (1H, m, C(1)H**H'**), 1.69 (1H, br s, N**H**), 1.64 – 1.47 (3H, m, C(2)**H**H', C(3)**HH'**), 1.43 – 1.32 (1H, m, C(2)H**H'**), -0.09 (9H, s, C(14)**H₃**)

δ_C(126 MHz, $CDCl_3$): 147.0 (**Ar**), 145.9 (**Ar**), 128.5 (**Ar**), 127. (**Ar**)7, 127.7 (**Ar**), 127.7 (**Ar**), 127.0 (**Ar**), 126.8 (**Ar**), 83.3 (**C**(5)), 65.5 (**C**(4)), 47.3 (**C**(1)), 27.6 (**C**(3)), 25.2 (**C**(2)), 2.3 (**C**(14))

$[\alpha]_D^{25}$ = −51.1 (c = 1.0, $CHCl_3$); lit.[4] $[\alpha]_D^{25}$ = −52.4 (c = 1.04, $CHCl_3$)

Data in accordance with literature.[4]

Synthesis of α-ketoester substrates 15a – 15g, 15i – 15j and 15r

Benzyl 2,3-dioxoindoline-1-carboxylate: S1

Prepared by the procedure of Suzuki.[6]

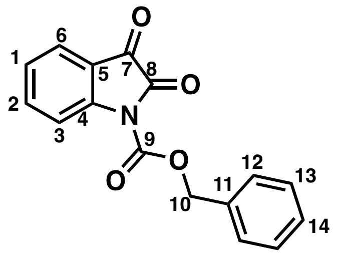

Isatin (5.0 g, 34.0 mmol, 1.0 equiv.), tetrahydrofuran (60 mL), triethylamine (5.7 mL, 40.8 mmol, 1.2 equiv.), and benzyl chloroformate (95%) (6.0 mL, 40.8 mmol, 1.2 equiv.) were subjected to **General Procedure B**, stirring for 90 min to give benzyl 2,3-dioxoindoline-1-carboxylate (**S1**) as a bright yellow solid (9.1 g, 32.4 mmol, 95%).

R_f: 0.1 (10% MeOH in CH_2Cl_2)

m.p.: 153.5–155.5 °C (lit.[121] 153–154 °C)

ν_{max} /cm^{-1} (thin film): 1793, 1732, 1612, 1595.

δ_H(500 MHz, DMSO-*d*$_6$): 8.00 (1H, dt, *J* = 8.0, 1.0 Hz, C(3)**H**), 7.77 (1H, ddd, *J* = 8.0, 7.5, 1.5 Hz, C(2)**H**), 7.72 (1H, ddd, *J* = 7.5, 1.5, 0.5 Hz, C(6)**H**), 7.55 – 7.50 (2H, m, 2 × C(12)**H**), 7.46 – 7.40 (2H, m, 2 × C(13)**H**), 7.39 – 7.35 (1H, m, C(14)**H**), 7.34 (1H, td, *J* = 7.5, 1.0 Hz, C(1)**H**), 5.46 (2H, s, C(10)**H$_2$**).

δ_C(126 MHz, DMSO-*d*$_6$): 179.5 (**C**(7)), 155.9 (**C**(8)), 149.9 (**C**(9)), 147.1 (**C**(4)), 137.8 (**C**(2)), 135.3 (**C**(11)), 128.5 (2 × **C**(13)), 128.3 (**C**(14)), 127.9 (2 × **C**(12)), 125.1 (**C**(1)), 124.4 (**C**(6)), 119.7 (**C**(5)), 116.1 (**C**(3)), 68.0 (**C**(10)).

m/z HRMS (CI, NH_3): 299.1024; $C_{16}H_{15}N_2O_4^+$ (M+NH_4^+) requires 299.1026

Data in accordance with literature.[6]

Isopropyl 2-(2-(((benzyloxy)carbonyl)amino)phenyl)-2-oxoacetate: 15a

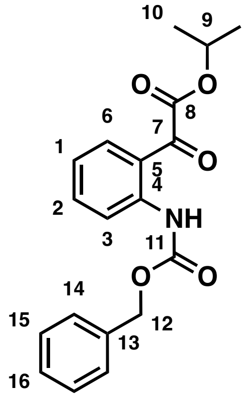

Benzyl 2,3-dioxoindoline-1-carboxylate (**S1**) (5.0 g, 17.8 mmol, 1.0 equiv.) and 2-propanol (160 mL) were subjected to **General Procedure C**, heating for 6 h. Purification by column chromatography (20% diethyl ether in petroleum ether) gave isopropyl 2-(2-(((benzyloxy)carbonyl)amino)phenyl)-2-oxoacetate (**15a**) as a viscous yellow oil that solidified on standing in the freezer (5.9 g, 17.3 mmol, 97%).

R_f: 0.3 (8% ethyl acetate in petroleum ether).

m.p.: 38.5–40.5 °C.

ν_{max} /cm^{-1} (thin film): 3295 (br), 3035, 2984, 2937, 1734, 1649, 1608, 1583, 1528.

δ_H(500 MHz, $CDCl_3$): 10.69 (1H, s, N**H**), 8.55 (1H, dd, *J* = 8.5, 1.0 Hz, C(3)**H**), 7.66 (1H, ddd, *J* = 8.0, 1.5, 0.5 Hz, C(6)**H**), 7.63 (1H, dddd, *J* = 8.5, 7.5, 1.5, 0.5 Hz, C(2)**H**), 7.44 – 7.40 (2H, m, 2 × C(14)**H**), 7.40 – 7.36 (2H, m,

2 × C(15)**H**), 7.36 – 7.32 (1H, m, C(16)**H**), 7.10 (1H, ddd, *J* = 8.0, 7.5, 1.0 Hz, C(1)**H**), 5.32 (1H, hept, *J* = 6.5 Hz, C(9)**H**), 5.23 (2H, s, C(12)$\mathbf{H_2}$), 1.41 (6H, d, *J* = 6.5 Hz, 2 × C(10)$\mathbf{H_3}$).

δ_C(126 MHz, $CDCl_3$): 190.5 (**C**(7)), 163.4 (**C**(8)), 153.5 (**C**(11)), 143.1 (**C**(4)), 137.0 (**C**(2H), 136.0 (**C**(13)), 133.6 (C(**6**)), 128.7 (2 × **C**(15)), 128.5 (**C**(16)), 128.4 (2 × **C**(14)), 121.9 (**C**(1)), 119.4 (**C**(3)), 117.0 (**C**(5)), 71.1 (**C**(9)), 67.3 (**C**(12)), 21.8 (2 × **C**(10)).

m/z HRMS (ESI): found 340.1192; $C_{19}H_{18}NO_5^-$ (M–H)$^-$ requires 340.1191.

Methyl 2-(2-(((benzyloxy)carbonyl)amino)phenyl)-2-oxoacetate: 15b

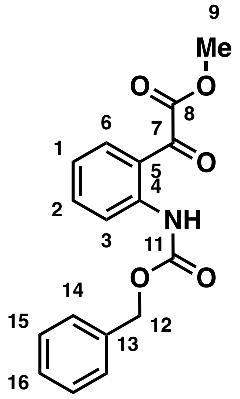

Benzyl 2,3-dioxoindoline-1-carboxylate (**S1**) (3.9 g, 13.9 mmol, 1.0 equiv.) was dissolved in methanol (50 mL) and heated at reflux for 90 min. The solution was allowed to cool to room temperature and was then cooled to 0 °C. The crystals were collected by filtration, washing with ice cold methanol, and dried *in vacuo* to give methyl 2-(2-(((benzyloxy)carbonyl)amino)phenyl)-2-oxoacetate (**15b**) as a pale yellow solid (2.9 g, 9.4 mmol, 68%). The filtrate was concentrated *in vacuo*, and then methanol (16 mL) added and the mixture heated at reflux for 90 min. Cooling to room temperature then to 0 °C provided a second batch of crystalline product identical to the first (0.6 g, 1.9 mmol, 13%) to give the combined yield (3.5 g, 11.3 mmol, 81%).

R_f: 0.4 (20% diethyl ether in petroleum ether)

m.p.: 86.5–88.0 °C.

ν_{max} /cm^{-1} (thin film): 3298 (br), 2955, 2924, 2853, 1736, 1650, 1607, 1583, 1526.

δ_H(500 MHz, DMSO-d_6): 10.15 (1H, s, N**H**), 7.83 – 7.77 (1H, m, C(3)**H**), 7.73 – 7.66 (2H, m, C(2)**H**, C(6)**H**), 7.44 – 7.38 (4H, m, 2 × C(14)**H**, 2 × C(15**H**), 7.37 – 7.33 (1H, m, C(16)**H**), 7.28 (1H, ddd, *J* = 8.0, 7.5, 1.0 Hz, C(1)**H**), 5.15 (2H, s, C(12)$\mathbf{H_2}$), 3.83 (3H, s, C(9)$\mathbf{H_3}$).

δ_C(126 MHz, DMSO-d_6): 187.3 (**C**(7)), 163.1 (**C**(8)), 153.7 (**C**(11)), 139.5 (**C**(4)), 136.1 (**C**(13)), 135.3 (**C**(2)), 131.8 (**C**(6)), 128.5 (2 × **C**(14)), 128.2 (**C**(16)), 128.2 (2 × **C**(15)**H**), 123.7 (**C**(1)), 123.1 (**C**(5)), 121.5 (**C**(3)), 66.6 (**C**(12)), 53.0 (**C**(9)).

m/z HRMS (ESI): 312.0875; $C_{17}H_{14}NO_5^-$ (M–H)$^-$ requires 312.0878.

Methyl 2,3-dioxoindoline-1-carboxylate: S2

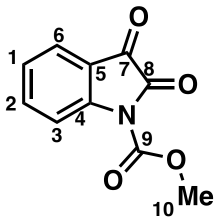

To isatin (500 mg, 3.4 mmol, 1.0 equiv.) in tetrahydrofuran (10 mL) at 0 °C was added triethylamine (0.57 mL, 4.1 mmol, 1.2 equiv.) followed by dropwise methyl chloroformate (0.32 mL, 4.1 mmol, 1.2 equiv.) and the mixture was stirred at RT for 60 min. The reaction was concentrated *in vacuo*, then water added and the solid

collected by filtration, washing with water, to give methyl 2,3-dioxoindoline-1-carboxylate (**S2**) as a bright yellow solid (608 mg, 3.0 mmol, 87%).

R_f: 0.1 (10% methanol in CH_2Cl_2).

m.p.: 181.5–183.5 °C (lit.[7] 179–180 °C).

ν_{max} /cm^{-1} (thin film): 3140, 3035, 2966, 1784, 1744, 1728, 1614, 1596.

δ_H(500 MHz, DMSO-d_6): 7.99 (1H, dt, *J* = 8.5, 1.0 Hz, C(3)**H**), 7.78 (1H, ddd, *J* = 8.5, 7.5, 1.5 Hz, C(2)**H**), 7.72 (1H, ddd, *J* = 7.5, 1.5, 0.5 Hz, C(6)**H**), 7.34 (1H, td, *J* = 7.5, 0.1 Hz, C(1)**H**), 3.94 (3H, s, C(10)**H$_3$**).

δ_C(126 MHz, DMSO-d_6): 179.6 (**C**(7)), 155.8 (**C**(8)), 150.5 (**C**(9)), 147.2 (**C**(4)), 137.8 (**C**(2)), 125.1 (**C**(1)), 124.4 (**C**(6)), 119.6 (**C**(5)), 116.2 (**C**(3)), 53.9 (**C**(10)).

m/z HRMS (ESI): 228.0264; $C_{10}H_7NNaO_4^+$ $(M+Na)^+$ requires 228.0267.

Isopropyl 2-(2-((methoxycarbonyl)amino)phenyl)-2-oxoacetate: 15c

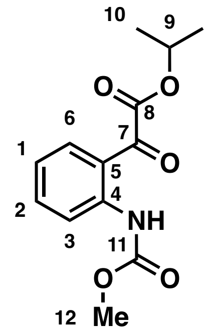

Methyl 2,3-dioxoindoline-1-carboxylate (**S2**) (300 mg, 1.5 mmol, 1.0 equiv.) was dissolved in 2-propanol (15 mL) and heated at reflux for 6.5 h. The solution was allowed to cool to room temperature and concentrated *in vacuo*. The residue was purified by column chromatography (20% diethyl ether in petroleum ether) to give isopropyl 2-(2-((methoxycarbonyl)amino)phenyl)-2-oxoacetate (**15c**) as a pale yellow solid (335 mg, 1.3 mmol, 86%).

R_f: 0.5 (30% diethyl ether in petroleum ether).

m.p.: 56.5–58.0 °C.

ν_{max} /cm^{-1} (thin film): 3299 (br), 2985, 2956, 1735, 1650, 1608, 1583, 1528;

δ_H(500 MHz, $CDCl_3$): 10.62 (1H, s, N**H**), 8.52 (1H, dd, *J* = 8.5, 1.0 Hz, C(3)**H**), 7.68 – 7.59 (2H, m, C(2)**H**, C(6)**H**), 7.09 (1H, ddd, *J* = 8.0, 7.5, 1.0 Hz, C(1)**H**), 5.32 (1H, hept, *J* = 6.5 Hz, C(9)**H**), 3.80 (3H, s, C(12)**H$_3$**), 1.41 (6H, d, *J* = 6.5 Hz, 2 × C(10)**H$_3$**).

δ_C(126 MHz, $CDCl_3$): 190.5 (**C**(7)), 163.5 (**C**(8)), 154.1 (**C**(11)), 143.2 (**C**(4)), 136.9 (**C**(2)), 133.5 (**C**(6)), 121.8 (**C**(1)), 119.3 (**C**(3)), 117.0 (**C**(5)), 71.1 (**C**(9)), 52.7 (**C**(12)), 21.8 (2 × **C**(10)).

m/z HRMS (ESI): 266.1023; $C_{13}H_{16}NO_5^+$ $(M+H^+)$ requires 266.1023.

Isopropyl 2-(2-((*tert*-butoxycarbonyl)amino)phenyl)-2-oxoacetate: 15d

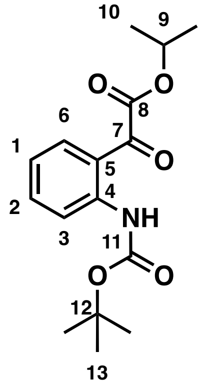

To isatin (500 mg, 3.40 mmol, 1.0 equiv.) in tetrahydrofuran (8 mL) at 0 °C was added 4-(dimethylamino)pyridine (42 mg, 0.34 mmol, 0.1 equiv.) followed by dropwise di-*tert*-butyl dicarbonate (890 mg, 4.10 mmol, 1.2 equiv.) in tetrahydrofuran (2 mL). The solution was stirred at room temperature for 4 h, and then the solvent was removed *in vacuo* to give a yellow solid that was used without further purification. To a portion of the product (200 mg, 0.77 mmol, 1.0 equiv.) was added 2-propanol and the mixture heated at reflux for 7 h. The reaction was allowed to cool and concentrated *in vacuo*. The residue was purified by column chromatography (10% diethyl ether in petroleum ether) to give isopropyl 2-(2-((*tert*-butoxycarbonyl)amino)phenyl)-2-oxoacetate (**15d**) as a viscous yellow oil that solidified on standing in the freezer (206 mg, 0.67 mmol, 74% from isatin).

R_f: 0.5 (10% diethyl ether in petroleum ether).

ν_{max} /cm^{-1} (thin film): 3305 (br), 2983, 2936, 1732, 1650, 1608, 1580, 1525.

δ_H(500 MHz, $CDCl_3$): 10.43 (1H, s, N**H**), 8.52 (1H, dd, *J* = 8.5, 1.0 Hz, C(3)**H**), 7.64 (1H, ddd, *J* = 8.0, 1.5, 0.5 Hz, C(6)**H**), 7.60 (1H, dddd, *J* = 8.5, 7.0, 1.5, 0.5 Hz, C(2)**H**), 7.05 (1H, ddd, *J* = 8.0, 7.0, 1.0 Hz, C(1)**H**), 5.32 (1H, hept, *J* = 6.5 Hz, C(9)**H**), 1.53 (9H, s, 3 × C(13)**H₃**), 1.40 (6H, d, *J* = 6.5 Hz, 2 × C(10)**H₃**).

δ_C(126 MHz, $CDCl_3$): 190.5 (**C**(7)), 163.6 (**C**(8)), 152.8 (**C**(11)), 143.8 (**C**(4)), 136.9 (**C**(2)), 133.5 (**C**(6)), 121.3 (**C**(1)), 119.3 (**C**(3)), 116.8 (**C**(5)), 81.3 (**C**(12)), 71.0 (**C**(9)), 28.4 (3 × **C**(13)), 21.8 (2 × **C**(10)).

m/z HRMS (ESI): 330.1311; $C_{16}H_{21}NNaO_5^+$ (M+H$^+$) requires 330.1312.

Benzyl 5-chloro-2,3-dioxoindoline-1-carboxylate: S3

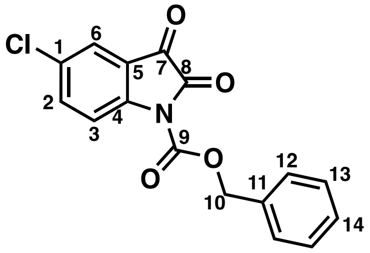

5-Chloroisatin (420 mg, 2.3 mmol, 1.0 equiv.), tetrahydrofuran (10 mL), triethylamine (0.38 mL, 2.8 mmol, 1.2 equiv.), and benzyl chloroformate (95%) (0.41 mL, 2.8 mmol, 1.2 equiv.) were subjected to **General Procedure B**, stirring for 90 min to give benzyl 5-chloro-2,3-dioxoindoline-1-carboxylate (**S3**) as a yellow solid (673 mg, 2.13 mmol, 93%).

R_f: 0.1 (10% methanol in dichloromethane).

m.p.: 168.0–170.0 °C.

ν_{max} /cm^{-1} (thin film): 3136, 3075, 3035, 1799, 1774, 1752, 1724, 1605, 1589;

δ_H(500 MHz, DMSO-d_6): 8.01 (1H, dd, J = 8.5, 0.5 Hz, C(3)**H**), 7.82 (1H, dd, J = 8.5, 2.5 Hz, C(2)**H**), 7.79 (1H, dd, J = 2.5, 0.5 Hz, C(6)**H**), 7.54 – 7.50 (2H, m, 2 × C(12)**H**), 7.45 – 7.40 (2H, m, 2 × C(13)**H**), 7.40 – 7.35 (1H, m, C(14)**H**), 5.47 (2H, s, C(10)**H₂**).

δ_C(126 MHz, DMSO-d_6): δ 178.4 (**C**(7)), 155.4 (**C**(8)), 149.8 (**C**(9)), 145.5 (**C**(4)), 136.7 (**C**(2)), 135.2 (**C**11)), 129.5 (**C**(1)), 128.5 (2 × **C**(13)), 128.3 (**C**(14)), 127.9 (2 × **C**(12)), 123.8 (**C**(6)), 121.2 (**C**(5)), 117.9 (**C**(3)), 68.2 (**C**(10)).

m/z HRMS (ESI): 338.0191; $C_{16}H_{10}{}^{35}ClNNaO_4^+$ (M+Na$^+$) requires 338.0191.

Isopropyl 2-(2-(((benzyloxy)carbonyl)amino)-5-chlorophenyl)-2-oxoacetate: 15e

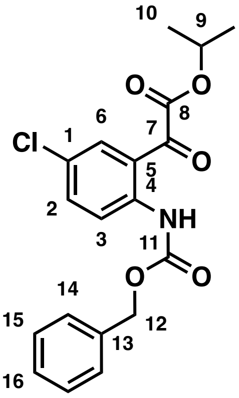

Benzyl 5-chloro-2,3-dioxoindoline-1-carboxylate (**S3**, 600 mg, 1.9 mmol, 1.0 equiv.) and 2-propanol (20 mL) were subjected to **General Procedure C**, heating for 7 h. Purification by column chromatography (10% diethyl ether in petroleum ether) gave isopropyl 2-(2-(((benzyloxy)carbonyl)amino)-5-chlorophenyl)-2-oxoacetate (**15e**) as a pale yellow solid (521 g, 1.4 mmol, 73%).

R_f: 0.4 (10% diethyl ether in petroleum ether).

m.p.: 87.0–88.5 °C.

ν_{max} /cm^{-1} (thin film): 3295 (br), 3066, 3035, 2986, 2938, 1735, 1661, 1579, 1516;

δ_H(500 MHz, CDCl$_3$): 10.58 (1H, s, N**H**), 8.54 (1H, d, J = 9.0 Hz, C(3)**H**), 7.66 (1H, d, J = 2.5 Hz, C(6)**H**), 7.57 (1H, dd, J = 9.0, 2.5 Hz, C(2)**H**), 7.44 – 7.32 (5H, m, 2 × C(14)**H**, 2 × C(15)**H**, C(16)**H**), 5.33 (1H, hept, J = 6.5 Hz, C(9)**H**), 5.22 (2H, s, C(12)**H₂**), 1.42 (6H, d, J = 6.5 Hz, 2 × C(10)**H₃**).

δ_C(126 MHz, CDCl$_3$): 189.3 (**C**(7)), 162.7 (**C**(8)), 153.3 (**C**(11)), 141.6 (**C**(4)), 136.7 (**C**(2)), 135.7 (**C**(13)), 132.7 (**C**(6)), 128.8 (2 × **C**(15)), 128.6 (**C**(16)), 128.4 (2 × **C**(14)), 127.0 (**C**(1)), 121.0 (**C**(3)), 118.1 (**C**(5)), 71.6 (**C**(9)), 67.5 (**C**(12)), 21.8 (2 × **C**(10)).

m/z HRMS (ESI): 376.0946; $C_{19}H_{19}{}^{35}ClNO_5^+$ (M+H$^+$) requires 376.0946.

Benzyl 5-methyl-2,3-dioxoindoline-1-carboxylate (S4)

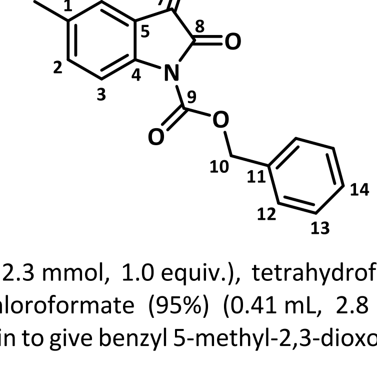

5-Methylindoline-2,3-dione (371 mg, 2.3 mmol, 1.0 equiv.), tetrahydrofuran (10 mL), triethylamine (0.38 mL, 2.8 mmol, 1.2 equiv.), and benzyl chloroformate (95%) (0.41 mL, 2.8 mmol, 1.2 equiv.) were subjected to **General Procedure B**, stirring for 90 min to give benzyl 5-methyl-2,3-dioxoindoline-1-carboxylate (**S4**) as a brown solid (579 mg, 2.0 mmol, 85%).

R_f: 0.1 (10% methanol in dichloromethane).

m.p.: 159.5–161.5 °C.

ν_{max} /cm^{-1} (thin film): 3063, 3032, 2952, 2925, 1784, 1723, 1655, 1620, 1527.

δ_H(500 MHz, DMSO-d_6): 7.88 (1H, dt, J = 8.5, 0.5 Hz, C(3)**H**), 7.58 (1H, ddd, J = 8.5, 2.0, 1.0 Hz, C(2)**H**), 7.54 – 7.49 (3H, m, C(6)**H**, 2 × C(12)**H**), 7.45 – 7.40 (2H, m, 2 × C(13)**H**), 7.40 – 7.35 (1H, m, C(14)**H**), 5.44 (2H, s, C(10)**H₂**), 2.34 (3H, t, J = 1.0 Hz, C(15)**H₃**).

δ_C(126 MHz, DMSO-d_6): 179.6 (**C**(7)), 156.0 (**C**(8)), 149.8 (**C**(9)), 145.1 (**C**(4)), 138.3 (**C**(2)), 135.3 (**C**(11)), 134.7 (**C**(1)), 128.5 (2 × **C**(13)), 128.3 (**C**(14)), 127.9 (2 × **C**(12)), 124.4 (**C**(6)), 119.6 (**C**(5)), 116.0 (**C**(3)), 68.0 (**C**(10)), 20.1 (**C**(15)).

m/z HRMS (ESI): 318.0739; $C_{17}H_{13}NNaO_4^+$ (M+Na$^+$) requires 318.0737.

Isopropyl 2-(2-(((benzyloxy)carbonyl)amino)-5-methylphenyl)-2-oxoacetate: 15f

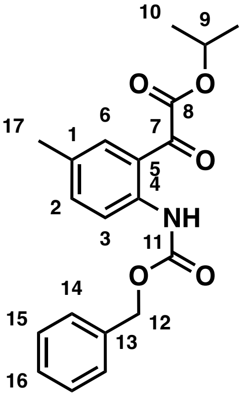

Benzyl 5-methyl-2,3-dioxoindoline-1-carboxylate (**S4**, 500 mg, 1.7 mmol, 1.0 equiv.) and 2-propanol (17 mL) were subjected to **General Procedure C**, heating for 23 h. Purification by column chromatography (15% diethyl ether in petroleum ether) gave the title compound as a pale yellow solid (528 mg, 1.5 mmol, 88%).

R_f: 0.5 (20% diethyl ether in petroleum ether)

m.p.: 73.0–75.0 °C.

ν_{max} /cm^{-1} (thin film): 3298 (br), 3034, 2983, 2939, 1733, 1651, 1586, 1524.

δ_H(500 MHz, $CDCl_3$): 10.58 (1H, s, N**H**), 8.43 (1H, d, J = 8.5 Hz, C(3)**H**), 7.48 – 7.31 (7H, m, C(2)**H**, C(6)**H**, 2 × C(14)**H**, 2 × C(15)**H**, C(16)**H**), 5.33 (1H, hept, J = 6.5 Hz, C(9)**H**), 5.22 (2H, s, C(12)**H₂**), 2.33 (3H, t, J = 1.0 Hz, C(17)**H₃**), 1.41 (6H, d, J = 6.5 Hz, 2 × C(10)**H₃**).

δ_C(126 MHz, $CDCl_3$): 190.5 (**C**(7)), 163.5 (**C**(8)), 153.5 (**C**(11)), 140.8 (**C**(4)), 137.9 (**C**(2)), 136.0 (**C**(13)), 133.4 (**C**(6)), 131.4 (**C**(1)), 128.7 (2 × **C**(15)), 128.4 (**C**(16)), 128.3 (2 × **C**(14)), 119.4 (**C**(3)), 117.0 (**C**(5)), 71.0 (**C**(9)), 67.2 (**C**(12)), 21.8 (2 × **C**(10)), 20.7 (**C**(17)).

m/z HRMS (ESI): 356.1494; $C_{20}H_{22}NO_5^+$ (M+H$^+$) requires 356.1493.

Benzyl 7-fluoro-2,3-dioxoindoline-1-carboxylate: S5

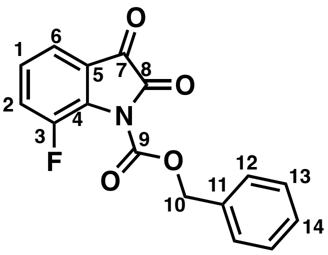

7-Fluoroisatin (380 mg, 2.3 mmol, 1.0 equiv.), tetrahydrofuran (10 mL), triethylamine (0.38 mL, 2.8 mmol, 1.2 equiv.), and benzyl chloroformate (95%) (0.41 mL, 2.8 mmol, 1.2 equiv.) were subjected to **General Procedure B**, stirring for 90 min to give benzyl 7-fluoro-2,3-dioxoindoline-1-carboxylate (**S5**) as an orange solid (578 mg, 1.9 mmol, 84%);

R_f: 0.1 (10% methanol in dichloromethane).

m.p.: 155.0–157.0 °C.

ν_{max} /cm^{-1} (thin film): 3082, 3037, 2962, 1802, 1746, 1621.

δ_H(500 MHz, DMSO-d_6): 7.68 (ddd, J = 11.5, 8.5, 1.0 Hz, C(2)**H**), 7.59 (1H, dd, J = 7.5, 1.0 Hz, C(6)**H**), 7.51 – 7.49 (2H, m, 2 × C(12)**H**), 7.44 – 7.35 (4H, m, C(1)**H**, 2 × C(13)**H**, C(14)**H**), 5.42 (2H, s, C(10)**H₂**).

δ_C(126 MHz, DMSO-d_6): 178.8 (d, J = 3.0 Hz, **C**(7)), 156.1 (s, **C**(8)), 149.2 (d, J = 253.5 Hz, **C**(3)), 148.5 (s, **C**(9)), 135.0 (s, **C**(11)), 133.3 (d, J = 10.0 Hz, **C**(4)), 128.5 (s, 2 × **C**(13)), 128.4 (s, **C**(14)), 128.1 (s, 2 × **C**(12)), 127.0 (d, J = 6.5 Hz, **C**(1)), 125.5 (d, J = 21.0 Hz, **C**(2)), 123.3 (d, J = 1.5 Hz, **C**(5)), 120.5 (d, J = 3.5 Hz, **C**(6)), 68.8 (s, **C**(10))

δ_F(471 MHz, DMSO-d_6): -116.2 (dd, J = 11.5, 4.0 Hz).

m/z HRMS (ESI): 322.0487; $C_{16}H_{10}FNNaO_4^+$ (M+Na$^+$) requires 322.0486.

Isopropyl 2-(2-(((benzyloxy)carbonyl)amino)-3-fluorophenyl)-2-oxoacetate: 15i

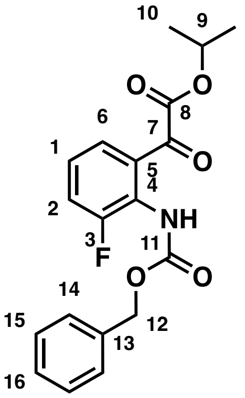

Benzyl 7-fluoro-2,3-dioxoindoline-1-carboxylate (**S5**, 500 mg, 1.7 mmol, 1.0 equiv.) and 2-propanol (18 mL) were subjected to **General Procedure C**, heating for 7 h. Purification by column chromatography (10% ethyl acetate in petroleum ether) gave isopropyl 2-(2-(((benzyloxy)carbonyl)amino)-3-fluorophenyl)-2-oxoacetate (**15i**) as a viscous orange oil (502 mg, 1.4 mmol, 84%).

R_f: 0.5 (40% diethyl ether n petroleum ether).

ν_{max} /cm^{-1} (thin film): 3331 (br), 3035, 2984, 2940, 1728, 1616, 1588, 1506.

δ_H(500 MHz, $CDCl_3$): 7.91 (1H, s, N**H**), 7.52 (1H, dt, J = 8.0, 1.0 Hz, C(6)**H**), 7.42 – 7.30 (6H, m, C(2)**H**, 2 × C(14)**H**, 2 ×C(15)**H**, C(16)**H**), 7.25 (1H, td, J = 8.0, 5.0 Hz, C(1)**H**), 5.23 (1H, hept, J = 6.5 Hz, C(9)**H**), 5.20 (2H, s, C(12)**H₂**), 1.37 (6H, d, J = 6.5 Hz, 2 × C(10)**H₃**).

δ_C(126 MHz, $CDCl_3$): 186.4 (d, J = 3.0 Hz, **C**(7)), 162.1 (s, **C**(8)), 155.6 (d, J = 251.0 Hz, **C**(3)), 153.6 (s, **C**(11)), 135.6 (s, **C**(13)), 128.7 (s, 2 × **C**(15)), 128.6 (s, **C**(16)), 128.5 (s, 2 × **C**(14)), 127.9 (d, J = 1.5 Hz, **C**(5)), 127.1 (d, J = 3.5 Hz, **C**(6)), 126.7 (d, J = 13.0 Hz, **C**(4)), 125.7 (d, J = 8.0 Hz, **C**(1)), 121.5 (d, J = 21.0 Hz, **C**(2)), 71.3 (s, **C**(9)), 68.2 (s, **C**(12)), 21.7 (s, 2 × **C**(10)).

δ_F(471 MHz, $CDCl_3$): 119.1.

m/z HRMS (ESI): 360.1240; $C_{19}H_{19}FNO_5^+$ (M+H$^+$) requires 360.1248.

Isopropyl 2-(2-(((benzyloxy)carbonyl)amino)-5-nitrophenyl)-2-oxoacetate: 15g

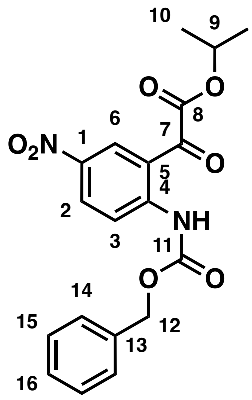

5-Nitroindoline-2,3-dione (442 mg, 2.3 mmol, 1.0 equiv.), tetrahydrofuran (10 mL), triethylamine (0.38 mL, 2.8 mmol, 1.2 equiv.), and benzyl chloroformate (95%) (0.41 mL, 2.8 mmol, 1.2 equiv.) were subjected to **General Procedure B**, stirring for 90 min to give a light brown solid (718 mg) that was used without further purification. The previous product (600 mg, 1.8 mmol, 1.0 equiv.) and 2-propanol (18 mL) were subjected to **General Procedure C**, heating for 15 h. Purification by column chromatography (20% diethyl ether in petroleum ether) gave isopropyl 2-(2-(((benzyloxy)carbonyl)amino)-5-nitrophenyl)-2-oxoacetate (**15g**) as a pale yellow solid (517 mg, 1.3 mmol, 70%).

R_f: 0.4 (20% diethyl ether in petroleum ether).

m.p.: 130.5–132.5 °C.

ν_{max} /cm^{-1} (thin film): 3273 (br), 2986, 2938, 1737, 1665, 1618, 1583, 1542, 1511, 1339.

δ_H(500 MHz, $CDCl_3$): 10.96 (1H, s, N**H**), 8.77 (1H, d, *J* = 9.5 Hz, C(3)**H**), 8.68 (1H, dd, *J* = 2.5, 0.5 Hz, C(6)**H**), 8.45 (1H, ddd, *J* = 9.5, 2.5, 0.5 Hz, C(2)**H**), 7.46 – 7.32 (5H, m, 2 × C(14)**H**, 2 × C15H, C(16)**H**), 5.39 (1H, hept, *J* = 6.5 Hz, C(9)**H**), 5.27 (2H, s, C(12)**H₂**), 1.45 (6H, d, *J* = 6.5 Hz, 2 × C(10)**H₃**).

δ_C(126 MHz, $CDCl_3$): 189.0 (**C**(7)), 162.1 (**C**(8)), 152.9 (**C**(11)), 148.0 (**C**(4)), 141.3 (**C**(1)), 135.2 (**C**(13)), 131.2 (**C**(2)), 129.4 (**C**(6)), 128.8 (2 × **C**(15)), 128.7 (**C**(16)) 128.6 (2 × **C**(14)), 119.7 (**C**(3)), 116.3 (**C**(5)), 72.2 (**C**(9)), 68.2 (**C**(12)), 21.8 (2 ×**C**(10)).

m/z HRMS (ESI): 409.1007; $C_{19}H_{18}N_2NaO_7^+$ (M+Na$^+$) requires 409.1006.

Isopropyl 2-(2-(((benzyloxy)carbonyl)amino)-5-methoxyphenyl)-2-oxoacetate: 15r

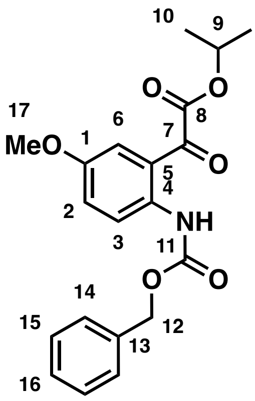

To a well-stirred suspension of sodium hydride (60% in mineral oil) (1.8 g, 45.2 mmol, 2.0 equiv.) in tetrahydrofuran (90 mL) at 0 °C was added portionwise 5-methoxyindoline-2,3-dione (**20**, 4.0 g, 22.6 mmol, 1.0 equiv.) over 5 min and the mixture stirred at room temperature for 35 min. To this was added dropwise benzyl chloroformate (95%) (4.0 mL, 27.1 mmol, 1.2 equiv.) and the mixture stirred for 3.5 h. The reaction was cooled to 0 °C and hydrochloric acid (2 M) (50 mL) carefully added. The tetrahydrofuran was removed *in vacuo* then ethyl acetate added and the layers separated. The aqueous portion was extracted with ethyl acetate (× 4)

and the combined organic portions dried (Na_2SO_4) and concentrated *in vacuo* to give a dark brown solid that was used without further purification. To this was added 2-propanol (250 mL) and concentrated sulfuric acid (30 drops) then the mixture was heated at reflux for 61 h. Purification by column chromatography (20% diethyl ether in petroleum ether) gave isopropyl 2-(2-(((benzyloxy)carbonyl)amino)-5-methoxyphenyl)-2-oxoacetate (**15r**) as a yellow solid (6.5 g, 17.6 mmol, 78% from 5-methoxyindoline-2,3-dione).

R_f: 0.3 (20% diethyl ether in petroleum ether)

m.p.: 57.5–59.5 °C.

ν_{max} /cm^{-1} (thin film): 3307 (br), 3034, 2983, 2939, 2838, 1732, 1655, 1619, 1588, 1523;

δ_H(500 MHz, $CDCl_3$): 10.39 (1H, s, N**H**), 8.46 (1H, d, *J* = 9.5 Hz, C(3)**H**), 7.44 – 7.31 (5H, m, 2 × C(14)**H**, 2 × C(15)**H**, C(16)**H**), 7.22 (1H, ddd, *J* = 9.5, 3.0, 0.5 Hz, C(2)**H**), 7.15 (1H, d, *J* = 3.0 Hz, C(6)**H**), 5.32 (1H, hept, *J* = 6.0 Hz, C(9)**H**), 5.21 (2H, s, C(12)$\mathbf{H_2}$), 3.80 (3H, s, C(17)$\mathbf{H_3}$), 1.41 (6H, d, *J* = 6.5 Hz, 2 × C(10)$\mathbf{H_3}$).

δ_C(126 MHz, $CDCl_3$): 190.0 (**C**(7)), 163.3 (**C**(8)), 154.0 (**C**(1)), 153.6 (**C**(11)), 136.7 (**C**(4)), 136.1 (**C**(13)), 128.7 (2 × **C**(15)), 128.4 (**C**(16)), 128.3 (2 × **C**(14)), 123.7 (**C**(2)), 121.1 (**C**(3)), 117.8 (**C**(5)), 116.7 (**C**(6)), 71.1 (**C**(9)), 67.2 (**C**(12)), 55.8 (**C**(17)), 21.8 (2 × **C**(10)).

m/z HRMS (ESI): 394.1263; $C_{20}H_{21}NNaO_6^+$ (M+Na$^+$) requires 394.1261.

Isopropyl 2-(2-(((benzyloxy)carbonyl)amino)-4,6-dichlorophenyl)-2-oxoacetate: 15j

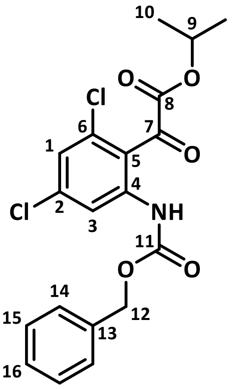

4,6-Dichloroindoline-2,3-dione (497 mg, 2.3 mmol, 1.0 equiv.), tetrahydrofuran (10 mL), triethylamine (0.38 mL, 2.8 mmol, 1.2 equiv.), and benzyl chloroformate (95%) (0.41 mL, 2.8 mmol, 1.2 equiv.) were subjected to **General Procedure B**, stirring for 90 min to give a brown solid (814 mg) that was used without further purification. The previous product (683 mg, 2.0 mmol, 1.0 equiv.) and 2-propanol (20 mL) were subjected to **General Procedure C**, heating for 6 h. Purification by column chromatography (10% diethyl ether in petroleum ether) gave isopropyl 2-(2-(((benzyloxy)carbonyl)amino)-4,6-dichlorophenyl)-2-oxoacetate (**15j**) as an orange solid (426 mg, 1.0 mmol, 45% from 4,6-dichloroindoline-2,3-dione).

R_f: 0.4 (15% diethyl ether in petroleum ether)

m.p.: 66.0–68.0 °C.

ν_{max} /cm^{-1} (thin film): 3340 (br), 3107, 3069, 3034, 2984, 2938, 1732, 1672, 1589, 1562, 1510;

δ_H(500 MHz, $CDCl_3$): 9.36 (1H, s, N**H**), 8.42 (1H, d, *J* = 2.0 Hz, C(3)**H**), 7.43 – 7.33 (5H, m, 2 × C(14)**H**, 2 × C(15)**H**, C(16)**H**), 7.12 (1H, d, *J* = 2.0 Hz, C(1)**H**), 5.21 (1H, hept, *J* = 6.5 Hz, C(9)**H**), 5.20 (2H, s, C(12)$\mathbf{H_2}$), 1.36 (6H, d, *J* = 6.5 Hz, 2 × C(10)$\mathbf{H_3}$).

δ_C(126 MHz, $CDCl_3$): 188.5 (**C**(7)), 162.2 (**C**(8)), 152.9 (**C**(11)), 142.6 (**C**(4)), 141.3 (**C**(2)), 135.9 (**C**(6)), 135.5 (**C**(13)), 128.8 (2 × **C**(15)), 128.7 (**C**(16)), 128.6 (2 × **C**(14)), 124.3 (**C**(1)), 119.0 (**C**(3)), 118.5 (**C**(5)), 71.7 (**C**(9)), 67.9 (**C**(12)), 21.5 (2 × **C**(10)).

m/z HRMS (ESI): 408.0407; $C_{19}H_{16}{}^{35}Cl_2NO_5^-$ (M–H)$^-$ requires 408.0411.

***(E)*-3-(2-Bromophenyl)acrylaldehyde: 16r**

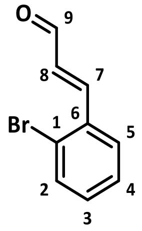

Prepared according to the procedure of Meisner.[8]

To a suspension of (1,3-dioxolan-2-ylmethyl)triphenylphosphonium bromide (**22**, 4.31 g, 10.0 mmol, 2.0 equiv.) in tetrahydrofuran (30 mL) at 0 °C was added lithium bis(trimethylsilyl)amide (1 M in tetrahydrofuran, 12.5 mL, 12.5 mmol, 2.5 equiv.). The resultant yellow solution was warmed to RT and stirred for 15 min. A solution of 2-bromobenzaldehyde (**21**, 919.5 mg, 5.0 mmol, 1.0 equiv.) in tetrahydrofuran (15 mL) was added dropwise to the reaction. The reaction was heated to reflux and stirred for 40 h then cooled to room temperature. Aqueous hydrochloric acid solution (2 M, 45 mL) was added and the reaction was stirred at RT for a further 15 h then extracted with ethyl acetate (x 3). The combined organic layers were dried over sodium sulfate, concentrated *in vacuo* and purified by flash column chromatography (1% to 5% diethyl ether in pentane) to give the title compound as a white solid (832.2 mg, 3.96 mmol, 79%).

R_f: 0.4 (10% diethyl ether in pentane).

m.p.: 66.5 – 68.9 °C.

ν_{max}/cm^{-1} (thin film): 1683 (s), 1619 (m), 1466 (m), 1288 (m), 1134 (s), 1107 (m), 977 (m), 760 (m), 750 (m).

δ_H(600 MHz, $CDCl_3$): 9.78 (1H, d, *J* = 7.5 Hz, C(9)**H**), 7.90 (1H, d, *J* = 16.0 Hz, C(7)**H**), 7.66 (1H, dd, *J* = 8.0, 1.5 Hz, C(2)**H**), 7.66 (1H, dd, *J* = 8.0, 1.5 Hz, C(5)**H**), 7.38 (1H, dddd, *J* = 8.0, 7.5, 1.5, 0.5 Hz, C(4)**H**), 7.29 (1H, ddd, *J* = 8.0, 7.5, 1.5 Hz, C(3)**H**), 6.69 (1H, dd, *J* = 16.0, 7.5 Hz, C(8)**H**).

δ_C(151 MHz, $CDCl_3$): 193.6 (**C**(9)), 150.7 (**C**(7)), 134.0 (**C**(6)), 133.8 (**C**(2)), 132.3 (**C**(3)), 130.9 (**C**(8)), 128.2 (**C**(5)), 128.1 (**C**(4)), 125.9 (**C**(1)).

m/z HRMS ($APCI^+$) found 210.9751; $C_9H_8O^{79}Br^+$ ($M+H^+$) requires 210.9753.

Data in accordance with literature.[9]

Dihydroquinoline synthesis

1-Benzyl 4-isopropyl (*R*)-3-formyl-2-phenylquinoline-1,4(2H)-dicarboxylate: (–)-18a and

1-Benzyl 4-isopropyl (2*S*,3*R*,4*S*)-3-formyl-4-hydroxy-2-phenyl-3,4-dihydroquinoline-1,4(2H)-dicarboxylate: (–)-19a

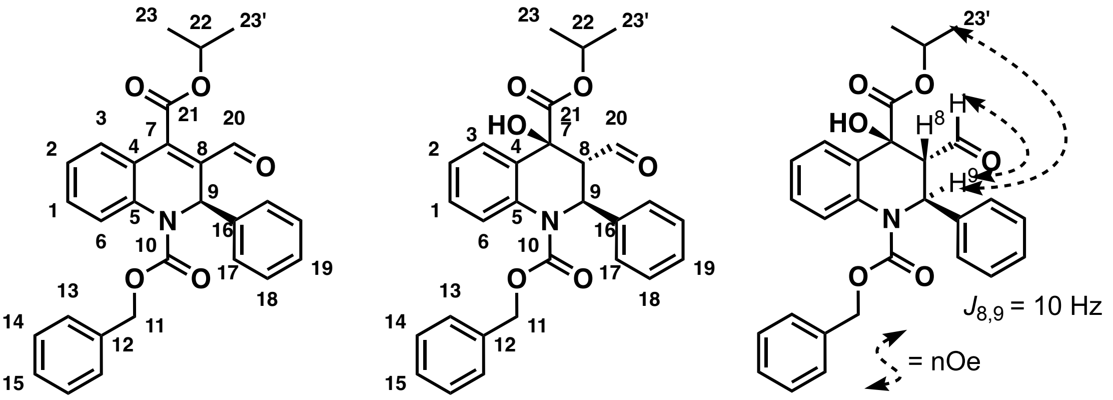

To a solution of (*S*)-2-(diphenyl((triethylsilyl)oxy)methyl)pyrrolidine (**(*S*)-17a**, 11.0 mg, 0.03 mmol, 0.2 equiv.) in 1,2-dichloroethane (0.15 mL) was added (*E*)-cinnamaldehyde (**16a**, 18.9 µL, 0.15 mmol, 1.0 equiv.), isopropyl 2-(2-(((benzyloxy)carbonyl)amino)phenyl)-2-oxoacetate (**15a**, 153.6 mg, 0.45 mmol, 3.0 equiv.), sodium acetate (6.2 mg, 0.08 mmol, 0.5 equiv.) and freshly activated 4 Å molecular sieves (ground) (75 mg). The reaction was stirred at RT for 69 h and then purified by flash column chromatography (5% to 30% ethyl acetate in petroleum ether) to give 1-benzyl 4-isopropyl (*R*)-3-formyl-2-phenylquinoline-1,4(2H)-dicarboxylate (**(–)-18a**) as a yellow solid (5.7 mg, 0.01 mmol, 8%) and 1-benzyl 4-isopropyl (2*S*,3*R*,4*S*)-3-formyl-4-hydroxy-2-phenyl-3,4-dihydroquinoline-1,4(2H)-dicarboxylate (**(–)-19a**) as a viscous yellow oil (7.0 mg, 0.01 mmol, 10%).

Dihydroquinoline: **(–)-18a**

R_f: 0.3 (15% ethyl acetate in petroleum ether).

m.p. 13.5 – 132.5 °C

ν_{max}/cm^{-1} (thin film): 3034, 2983, 1710, 1674, 1619, 1600, 1567

δ_H(500 MHz, $CDCl_3$): 9.89 (1H, s, C(20)**H**), 7.75 – 7.55 (1H, m, Ar**H**), 7.49 – 7.31 (7H, m, 7 x Ar**H**), 7.23 – 7.07 (6H, m, 6 x Ar**H**), 6.82 (1H, s, C(9)**H**), 5.42 (1H, hept, *J* = 6.5 Hz, C(22)**H**), 5.33 (1H, d, *J* = 12.0 Hz, C(11)**H**H'), 5.31 (1H, d, *J* = 12.0 Hz, C(11)H**H'**), 1.43 (3H, d, *J* = 6.5 Hz, C(23)**H₃**), 1.41 (3H, d, *J* = 6.5 Hz, C(23')**H₃**)

δ_C(126 MHz, $CDCl_3$): 187.9 (**C**(20)), 165.2 (**C**(21)), 153.7 (**C**(10)), 143.5 (**C**(7)), 137.6 (**Ar**), 137.0 (**Ar**), 135.7 (**Ar**), 133.9 (**C**(8)), 132.0 (**Ar**), 128.8 (**Ar**), 128.7 (**Ar**), 128.6 (**Ar**), 128.4 (**Ar**), 128.3 (**Ar**), 127.1 (**Ar**), 126.5 (**Ar**), 125.4 (**Ar**), 124.8 (**Ar**), 123.6 (**Ar**), 71.2 (**C**(22)), 68.6 (**C**(11)), 52.1 (**C**(9)), 22.1 (**C**(23)), 22.0 (**C**(23'))

m/z HRMS (ESI$^+$) found 478.1622; $C_{28}H_{25}NNaO_5^+$ (M+Na$^+$) requires 478.1625.

ee: 99% (Chiralcel OD, 10% isopropanol in hexane, flow rate 0.8 mL/min; λ = 254 nm): t_R (major) = 14.8 min, t_R (minor) = 16.3 min.

$[\alpha]_D^{25}$ = –456.2 (c = 1.0, $CHCl_3$)

Aldol: (–)-19a

R_f: 0.1 (15% ethyl acetate in petroleum ether).

ν_{max}/cm^{-1} (thin film): 3468 (br), 3033, 2984, 1708, 1603, 1586

δ_H(500 MHz, $CDCl_3$): 9.82 (1H, s, C(20)**H**), 7.68 (1H, dd, *J* = 8.0, 1.5 Hz, Ar**H**), 7.57 (1H, d, *J* = 8.0 Hz, Ar**H**), 7.38 (1H, td, *J* = 8.0, 1.5 Hz, Ar**H**), 7.33 – 7.14 (11H, m, 11 x Ar**H**), 5.76 (1H, d, *J* = 10.0 Hz, C(9)**H**), 5.15 (1H, d, *J* = 12.5 Hz, C(11)**H**H'), 5.12 (1H, d, *J* = 12.5 Hz, C(11)H**H'**), 5.00 (1H, hept, *J* = 6.5 Hz, C(22)**H**), 4.55 (1H, s, C(7)O**H**), 3.36 (1H, d, *J* = 10.0 Hz, C(8)**H**), 1.20 (3H, d, *J* = 6.5 Hz, C(23)**H₃**), 1.02 (3H, d, *J* = 6.5 Hz, C(23')**H₃**)

δ_C(126 MHz, $CDCl_3$): 199.7 (**C**(20)), 172.2 (**C**(21)), 154.3 (**C**(10)), 142.8 (**Ar**), 136.6 (**Ar**), 135.8 (**Ar**), 131.8 (**Ar**), 128.9 (**Ar**), 128.9 (**Ar**), 128.6 (**Ar**), 128.3 (**Ar**), 128.1 (**Ar**), 127.9 (**Ar**), 127.0 (**Ar**), 126.0 (**Ar**), 125.2 (**Ar**), 124.3 (**Ar**), 74.4 (**C**(7)), 71.9(**C**(22)), 68.0 (**C**(11)), 65.1 (**C**(8)), 55.9 (**C**(9)), 21.5 (**C**(23)), 21.2 (**C**(23'))

m/z HRMS (N_2 APCI) found 474.1911; $C_{28}H_{28}NO_6^+$ ($M+H^+$) requires 474.1911.

$[\alpha]_D^{25}$ = –41.0 (c = 1.0, $CHCl_3$)

The relative configuration of **(*rac*)-19a** was assigned as follows: $^3J_{8,9}$ = 10.0 Hz indicating the H-8/H-9 torsion angel is *ca.* 180° and that H-8 and H-9 are in a *trans*-diaxial relationship. Additionally, $^1H/^1H$ NOESY analysis (p S116) indicated that H-9 is on the same face of **(*rac*)-19a** as the *iso*-propyl ester (see diagram above).

Using optimised conditions to prepare a sample of the enantiomer 1-benzyl 4-isopropyl (*S*)-3-formyl-2-phenylquinoline-1,4(2H)-dicarboxylate (**(+)-18a**):

To (*R*)-2-(diphenyl((triethylsilyl)oxy)methyl)pyrrolidine (**(*R*)-17a**, 221 mg, 0.6 mmol, 0.2 equiv.) in CH_2Cl_2 (6.0 mL) were added sequentially (*E*)-cinnamaldehyde (**16a**, 0.38 mL, 3.0 mmol, 1.0 equiv.), benzoic acid (183 mg, 1.5 mmol, 0.5 equiv.), freshly activated 4 Å molecular sieves (beads) (1.0 g), and then isopropyl 2-(2-(((benzyloxy)carbonyl)amino)phenyl)-2-oxoacetate (**15a**, 2.05 g, 6.0 mmol, 2.0 equiv.), then the mixture stirred at room temperature for 71 h. The reaction was filtered, and the filter cake washed with ethyl acetate. The filtrate was washed sequentially with sat. aq. sodium bicarbonate solution, hydrochloric acid (2 M), and brine. The organic portion was dried (Na_2SO_4), concentrated *in vacuo* and purified by flash column chromatography (5% to 15% ethyl acetate in petroleum ether) to give the title compound as a yellow solid.

ee: 99% (Chiralcel OD, 10% isopropanol in hexane, flow rate 0.8 mL/min; λ = 254 nm): t_R (minor) = 13.2 min, t_R (minor) = 14.8 min.

$[\alpha]_D^{25}$ = +487.9 (c = 1.0, $CHCl_3$)

All other characterisation identical

1-Benzyl 4-methyl (*R*)-3-formyl-2-phenylquinoline-1,4(2H)-dicarboxylate: (–)-18b

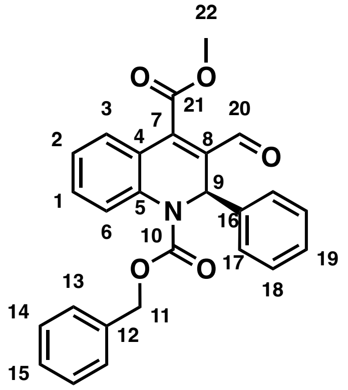

(*E*)-Cinnamaldehyde (**16a**, 18.9 µL, 0.15 mmol, 1.0 equiv.) and methyl 2-(2-(((benzyloxy)carbonyl)amino)phenyl)-2-oxoacetate (**15b**, 94.0 mg, 0.30 mmol, 2.0 equiv.) were subjected to **General Procedure A**, stirring at room temperature for 24 h. Purification by column chromatography (EtOAc:petroleum ether 40-60, 5:95 to 15:85) gave the title compound as a viscous yellow oil (54.0 mg, 0.13 mmol, 84%).

R_f: 0.4 (15% ethyl acetate in petroleum ether).

ν_{max}/cm^{-1} (thin film): 3064, 3034, 2952, 1778, 1708, 1674, 1619, 1600, 1567;

δ_H(500 MHz, $CDCl_3$): 9.85 (1H, s, C(20)**H**), 7.67 (1H, br s, C(6)**H**), 7.43 – 7.32 (7H, m, C(1)**H**, C(3)**H**, 2 × C(13)**H**, 2 × C(14)**H**, C(15)**H**), 7.19 – 7.11 (1H, m, C(2)**H**, 2 × C(17)**H**, 2 × C(18)**H**, C(19)**H**), 6.83 (1H, s, C(9)**H**), 5.33 (1H, d, *J* = 12.5 Hz, C(11)$\boldsymbol{H_A}H_B$), 5.32 (1H, d, *J* = 12.5 Hz, C(11)$H_A\boldsymbol{H_B}$), 4.02 (3H, s, C(22)$\mathbf{H_3}$).

δ_C(126 MHz, $CDCl_3$): 187.9 (**C**(20)), 166.1 (**C**(21)), 153.7 (**C**(10)), 142.7 (**C**(7)), 137.5 (**C**(16)), 136.9 (**C**(5)), 135.6 (**C**(12)), 134.6 (**C**(8)), 132.2 (**C**(1)), 128.8 (2 × **C**(14)), 128.7 (2 × **C**(18)), 128.6 (**C**(15)), 128.4 (**C**(19)), 128.3 (2 × **C**(13)), 127.0 (2 × **C**(17)), 126.7 (**C**(3)), 125.4 (**C**(6)), 124.9 (**C**(2)), 123.4 (**C**(4)), 68.7 (**C**(11)), 53.2 (**C**(22)), 52.1 (**C**(9)).

m/z HRMS (ESI) found 450.1310; for $C_{26}H_{21}NNaO_5^+$ ($M+Na^+$) requires 450.1312.

ee: 98% (Chiralcel AD-H; hexane:2-propanol, 60:40; flow rate 0.8 mL.min^{-1}; λ = 254 nm): t_R (minor) = 13.30 min,t_R (major) = 17.38 min.

$[\alpha]_D^{25} = -487.7$ (c = 1.0, $CHCl_3$).

A sample of the enantiomer, 1-benzyl 4-methyl (*S*)-3-formyl-2-phenylquinoline-1,4(2H)-dicarboxylate (**(+)-18b**) (56.3 mg, 0.13 mmol, 88%) was prepared as above using (*R*)-2-(diphenyl((triethylsilyl)oxy)methyl)pyrrolidine (**(*R*)-17a**) in place of (*S*)-2-(diphenyl((triethylsilyl)oxy)methyl)pyrrolidine (**(*S*)-17a**), stirring for 28 h.

$[\alpha]_D^{25} = +455.8$ (*c* = 1.0, $CHCl_3$).

ee: 98% (Chiralcel AD-H; hexane:2-propanol, 60:40; flow rate 0.8 mL.min^{-1}; λ = 254 nm): t_R (major) = 13.31 min, t_R (minor) = 17.50 min; all other characterisation identical.

4-Isopropyl 1-methyl (*R*)-3-formyl-2-phenylquinoline-1,4(2H)-dicarboxylate: (–)-18c

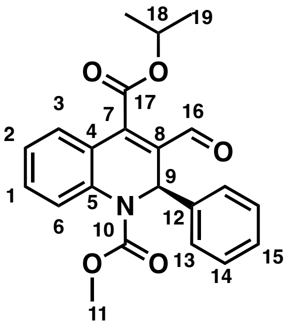

(*E*)-Cinnamaldehyde (**16a**, 18.9 µL, 0.15 mmol, 1.0 equiv.) and isopropyl 2-(2-((methoxycarbonyl)amino)phenyl)-2-oxoacetate (**15c**, 79.6 mg, 0.30 mmol, 2.0 equiv.) were subjected to **General Procedure A**, stirring at room temperature for 49 h. Purification by column chromatography (5% to 15% ethyl acetate in petroleum ether) gave the title compound as a yellow solid (46.4 mg, 0.12 mmol, 82%).

R_f: 0.2 (15% ethyl acetate in petroleum ether).

m.p.: 111.0–113.0 °C.

ν_{max}/cm^{-1} (thin film): 3065, 3034, 2983, 2957,2850, 1717, 1676, 1620, 1601, 1567.

δ_H(500 MHz, $CDCl_3$): 9.89 (1H, s, C(16)**H**), 7.66 (1H, br s, C(6)**H**), 7.39 (1H, ddd, *J* = 8.3, 7.3, 1.5 Hz, C(1)**H**), 7.37 (1H, ddd, *J* = 7.9, 1.5, 0.5 Hz, C(3)**H**), 7.22 – 7.12 (6H, m, C(2)**H**, 2 × C(13)**H**, 2 × C(14)**H**, C(15)**H**), 6.77 (1H, s, C(9)**H**), 5.42 (1H, hept, *J* = 6.3 Hz, C(18)**H**), 3.88 (3H, s, C(11)**H$_3$**), 1.43 (3H, d, *J* = 6.3 Hz, C(19)**H$_3$**), 1.41 (3H, d, *J* = 6.3 Hz, C(19')**H$_3$**).

δ_C(126 MHz, $CDCl_3$): 188.0 (**C**(16)), 165.2 (**C**(17)), 154.4 (**C**(10)), 143.5 (**C**(7)), 137.6 (**C**(12)), 137.0 (**C**(5)), 134.0 (**C**(8)), 132.1 (**C**(1)), 128.7 (2 × **C**(14)), 128.3 (**C**(15)), 127.0 (2 × **C**(13)), 126.5 (**C**(3)), 125.4 (**C**(6)), 124.8 (**C**(2)), 123.6 (**C**(4)), 71.2 (**C**(18)), 53.8 (**C**(11)), 52.0 (**C**(9)), 22.1 (**C**(19)), 22.0 (**C**(19')).

m/z HRMS (ESI) found 402.1311; for $C_{22}H_{21}NNaO_5^+$ ($M+Na^+$) requires 402.1312.

ee: 99% (Chiralcel OD; hexane: 2-propanol, 98:2; flow rate 0.8 mL.min^{-1}; λ = 254 nm): t_R (major) = 23.49 min, t_R (minor) = 26.31 min.

$[\alpha]_D^{25} = -518.7$ (*c* = 1.0, $CHCl_3$).

A sample of racemic 4-isopropyl 1-methyl 3-formyl-2-phenylquinoline-1,4(2H)-dicarboxylate (*rac*-**18c**) was prepared as above using pyrrolidine (12.3 µL, 0.15 mmol, 1.0 equiv.) in place of (*S*)-2-(diphenyl((triethylsilyl)oxy)methyl)pyrrolidine (**(*S*)-17a**).

1-(*tert*-Butyl) 4-isopropyl (*R*)-3-formyl-2-phenylquinoline-1,4(2H)-dicarboxylate: (–)-18d

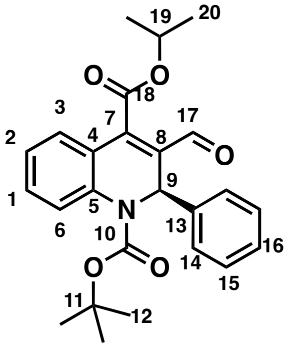

(*E*)-Cinnamaldehyde (**16a**, 18.9 µL, 0.15 mmol, 1.0 equiv.) and isopropyl 2-(2-((*tert*-butoxycarbonyl)amino)phenyl)-2-oxoacetate (**15d**, 92.2 mg, 0.30 mmol, 2.0 equiv.) were subjected to **General Procedure A**, stirring at room temperature for 121 h. Purification by column chromatography (5% to 15% ethyl acetate in petroleum ether) gave the title compound as a viscous yellow oil (43.9 mg, 0.10 mmol, 69%).

R_f: 0.5 (15% ethyl acetate in petroleum ether).

ν_{max}/cm^{-1} (thin film): 3064, 3033, 2980, 2935, 2877, 1703, 1674, 1618, 1600, 1566.

δ_H(500 MHz, $CDCl_3$): 9.89 (1H, s, C(17)**H**), 7.67 (1H, br d, *J* = 8.0 Hz, C(6)**H**), 7.36 (1H, dd, *J* = 8.0, 1.5 Hz, C(3)**H**), 7.36 (1H, ddd, *J* = 8.0, 7.0, 1.5 Hz, C(1)**H**), 7.22 – 7.14 (5H, m, 2 × C(14)**H**, 2 × C(15)**H**, C(16)**H**), 7.11 (1H, ddd, *J* = 8.5, 7.0, 1.0 Hz, C(2)**H**), 6.72 (1H, s, C(9)**H**), 5.43 (1H, hept, *J* = 6.5 Hz, C(19)**H**), 1.57 (9H, s, 3 × C(12)$\mathbf{H_3}$), 1.44 (3H, d, *J* = 6.5 Hz, C(20)$\mathbf{H_3}$), 1.42 (3H, d, *J* = 6.5 Hz, C(20')$\mathbf{H_3}$).

δ_C(126 MHz, $CDCl_3$): 188.0 (**C**(17)), 165.5 (**C**(18)), 152.6 (**C**(10)), 143.5 (**C**(7)), 138.2 (**C**(13)), 137.6 (**C**(5)), 133.9 (**C**(8)), 131.8 (**C**(1)), 128.6 (2 × **C**(15)), 128.2 (**C**(16)), 127.0 (2 × **C**(14)), 126.4 (**C**(3)), 125.5 (**C**(6)), 124.3 (**C**(2)), 123.5 (**C**(4)), 82.8 (**C**(11)), 71.1 (**C**(19)), 51.7 (**C**(9)), 28.5 (3 × **C**(12)), 22.1 (**C**(20)), 22.0 (**C**(20')).

m/z HRMS (ESI) found 444.1778; for $C_{25}H_{27}NNaO_5^+$ (M+Na^+) requires 444.1781.

ee: 99% (Chiralcel OD; hexane:2-propanol, 99:1; flow rate 0.8 mL.min^{-1}; λ = 254 nm): t_R (major) = 14.73 min, t_R (minor) = 17.13 min.

$[\alpha]_D^{25} = -449.9$ (*c* = 1.0, $CHCl_3$).

A sample of racemic 1-(*tert*-butyl) 4-isopropyl 3-formyl-2-phenylquinoline-1,4(2H)-dicarboxylate (*rac*-**18d**) was prepared as above using pyrrolidine (12.3 µL, 0.15 mmol, 1.0 equiv.) in place of (*S*)-2-(diphenyl((triethylsilyl)oxy)methyl)pyrrolidine (**(*S*)-17a**).

1-Benzyl 4-isopropyl (*R*)-6-chloro-3-formyl-2-phenylquinoline-1,4(2H)-dicarboxylate : (–)-18e

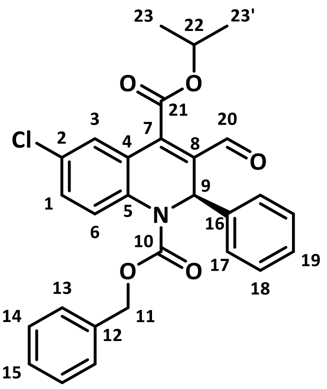

(*E*)-Cinnamaldehyde (**16a**, 18.9 µL, 0.15 mmol, 1.0 equiv.) and isopropyl 2-(2-(((benzyloxy) carbonyl)amino)-5-chlorophenyl)-2-oxoacetate (**15e**, 112.7 mg, 0.30 mmol, 2.0 equiv.) were subjected to **General Procedure A**, stirring at room temperature for 47 h. Purification by column chromatography (5% to 10% ethyl acetate in petroleum ether) gave the title compound as a viscous yellow oil (67.3 mg, 0.14 mmol, 92%).

R_f: 0.4 (10% ethyl acetate in petroleum ether).

ν_{max}/cm^{-1} (thin film): 3065, 3034, 2982, 1780, 1713, 1678, 1620, 1558.

δ_H(500 MHz, $CDCl_3$): 9.91 (1H, s, C(20)**H**), 7.42 – 7.28 (8H, m, 8 x Ar**H**), 7.25 – 7.16 (3H, m, 3 x Ar**H**), 7.15 – 7.08 (2H, m, 2 x Ar**H**), 6.81 (1H, s, C(9)**H**), 5.43 (1H, hept, *J* = 6.5 Hz, C(22)**H**), 5.31 (2H, s, C(11) **HH'**), 1.44 (3H, d, *J* = 6.5 Hz, C(23)$\mathbf{H_3}$), 1.42 (3H, d, *J* = 6.5 Hz, C(23')$\mathbf{H_3}$)

δ_C(126 MHz, $CDCl_3$): 187.8 (**C**(20)), 164.6 (**C**(21)), 153.5 (**C**(10)), 141.9 (**C**(7)), 137.1 (**Ar**), 135.4 (**Ar**), 135.4 (**Ar**), 135.1 (**C**(8)), 131.7 (**Ar**), 130.2 (**Ar**), 128.8 (**Ar**), 128.8 (**Ar**), 128.7 (**Ar**), 128.6 (**Ar**), 128.4 (**Ar**), 127.0 (**Ar**), 126.6 (**Ar**), 126.2 (**Ar**), 125.0 (**Ar**), 71.6 (**C**(22)), 68.9 (**C**(11)), 52.2 (**C**(9)), 22.1 (**C**(23)), 22.0 (**C**(23'))

m/z HRMS (ESI^+) found 512.1236; $C_{28}H_{24}{}^{35}ClNNaO_5^+$ ($M+Na^+$) requires 512.1235.

ee: 98% (Chiralcel OD, 1.6% isopropanol in hexane, flow rate 0.8 mL/min; λ = 254 nm): t_R (major) = 30.3 min, t_R (minor) = 34.6 min.

$[\alpha]_D^{25}$ = –303.6 (c = 1.0, $CHCl_3$)

1-Benzyl 4-isopropyl (*R*)-3-formyl-6-methyl-2-phenylquinoline-1,4(2H)-dicarboxylate: (–)-18f

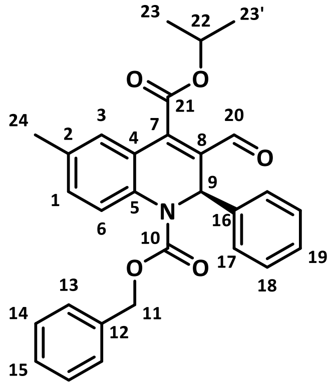

(*E*)-Cinnamaldehyde (**16a**, 18.9 μL, 0.15 mmol, 1.0 equiv.) and isopropyl 2-(2-(((benzyloxy) carbonyl)amino)-5-methylphenyl)-2-oxoacetate (**15f**, 106.6 mg, 0.30 mmol, 2.0 equiv.) were subjected to **General Procedure A**, stirring at room temperature for 48 h. Purification by column chromatography (5% to 15% ethyl acetate in petroleum ether) gave the title compound as a viscous yellow oil (66.2 mg, 0.14 mmol, 94%).

R_f: 0.3 (15% ethyl acetate in petroleum ether).

ν_{max}/cm^{-1} (thin film): 3064, 3033, 2981, 2934, 2858, 1774, 1708, 1674, 1617, 1567.

δ_H(500 MHz, $CDCl_3$): 9.89 (1H, s, C(20)**H**), 7.65 – 7.50 (1H, m, 1 x Ar**H**), 7.43 – 7.32 (5H, m, 5 x Ar**H**), 7.25 – 7.09 (7H, m, 7 x Ar**H**), 6.80 (1H, s, C(9)**H**), 5.44 (1H, hept, *J* = 6.0 Hz, C(22)**H**), 5.35 – 5.28 (2H, m, C(11) **HH'**), 2.31 (3H, s, C(24)$\mathbf{H_3}$), 1.44 (3H, d, *J* = 6.0 Hz, C(23)$\mathbf{H_3}$), 1.42 (3H, d, *J* = 6.0 Hz, C(23')$\mathbf{H_3}$)

δ_C(126 MHz, $CDCl_3$): 187.9 (**C**(20)), 165.3 (**C**(21)), 153.8 (**C**(10)), 143.6 (**C**(7)), 137.6 (**Ar**), 135.7 (**Ar**), 134.5 (**Ar**), 134.5 (**Ar**), 133.8 (**C**(8)), 133.0 (**Ar**), 128.8 (**Ar**), 128.6 (**Ar**), 128.5 (**Ar**), 128.3 (**Ar**), 128.2 (**Ar**), 127.1 (**Ar**), 126.7 (**Ar**), 125.2 (**Ar**), 123.4 (**Ar**), 71.1 (**C**(22)), 68.5 (**C**(11)), 52.0 (**C**(9)), 22.1 (**C**(23)), 21.9 (**C**(23')), 21.0 (**C**(24))

m/z HRMS (ESI^+) found 492.1779; $C_{29}H_{27}NNaO_5^+$ ($M+Na^+$) requires 492.1781.

ee: 98% (Chiralcel AD-H, 10% isopropanol in hexane, flow rate 0.8 mL/min; λ = 254 nm): t_R (minor) = 22.0 min, t_R (major) = 30.0 min

$[\alpha]_D^{25}$ = –422.2 (c = 1.0, $CHCl_3$)

1-Benzyl 4-isopropyl (*R*)-8-fluoro-3-formyl-2-phenylquinoline-1,4(2H)-dicarboxylate: (–)-18i

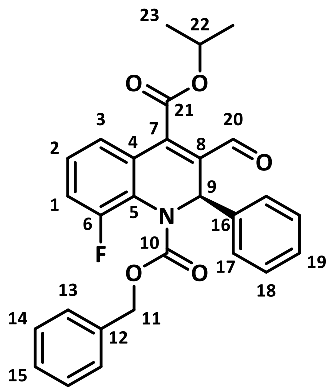

(*E*)-Cinnamaldehyde (**16a**, 18.9 µL, 0.15 mmol, 1.0 equiv.) and isopropyl 2-(2-(((benzyloxy)carbonyl)amino)-3-fluorophenyl)-2-oxoacetate (**15i**, 107.8 mg, 0.30 mmol, 2.0 equiv.) were subjected to **General Procedure A**, stirring at room temperature for 45 h. Purification by column chromatography (10% to 20% diethyl ether in petroleum ether) gave the title compound as a viscous yellow oil (55.5 mg, 0.12 mmol, 78%).

R_f: 0.3 (20% diethyl ether in petroleum ether).

ν_{max}/cm^{-1} (thin film): 3089, 3066, 3033, 2985, 2923, 2851, 1716, 1677, 1612, 1574

δ_H(500 MHz, $CDCl_3$): 10.00 (1H, s, C(20)**H**), 7.46 – 7.30 (5H, m, 5 x Ar**H**), 7.25 – 7.07 (8H, m, 8 x Ar**H**), 6.81 (1H, s, C(9)**H**), 5.43 (1H, hept, *J* = 6.5 Hz, C(22)**H**), 5.36 – 5.27 (2H, m, C(11) **HH'**), 1.44 (3H, d, *J* = 6.5 Hz, C(23)**H₃**), 1.42 (3H, d, *J* = 6.5 Hz, C(23')**H₃**).

δ_C(126 MHz, $CDCl_3$, 22 resonances out of 24 observed, 2 x Ar resonances not observed): 188.1 (**C**(20)), 164.8 (**C**(21)), 156.2 (d, *J* = 253.5 Hz, **Ar**), 153.7 (**C**(10)), 142.5 (**C**(7)), 136.5 (**C**(8)), 135.5 (**Ar**), 128.7 (**Ar**), 128.7 (**Ar**), 128.5 (**Ar**), 128.3 (**Ar**), 128.3 (**Ar**), 126.7 (**Ar**), 126.7 (**Ar**), 124.6 (d, *J* = 12.5 Hz, **Ar**), 122.1 (d, *J* = 3.5 Hz, **Ar**), 119.4 (**Ar**), 71.4 (**C**(22)), 69.1 (**C**(1)), 52.3 (**C**(9)), 22.1 (**C**(23)), 21.9 (**C**(23')).

δ_F(471 MHz, $CDCl_3$): –116.0

m/z HRMS (ESI$^+$) found 496.1530; $C_{28}H_{24}FNNaO_5^+$ (M+Na$^+$) requires 496.1531.

ee: 97% (Chiralcel AD-H, 30% isopropanol in hexane, flow rate 0.8 mL/min; λ = 254 nm): t_R (minor) = 14.4 min, t_R (major) = 16.7 min

$[\alpha]_D^{25}$ = –376.6 (c = 1.0, $CHCl_3$)

1-Benzyl 4-isopropyl (*R*)-3-formyl-6-nitro-2-phenylquinoline-1,4(2H)-dicarboxylate: (–)-18g

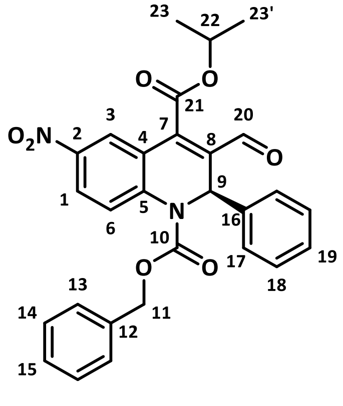

(*E*)-Cinnamaldehyde (**16a**, 18.9 µL, 0.15 mmol, 1.0 equiv.) and isopropyl 2-(2-(((benzyloxy)carbonyl)amino)-5-nitrophenyl)-2-oxoacetate (**15g**, 115.9 mg, 0.30 mmol, 2.0 equiv.) were subjected to **General Procedure A**, stirring at room temperature for 47 h. Purification by column chromatography (5% to 15% ethyl acetate in petroleum ether) gave the title compound as a viscous yellow oil (57.6 mg, 0.12 mmol, 77%).

R_f: 0.3 (40% ethyl acetate in petroleum ether).

ν_{max}/cm^{-1} (thin film): 3066, 3034, 2983, 2936, 2875, 1720, 1680, 1608, 1576, 1522, 1342.

δ_H(500 MHz, $CDCl_3$): 9.97 (1H, s, C(20)**H**), 8.37 (1H, d, *J* = 2.5 Hz, Ar**H**), 8.19 (1H, dd, *J* = 9.0, 2.5 Hz, Ar**H**), 7.88 (1H, d, *J* = 9.0 Hz, Ar**H**), 7.47 – 7.35 (5H, m, 5 x Ar**H**), 7.25 – 7.15 (3H, m, 3 x Ar**H**), 7.14 – 7.07 (2H, m, 2 x Ar**H**), 6.85 (1H, s, C(9)**H**), 5.48 (1H, hept, *J* = 6.5 Hz, C(22)**H**), 5.37 – 5.34 (2H, m, C(11) **HH'**), 1.47 (3H, d, *J* = 6.5 Hz, C(23)$\mathbf{H_3}$), 1.46 (3H, d, *J* = 6.5 Hz, C(23')$\mathbf{H_3}$)

δ_C(126 MHz, $CDCl_3$): 187.6 (**C**(20)), 164.0 (**C**(21)), 153.2 (**C**(10)), 143.8 (**Ar**), 142.4 (**Ar**), 140.8 (**C**(7)), 136.8 (**Ar**), 136.0 (**C**(8)), 134.9 (**Ar**), 129.0 (**Ar**), 129.0 (**Ar**), 128.9 (**Ar**), 128.9 (**Ar**), 128.6 (**Ar**), 127.0 (**Ar**), 126.4 (**Ar**), 125.5 (**Ar**), 123.9 (**Ar**), 122.4 (**Ar**), 72.0 (**C**(22)), 69.4 (**C**(11)), 52.7 (**C**(9)), 22.1 (**C**(23)), 22.0 (**C**(23'))

m/z HRMS (ESI^+) found 523.1478; $C_{28}H_{24}N_2NaO_7^+$ ($M+Na^+$) requires 523.1476.

ee: 97% (Chiralcel AD-H, 30% isopropanol in hexane, flow rate 0.8 mL/min; λ = 254 nm): t_R (minor) = 13.9 min, t_R (major) = 18.1 min

$[\alpha]_D^{25}$ = –409.0 (c = 1.0, $CHCl_3$)

1-Benzyl 4-isopropyl (*R*)-3-formyl-6-methoxy-2-phenylquinoline-1,4(2H)-dicarboxylate: (–)-18h

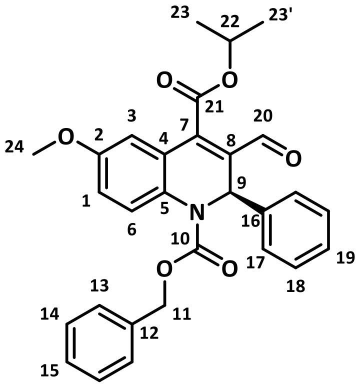

(*E*)-Cinnamaldehyde (**16a**, 18.9 µL, 0.15 mmol, 1.0 equiv.) and isopropyl 2-(2-(((benzyloxy)carbonyl)amino)-5-methoxyphenyl)-2-oxoacetate (**15r**, 111.4 mg, 0.30 mmol, 2.0 equiv.) were subjected to **General Procedure A**, stirring at room temperature for 25 h. Purification by column chromatography (5% to 20% ethyl acetate in petroleum ether) gave the title compound as a viscous yellow oil (60.2 mg, 0.12 mmol, 83%).

R_f: 0.2 (15% ethyl acetate in petroleum ether).

ν_{max}/cm^{-1} (thin film): 3064, 3032, 2981, 2938, 2838, 1707, 1674, 1609, 1568.

δ_H(500 MHz, $CDCl_3$): 9.91 (1H, s, C(20)**H**), 7.79 – 7.52 (1H, m, Ar**H**), 7.47 – 7.30 (5H, m, 5 x Ar**H**), 7.24 – 7.09 (5H, m, 5 x Ar**H**), 7.00 – 6.89 (2H, m, 2 x Ar**H**), 6.82 (1H, s, C(9)**H**), 5.43 (1H, hept, *J* = 6.5 Hz, C(22)**H**), 5.35 – 5.24 (2H, m, C(11) **HH'**), 3.78 (3H, s, C(24)$\mathbf{H_3}$), 1.44 (3H, d, *J* = 6.5 Hz, C(23)$\mathbf{H_3}$), 1.42 (3H, d, *J* = 6.3 Hz, C(23')$\mathbf{H_3}$).

δ_C(126 MHz, $CDCl_3$): 187.9 (**C**(20)), 165.1 (**C**(21)), 156.3 (**Ar**), 153.8 (**C**(10)), 143.3 (**C**(7)), 137.4 (**Ar**), 135.8 (**Ar**), 134.5 (**C**(8)), 130.0 (**Ar**), 128.7 (**Ar**), 128.6 (**Ar**), 128.5 (**Ar**), 128.3 (**Ar**), 128.3 (**Ar**), 127.1 (**Ar**), 126.6 (**Ar**), 124.4 (**Ar**), 118.1 (**Ar**), 110.6 (**Ar**), 71.1 (**C**(22)), 68.5 (**C**(11)), 55.6 (**C**(24)), 52.0 (**C**(9)), 22.1 (**C**(23)), 21.9 (**C**(23'))

m/z HRMS (ESI^+) found 508.1732; $C_{29}H_{27}NNaO_6^+$ ($M+Na^+$) requires 508.1731.

ee: 98% (Chiralcel AD-H, 40% isopropanol in hexane, flow rate 0.8 mL/min; λ = 254 nm): t_R (minor) = 12.9 min, t_R (major) = 15.0 min

$[\alpha]_D^{25}$ = –453.8 (c = 1.0, $CHCl_3$)

1-Benzyl 4-isopropyl (*R*)-5,7-dichloro-3-formyl-2-phenylquinoline-1,4(2H)-dicarboxylate: (–)-18j

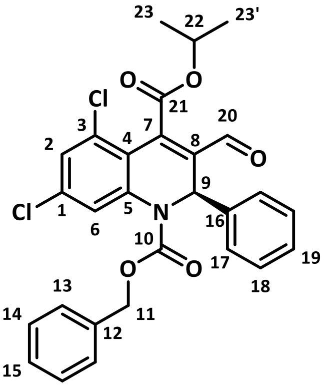

(*E*)-Cinnamaldehyde (**16a**, 18.9 µL, 0.15 mmol, 1.0 equiv.) and isopropyl 2-(2-(((benzyloxy)carbonyl)amino)-5-methoxyphenyl)-2-oxoacetate (**15j**, 123.1 mg, 0.30 mmol, 2.0 equiv.) were subjected to **General Procedure A**, stirring at room temperature for 49 h. Purification by column chromatography (5% to 10% ethyl acetate in petroleum ether) gave the title compound as a viscous yellow oil (77.1 mg, 0.15 mmol, 98%).

R_f: 0.3 (10% ethyl acetate in petroleum ether).

ν_{max}/cm^{-1} (thin film): 3089, 3066, 3033, 2981, 2936, 2880, 1718, 1674, 1603, 1585, 1543.

δ_H(500 MHz, $CDCl_3$): 10.13 (1H, s, C(20)**H**), 7.70 (1H, s, Ar**H**), 7.45 – 7.34 (5H, m, 5 x Ar**H**), 7.22 – 7.18 (3H, m, 3 x Ar**H**), 7.17 (1H, d, *J* = 2.0 Hz, Ar**H**), 7.09 – 7.04 (2H, m, 2 x Ar**H**), 6.88 (1H, s, C(9)**H**), 5.37 (1H, d, *J* = 12.0 Hz, C(11)**H**H'), 5.33 (1H, d, *J* = 12.0 Hz, C(11)H**H'**), 5.32 (1H, hept, *J* = 6.5 Hz, C(22)**H**), 1.39 (3H, d, *J* = 6.5 Hz, C(23)$\mathbf{H_3}$), 1.38 (3H, d, *J* = 6.5 Hz, C(23')$\mathbf{H_3}$)

δ_C(126 MHz, $CDCl_3$, 23 resonances out of 24 observed, 1 x Ar resonance not observed): 188.4 (**C**(20)), 164.4 (**C**(21)), 153.1 (**C**(10)), 140.6 (**C**(7)), 139.8 (**Ar**), 137.4 (**Ar**), 137.3 (**C**(8)), 136.1 (**Ar**), 135.2 (**Ar**), 133.4 (**Ar**), 128.9 (**Ar**), 128.8 (**Ar**), 128.5 (**Ar**), 128.4 (**Ar**), 126.8 (**Ar**), 126.8 (**Ar**), 124.2 (**Ar**), 122.3 (**Ar**), 71.6 (**C**(22)), 69.1 (**C**(11)), 51.4 (**C**(9)), 21.8 (**C**(23)), 21.8 (**C**(23'))

m/z HRMS (ESI^+) found 524.1027; $C_{28}H_{24}{}^{35}Cl_2NO_5^+$ ($M+H^+$) requires 524.1026.

ee: 78% (Chiralcel AD-H, 5% isopropanol in hexane, flow rate 0.8 mL/min; λ = 254 nm): t_R (minor) = 16.8 min, t_R (major) = 19.4 min

$[\alpha]_D^{25}$ = –199.8 (c = 1.0, $CHCl_3$)

1-Benzyl 4-isopropyl (*R*)-2-(4-chlorophenyl)-3-formylquinoline-1,4(2H)-dicarboxylate: (–)-18k

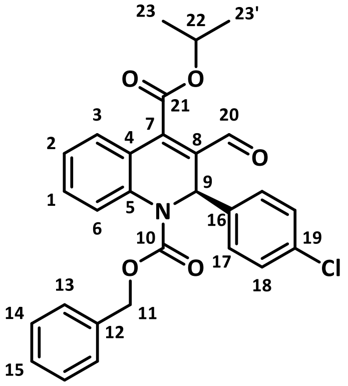

(*E*)-3-(4-Chlorophenyl)acrylaldehyde (25.0 mg, 0.15 mmol, 1.0 equiv.) and isopropyl 2-(2-(((benzyloxy)carbonyl)amino)phenyl)-2-oxoacetate (**15a**, 102.4 mg, 0.30 mmol, 2.0 equiv.) were subjected to **General Procedure A**, stirring at room temperature for 48 h. Purification by column chromatography (5% to 12% ethyl acetate in petroleum ether) gave the title compound as a yellow solid (67.4 mg, 0.14 mmol, 92%).

R_f: 0.3 (15% ethyl acetate in petroleum ether).

m.p.: 134.0 – 136.0 °C

$ν_{max}/cm^{-1}$ (thin film): 3067, 3035, 2987, 2936, 1710, 1673, 1619, 1600, 1567.

$δ_H$(500 MHz, $CDCl_3$): 9.87 (1H, s, C(20)**H**), 7.65 (1H, s, Ar**H**), 7.43 – 7.33 (7H, m, 7 x Ar**H**), 7.18 – 7.04 (5H, m, 5 x Ar**H**), 6.76 (1H, s, C(9)**H**), 5.42 (1H, hept, *J* = 6.5 Hz, C(22)**H**), 5.32 (2H, s, C(11) **HH'**), 1.43 (3H, d, *J* = 6.5 Hz, C(23)$\mathbf{H_3}$), 1.41 (3H, d, *J* = 6.5 Hz, C(23')$\mathbf{H_3}$)

$δ_C$(126 MHz, $CDCl_3$): 187.8 (**C**(20)), 165.1 (**C**(21)), 153.6 (**C**(10)), 143.7 (**C**(7)), 136.7 (**Ar**), 136.1 (**Ar**), 135.5 (**Ar**), 134.3 (**Ar**), 133.5 (**C**(8)), 132.2 (**Ar**), 128.9 (**Ar**), 128.8 (**Ar**), 128.7 (**Ar**), 128.5 (**Ar**), 128.4 (**Ar**), 126.6 (**Ar**), 125.3 (**Ar**), 125.0 (**Ar**), 123.4 (**Ar**), 71.3 (**C**(22)), 68.8 (**C**(11)), 51.5 (**C**(9)), 22.1 (**C**(23)), 22.0 (**C**(23'))

m/z HRMS (ESI$^+$) found 490.1415; $C_{28}H_{25}{}^{35}ClNO_5^+$ (M+H$^+$) requires 490.1416

ee: 98% (Chiralcel OD, 10% isopropanol in hexane, flow rate 0.8 mL/min; λ = 254 nm): t_R (major) = 13.3 min, t_R (minor) = 16.6 min

$[\alpha]_D^{25}$ = –447.6 (c = 1.0, $CHCl_3$)

1-Benzyl 4-isopropyl (*R*)-2-(4-acetoxy-3-methoxyphenyl)-3-formylquinoline-1,4(2H)-dicarboxylate: (–)-18n

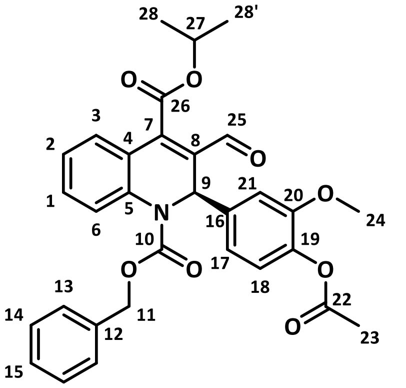

(*E*)-2-Methoxy-4-(3-oxoprop-1-en-1-yl)phenyl acetate (33.0 mg, 0.15 mmol, 1.0 equiv.) and isopropyl 2-(2-(((benzyloxy)carbonyl)amino)phenyl)-2-oxoacetate (**15a**, 102.4 mg, 0.30 mmol, 2.0 equiv.) were subjected to **General Procedure A**, stirring at room temperature for 49 h. Purification by column chromatography (20% to 25% ethyl acetate in petroleum ether) gave the title compound as a viscous yellow oil (73.2 mg, 0.13 mmol, 90%)

R_f: 0.4 (30% ethyl acetate in petroleum ether).

$ν_{max}/cm^{-1}$ (thin film): 3068, 3035, 2982, 2939, 1766, 1713, 1674, 1602, 1567, 1510.

$δ_H$(500 MHz, $CDCl_3$): 9.86 (1H, s, C(25)**H**), 7.67 (1H, s, Ar**H**), 7.46 – 7.29 (7H, m, 7 x Ar**H**), 7.15 (1H, td, *J* = 7.5, 1.0 Hz, Ar**H**), 6.82 – 6.77 (2H, m, 2 x Ar**H**), 6.76 (1H, s, C(9)**H**), 6.64 (1H, dd, *J* = 8.0, 2.0 Hz, Ar**H**), 5.42 (1H, hept, *J* = 6.5 Hz, C(27)**H**), 5.34 (1H, d, *J* = 12.0 Hz, C(11)**H**H'), 5.28 (1H, d, *J* = 12.0 Hz, C(11)H**H'**), 3.57 (3H, s, C(24)$\mathbf{H_3}$), 2.24 (3H, s, C(23)$\mathbf{H_3}$), 1.43 (3H, d, *J* = 6.5 Hz, C(28)$\mathbf{H_3}$), 1.41 (3H, d, *J* = 6.5 Hz, C(28')$\mathbf{H_3}$)

$δ_C$(126 MHz, $CDCl_3$): 187.8 (**C**(25)), 169.0 (**C**(22)), 165.2 (**C**(26)), 153.6 (**C**(10)), 151.0 (**Ar**), 143.5 (**C**(7)), 139.7 (**Ar**), 136.9 (**Ar**), 136.5 (**Ar**), 135.6 (**Ar**), 133.5 (**C**(8)), 132.2 (**Ar**), 128.8 (**Ar**), 128.6 (**Ar**), 128.6 (**Ar**), 126.6 (**Ar**), 125.4 (**Ar**), 124.9 (**Ar**), 123.4 (**Ar**), 122.8 (**Ar**), 119.2 (**Ar**), 111.6 (**Ar**), 71.2 (**C**(27)), 68.7 (**C**(11)), 55.7 (**C**(24)), 51.9 (**C**(9)), 22.1 (**C**(28)), 22.0 (**C**(28')), 20.8 (**C**(23))

m/z HRMS (ESI$^+$) found 566.1783; $C_{31}H_{29}NNaO_8^+$ (M+Na$^+$) requires 490.1416

ee: 98% (Chiralcel AD-H, 40% isopropanol in hexane, flow rate 0.8 mL/min; λ = 254 nm): t_R (major) = 13.9 min, t_R (minor) = 16.4 min

$[\alpha]_D^{25}$ = –364.8 (c = 1.0, $CHCl_3$)

1-Benzyl 4-isopropyl (*R*)-3-formyl-2-(4-nitrophenyl)quinoline-1,4(2H)-dicarboxylate: 18m

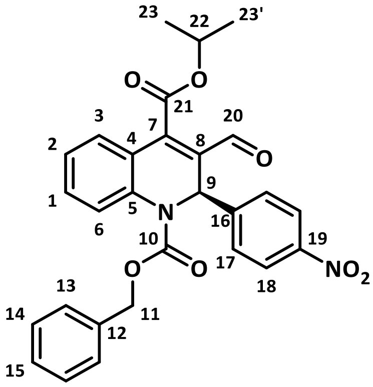

(*E*)-3-(4-Nitrophenyl)acrylaldehyde (26.6 mg, 0.15 mmol, 1.0 equiv.) and isopropyl 2-(2-(((benzyloxy)carbonyl)amino)phenyl)-2-oxoacetate (**15a**, 102.4 mg, 0.30 mmol, 2.0 equiv.) were subjected to **General Procedure A**, stirring at room temperature for 23 h. Purification by column chromatography (10% to 15% ethyl acetate in petroleum ether) gave the title compound as a yellow solid (68.3 mg, 0.13 mmol, 91%).

R_f: 0.3 (15% ethyl acetate in petroleum ether).

m.p.: 113.0 – 115.0 °C

ν_{max}/cm^{-1} (thin film): 3076, 3035, 2987, 2935, 2855, 1716, 1673, 1600, 1567, 1523, 1346.

δ_H(500 MHz, $CDCl_3$): 9.90 (1H, s, C(20)**H**), 8.09 – 8.00 (2H, m, 2 x Ar**H**), 7.73 – 7.62 (1H, m, Ar**H**), 7.47 – 7.33 (7H, m, 7 x Ar**H**), 7.32 – 7.28 (2H, m,2 x Ar**H**), 7.18 (1H, ddd, *J* = 8.0, 7.5, 1.0 Hz, Ar**H**), 6.87 (1H, s, C(9)**H**), 5.43 (1H, hept, *J* = 6.5 Hz, C(22)**H**), 5.33 (2H, s, C(11)**HH'**), 1.44 (3H, d, *J* = 6.5 Hz, C(23)$\mathbf{H_3}$), 1.42 (3H, d, *J* = 6.5 Hz, C(23)$\mathbf{H_3}$)

δ_C(126 MHz, $CDCl_3$): 187.7 (**C**(20)), 164.8 (**C**(21)), 153.5 (**C**(10)), 147.8 (**Ar**), 144.8 (**Ar**), 144.3 (**C**(7)), 136.6 (**Ar**), 135.3 (**Ar**), 132.8 (**C**(8)), 132.6 (**Ar**), 128.9 (**Ar**), 128.8 (**Ar**), 128.5 (**Ar**), 128.0 (**Ar**), 126.9 (**Ar**), 125.3 (**Ar**), 125.3 (**Ar**), 124.0 (**Ar**), 123.2 (**Ar**), 71.6 (**C**(22)), 69.0 (**C**(11)), 51.5 (**C**(9)), 22.1 (**C**(23)), 22.0 (**C**(23'))

m/z HRMS (ESI^+) found 501.1656; $C_{28}H_{25}N_2O_7^+$ ($M+H^+$) requires 501.1657

ee: 98% (Chiralcel AD-H, 40% isopropanol in hexane, flow rate 0.8 mL/min; λ = 254 nm): t_R (major) =17.6 min, t_R (minor) = 22.1 min

$[\alpha]_D^{25}$ = –431.3 (c = 1.0, CHC_3)

1-Benzyl 4-isopropyl (*R*)-3-formyl-2-(2-nitrophenyl)quinoline-1,4(2H)-dicarboxylate: (–)-18o

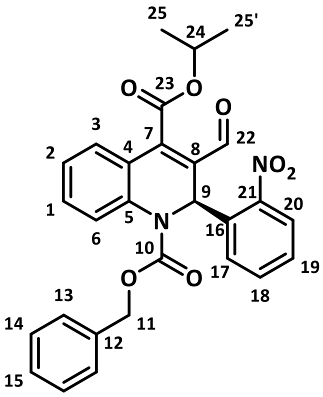

(*E*)-3-(2-Nitrophenyl)acrylaldehyde (26.6 mg, 0.15 mmol, 1.0 equiv.) and isopropyl 2-(2-(((benzyloxy)carbonyl)amino)phenyl)-2-oxoacetate (**15a**, 102.4 mg, 0.30 mmol, 2.0 equiv.) were subjected to **General Procedure A**, stirring at room temperature for 72 h. Purification by column chromatography (10% to 25% ethyl acetate in petroleum ether) gave the title compound as a yellow oil (52.8 mg, 0.11 mmol, 70%).

R_f: 0.3 (20% ethyl acetate in petroleum ether).

ν_{max}/cm^{-1} (thin film): 3067, 3035, 2987, 2937, 1718, 1676, 1600, 1567, 1532, 1359.

δ_H(500 MHz, $CDCl_3$): 9.86 (1H, s, C(22)**H**), 7.69 (1H, dd, *J* = 8.0, 1.5 Hz, Ar**H**), 7.52 (1H, dd, *J* = 8.5, 1.0 Hz, Ar**H**), 7.45 – 7.26 (10H, m, C(9)**H**, 9 x Ar**H**), 7.18 (1H, td, *J* = 7.5, 1.0 Hz, Ar**H**), 7.01 (1H, dd, *J* = 8.0, 1.5 Hz, Ar**H**), 5.43 (1H, hept, *J* = 6.5 Hz, C(24)**H**), 5.37 (1H, d, *J* = 12.0 Hz, C(11)**H**H'), 5.15 (1H, d, *J* = 12.0 Hz, C(11)H**H'**), 1.43 (3H, d, *J* = 6.5 Hz, C(24)**H₃**), 1.41 (3H, d, *J* = 6.5 Hz, C(24')**H₃**)

δ_C(126 MHz, $CDCl_3$): 187.3 (**C**(22)), 165.0 (**C**(23)), 153.6 (**C**(10)), 149.3 (**Ar**), 143.6 (**C**(7)), 136.4 (**Ar**), 135.5 (**Ar**), 133.2 (**C**(8)), 132.4 (**Ar**), 132.3 (**Ar**), 130.5 (**Ar**), 129.6 (**Ar**), 128.8 (**Ar**), 128.7 (**Ar**), 128.6 (**Ar**), 128.5 (**Ar**), 126.4 (**Ar**), 126.2 (**Ar**), 125.5 (**Ar**), 124.8 (**Ar**), 123.6 (**Ar**), 71.4 (**C**(24)), 68.9 (**C**(11)), 48.0 (**C**(9)), 22.0 (**C**(25)), 21.9 (**C**(25'))

m/z HRMS (ESI⁺) found 501.1656; $C_{28}H_{25}N_2O_7^+$ (M+H⁺) requires 501.1657

ee: 96% (Chiralcel AD-H, 40% isopropanol in hexane, flow rate 0.8 mL/min; λ = 254 nm): t_R (major) =24.3 min, t_R (minor) = 29.1 min

$[\alpha]_D^{25}$ = –225.8 (c = 1.0, $CHCl_3$).

1-Benzyl 4-isopropyl (*R*)-3-formyl-2-(4-methoxyphenyl)quinoline-1,4(2H)-dicarboxylate: (–)-18l

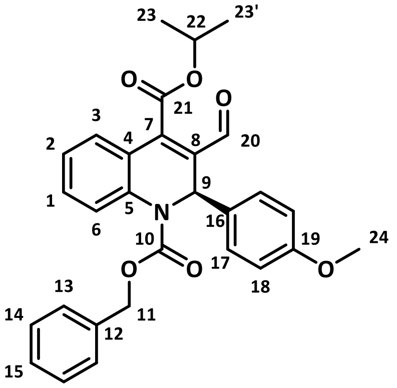

(*E*)-3-(4-Methoxyphenyl)acrylaldehyde (24.3 mg, 0.15 mmol, 1.0 equiv.) and isopropyl 2-(2-(((benzyloxy)carbonyl)amino)phenyl)-2-oxoacetate (**15a**, 102.4 mg, 0.30 mmol, 2.0 equiv.) were subjected to **General Procedure A**, stirring at room temperature for 165 h. Purification by column chromatography (10% to 15% ethyl acetate in petroleum ether) gave the title compound as a yellow solid (54.2 mg, 0.11 mmol, 74%).

R_f: 0.3 (15% ethyl acetate in petroleum ether).

m.p.: 160.0 – 162.0 °C

ν_{max}/cm^{-1} (thin film): 3066, 3035, 2982, 2935, 2838, 1709, 1674, 1609, 1601, 1585, 1567, 1511.

δ_H(500 MHz, $CDCl_3$): 9.86 (1H, s, C(20)**H**), 7.74 – 7.59 (1H, m, Ar**H**), 7.45 – 7.32 (7H, m, 7 x Ar**H**), 7.14 (1H, td, *J* = 7.5, 1.0 Hz, Ar**H**), 7.10 – 7.04 (2H, m, 2 x Ar**H**), 6.75 (1H, s, C(9)**H**), 6.73 – 6.65 (2H, m, 2 x Ar**H**), 5.42 (1H, hept, *J* = 6.5 Hz, C(22)**H**), 5.31 (2H, s, C(11)**HH'**), 3.70 (3H, s, C(24)**H₃**), 1.43 (3H, d, *J* = 6.5 Hz, C(23)**H₃**), 1.41 (3H, d, *J* = 6.5 Hz, C(23')**H₃**)

δ_C(126 MHz, $CDCl_3$): 187.8 (**C**(20)), 165.3 (**C**(21)), 159.6 (**Ar**), 153.7 (**C**(10)), 143.2 (**C**(7)), 136.9 (**Ar**), 135.7 (**Ar**), 134.0 (**C**(8)), 132.0 (**Ar**), 129.7 (**Ar**), 128.8 (**Ar**), 128.5 (**Ar**), 128.4 (**Ar**), 128.4 (**Ar**), 126.4 (**Ar**), 125.4 (**Ar**), 124.8 (**Ar**), 123.6 (**Ar**), 114.1 (**Ar**), 71.1 (**C**(22)), 68.6 (**C**(11)), 55.3 (**C**(24)), 51.7 (**C**(9)), 22.1 (**C**(23)), 22.0 (**C**(23'))

m/z HRMS (ESI⁺) found 508.1730; $C_{29}H_{27}NNaO_6^+$ (M+Na⁺) requires 508.1731

ee: 93% (Chiralcel AD-H, 40% isopropanol in hexane, flow rate 0.8 mL/min; λ = 254 nm): t_R (minor) = 16.2 min, t_R (major) = 19.2 min

$[\alpha]_D^{25}$ = –466.8 (c = 1.0, $CHCl_3$)

1-Benzyl 4-isopropyl (*R*)-3-formyl-2-(2-methoxyphenyl)quinoline-1,4(2H)-dicarboxylate: (–)-18p

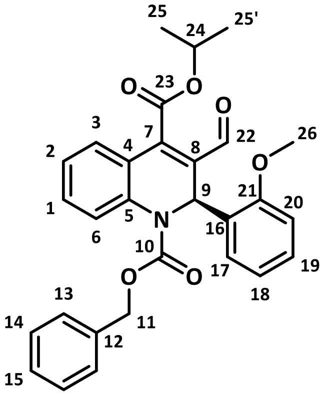

(*E*)-3-(2-Methoxyphenyl)acrylaldehyde (24.3 mg, 0.15 mmol, 1.0 equiv.) and isopropyl 2-(2-(((benzyloxy)carbonyl)amino)phenyl)-2-oxoacetate (**15a**) (102.4 mg, 0.30 mmol, 2.0 equiv.) were subjected to **General Procedure A**, stirring at room temperature for 172 h. Purification by column chromatography (15% to 25% ethyl acetate in petroleum ether) gave the title compound as a yellow oil (28.5 mg, 0.06 mmol, 39%).

R_f: 0.3 (20% ethyl acetate in petroleum ether).

$ν_{max}/cm^{-1}$ (thin film): 3067, 3033, 2981, 2937, 2839, 2745, 1775, 1714, 1676, 1599, 1567.

$δ_H$(500 MHz, $CDCl_3$): 9.83 (1H, s, C(22)**H**), 7.68 – 7.63 (1H, m, Ar**H**), 7.47 – 7.31 (7H, m, 7 x Ar**H**), 7.19 – 7.12 (2H, m, 2 x Ar**H**), 7.11 (1H, s, C(9)**H**), 6.90 (1H, dd, *J* = 7.5, 1.5 Hz, Ar**H**), 6.78 (1H, dd, *J* = 8.2, 1.1 Hz, Ar**H**), 6.68 (1H, td, *J* = 7.5, 1.5 Hz, Ar**H**), 5.41 (1H, hept, *J* = 6.5 Hz, C(24)**H**), 5.31 (1H, d, *J* = 12.5 Hz, C(11)**H**H’), 5.27 (1H, d, *J* = 12.5 Hz, C(11)H**H’**), 3.57 (3H, s, C(26)**H₃**), 1.43 (3H, d, *J* = 6.5 Hz, C(25)**H₃**), 1.40 (3H, d, *J* = 6.5 Hz, C(25’)**H₃**)

$δ_C$(126 MHz, $CDCl_3$, 26 resonances out of 27 observed, 1 x Ar resonance not observed): 187.9 (**C**(22)), 165.6 (**C**(21)), 157.6 (**Ar**), 153.8 (**C**(10)), 142.5 (**C**(7)), 137.4 (**Ar**), 136.0 (**Ar**), 133.8 (**C**(8)), 131.5 (**Ar**), 129.7 (**Ar**), 128.7 (**Ar**), 128.3 (**Ar**), 128.3 (**Ar**), 126.1 (**Ar**), 125.7 (**Ar**), 125.0 (**Ar**), 124.7 (**Ar**), 124.3 (**Ar**), 120.3 (**Ar**), 111.3 (**Ar**), 70.8 (**C**(24)), 68.3 (**C**(11)), 55.3 (**C**(26)), 48.6 (**C**(9)), 22.0 (**C**(25)), 21.9 (**C**(25’))

m/z HRMS (ESI^+) found 486.1910; $C_{29}H_{28}NO_6^+$ ($M+H^+$) requires 486.1911

ee: 91% (Chiralcel AD-H, 20% isopropanol in hexane, flow rate 0.8 mL/min; λ = 254 nm): t_R (minor) =20.5 min, t_R (major) = 27.0 min

$[\alpha]_D^{25}$ = –349.5 (c = 1.0, $CHCl_3$)

1-Benzyl 4-isopropyl (*S*)-2-(2-bromophenyl)-3-formylquinoline-1,4(2H)-dicarboxylate: (–)-18q

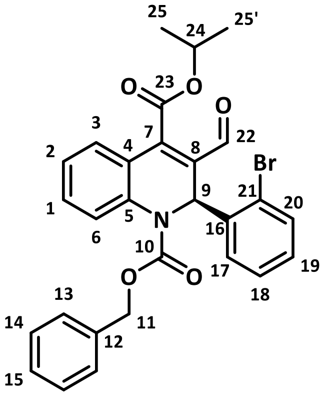

(*E*)-3-(2-Bromophenyl)acrylaldehyde (**16r**, 31.7 mg, 0.15 mmol, 1.0 equiv.) and isopropyl 2-(2-(((benzyloxy)carbonyl)amino)phenyl)-2-oxoacetate (**15a**) (102.4 mg, 0.30 mmol, 2.0 equiv.) were subjected to **General Procedure A**, stirring at room temperature for 93 h. Purification by column chromatography (10% to 15% ethyl acetate in petroleum ether) gave the title compound as a yellow oil (32.3 mg, 0.06 mmol, 40%).

R_f: 0.4 (20% ethyl acetate in petroleum ether).

v_{max}/cm^{-1} (thin film): 3066, 3033, 2982, 2934, 2873, 2855, 1716, 1676, 1600, 1567.

δ_H(500 MHz, $CDCl_3$): 9.86 (1H, s, C(22)**H**), 7.59 – 7.50 (2H, m, 2 x Ar**H**), 7.44 (1H, dd, *J* = 8.0, 1.5 Hz, Ar**H**), 7.42 – 7.30 (6H, m, 6 x Ar**H**), 7.20 (1H, td, *J* = 7.5, 1.5 Hz, Ar**H**), 7.14 (1H, s, C(9)**H**), 7.04 (1H, td, *J* = 7.5, 1.5 Hz, Ar**H**), 6.98 (1H, td, *J* = 7.5, 1.5 Hz, Ar**H**), 6.83 (1H, dd, *J* = 7.5, 1.5 Hz, Ar**H**), 5.43 (1H, hept, *J* = 6.5 Hz, C(24)**H**), 5.36 (1H, d, *J* = 12.5 Hz, C(11)**H**H'), 5.24 (1H, d, *J* = 12.5 Hz, C(11)H**H'**), 1.44 (3H, d, *J* = 6.5 Hz, C(25)$\mathbf{H_3}$), 1.42 (3H, d, *J* = 6.5 Hz, C(25')$\mathbf{H_3}$)

δ_C(126 MHz, $CDCl_3$): 187.4 (**C**(22)), 165.1 (**C**(23)), 153.7 (**C**(10)), 143.3 (**C**(13)), 136.8 (**Ar**), 135.8 (**Ar**), 135.7 (**Ar**), 134.9 (**C**(8)), 134.2 (**Ar**), 131.9 (**Ar**), 130.1 (**Ar**), 128.8 (**Ar**), 128.7 (**Ar**), 128.5 (**Ar**), 128.4 (**Ar**), 127.5 (**Ar**), 127.0 (**Ar**), 126.2 (**Ar**), 125.6 (**Ar**), 124.6 (**Ar**), 123.7 (**Ar**), 71.2 (**C**(24)), 68.6 (**C**(11)), 52.0 (**C**(9)), 22.1 (**C**(25)), 22.0 (**C**(25'))

m/z HRMS (ESI^+) found 556.0731; $C_{28}H_{24}{}^{79}BrNNaO_5{}^+$ ($M+Na^+$) requires 556.0730

ee: 96% (Chiralcel OD, 7% isopropanol in hexane, flow rate 0.8 mL/min; λ = 254 nm): t_R (major) = 18.0 min, t_R (minor) = 22.4 min

$[\alpha]_D^{25}$ = –342.5 (c = 1.0, $CHCl_3$)

Total Synthesis of Sealutomicin C

1-Benzyl 4-isopropyl (*S*)-2-(2-bromophenyl)-3-formyl-6-methoxyquinoline-1,4(2*H*)-dicarboxylate: (–)-18r

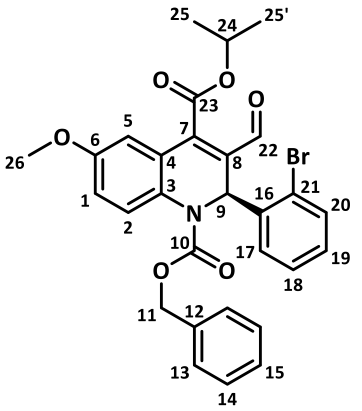

To a solution of (*S*)-2-(((triethylsilyl)oxy)diphenylmethyl)pyrrolidine (**(*S*)-17a**, 804 mg, 2.19 mmol, 0.2 equiv.) in dichloromethane (13 mL) was added 2-bromocinnamaldehyde (**16r**, 2.30 g, 10.9 mmol, 1.0 equiv.), benzoic acid (614 mg, 5.48 mmol, 0.5 eq), freshly activated 4 Å molecular sieves (4.000 g) and isopropyl 2-(2-(((benzyloxy)carbonyl)amino)-5-methoxyphenyl)-2-oxoacetate (**15r**, 8.13 g, 21.9 mmol, 2.0 equiv.). The reaction was stirred at RT for 72 h then filtered through Celite™ and washed with ethyl acetate. The filtrate was washed sequentially with sat. aq sodium bicarbonate and hydrochloric acid (2 M) and the combined organic fractions were dried over magnesium sulfate, concentrated *in vacuo* and purified by flash column chromatography (10% to 30% ethyl acetate in petroleum ether) to give the title compound as a yellow solid (4.92 g, 8.73 mmol, 80%).

R_f: 0.5 (30% ethyl acetate in petroleum ether).

m.p.: 43.8 – 48.4 °C.

ν_{max}/cm^{-1} (thin film): 2982 (w), 1714 (s), 1676 (m), 1493 (m), 1254 (s), 1234 (s), 1212 (s), 1101 (m), 1025 (m).

δ_H(500 MHz, $CDCl_3$): 9.85 (1H, s, C(22)**H**), 7.54 (1H, dd, *J* = 7.5, 1.5 Hz, Ar**H**), 7.47 – 7.29 (6H, m, 6 x Ar**H**), 7.11 (1H, s, C(9)**H**), 7.04 (1H, td, *J* =7.5, 1.5 Hz, Ar**H**), 6.99 (1H, td, *J* = 7.5, 1.5 Hz, Ar**H**), 6.96 (1H, d, *J* = 3.0 Hz, Ar**H**), 6.90 (1H, dd, *J* = 9.0, 3.0 Hz, Ar**H**), 6.80 (1H, dd, *J* = 8.0, 2.0 Hz, Ar**H**), 5.43 (1H, hept, *J* = 6.5 Hz, C(24)**H**), 5.34 (1H, d, *J* = 12.5 Hz, C(11)**H**H'), 5.22 (1H, d, *J* = 12.5 Hz, C(11)H**H'**), 3.80 (3H, s, C(26)$\mathbf{H_3}$), 1.44 (3H, d, *J* = 6.5 Hz, C(25)$\mathbf{H_3}$), 1.42 (3H, d, *J* = 6.5 Hz, C(25')$\mathbf{H_3}$).

δ_C(126 MHz, $CDCl_3$): 187.3 (**C**(22)), 165.0 (**C**(23)), 156.9 (**Ar**), 153.9 (**C**(10)), 143.1 (**C**(7)), 135.8 (**Ar**), 135.5 (**C**(8)), 135.3 (**Ar**), 134.1 (**Ar**), 130.0 (**Ar**), 129.8 (**Ar**), 128.8 (**Ar**), 128.6 (**Ar**), 128.3 (**Ar**), 128.3 (**Ar**), 128.1 (**Ar**), 127.4 (**Ar**), 125.4 (**Ar**), 123.8 (**Ar**), 117.7 (**Ar**), 110.4 (**Ar**), 71.1 (**C**(24)), 68.4 (**C**(11)), 55.6 (**C**(26)), 52.0 (**C**(9)), 22.0 (**C**(25)), 21.9 (**C**(25')).

m/z HRMS (ESI^+) found 564.1016; $C_{29}H_{27}NO_6{}^{79}Br^+$ ($M+H^+$) requires 564.1016.

ee: 98% (Chiracel OD, 5% isopropanol in hexane, flow rate 0.8 mL/min; λ = 254 nm): t_R (major) = 28.7 min, t_R (minor) = 33.8 min.

$[\alpha]_D^{25}$ = –487.0 ($CHCl_3$)

A sample of the enantiomer, 1-benzyl 4-isopropyl (*R*)-2-(2-bromophenyl)-3-formyl-6-methoxyquinoline-1,4(2*H*)-dicarboxylate (**(+)-18r**, 7.17 g, 12.7 mmol, 89%), was prepared as above using (*R*)-2-(((triethylsilyl)oxy)diphenylmethyl) pyrrolidine (**(*R*)-17a**) in place of (*S*)-2-(((triethylsilyl)oxy)diphenylmethyl) pyrrolidine (**(*S*)-17a**).

ee: 98% (Chiracel OD, 5% isopropanol in hexane, flow rate 0.8 mL/min; λ = 254 nm): t_R (minor) = 29.5 min, t_R (major) = 33.1 min.

$[\alpha]_D^{25}$ = +336.5 ($CHCl_3$)

All other characterisation identical.

A racemic sample of ***rac*-18r** (22.1 mg, 0.04 mmol, 16%) was prepared as described above using catalytic pyrrolidine in place of (S)-2-(((triethylsilyl)oxy)diphenylmethyl) pyrrolidine.

Benzyl (*R*)-4-(2-bromophenyl)-8-methoxy-1-oxo-1,4-dihydrofuro[3,4-c]quinoline-5(3*H*)-carboxylate: 23

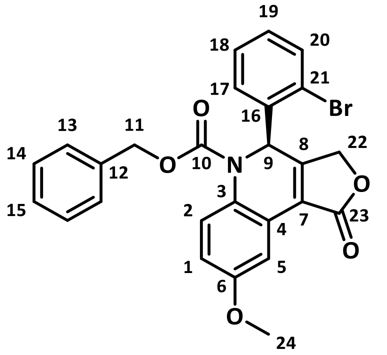

Prepared according to the procedure of Janthawornpong.[60]

To a solution of 1-benzyl 4-isopropyl (*R*)-2-(2-bromophenyl)-3-formyl-6-methoxyquinoline-1,4(2*H*)-dicarboxylate (**(+)-18r**, 8.371 g, 14.87 mmol, 1.0 equiv.) and cerium(III) trichloride heptahydrate (5.540 g, 14.87 mmol, 1.0 equiv.) in anhydrous methanol (150 mL) was added sodium borohydride (562.4 mg, 14.87 mmol, 1.0 equiv.) *[Note: sonication is required to fully dissolve the dihydroquinoline starting material]*. The reaction was stirred at RT for 60 min then quenched with brine (100 mL). The methanol was removed *in vacuo* and ethyl acetate (300 mL) was added to the resultant aqueous suspension. The layers were separated, the aqueous layer was washed with ethyl acetate (x 3) and the combined organic fractions were dried over sodium sulfate, concentrated *in vacuo* and purified by flash column chromatography (10% to 20% ethyl acetate in pentane) to give the title compound as a yellow solid (7.138 g, 14.13 mmol, 95%).

R_f: 0.6 (30% ethyl acetate in petroleum ether).

m.p.: 50.1 – 55.5 °C.

ν_{max}/cm^{-1} (thin film): 2998 (s), 2886 (m), 1762 (s), 1717 (s), 1382 (s), 1262 (s), 954 (m).

δ_H(500 MHz, $CDCl_3$): 7.91-7.60 (1H, br, Ar**H**), 7.75 (1H, d, *J* = 3.0 Hz, Ar**H**), 7.58 – 7.54 (1H, m, Ar**H**), 7.37 – 7.27 (5H, m, 5 x Ar**H**), 7.19 – 7.11 (3H, m, 3 x Ar**H**), 6.99 (1H, s, C(9)**H**), 6.93 (1H, dd, *J* = 9.0, 3.0 Hz, Ar**H**), 5.28 (1H, d, *J* = 12.5 Hz, C(11)**H**H'), 5.19 (1H, d, *J* = 12.5 Hz, C(11)H**H'**), 5.04 (1H, d, *J* = 18.0 Hz, C(22)**H**H'), 4.86 (1H, d, *J* = 18.0 Hz, C(22)H**H'**), 3.87 (3H, s, C(24)$\mathbf{H_3}$).

δ_C(126 MHz, $CDCl_3$): 169.7 (**C**(23)), 157.5 (**C**(8)), 156.7 (**Ar**), 154.4 (**C**(10)), 138.8 (**C**(7)), 135.6 (**Ar**), 133.2 (**Ar**), 130.5 (**Ar**), 129.0 (**Ar**), 128.7 (**Ar**), 128.7 (**Ar**), 128.5 (**Ar**), 128.4 (**Ar**), 128.3 (**Ar**), 127.9 (**Ar**), 125.4 (**Ar**) 120.8 (**Ar**), 120.5 (**Ar**), 116.2 (**Ar**), 108.1 (**Ar**), 69.5 (**C**(22)), 68.7 (**C**(11)), 56.5 (**C**(9)), 55.8 (**C**(24)).

m/z HRMS (ESI^+) found 528.0417; $C_{26}H_{20}NO_5{}^{79}BrNa^+$ ($M+Na^+$) requires 528.0417.

$[\alpha]_D^{25}$ = +558.8 (*c* = 0.81, $CHCl_3$).

Benzyl (3a*R*,4*S*,9b*R*)-4-(2-bromophenyl)-3a,9b-dihydroxy-8-methoxy-1-oxo-1,3a,4,9b-tetrahydrofuro[3,4-c]quinoline-5(3*H*)-carboxylate: 24

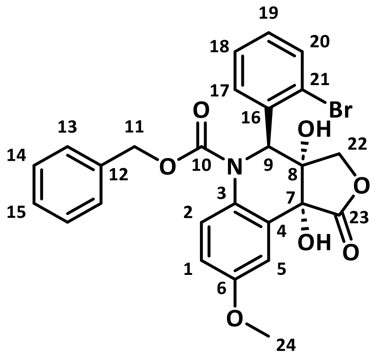

Prepared according to the procedure of Niggemann.[10]

A solution of cerium(III) trichloride heptahydrate (402.4 mg, 1.08 mmol, 0.1 equiv.) and sodium periodate (3.478 mg, 16.26, mmol, 1.5 equiv.) in water (9 mL) was heated to 35 °C for 10 min until the solution turned pale yellow in colour. The solution was cooled to 0 °C and ethyl acetate (18 mL) and acetonitrile (28 mL) were added. Ruthenium(III) trichloride trihydrate (141.2 mg, 0.54 mmol, 0.05 equiv.) was added as a single solid portion and the suspension was stirred for 2 min. A solution of benzyl (*S*)-4-(2-bromophenyl)-8-methoxy-1-oxo-1,4-dihydrofuro[3,4-c]quinoline-5(3*H*)-carboxylate (**23**, 5.475 g, 10.84 mmol, 1.0 equiv.) in ethyl acetate (10 mL) and the reaction was stirred at 0 °C for 10 min. Sodium sulfate (15 g) was added and the reaction mixture was filtered through Celite™ and washed with ethyl acetate. The filtrate was washed with sat. aq. sodium sulfite, dried over sodium sulfate and concentrated *in vacuo*. The crude material was purified by flash column chromatography (20% to 30% ethyl acetate in pentane) to give the title compound as a white solid (5.177 g, 9.568 mmol, 88%).

R_f: 0.2 (30% ethyl acetate in petroleum ether).

m.p.: 64.1 – 69.0 °C.

ν_{max}/cm^{-1} (thin film): 3407 (br,m), 2964 (m), 1788 (w), 1700 (m), 1504 (s), 1299 (m), 1270 (m), 1150 (m), 1021 (m), 748 (m).

δ_H(600 MHz, $CDCl_3$, C(7)O**H** and C(8)O**H** not observed): 7.74 (1H, d, *J* = 9.0 Hz, Ar**H**), 7.59 (1H, dd, *J* = 8.0, 1.5 Hz, Ar**H**), 7.28 – 7.24 (3H, m, 3 x Ar**H**), 7.18 (1H, td, *J* = 7.5, 1.5 Hz, Ar**H**), 7.15 (1H, dd, *J* = 7.5, 2.0 Hz, Ar**H**), 7.14 – 7.11 (2H, m, 2 x Ar**H**), 7.04 (1H, dd, *J* = 9.0, 3.0 Hz, Ar**H**), 7.03 (1H, d, *J* = 7.5 Hz, Ar**H**), 6.96 (1H, d, *J* = 3.0 Hz, Ar**H**), 6.18 (1H, s, C(9)**H**), 5.15 (1H, d, *J* = 12.5 Hz, C(11)**H**H'), 5.13 (1H, d, *J* = 12.5 Hz, C(11)H**H'**), 3.81 (3H, s, C(24)$\mathbf{H_3}$), 3.67 (1H, d, *J* = 10.0 Hz, C(22)**H**H'), 3.60 (1H, d, *J* = 10.0 Hz, C(22)H**H'**).

δ_C(151 MHz, $CDCl_3$): 174.5 (**C**(23)), 157.4 (**Ar**), 154.3 (**C**(10)), 138.1 (**Ar**), 135.6 (**Ar**), 133.9 (**Ar**), 130.5 (**Ar**), 129.8 (**Ar**), 128.6 (**Ar**), 128.3 (**Ar**), 128.1 (**Ar**), 128.0 (**Ar**), 127.4 (**Ar**), 127.2 (**Ar**), 125.6 (**Ar**), 123.7 (**Ar**), 117.6 (**Ar**), 111.5 (**Ar**), 79.8 (**C**(8)), 75.5 (**C**(7)), 72.5 (**C**(22)), 68.5 (**C**(11)), 61.7 (**C**(9)), 55.8 (**C**(24)).

m/z HRMS (ESI$^+$) found 540.0653; $C_{26}H_{23}NO_7{}^{79}Br^+$ (M+H$^+$) requires 540.0652.

$[\alpha]_D^{25}$ = +60.2 (*c* = 0.95, $CHCl_3$).

Benzyl (3a*R*,4*S*,9b*R*)-4-(2-bromophenyl)-8-methoxy-3a,9b-bis((4-methoxybenzyl)oxy)-1-oxo-1,3a,4,9b-tetrahydrofuro[3,4-c]quinoline-5(3*H*)-carboxylate: 25

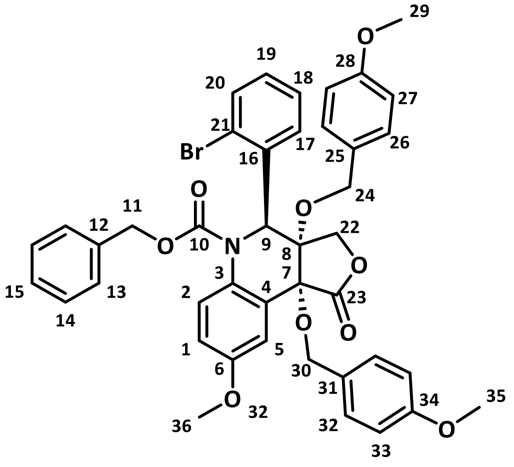

Preparation of 4-methoxybenzyl bromide according to the procedure of Burke.[11]

To a mixture of hydrobromic acid (48% in water, 4.5 mL) and diethyl ether (5 mL) at RT was added a solution of 4-methoxybenzyl alcohol (3.00 g, 21.9 mmol) in diethyl ether (5 mL). The reaction mixture was stirred vigorously for 2 h at RT then diluted with diethyl ether (25 mL) and the layers were separated. The organic layer was washed sequentially with sat. aq. sodium bicarbonate and sat. aq. sodium bromide and then dried over magnesium sulfate and concentrated *in vacuo* at 0 °C to give 4-methoxybenzyl bromide as a colourless oil (3.95 g, 19.7 mmol, 90%). The crude product was used immediately in subsequent procedures without further purification.

Preparation of 25

To a solution of benzyl (3a*R*,4*S*,9b*R*)-4-(2-bromophenyl)-3a,9b-dihydroxy-8-methoxy-1-oxo-1,3a,4,9b-tetrahydrofuro[3,4-c]quinoline-5(3*H*)-carboxylate (**24**, 1.00 g, 1.85 mmol, 1.0 equiv.) in dimethylformamide (25 mL) at 0 °C was added dropwise sodium bis(trimethylsilyl)amide (1 M in tetrahydrofuran, 5.6 mL, 5.60 mmol, 3.0 equiv.). The reaction was warmed to RT and stirred for 30 min. 4-methoxybenzyl bromide (1.34 mL, 9.30 mmol, 5.0 equiv.) was added dropwise and the reaction was stirred for 12 h at RT. The reaction was then cooled to 0 °C and quenched by dropwise addition of sat. aq. ammonium chloride (20 mL). The mixture was extracted with ethyl acetate (x 3) and the combined organic fractions were dried over sodium sulfate, concentrated *in vacuo* and purified by flash column chromatography (2% to 4% diethyl ether in toluene) to give the title compound as a white solid (1.998 g, 1.411 mmol, 76%).

R_f: 0.8 (10% diethyl ether in toluene).

m.p.: 53.4 – 56.8 °C.

ν_{max}/cm^{-1} (thin film): 1791 (m), 1709 (m), 1613 (m), 1514 (s), 1586 (s), 1302 (m), 1250 (s), 1030 (s)

δ_H(600 MHz, $CDCl_3$): 7.69 (1H, d, *J* = 9.0 Hz, Ar**H**), 7.56 (1H, dd, *J* = 7.5, 1.5 Hz, Ar**H**), 7.29 – 7.21 (6H, m, 6 x Ar**H**), 7.18 – 7.13 (2H, m, 2 x Ar**H**), 7.14 – 7.06 (3H, m, 3 x Ar**H**), 7.06 – 7.00 (2H, m, 2 x Ar**H**), 6.87 (1H, dd, *J* = 7.5, 2.0 Hz, Ar**H**), 6.82 – 6.78 (2H, m, 2 x Ar**H**), 6.77 – 6.70 (2H, m, 2 x Ar**H**), 6.73 (1H, s, C(9)**H**), 5.25 (1H, d, *J* = 12.5 Hz, C(11)**H**H'), 5.13 (1H, d, *J* = 12.5 Hz, C(11)H**H'**), 5.03 (1H, d, *J* = 10.0 Hz, C(24)**H**H'), 4.80 (1H, d, *J* = 10.0 Hz, C(24)H**H'**), 4.77 (1H, d, *J* = 10.5 Hz, C(30)**H**H'), 4.32 (1H, d, *J* = 10.5 Hz, C(30)H**H'**), 4.01 (1H, d, *J* = 10.0 Hz, C(22)**H**H'), 3.99 (1H, d, *J* = 10.0 Hz, C(22)H**H'**), 3.84 (3H, s, C(36)$\mathbf{H_3}$), 3.80 and 3.76 (2 x 3H, s, C(29)$\mathbf{H_3}$ and C(35)$\mathbf{H_3}$).

δ_C(151 MHz, $CDCl_3$, 35 resonances out of 36 observed, 1 x **Ar**, resonance not observed): 173.1 (**C**(23)), 159.2 (**Ar**), 157.1 (**Ar**), 154.4 (**C**(10)), 137.4 (**Ar**), 135.8 (**Ar**), 133.9 (**Ar**), 131.9 (**Ar**), 130.2 (**Ar**), 130.0 (**Ar**), 130.0 (**Ar**), 129.6 (**Ar**), 129.4 (**Ar**), 128.6 (**Ar**), 128.4 (**Ar**), 128.3 (**Ar**), 128.2 (**Ar**), 128.0 (**Ar**), 127.4 (**Ar**), 124.5 (**Ar**), 124.2 (**Ar**), 117.5 (**Ar**), 113.8 (**Ar**), 113.7 (**Ar**), 113.2 (**Ar**), 84.8 (**C**(8)), 80.4 (**C**(7)), 71.7 (**C**(22)), 68.4 (**C**(11)), 68.3 (**C**(30)), 67.6 (**C**(24)), 58.3 (**C**(9)), 55.8 (**C**(36)), 55.4 and 55.4 (**C**(29) and **C**(35)).

m/z HRMS (ESI$^+$) found 802.1617; $C_{42}H_{38}NO_9{}^{79}BrNa^+$ (M+Na$^+$) requires 802.1622.

$[\alpha]_D^{25}$ = –64.3 (c = 0.58, $CHCl_3$)

Benzyl (6*S*,12*R*,13*R*)-13-(hydroxymethyl)-2-methoxy-12,13-bis((4-methoxybenzyl)oxy)-11-oxo-11,12-dihydro-6,12-methanodibenzo[*b*,*f*]azocine-5(6*H*)-carboxylate: 26

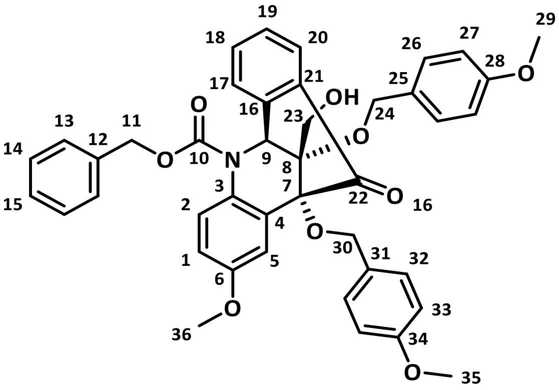

To a solution of benzyl (3a*R*,4*S*,9b*R*)-4-(2-bromophenyl)-8-methoxy-3a,9b-bis((4-methoxybenzyl)oxy)-1-oxo-1,3a,4,9b-tetrahydrofuro[3,4-c]quinoline-5(3*H*)-carboxylate (**25**, 1.096 g, 1.41 mmol, 1.0 equiv.) in tetrahydrofuran (27 mL) at –78 °C was added dropwise *n*-butyllithium (1.6 M in hexane, 1.05 mL, 1.688 mmol, 1.2 equiv.). The reaction was stirred at –78 °C for 5 min then warmed to RT and stirred for a further 30 min. The reaction was then cooled to 0 °C and quenched by dropwise addition of sat. aq. ammonium chloride solution (20 mL). The mixture was extracted with ethyl acetate (x 3) and the combined organic fractions were dried over sodium sulfate, concentrated *in vacuo* and purified by flash column chromatography (5% to 10% ethyl acetate in toluene) to give the title compound as a white solid (924.1 mg, 1.318 mmol, 94%).

*At room temperature, compound **26** exists as a 2 : 1 inseparable mixture of the primary alcohol (shown) and the corresponding lactol. The ^{1}H and ^{13}C NMR spectra of the mixture at room temperature display broad resonances due to the presence of rotamers. Upon heating to 105 °C in DMSO-d_6, the resonances in both the ^{1}H and the ^{13}C NMR spectra coalesce. At this temperature, the ratio of primary alcohol : lactol is 5 : 1. NMR data is given for the primary alcohol form only, taken from the NMR spectra of the mixture at 105 °C.*

R_f: 0.3 (10% ethyl acetate in toluene).

m.p.: 63.1 – 65.4 °C.

ν_{max}/cm^{-1} (thin film): 1699 (m), 1690 (m), 1515 (s), 1500 (m), 1301 (m), 1250 (s), 1230 (m), 1035 (m).

δ_H(500 MHz, DMSO, 378 K): 7.87 – 7.76 (1H, m, Ar**H**), 7.72 – 7.57 (3H, m, 3 x Ar**H**), 7.49 – 7.31 (8H, m, 8 x Ar**H**), 7.12 – 7.07 (2H, m, 2 x Ar**H**), 6.97 – 6.91 (2H, m, 2 x Ar**H**), 6.87 (1H, d, *J* = 3.0 Hz, Ar**H**), 6.81 – 6.78 (2H, m, 2 x Ar**H**), 6.75 (1H, dd, *J* = 9.0, 3.0 Hz, Ar**H**), 6.60 (1H, s, C(9)**H**), 5.34 (1H, d, *J* = 12.0 Hz, C(11)**H**H'), 5.27 (1H, d, *J* = 12.0 Hz, C(11)H**H'**), 4.93 (1H, d, *J* = 12.0 Hz, C(30)**H**H'), 4.87 (1H, t, *J* = 4.5 Hz, C(23)O**H**), 4.85 (1H, d, *J* = 11.0 Hz, C(24)**H**H'), 4.67 (1H, d, *J* = 11.0 Hz, C(24)H**H'**), 4.56 (1H, d, *J* = 12.0 Hz, C(30)H**H'**), 4.26 (1H, dd, *J* = 12.5, 4.5 Hz, C(23)**H**H'), 3.79 and 3.72 (2 x 3H, s, C(29)$\mathbf{H_3}$ and C(35)$\mathbf{H_3}$), 3.60 (1H, dd, *J* = 12.5, 4.5 Hz, C(23)H**H'**), 3.57 (3H, s, C(36)$\mathbf{H_3}$).

δ_C(126 MHz, DMSO, 378K): 190.0 (**C**(22)), 158.3 (**Ar**), 158.3 (**Ar**), 155.1 (**Ar**), 153.4 (**C**(10)), 136.8 (**Ar**), 135.4 (**Ar**), 134.0 (**Ar**), 131.3 (**Ar**), 130.4 (**Ar**), 130.1 (**Ar**), 129.4 (**Ar**), 128.9 (**Ar**), 128.4 (**Ar**), 128.2 (**Ar**), 128.0 (**Ar**), 127.9 (**Ar**), 127.6 (**Ar**), 127.6 (**Ar**), 125.6 (**Ar**), 123.8 (**Ar**), 123.7 (**Ar**), 114.8 (**Ar**), 113.4 (**Ar**), 113.3 (**Ar**), 110.5 (**Ar**), 84.4 (**C**(7)), 76.1 (**C**(8)), 67.9 (**C**(30)), 67.4 (**C**(11)), 64.8 (**C**(24)), 61.7 (**C**(23)), 54.9 (**C**(9)), 54.8 (**C**(36)), 54.7 and 54.6 (**C**(29) and **C**(35)).

m/z HRMS (ESI$^+$) found 724.2513; $C_{42}H_{39}NO_9Na^+$ (M+Na$^+$) requires 724.2517.

$[\alpha]_D^{25}$ = +102.8 (c = 0.52, $CHCl_3$)

Benzyl (6S,12R,13S)-13-formyl-2-methoxy-12,13-bis((4-methoxybenzyl)oxy)-11-oxo-11,12-dihydro-6,12-methanodibenzo[*b,f*]azocine-5(6*H*)-carboxylate: 27

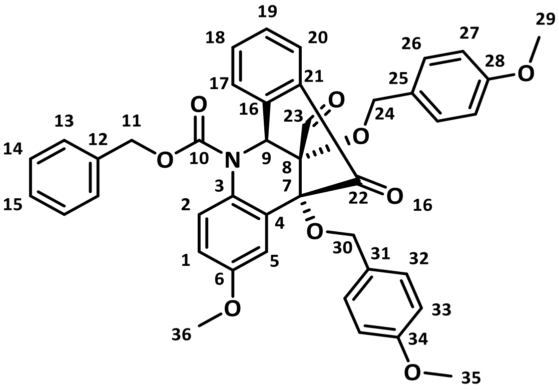

To a solution of benzyl (6*S*,12*R*,13*R*)-13-(hydroxymethyl)-2-methoxy-12,13-bis((4-methoxybenzyl)oxy)-11-oxo-11,12-dihydro-6,12-methanodibenzo[*b,f*]azocine-5(6*H*)-carboxylate (**26**, 881.9 mg, 1.258 mmol, 1.0 equiv.) in dichloromethane (25 mL) at 0 °C was added sodium bicarbonate (951.1 mg, 11.32 mmol, 9.0 equiv.) followed by Dess-Martin periodinane (1.60 g, 3.774 mmol, 3.0 equiv.). The reaction was warmed to RT and stirred for 1 h then cooled to 0 °C and quenched with sat. aq. sodium bicarbonate (25 mL). Sodium thiosulfate pentahydrate (5 g) was added and the biphasic mixture was stirred rigorously for 10 min. The layers were separated and the aqueous layer was extracted with dichloromethane (x 3). The combined organic fractions were dried over sodium sulfate, concentrated *in vacuo* and purified by flash column chromatography (30% to 50% ethyl acetate in pentane) to give the title compound as a white solid (788.7 mg, 1.128 mmol, 90%).

R_f: 0.2 (30% ethyl acetate in petroleum ether).

m.p.: 74.2 – 77.2 °C.

ν_{max}/cm^{-1} (thin film): 1734 (m), 1706 (m), 1515 (s), 1499 (m), 1302 (m), 1273 (m), 1253 (s), 1235 (m), 912 (m).

δ_H(600 MHz, $CDCl_3$): 9.96 (1H, s, C(23)**H**), 7.90 (1H, dd, *J* = 8.0, 1.5 Hz, Ar**H**), 7.68 – 7.61 (1H, m, Ar**H**), 7.58 (1H, td, *J* = 7.5, 1.5 Hz, Ar**H**), 7.43 – 7.38 (2H, m, 2 x Ar**H**), 7.38 – 7.30 (7H, m, 7 x Ar**H**), 7.07 – 7.02 (2H, m, 2 x Ar**H**), 6.96 (1H, d, *J* = 3.0 Hz, Ar**H**), 6.88 – 6.84 (2H, m, 2 x Ar**H**), 6.81 – 6.75 (2H, m, 2 x Ar**H**), 6.74 – 6.72 (1H, m, Ar**H**), 6.53 (1H, s, C(9)**H**), 5.33 (1H, d, *J* = 12.0 Hz, C(11)**H**H'), 5.22 (1H, br s, C(11)H**H'**), 5.04 (1H, d, *J* = 12.5 Hz, C(30)**H**H'), 4.75 (1H, d, *J* = 12.5 Hz, C(30)H**H'**), 4.61 (1H, br s, C(24)**H**H'), 4.45 (1H, br s, C(24)H**H'**), 3.81 and 3.76 (2 x 3H, s, C(29)$\mathbf{H_3}$ and C(35)$\mathbf{H_3}$), 3.55 (3H, s, C(36)$\mathbf{H_3}$).

δ_C(151 MHz, $CDCl_3$): 199.5 (**C**(23)), 190.0 (**C**(22)), 159.5 (**Ar**), 158.9 (**Ar**), 156.6 (**Ar**), 154.4 (**C**(10)*), 136.8 (**Ar**), 135.6 (**Ar**), 135.1 (**Ar**), 131.7 (**Ar**), 131.4 (**Ar**), 129.8 (**Ar**), 129.4 (**Ar**), 129.3 (**Ar**), 129.0 (**Ar**), 128.9 (**Ar**), 128.8 (**Ar**), 128.7 (**Ar**), 128.7 (**Ar**), 128.2 (**Ar**), 127.4 (**Ar**), 125.2 (**Ar**), 123.6 (**Ar**), 117.1 (**Ar**), 114.0 (**Ar**), 113.9 (**Ar**), 110.2 (**Ar**), 84.5 (**C**(7)), 80.0 (**C**(8)), 70.0 (**C**(30)), 68.9 (**C**(11)), 67.1 (**C**(24)), 55.5 and 55.5 (**C**(29) and **C**(35)), 55.4 (**C**(36)), 54.9 (**C**(9)).

m/z HRMS (ESI$^+$) found 722.2355; $C_{42}H_{37}NO_9Na^+$ (M+Na$^+$) requires 722.2361.

$[\alpha]_D^{25}$ = +177.2 (c = 0.61, $CHCl_3$)

* *assigned from $^1H/^{13}C$ HMBC spectrum*

Benzyl (6*S*,12*R*,13*S*)-13-ethynyl-2-methoxy-12,13-bis((4-methoxybenzyl)oxy)-11-oxo-11,12-dihydro-6,12-methanodibenzo[*b*,*f*]azocine-5(6*H*)-carboxylate: 28

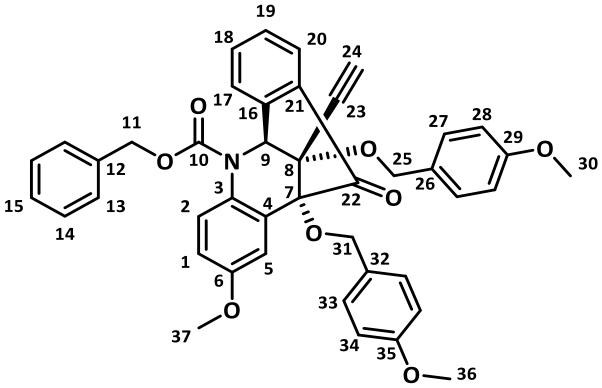

To a solution of benzyl (6*S*,12*R*,13*S*)-13-formyl-2-methoxy-12,13-bis((4-methoxybenzyl)oxy)-11-oxo-11,12-dihydro-6,12-methanodibenzo[*b*,*f*]azocine-5(6*H*)-carboxylate (**27**, 779.8 mg, 1.115 mmol, 1.0 equiv.) in methanol (13 mL) was added potassium carbonate (462.3 mg, 3.345 mmol, 3.0 equiv.). Dimethyl (1-diazo-2-oxopropyl)phosphonate (428.4 mg, 2.23 mmol, 2.0 equiv.) was dissolved in methanol (4 mL) and added dropwise to the reaction mixture at RT. The reaction was stirred at RT for 20 h then quenched with sat. aq. sodium bicarbonate (20 mL) and extracted with ethyl acetate (x 3). The combined organic fractions were dried over sodium sulfate, concentrated *in vacuo* and purified by flash column chromatography (2% to 5% diethyl ether in toluene) to give the title compound as a pale yellow solid (661.5 mg, 0.951 mmol, 85%).

R_f: 0.5 (10% diethyl ether in toluene)

ν_{max}/cm^{-1} (thin film): 1703 (m), 1613 (m), 1514 (s), 1498 (m), 1300 (m), 1269 (s), 1251 (s), 1234 (m), 1172 (m), 1019 (m)

δ_H(600 MHz, DMSO): 7.84 (1H, dd, *J* = 7.5, 1.5 Hz, Ar**H**), 7.74 (1H, td, *J* = 7.5, 1.5 Hz, Ar**H**), 7.68 (1H, dd, *J* = 7.5, 1.5 Hz, Ar**H**), 7.53 – 7.44 (4H, m, 4 x Ar**H**), 7.43 – 7.34 (5H, m, 5 x Ar**H**), 7.08 (2H, d, *J* = 8.5 Hz, 2 x Ar**H**), 6.98 – 6.92 (2H, m, 2 x Ar**H**), 6.84 – 6.80 (3H, m, 3 x Ar**H**), 6.78 (1H, dd, *J* = 9.0, 3.0 Hz, Ar**H**), 6.39 (1H, s, C(9)**H**), 5.35 (1H, d, *J* = 12.0 Hz, C(11)**H**H'), 5.29 (1H, d, *J*=12.0 Hz, C(11)H**H'**), 4.96 (1H, d, *J* = 11.5 Hz, C(31)**H**H'), 4.84 (1H, d, *J* = 10.0 Hz, C(25)**H**H'), 4.69 (1H, d, *J* = 11.5 Hz, C(31)H**H'**), 4.52 (1H, d, *J* = 10.0 Hz, C(25)H**H'**), 3.98 (1H, s, C(24)**H**), 3.77 and 3.70 (2 x 3H, s, C(30)$\mathbf{H_3}$ and C(36)$\mathbf{H_3}$), 3.56 (3H, s, C(37)$\mathbf{H_3}$)

δ_C(151 MHz, DMSO): 190.3 (**C**(22)), 158.9 (**Ar**), 158.6 (**Ar**), 155.7 (**Ar**), 153.8 (**C**(10)), 136.5 (**Ar**), 135.6 (**Ar**), 135.2 (**Ar**), 130.9 (**Ar**), 130.8 (**Ar**), 130.2 (**Ar**), 129.7 (**Ar**), 129.6 (**Ar**), 128.9 (**Ar**), 128.7 (**Ar**), 128.6 (**Ar**), 128.5 (**Ar**), 128.5 (**Ar**), 128.4 (**Ar**), 128.2 (**Ar**), 127.4 (**Ar**), 124.0 (**Ar**), 115.2 (**Ar**), 113.7 (**Ar**), 113.6 (**Ar**), 110.9 (**Ar**), 84.4 (**C**(24)), 84.3 (**C**(7)), 78.6 (**C**(23)), 72.0 (**C**(8)), 68.3 (**C**(31)), 68.2 (**C**(11)), 66.7 (**C**(25)), 59.2 (**C**(9)), 55.1 (**C**(37)), 55.1 and 55.0 (**C**(30) and **C**(36))

m.p.: 61.4 – 64.2 °C;

m/z HRMS (ESI$^+$) found 718.2394; $C_{43}H_{37}NO_8Na^+$ (M+Na$^+$) requires 718.2411

$[\alpha]_D^{25}$ = +210.9 (c = 0.53, $CHCl_3$)

Benzyl (6*S*,11*R*,12*S*,13*S*)-13-ethynyl-11-hydroxy-2-methoxy-12,13-bis((4-methoxybenzyl)oxy)-11,12-dihydro-6,12-methanodibenzo[*b*,*f*]azocine-5(6*H*)-carboxylate: 29

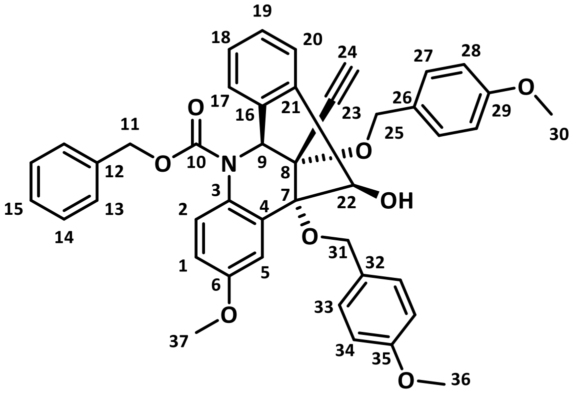

To a solution of benzyl (6*S*,12*R*,13*S*)-13-ethynyl-2-methoxy-12,13-bis((4-methoxybenzyl)oxy)-11-oxo-11,12-dihydro-6,12-methanodibenzo[*b*,*f*]azocine-5(6*H*)-carboxylate (**28**, 691.7 mg, 0.995 mmol, 1.0 equiv.) in tetrahydrofuran (16 mL) at 0 °C was added dropwise lithium borohydride (2 M in tetrahydrofuran, 1.49 mL, 2.985 mmol, 3.0 equiv.). The reaction was warmed to RT and stirred for 18 h then cooled to 0 °C and quenched with sat. aq. ammonium chloride (15 mL). The mixture was extracted with ethyl acetate (x 3) and the combined organic fractions were dried over sodium sulfate, concentrated *in vacuo* and purified by flash column chromatography (20% to 30% ethyl acetate in pentane) to give the title compound as a white solid (596.7 mg, 0.856 mmol, 86%).

R_f: 0.4 (30% ethyl acetate in petroleum ether).

m.p.: 85.7 – 87.1 °C.

$ν_{max}$/cm^{-1} (thin film): 1697 (m), 1691 (m), 1515 (m), 1498 (m), 1299 (m), 1252 (s), 1035 (m).

$δ_H$(600 MHz, DMSO): 7.52 – 7.43 (3H, m, 3 x Ar**H**), 7.43 – 7.30 (7H, m, 7 x Ar**H**), 7.29 – 7.24 (2H, m, 2 x Ar**H**), 7.08 (1H, d, *J* = 3.0 Hz, Ar**H**), 7.01 (2H, d, *J* = 8.5 Hz, 2 x Ar**H**), 6.94 – 6.89 (2H, m, 2 x Ar**H**), 6.81 – 6.76 (2H, m, 2 x Ar**H**), 6.71 – 6.61 (1H, d, *J* = 8.5 Hz, Ar**H**), 6.12 (1H, s, C(9)**H**), 6.03 (1H, d, *J* = 6.5 Hz, C(22)O**H**), 5.35 (1H, d, *J* = 6.5 Hz, C(22)**H**), 5.28 (1H, d, *J* = 12.0 Hz, C(11)**H**H'), 5.24 – 5.14 (1H, br s, C(11)H**H'**), 4.86 (1H, d, *J* = 12.0 Hz, C(31)**H**H'), 4.82 (1H, d, *J* = 10.0 Hz, C(25)**H**H'), 4.51 (1H, d, *J* = 12.0 Hz, C(31)H**H'**), 4.44 (1H, d, *J* = 10.0 Hz, C(25)H**H'**), 3.89 (1H, s, C(24)**H**), 3.75 and 3.68 (2 x 3H, s, C(30)$\mathbf{H_3}$ and C(36)$\mathbf{H_3}$), 3.57 (3H, s, C(37)$\mathbf{H_3}$).

$δ_C$(151 MHz, DMSO, 35 resonances out of 37 observed, 2 x Ar resonances not observed): 158.7 (**Ar**), 158.3 (**Ar**), 154.1 (**Ar**), 153.8 (**C**(10)*), 138.9 (**Ar**), 135.8 (**Ar**), 131.7 (**Ar**), 131.5 (**Ar**), 129.4 (**Ar**), 129.1 (**Ar**), 128.7 (**Ar**), 128.5 (**Ar**), 128.4 (**Ar**), 128.3 (**Ar**), 128.0 (**Ar**), 128.0 (**Ar**), 127.9 (**Ar**), 126.9 (**Ar**), 124.9 (**Ar**), 115.7 (**Ar**), 113.6 (**Ar**), 113.5 (**Ar**), 113.1 (**Ar**), 81.9 (**C**(24)), 80.6 (**C**(7)), 79.0 (**C**(23)), 74.9 (**C**(8)), 74.7 (**C**(22)), 68.0 (**C**(31)), 67.8 (**C**(11)), 66.0 (**C**(25)), 59.8 (**C**(9)), 55.0 and 55.0 (**C**(30) and **C**(36)), 54.8 (**C**(37)).

m/z HRMS (ESI$^+$) found 720.2559; $C_{43}H_{39}NO_8Na^+$ (M+Na$^+$) requires 720.2568.

$[\alpha]_D^{25}$ = +27.0 (c = 0.34, $CHCl_3$)

* *assigned from $^1H/^{13}C$ HMBC spectrum*

Benzyl (6*S*,11*R*,12*S*,13*S*)-13-ethynyl-2-methoxy-11,12,13-tris((4-methoxybenzyl)oxy)-11,12-dihydro-6,12-methanodibenzo[*b*,*f*]azocine-5(6*H*)-carboxylate: S6

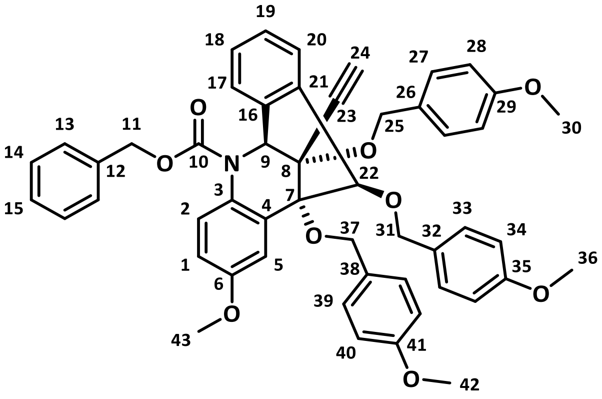

To a solution of benzyl (6*S*,11*R*,12*S*,13*S*)-13-ethynyl-11-hydroxy-2-methoxy-12,13-bis((4-methoxybenzyl)oxy)-11,12-dihydro-6,12-methanodibenzo[*b*,*f*]azocine-5(6*H*)-carboxylate (**29**, 546.2 mg, 0.783 mmol, 1.0 equiv.) in dimethyl formamide (15 mL) at 0 °C was added sodium hydride (60% in mineral oil, 37.6 mg, 0.940 mmol, 1.2 equiv.) as a single solid portion. The reaction mixture was warmed to RT and stirred for 15 min. 4-Methoxybenzyl bromide (0.25 mL, 1.566 mmol, 2.0 equiv.) was added and the reaction was stirred at RT for 14 h then cooled to 0 °C and quenched by dropwise addition of sat. aq. ammonium chloride (10 mL). The mixture was extracted with ethyl acetate (x 3) and the combined organic fractions were dried over sodium sulfate, concentrated *in vacuo* and purified by flash column chromatography (2% to 5% diethyl ether in toluene) to give the title compound as a white solid (556.4 mg, 0.681 mmol, 87%).

R_f: 0.6 (10% diethyl ether in toluene).

m.p.: 65.4 – 67.9 °C.

ν_{max}/cm^{-1} (thin film): 1698 (m), 1614 (m), 1515 (s), 1498 (m), 1394 (m), 1302 (m), 1252 (s), 1035 (m), 736 (m).

δ_H(600 MHz, DMSO): 7.52 – 7.35 (6H, m, 6 x Ar**H**), 7.33 (2H, d, *J* = 8.5 Hz, 2 x Ar**H**), 7.30 – 7.23 (4H, m, 4 x Ar**H**), 7.23 – 7.19 (2H, m, 2 x Ar**H**), 7.05 (2H, d, *J* = 8.0 Hz, 2 x Ar**H**), 6.97 – 6.95 (2H, m, 2 x Ar**H**), 6.94 (1H, d, *J* = 3.0 Hz, Ar**H**), 6.89 – 6.85 (2H, m, 2 x Ar**H**), 6.82 – 6.77 (2H, m, 2 x Ar**H**), 6.67 – 6.60 (1H, m, Ar**H**), 6.13 (1H, s, C(9)**H**), 5.28 (1H, d, *J* = 11.5 Hz, C(11)**H**H'), 5.27 (1H, s, C(22)**H**), 5.23 – 5.12 (1H, br s, C(11)H**H'**), 5.00 (1H, d, *J* = 10.0 Hz, C(31)**H**H'), 4.92 (1H, d, *J* = 10.0 Hz, C(31)H**H'**), 4.88 (1H, d, *J* = 11.0 Hz, C(37)**H**H'), 4.86 (1H, d, *J* = 9.5 Hz, C(25)**H**H'), 4.44 (1H, d, *J* = 11.5 Hz, C(37)H**H'**), 4.44 (1H, d, *J* = 9.5 Hz, C(25)H**H'**), 3.94 (1H, s, C(24)**H**), 3.77, 3.74 and 3.68 (3 x 3H, s, C(30)$\mathbf{H_3}$, C(36)$\mathbf{H_3}$ and C(42)$\mathbf{H_3}$), 3.36 (3H, s, C(43)$\mathbf{H_3}$).

δ_C(151 MHz, DMSO): 158.9 (**Ar**), 158.8 (**Ar**), 158.6 (**Ar**), 154.1 (**Ar**), 153.8 (**C**(10)*), 137.1 (**Ar**), 135.8 (**Ar**), 131.5 (**Ar**), 131.5 (**Ar**), 130.2 (**Ar**), 130.1 (**Ar**), 129.5 (**Ar**), 129.0 (**Ar**), 128.9 (**Ar**), 128.8 (**Ar**), 128.6 (**Ar**), 128.5(**Ar**), 128.5 (**Ar**), 128.3 (**Ar**), 128.3 (**Ar**), 128.2 (**Ar**), 126.9 (**Ar**), 125.3 (**Ar**), 124.3 (**Ar**), 114.5 (**Ar**), 114.4 (**Ar**), 113.7 (**Ar**), 113.7 (**Ar**), 113.6 (**Ar**), 83.3 (**C**(22)), 82.1 (**C**(24)), 82.0 (**C**(7)), 78.5 (**C**(23)), 75.6 (**C**(31)), 75.0 (**C**(8)), 68.7 (**C**(37)), 67.9 (**C**(11)), 66.0 (**C**(25)), 59.6 (**C**(9)), 55.1, 55.1 and 55.0 (**C**(30), **C**(36) and **C**(42)), 54.6 (**C**(43)).

m/z HRMS (ESI$^+$) found 840.3129; $C_{51}H_{47}NO_9Na^+$ (M+Na$^+$) requires 840.3143.

$[\alpha]_D^{25}$= +71.1 (c = 0.59. $CHCl_3$)

* *assigned from $^1H/^{13}C$ HMBC spectrum*

Benzyl (6*S*,11*R*,12*S*,13*S*)-2-methoxy-13-(3-methoxy-3-oxoprop-1-yn-1-yl)-11,12,13-tris((4-methoxybenzyl)oxy)-11,12-dihydro-6,12-methanodibenzo[*b*,*f*]azocine-5(6*H*)-carboxylate: 30

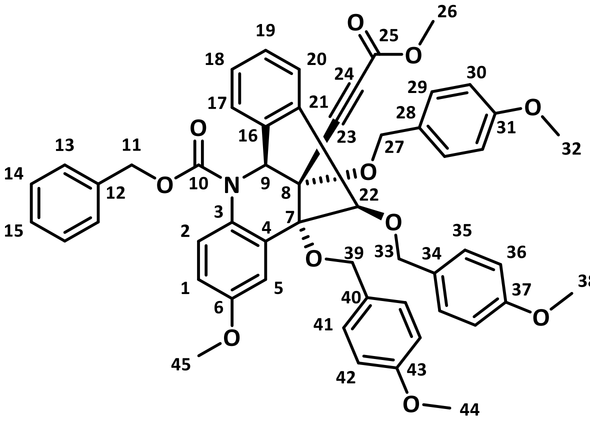

To a solution of benzyl (6*S*,11*R*,12*S*,13*S*)-13-ethynyl-2-methoxy-11,12,13-tris((4-methoxybenzyl)oxy)-11,12-dihydro-6,12-methanodibenzo[*b*,*f*]azocine-5(6*H*)-carboxylate (**S6**, 556.4 mg, 0.681 mmol, 1.0 equiv.) in tetrahydrofuran (14 mL) at – 78 °C was added dropwise lithium bis(trimethylsilyl)amide (1 M in toluene, 0.82 mL, 0.82 mmol, 1.2 equiv.) followed by hexamethylphosphoramide (0.59 mL, 3.405 mmol, 5.0 equiv.). The reaction mixture was stirred at –78 °C for 15 min. Methyl chloroformate (0.11 mL, 1.362 mmol, 2.0 equiv.) was added and the reaction was stirred at –78 °C for 2h then quenched at –78 °C with pre-cooled methanol (5 mL). The mixture was warmed to RT and partitioned between water (30 mL) and ethyl acetate (40 mL). The layers were separated and the aqueous fraction was extracted with ethyl acetate (x 2). The combined organic fractions were dried over sodium sulfate, concentrated *in vacuo* and purified by flash column chromatography (20% to 30% ethyl acetate in pentane)to give the title compound as a white solid (477.8 mg, 0.546 mmol, 80%).

R_f: 0.4 (30% ethyl acetate in petroleum ether).

m.p.: 68.6 – 71.0 °C.

ν_{max}/cm^{-1} (thin film): 1718 (m), 1614 (m), 1515 (s), 1498 (m), 1251 (s), 1123 (m), 1102 (m), 760 (m).

δ_H(600 MHz, DMSO): 7.52 – 7.42 (2H, m, 2 x Ar**H**), 7.42 – 7.25 (10H, m, 10 x Ar**H**), 7.25 – 7.20 (2H, m, 2 x Ar**H**), 7.08 – 7.01 (2H, m, 2 x Ar**H**), 6.99 – 6.94 (2H, m, 2 x Ar**H**), 6.93 (1H, d, *J* = 3.0 Hz, Ar**H**), 6.89 – 6.84 (2H, m, 2 x Ar**H**), 6.83 – 6.77 (2H, m, 2 x Ar**H**), 6.66 (1H, dd, *J* = 9.0, 3.0 Hz, Ar**H**), 6.25 (1H, s, C(9)**H**), 5.29 (1H, d, *J* = 12.0 Hz, C(11)**H**H'), 5.25 – 5.17 (1H, br s, C(11)H**H'**), 5.20 (1H, s, C(22)**H**), 4.99 (1H, d, *J* = 10.5 Hz, C(33)**H**H'), 4.95 (1H, d, *J* = 10.5 Hz, C(33)H**H'**), 4.87 (1H, d, *J* = 12.0 Hz, C(39)**H**H'), 4.81 (1H, d, *J* = 10.0 Hz, C(27)**H**H'), 4.50 (1H, d, *J* = 10.0 Hz, C(27)H**H'**), 4.47 (1H, d, *J* = 12.0 Hz, C(39)H**H'**), 3.77, 3.73 and 3.69 (3 x 3H, s, C(32)$\mathbf{H_3}$, C(38)$\mathbf{H_3}$ and C(44)$\mathbf{H_3}$), 3.74 (3H, s, C(26)$\mathbf{H_3}$), 3.38 (3H, s, C(45)$\mathbf{H_3}$).

δ_C(151 MHz, DMSO, 43 resonances out of 45 observed, 2 x Ar resonances not observed): 159.0 (**Ar**), 158.9 (**Ar**), 158.6 (**Ar**), 154.2 (**Ar**), 153.6 (**C**(10)), 152.3 (**C**(25)), 136.7 (**Ar**), 135.7 (**Ar**), 131.2 (**Ar**), 130.8 (**Ar**), 130.3 (**Ar**), 129.9 (**Ar**), 129.6 (**Ar**), 128.9 (**Ar**), 128.9 (**Ar**), 128.7 (**Ar**), 128.6 (**Ar**), 128.6 (**Ar**), 128.5 (**Ar**), 128.5 (**Ar**), 128.4 (**Ar**), 127.0 (**Ar**), 123.7 (**Ar**), 114.7 (**Ar**), 114.4 (**Ar**), 113.8 (**Ar**), 113.7 (**Ar**), 113.6 (**Ar**), 82.7 (**C**(22)), 82.4 (**C**(7)), 81.8 and 81.1 (**C**(23) and **C**(24)), 75.5 (**C**(33)), 75.5 (**C**(8)), 68.9 (**C**(39)), 68.0 (**C**(11)), 66.7 (**C**(27)), 58.8 (**C**(9)), 55.1, 55.1 and 55.0 (**C**(32), **C**(38) and **C**(44)), 54.6 (**C**(45)), 53.3 (**C**(26)).

m/z HRMS (ESI+) found 898.3181; $C_{53}H_{49}NO_{11}Na^+$ (M+Na+) requires 898.3198.

$[\alpha]_D^{25}$= +79.4 (c = 0.6, $CHCl_3$)

Benzyl (6*S*,11*R*,12*S*,13*S*)-2-methoxy-13-((*E*)-4-methoxy-4-oxobut-2-en-2-yl)-11,12,13-tris((4-methoxybenzyl)oxy)-11,12-dihydro-6,12-methanodibenzo[*b*,*f*]azocine-5(6*H*)-carboxylate (31)

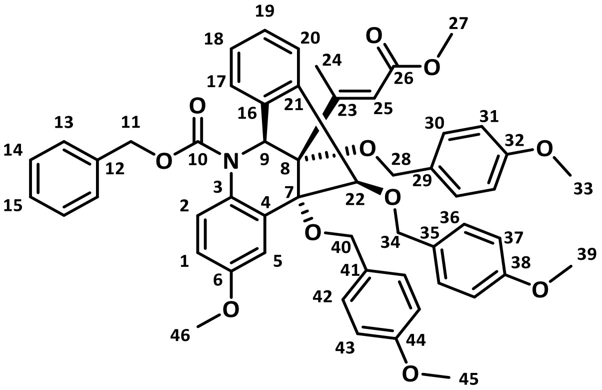

Copper(I) cyanide (90.0 mg, 1.005 mmol, 4.0 equiv.) was suspended in freeze-pump-thaw degassed diethyl ether (4.0 mL) and cooled to 0°C. Methyllithium solution (1.7M in diethyl ether, 1.17 mL, 1.983 mmol, 7.9 equiv.) was added dropwise, initially giving a bright yellow solution whose colour dissipated over the course of the addition to give a light grey solution. A solution of benzyl (6*R*,11*S*,12*R*,13*R*)-11,12,13-tris(benzyloxy)-2-methoxy-13-(3-methoxy-3-oxoprop-1-yn-1-yl)-11,12-dihydro-6,12-methanodibenzo[*b*,*f*]azocine-5(6*H*)-carboxylate (**30**, 61.5 mg, 0.078 mmol, 1.0 equiv.) in diethyl ether (3.0 mL) was added dropwise at 0 °C, giving a pale yellow solution. The reaction was stirred at 0 °C for 3 h then quenched by dropwise addition of methanol (3.0 mL). Ammonium hydroxide (10% wt. in sat. aq. ammonium chloride, 10 mL) was added and the resultant biphasic mixture was stirred vigorously at RT for 5 min then extracted with ethyl acetate (x 3). The combined organic fractions were dried over sodium sulfate, concentrated *in vacuo* and purified by flash column chromatography (2% to 10% diethyl ether in toluene) to give the title compound as a colourless oil (105.5 mg, 0.118 mmol, 47%).

R_f: 0.6 (10% diethyl ether in toluene).

m.p.: 69.4 – 73.2 °C.

ν_{max}/cm^{-1} (thin film): 1719 (m), 1687 (m), 1515 (s), 1303 (m), 1252 (s), 1213 (m), 1121 (m), 1034 (m), 736 (m).

δ_H(600 MHz, DMSO): 7.53 – 7.31 (7H, m, 7 x Ar**H**), 7.32 – 7.25 (3H, m, 3 x Ar**H**), 7.25 – 7.19 (2H, m, 2 x Ar**H**), 7.13 – 7.04 (4H, m, 4 x Ar**H**), 7.00 – 6.96 (2H, m, 2 x Ar**H**), 6.93 (1H, d, *J* = 3.0 Hz, Ar**H**), 6.83 – 6.76 (4H, m, 4 x Ar**H**), 6.62 (1H, d, *J* = 8.0 Hz, Ar**H**), 6.29 (1H, s, C(9)**H**), 5.56 (1H, s, C(25)**H**), 5.29 (1H, d, *J* = 12.0 Hz, C(11)**H**H'), 5.27 – 5.12 (1H, br s, C(11)H**H'**), 4.94 (1H, d, *J* = 11.5 Hz, C(40)**H**H'), 4.90 (1H, d, *J* = 10.0 Hz, C(34)**H**H'), 4.81 (1H, d, *J* = 10.0 Hz, C(34)H**H'**), 4.48 (1H, s, C(22)**H**), 4.36 (1H, d, *J* = 11.5 Hz, C(40)H**H'**), 4.36 (1H, d, *J* = 10.5 Hz, C(28)**H**H'), 4.18 (1H, d, *J* = 10.5 Hz, C(28)H**H'**), 3.77, 3.72 and 3.68 (3 x 3H, s, C(33)**H₃**, C(39)**H₃**, C(45)**H₃**), 3.57 (3H, s, C(27)**H₃**), 3.29 (3H, s, C(46)**H₃**), 2.67 (3H, s, C(24)**H₃**).

δ_C(151 MHz, DMSO, 45 resonances out of 46 observed, **C**(23) not observed): 165.2 (**C**(26)), 158.9 (**Ar**), 158.8 (**Ar**), 158.7 (**Ar**), 153.9 (**Ar**), 153.5 (**C**(10)), 137.3 (**Ar**), 135.8 (**Ar**), 133.1 (**Ar**), 131.1 (**Ar**), 130.2 (**Ar**), 130.1 (**Ar**), 129.5 (**Ar**), 129.2 (**Ar**), 129.2 (**Ar**), 129.0 (**Ar**), 128.8 (**Ar**), 128.8 (**Ar**), 128.5 (**Ar**), 128.5 (**Ar**), 128.4 (**Ar**), 128.3 (**Ar**), 127.4 (**Ar**), 126.8 (**Ar**), 125.4 (**Ar**), 121.0 (**C**(25)), 114.8 (**Ar**), 114.5 (**Ar**), 113.8 (**Ar**), 113.7 (**Ar**), 113.5 (**Ar**), 84.2 (**C**(7)), 81.7 (**C**(22)), 80.2 (**C**(8)), 75.6 (**C**(34)), 68.4 (**C**(40)), 67.8 (**C**(11)), 64.4 (**C**(28)), 57.2 (**C**(9)), 55.1, 55.1 and 55.0 (**C**(33), **C**(39) and **C**(45)), 54.4 (**C**(46)), 51.2 (**C**(27)), 16.2 (**C**(24)).

m/z HRMS (ESI$^+$) found 914.3507; $C_{54}H_{53}NO_{11}Na^+$ (M+Na$^+$) requires 914.3511.

$[\alpha]_D^{25}$= +133.4 (c = 0.35, $CHCl_3$)

Methyl (*E*)-3-((6*S*,11*R*,12*S*,13*S*)-2-methoxy-11,12,13-tris((4-methoxybenzyl)oxy)-5,6,11,12-tetrahydro-6,12-methanodibenzo[*b,f*]azocin-13-yl)but-2-enoate: S7

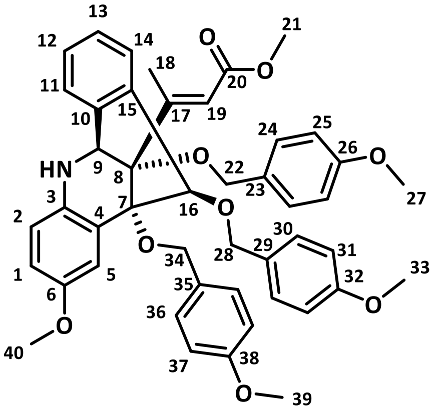

Prepared according to the procedure of Epplin.[134]

To a solution of benzyl (6*S*,11*R*,12*S*,13*S*)-2-methoxy-13-((*E*)-4-methoxy-4-oxobut-2-en-2-yl)-11,12,13-tris((4-methoxybenzyl)oxy)-11,12-dihydro-6,12-methanodibenzo[*b,f*]azocine-5(6*H*)-carboxylate (**31**, 109.9 mg, 0.123 mmol, 1.0 equiv.) in methanol (13 mL) and ethyl acetate (1 mL) was added palladium on carbon (10% wt. Pd, 13.1 mg, 0.012 mmol, 0.1 equiv.). The resulting suspension was degassed with nitrogen for five min. A balloon of hydrogen was added and bubbled with an escape needle for 5 min before the escape needle was removed and the reaction mixture was stirred under positive hydrogen pressure for 3 h. The hydrogen balloon was removed and the suspension was degassed with nitrogen for five min then filtered through Celite™, washing with ethyl acetate. The filtrate was dried over sodium sulfate, concentrated *in vacuo* and purified by flash column chromatography (20% to 30% ethyl acetate in pentane) to give the title compound as a pale yellow solid (82.7 mg, 0.109 mmol, 89%).

R_f: 0.3 (30% ethyl acetate in petroleum ether).

m.p.: 89.4 – 91.5 °C.

ν_{max}/cm^{-1} (thin film): 1721 (m), 1614 (m), 1514 (s), 1302 (m), 1252 (s), 1211 (m), 1174 (m), 1038 (s), 764 (m).

δ_H(600 MHz, DMSO): 7.46 (1H, d, *J* = 7.5 Hz, Ar**H**), 7.31 – 7.20 (6H, m, 6 x Ar**H**), 7.17 – 7.13 (1H, m, Ar**H**), 7.12 – 7.08 (2H, m, 2 x Ar**H**), 6.97 – 6.94 (2H, m, 2 x Ar**H**), 6.88 – 6.84 (2H, m, 2 x Ar**H**), 6.83 – 6.80 (2H, m, 2 x Ar**H**), 6.79 (1H, d, *J* = 3.0 Hz, Ar**H**), 6.50 (1H, dd, *J* = 8.5, 3.0 Hz, Ar**H**), 6.42 (1H, d, *J* = 8.5 Hz, Ar**H**), 6.05 (1H, d, *J* = 4.0 Hz, N**H**), 5.61 (1H, s, C(19)**H**), 4.93 (1H, d, *J* = 10.0 Hz, C(28)**H**H'), 4.92 (1H, d, *J* = 4.0 Hz, C(9)**H**), 4.88 (1H, d, *J* = 12.0 Hz, C(34)**H**H'), 4.78 (1H, d, *J* = 10.0 Hz, C(28)H**H'**), 4.74 (1H, d, *J* = 10.5 Hz, C(22)**H**H'), 4.51 (1H, s, C(16)**H**), 4.40 (1H, d, *J* = 12.0 Hz, C(34)H**H'**), 4.13 (1H, d, *J* = 10.5 Hz, C(22)H**H'**), 3.76, 3.72 and 3.70 (3 x 3H, s, C(27)$\mathbf{H_3}$, C(33)$\mathbf{H_3}$, C(39)$\mathbf{H_3}$), 3.55 (3H, s, C(21)$\mathbf{H_3}$), 3.29 (3H, s, C(40)$\mathbf{H_3}$), 2.58 (3H, s, C(18)$\mathbf{H_3}$).

δ_C(151 MHz, DMSO, 39 resonances out of 40 observed, 1 x Ar resonance not observed): 165.7 (**C**(20)), 158.8 (**Ar**), 158.6 (**Ar**), 158.5 (**Ar**), 155.4 (**C**(17)), 149.8 (**Ar**), 138.9 (**Ar**), 137.1 (**Ar**), 136.0 (**Ar**), 131.6 (**Ar**), 130.5 (**Ar**), 130.4 (**Ar**), 130.0 (**Ar**), 129.0 (**Ar**), 128.8 (**Ar**), 128.3 (**Ar**), 127.5 (**Ar**), 127.0 (**Ar**), 126.4 (**Ar**), 120.7 (**C**(19)), 119.0 (**Ar**), 115.5 (**Ar**), 115.1 (**Ar**), 113.8 (**Ar**), 113.6 (**Ar**), 113.5 (**Ar**), 84.3 (**C**(7)), 83.5 (**C**(16)), 80.0 (**C**(28)), 75.4 (**C**(34)), 67.9 (**C**(22)), 55.1, 55.0 and 55.0 (**C**(27), **C**(33) and **C**(39)), 54.9 (**C**(8)), 54.8 (**C**(9)), 54.6 (**C**(40)), 51.0 (**C**(21)), 15.9 (**C**(18)).

m/z HRMS (ESI$^+$) found 780.3130; $C_{46}H_{47}NO_9Na^+$ (M+Na$^+$) requires 780.3143.

$[\alpha]_D^{25}$ = +9.5 (c = 0.52, $CHCl_3$)

Methyl (E)-3-((6*S*,11*R*,12*S*,13*S*)-11,12,13-tris((4-methoxybenzyl)oxy)-2-oxo-2,6,11,12-tetrahydro-6,12-methanodibenzo[*b*,*f*]azocin-13-yl)but-2-enoate: 32

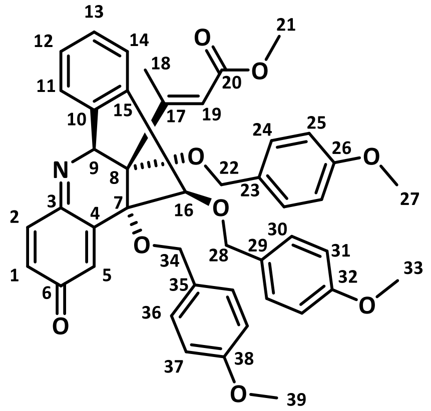

Prepared according to the procedure of Lebouef.[12]

To a solution of methyl (*E*)-3-((6*S*,11*R*,12*S*,13*S*)-2-methoxy-11,12,13-tris((4-methoxybenzyl)oxy)-5,6,11,12-tetrahydro-6,12-methanodibenzo[*b*,*f*]azocin-13-yl)but-2-enoate (**S7**, 82.7 mg, 0.109 mmol, 1.0 equiv.) in acetonitrile (4.0 mL) and water (1.3 mL) was added cerium (IV) ammonium nitrate (131.7 mg, 0.240 mmol, 2.2 equiv.) as a single solid portion. The reaction mixture was stirred for 15 min then diluted with ethyl acetate (15 mL) and water (15 mL). The layers were separated and the aqueous layer was washed with ethyl acetate (x 2). The combined organic fractions were dried over sodium sulfate, concentrated *in vacuo* and purified by flash column chromatography (30% ethyl acetate in pentane) to give the title compound as pale yellow solid (68.2 mg, 0.092 mmol, 84%).

R_f: 0.4 (20% ethyl acetate in petroleum ether).

m.p.: 85.7 – 88.7 °C.

ν_{max}/cm^{-1} (thin film): 1724 (m), 1647 (m), 1614 (m), 1516 (s), 1303 (m), 1254 (s), 1217 (m), 1176 (m), 1061 (m), 1036 (s), 821 (m), 760 (s).

δ_H(600 MHz, DMSO): 7.52 (1H, dd, *J* = 7.5, 1.5 Hz, Ar**H**), 7.33 (1H, d, *J* = 7.5 Hz, Ar**H**), 7.30 (1H, t, *J* = 7.5 Hz, Ar**H**), 7.27 (1H, td, *J* = 7.5, 1.5 Hz, Ar**H**), 7.26 – 7.23 (2H, m, 2 x Ar**H**), 7.22 (1H, d, *J* = 10.0 Hz, C(2)**H**), 7.21 – 7.18 (2H, m, 2 x Ar**H**), 7.11 – 7.07 (2H, m, 2 x Ar**H**), 6.98 – 6.94 (2H, m, 2 x Ar**H**), 6.91 – 6.88 (2H, m, 2 x Ar**H**), 6.87 – 6.82 (2H, m, 2 x Ar**H**), 6.53 (1H, dd, *J* = 10.0, 2.0 Hz, C(1)**H**), 6.44 (1H, d, *J* = 2.0 Hz, C(5)**H**), 6.21 (1H, s, C(9)**H**), 5.79 (1H, q, *J* = 1.5 Hz, C(19)**H**), 4.89 (1H, d, *J* = 11.5 Hz, C(34)**H**H'), 4.83 (1H, d, *J* = 10.0 Hz, C(28)**H**H'), 4.77 (1H, d, *J* = 10.0 Hz, C(28)H**H'**), 4.52 (1H, d, *J* = 11.5 Hz, C(34)H**H'**), 4.49 (1H, s, C(16)**H**), 4.31 (1H, d, *J* = 10.5 Hz, C(22)**H**H'), 4.20 (1H, d, *J* = 10.5 Hz, C(22)H**H'**), 3.76, 3.73 and 3.71 (3 x 3H, s, C(27)$\mathbf{H_3}$, C(33)$\mathbf{H_3}$, C(39)$\mathbf{H_3}$), 3.57 (3H, s, C(21)$\mathbf{H_3}$), 2.52 (3H, d, *J* = 1.5 Hz, C(18)$\mathbf{H_3}$).

δ_C(151 MHz, DMSO): 186.5 **C**(6), 165.5 **C**(20), 160.2 **C**(3), 158.9 (**Ar**), 158.8 (**Ar**), 158.8 (**Ar**), 153.0 **C**(17), 140.8 (**C**(2)), 136.3 **C**(4), 134.5 (**Ar**), 133.9 (**Ar**), 132.5 (**C**(5)), 131.7 (**C**(1)), 130.3 (**Ar**), 130.1 (**Ar**), 129.8 (**Ar**), 129.4 (**Ar**), 129.2 (**Ar**), 128.9 (**Ar**), 128.5 (**Ar**), 128.4 (**Ar**), 128.2 (**Ar**), 128.1 (**Ar**), 121.9 (**C**(19)), 113.9 (**Ar**), 113.8 (**Ar**), 113.6 (**Ar**), 83.8 (**C**(7)), 83.6 (**C**(8)), 79.1 (**C**(16)), 75.6 (**C**(28)), 68.2 (**C**(34)), 64.3 (**C**(22)), 64.0 (**C**(9)), 55.1, 55.0 and 55.0 (**C**(27), **C**(33) and **C**(39)), 51.2 (**C**(21)), 15.5 (**C**(18)).

m/z HRMS (ESI$^+$) found 764.2824; $C_{45}H_{43}NO_9Na^+$ (M+Na$^+$) requires 764.2830.

$[\alpha]_D^{25}$ = +110.1 (c = 0.6, $CHCl_3$)

Methyl (*E*)-3-((8*S*,9*R*,14*S*,17*S*)-6-hydrox-8,9,17-tris((4-methoxybenzyl)oxy)-5,16-dioxo-5,8,9,14,15,16-hexahydro-8,14-methanoanthra[1,2-b]benzo[*f*]azocin-17-yl)but-2-enoate: 33

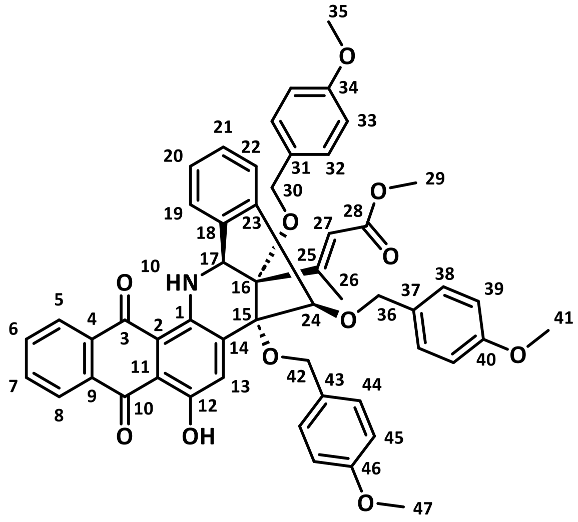

Prepared according to the procedure of Nicolaou.[13]

To a solution of 3-cyanophthalide (16.9 mg, 0.110 mmol, 1.3 equiv.) in tetrahydrofuran (1.7 mL) at –78 °C was added dropwise lithium bis(trimethylsilyl)amide (1 M in toluene, 0.11 mL, 0.110 mmol, 1.3 equiv.). The resultant bright yellow solution was stirred at –78 °C for 15 min. A solution of methyl (*E*)-3-((6*S*,11*R*,12*S*,13*S*)-11,12,13-tris((4-methoxybenzyl)oxy)-2-oxo-2,6,11,12-tetrahydro-6,12-methanodibenzo[b,f]azocin-13-yl)but-2-enoate (**32**, 60.8 mg, 0.082 mmol, 1.0 equiv.) in tetrahydrofuran (1.7 mL) was added dropwise. The cool bath was removed and the reaction mixture was allowed to warm to RT, during which time the bright yellow solution turned first dark green then dark purple. The reaction was stirred at RT for 15 min then quenched by dropwise addition of sat. aq. ammonium chloride solution (5 mL) and extracted with ethyl acetate (x 3). The combined organic fractions were dried over sodium sulfate, concentrated *in vacuo* and purified by flash column chromatography (40% ethyl acetate in pentane) to give the title compound as dark purple solid (61.9 mg, 0.071 mmol, 86%).

R_f: 0.5 (10% ethyl acetate in toluene).

m.p.: 115.1 – 118.9 °C.

ν_{max}/cm^{-1} (thin film): 1795 (m), 1732 (m), 1616 (m), 1588 (m), 1515 (s), 1497 (m), 1303 (m), 1251 (s), 1211 (m), 1103 (m), 820 (m), 732 (m).

δ_H(600 MHz, DMSO): 13.21 (1H, s, C(12)O**H**), 10.71 (1H, d, *J* = 5.5 Hz (N**H**)), 8.25 (1H, dd, *J* = 8.0, 1.5 Hz, C(5)**H**), 8.20 (1H, dd, *J* = 8.0, 1.5 Hz, C(8)**H**), 7.92 (1H, td, *J* = 8.0, 1.5 Hz, C(6)**H**), 7.85 (1H, td, *J* = 8.0, 1.5 Hz, C(7)**H**), 7.61 (1H, d, *J* = 7.5 Hz, Ar**H**), 7.37 (1H, s, C(13)**H**), 7.31 (1H, t, *J* = 7.5 Hz, Ar**H**), 7.28 (2H, m, 2 x Ar**H**), 7.26 – 7.23 (1H, m, Ar**H**), 7.19 (1H, d, *J* = 7.5 Hz, Ar**H**), 7.18 (2H, m, 2 x Ar**H**), 7.15 – 7.12 (2H, m, 2 x Ar**H**), 7.01 – 6.97 (2H, m, 2 x Ar**H**), 6.88 – 6.84 (2H, m, 2 x Ar**H**), 6.81 – 6.77 (2H, m, 2 x Ar**H**), 5.67 (1H, q, *J* = 1.5 Hz, C(27)**H**), 5.62 (1H, d, *J* = 5.5 Hz, C(17)**H**), 4.94 (1H, d, *J* = 11.5 Hz, C(42)**H**H'), 4.92 (1H, d, *J* = 10.0 Hz, C(36)**H**H'), 4.82 (1H, d, *J* = 10.0 Hz, C(36)H**H'**), 4.73 (1H, d, *J* = 10.0 Hz, C(30)**H**H'), 4.62 (1H, s, C(24)**H**), 4.32 (1H, d, *J* = 11.5 Hz, C(42)H**H'**), 4.19 (1H, d, *J* = 10.2 Hz, C(30)H**H'**), 3.78, 3.74 and 3.63 (3 x 3H, s, C(35)$\mathbf{H_3}$, C(41)$\mathbf{H_3}$, C(47)$\mathbf{H_3}$), 3.57 (3H, s, C(29)$\mathbf{H_3}$), 2.62 (3H, d, *J* = 1.5 Hz, C(26)$\mathbf{H_3}$).

δ_C(151 MHz, DMSO): 186.6 (**C**(10)), 180.9 (**C**(3)), 165.4 (**C**(28)), 158.9 (**Ar**), 158.9 (**Ar**), 158.7 (**Ar**), 154.6 (**C**(11)), 153.5 (**C**(25)), 144.2 (**C**(14)), 136.1(**Ar**), 134.7 (**C**(6)), 134.7 (**Ar**), 134.5 (**C**(9)), 134.2 (**C**(1)), 133.2 (**C**(7)), 132.1 (**C**(4)), 130.5 (**C**(13)), 130.4 (**Ar**), 130.1 (**Ar**), 129.9 (**Ar**), 129.6 (**Ar**), 129.4 (**Ar**), 129.1 (**Ar**), 129.1 (**Ar**), 127.8 (**Ar**), 127.4 (**Ar**), 127.0 (**Ar**), 126.4 (**C**(5)), 126.0 (**C**(8)), 121.5 (**C**(27)), 114.0 (**Ar**), 113.7 (**Ar**), 113.7 (**Ar**), 113.0 (**C**(12)), 107.8 (**C**(2)), 84.4 (**C**(15)), 82.0 (**C**(24)), 80.1 (**C**(16)), 75.6 (**C**(36)), 68.5 (**C**(42)), 63.4 (**C**(30)), 55.1, 55.0 and 54.9 (**C**(35), **C**(41) and **C**(47)), 53.3 (**C**(17)), 51.2 (**C**(29)), 15.7 (**C**(26)).

m/z HRMS (ESI$^+$) found 874.3235; $C_{53}H_{48}NO_{11}^+$ (M+H$^+$) requires 874.3222.

Anthraquinone **32** was so highly colored (purple) that it was not possible to measure an optical rotation.

Methyl (*E*)-3-((8*S*,9*R*,14*S*,17*S*)-6,8,9,17-tetrahydroxy-5,16-dioxo-5,8,9,14,15,16-hexahydro-8,14-methanoanthra[1,2-b]benzo[*f*]azocin-17-yl)but-2-enoate: Sealutomicin C (3)

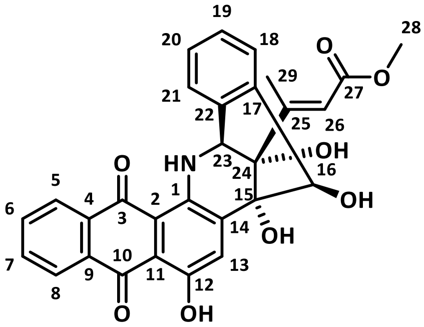

Prepared according to the procedure of Carreira.[136]

To a solution of methyl (*E*)-3-((8*S*,9*R*,14*S*,17*S*)-6-hydroxy-8,9,17-tris((4-methoxybenzyl)oxy)-5,16-dioxo-5,8,9,14,15,16-hexahydro-8,14-methanoanthra[1,2-b]benzo[*f*]azocin-17-yl)but-2-enoate (**33**, 20.0 mg, 0.023 mmol, 1.0 equiv.) in dichloromethane (2.5 mL) and water (0.25 mL) at RT was added 2,3-dichloro-5,6-dicyano-1,4-benzoquinone (104.0 mg, 0.46 mmol, 20.0 equiv.). The reaction mixture was stirred vigorously at RT for 16 h then cooled to 0 °C and quenched by dropwise addition of sat. aq. sodium bicarbonate (4 mL). The mixture was extracted with ethyl acetate (x 3) and the combined organic fractions were dried over sodium sulfate, concentrated *in vacuo* and purified by flash column chromatography (30% to 50% ethyl acetate in pentane) to give the title compound as a dark purple solid (8.3 mg, 0.016 mmol, 70%).

R_f: 0.5 (50% ethyl acetate in petroleum ether).

m.p.: 152.3 – 155 °C.

ν_{max}/cm^{-1} (thin film): 3445 (br, m), 1716 (m), 1617 (m), 1586 (s), 1267 (s), 1243 (s), 1216 (m), 730 (m).

δ_H(600 MHz, DMSO): 13.58 (1H, s, C(12)O**H**), 10.83 (1H, d, *J* = 5.5 Hz, N**H**), 8.22 (1H, dd, *J* = 7.5, 1.0 Hz, C(8)**H**), 8.21 (1H, dd, *J* = 7.5, 1.0 Hz, C(5)**H**), 7.88 (1H, td, *J* = 7.5, 1.0 Hz, C(7)**H**), 7.81 (1H, td, *J* = 7.5, 1.0 Hz, C(6)**H**), 7.63 (1H, s, C(13)**H**), 7.63 (1H, d, *J* = 7.5 Hz, C(21)**H**), 7.35 (1H, d, *J* = 7.5 Hz, C(18)**H**), 7.27 (1H, t, *J* = 7.5 Hz, C(20)**H**), 7.19 (1H, t, *J* = 7.5 Hz, C(19)**H**), 6.01 (1H, d, *J* = 6.0 Hz, C(16)O**H**), 5.95 (1H, s, C(15)O**H**), 5.95 (1H, s, C(24)O**H**), 5.79 (1H, q, *J* = 1.0 Hz, C(26)**H**), 4.95 (1H, d, *J* = 5.5 Hz, C(23)**H**), 4.59 (1H, d, *J* = 6.0 Hz, C(16)**H**), 3.52 (3H, s, C(28)$\mathbf{H_3}$), 2.34 (3H, d, *J* = 1.0 Hz, C(29)$\mathbf{H_3}$).

δ_C(151 MHz, DMSO): 186.1 (**C**(3)), 180.1 (**C**(10)), 166.1 (**C**(27)), 158.1 (**C**(25)), 155.9 (**C**(12)), 143.4 (**C**(1)), 141.7 (**C**(14)), 137.5 (**C**(22)), 135.9 (**C**(17)), 134.8 (**C**(9)), 134.5 (**C**(7)), 132.8 (**C**(6)), 132.0 (**C**(4)), 130.4 (**C**(13)), 128.4 (**C**(20)), 127.4 (**C**(19)), 127.3 (**C**(21)), 127.2 (**C**(18)), 126.2 (**C**(8)), 125.9 (**C**(5)), 117.5 (**C**(26)), 111.9 (**C**(11)), 106.1 (**C**(2)), 77.9 (**C**(15)), 74.2 (**C**(16)), 72.4 (**C**(24)), 57.7 (**C**(23)), 50.9 (**C**(28)), 16.9 (**C**(29)).

δ_H(600 MHz, Acetone): 8.26 (1H, dd, *J* = 8.0, 1.0 Hz, C(5)**H**), 8.24 (1H, dd, *J* = 8.0, 1.0 Hz, C(8)**H**), 7.85 (1H, td, *J* = 8.0, 1.0 Hz, C(6)**H**), 7.80 (1H, td, *J* = 8.0, 1.0 Hz, C(7)**H**), 7.79 (1H, s, C(13)**H**), 7.66 (1H, dd, *J* = 7.5, 1.5 Hz, C(18)**H**), 7.51 (1H, dt, *J* = 7.5, 1.5 Hz, C(21)**H**), 7.30 (1H, tt, *J* = 7.5, 1.5 Hz, C(19)**H**), 7.22 (1H, td, *J* = 7.5, 1.5 Hz, C(20)**H**), 5.93 (1H, q, *J* = 1.0 Hz, C(26)**H**), 5.07 (1H, s, C(23)**H**), 4.89 (1H, s, C(16)**H**), 3.55 (3H, s, C(28)$\mathbf{H_3}$), 2.46 (3H, d, *J* = 1.0 Hz, C(29)$\mathbf{H_3}$)

δ_C(151 MHz, Acetone): 186.9 (**C**(10)), 181.3 (**C**(3)), 166.2 (**C**(27)), 157.1 (**C**(25)), 156.0 (**C**(12)), 143.0 (**C**(1)), 140.5 (**C**(14)), 137.6 (**C**(22)), 135.9 (**C**(17)), 135.2 (**C**(4)), 134.1 (**C**(6)), 132.7 (**C**(9)), 132.6 (**C**(7)), 130.3 (**C**(13)), 128.7 (**C**(19)), 127.7 (**C**(20)), 127.3 (**C**(18)), 127.3 (**C**(21)), 126.4 (**C**(5)), 125.9 (**C**(8)), 118.4 (**C**(26)), 112.7 (**C**(11)), 107.7 (**C**(2)), 78.5 (**C**(15)), 74.9 (**C**(16)), 72.6 (**C**(24)), 58.5 (**C**(23)), 50.2 (**C**(28)), 16.3 (**C**(29))

m/z HRMS (ESI^+) found 514.1490; $C_{29}H_{24}NO_8^+$ ($M+H^+$) requires 514.1496.

Sealutomicin C **3** was so highly colored (purple) that it was not possible to measure an optical rotation – the CD spectrum is on p S44.

Data in accordance with literature.[14]

Circular Dichroism

To a cuvette with 10 mm path length and blackened sides was added a solution of synthetic **3** in methanol (0.025 µmol/mL). The sample was then measured on an Applied Photophysics Chirascan^{TM} Spectrophotometer from 210 to 700 nm with a step size of 0.5 nm and a time per point of 1 s. Three successive spectra were recorded, averaged, and smoothed using a Savitzky-Golay filter. *We are grateful to Dennis Hartmann (University of Oxford) for acquiring this CD spectrum.*

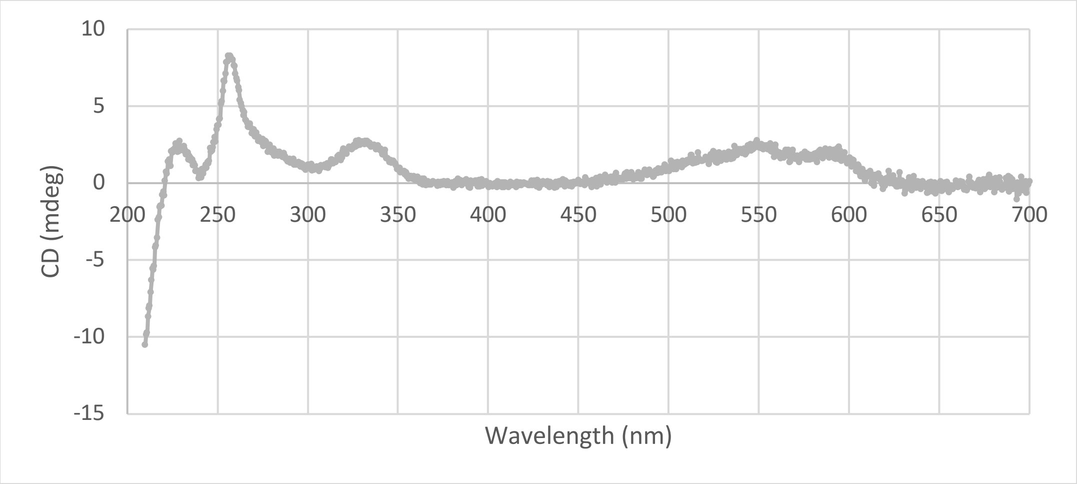

Data in accordance with literature[14]

NMR Data Comparison for Synthetic and Natural Sealutomicin C

The ^{13}C NMR data for the sample of synthetic sealutomicin C in deuterated DMSO matched those reported for natural sealutomicin by Igarashi, with all resonances consistent to within 0.1 ppm (Table 4.1).[14]

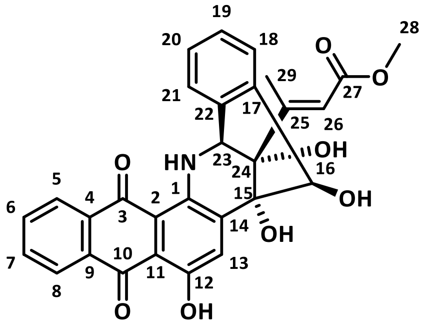

Position	δ_C[a] Natural[14]	δ_C[b] Synthetic	Δδ(Natural – Synthetic)
C(1)	143.4 (s)	143.4 (s)	0.0
C(2)	106.1 (s)	106.1 (s)	0.0
C(3)	186.1 (s)	186.1 (s)	0.0
C(4)	132.0 (s)	132.0 (s)	0.0
C(5)	125.9 (d)	125.9 (s)	0.0
C(6)	132.9 (d)	132.8 (s)	+0.1
C(7)	134.6 (d)	134.5 (s)	+0.1
C(8)	126.3 (d)	126.2 (s)	+0.1
C(9)	134.8 (s)	134.8 (s)	0.0
C(10)	180.1 (s)	180.1 (s)	0.0
C(11)	111.9 (s)	111.9 (s)	0.0
C(12)	155.9 (s)	155.9 (s)	0.0
C(13)	130.4 (d)	130.4 (s)	0.0
C(14)	141.7 (s)	141.7 (s)	0.0
C(15)	77.9 (s)	77.9 (s)	0.0
C(16)	74.2 (d)	74.2 (s)	0.0
C(17)	135.9 (s)	135.9 (s)	0.0
C(18)	127.2 (d)	127.2 (s)	0.0
C(19)	127.5 (d)	127.5 (s)	0.0
C(20)	128.4 (d)	128.4 (s)	0.0
C(21)	127.3 (d)	127.3 (s)	0.0
C(22)	137.5 (s)	137.5 (s)	0.0
C(23)	57.8 (d)	57.7 (s)	+0.1
C(24)	72.4 (s)	72.4 (s)	0.0
C(25)	158.2 (s)	158.1 (s)	-0.1
C(26)	117.4 (d)	117.5(s)	0.0
C(27)	166.1 (s)	166.1 (s)	0.0
C(28)	50.9 (q)	50.9 (s)	0.0
C(29)	16.9 (q)	16.9 (s)	0.0

Table 4.1: Comparison of ^{13}C NMR data for natural sealutomicin C (reported by Igarashi)[14] and synthetic sealutomicin C. [a] 150 MHz, DMSO-d_6, proton-coupled; [b] 150 MHz, DMSO-d_6, proton-decoupled

The ^{1}H NMR data for the sample of synthetic sealutomicin C in deuterated DMSO matched those reported for natural sealutomicin C by Igarashi, with all resonances equal to within 0.02 ppm (**Table 4.2**).[14]

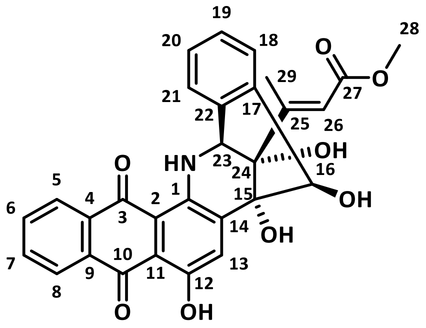

Position	δ_H[a] Natural[14]	δ_H Synthetic[a]	Δδ(Natural – Synthetic)
**C(1)NH**	**10.82 (d, *J* = 5.4 Hz)**	**10.83 (d, *J* = 5.5 Hz)**	**-0.01**
**C(1)**	-	-	
**C(2)**	-	-	
**C(3)**	-	-	
**C(4)**	-	-	
**C(5)**	**8.21 (m)**	**8.21 (dd, *J* = 7.5, 1.0 Hz)**	**0.00**
**C(6)**	**7.82 (td, *J* = 7.5, 1.4 Hz)**	**7.81 (td, *J* = 7.5, 1.0 Hz)**	**+0.01**
**C(7)**	**7.89 (td, *J* = 7.5, 1.4 Hz)**	**7.88 (td, *J* = 7.5, 1.0)**	**+0.01**
**C(8)**	**8.22 (m)**	**8.22 (dd, *J* = 7.5, 1.0 Hz)**	**0.00**
**C(9)**	-	-	
**C(10)**	-	-	
**C(11)**	-	-	
**C(12)**	-	-	
**C(12)OH**	**13.57 (s)**	**13.58 (s)**	**-0.01**
**C(13)**	**7.62 (s)**	**7.63 (s)**	**-0.01**
**C(14)**	-	-	
**C(15)**	-	-	
**C(15)OH**	**5.95 (s)**	**5.95 (s)**	**0.00**
**C(16)**	**4.57 (d, *J* = 5.9 Hz)**	**4.59 (d, *J* = 6.0 Hz)**	**-0.02**
**C(16)OH**	**6.01 (d, *J* = 5.9 Hz)**	**6.01 (d, *J* = 6.0 Hz)**	**0.00**
**C(17)**	-	-	
**C(18)**	**7.33 (d, *J* = 7.5 Hz)**	**7.35 (d, *J* = 7.5 Hz)**	**-0.02**
**C(19)**	**7.18 (td, *J* = 7.5, 1.3 Hz)**	**7.19 (t, *J* = 7.5 Hz)**	**-0.01**
**C(20)**	**7.25 (t, *J* = 7.5 Hz)**	**7.27 (t, *J* = 7.5)**	**-0.02**
**C(21)**	**7.61 (dt *J* = 7.5, 1.3)**	**7.63 (d, *J* = 7.5 Hz)**	**-0.02**
**C(22)**	-	-	
**C(23)**	**4.94 (d, *J* = 5.4 Hz)**	**4.95 (d, *J* = 5.5 Hz)**	**-0.01**
**C(24)**	-	-	
**C(24)OH**	**5.94 (s)**	**5.94 (s)**	**0.00**
**C(25)**	-	-	
**C(26)**	**5.78 (q, *J* = 1.2 Hz)**	**5.79 (q, *J* = 1.0 Hz)**	**-0.01**
**C(27)**	-	-	
**C(28)**	**3.51 (s)**	**3.52 (s)**	**-0.01**
**C(29)**	**2.33 (d, *J* = 1.2 Hz)**	**2.34 (d, *J* = 1.0 Hz)**	**-0.01**

Table 4.2: Comparison of ^{1}H NMR data for natural sealutomicin C (reported by Igarashi)[14] and synthetic sealutomicin C . [a] 150 MHz, DMSO-d_6.

The graphical ^{1}H and ^{13}C NMR spectra for the sample of synthetic sealutomicin C in DMSO-d_6 were both in accordance with the analogous spectra reported by Igarashi[14] for natural sealutomicin C in the same solvent (Figure 4.2 and Figure 4.3).

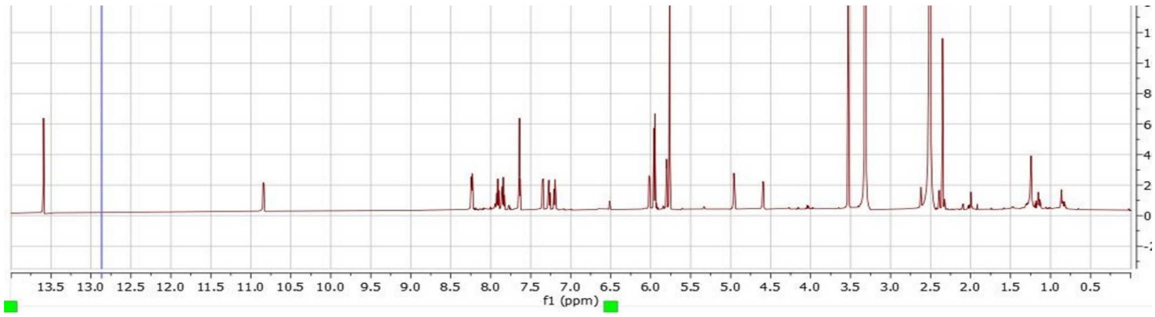

Figure 4.2: ^{1}H NMR spectrum of synthetic sealutomicin C in DMSO-d_6 (600 MHz); the ^{1}H NMR spectrum of natural sealutomicin C in DMSO-d_6 (600 MHz) can be found in Figure S21 of the Electronic Supporting Information of ref. 14.

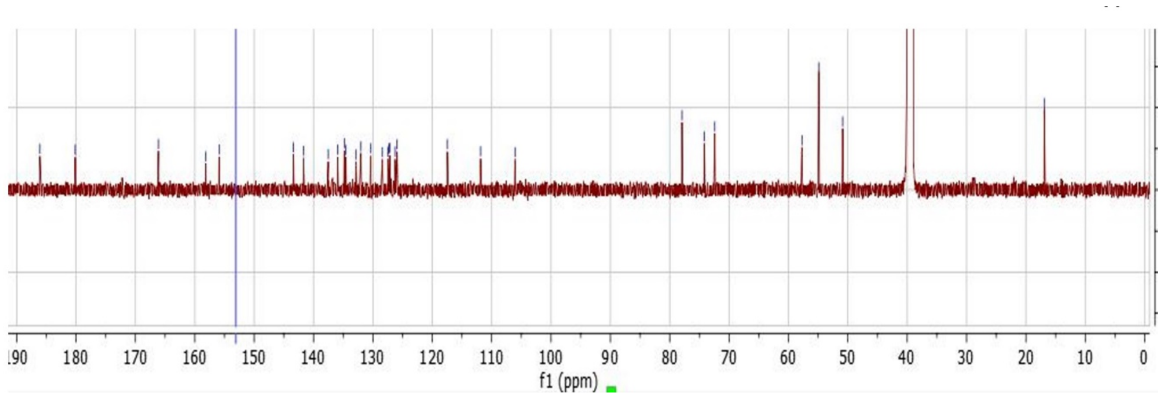

Figure 4.3: ^{13}C NMR spectrum of synthetic sealutomicin C in DMSO-d_6 (150 MHz): the ^{13}C NMR spectrum of natural sealutomicin C in DMSO-d_6 (150MHz) can be found in Figure S22 of the Electronic Supporting Information of ref. 14.

The ^{1}H and ^{13}C NMR data of sealutomicin C were compared to those reported for the compound proposed by Shen to be sealutomicin C (compound ***Shen-14*** of the referenced publication).[15] The graphical ^{1}H NMR spectrum for the sample of our synthetic sealutomicin C in acetone-d_6 (600 MHz) was in accordance with the graphical ^{1}H NMR spectrum of compound ***Shen-14*** reported by Shen in acetone-d_6 (700 MHz) (**Figure 4.5**). The resonances corresponding to the C(1)N**H**, C(12)O**H**, C(15)O**H**, C(16)O**H** and C(24)O**H** protons are visible in the ^{1}H NMR spectrum of natural sealutomicin C; however, they are not observed in the ^{1}H NMR spectrum of synthetic sealutomicin C due to proton exchange.

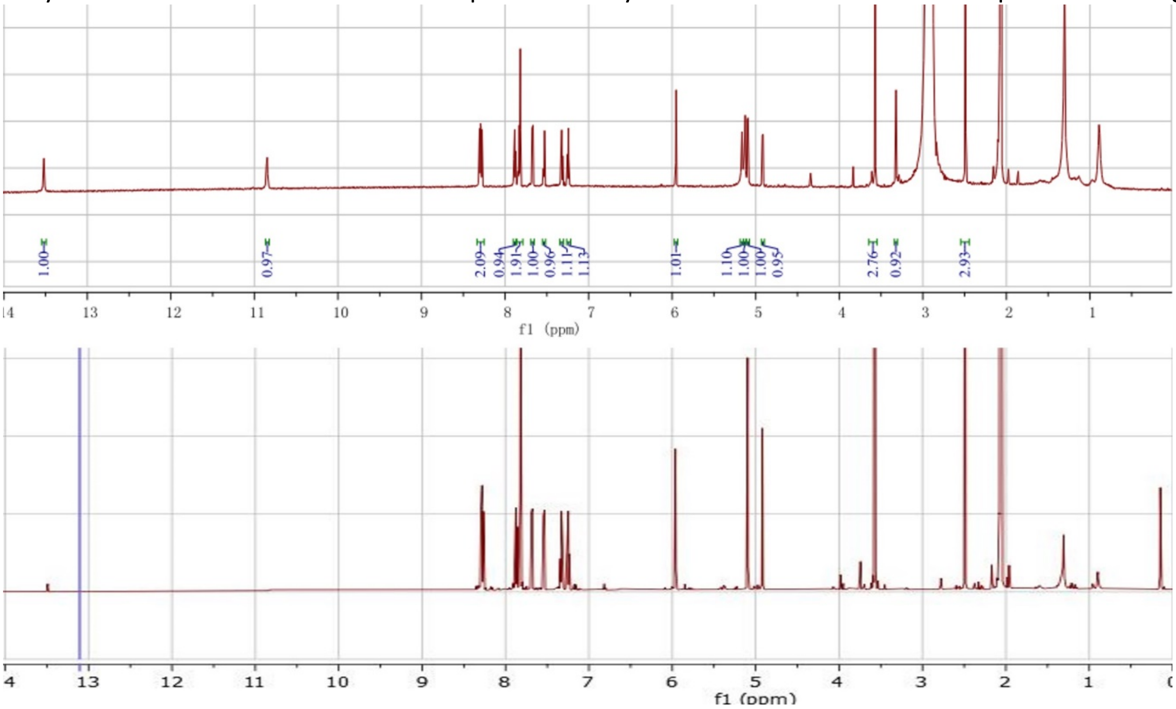

Figure 4.5: Top) ^{1}H NMR spectrum of natural sealutomicin C in acetone-d_6 (700 MHz) reported by Shen – Adapted with permission from Yan, X.; Chen, J. J.; Adhikari, A.; Teijaro, C. N.; Ge, H.; Crnovcic, I.; Chang, C. Y.; Annaval, T.; Yang, D.; Rader, C.; Shen, B. Comparative Studies of the Biosynthetic Gene Clusters for Anthraquinone-Fused Enediynes Shedding Light into the Tailoring Steps of Tiancimycin Biosynthesis. *Org. Lett*. **2018,** *20*, 5918-5921. Copyright **2018** American Chemical Society;[15]

bottom) ^{1}H NMR spectrum of synthetic sealutomicin C in acetone-d_6 (600 MHz).

The tabulated ^{13}C NMR data of ***Shen-14*** reported by Shen[15] does not match the ^{13}C NMR spectrum of ***Shen-14***. Consequently, the ^{13}C NMR data for the sample of synthetic sealutomicin C in deuterated acetone was compared to the peak-picked resonances shown on the graphical ^{13}C NMR spectrum of compound ***Shen-14***[15] (**Table 4.3** and **Figure 4.6**) in the same solvent. With the exception of phenolic **C**(12), the ^{13}C NMR resonances of synthetic sealutomicin C were equal to within 0.3 ppm of those reported by Shen for compound ***Shen-14***. We attribute the greater difference observed for the ^{13}C NMR chemical shift of **C**(12) to hydrogen bonding effects of the phenol unit in the deuterated acetone solvent.

The ^{13}C NMR spectrum of ***Shen-14*** reported by Shen shows two resonances at ~141 ppm and ~135 ppm which were not been peak-picked; we propose that these resonances correspond to those observed for **C**(14) and **C**(4) respectively. The ^{13}C NMR resonance of **C**(9) in the sample of synthetic sealutomicin C partially overlaps with the stronger resonance of **C**(7); we propose that this overlap is responsible for the **C**(9) resonance of compound ***Shen-14*** not being reported by Shen.

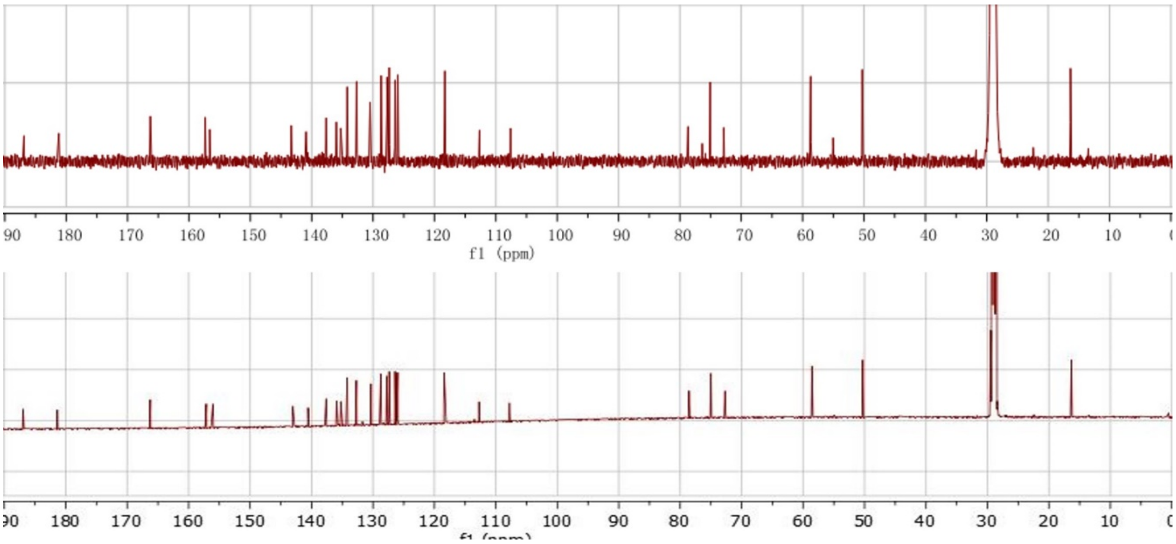

Figure 4.6: Top) ^{13}C NMR spectrum of natural sealutomicin C in acetone-d_6 (176 MHz) reported by Shen - Adapted with permission from Yan, X.; Chen, J. J.; Adhikari, A.; Teijaro, C. N.; Ge, H.; Crnovcic, I.; Chang, C. Y.; Annaval, T.; Yang, D.; Rader, C.; Shen, B. Comparative Studies of the Biosynthetic Gene Clusters for Anthraquinone-Fused Enediynes Shedding Light into the Tailoring Steps of Tiancimycin Biosynthesis. *Org. Lett*. **2018,** *20*, 5918-5921. Copyright **2018** American Chemical Society;[15]

bottom) ^{13}C NMR spectrum of synthetic sealutomicin C in acetone-d_6 (150 MHz).

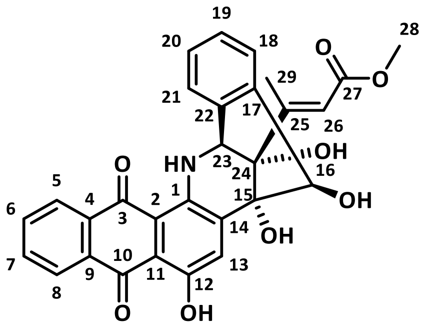

Position	δ_C[a] Natural[15]	δ_C[b] Synthetic	Δδ(Natural – Synthetic)
C(1)	143.3	143.0 (s)	0.3
C(2)	107.6	107.7 (s)	-0.1
C(3)	181.2	181.3 (s)	-0.1
C(4)	-	135.2 (s)	-
C(5)	126.4	126.4 (s)	0.0
C(6)	134.2	134.1 (s)	0.1
C(7)	132.7	132.6 (s)	0.1
C(8)	126.0	125.9 (s)	0.1
C(9)	-	132.7 (s)	-
C(10)	186.9	186.9 (s)	0.0
C(11)	112.7	112.7 (s)	0.0
C(12)	156.6	156.0 (s)	0.6
C(13)	130.5	130.3 (s)	0.2
C(14)	-	140.5 (s)	-
C(15)	78.7	78.5 (s)	0.2
C(16)	75.0	74.9 (s)	0.1
C(17)	136.0	135.9 (s)	0.1
C(18)	127.4	127.3 (s)	0.1
C(19)	128.7	128.7 (s)	0.0
C(20)	127.7	127.7 (s)	0.0
C(21)	127.3	127.3 (s)	0.0
C(22)	137.7	137.6 (s)	0.1
C(23)	58.7	58.5 (s)	0.2
C(24)	72.9	72.6 (s)	0.3
C(25)	157.4	157.1 (s)	0.3
C(26)	118.3	118.4 (s)	-0.1
C(27)	166.3	166.2 (s)	0.1
C(28)	50.3	50.2 (s)	0.1
C(29)	16.4	16.3 (s)	0.1

Table 4.3: Comparison of ^{13}C NMR data for natural sealutomicin C (reported by Shen) and synthetic sealutomicin C. [a] 176 MHz, Acetone-d_6, peak-picked resonance chemical shifts from graphical NMR spectrum; [b] 150 MHz, Acetone-d_6.

The close agreement between the NMR data for our sample of synthetic sealutomicin C and the data reported for compound ***Shen-14*** by Shen[15s] enabled us to conclude that the two compounds are indeed the same structure, as proposed by Igarashi.

Single Crystal X-ray Diffraction

Low temperature (J. Appl. Crystallogr., 1986, 19, 105) single crystal X-ray diffraction data were collected using a (Rigaku) Oxford Diffraction SuperNova diffractometer. Raw frame data were reduced using CrysAlisPro and the structures were solved using 'Superflip' [Palatinus, L.; Chapuis, G. J. Appl. Crystallogr. 2007, 40, 786-790] before refinement with CRYSTALS [(J. Appl. Crystallogr., 2003, 36, 1487; Parois, P.; Cooper, R. I.; Thompson, A. L. Chem. Cent. J. 2015, 9, 30] as per the SI (CIF). Full refinement details are given in the Supporting Information (CIF); Crystallographic data have been deposited with the Cambridge Crystallographic Data Centre (CCDC 2327991-92) and can be obtained via www.ccdc.cam.ac.uk/data_request/cif.

Table S1. Crystal data and structure refinement for (–)-18a.

CCDC code	2327991	
Empirical formula	C28 H25 N O5	
Formula weight	455.51	
Temperature	150 K	
Wavelength	1.54184 Å	
Crystal system / Space group	Monoclinic /	P 2_1
Unit cell dimensions	a = 11.9537(4) Å	α= 90°.
	b = 6.1904(2) Å	β= 102.519(4)°.
	c = 16.0313(6) Å	γ = 90°.
Volume	1158.08(7) $Å^3$	
Z	2	
Density (calculated)	1.306 Mg/m^3	
Absorption coefficient	0.730 mm^{-1}	
F(000)	480	
Crystal size	0.40 x 0.05 x 0.02 mm^3	
Theta range for data collection	2.824 to 75.911°.	
Index ranges	-13<=h<=15, -7<=k<=7, -20<=l<=19	
Reflections collected	11221	
Independent reflections	4774 [R(int) = 0.053]	
Completeness to theta = 75.911°	99.5 %	
Absorption correction	Semi-empirical from equivalents	
Max. and min. transmission	0.99 and 0.51	
Refinement method	Full-matrix least-squares on F^2	
Data / restraints / parameters	4774 / 1 / 308	
Goodness-of-fit on F^2	1.0020	
Final R indices [I>2sigma(I)]	R1 = 0.0505, wR2 = 0.1302	
R indices (all data)	R1 = 0.0578, wR2 = 0.1396	
Absolute structure parameter	0.2(2)	
Largest diff. peak and hole	0.34 and -0.27 e.$Å^{-3}$	

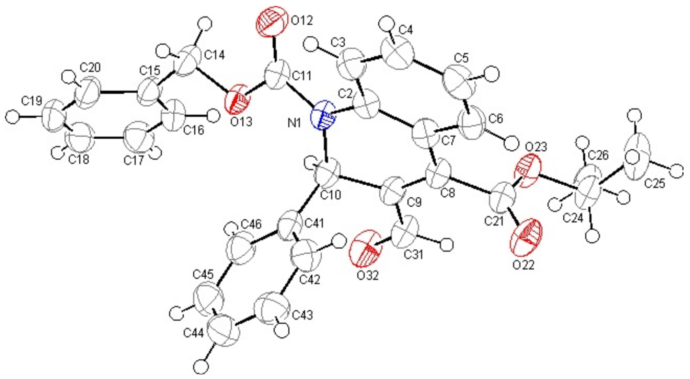

Table S2. Crystal data and structure refinement for (–)-18k.

CCDC code	2327992	
Empirical formula	C28 H24 Cl N O5	
Formula weight	489.95	
Temperature	150 K	
Wavelength	1.54184 Å	
Crystal system / Space group	Triclinic /	P 1
Unit cell dimensions	a = 6.3621(2) Å	α= 98.137(2)°.
	b = 9.4058(3) Å	β= 99.359(2)°.
	c = 10.6203(3) Å	γ = 102.050(3)°.
Volume	603.02(3) Å^3	
Z	1	
Density (calculated)	1.349 Mg/m^3	
Absorption coefficient	1.736 mm^{-1}	
F(000)	256	
Crystal size	0.25 x 0.21 x 0.17 mm^3	
Theta range for data collection	4.889 to 75.749°.	
Index ranges	-7<=h<=7, -11<=k<=11, -12<=l<=13	
Reflections collected	10900	
Independent reflections	4657 [R(int) = 0.018]	
Completeness to theta = 73.477°	99.6 %	
Absorption correction	Semi-empirical from equivalents	
Max. and min. transmission	0.74 and 0.64	
Refinement method	Full-matrix least-squares on F^2	
Data / restraints / parameters	4656 / 3 / 318	
Goodness-of-fit on F^2	0.9996	
Final R indices [I>2sigma(I)]	R1 = 0.0270, wR2 = 0.0717	
R indices (all data)	R1 = 0.0270, wR2 = 0.0717	
Absolute structure parameter	0.002(3)	
Extinction coefficient	22(3)	
Largest diff. peak and hole	0.15 and -0.17 e.Å^{-3}	

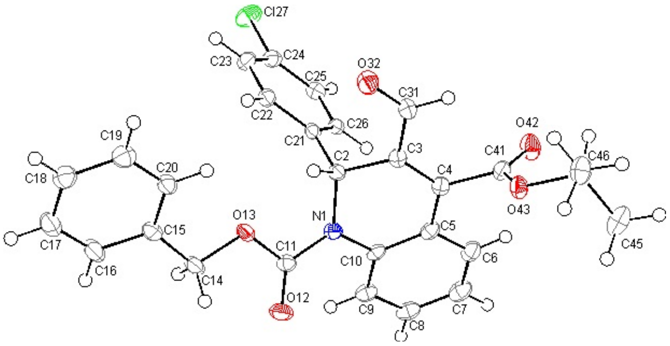

References

(1) Boeckman, R. K.; Tusch, D. J.; Biegasiewicz, K. F. (S)-1,1-Diphenylprolinol Trimethylsilyl Ether. *Org. Synth.* **2015**, *92*, 309-319.
(2) Ouyang, B.; Yu, T.; Luo, R.; Lu, G. The asymmetric Cu(II)-indolinylmethanol complex catalyzed Diels-Alder reaction of 2-vinylindoles with beta,gamma-unsaturated alpha-ketoesters: an efficient route to functionalized tetrahydrocarbazoles. *Org. Biomol. Chem.* **2014**, *12*, 4172-4176.
(3) Nikolic, N. A.; Beak, P. (R)-(+)-2-(Diphenylhydroxymethyl)Pyrrolidine. *Org. Synth.* **1997**, *74*, 23-28.
(4) Hayashi, Y.; Gotoh, H.; Hayashi, T.; Shoji, M. Diphenylprolinol silyl ethers as efficient organocatalysts for the asymmetric Michael reaction of aldehydes and nitroalkenes. *Angew. Chem. Int. Ed. Engl.* **2005**, *44*, 4212-4215.
(5) Gao, D.; Cui, C. N-heterocyclic carbene organocatalysts for dehydrogenative coupling of silanes and hydroxyl compounds. *Chem. Eur. J.* **2013**, *19*, 11143-11147.
(6) Yamagishi, M.; Yamada, Y.; Ozaki, K.-i.; Tani, J.; Suzuki, M. Quinazolin-2ones Having a Spirohydantoin Ring. II. Synthesis of Several Spiro[imidazolidine-4, 4'(1'H)-quinazoline]-2, 2', 5(3'H)-triones via 5-Hydroxyhydantoin Derivatives. *Chem. Pharm. Bull.* **1991**, *39*, 626-629.
(7) Tacconi, G.; Righetti, P. P.; Desimoni, G. Einfache Darstellung von N-substituierten Isatinen. *J. für Prakt. Chemie* **1973**, *315*, 339-344.
(8) Meisner, J. S.; Sedbrook, D. F.; Krikorian, M.; Chen, J.; Sattler, A.; Carnes, M. E.; Murray, C. B.; Steigerwald, M.; Nuckolls, C. Functionalizing molecular wires: a tunable class of α,ω-diphenyl-μ,ν-dicyano-oligoenes. *Chem. Sci.* **2012**, *3*, 1007-1014.
(9) Kim, E.; Koh, M.; Lim, B. J.; Park, S. B. Emission wavelength prediction of a full-color-tunable fluorescent core skeleton, 9-aryl-1,2-dihydropyrrolo[3,4-b]indolizin-3-one. *J. Am. Chem. Soc.* **2011**, *133*, 6642-6649.
(10) Plietker, B.; Niggemann, M. RuCl3/CeCl3/NaIO4: A New Bimetallic Oxidation System for the Mild and Efficient Dihydroxylation of Unreactive Olefins. *J. Org. Chem.* **2005**, *70*, 2402-2405.
(11) Burke, S. D.; Hong, J.; Lennox, J. R.; Mongin, A. P. Synthetic Studies of Antitumor Macrolide Rhizoxin: Stereoselective Syntheses of the C(1)−C(9) and C(12)−C(26) Subunits. *J. Org. Chem.* **1998**, *63*, 6952-6967.
(12) Wang, S.; Guillot, R.; Carpentier, J. F.; Sarazin, Y.; Bour, C.; Gandon, V.; Leboeuf, D. Synthesis of Bridged Tetrahydrobenzo[b]azepines and Derivatives through an Aza-Piancatelli Cyclization/Michael Addition Sequence. *Angew. Chem. Int. Ed. Engl.* **2020**, *59*, 1134-1138.
(13) Nicolaou, K. C.; Wang, Y.; Lu, M.; Mandal, D.; Pattanayak, M. R.; Yu, R.; Shah, A. A.; Chen, J. S.; Zhang, H.; Crawford, J. J.; et al. Streamlined Total Synthesis of Uncialamycin and Its Application to the Synthesis of Designed Analogues for Biological Investigations. *J. Am. Chem. Soc.* **2016**, *138*, 8235-8246.
(14) Igarashi, M.; Sawa, R.; Umekita, M.; Hatano, M.; Arisaka, R.; Hayashi, C.; Ishizaki, Y.; Suzuki, M.; Kato, C. Sealutomicins, new enediyne antibiotics from the deep-sea actinomycete Nonomuraea sp. MM565M-173N2. *J. Antibiot.* **2021**, *74*, 291-299.
(15) Yan, X.; Chen, J. J.; Adhikari, A.; Teijaro, C. N.; Ge, H.; Crnovcic, I.; Chang, C. Y.; Annaval, T.; Yang, D.; Rader, C.; Shen, B. Comparative Studies of the Biosynthetic Gene Clusters for Anthraquinone-Fused Enediynes Shedding Light into the Tailoring Steps of Tiancimycin Biosynthesis. *Org. Lett.* **2018**, *20*, 5918-5921.

NMR Spectra

(*S*)-Diphenyl(pyrrolidin-2-yl)methanol (*S*)-17c

^{1}H NMR (500 MHz, $CDCl_3$)

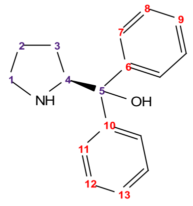

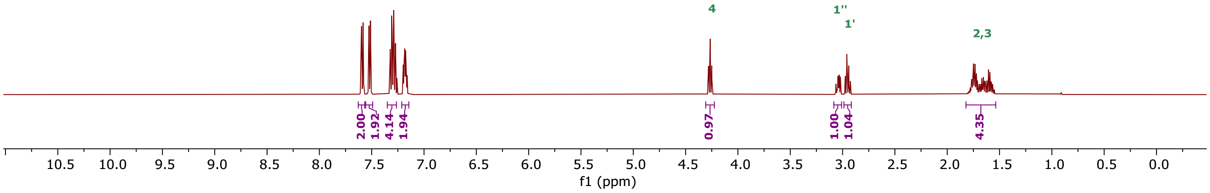

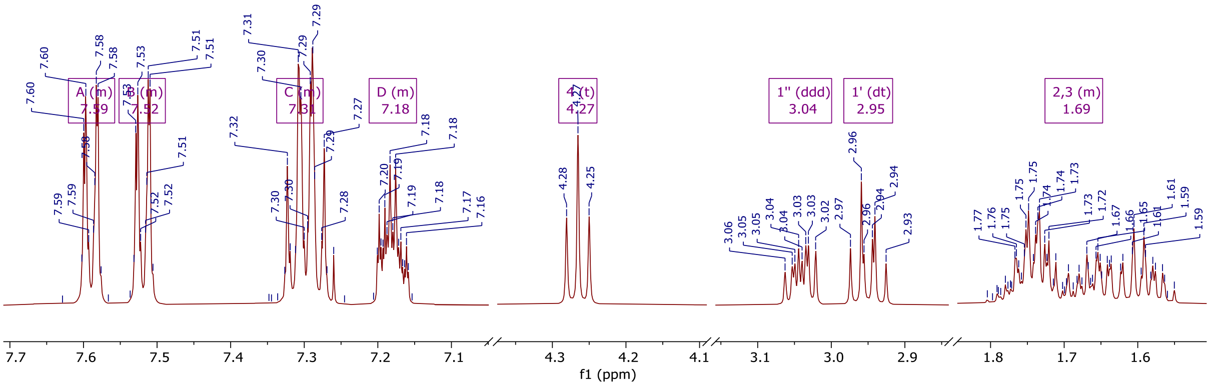

^{13}C NMR (126 MHz, $CDCl_3$)

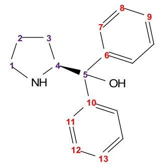

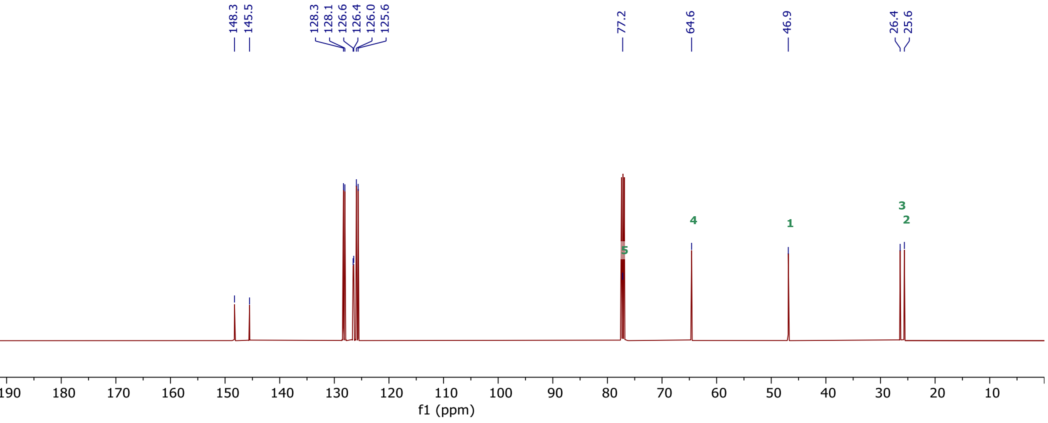

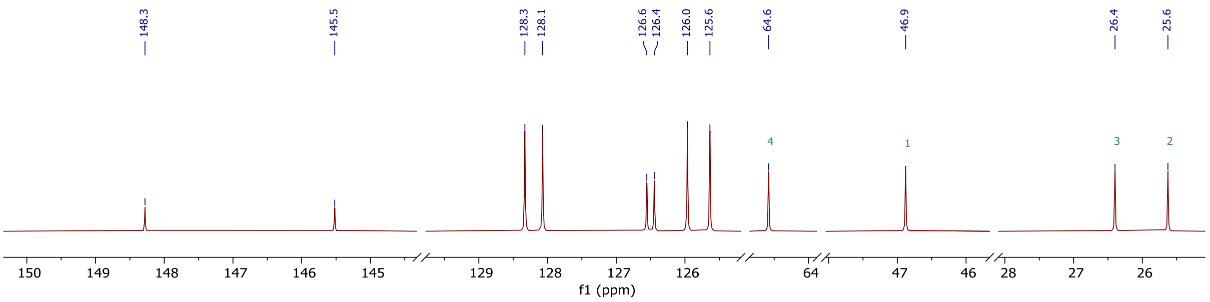

$^{1}H/^{1}H$ COSY

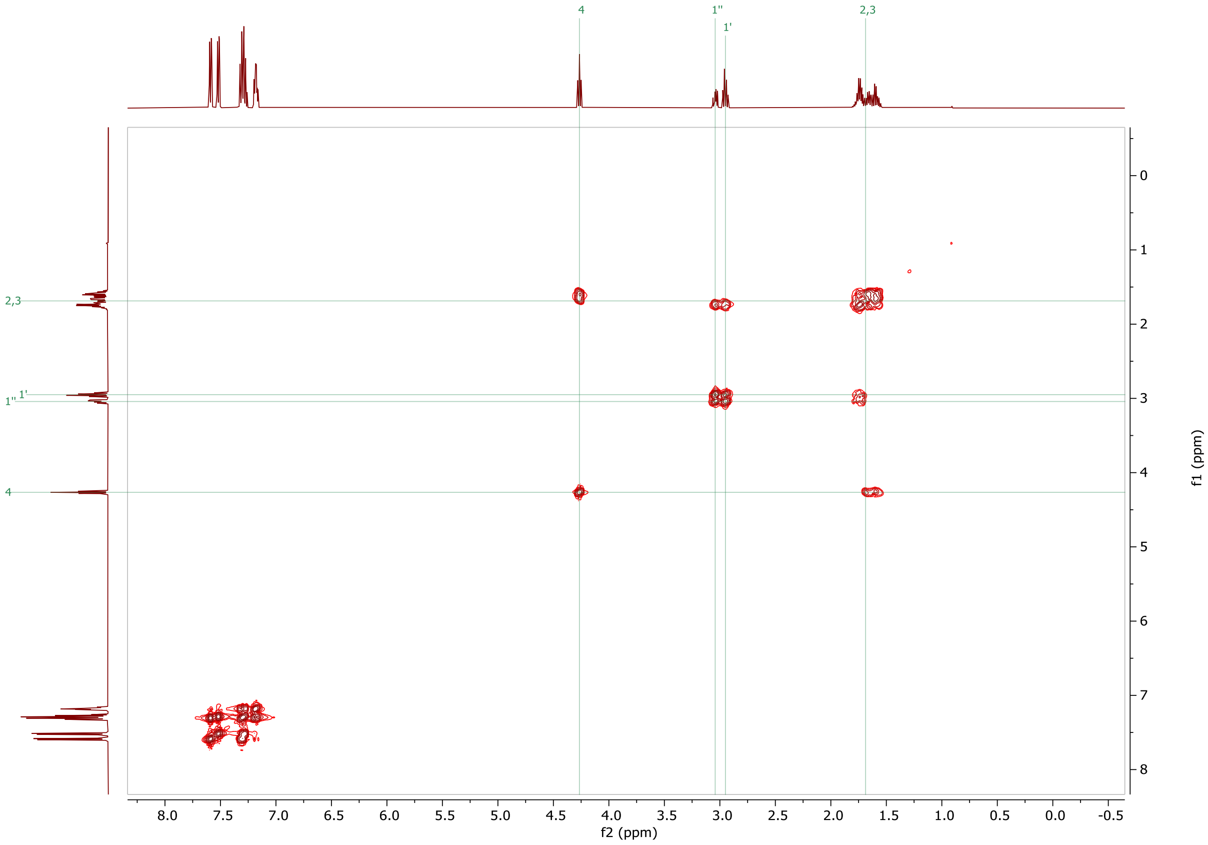

$^{1}H/^{13}C$ HSQC

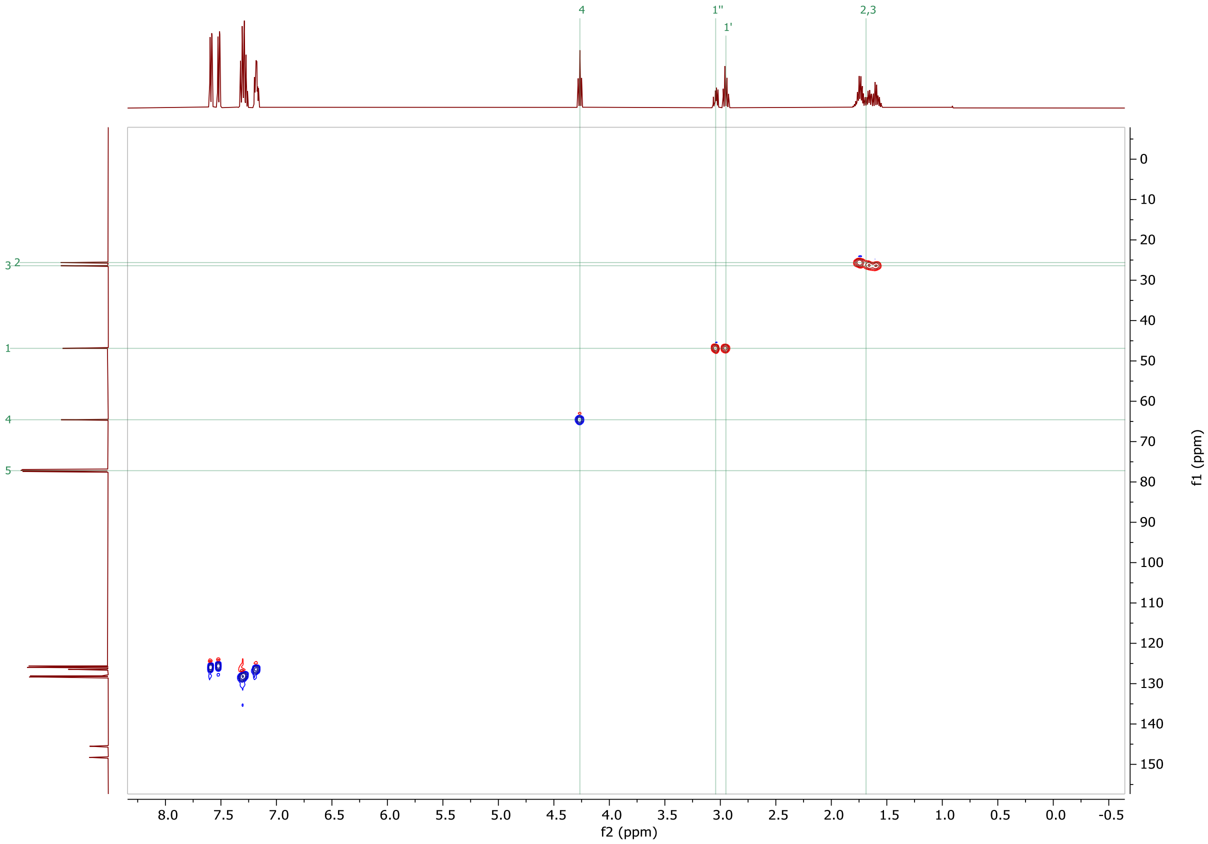

$^{1}H/^{13}C$ HMBC

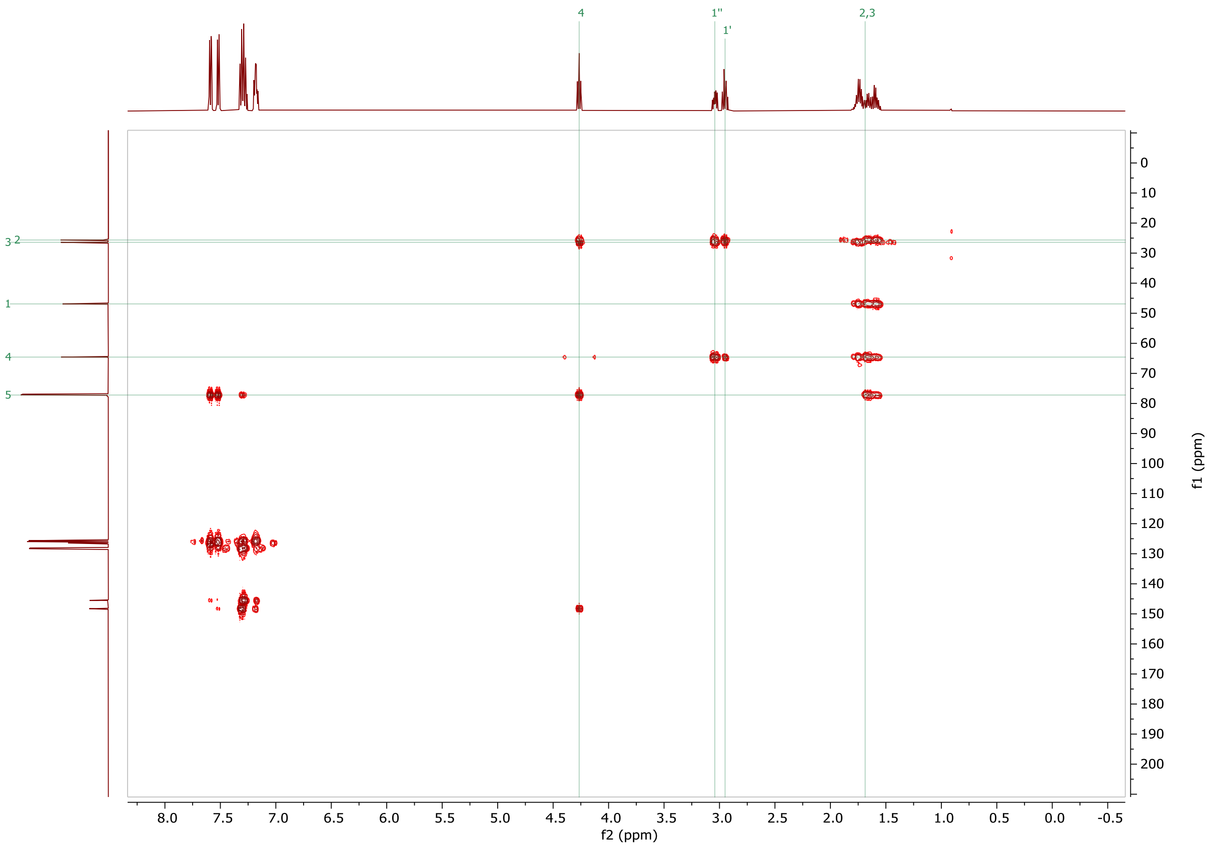

(*S*)-2-(Diphenyl((triethylsilyl)oxy)methyl)pyrrolidine (*S*)-17a

^{1}H NMR (600 MHz, $CDCl_3$)

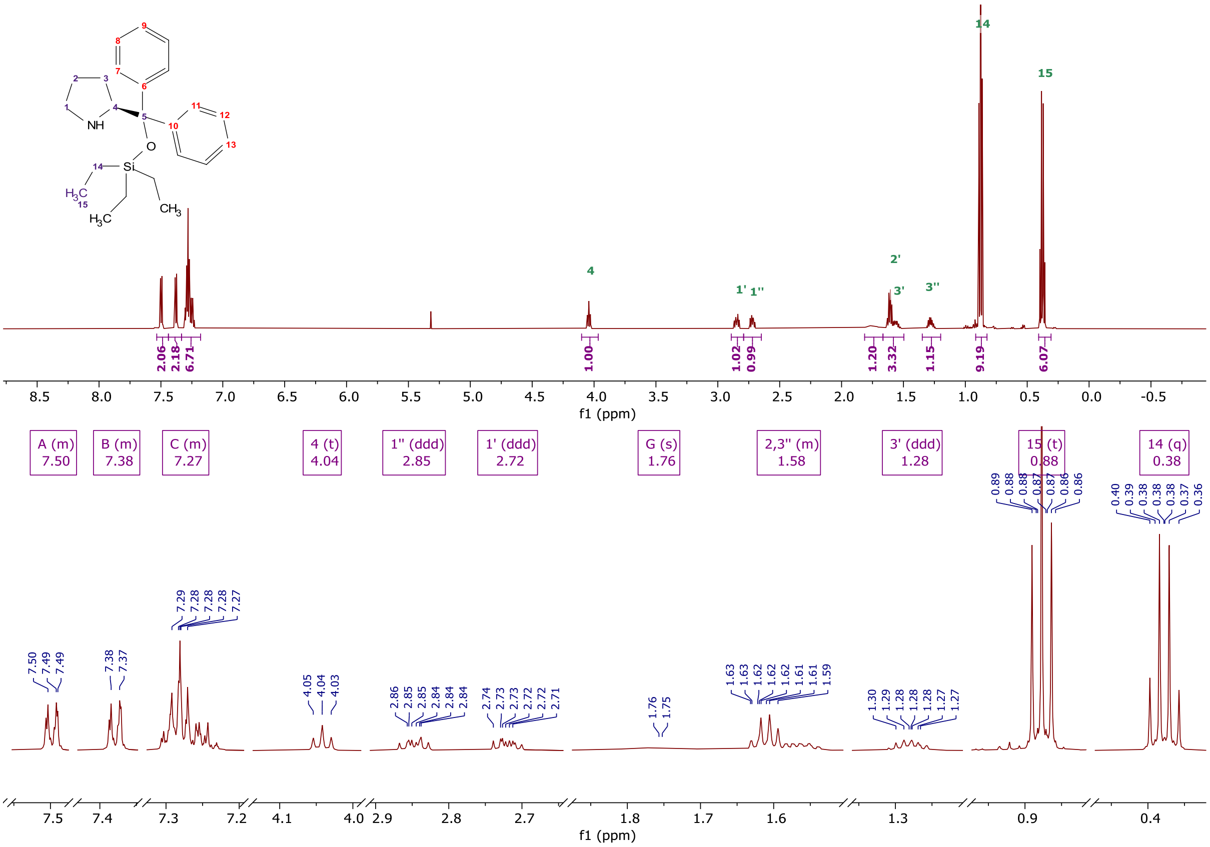

^{13}C NMR (151 MHz, $CDCl_3$)

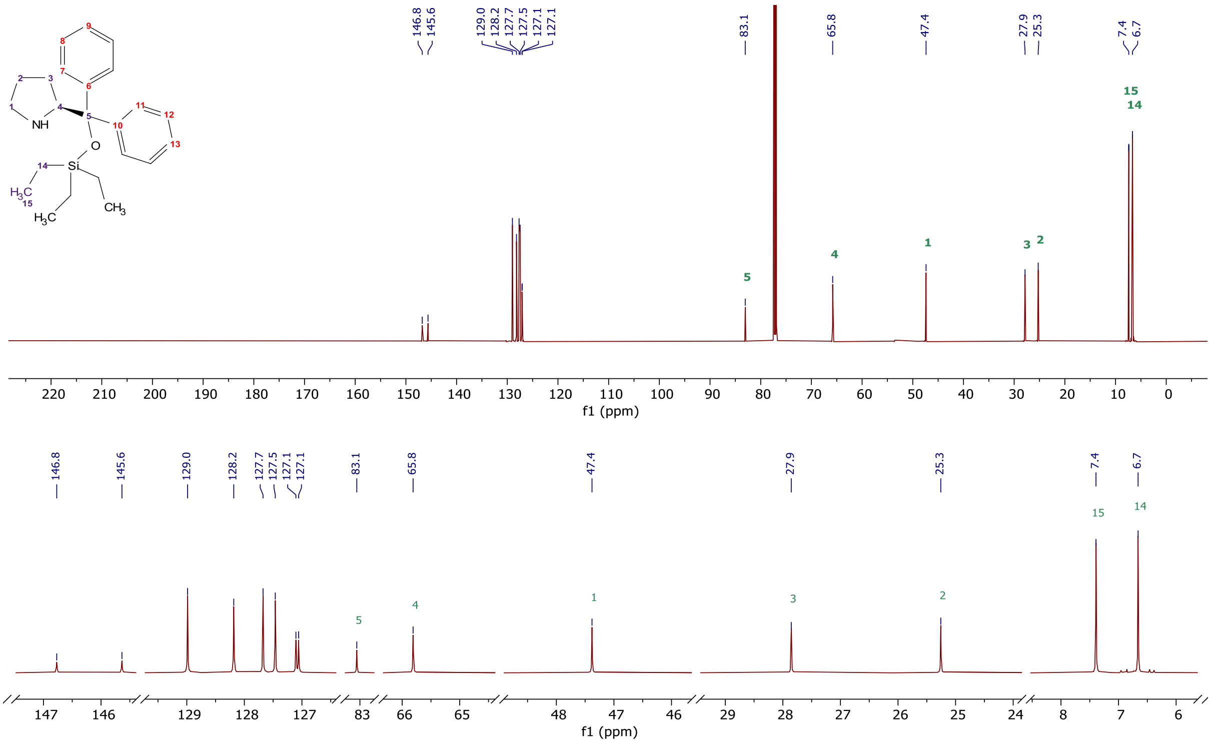

$^1H/^1H$ COSY

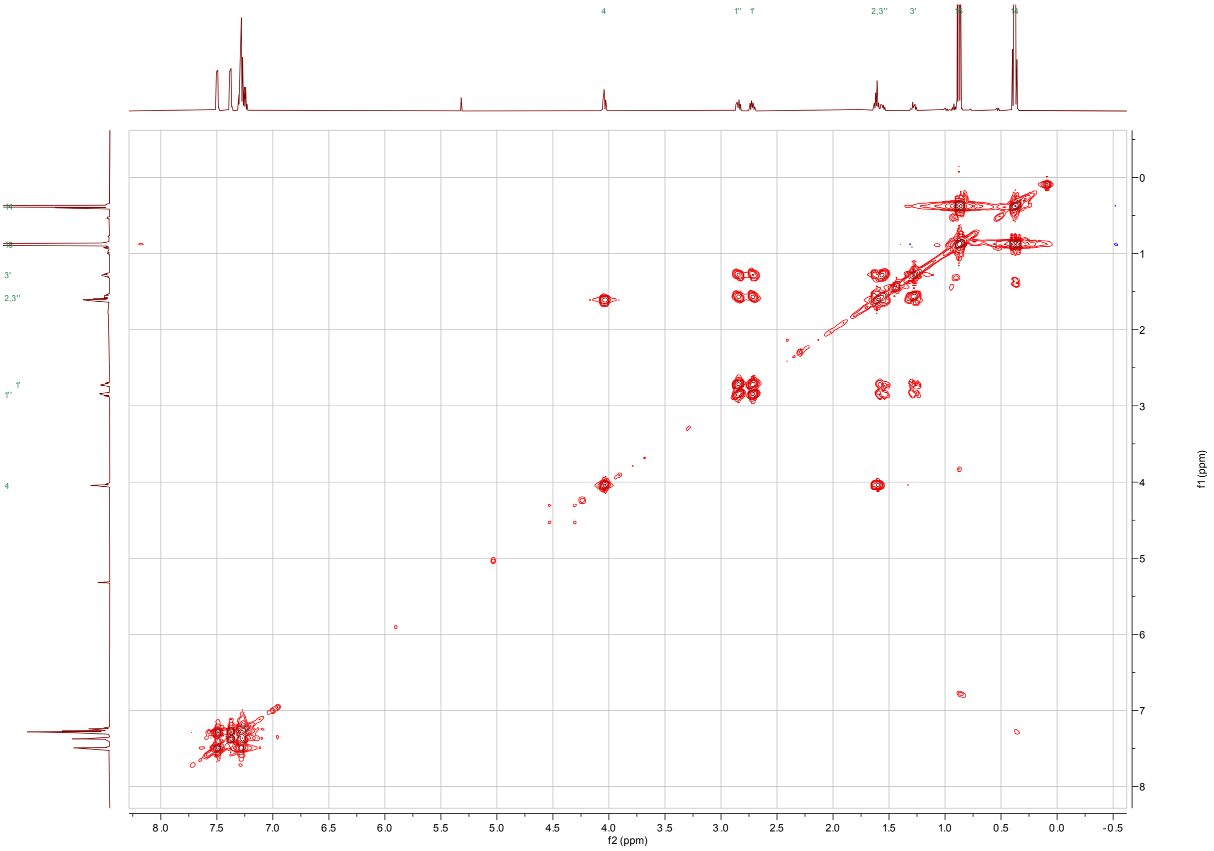

$^1H/^{13}C$ HSQC

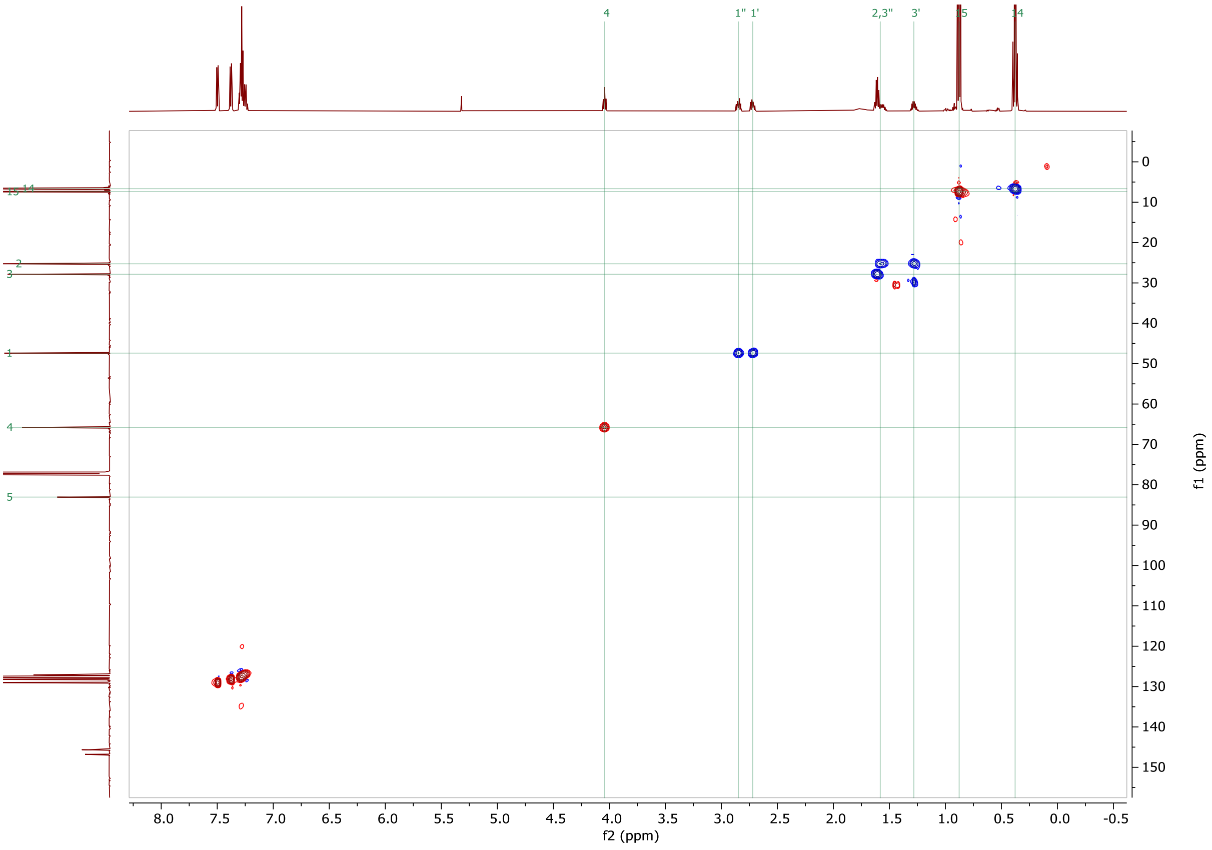

$^{1}H/^{13}C$ HMBC

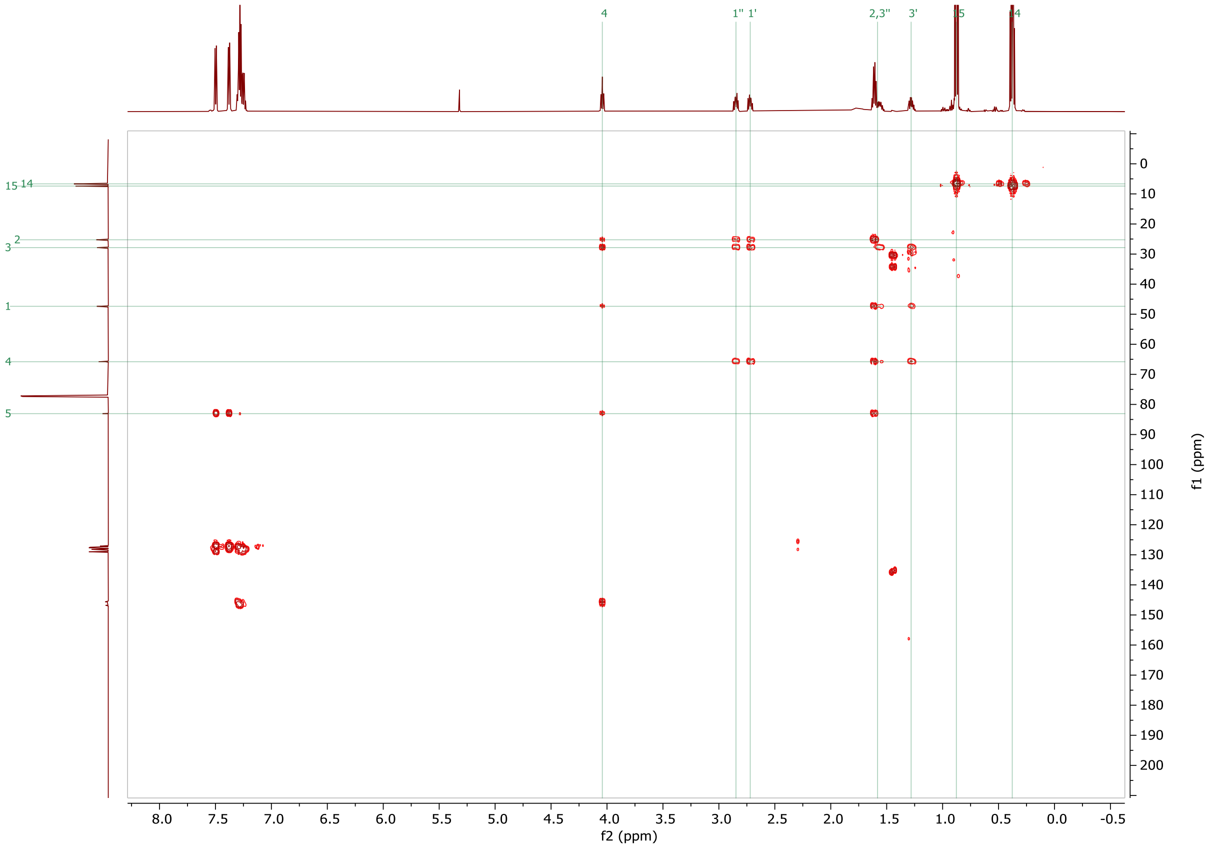

(*S*)-2-(Diphenyl((trimethylsilyl)oxy)methyl)pyrrolidine (*S*)-17b

^{1}H NMR (500 MHz, $CDCl_3$)

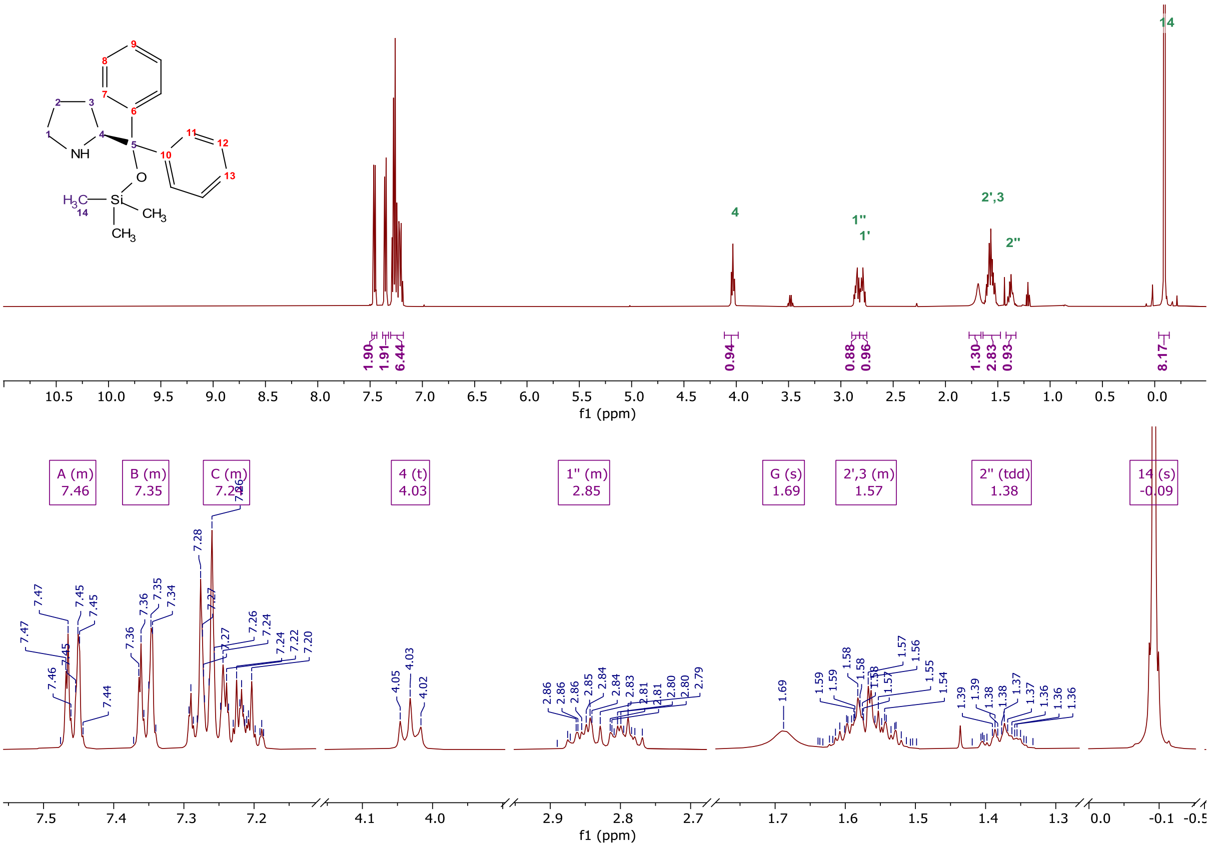

^{13}C NMR (126 MHz, $CDCl_3$)

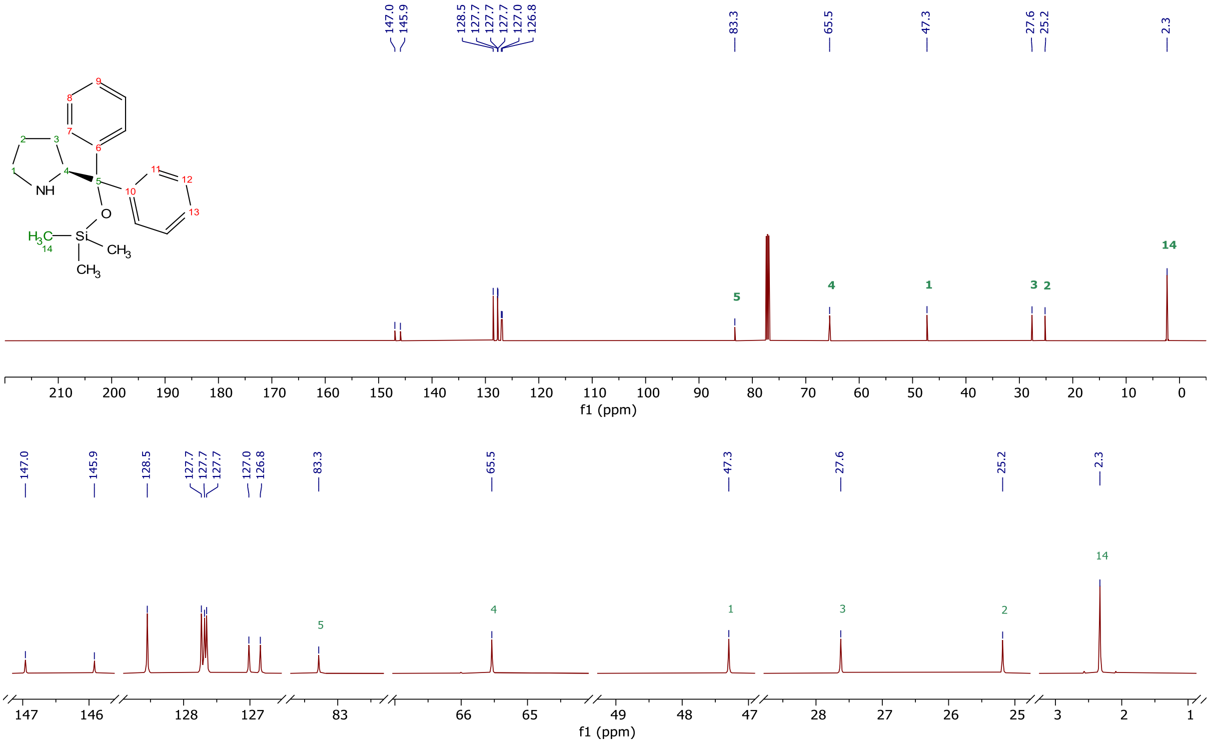

$^{1}H/^{1}H$ COSY

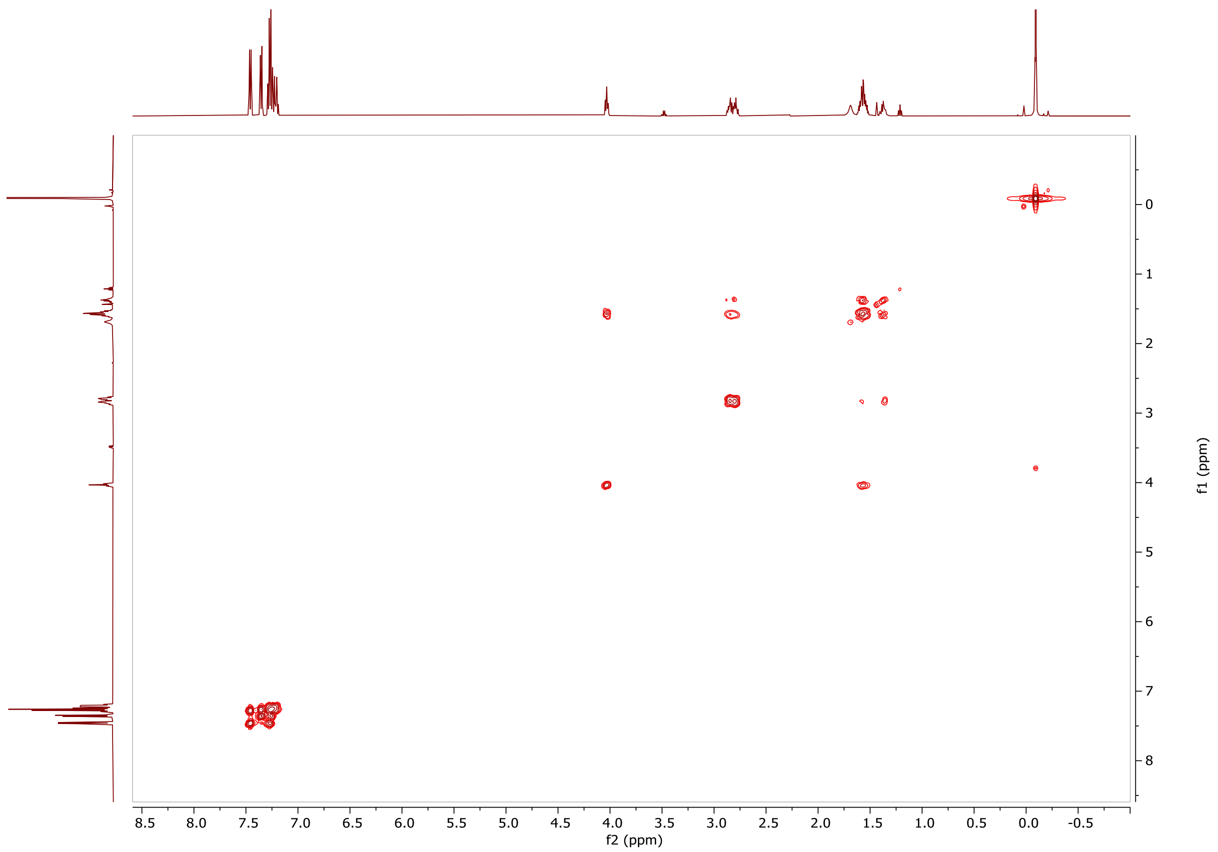

$^{1}H/^{13}C$ HSQC

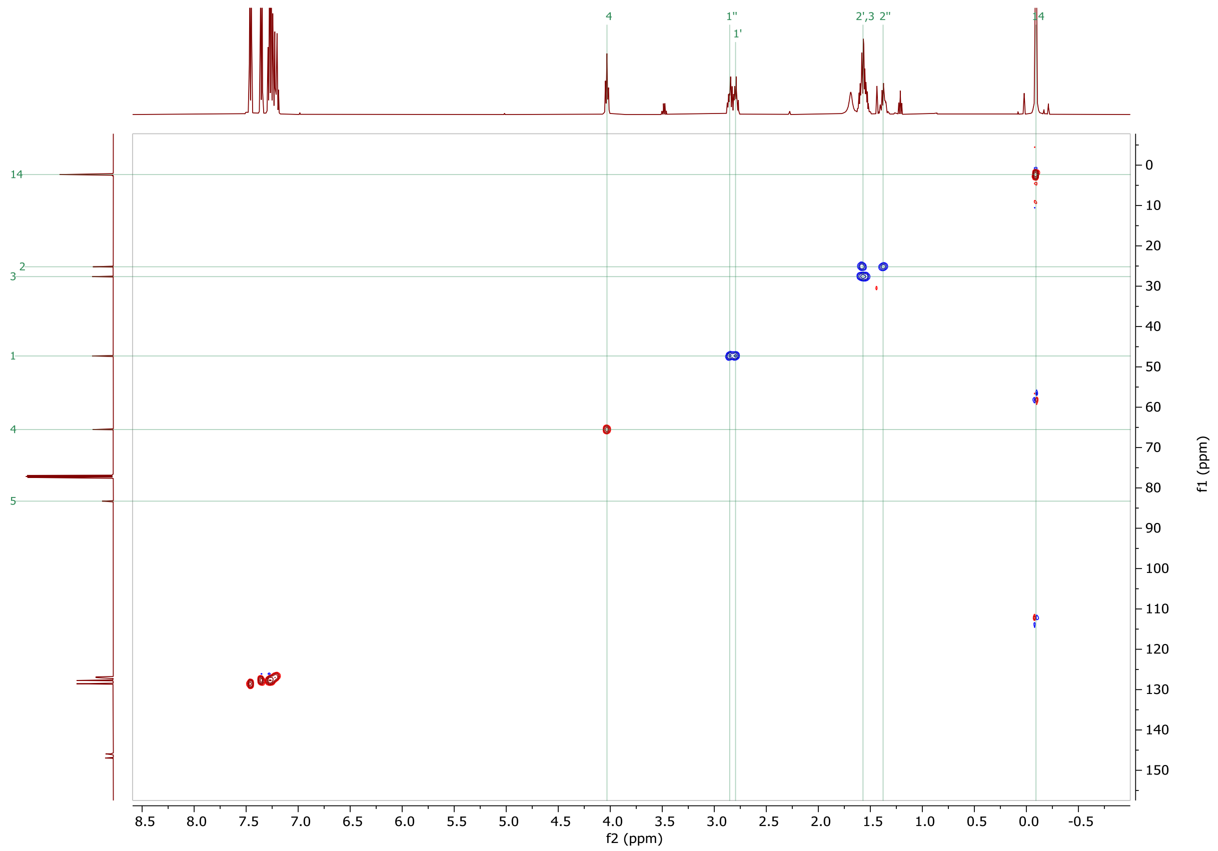

$^{1}H/^{13}C$ HMBC

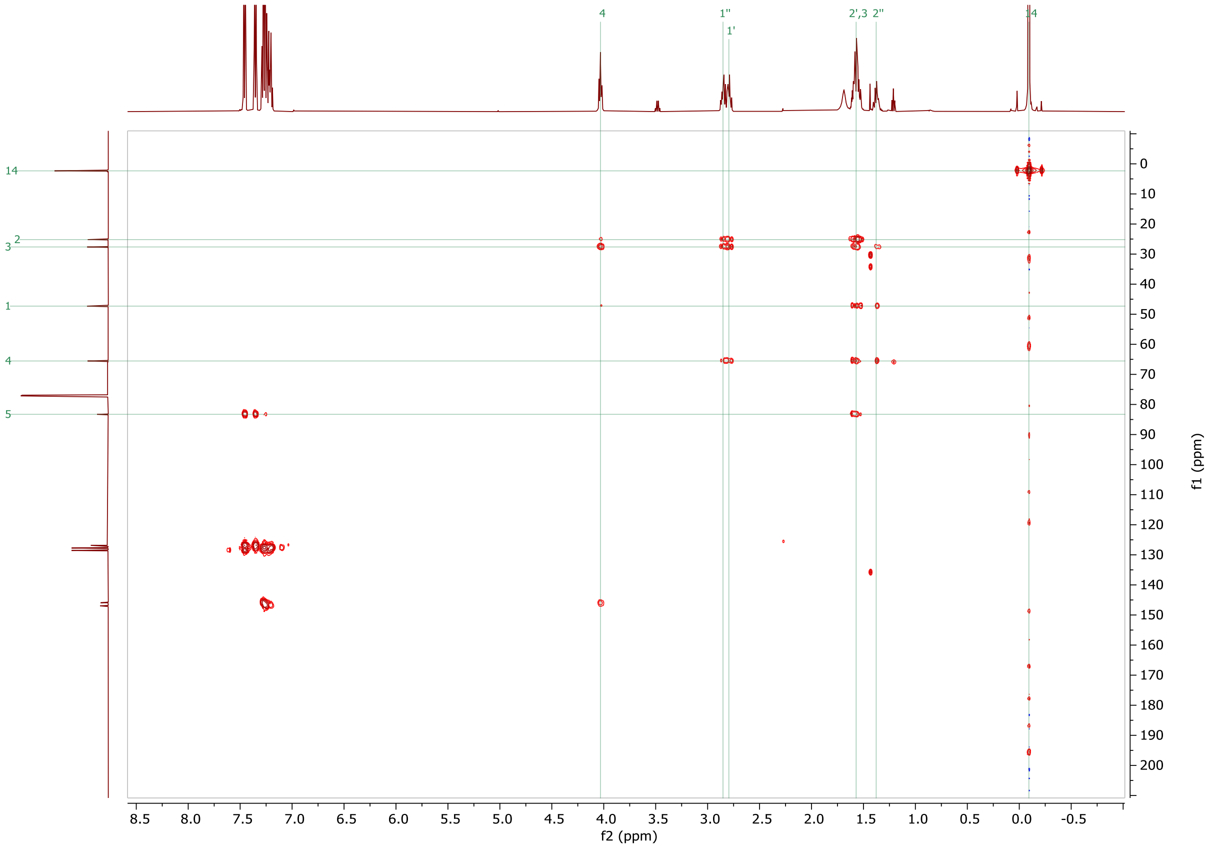

α-Ketoester substrates and precursors

Benzyl 2,3-dioxoindoline-1-carboxylate S1

^{1}H NMR (500 MHz, DMSO-d_6)

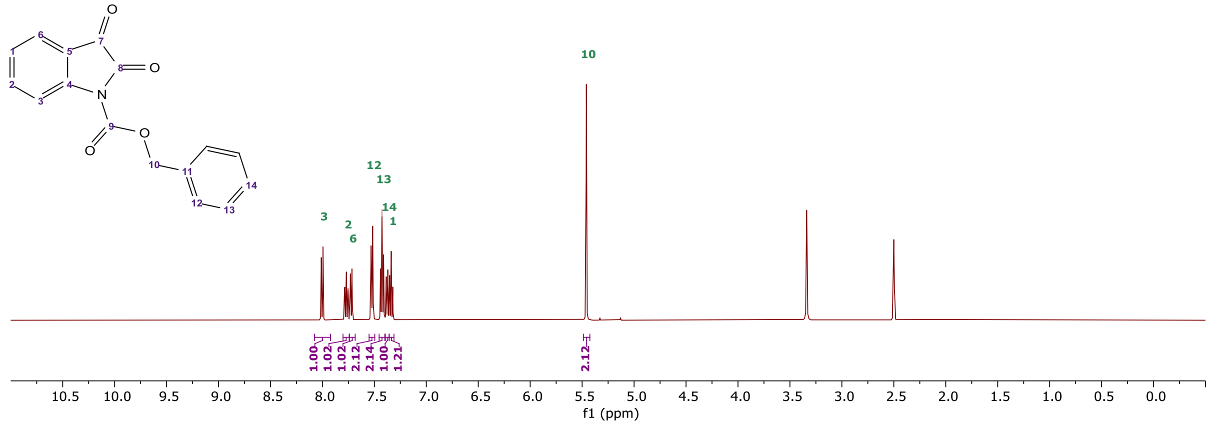

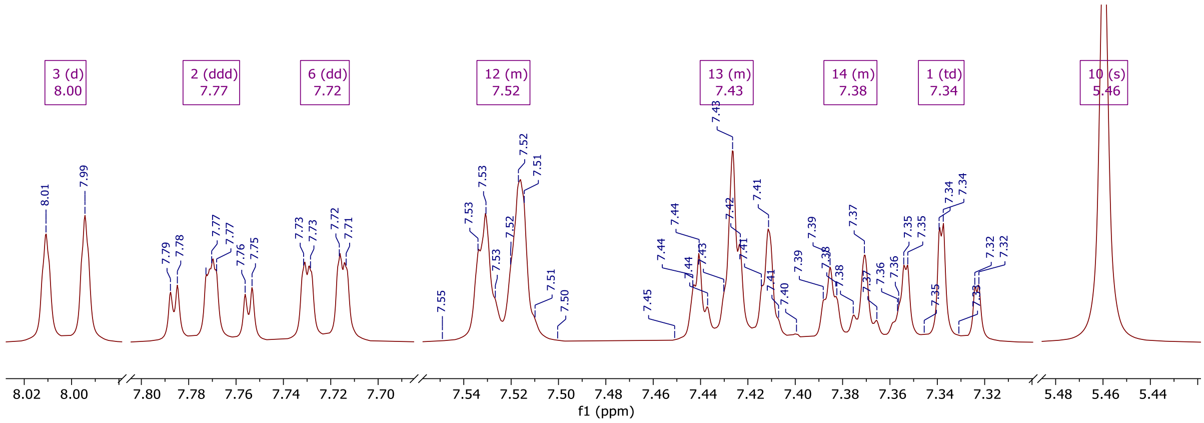

^{13}C NMR (126 MHz, DMSO-d_6)

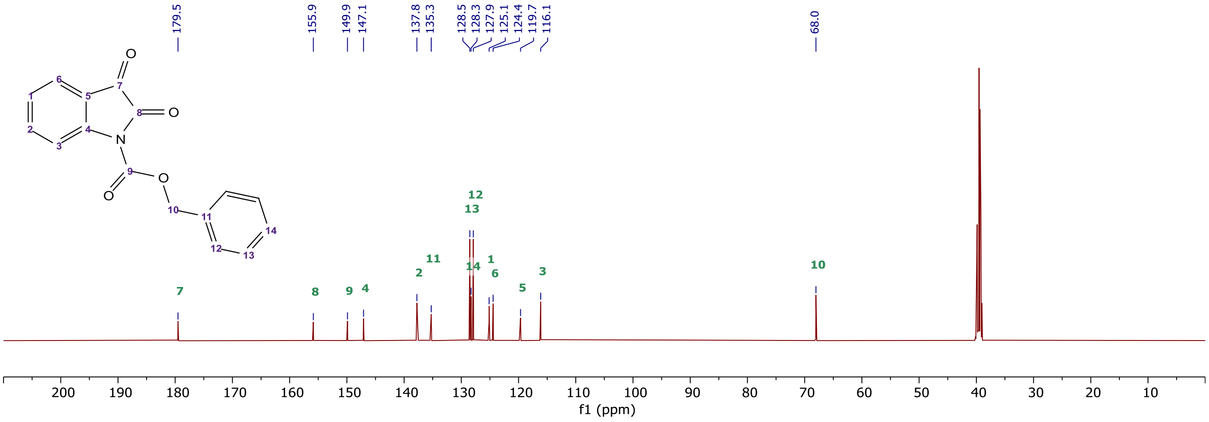

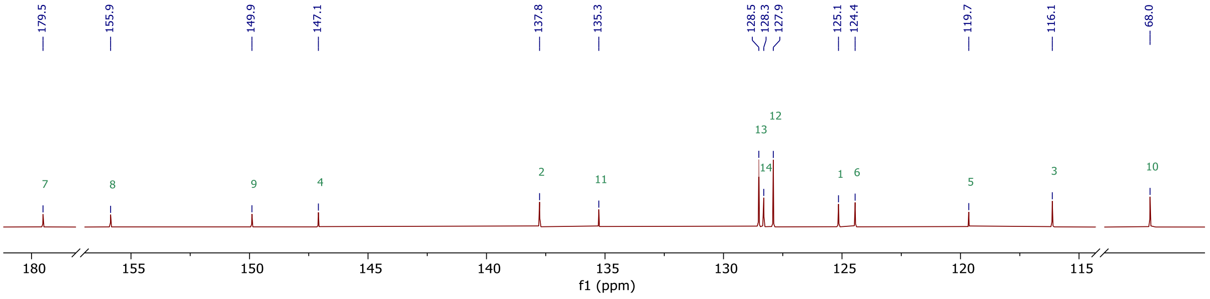

$^1H/^1H$ COSY

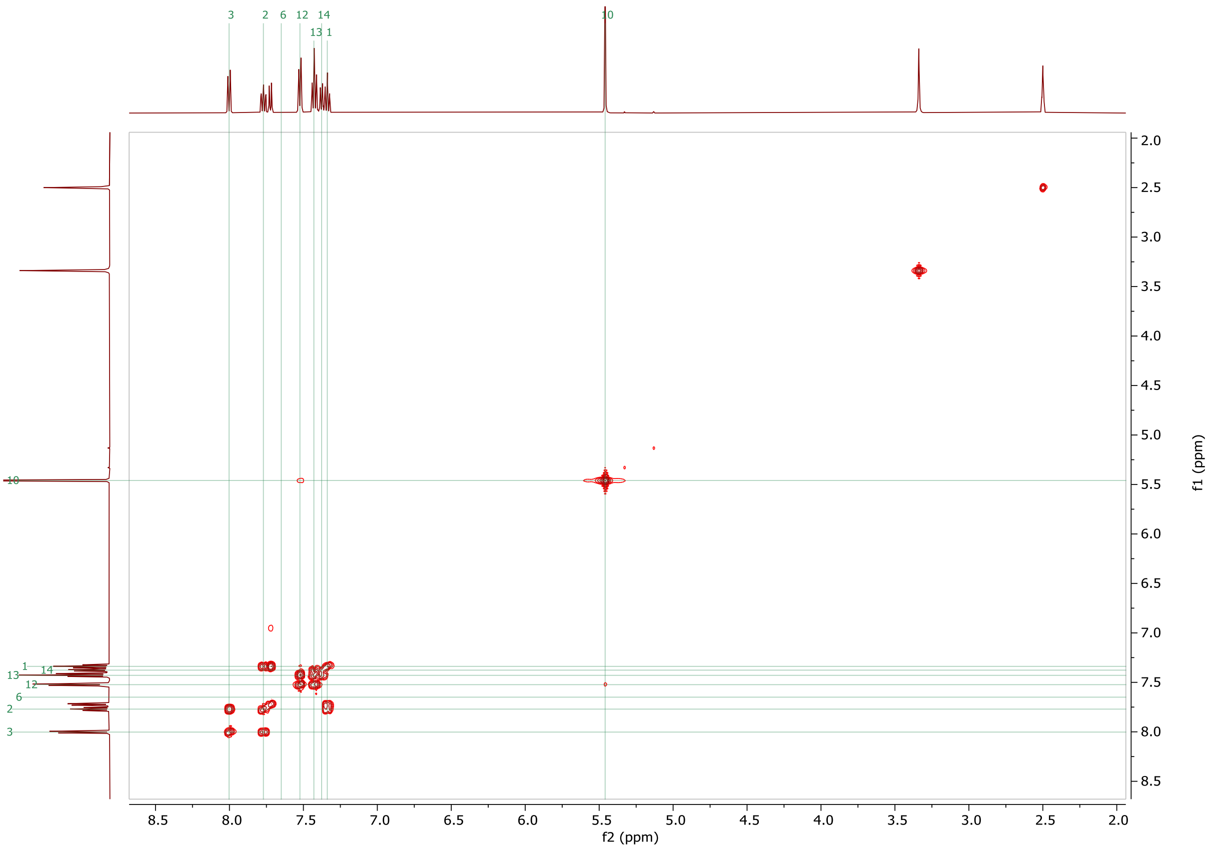

$^1H/^{13}C$ HSQC

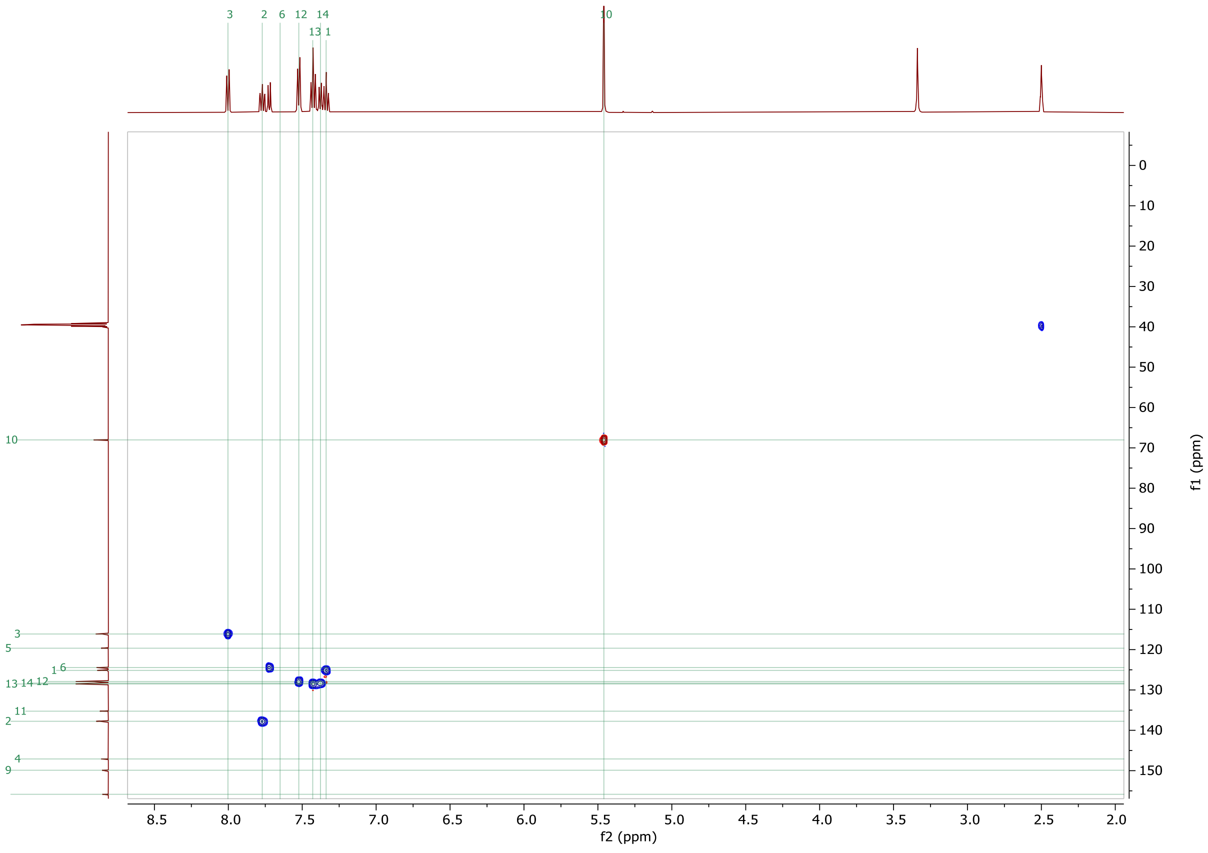

$^{1}H/^{13}C$ HMBC

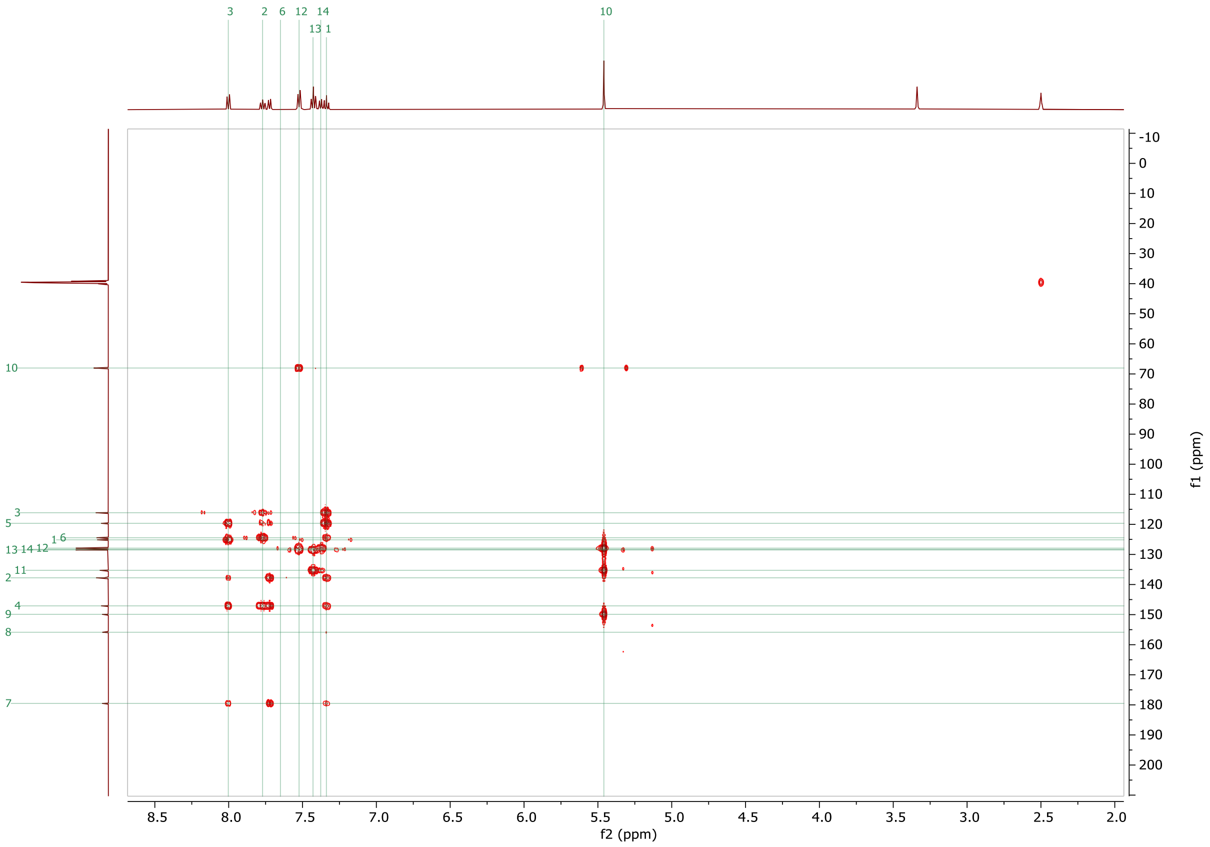

Isopropyl 2-(2-(((benzyloxy)carbonyl)amino)phenyl)-2-oxoacetate 15a

^{1}H NMR (500 MHz, $CDCl_3$)

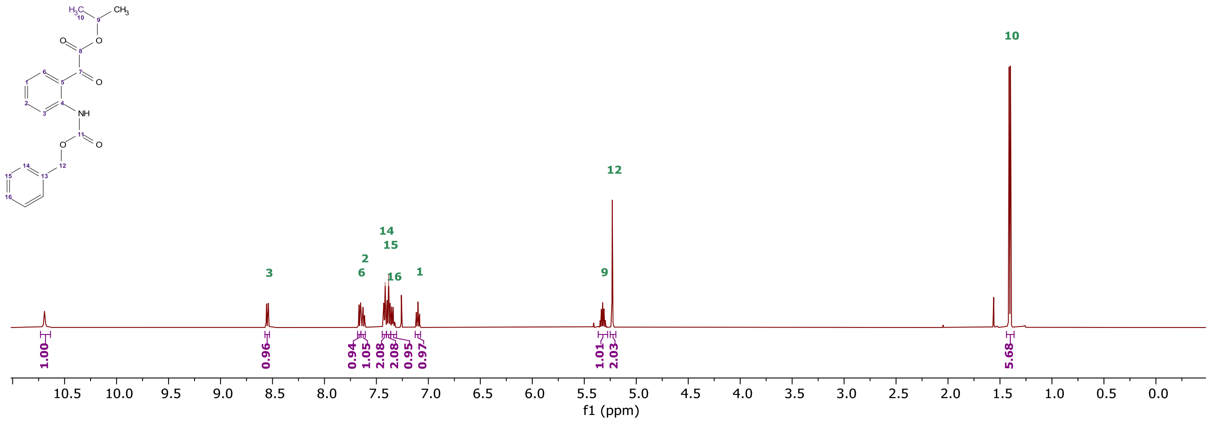

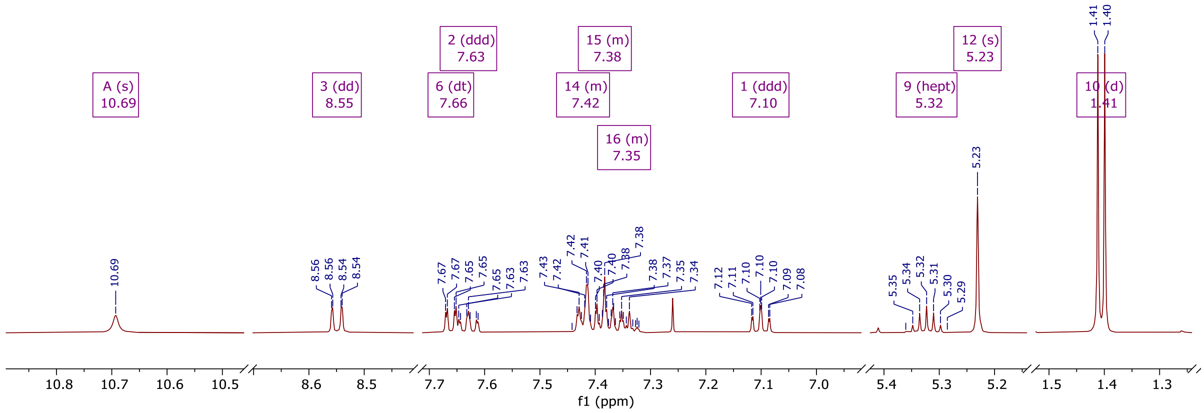

^{13}C NMR (126 MHz, $CDCl_3$)

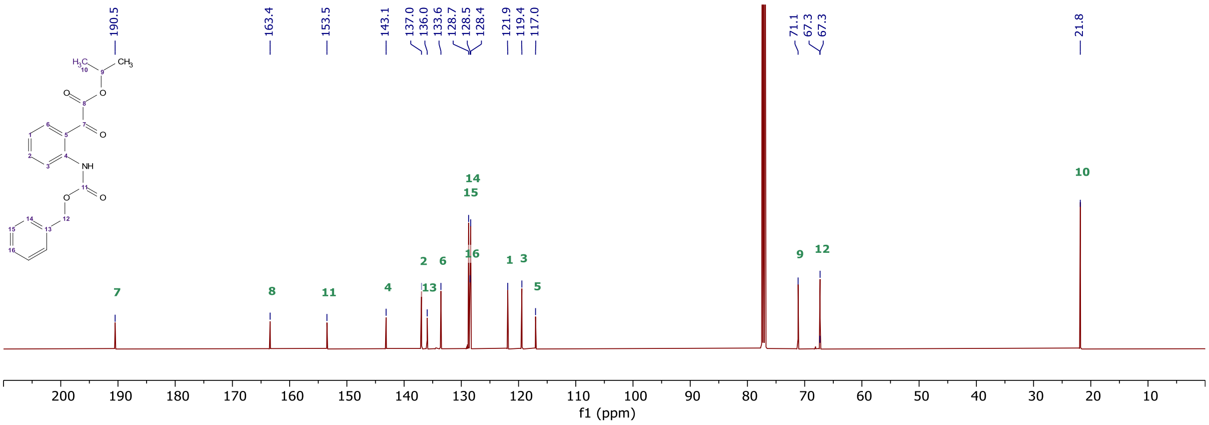

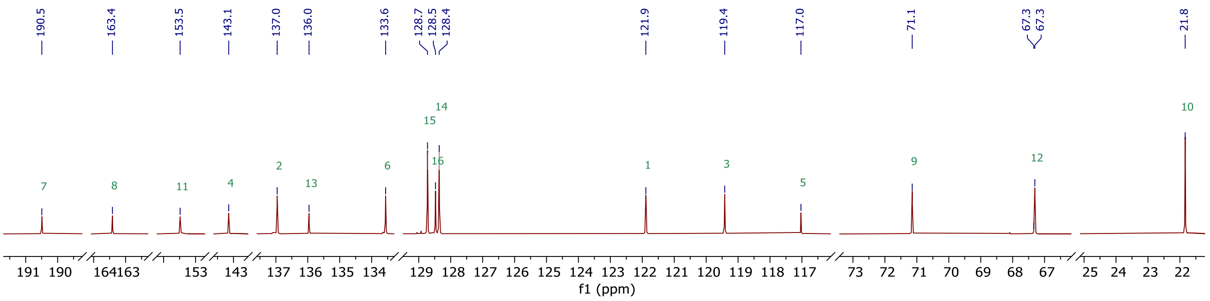

$^1H/^1H$ COSY

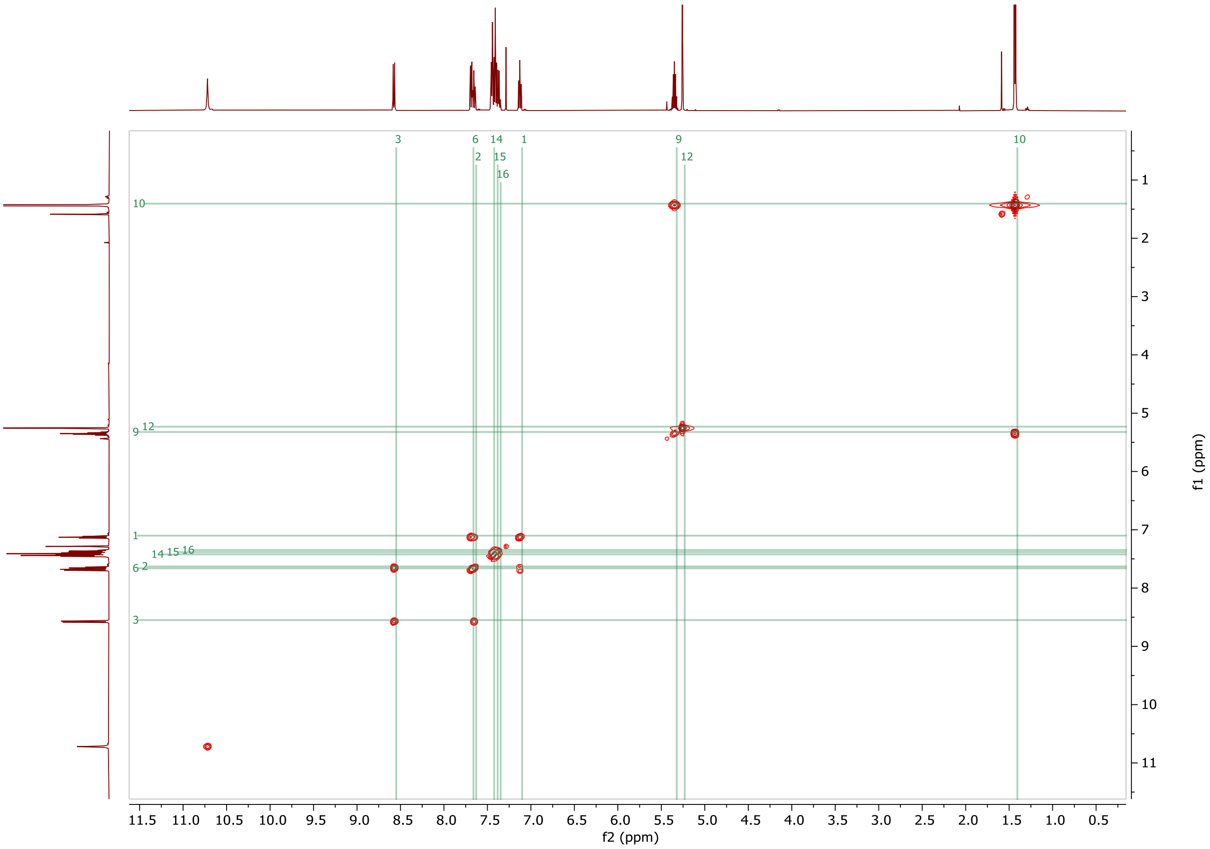

$^1H/^{13}C$ HSQC

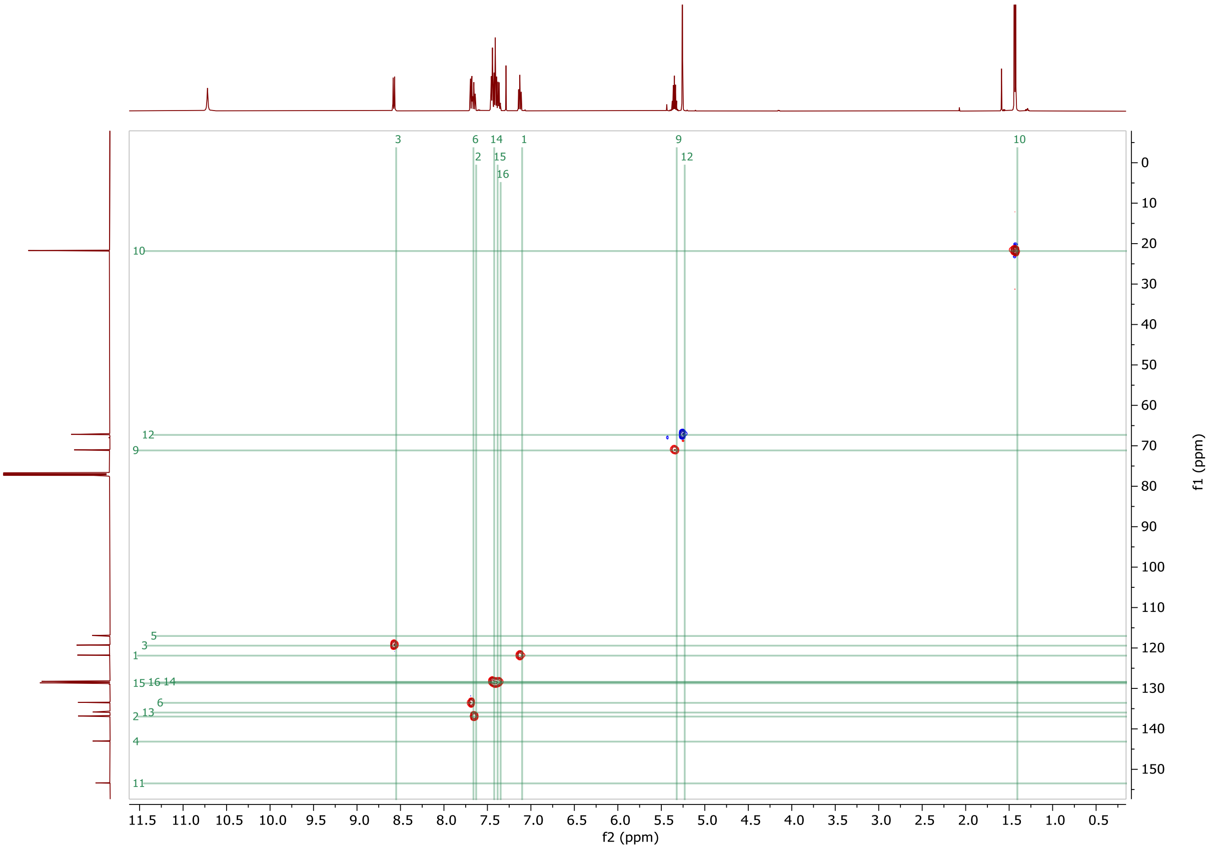

$^{1}H/^{13}C$ HMBC

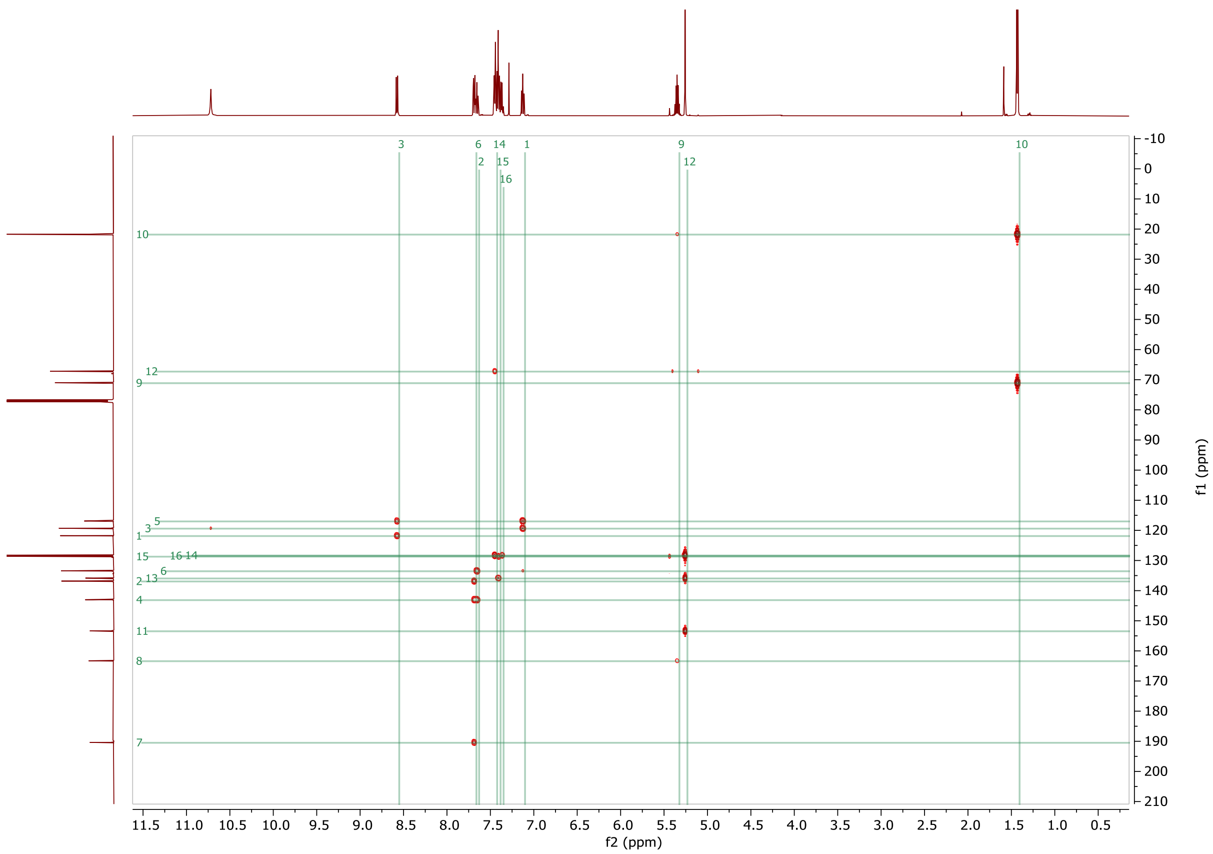

Methyl 2-(2-(((benzyloxy)carbonyl)amino)phenyl)-2-oxoacetate 15b

^{1}H NMR (500 MHz, DMSO-d_6)

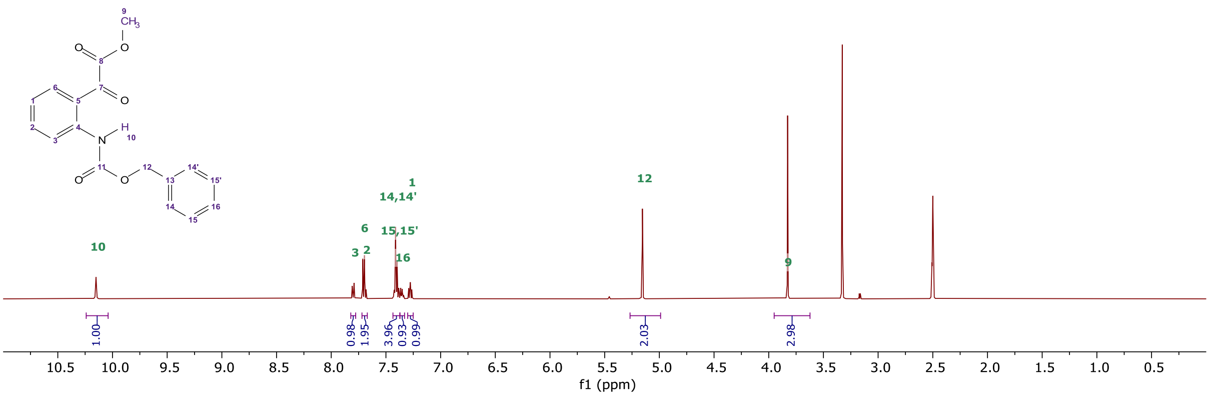

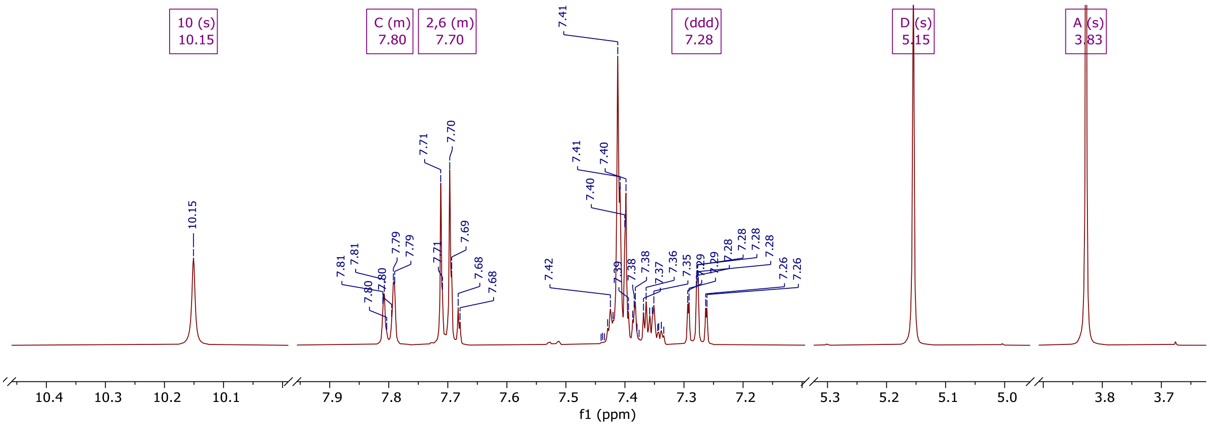

^{13}C NMR (126 MHz, DMSO-d_6)

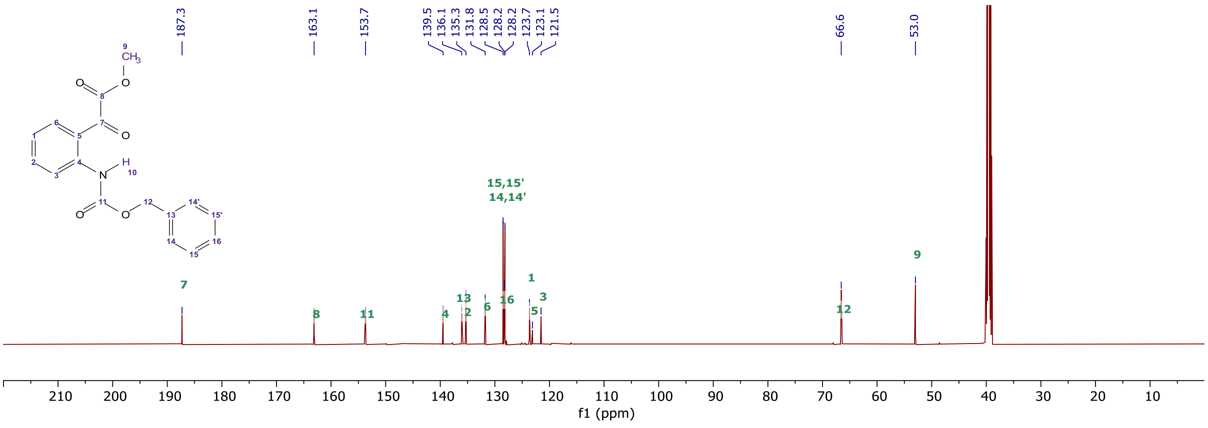

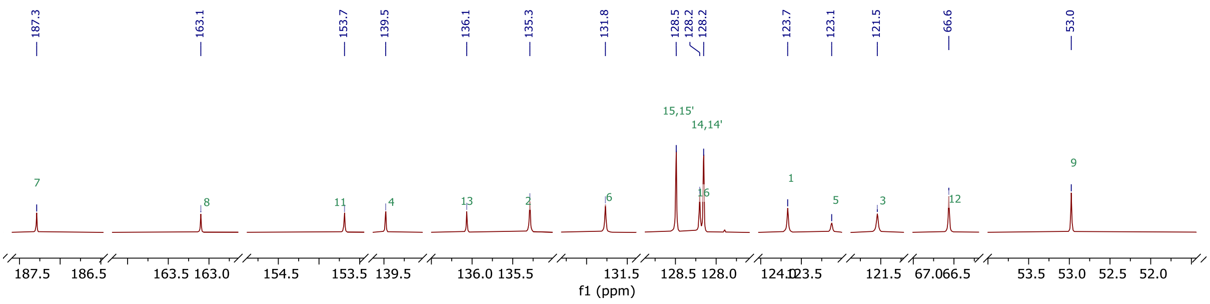

$^1H/^1H$ COSY

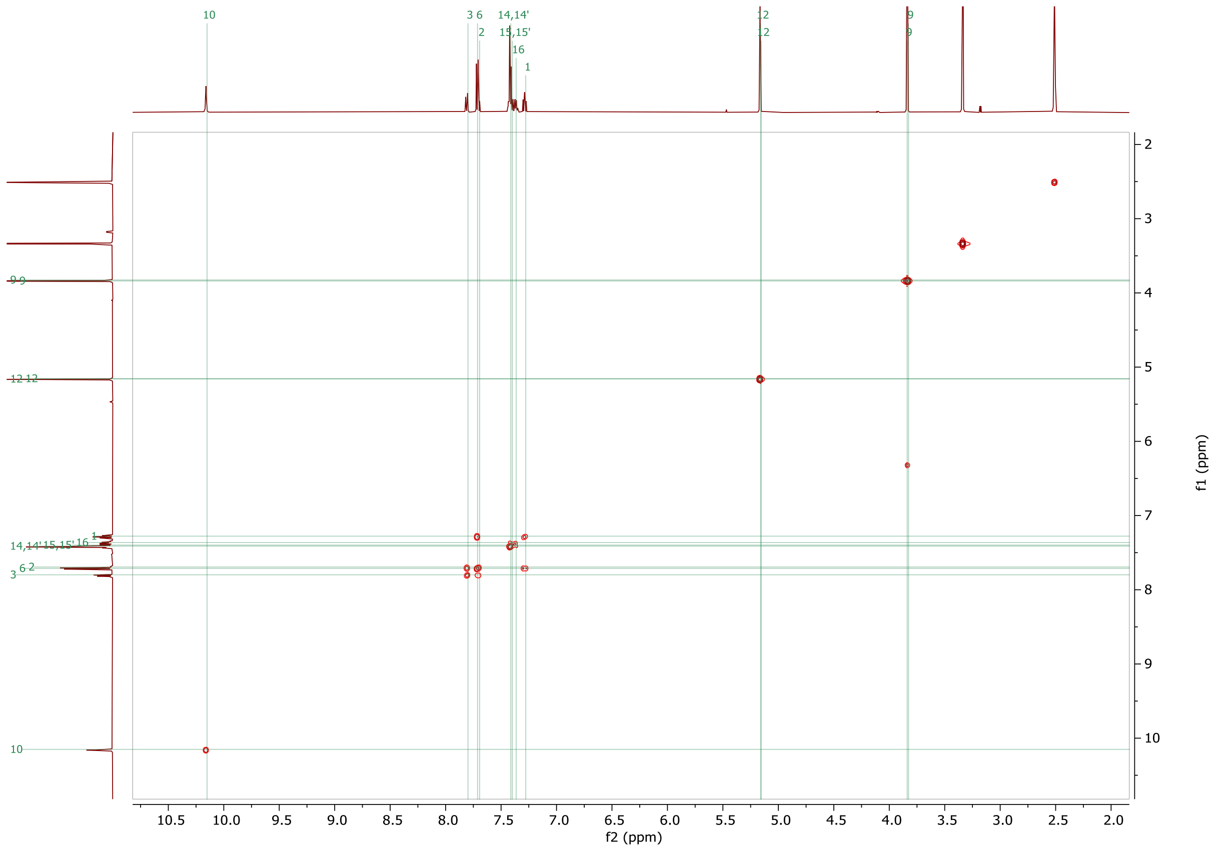

$^1H/^{13}C$ HSQC

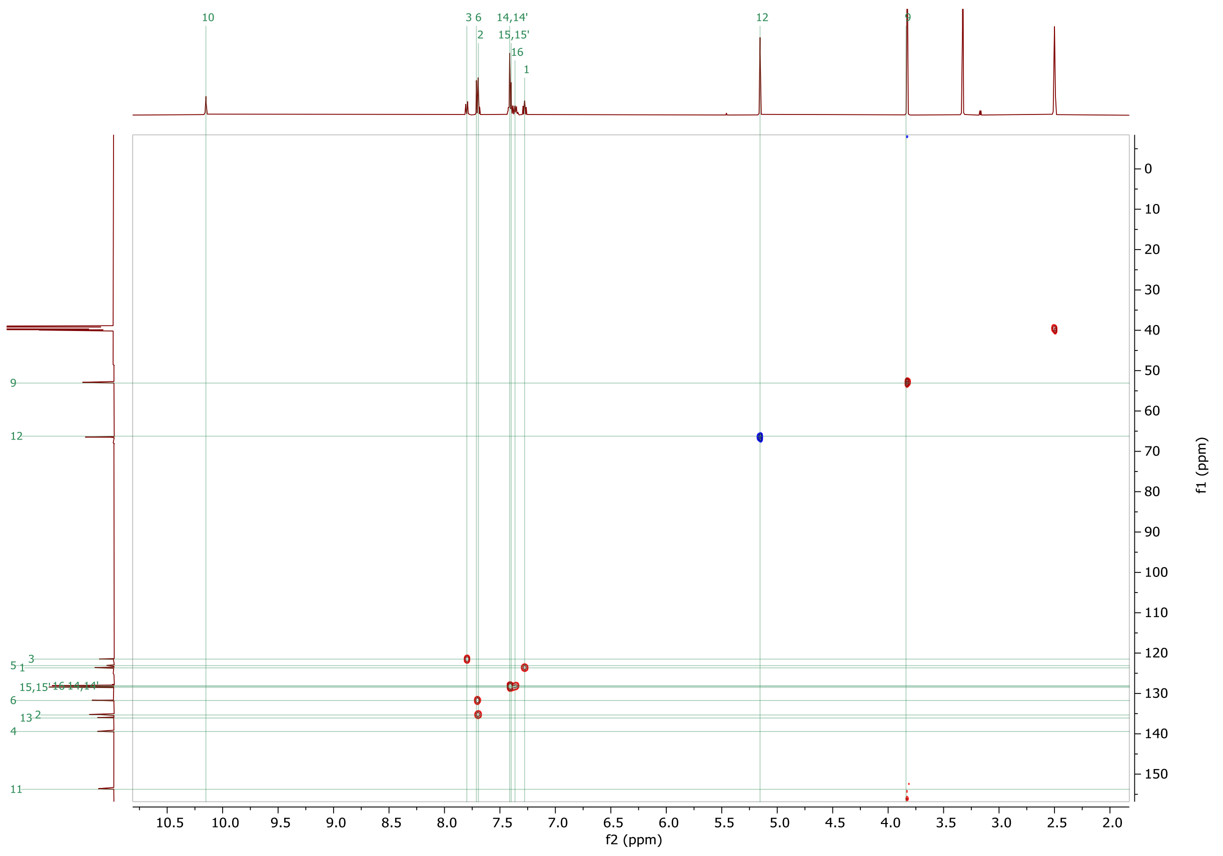

$^{1}H/^{13}C$ HMBC

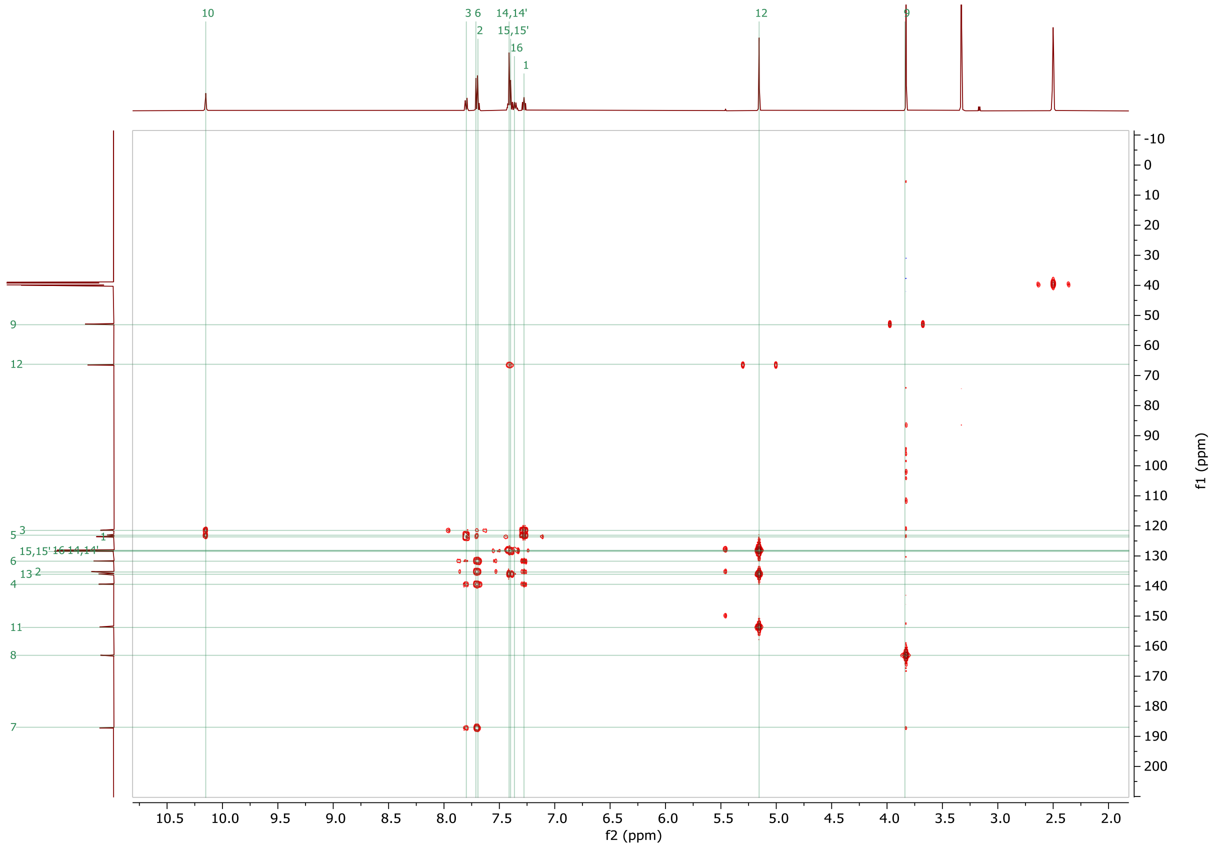

10
3 6
2
14,14'
15,15'
16
1
12
9
5
3
1
15,15' 16 14,14'
6
13 2
4
11
8
7
f2 (ppm)
f1 (ppm)

Methyl 2,3-dioxoindoline-1-carboxylate S2

^{1}H NMR (500 MHz, DMSO-d_6)

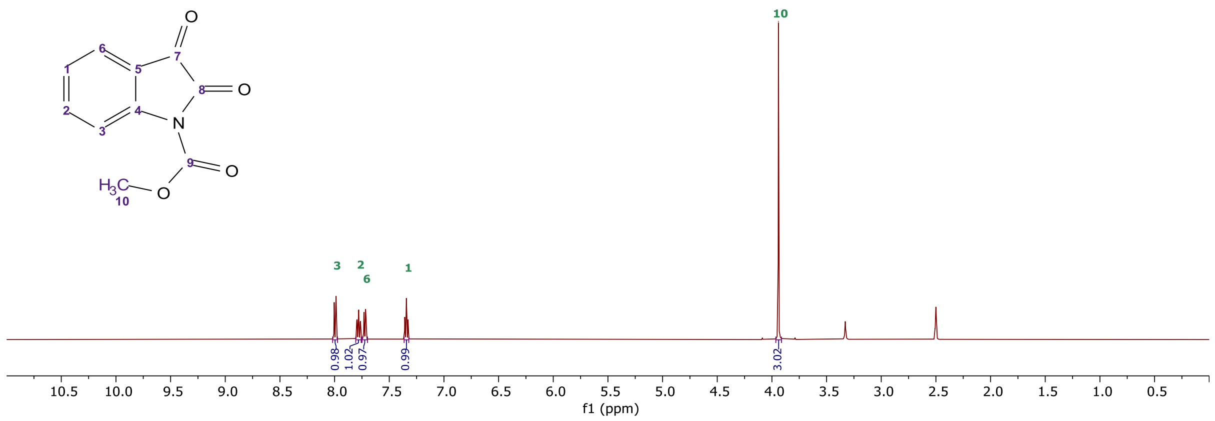

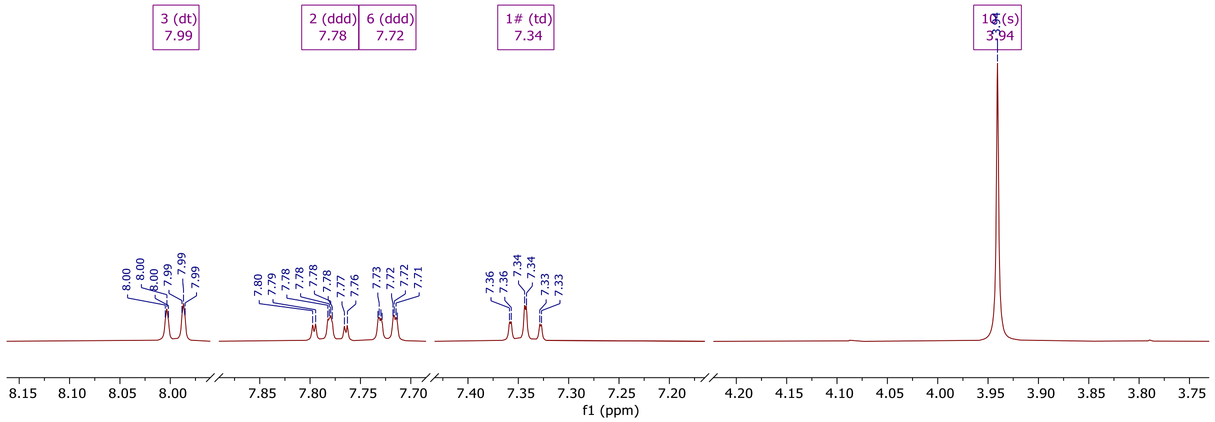

^{13}C NMR (126 MHz, DMSO-d_6)

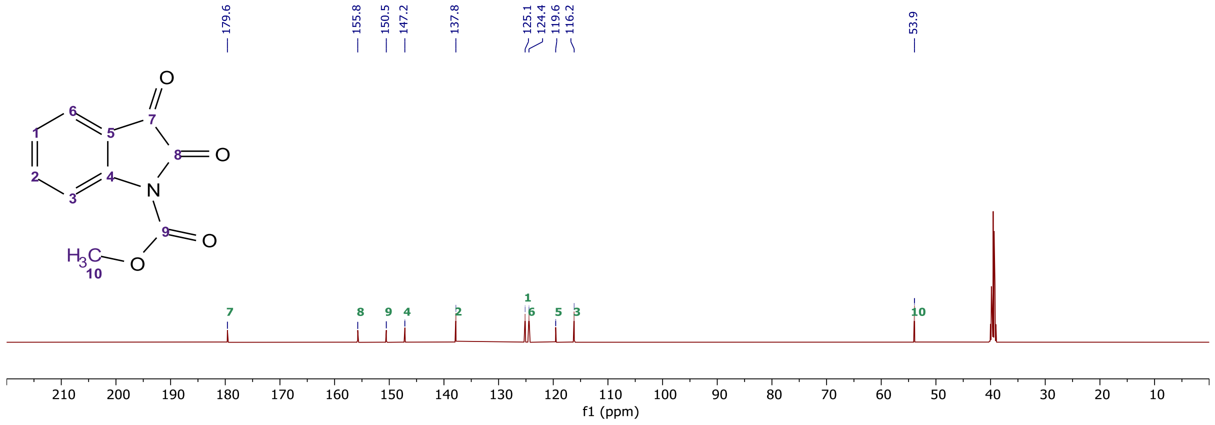

$^1H/^1H$ COSY

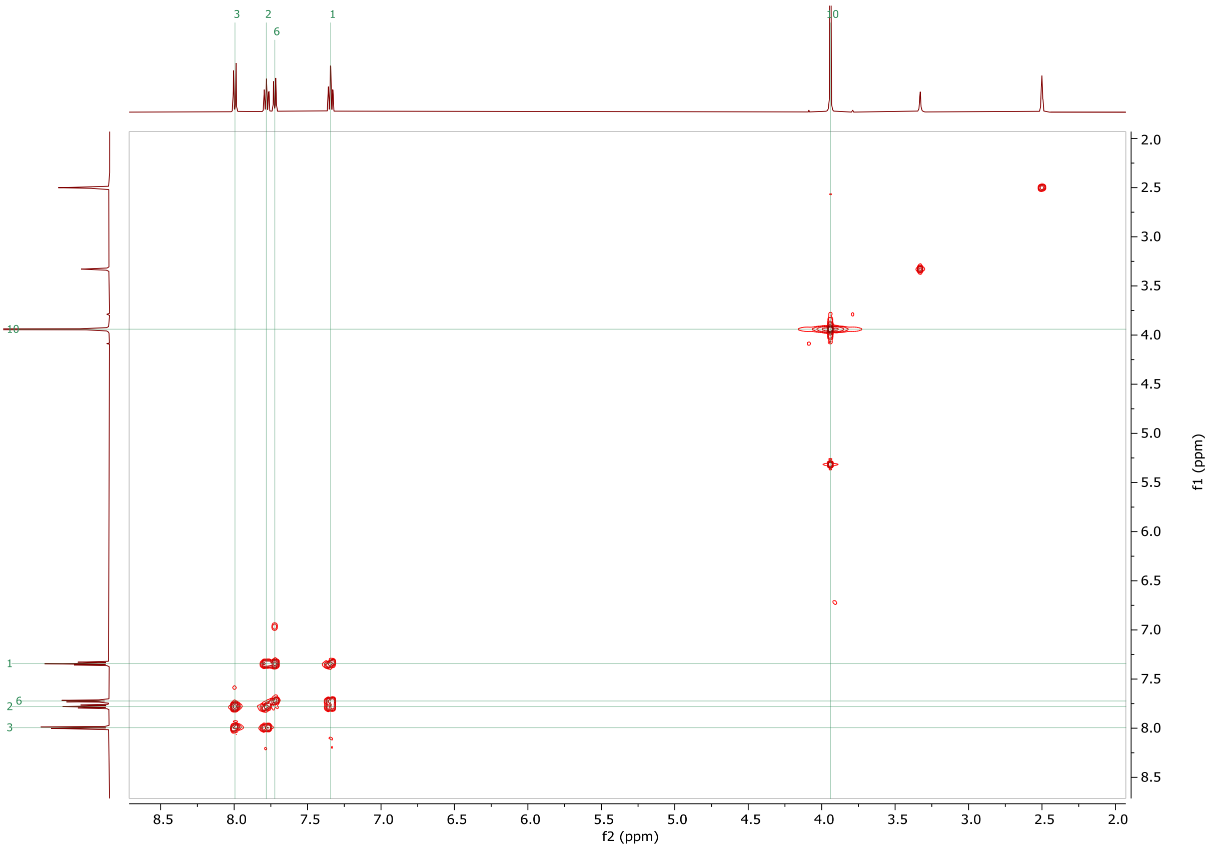

$^1H/^{13}C$ HSQC

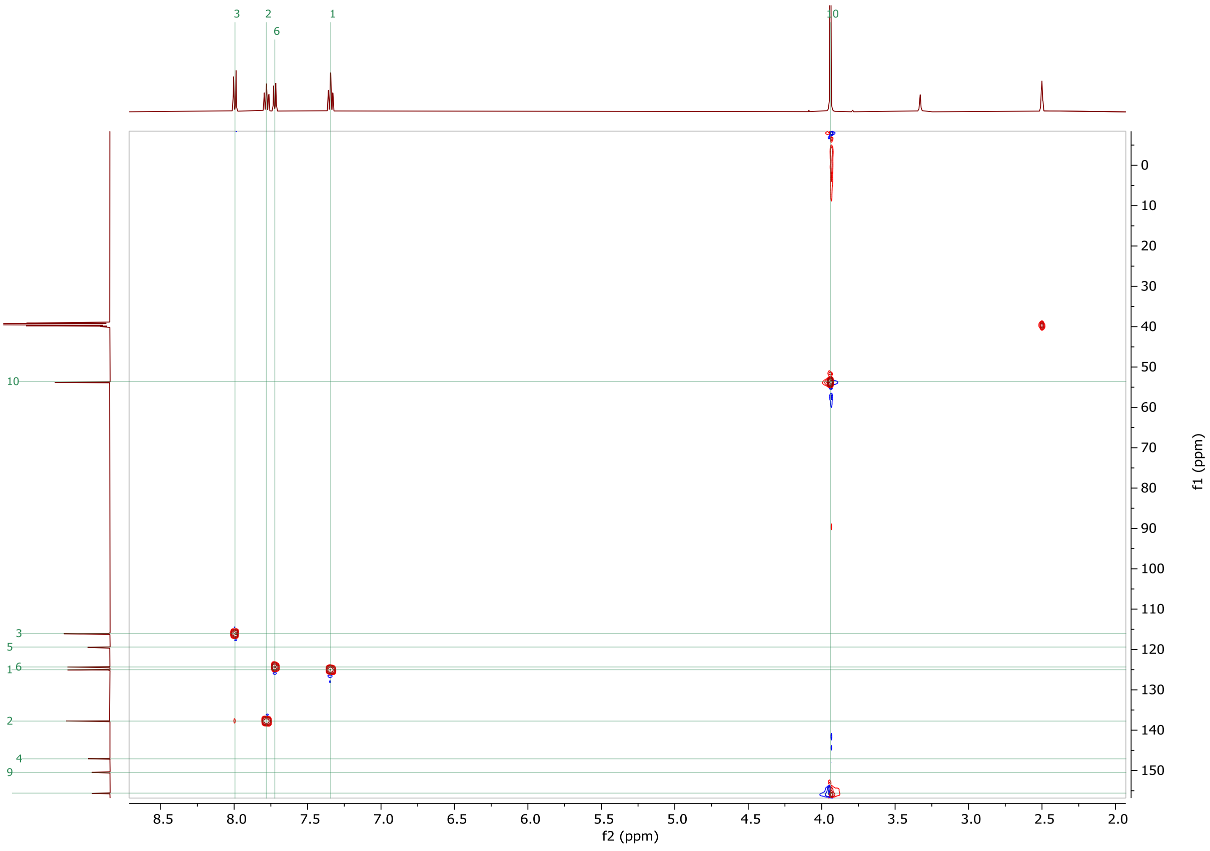

$^1H/^{13}C$ HMBC

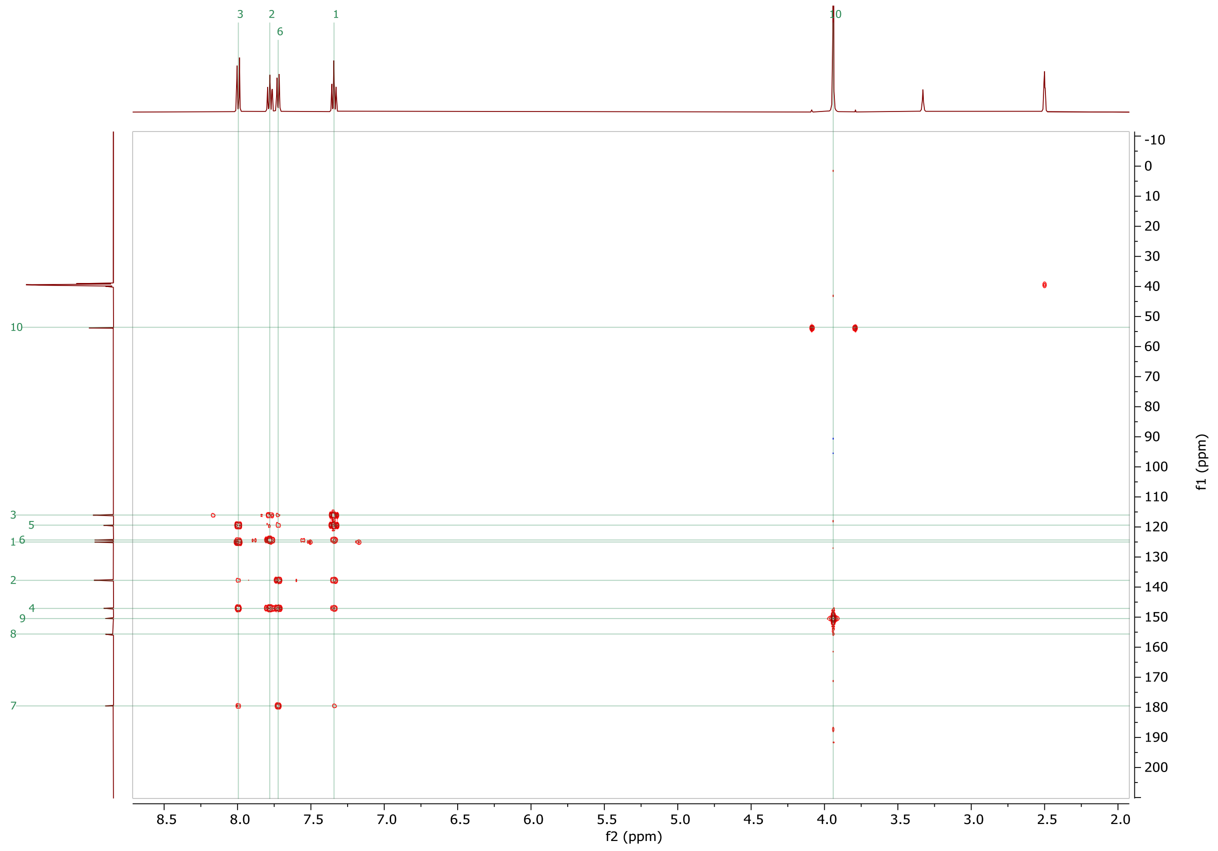

Isopropyl 2-(2-((methoxycarbonyl)amino)phenyl)-2-oxoacetate 15c

^{1}H NMR (500 MHz, $CDCl_3$)

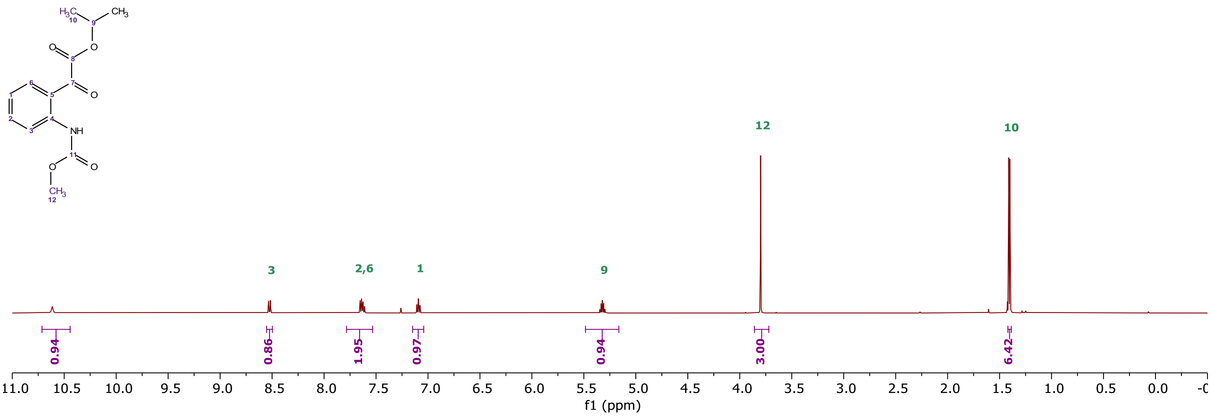

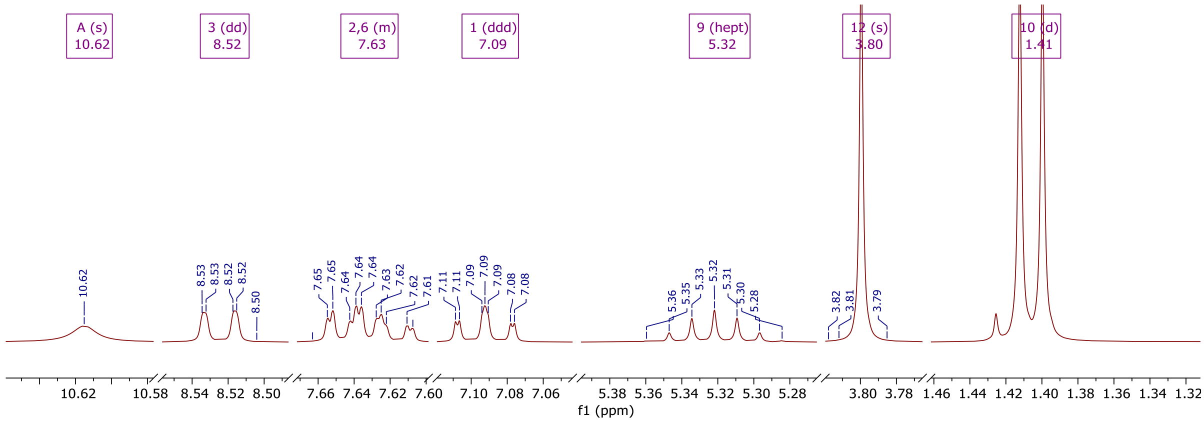

^{13}C NMR (126 MHz, $CDCl_3$)

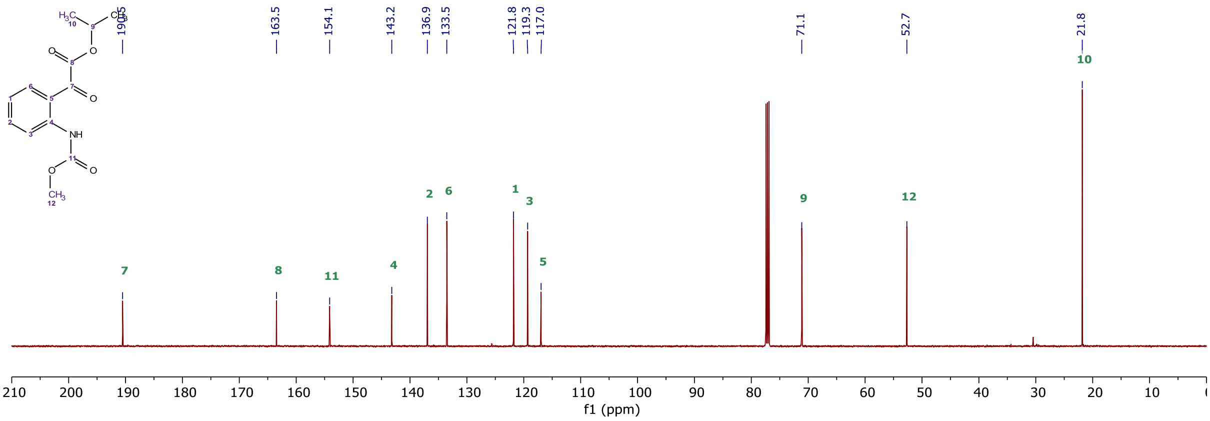

$^{1}H/^{1}H$ COSY

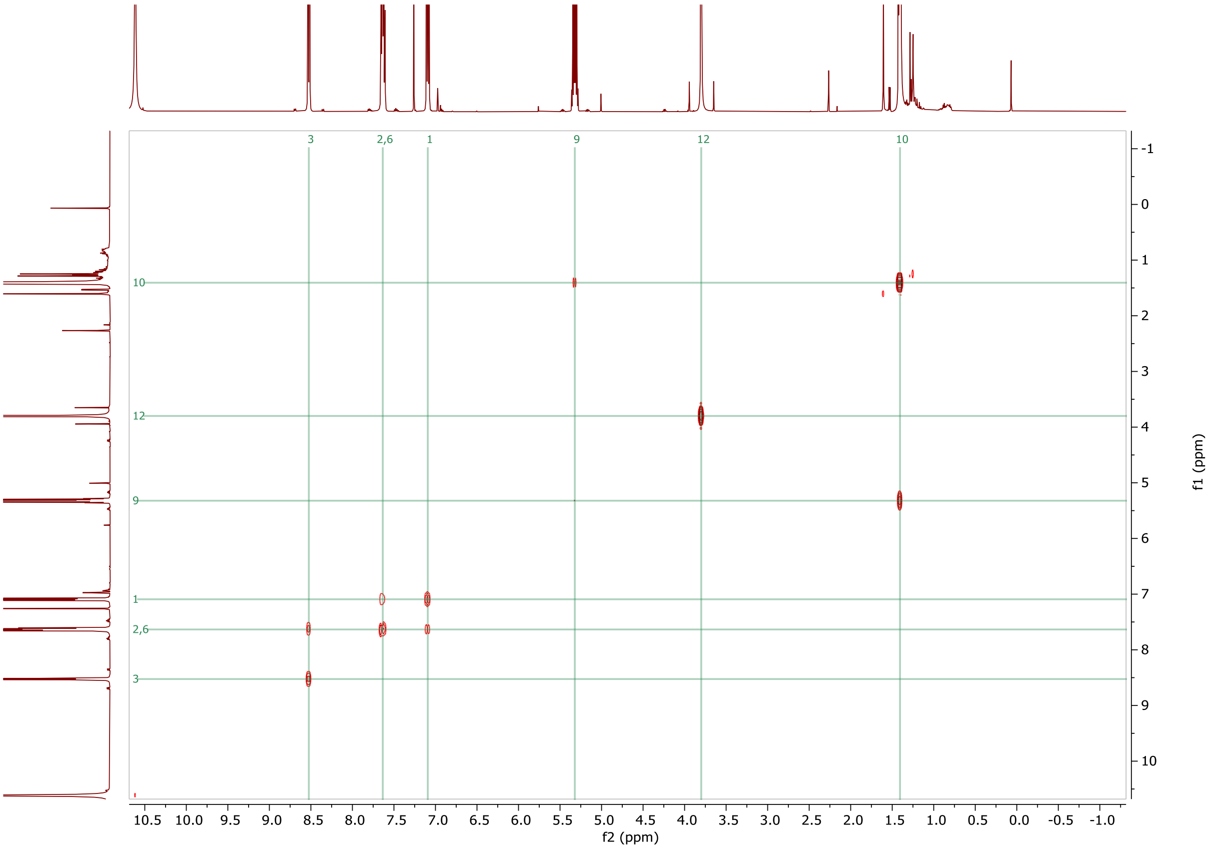

$^{1}H/^{13}C$ HSQC

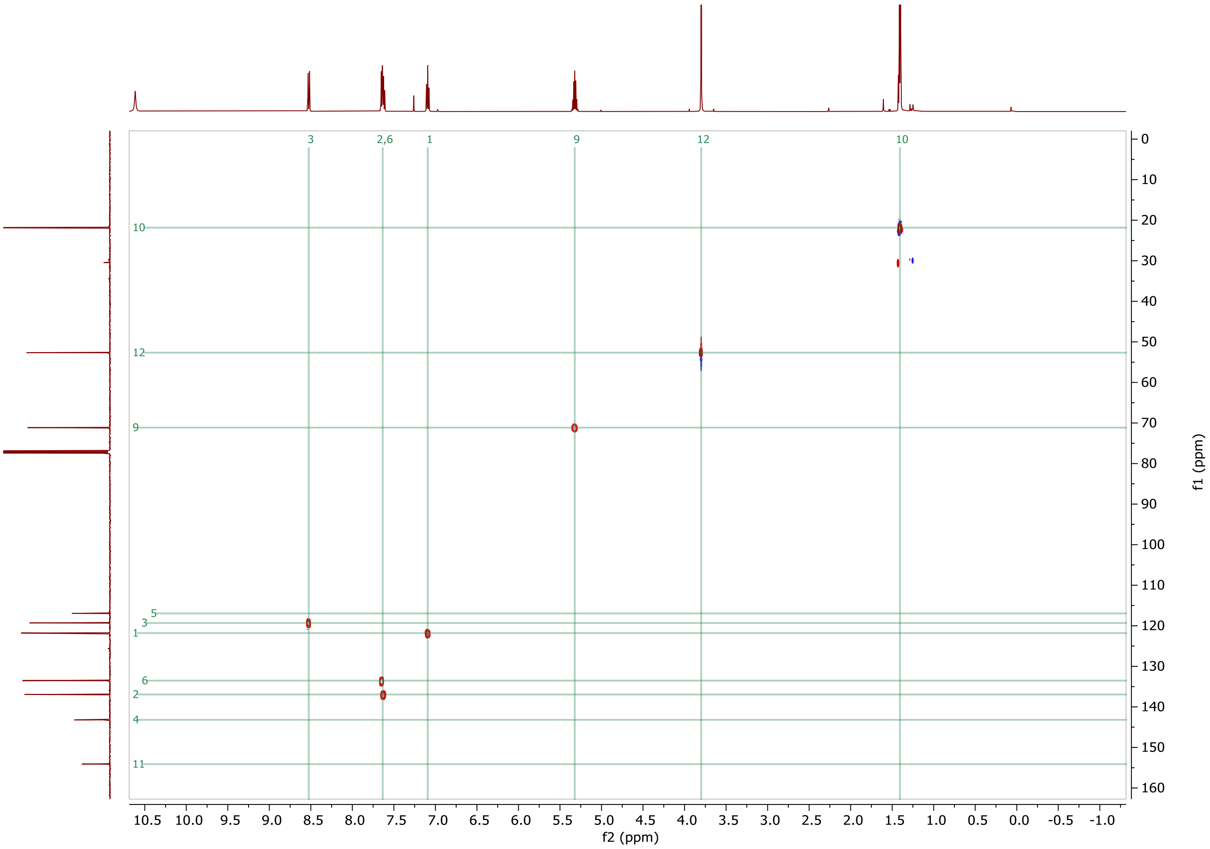

$^{1}H/^{13}C$ HMBC

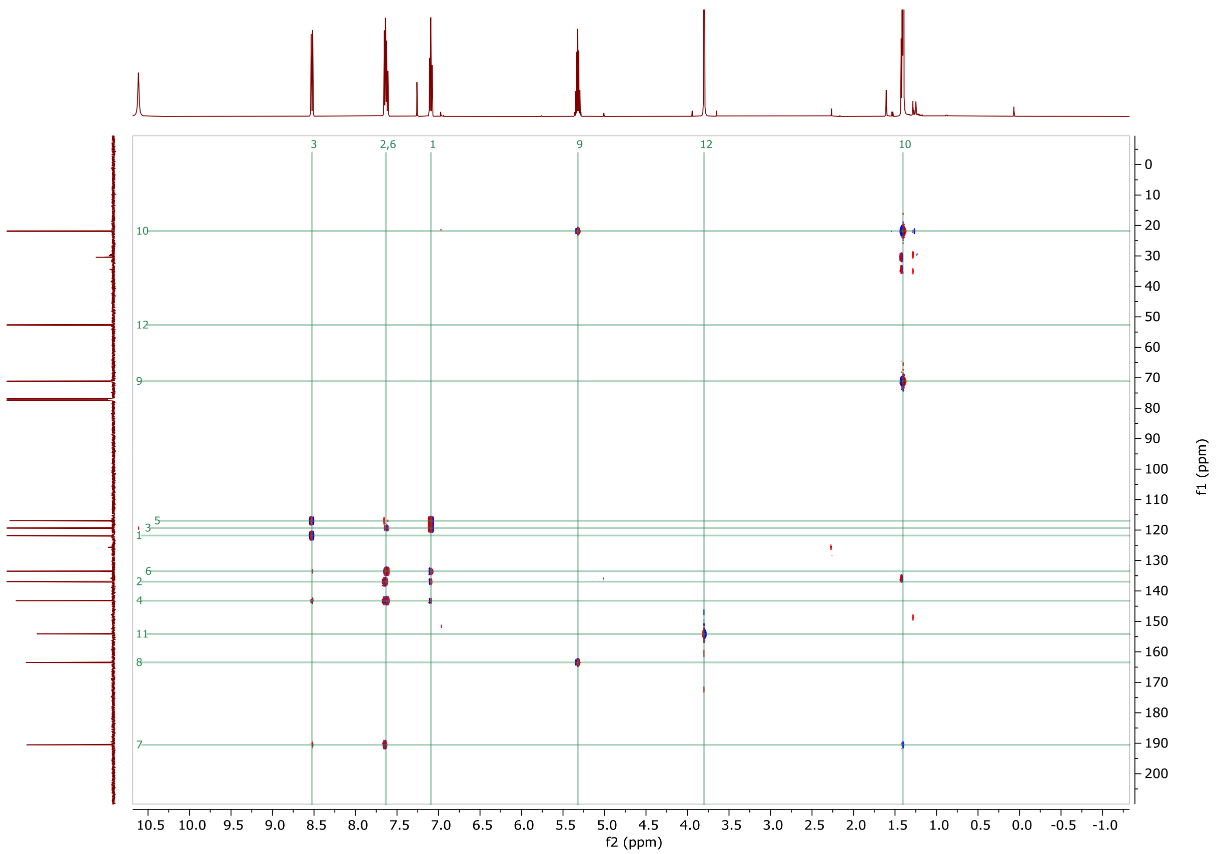

Isopropyl 2-(2-((*tert*-butoxycarbonyl)amino)phenyl)-2-oxoacetate 15d

^{1}H NMR (500 MHz, $CDCl_3$)

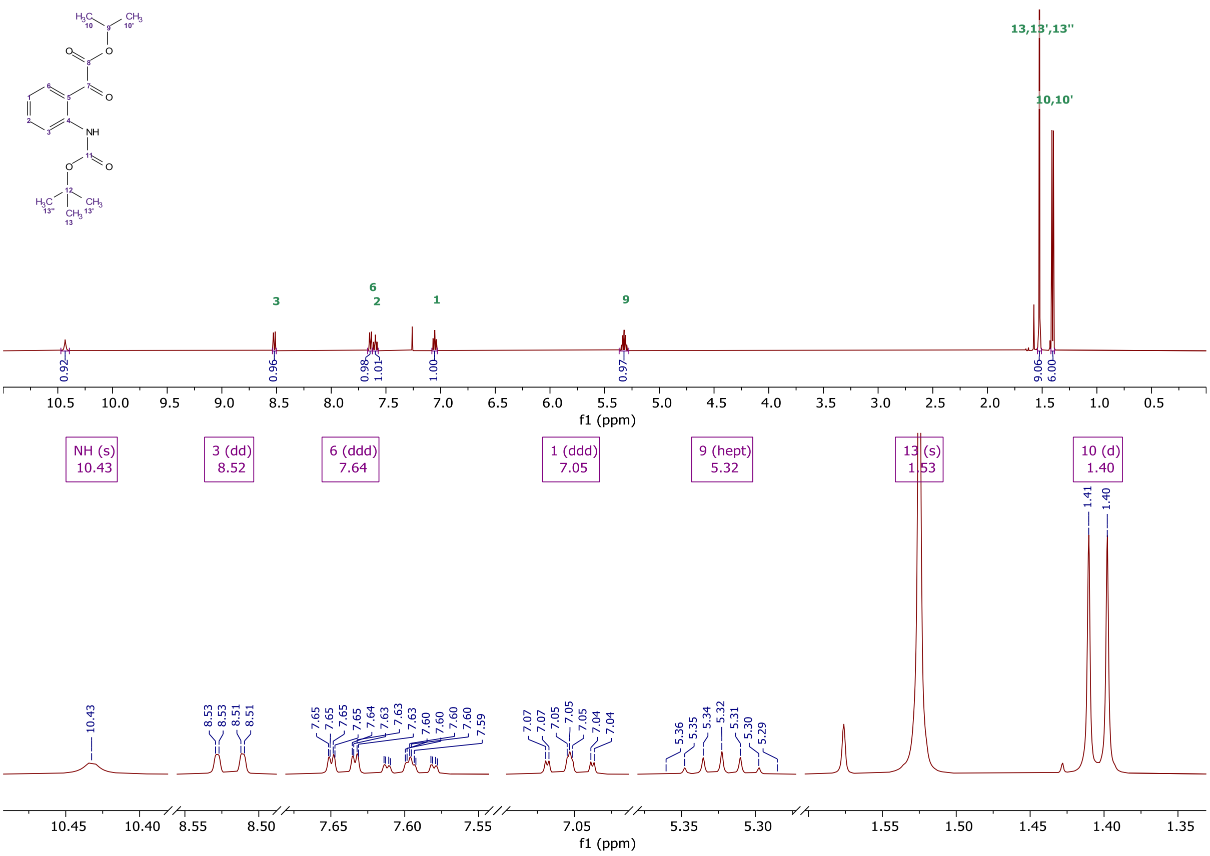

^{13}C NMR (126 MHz, $CDCl_3$)

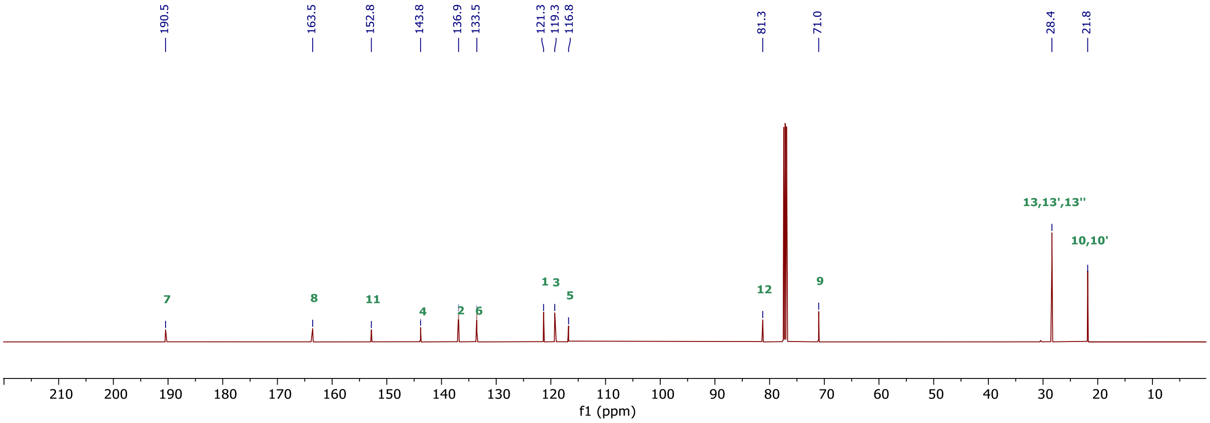

$^1H/^1H$ COSY

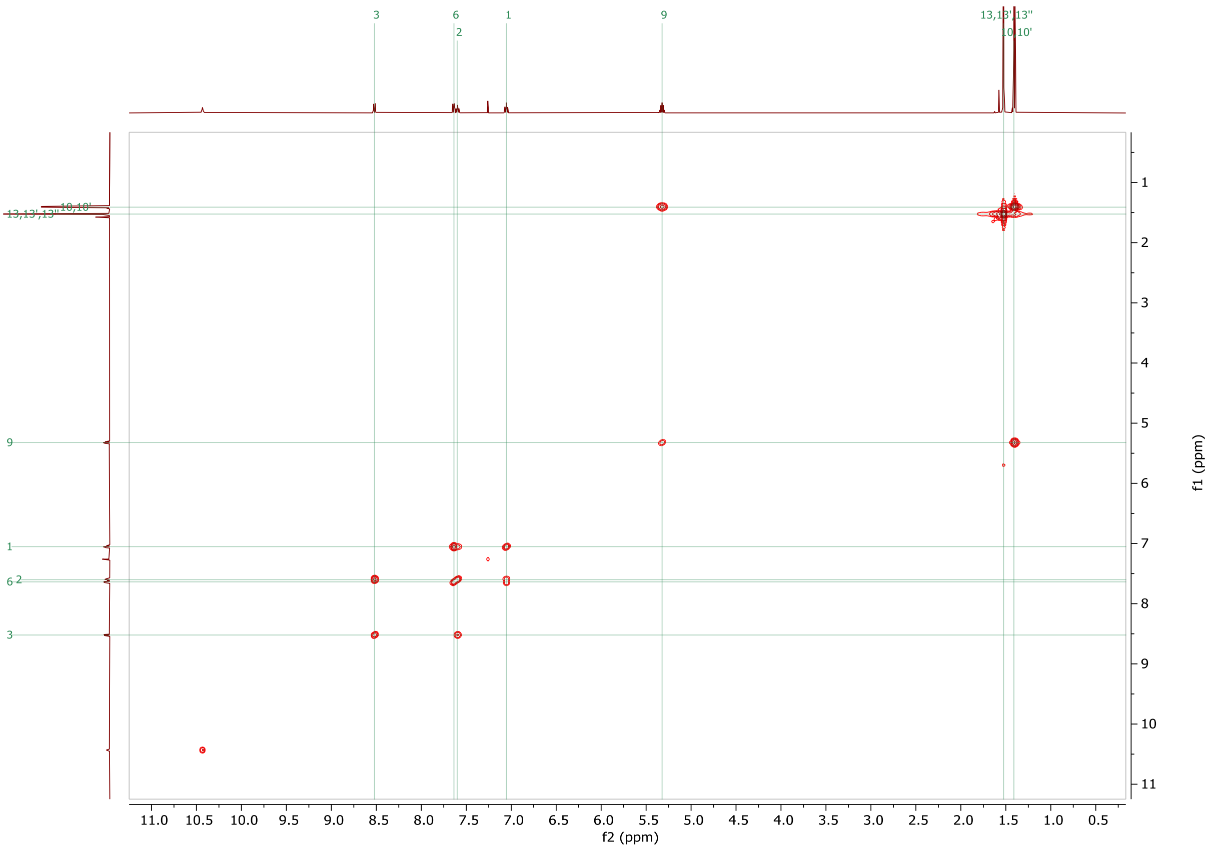

$^1H/^{13}C$ HSQC

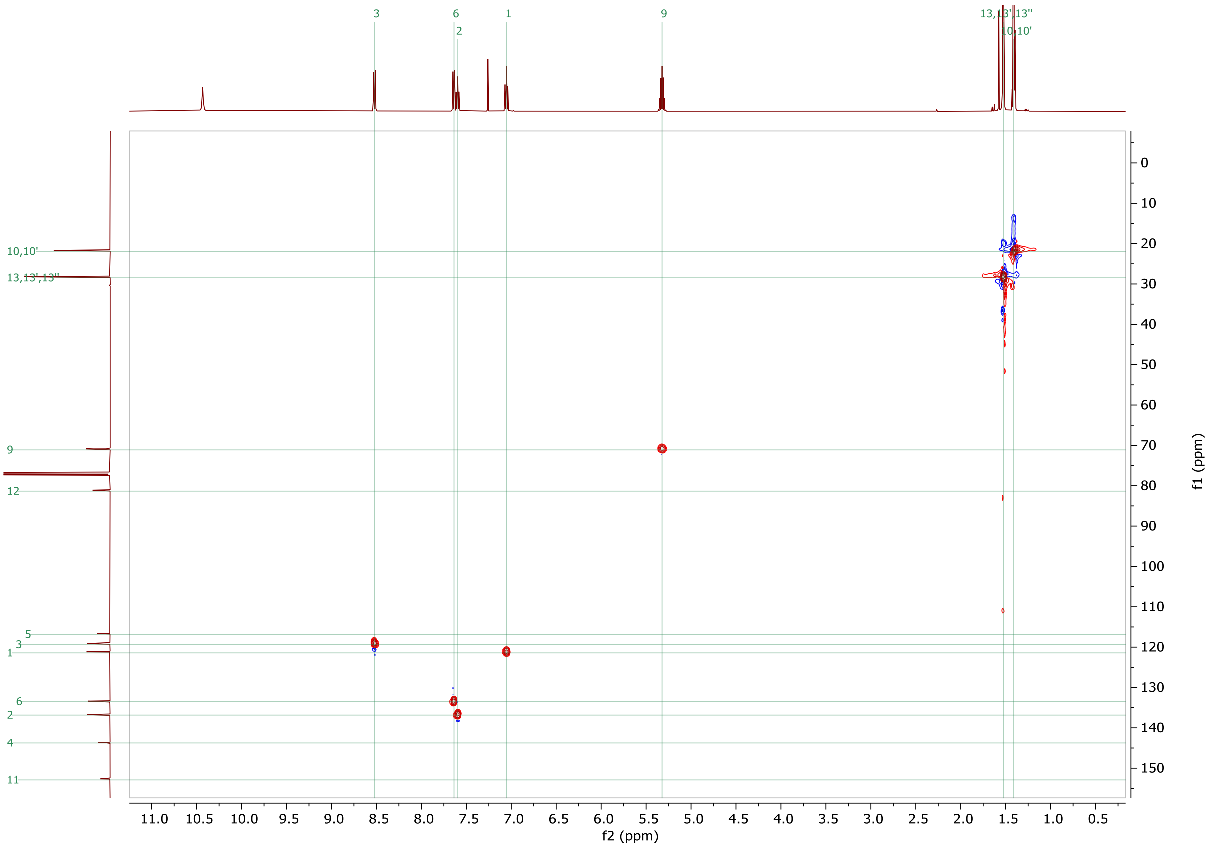

$^1H/^{13}C$ HMBC

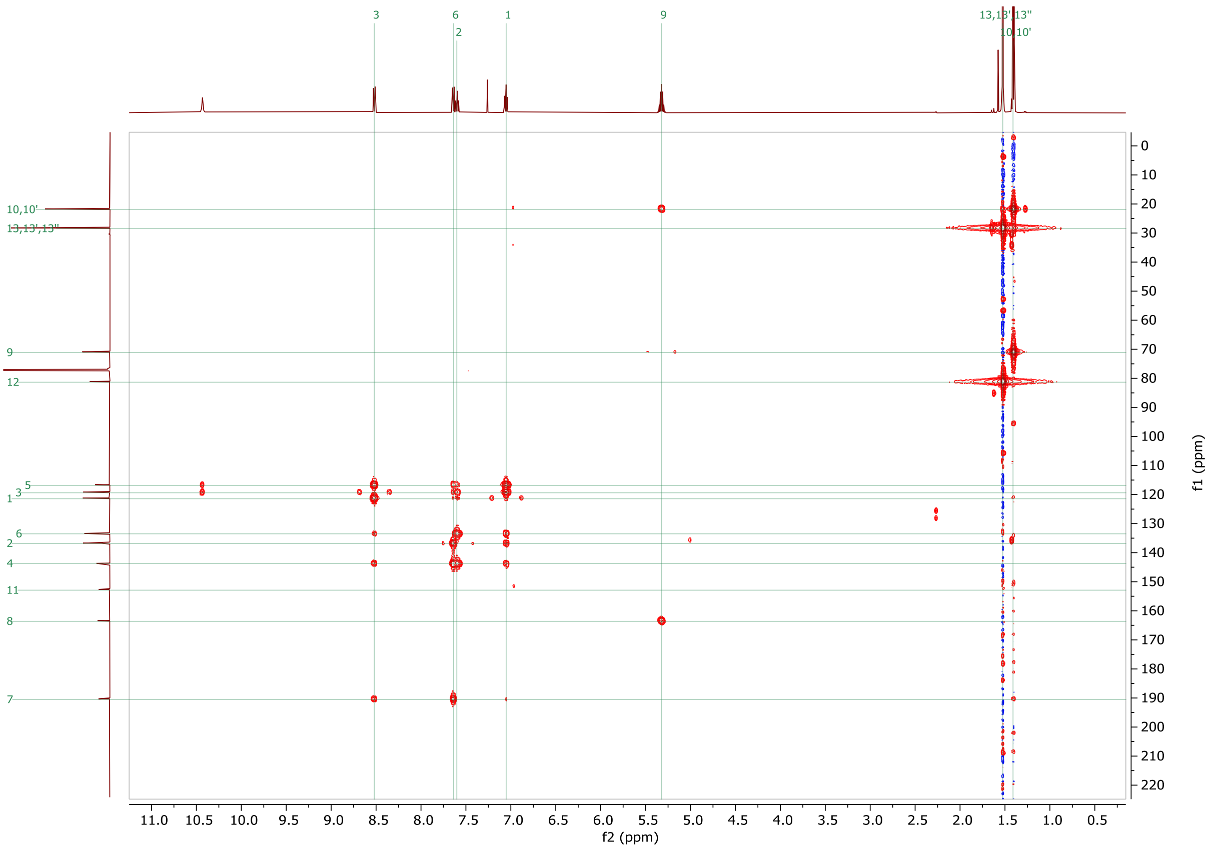

Benzyl 5-chloro-2,3-dioxoindoline-1-carboxylate S3

^{1}H NMR (500 MHz, DMSO-d_6)

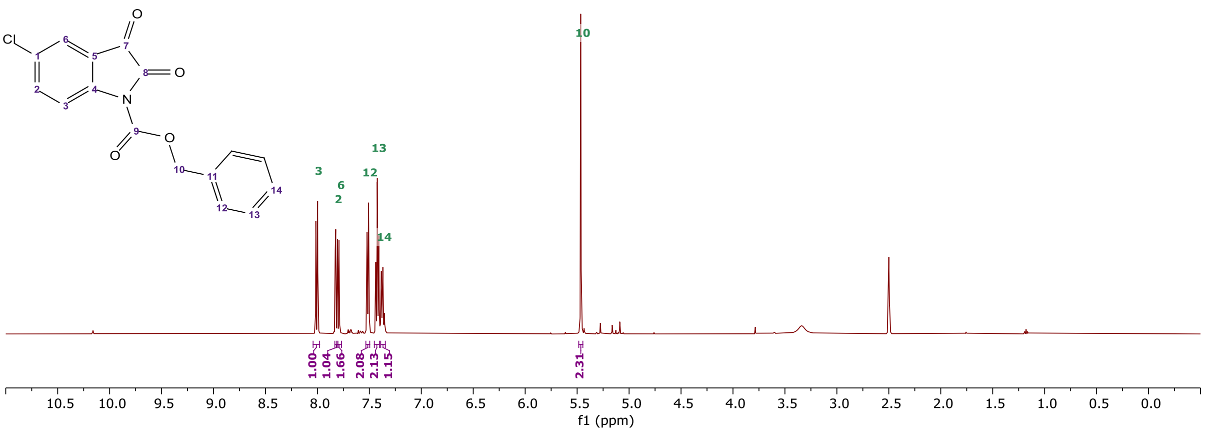

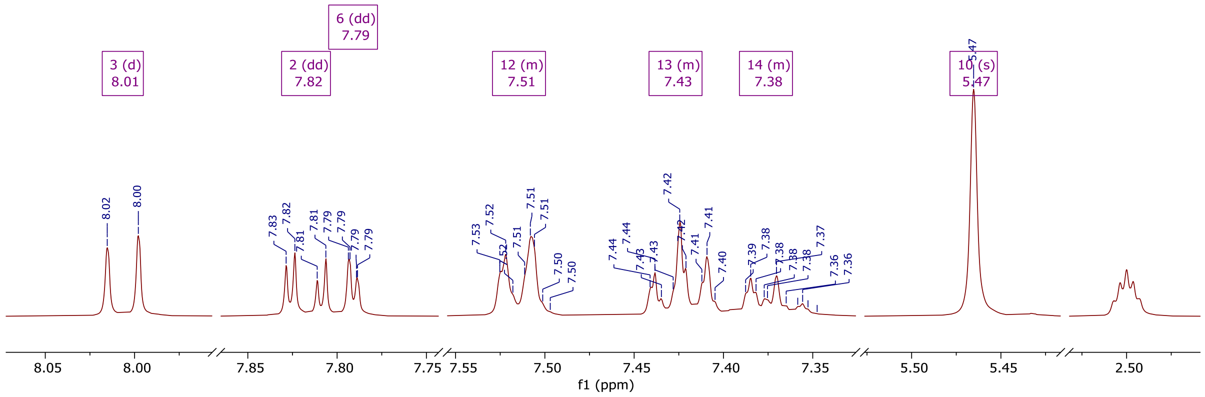

^{13}C NMR (126 MHz, DMSO-d_6)

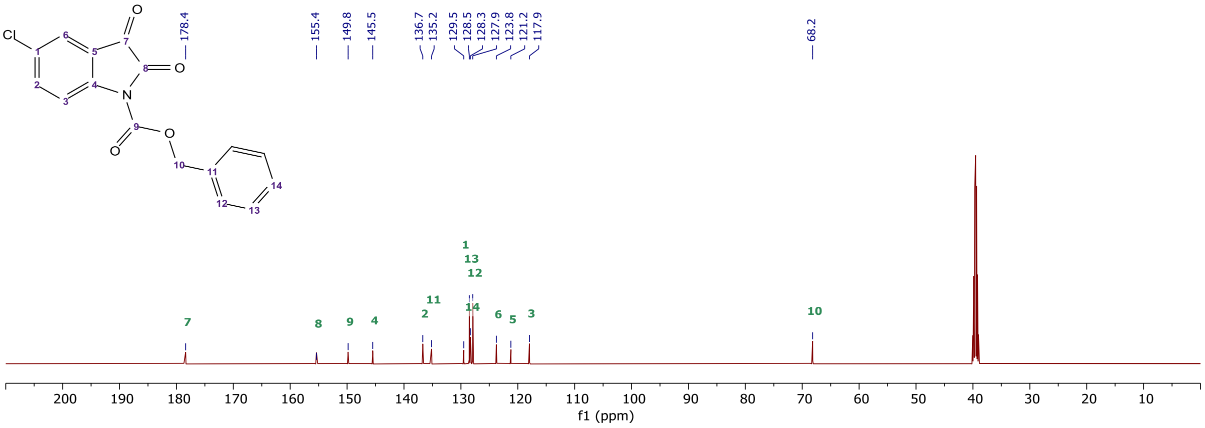

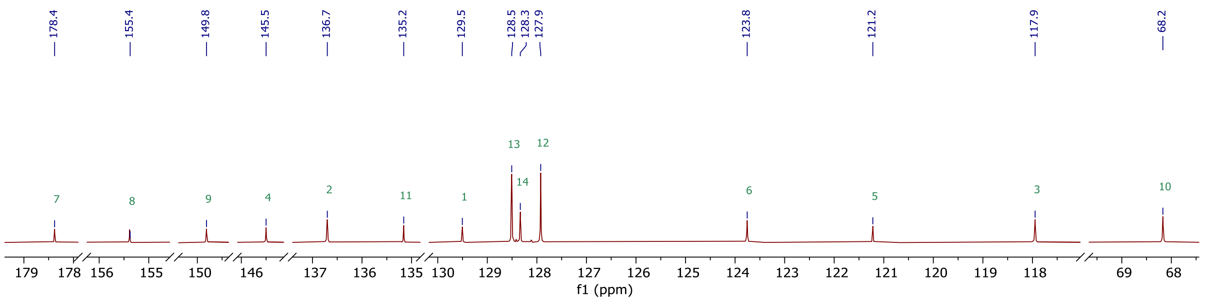

$^1H/^1H$ COSY

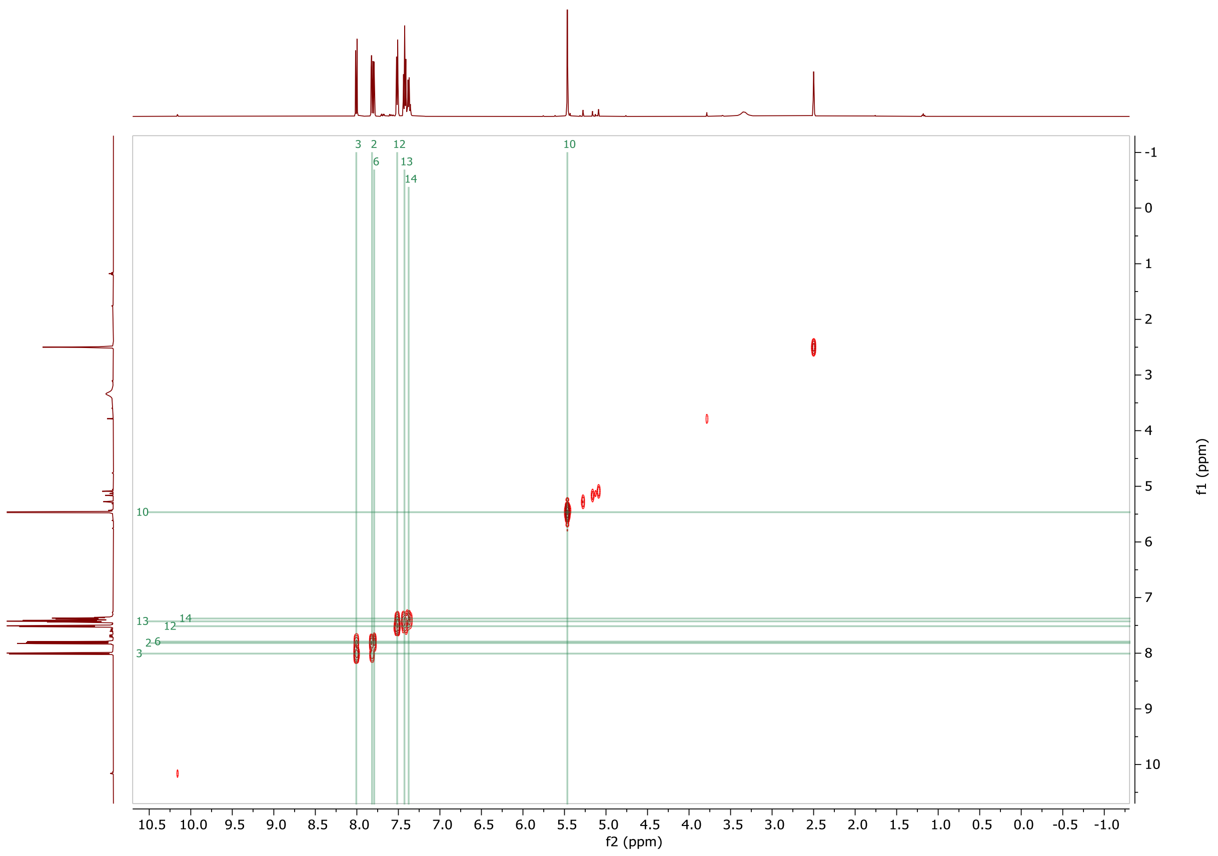

$^1H/^{13}C$ HSQC

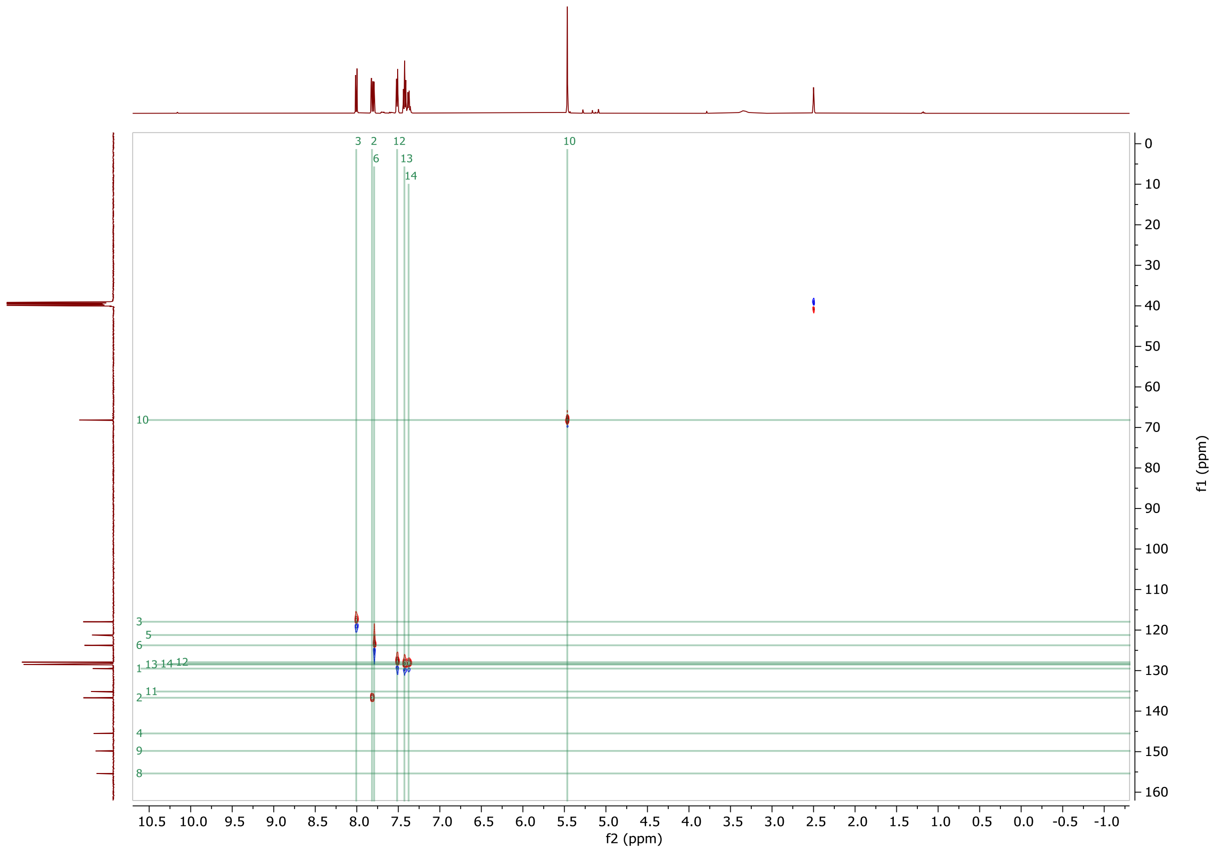

$^{1}H/^{13}C$ HMBC

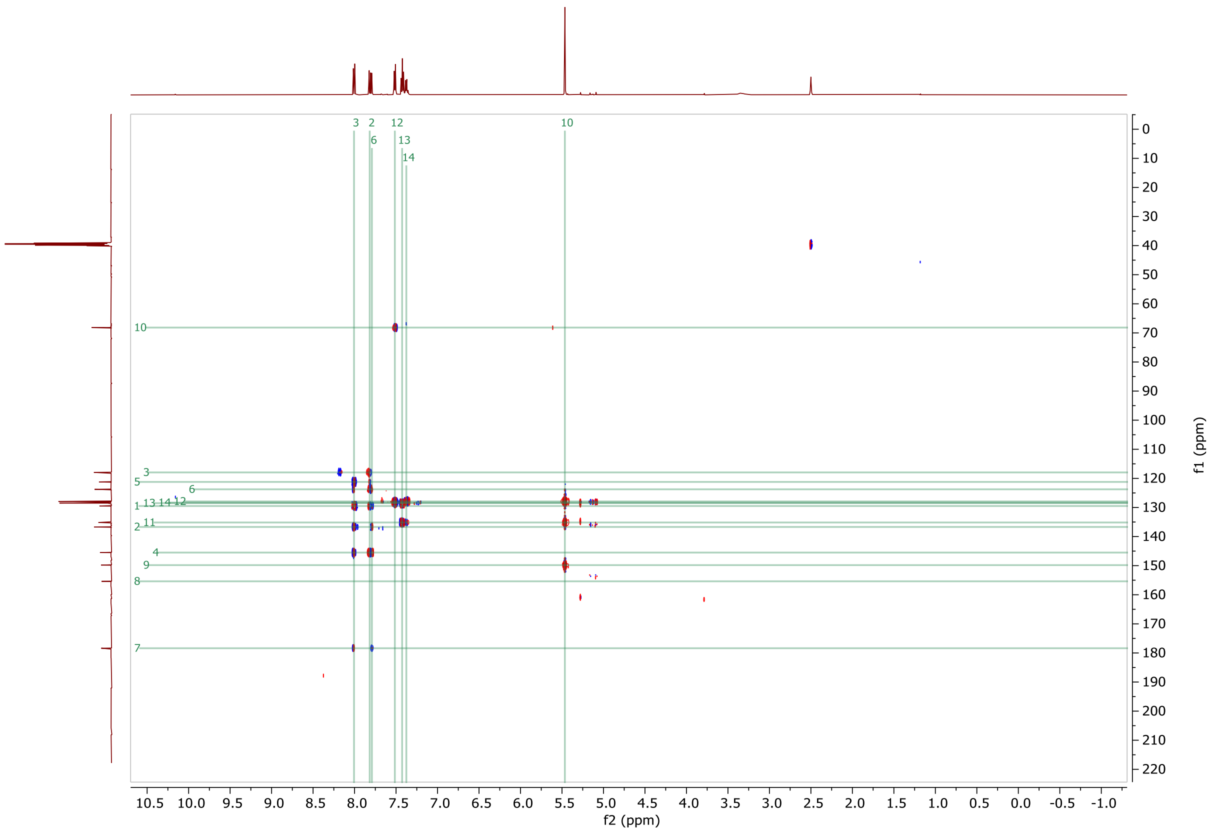

Isopropyl 2-(2-(((benzyloxy)carbonyl)amino)-5-chlorophenyl)-2-oxoacetate 15e

^{1}H NMR (500 MHz, $CDCl_3$)

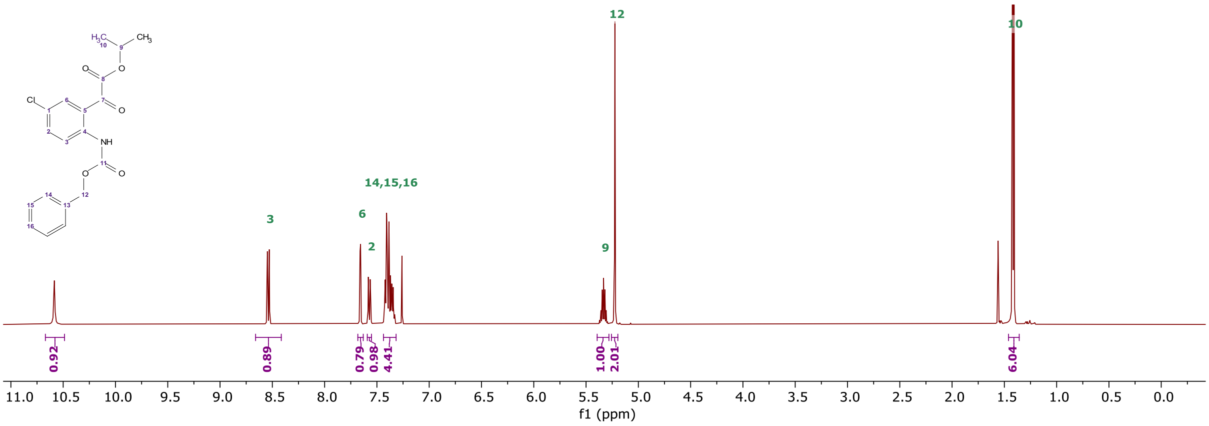

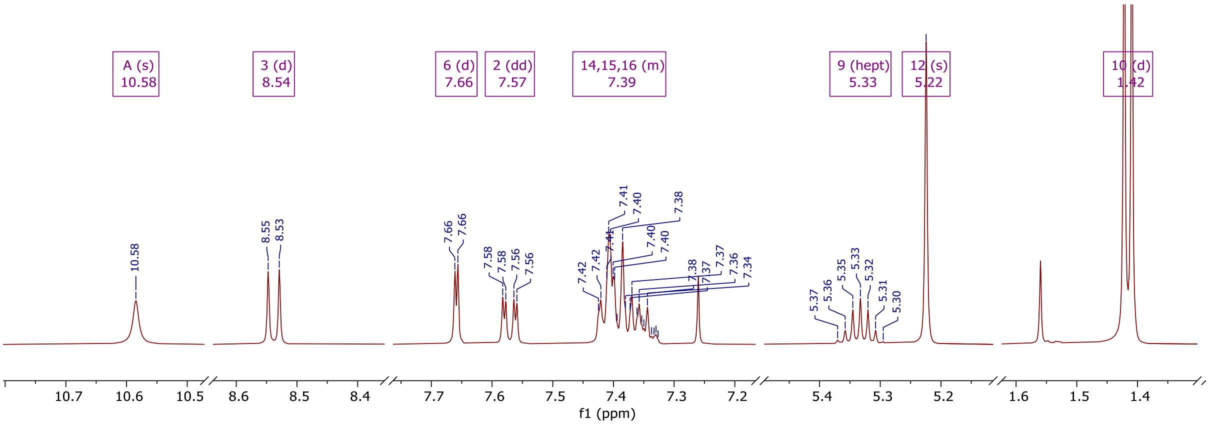

^{13}C NMR (126 MHz, $CDCl_3$)

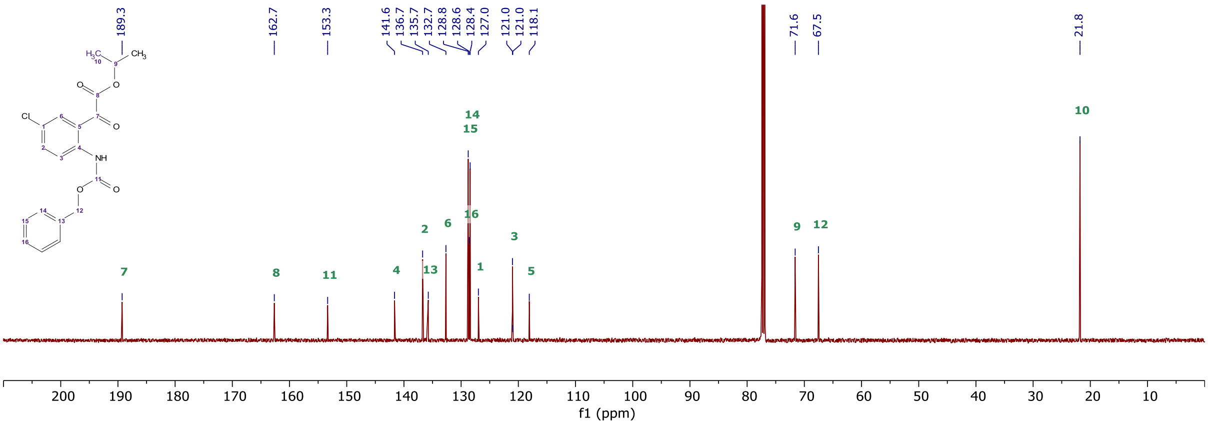

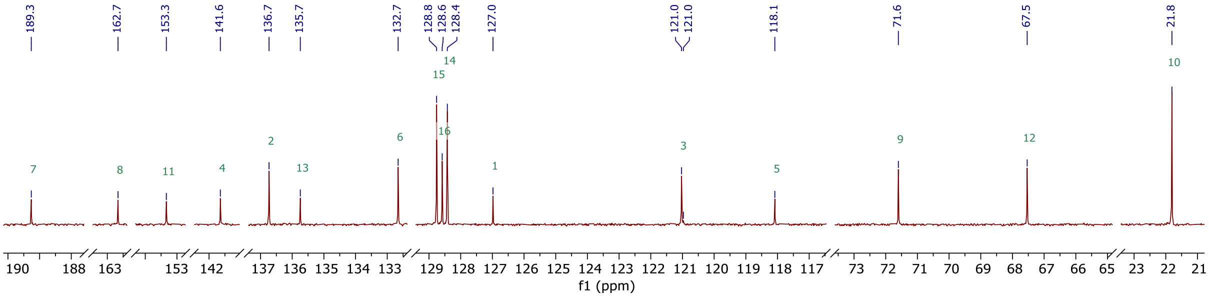

$^{1}H/^{1}H$ COSY

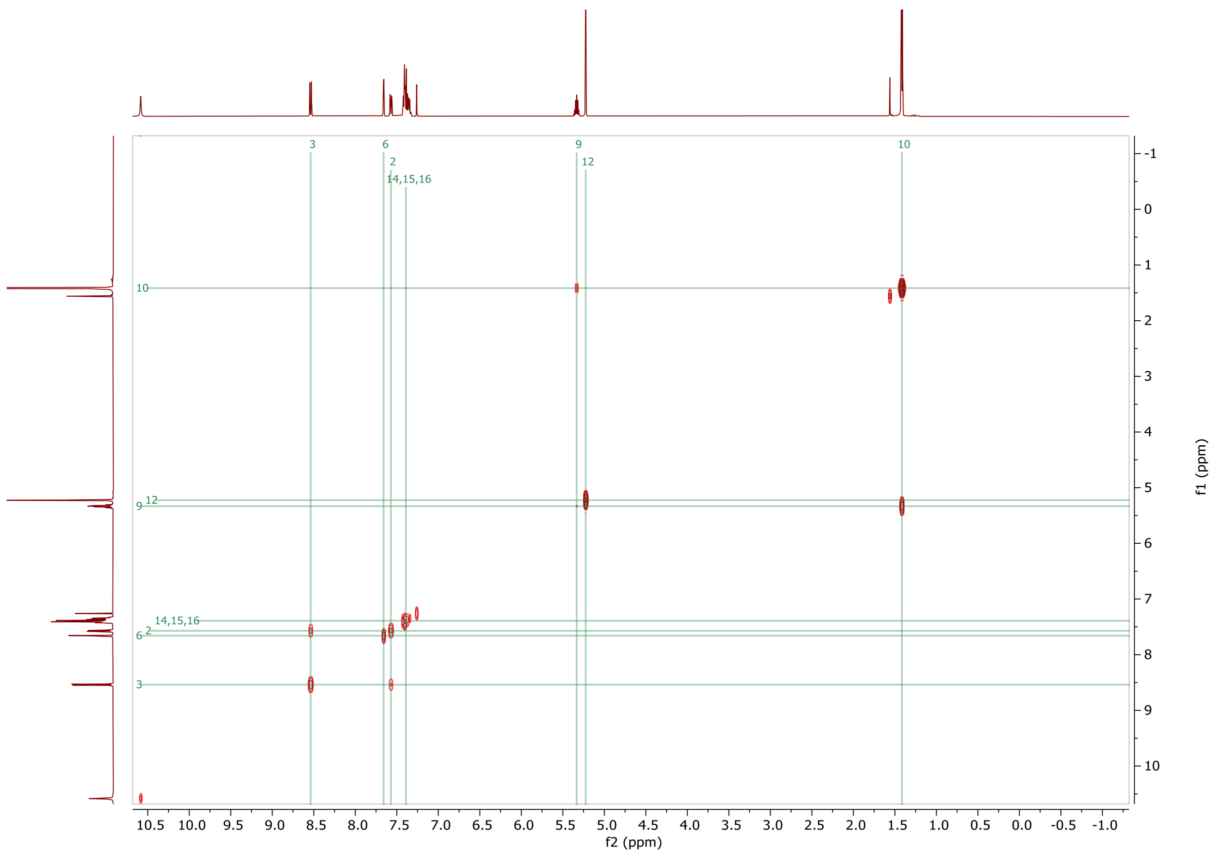

$^{1}H/^{13}C$ HSQC

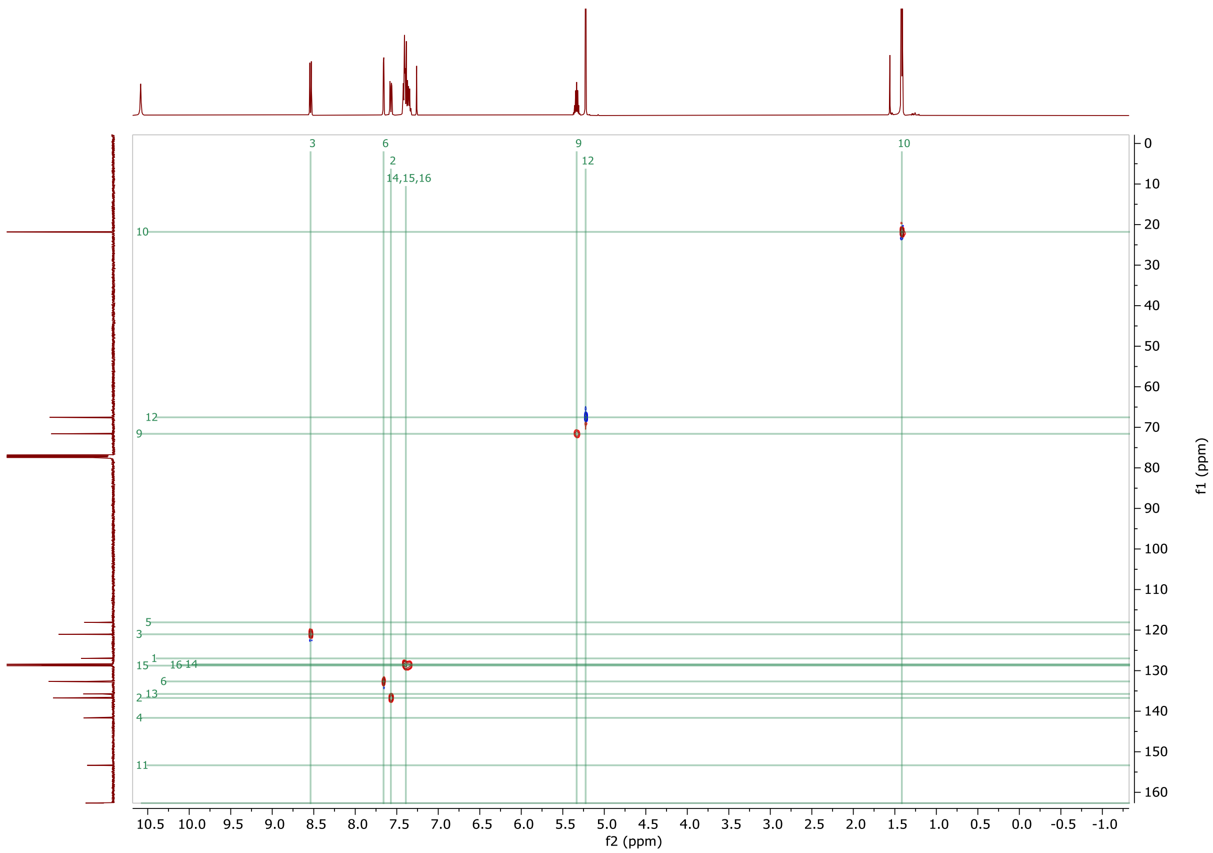

$^{1}H/^{13}C$ HMBC

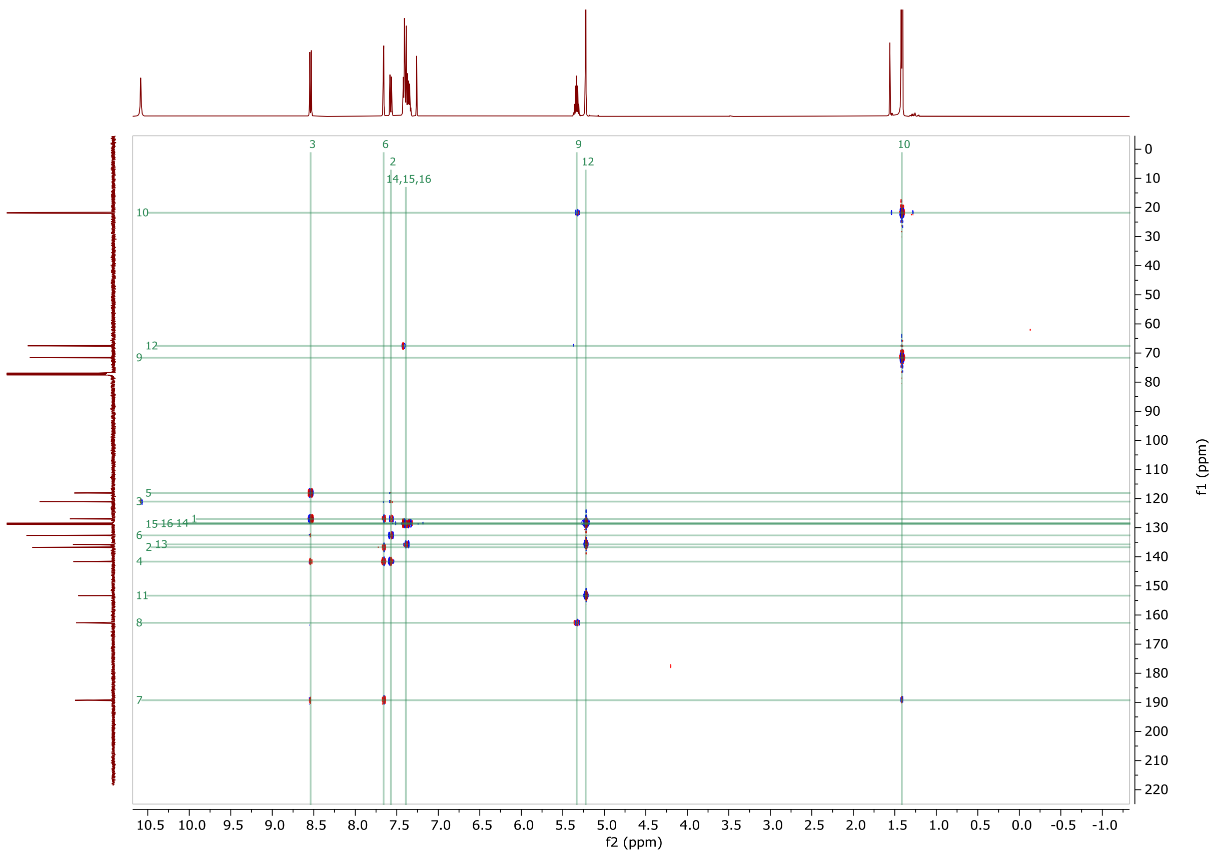
3
6
2
14,15,16
9
12
10
10
12
9
5
3
15 16 14 1
6
2 13
4
11
8
7
10.5 10.0 9.5 9.0 8.5 8.0 7.5 7.0 6.5 6.0 5.5 5.0 4.5 4.0 3.5 3.0 2.5 2.0 1.5 1.0 0.5 0.0 -0.5 -1.0
f2 (ppm)
0 10 20 30 40 50 60 70 80 90 100 110 120 130 140 150 160 170 180 190 200 210 220
f1 (ppm)

Benzyl 5-methyl-2,3-dioxoindoline-1-carboxylate S4

^{1}H NMR (500 MHz, DMSO-d_6)

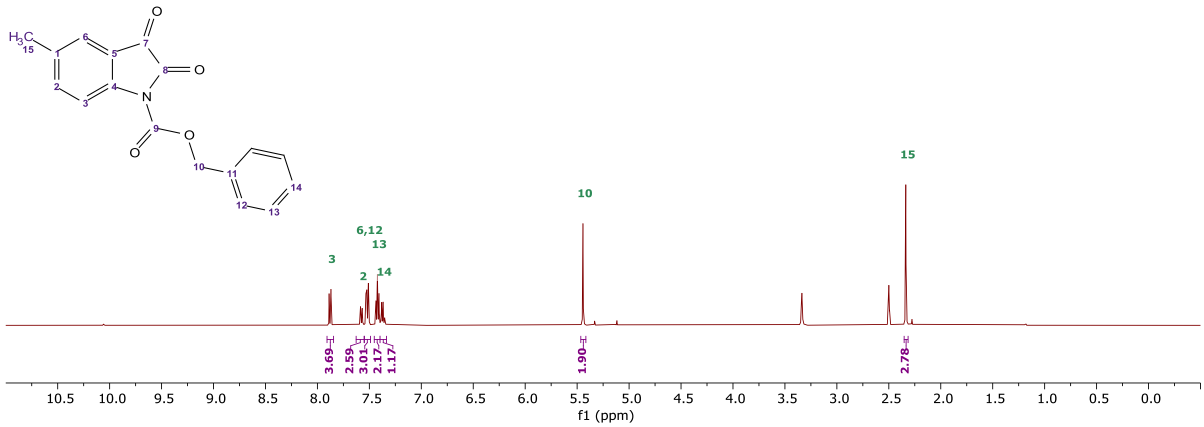

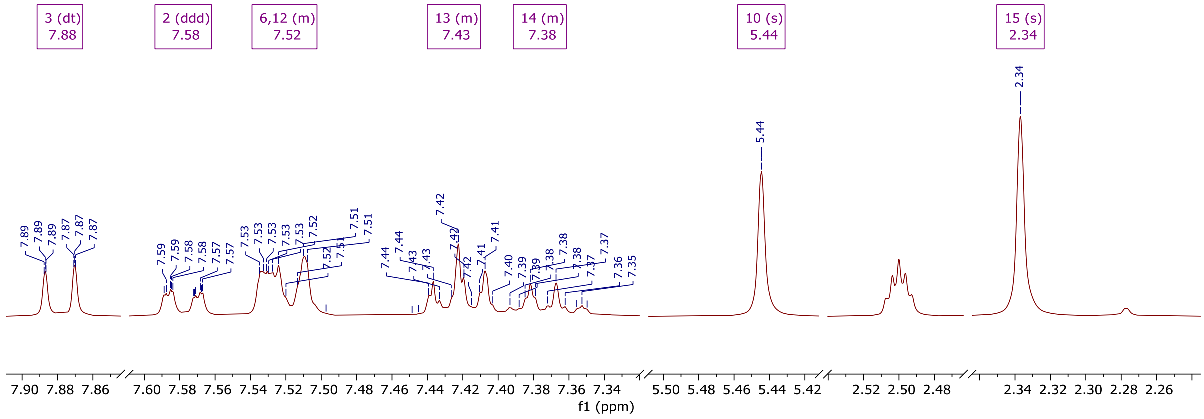

^{13}C NMR (126 MHz, DMSO-d_6)

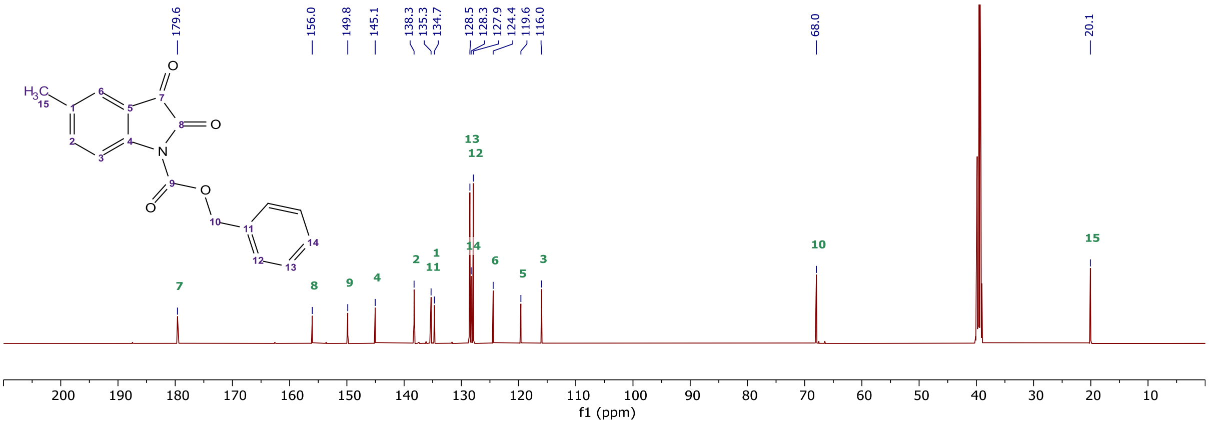

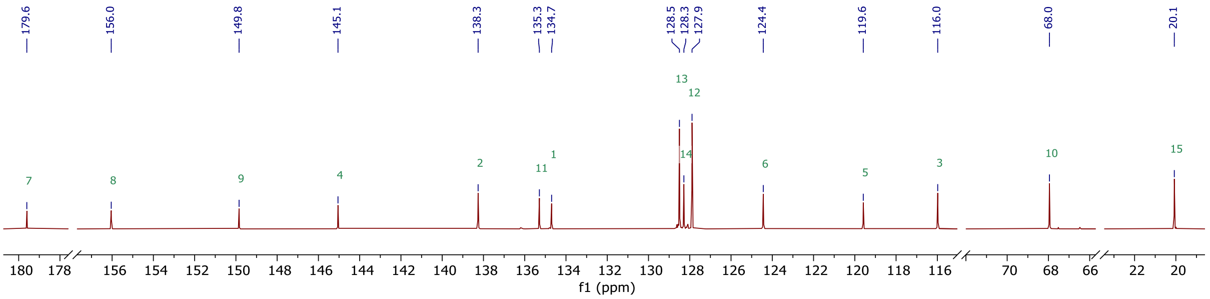

$^{1}H/^{1}H$ COSY

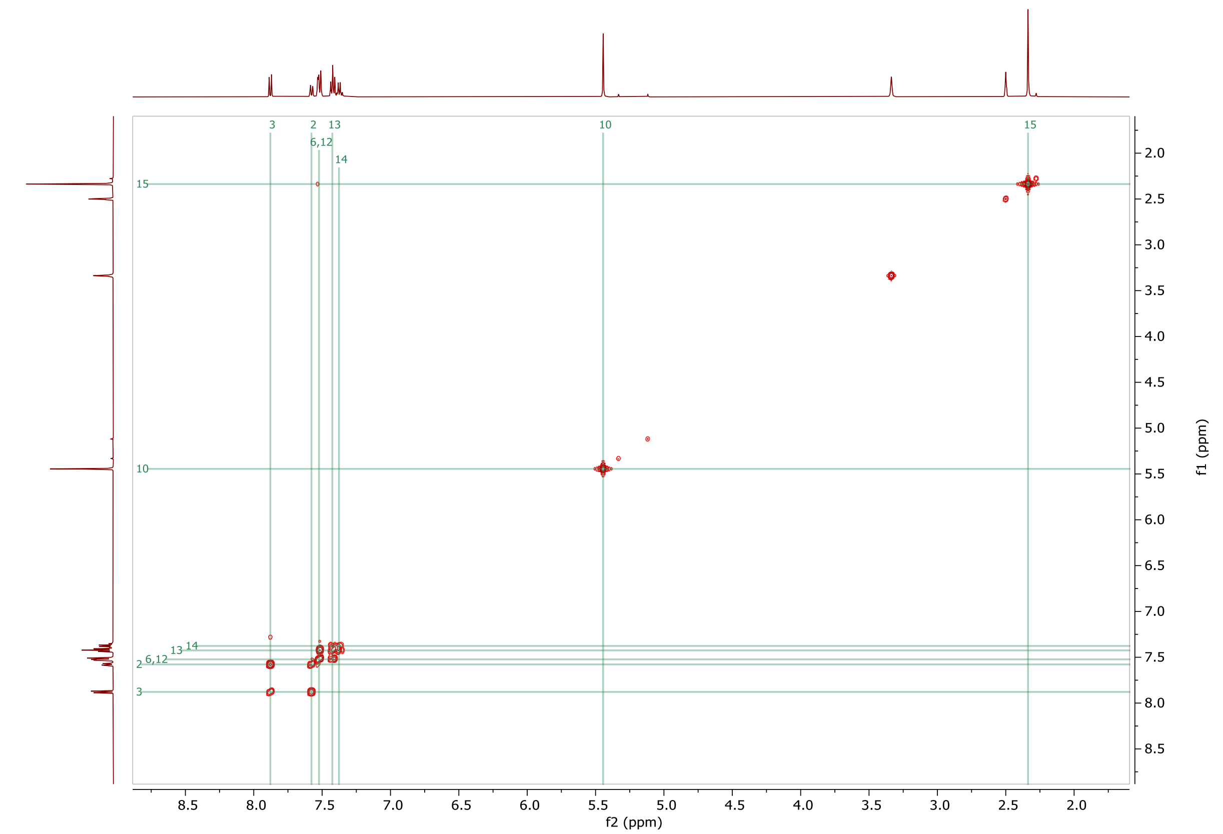

$^{1}H/^{13}C$ HSQC

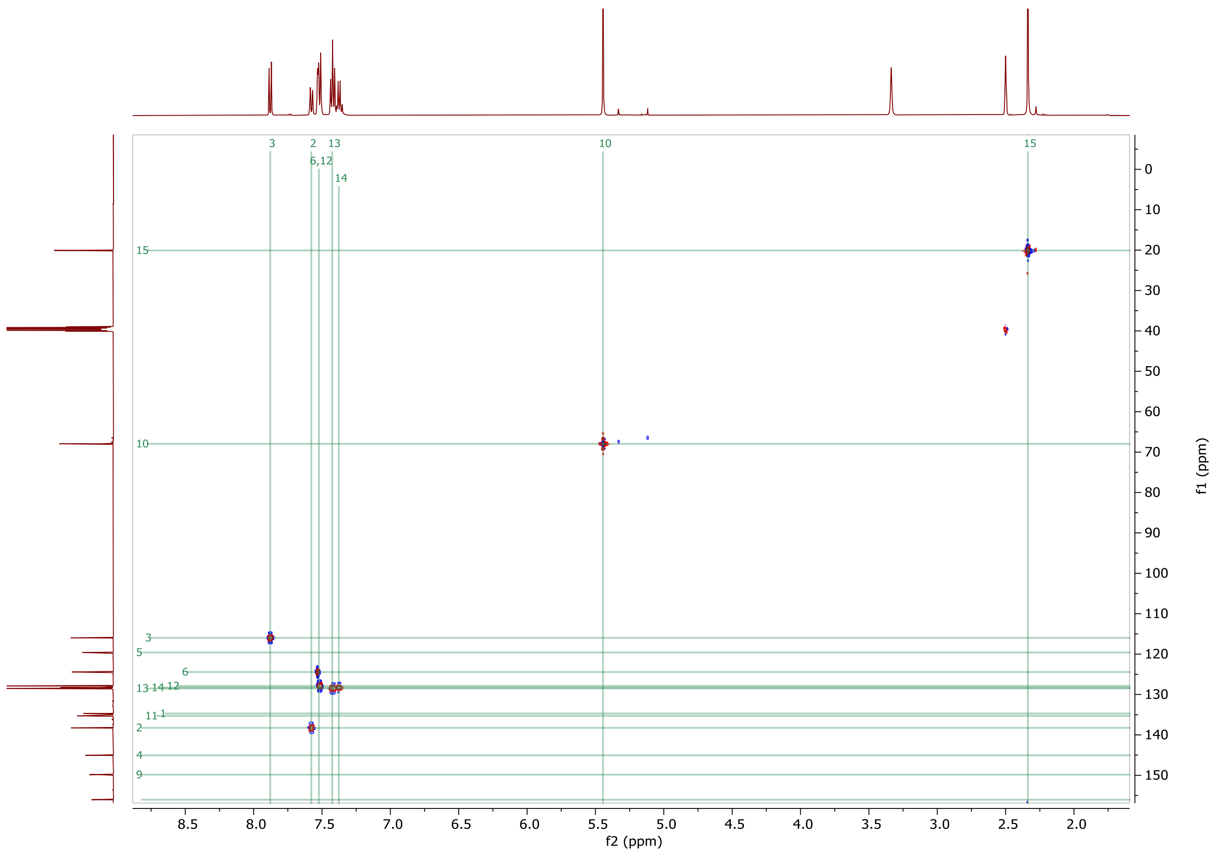

$^1H/^{13}C$ HMBC

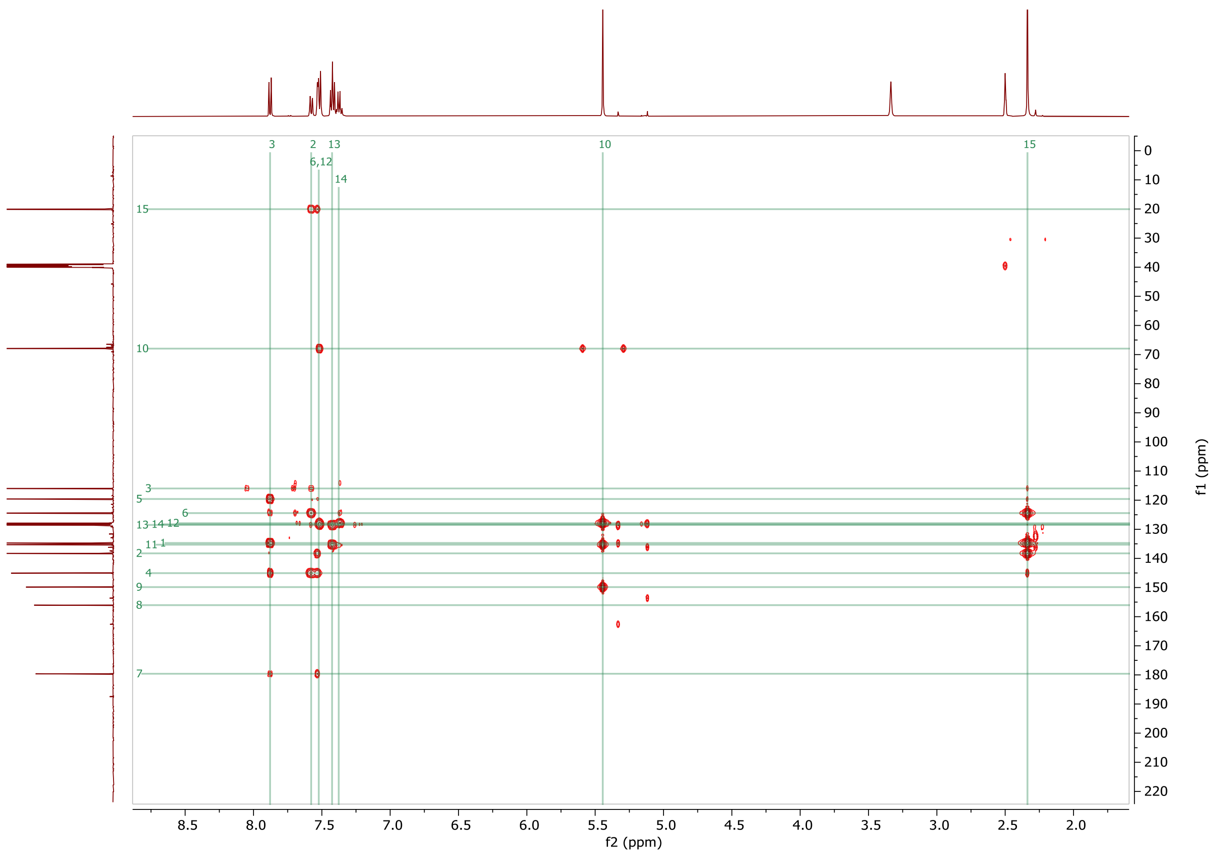

Isopropyl 2-(2-(((benzyloxy)carbonyl)amino)-5-methylphenyl)-2-oxoacetate 15f

^{1}H NMR (500 MHz, $CDCl_3$)

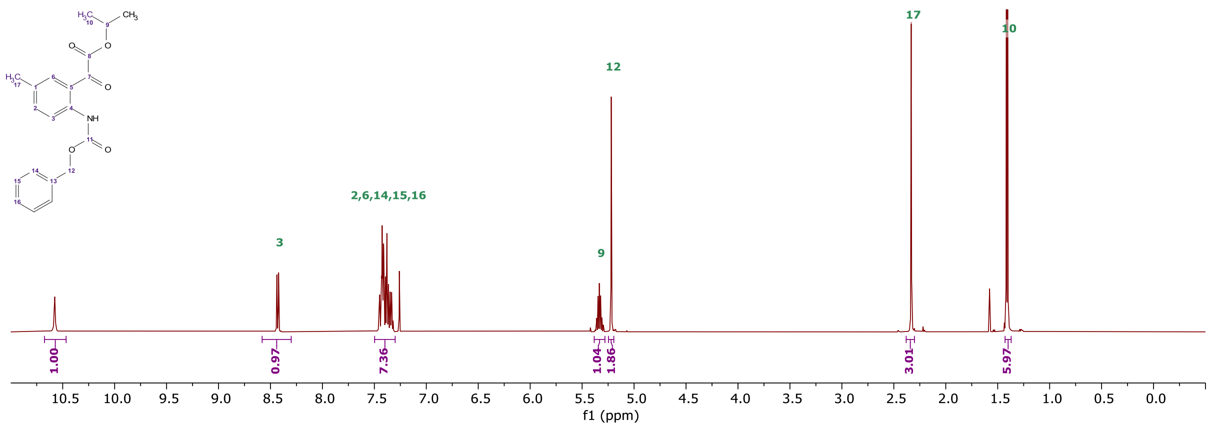

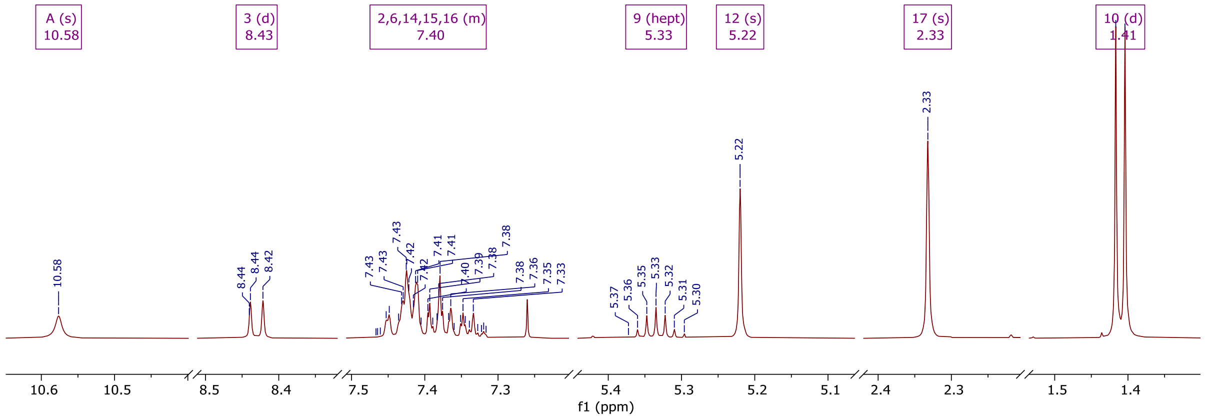

^{13}C NMR (126 MHz, $CDCl_3$)

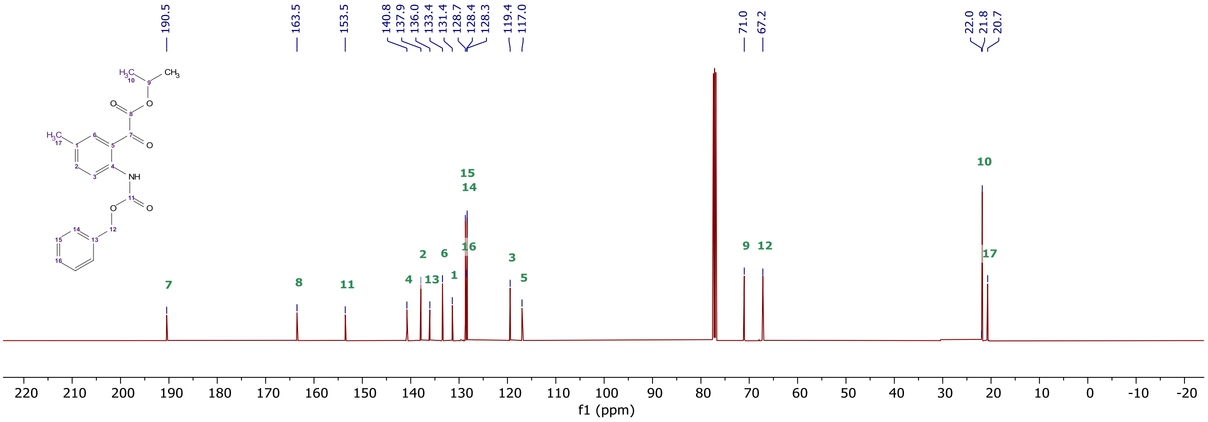

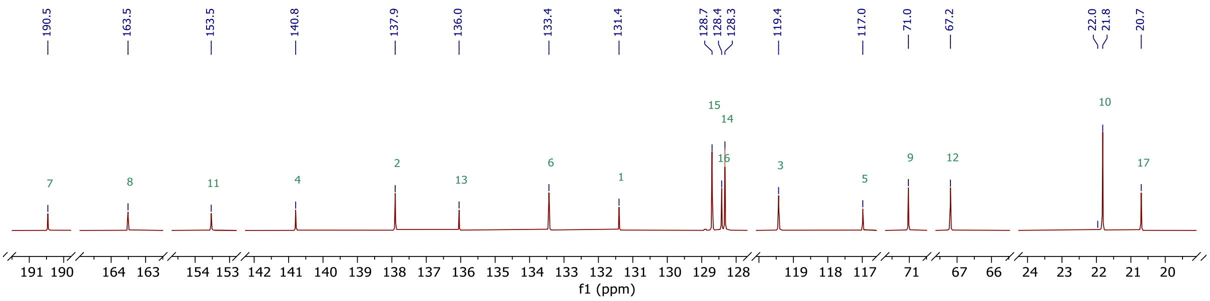

$^1H/^1H$ COSY

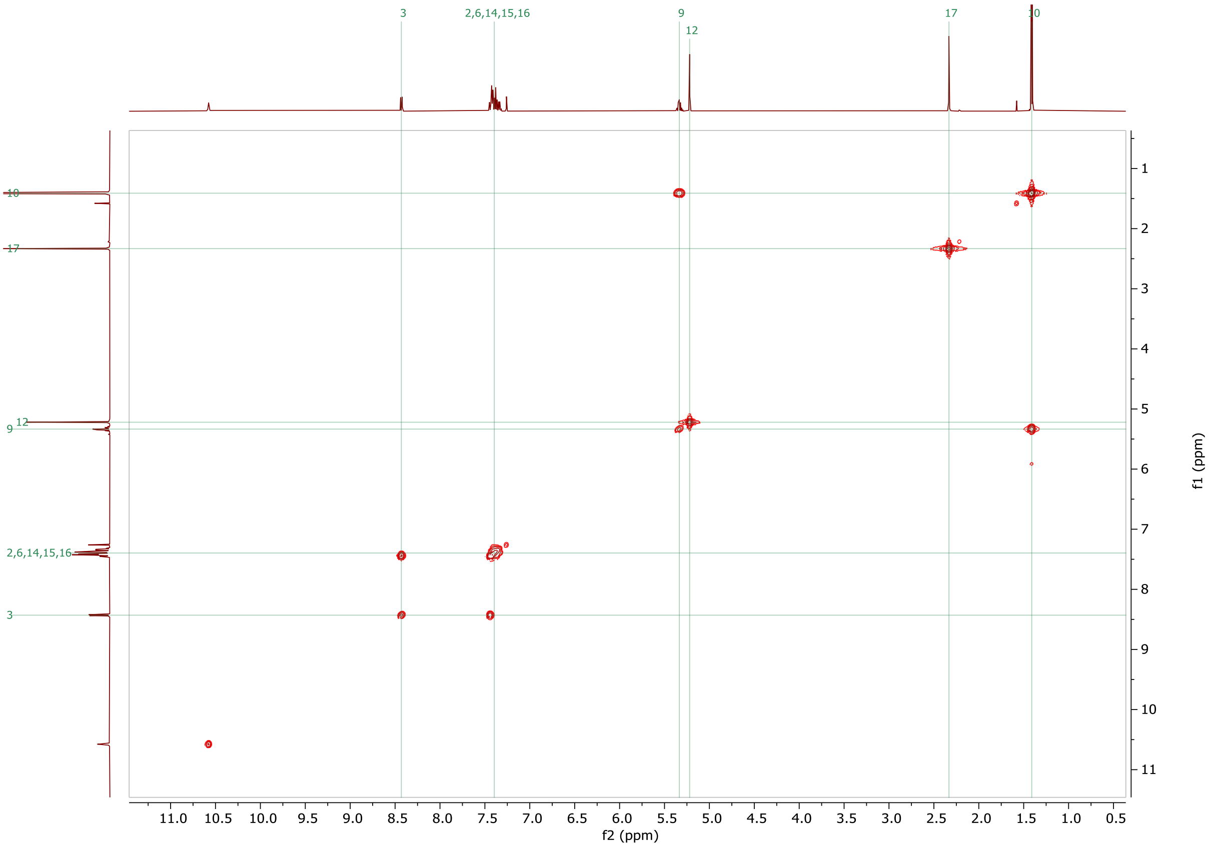

$^1H/^{13}C$ HSQC

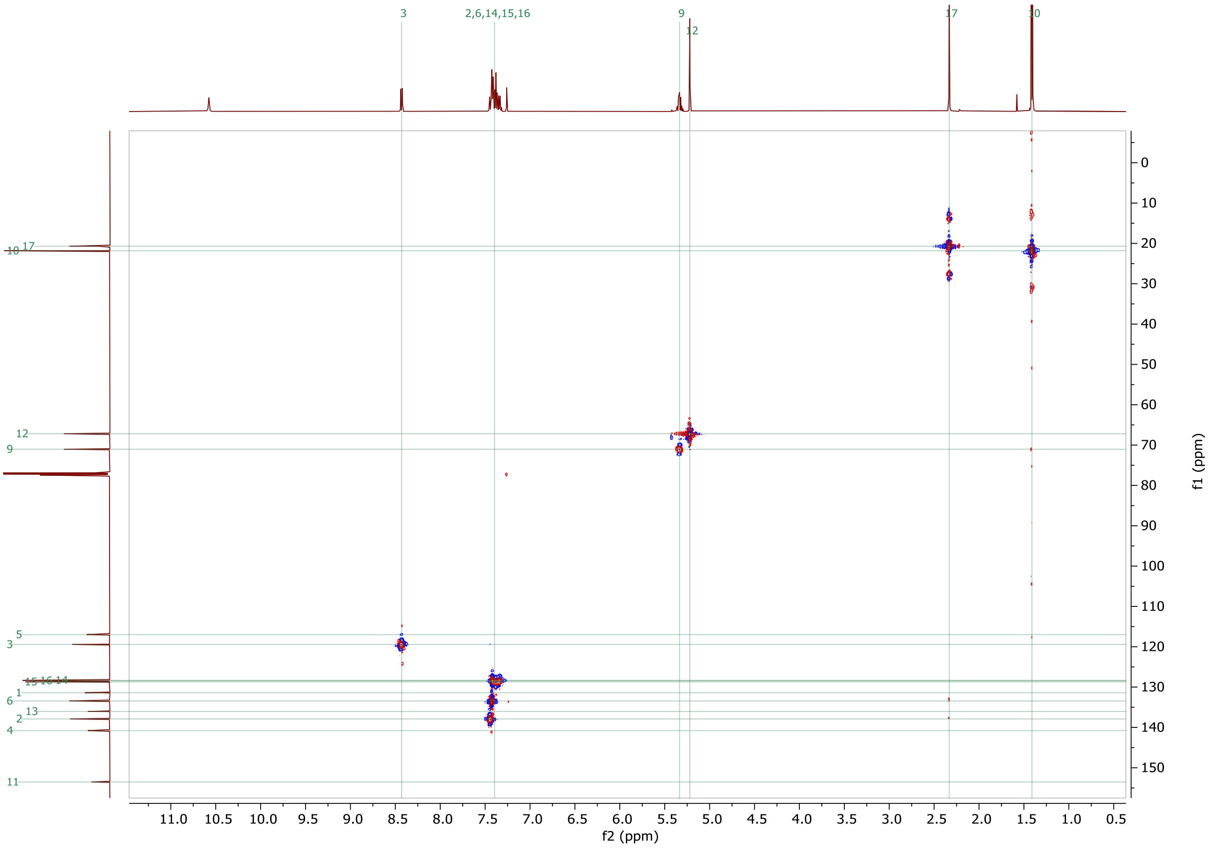

$^1H/^{13}C$ HMBC

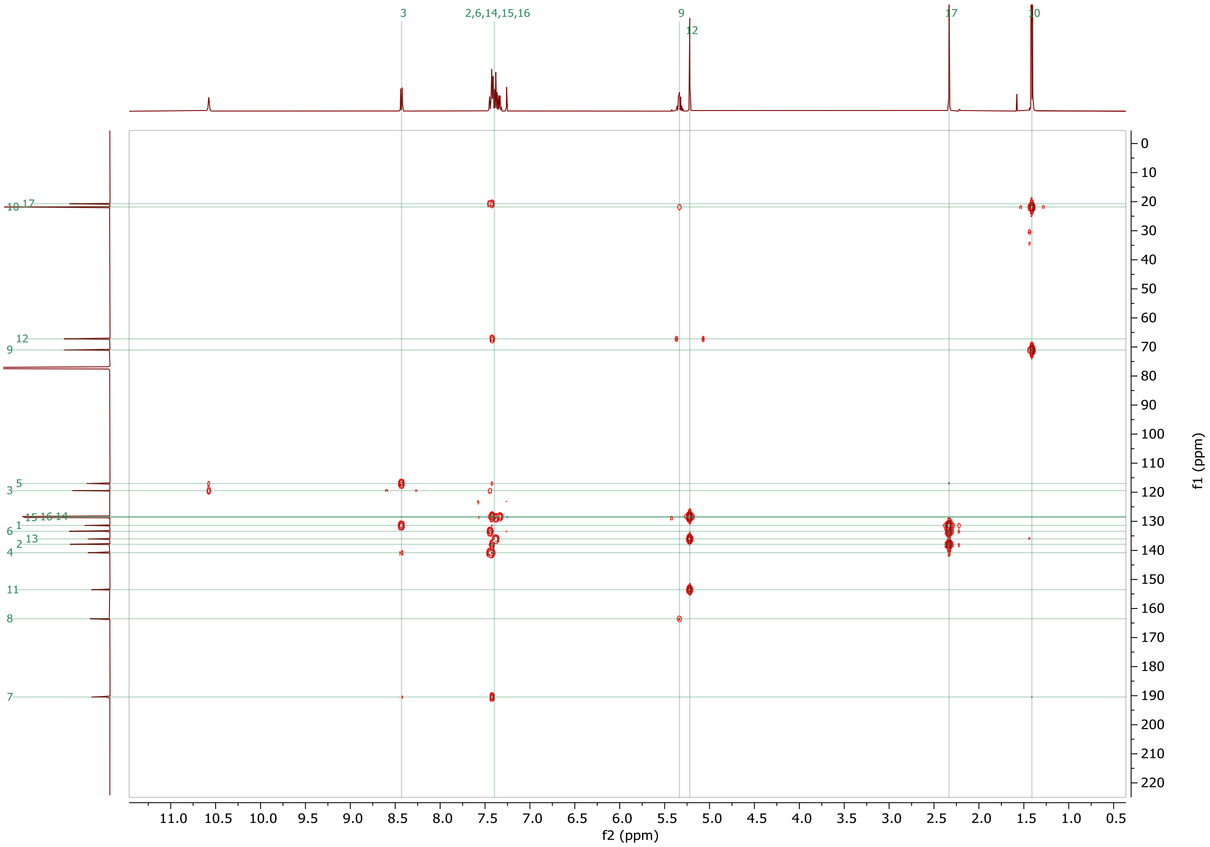

Benzyl 7-fluoro-2,3-dioxoindoline-1-carboxylate S5

^{1}H NMR (500 MHz, DMSO-d_6)

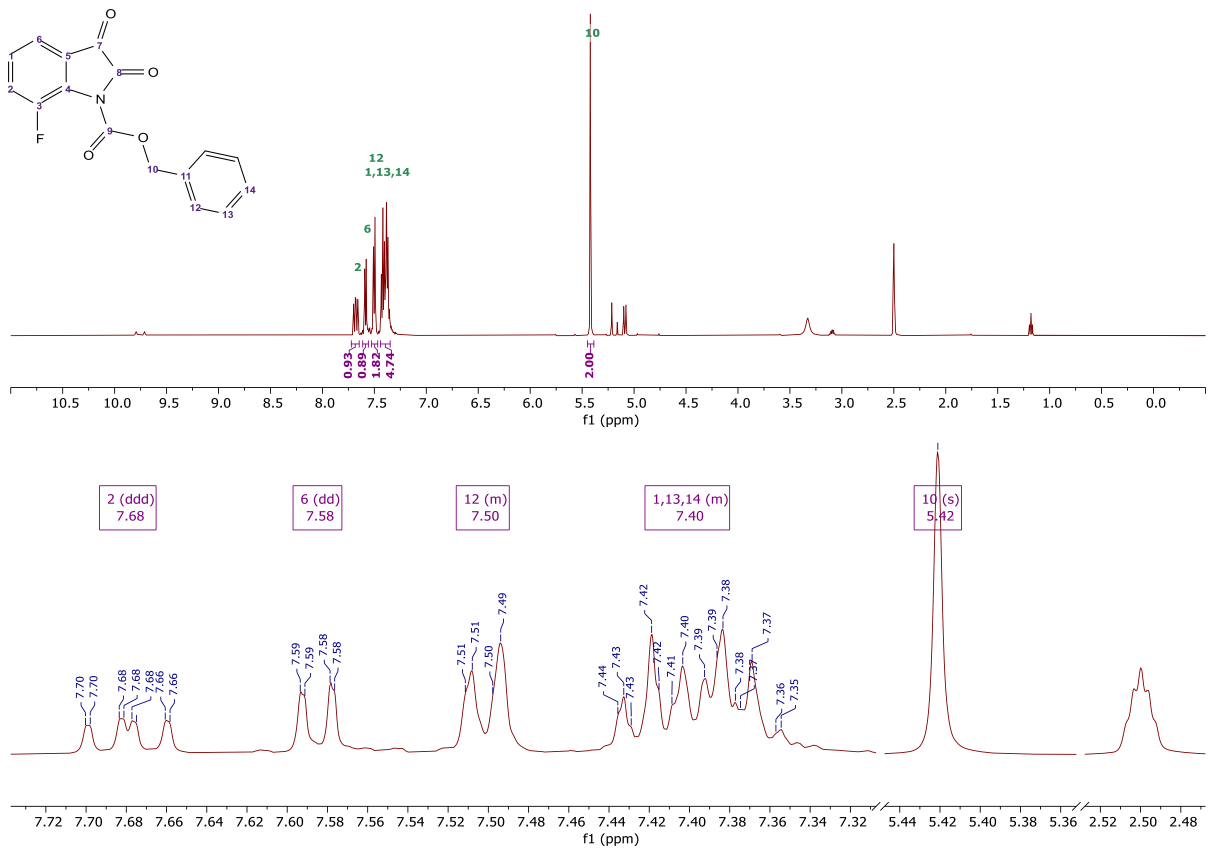

^{13}C NMR (126 MHz, DMSO-d_6)

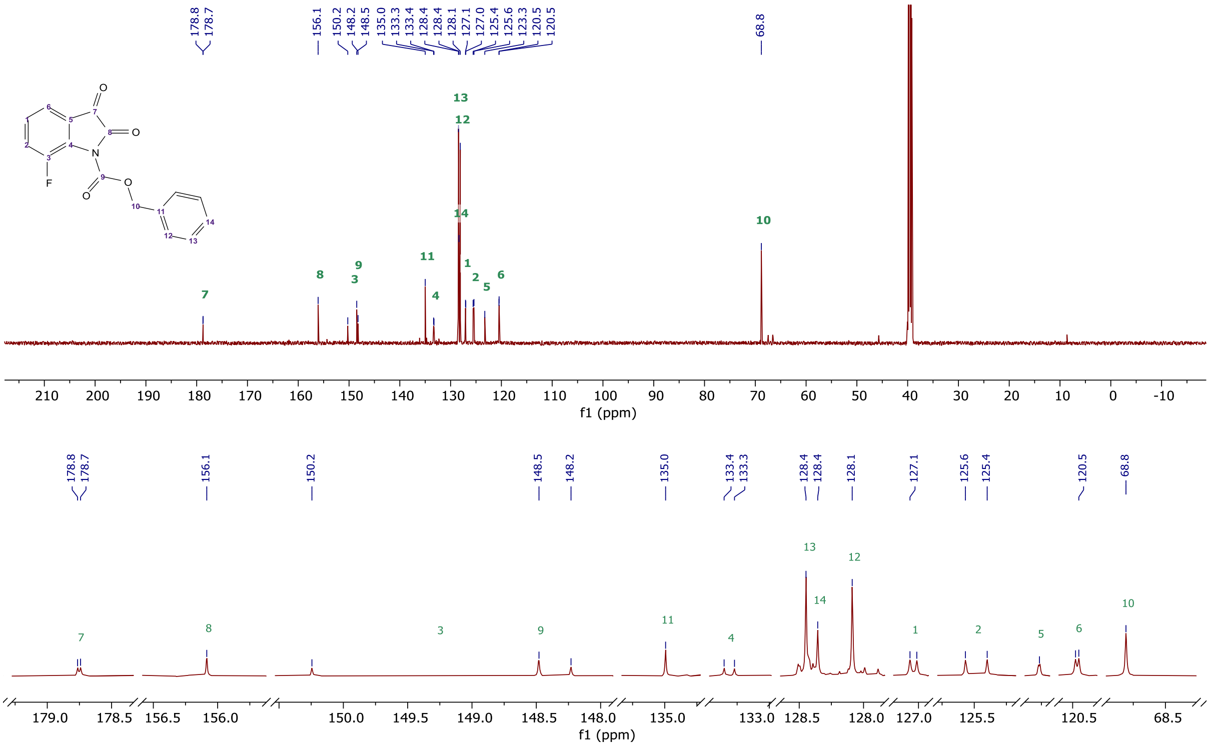

^{19}F NMR (471 MHz, DMSO-d_6)

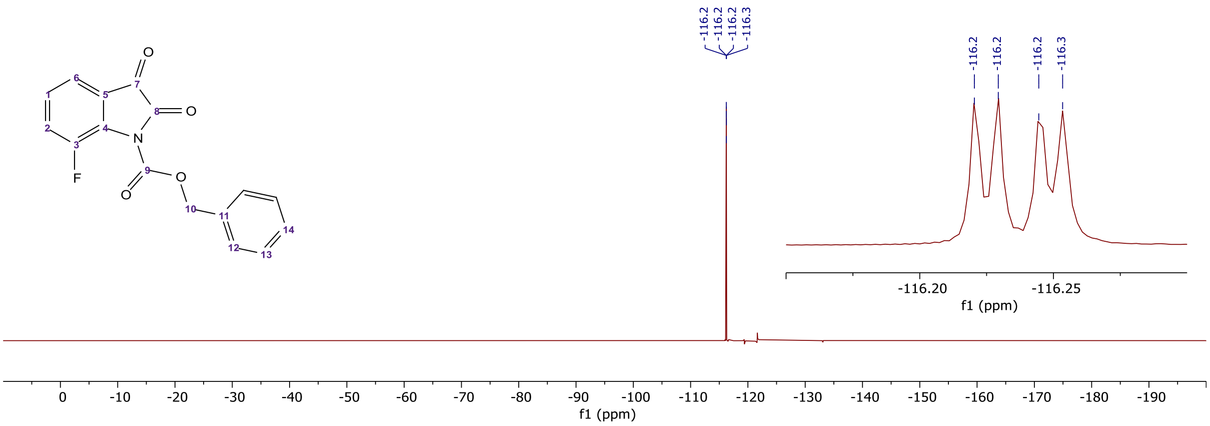

$^{1}H/^{1}H$ COSY

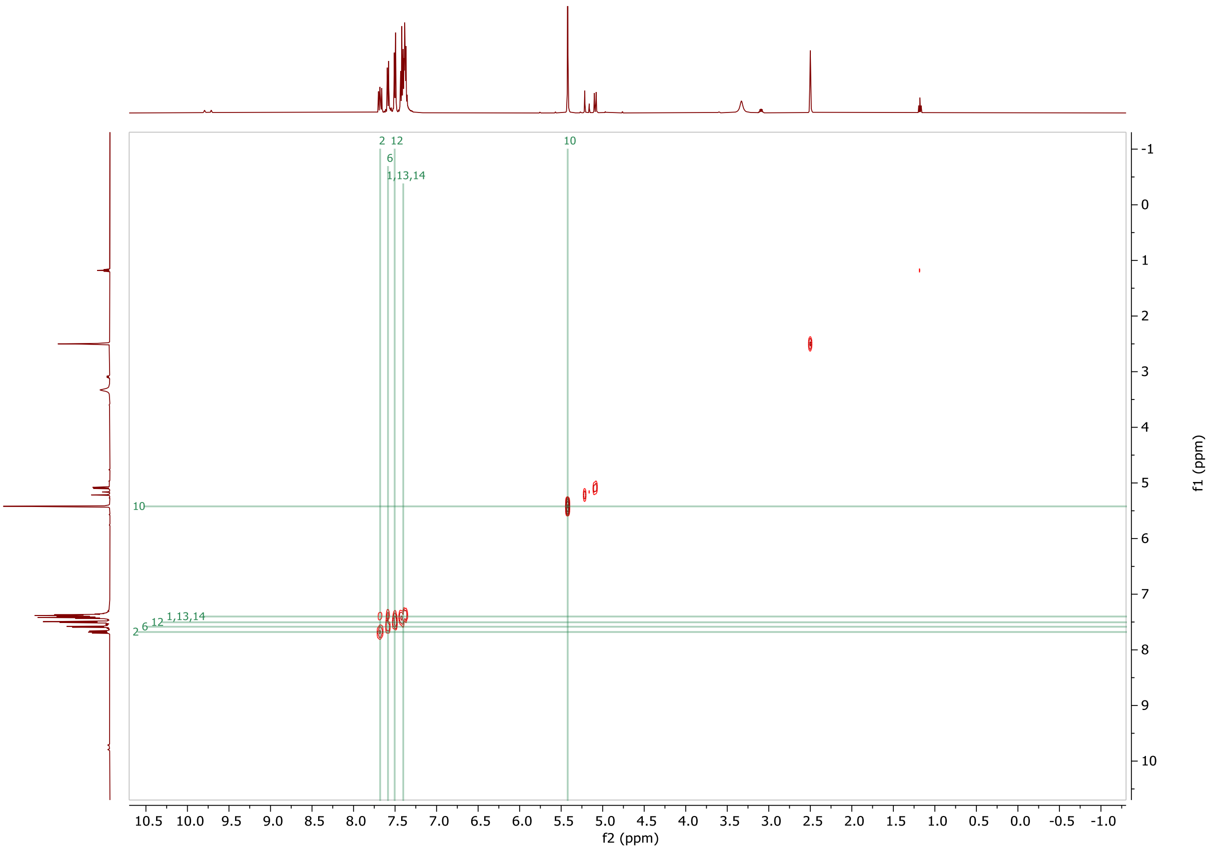

$^{1}H/^{13}C$ HSQC

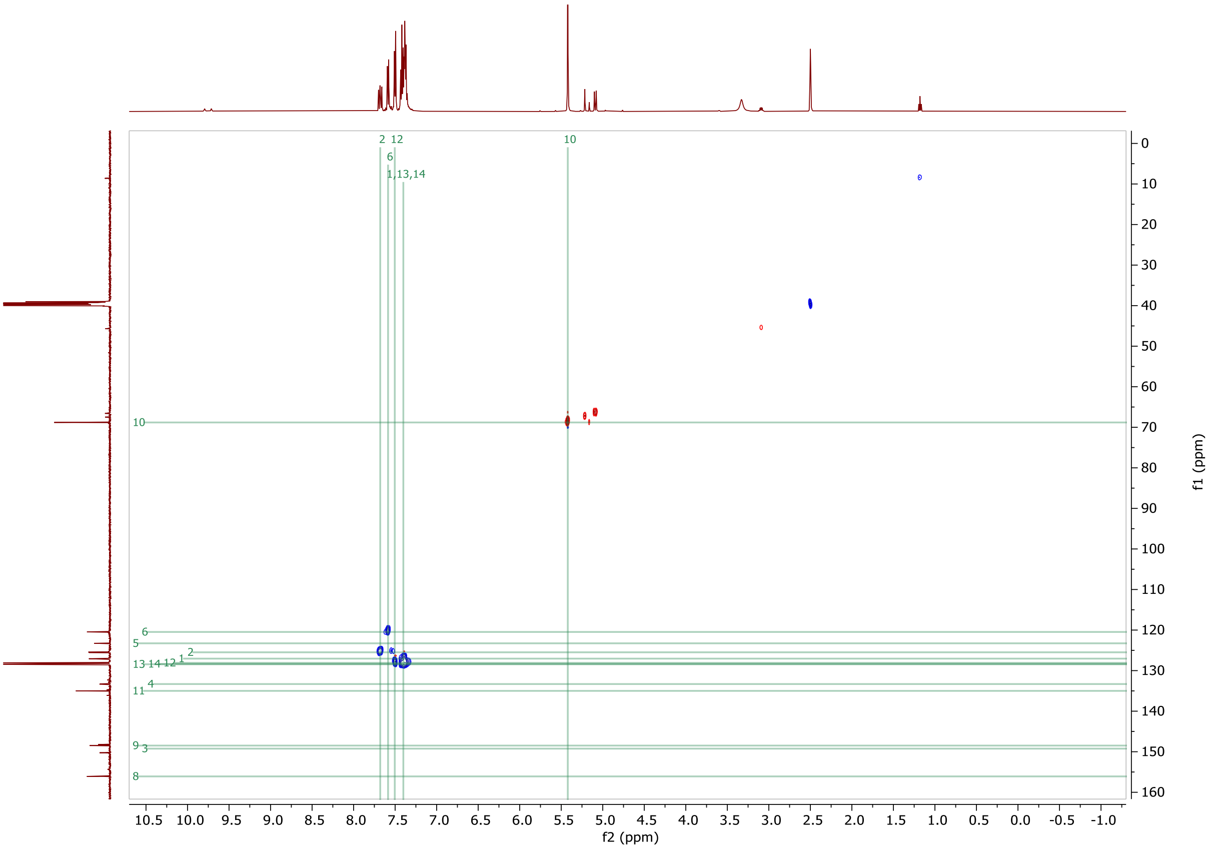

$^{1}H/^{13}C$ HMBC

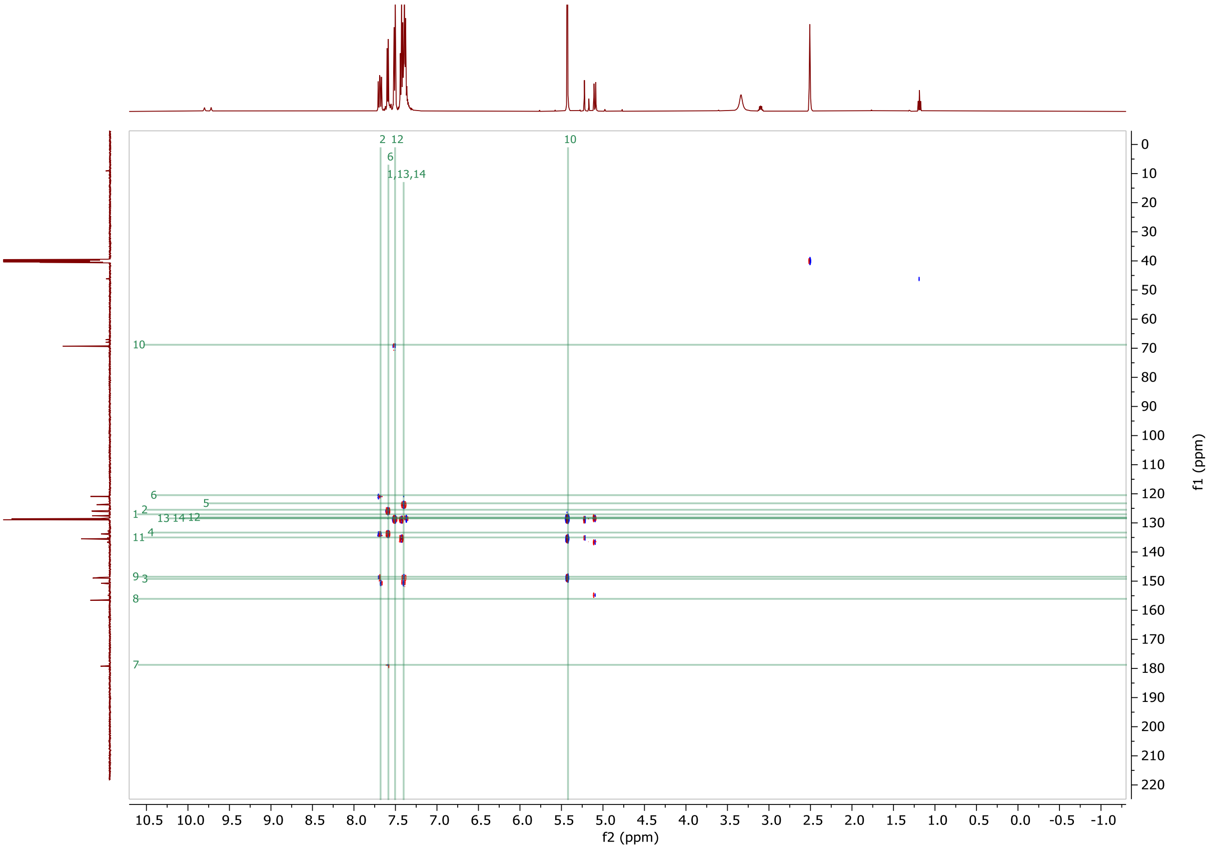

Isopropyl 2-(2-(((benzyloxy)carbonyl)amino)-3-fluorophenyl)-2-oxoacetate 15i

^{1}H NMR (500 MHz, $CDCl_3$)

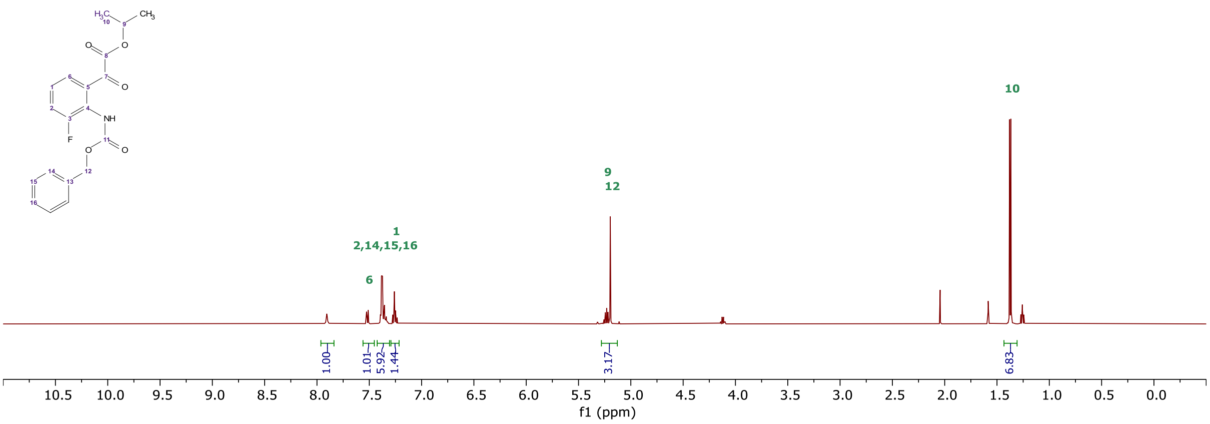

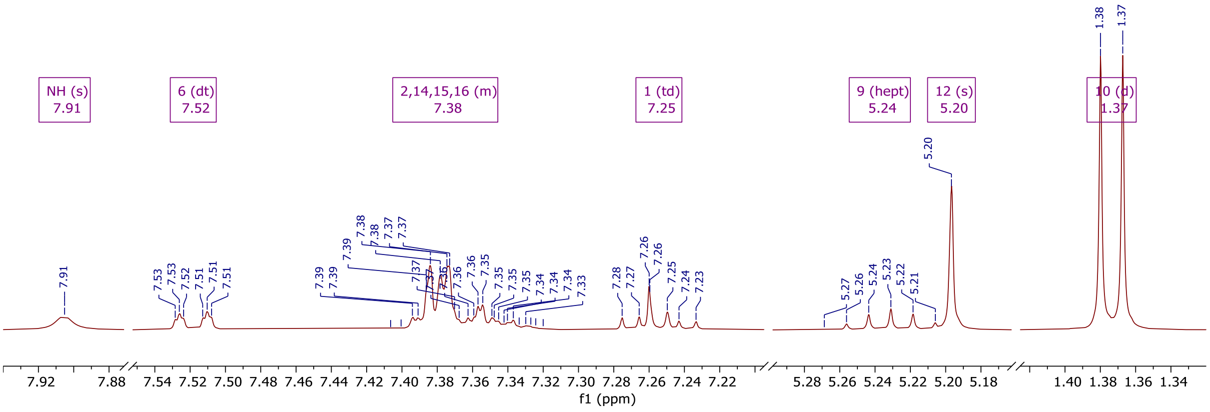

^{13}C NMR (126 MHz, $CDCl_3$)

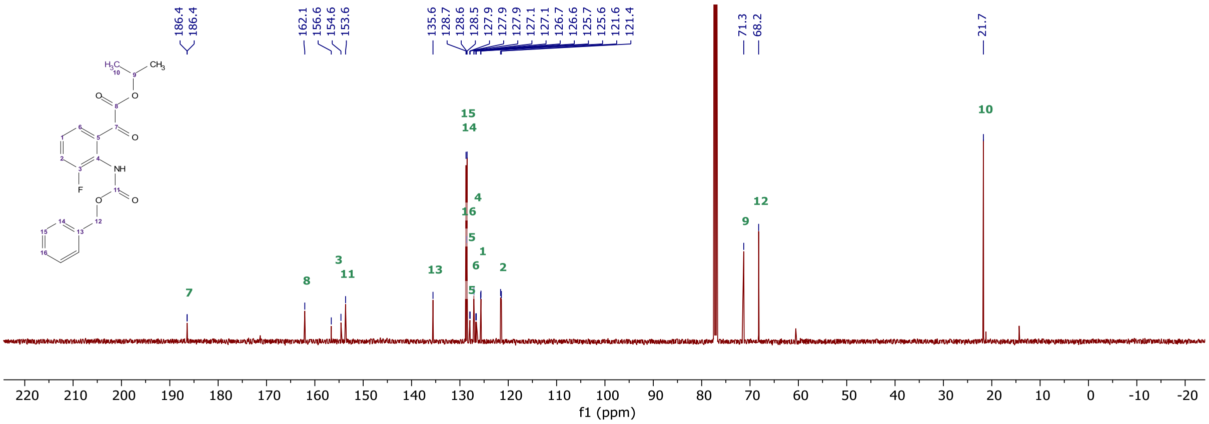

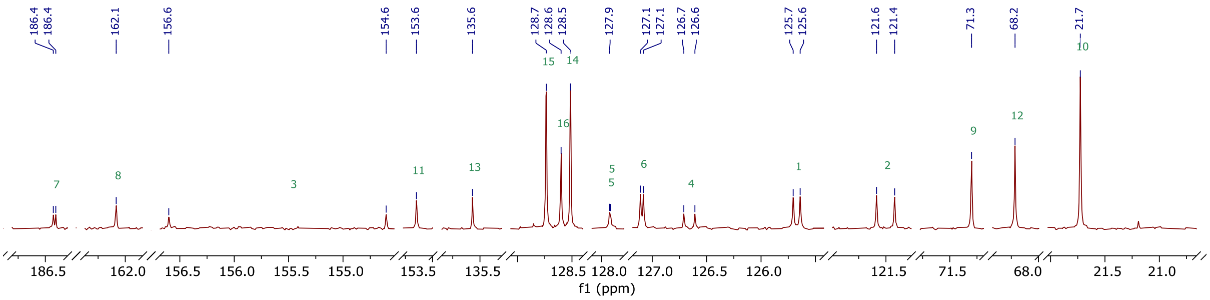

^{19}F NMR (471 MHz, $CDCl_3$)

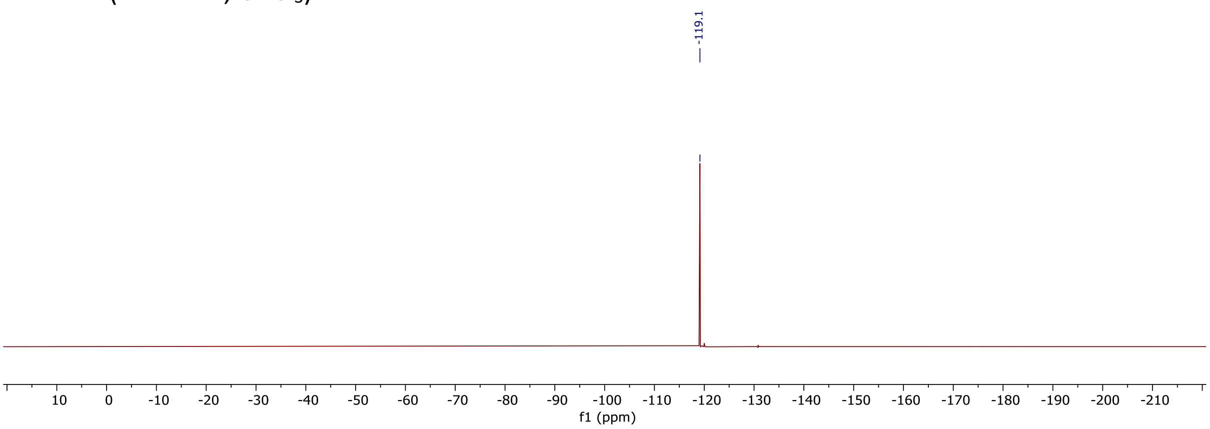

$^{1}H/^{1}H$ COSY

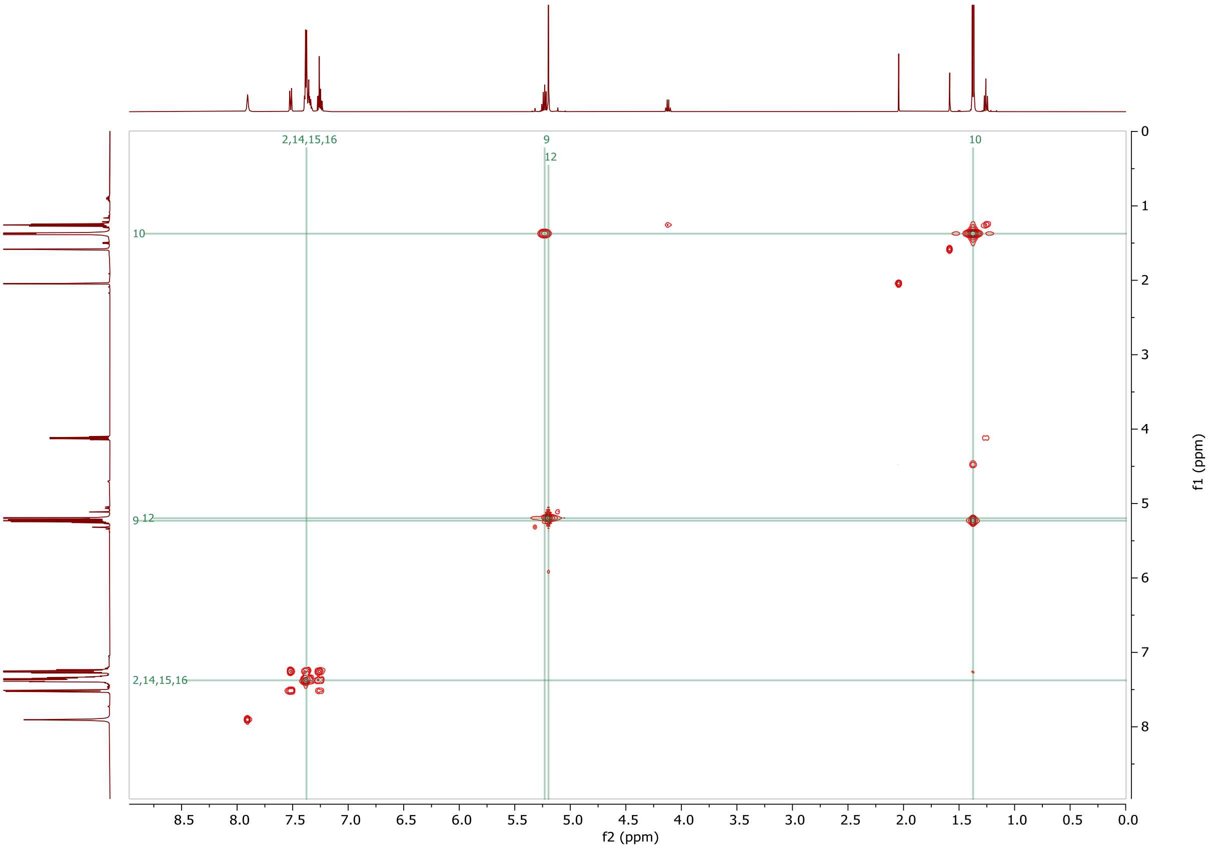

$^{1}H/^{13}C$ HSQC

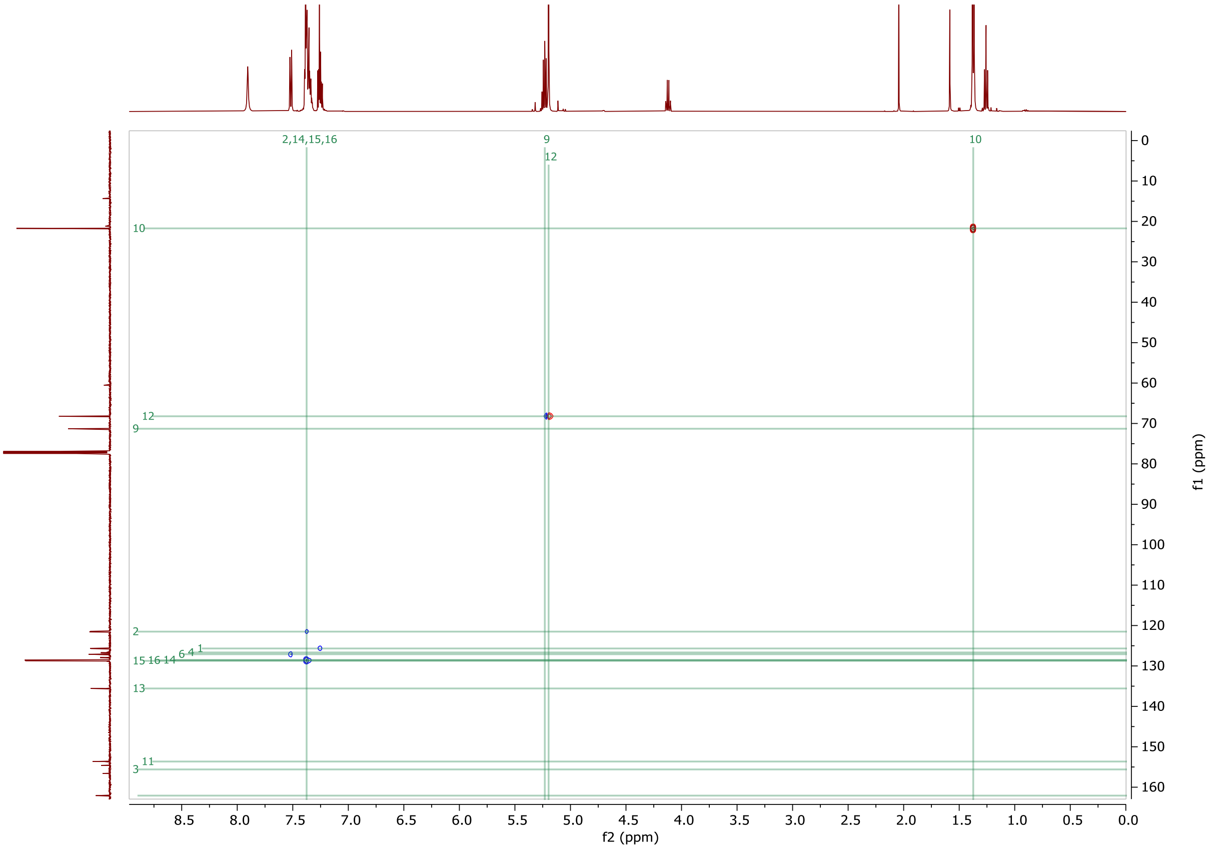

$^{1}H/^{13}C$ HMBC

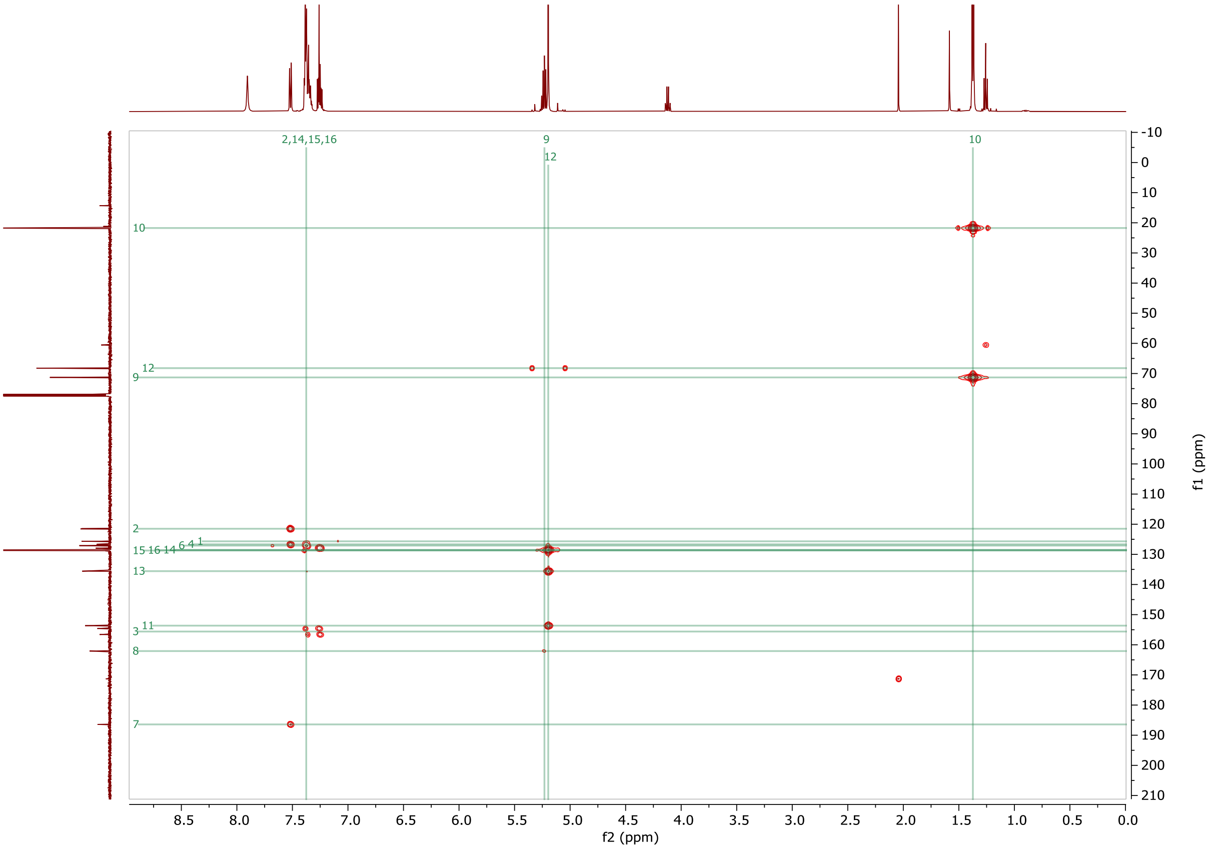

Isopropyl 2-(2-(((benzyloxy)carbonyl)amino)-5-nitrophenyl)-2-oxoacetate 15g

^{1}H NMR (500 MHz, $CDCl_3$)

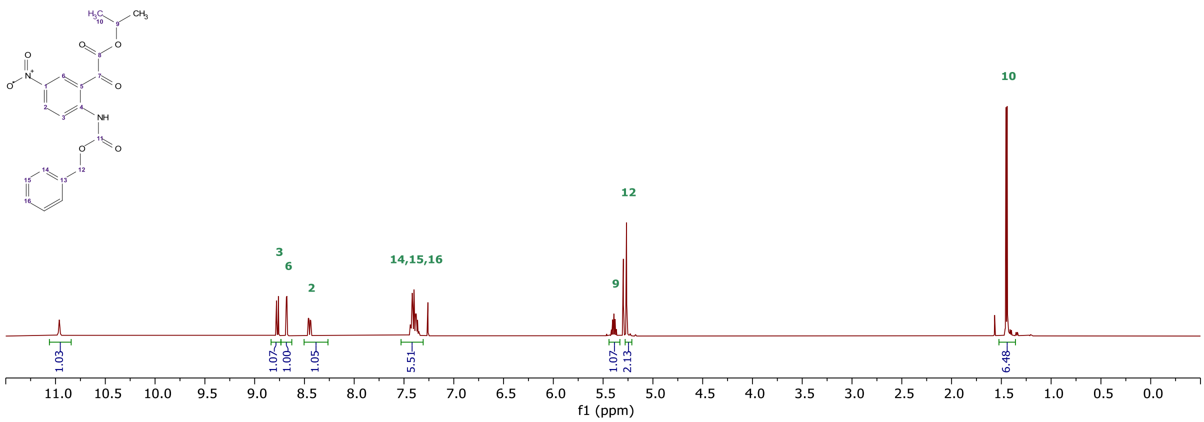

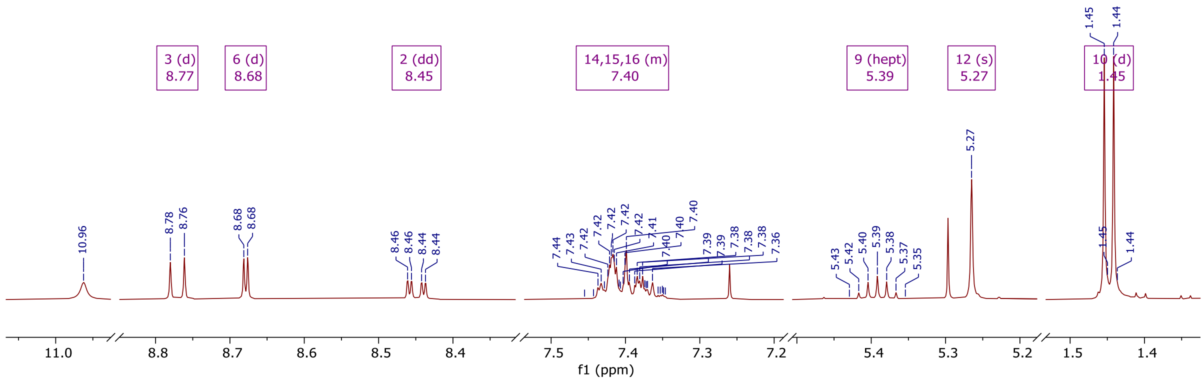

^{13}C NMR (126 MHz, $CDCl_3$)

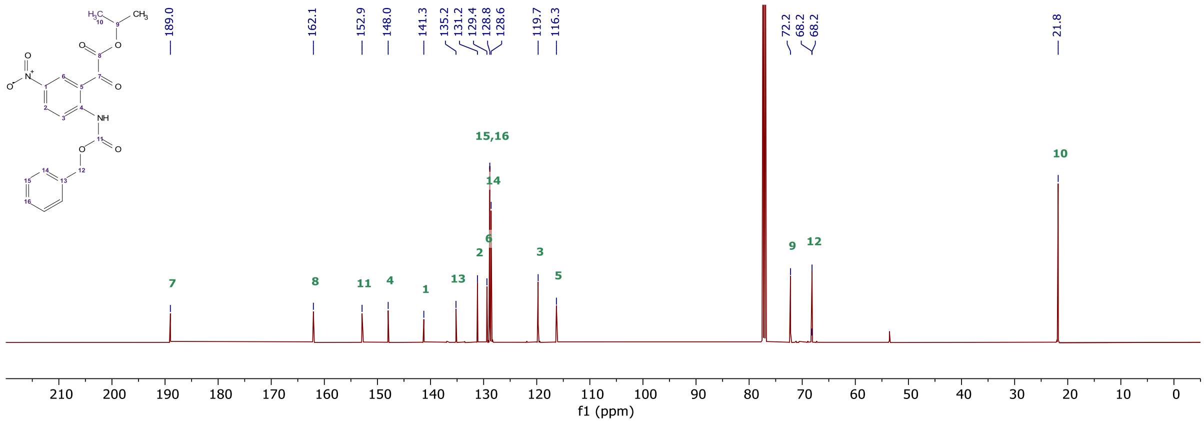

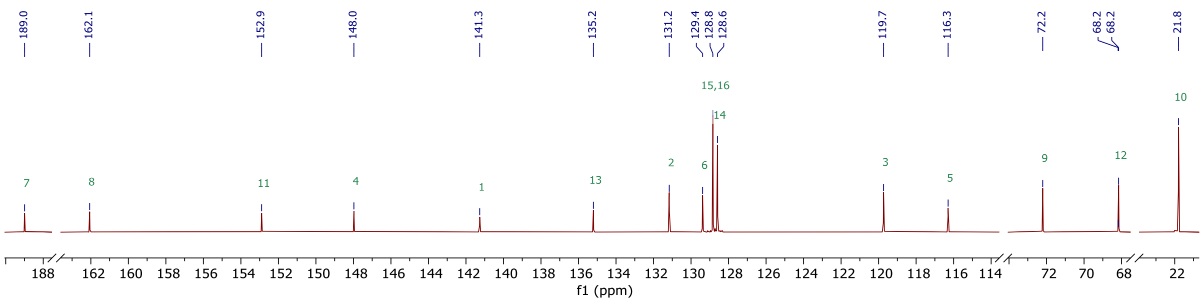

$^{1}H/^{1}H$ COSY

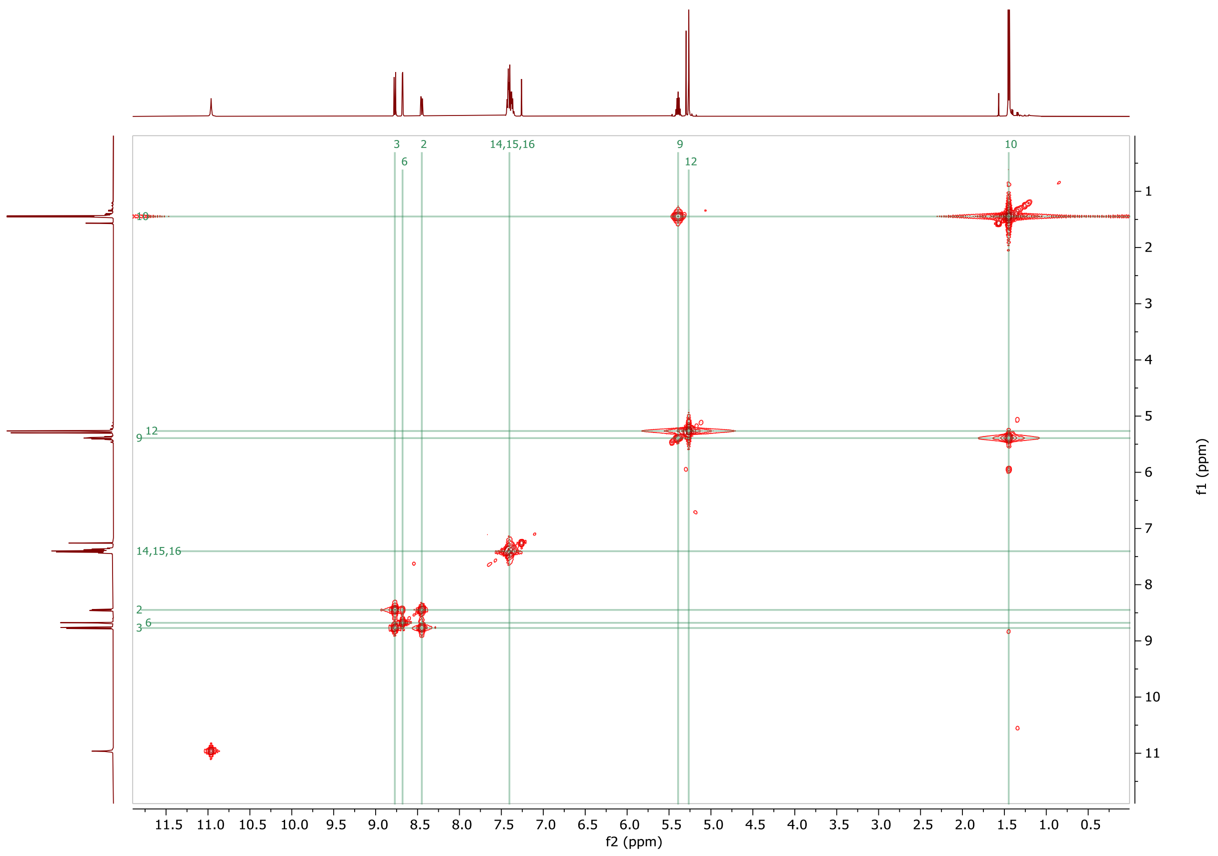

$^{1}H/^{13}C$ HSQC

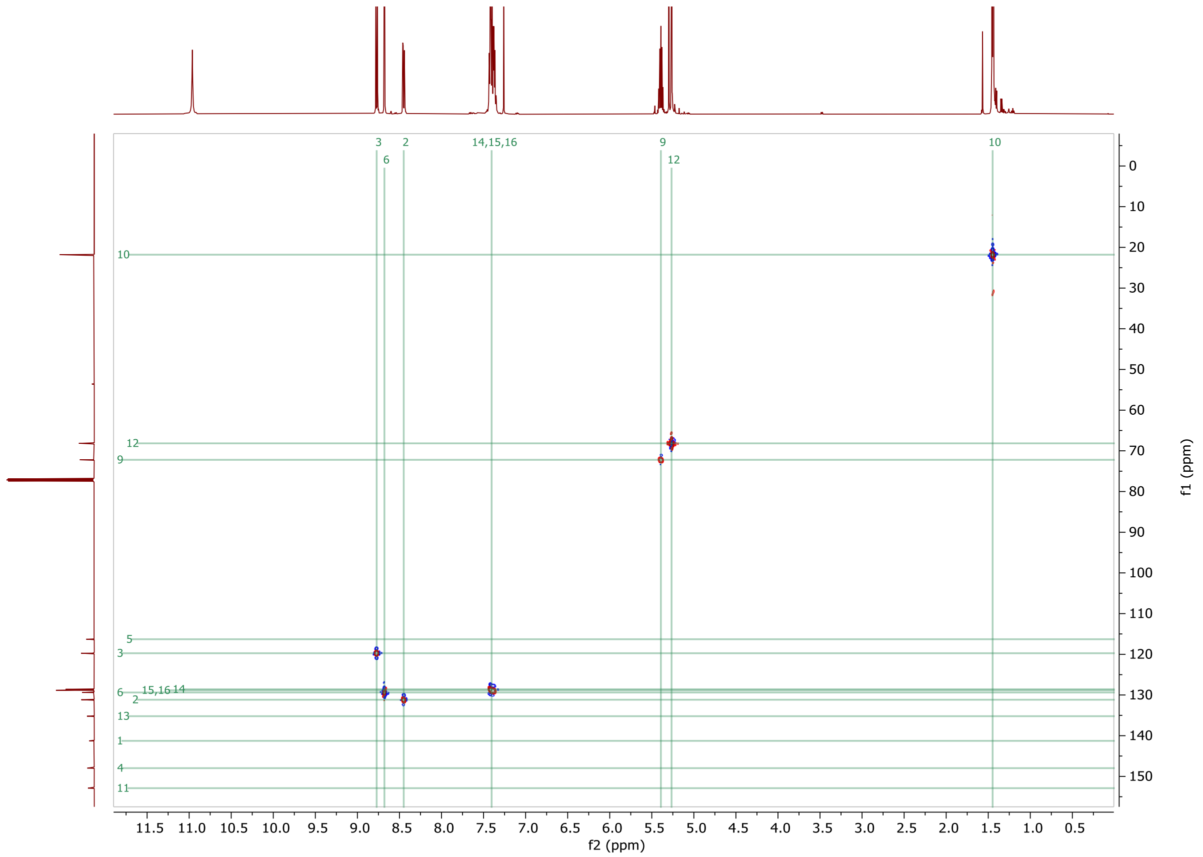

$^{1}H/^{13}C$ HMBC

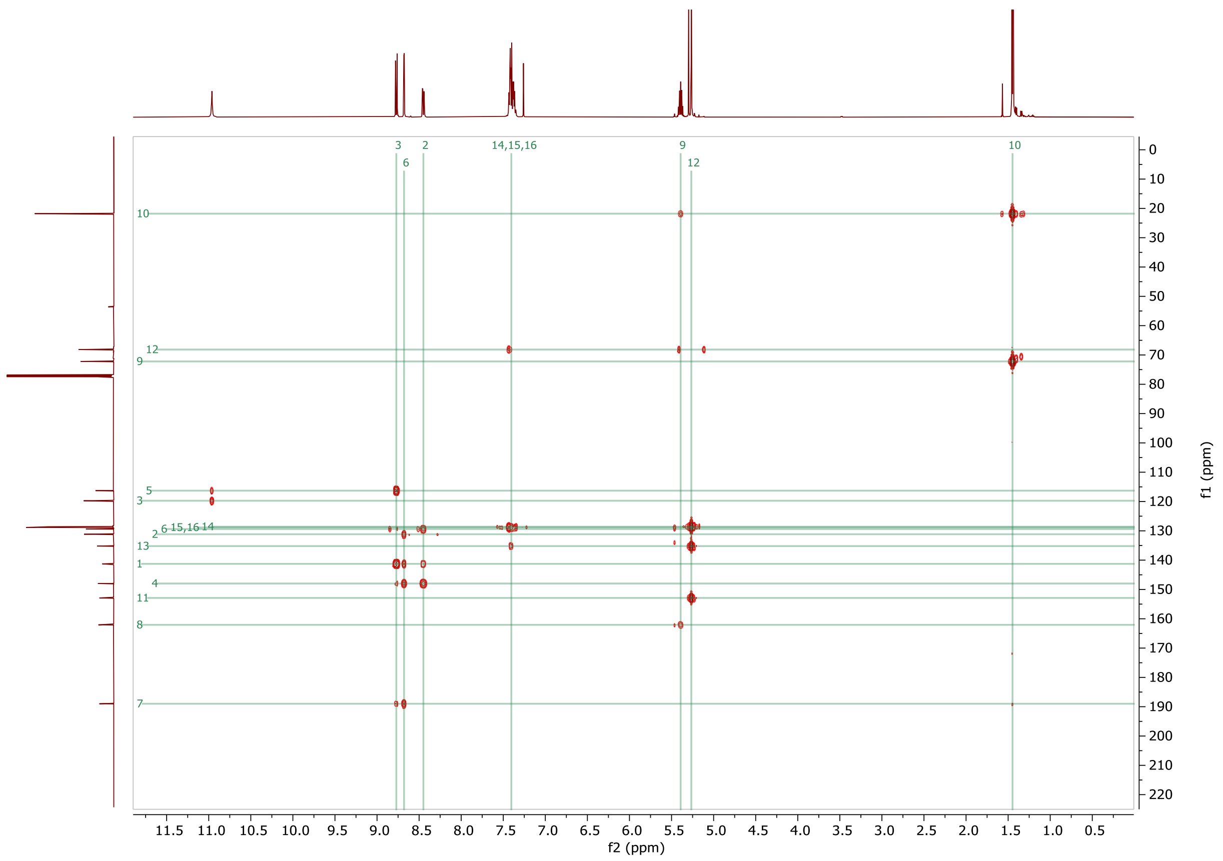

Isopropyl 2-(2-(((benzyloxy)carbonyl)amino)-5-methoxyphenyl)-2-oxoacetate 15r

^{1}H NMR (500 MHz, $CDCl_3$)

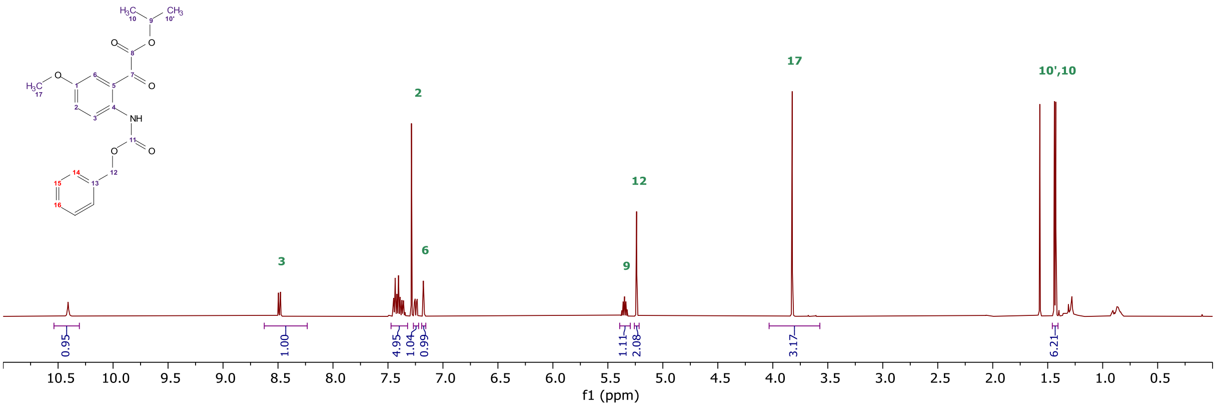

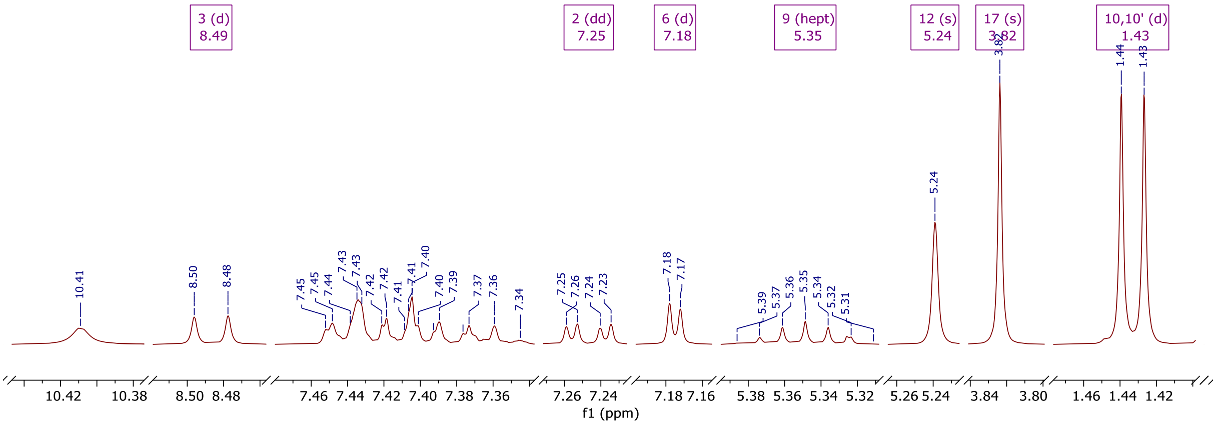

^{13}C NMR (126 MHz, $CDCl_3$)

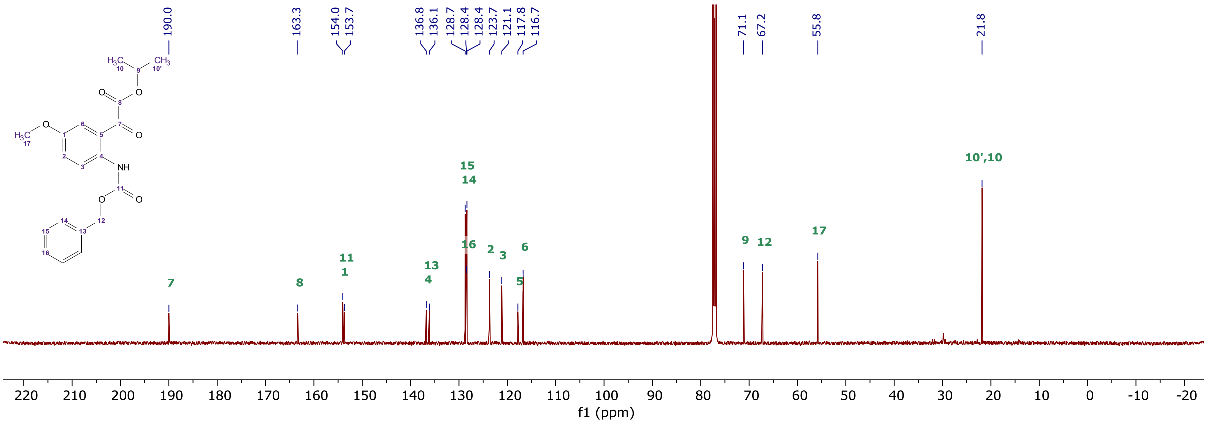

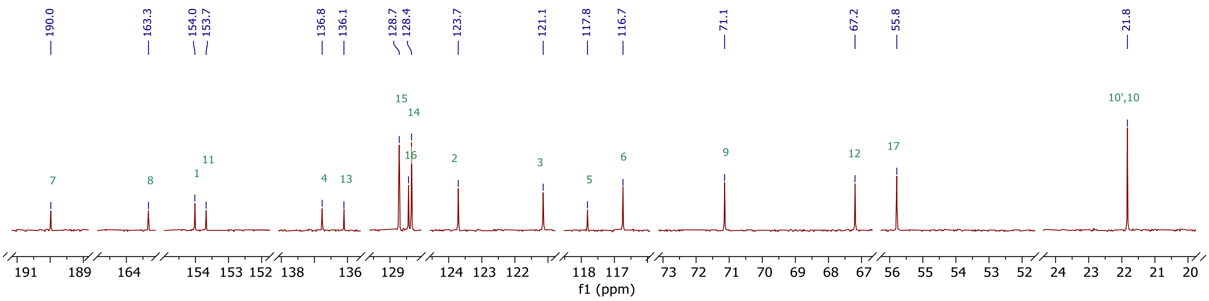

$^1H/^1H$ COSY

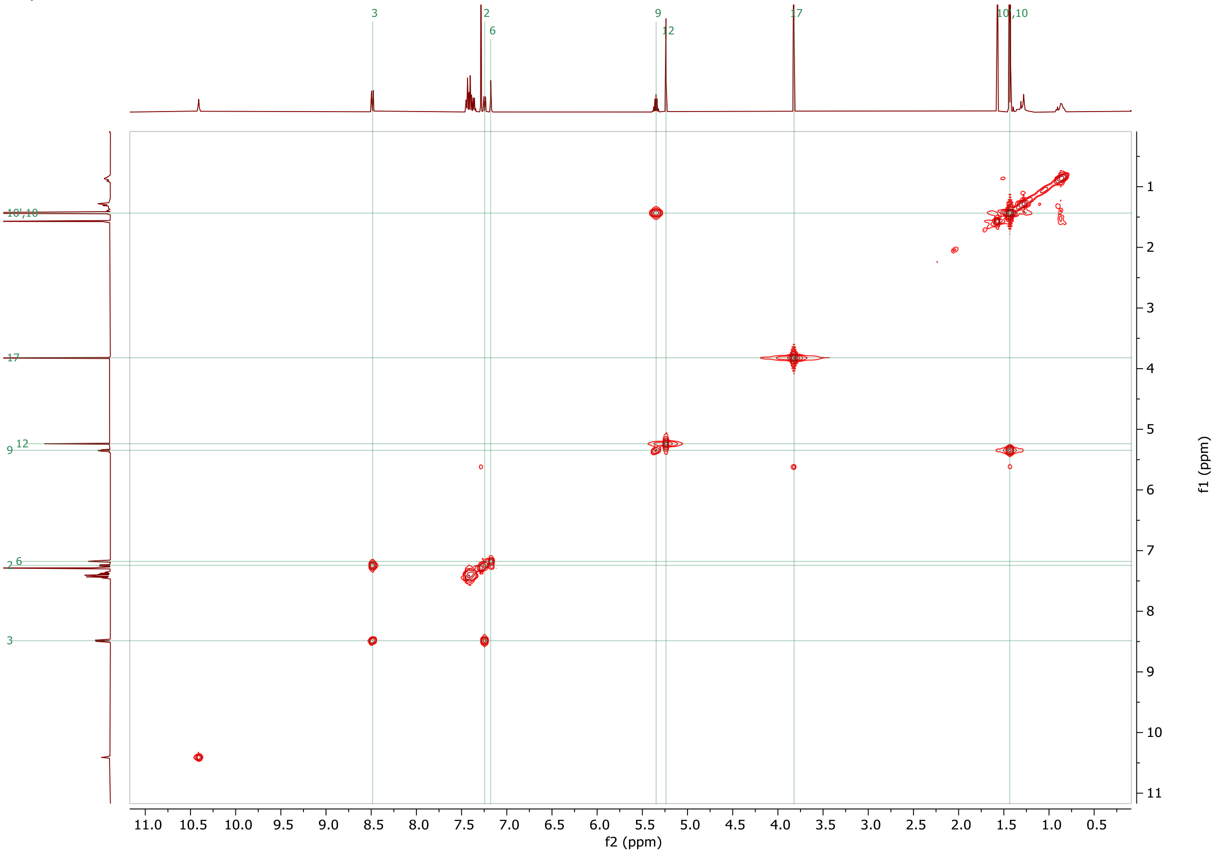

$^1H/^{13}C$ HSQC

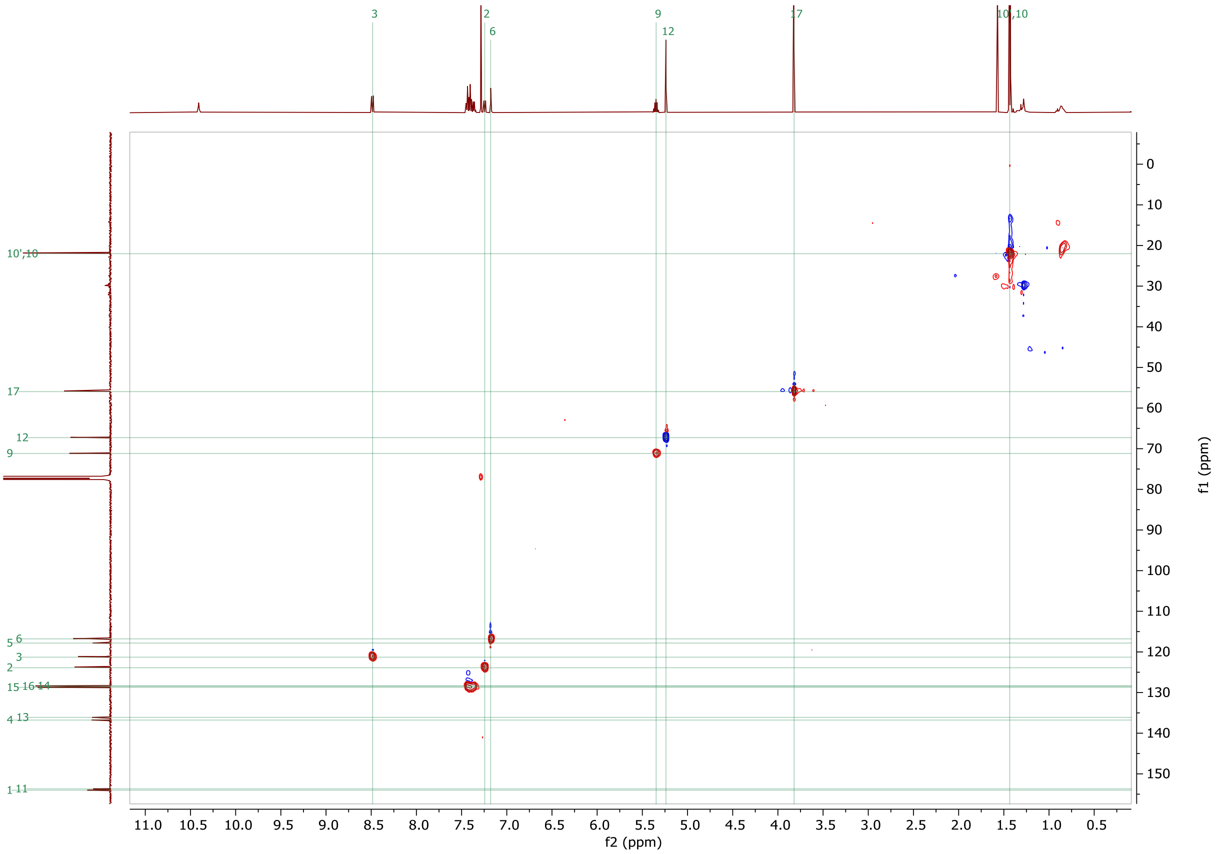

$^{1}H/^{13}C$ HMBC

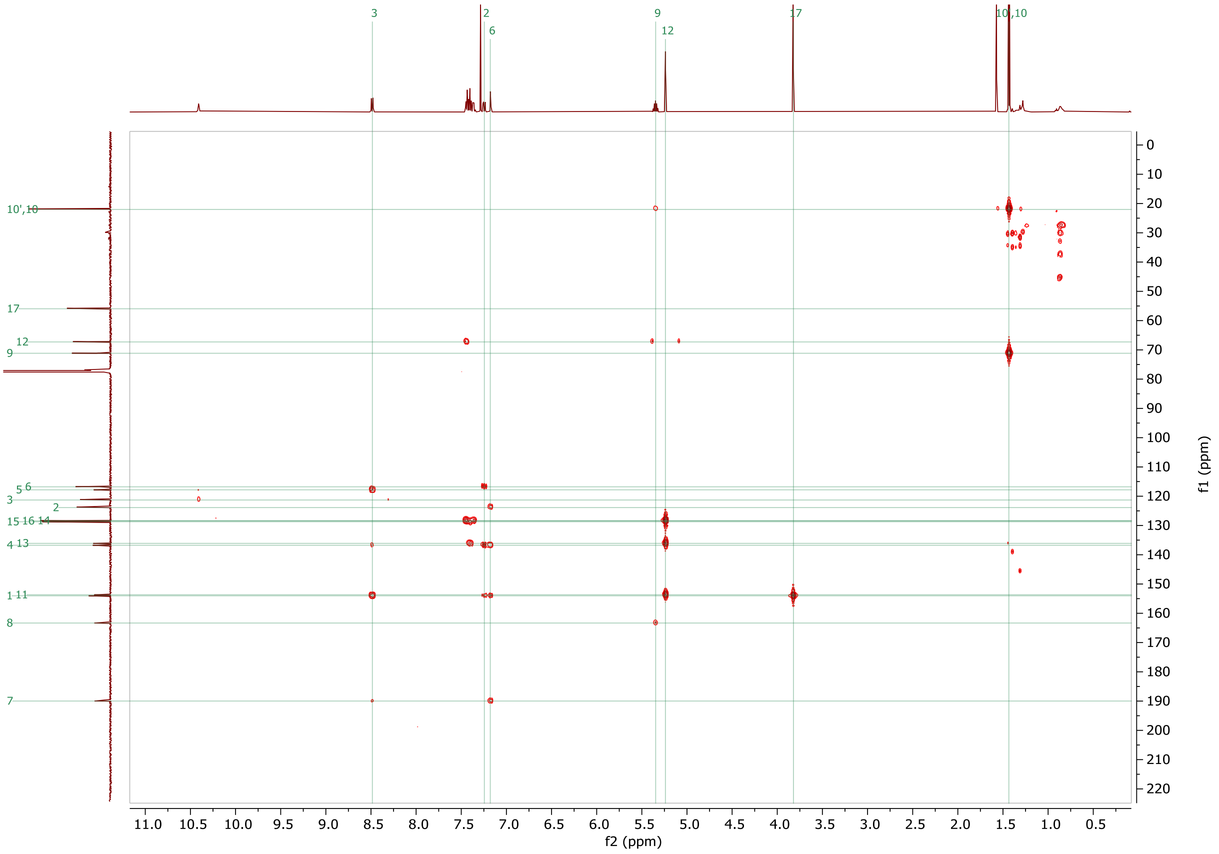

Isopropyl 2-(2-(((benzyloxy)carbonyl)amino)-4,6-dichlorophenyl)-2-oxoacetate 15j

^{1}H NMR (500 MHz, $CDCl_3$)

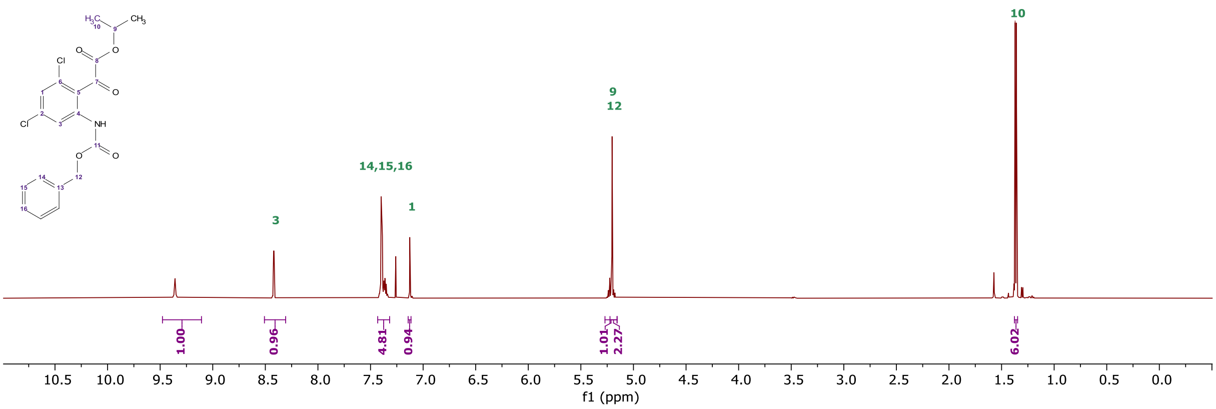

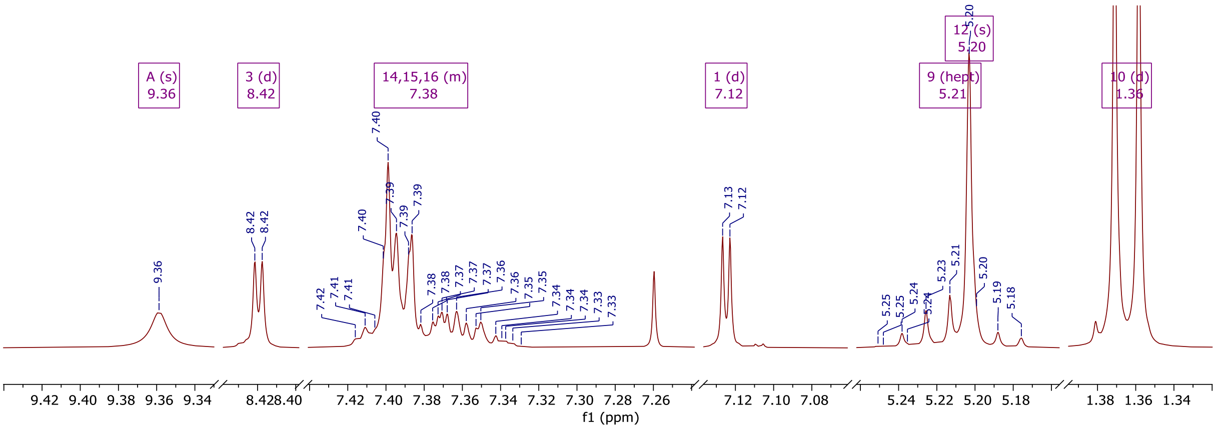

^{13}C NMR (126 MHz, $CDCl_3$)

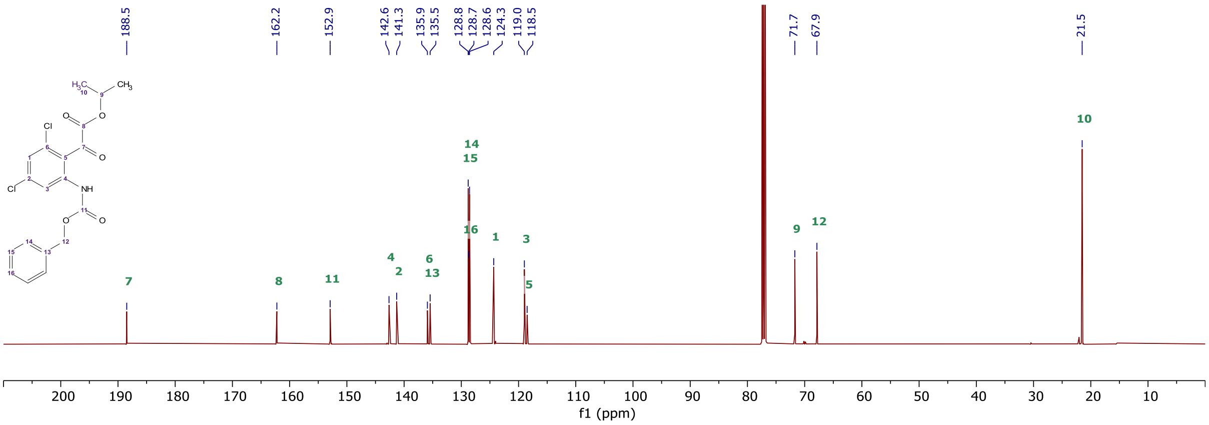

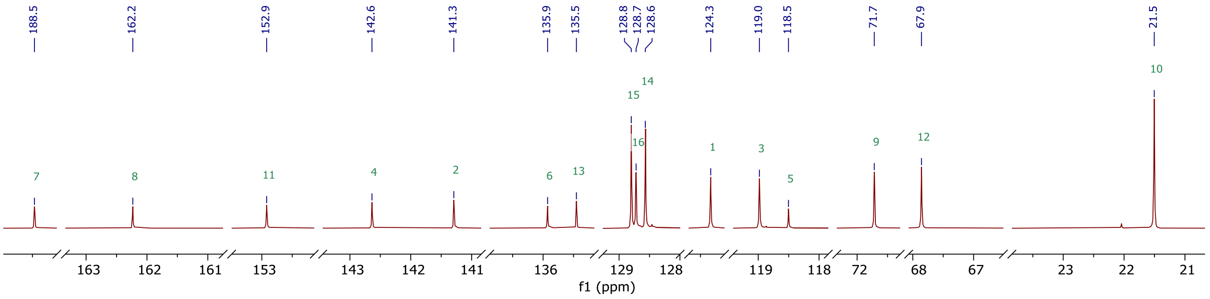

$^1H/^{13}C$ HSQC

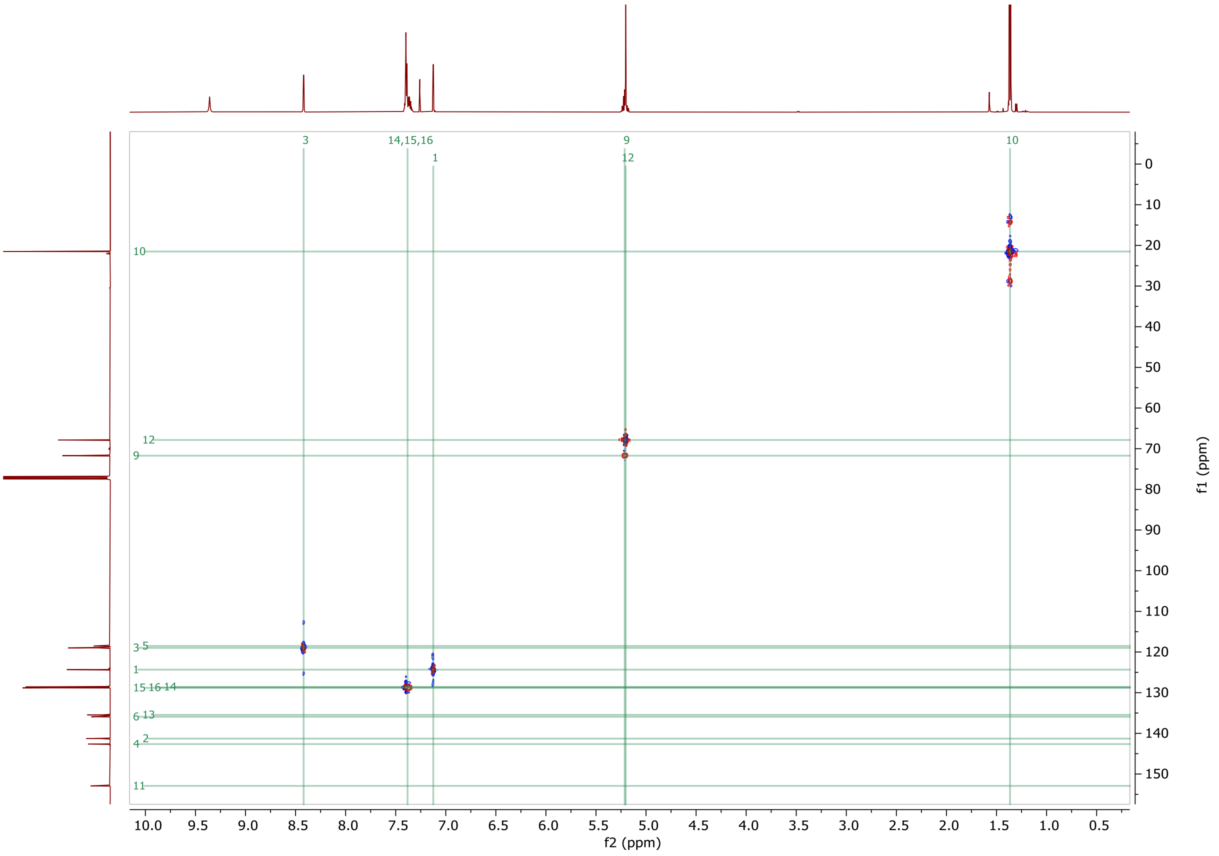

$^1H/^{13}C$ HMBC

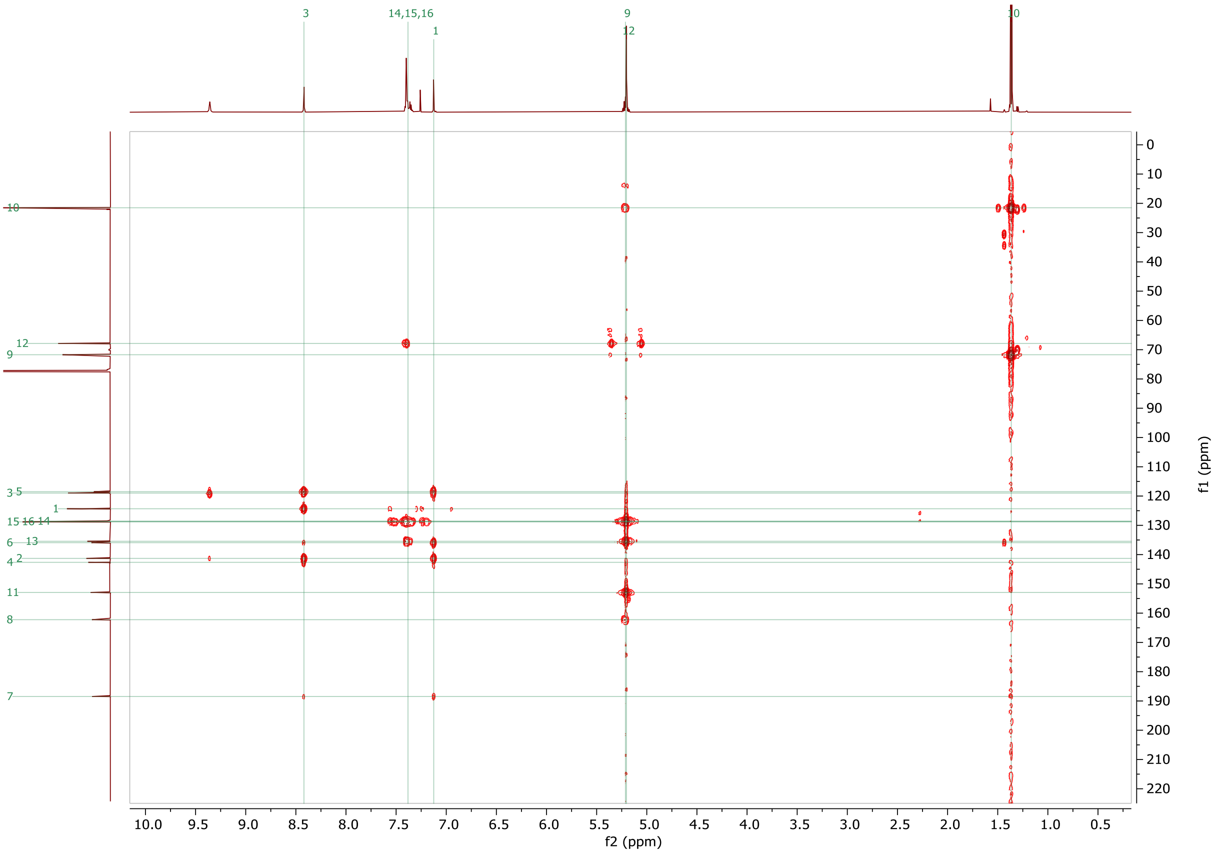

(*E*)-3-(2-Bromophenyl)acrylaldehyde 16r

^{1}H NMR (600 MHz, $CDCl_3$)

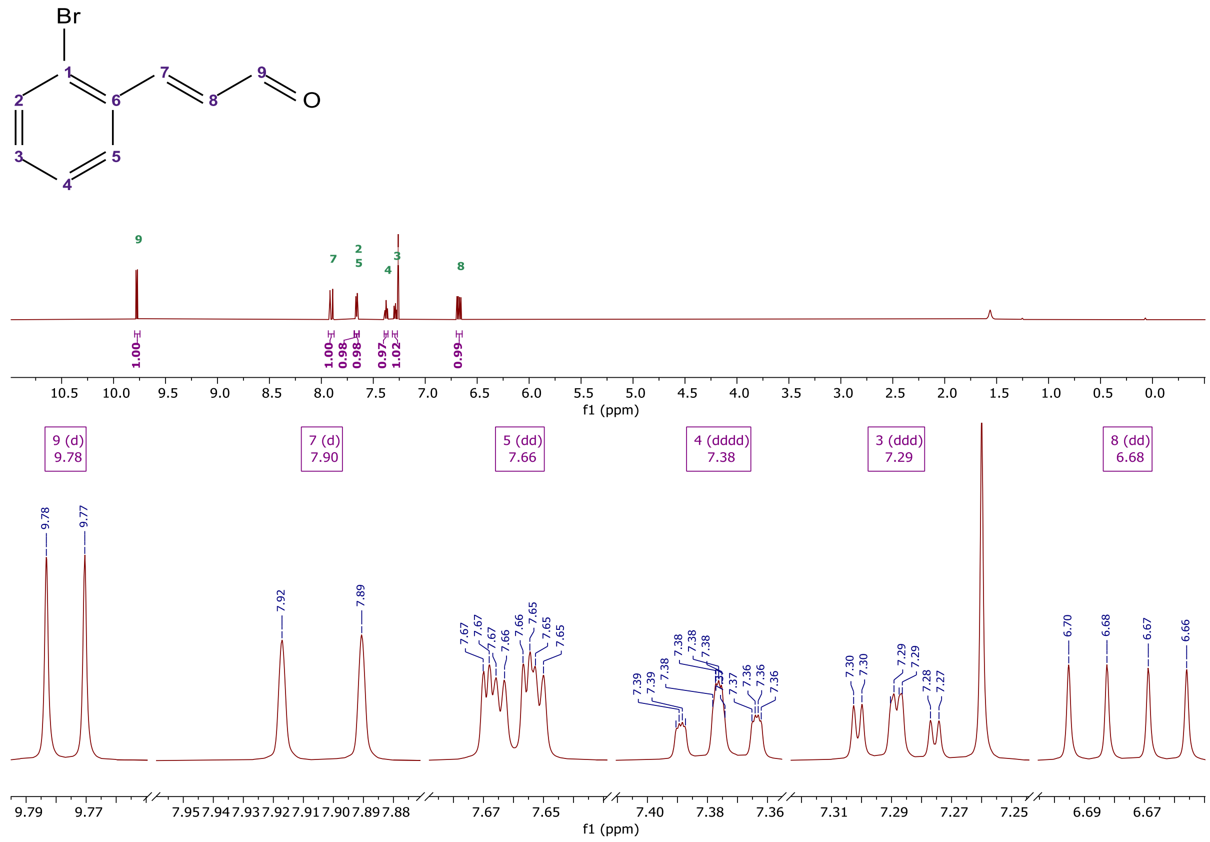

^{13}C NMR (151 MHz, $CDCl_3$)

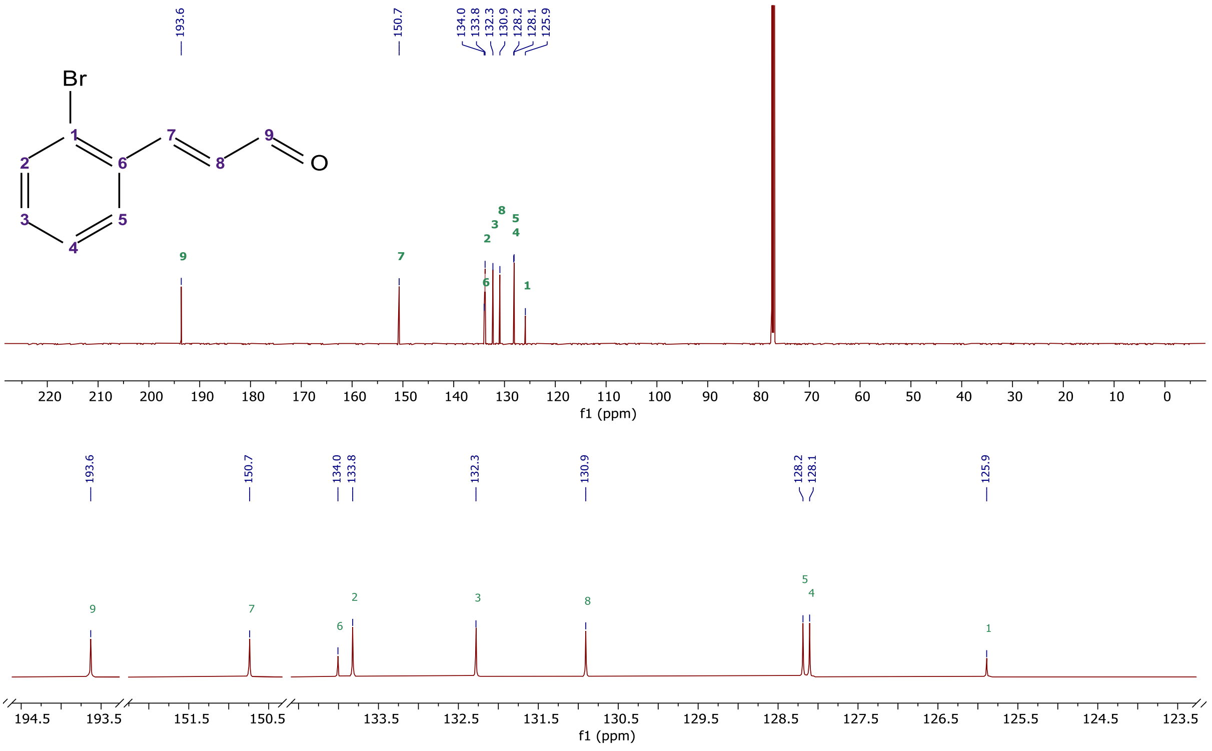

$^{1}H/^{1}H$ COSY

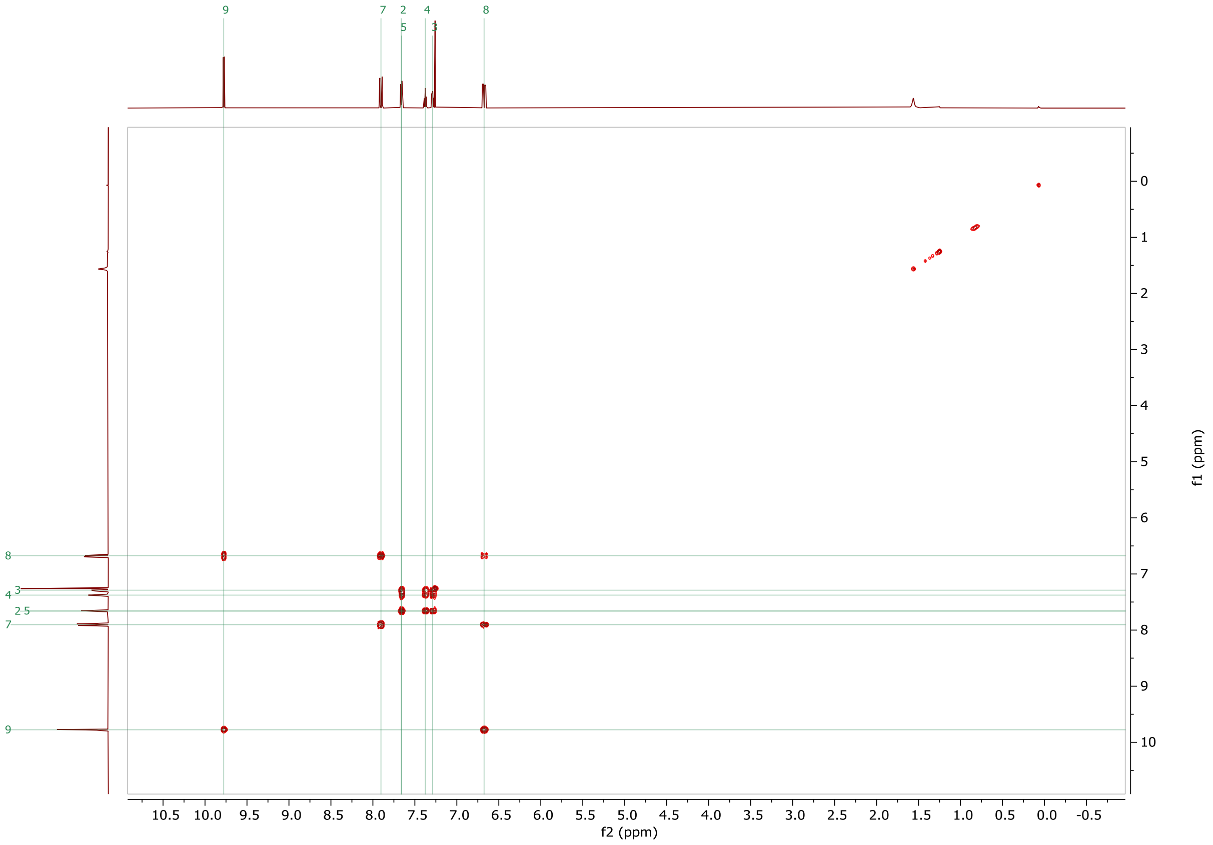

$^{1}H/^{13}C$ HSQC

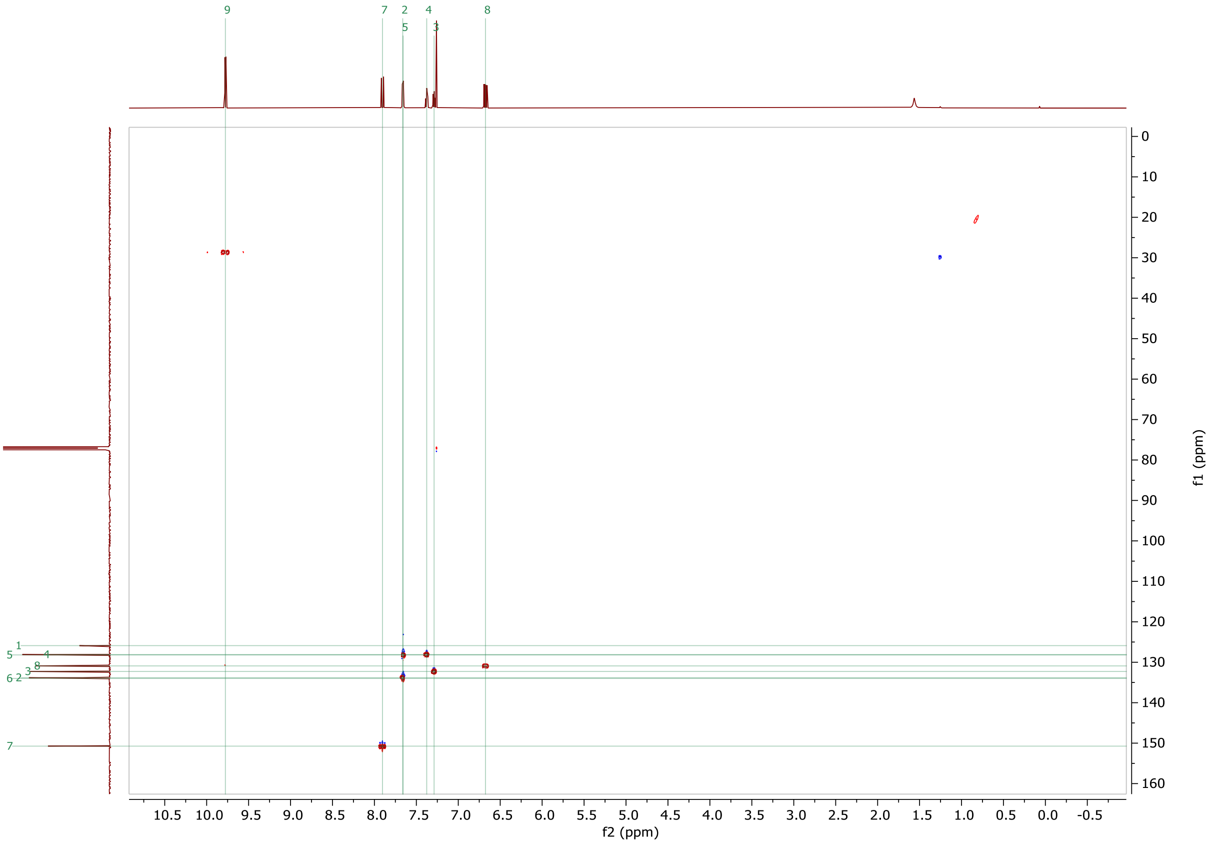

$^{1}H/^{13}C$ HMBC

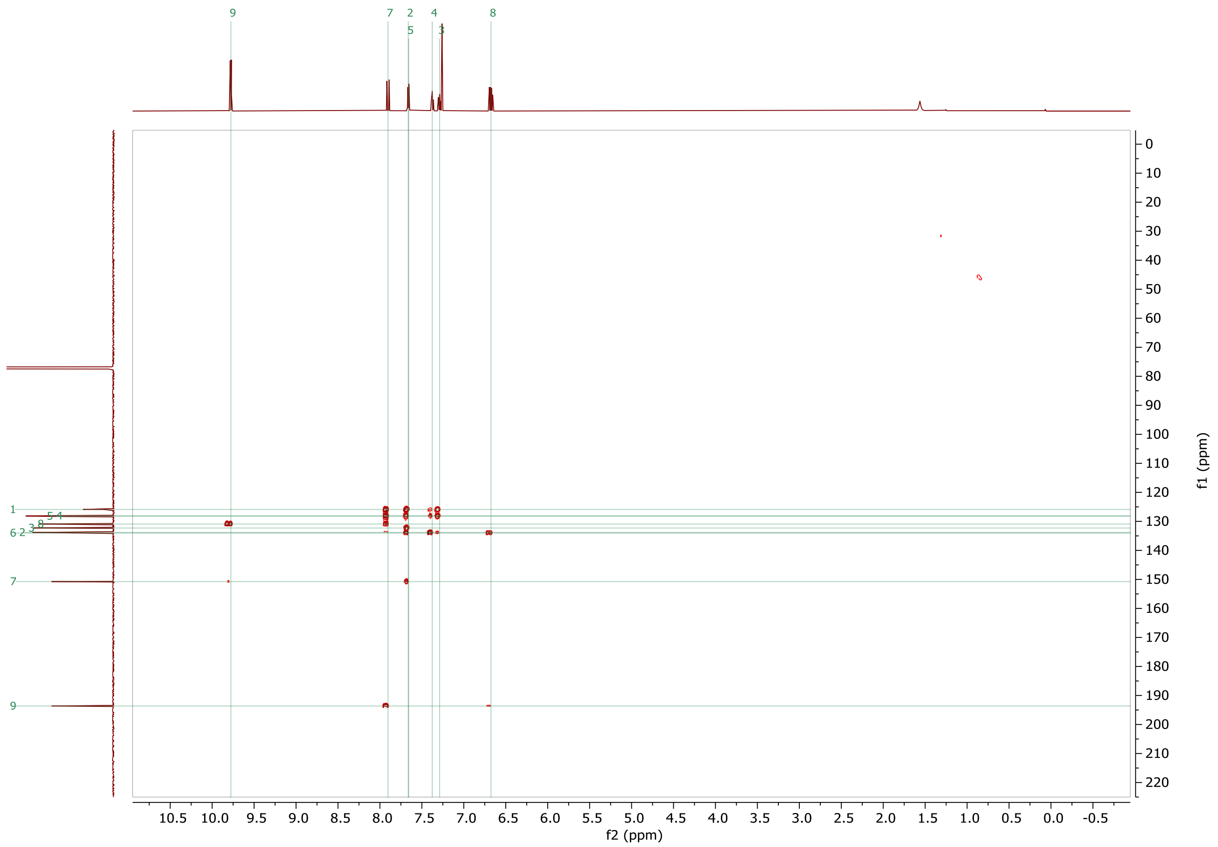

Dihydroquinoline synthesis

1-Benzyl 4-isopropyl (*R*)-3-formyl-2-phenylquinoline-1,4(2H)-dicarboxylate (–)-18a

^{1}H NMR (500 MHz, CDCl$_3$)

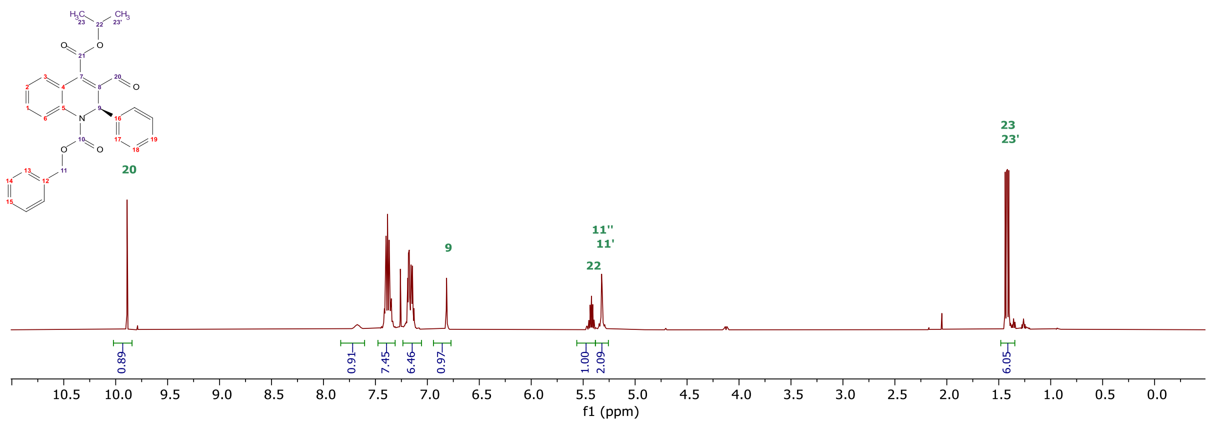

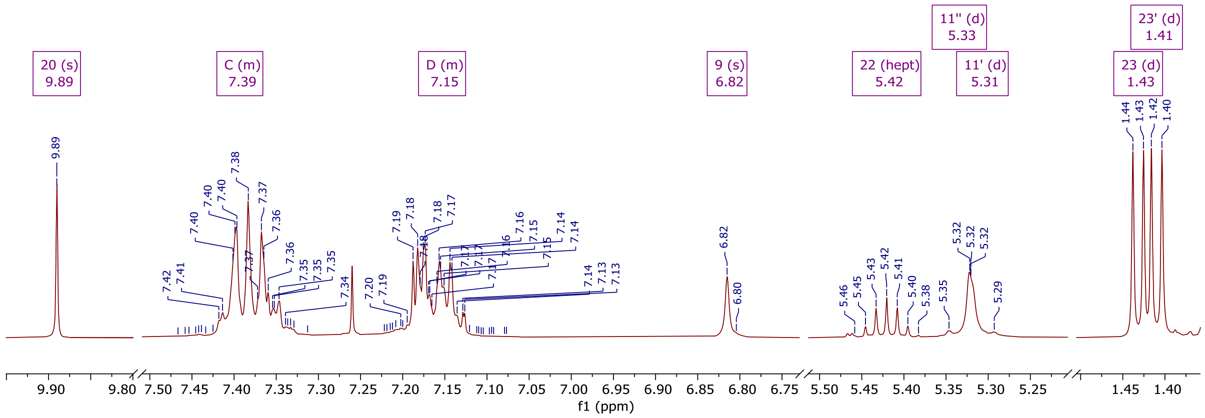

^{13}C NMR (126 MHz, CDCl$_3$)

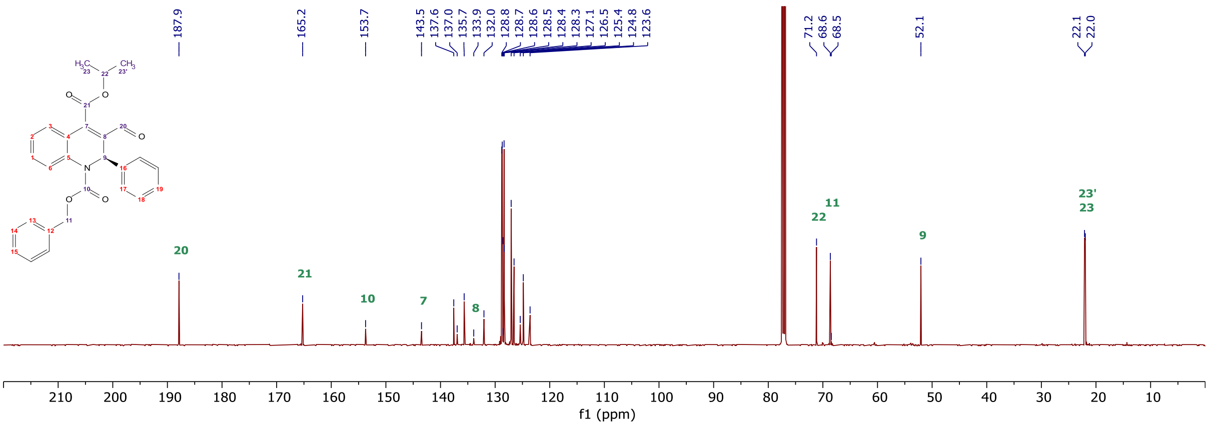

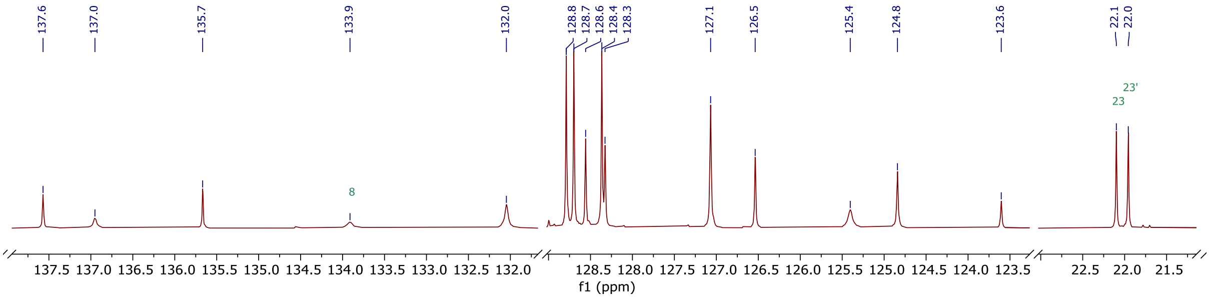

$^{1}H/^{1}H$ COSY

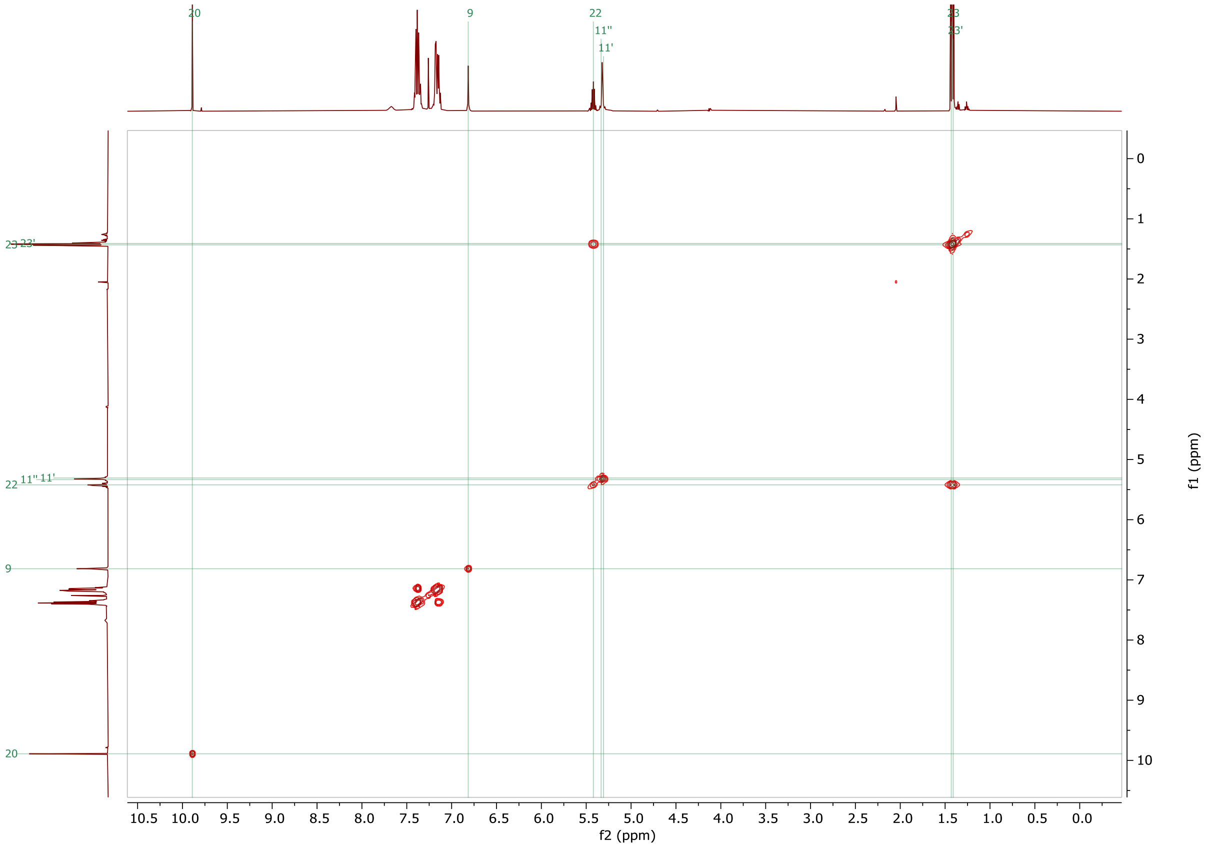

$^{1}H/^{13}C$ HSQC

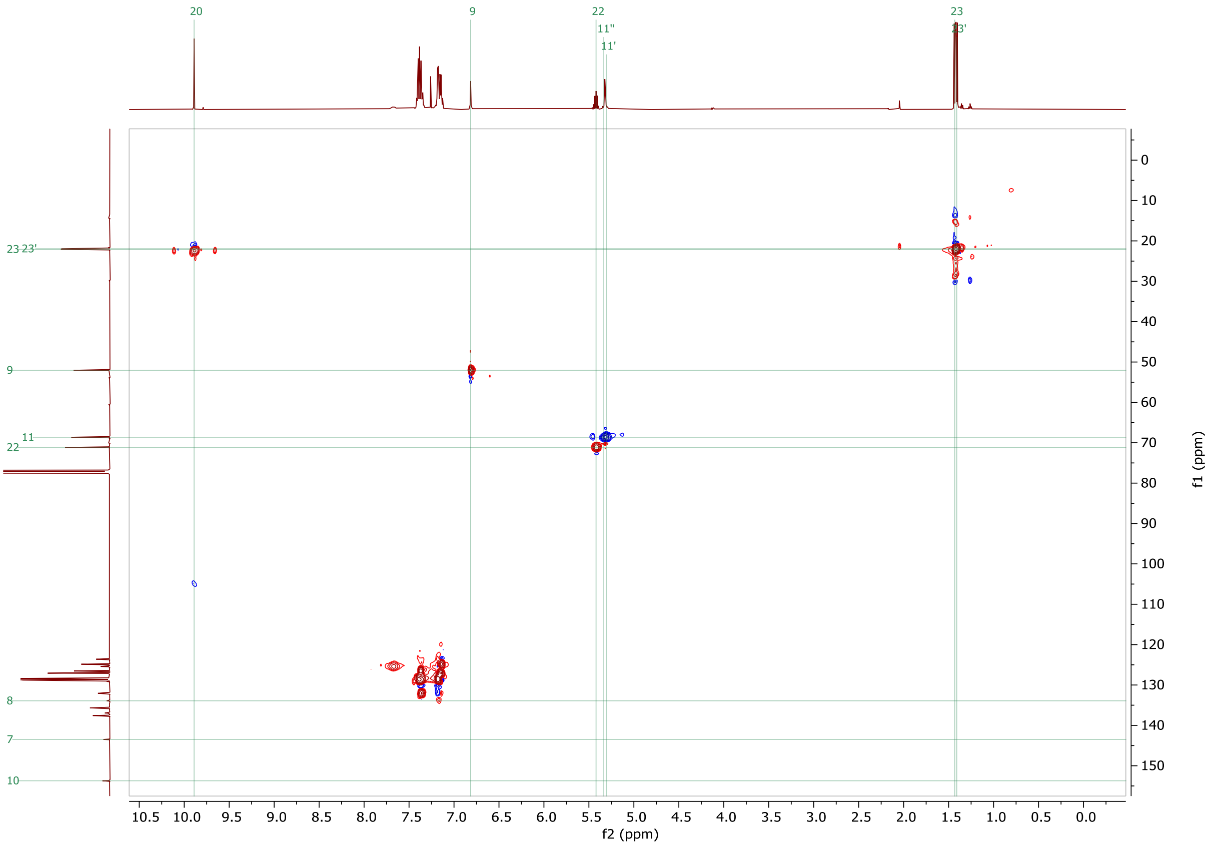

$^{1}H/^{13}C$ HMBC

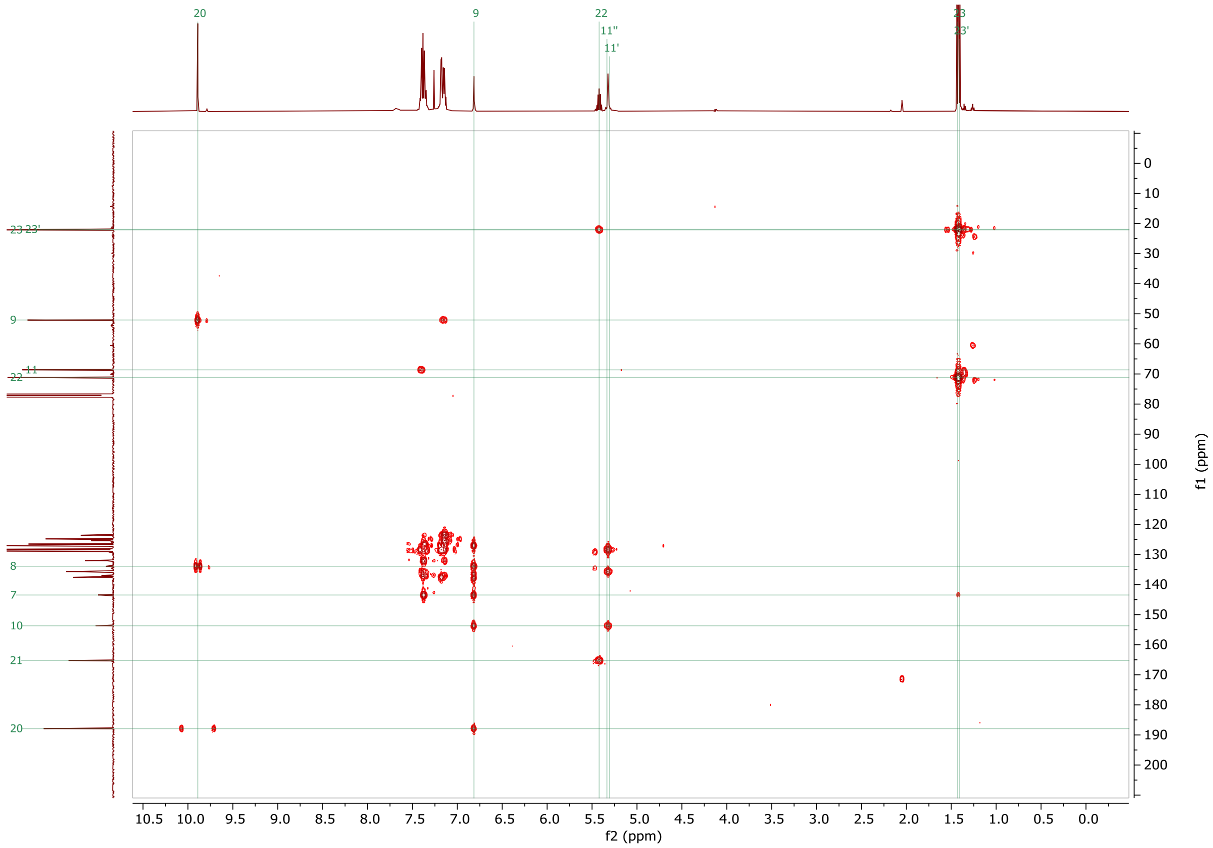

1-Benzyl 4-isopropyl (2*S*,3*R*,4*S*)-3-formyl-4-hydroxy-2-phenyl-3,4-dihydroquinoline-1,4(2H)-dicarboxylate (–)-19a

^{1}H NMR (500 MHz, CDCl$_3$)

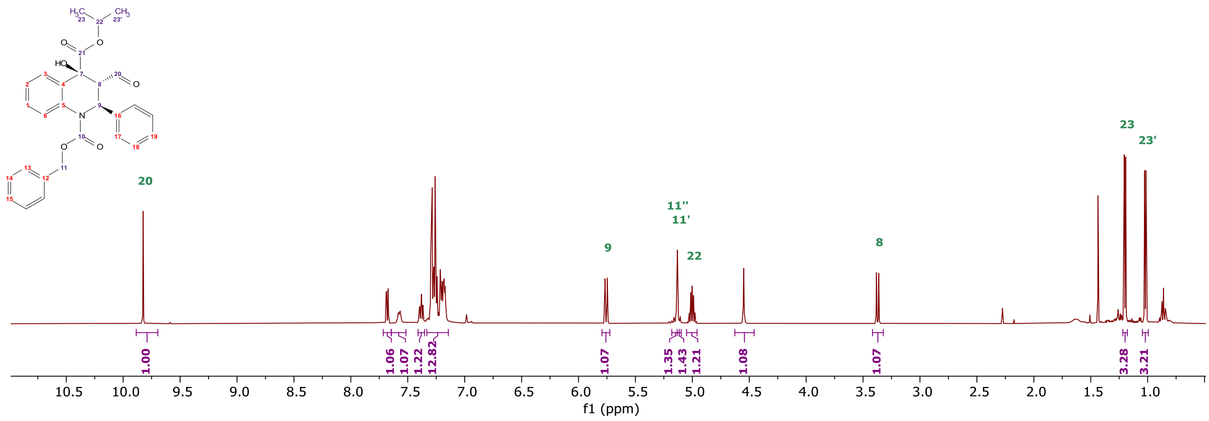

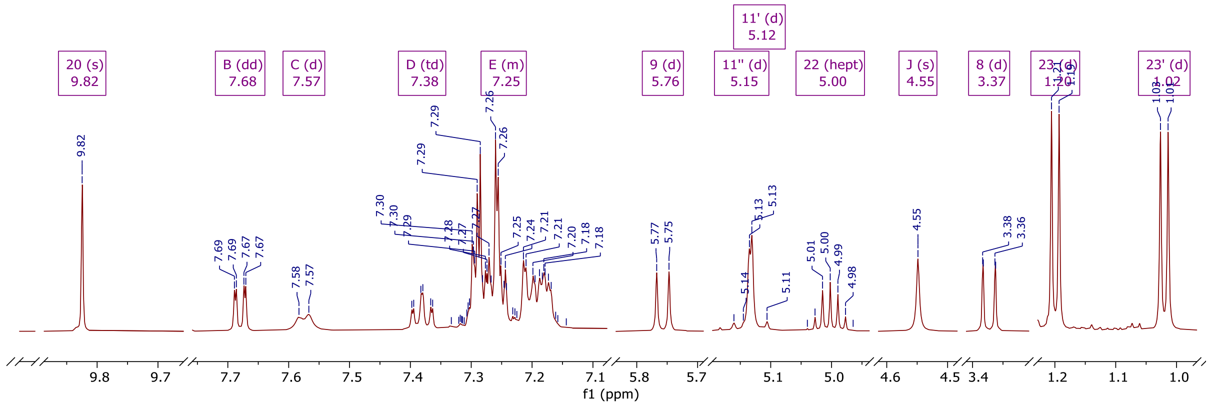

^{13}C NMR (126 MHz, CDCl$_3$)

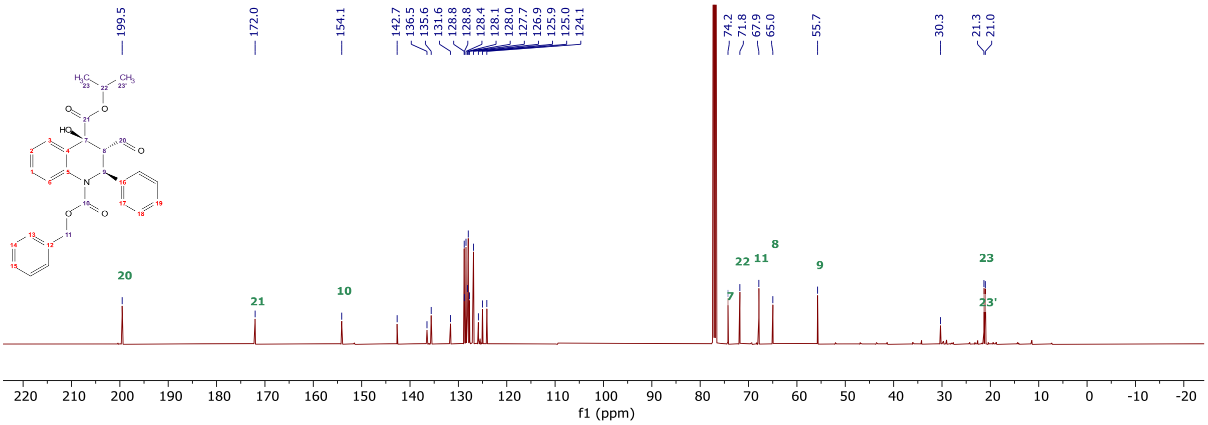

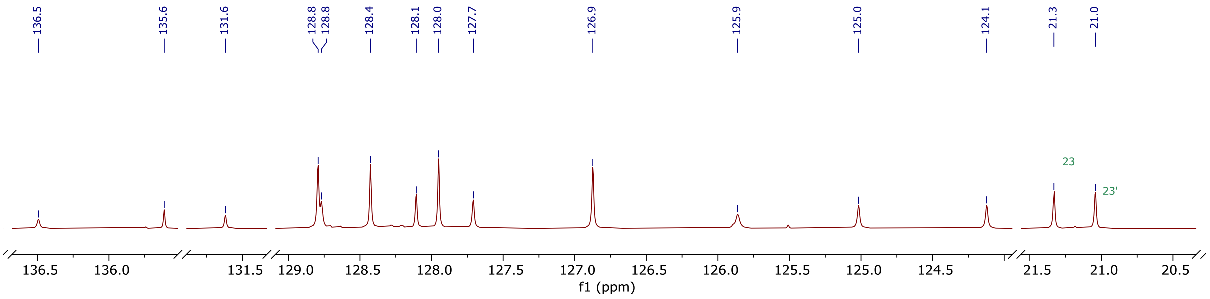

$^1H/^1H$ COSY

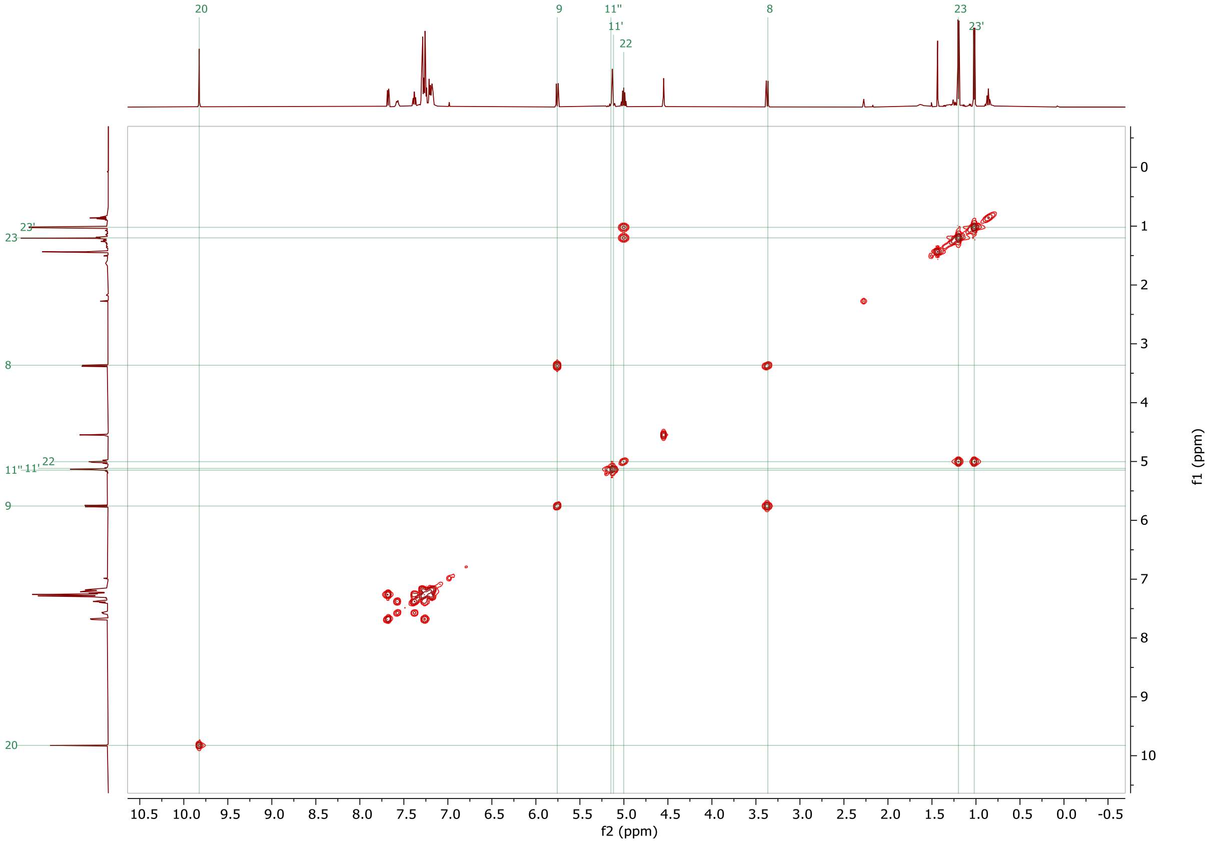

$^1H/^{13}C$ HSQC

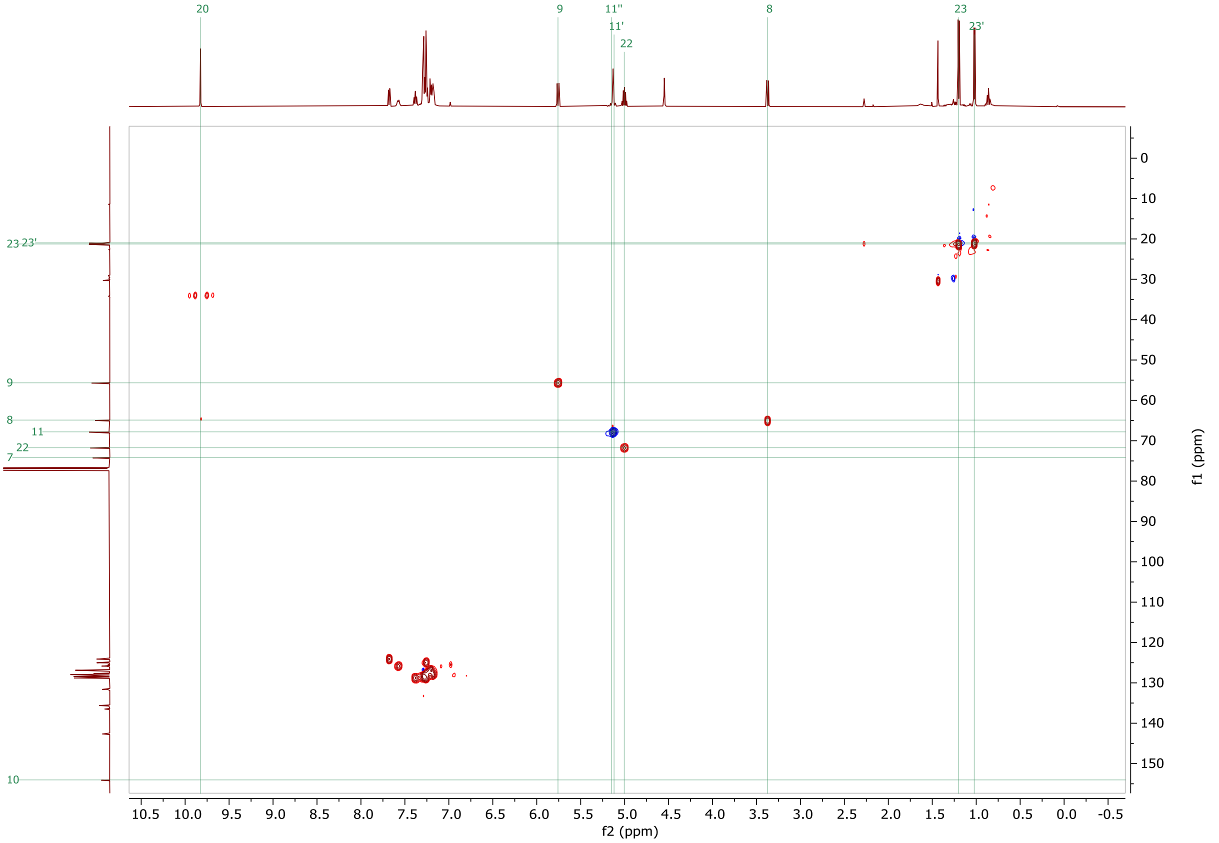

$^{1}H/^{13}C$ HMBC

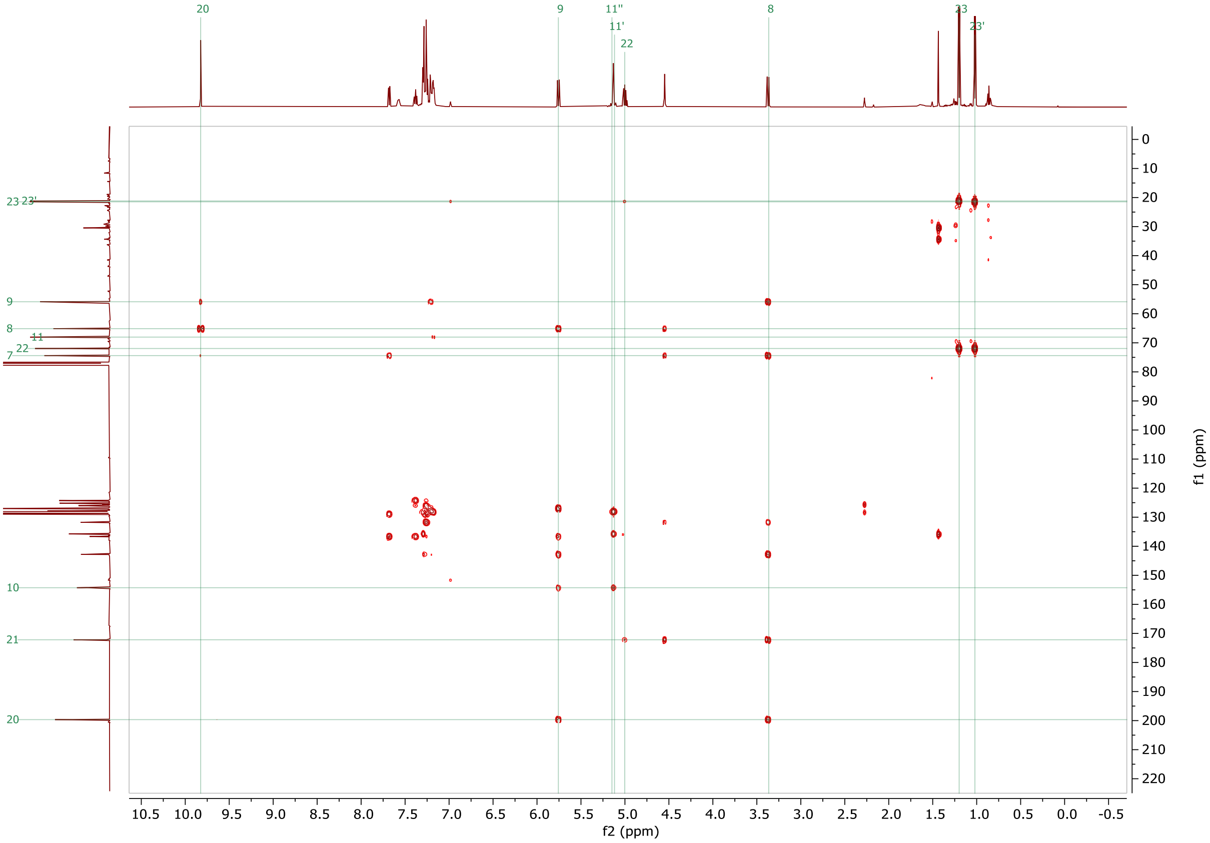

$^{1}H/^{1}H$ NOESY – racemic material

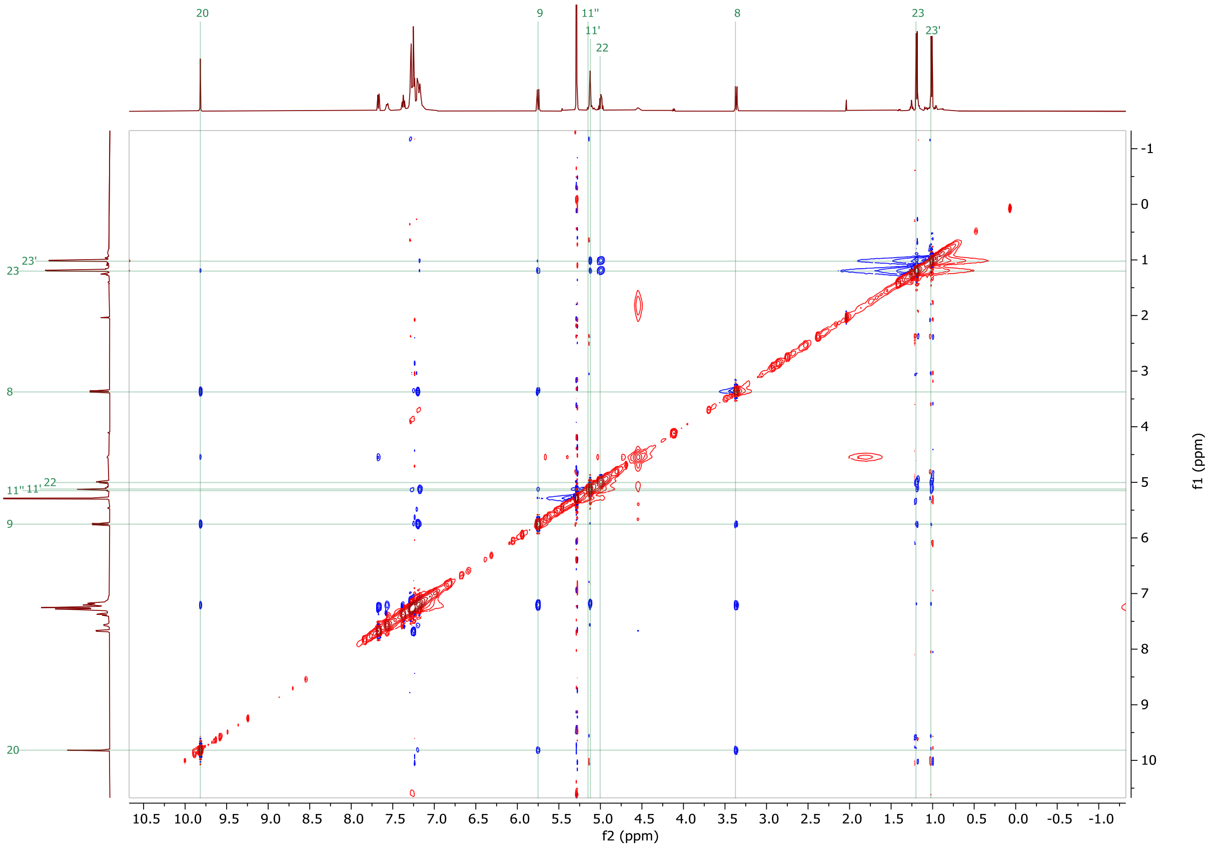

1-Benzyl 4-methyl (*R*)-3-formyl-2-phenylquinoline-1,4(2H)-dicarboxylate (–)-18b

^{1}H NMR (500 MHz, $CDCl_3$)

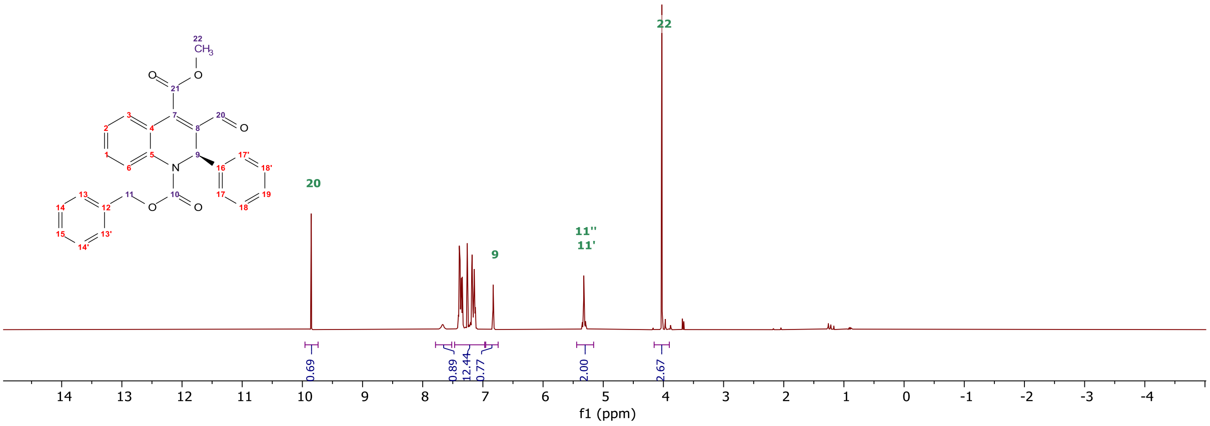

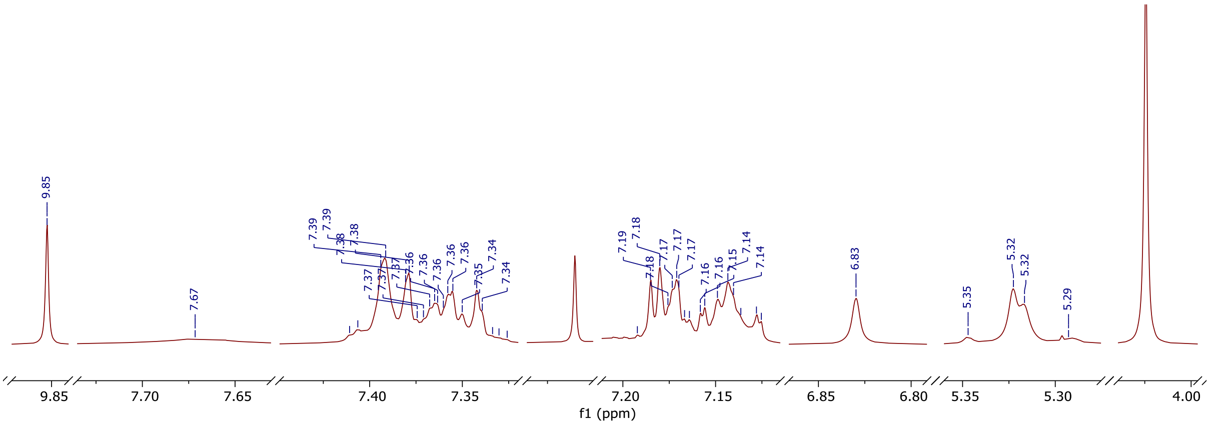

^{13}C NMR (126 MHz, $CDCl_3$)

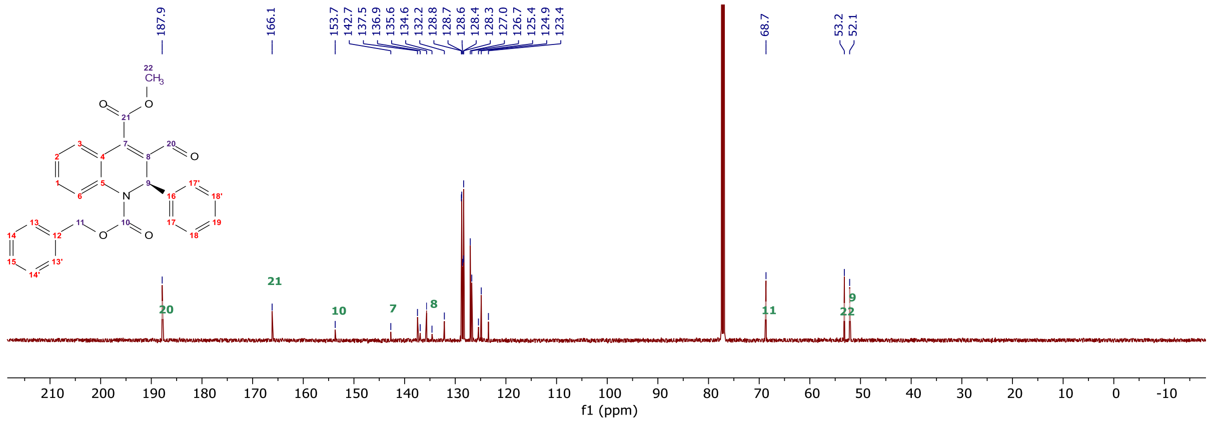

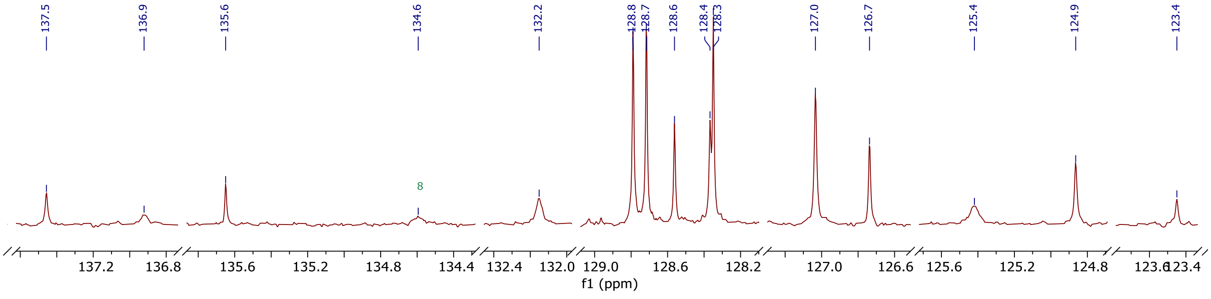

$^1H/^1H$ COSY

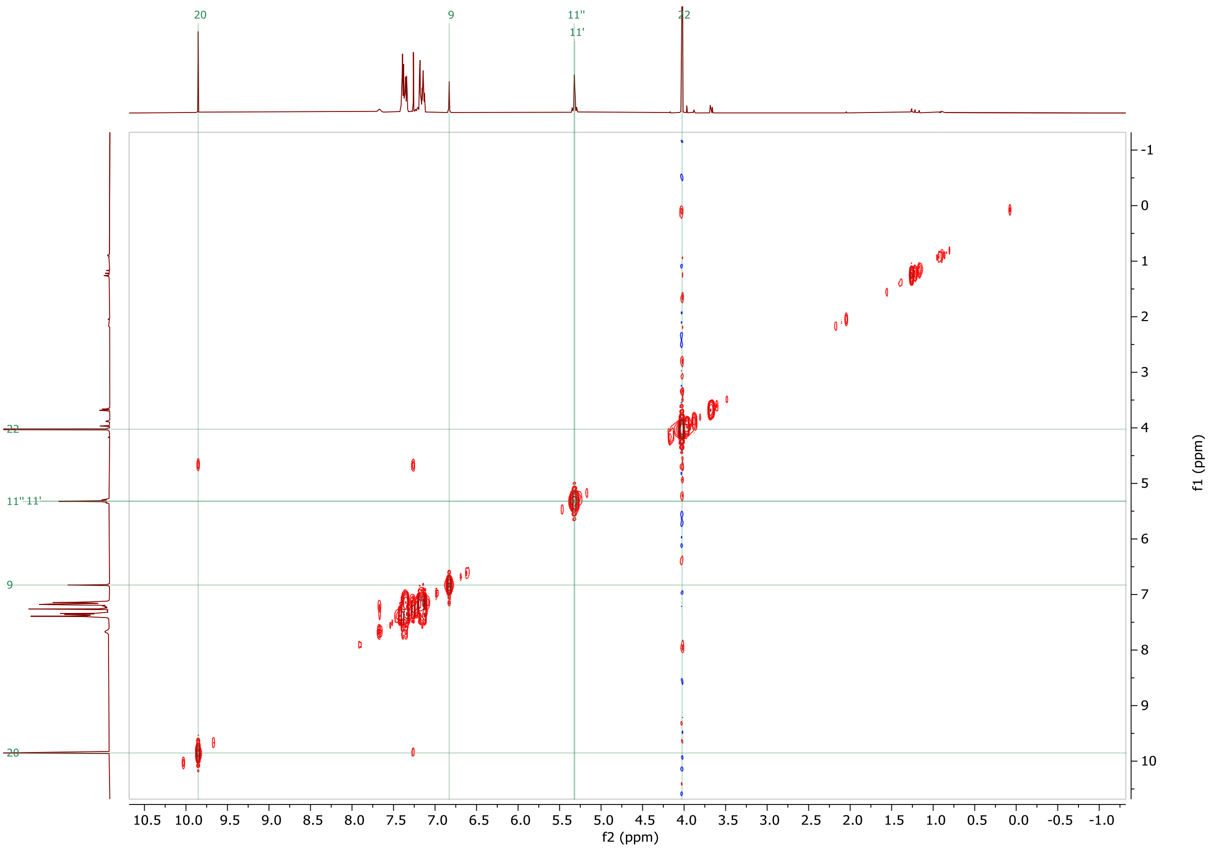

$^1H/^{13}C$ HSQC

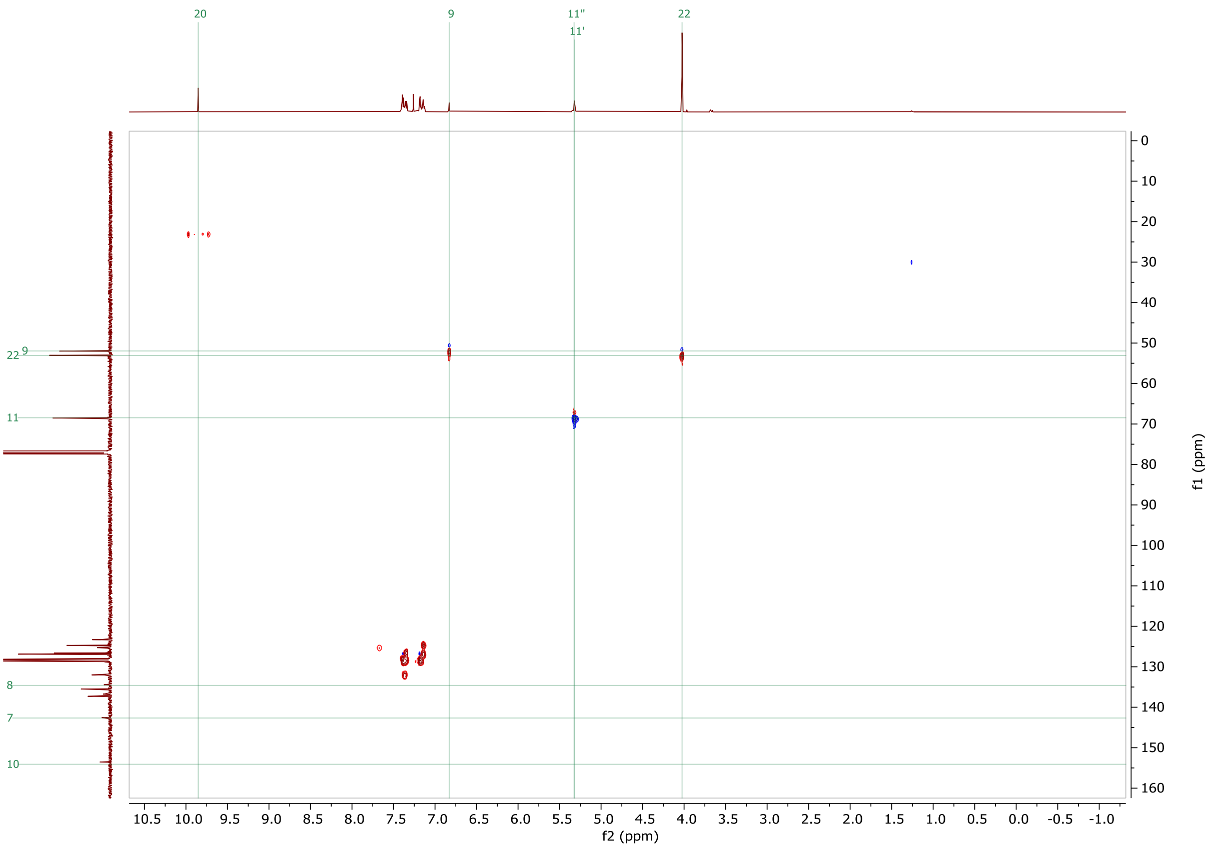

$^{1}H/^{13}C$ HMBC

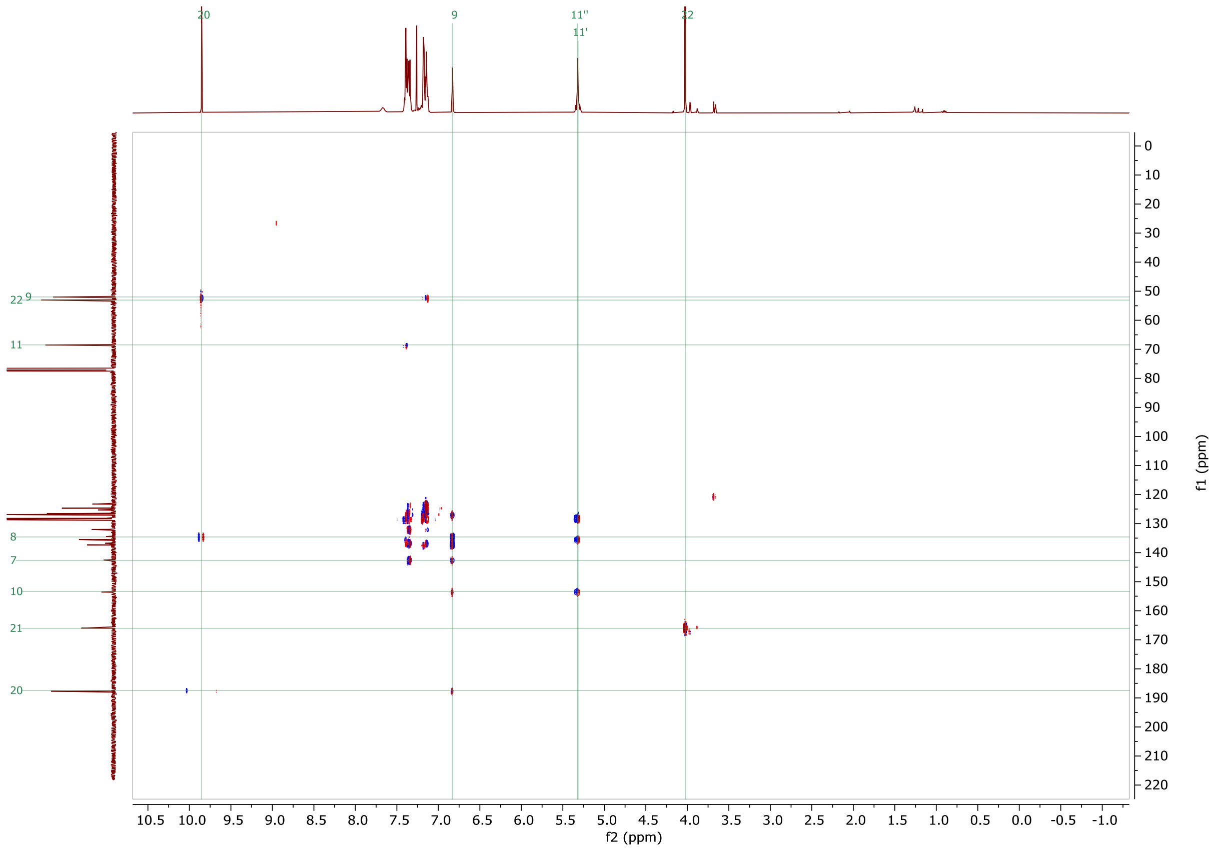

4-Isopropyl 1-methyl (*R*)-3-formyl-2-phenylquinoline-1,4(2H)-dicarboxylate (–)-18c

^{1}H NMR (500 MHz, $CDCl_3$)

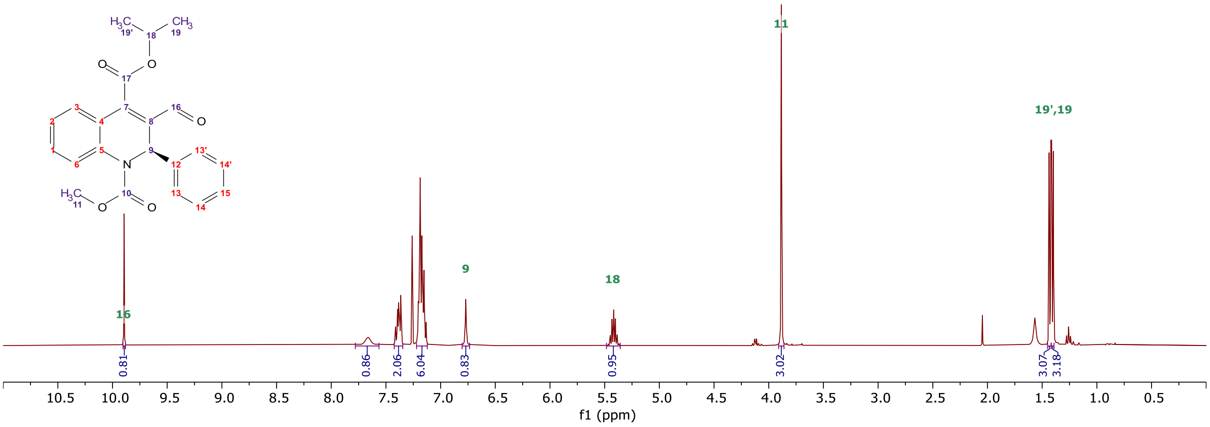

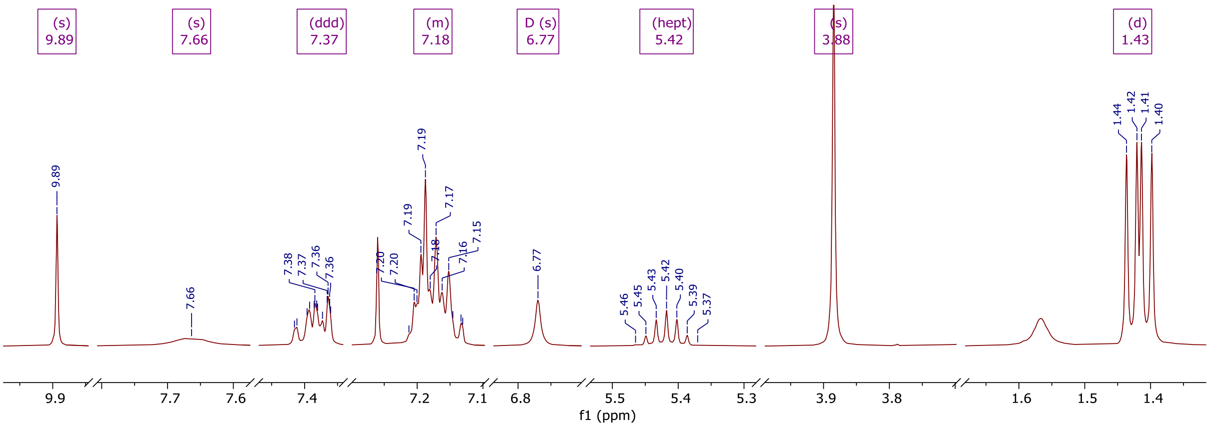

^{13}C NMR (126 MHz, $CDCl_3$)

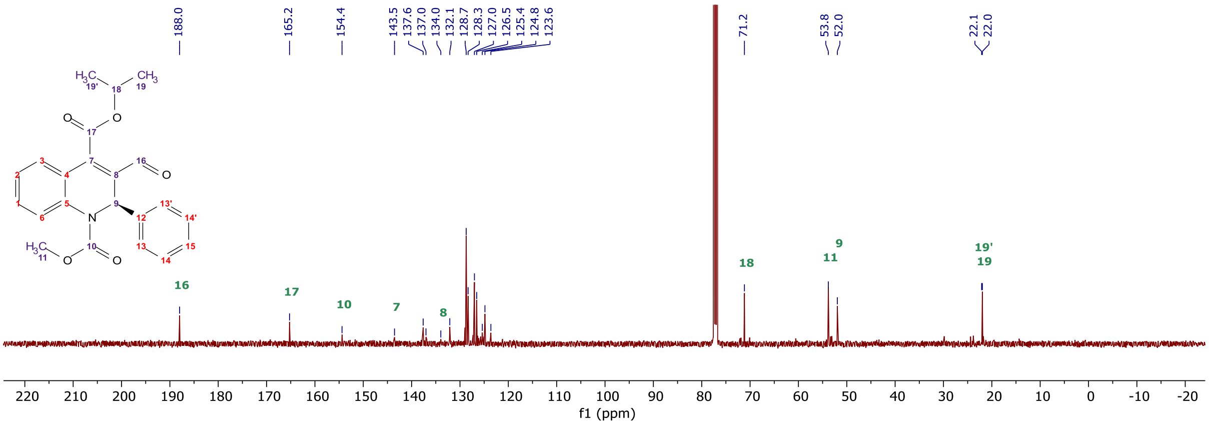

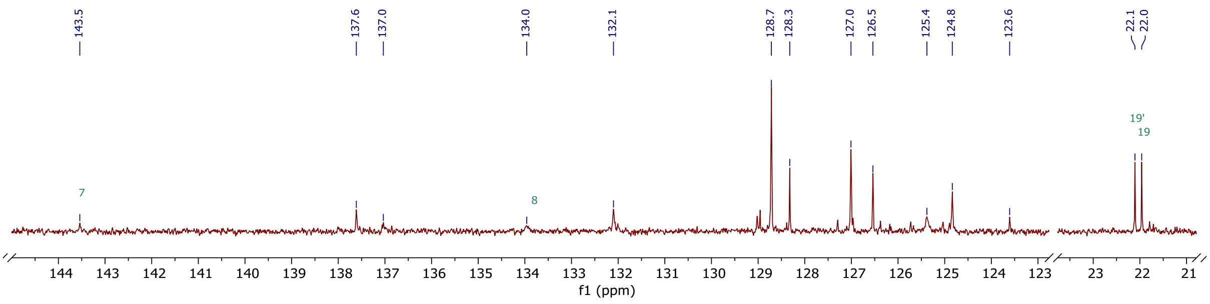

$^{1}H/^{1}H$ COSY

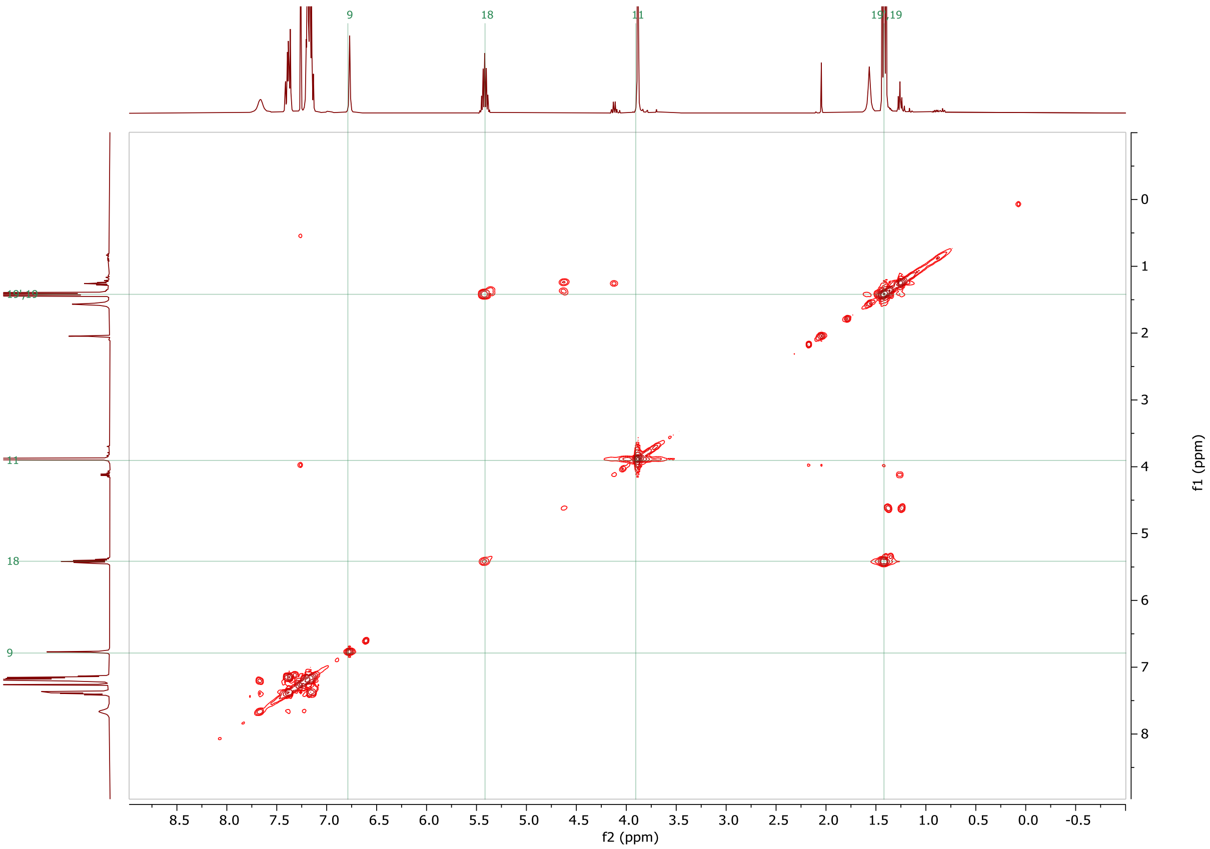

$^{1}H/^{13}C$ HSQC

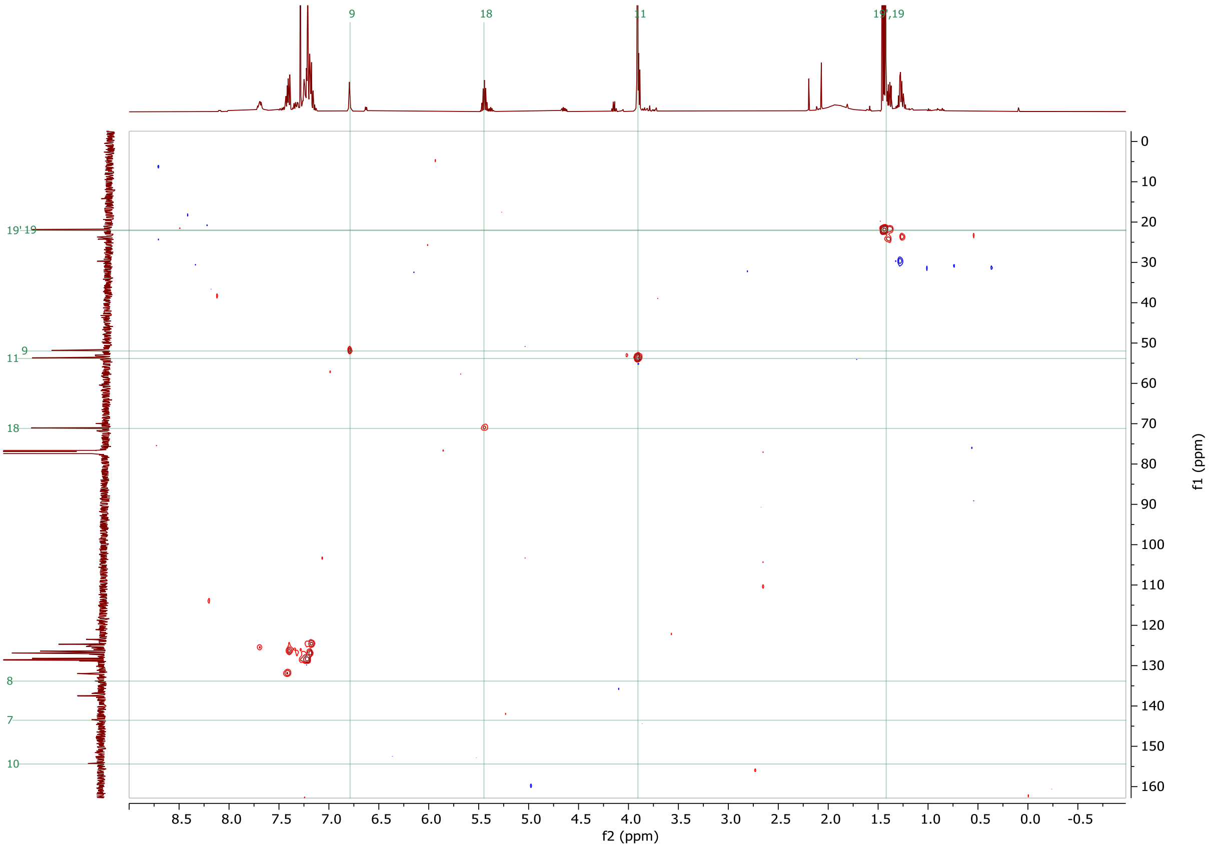

$^{1}H/^{13}C$ HMBC

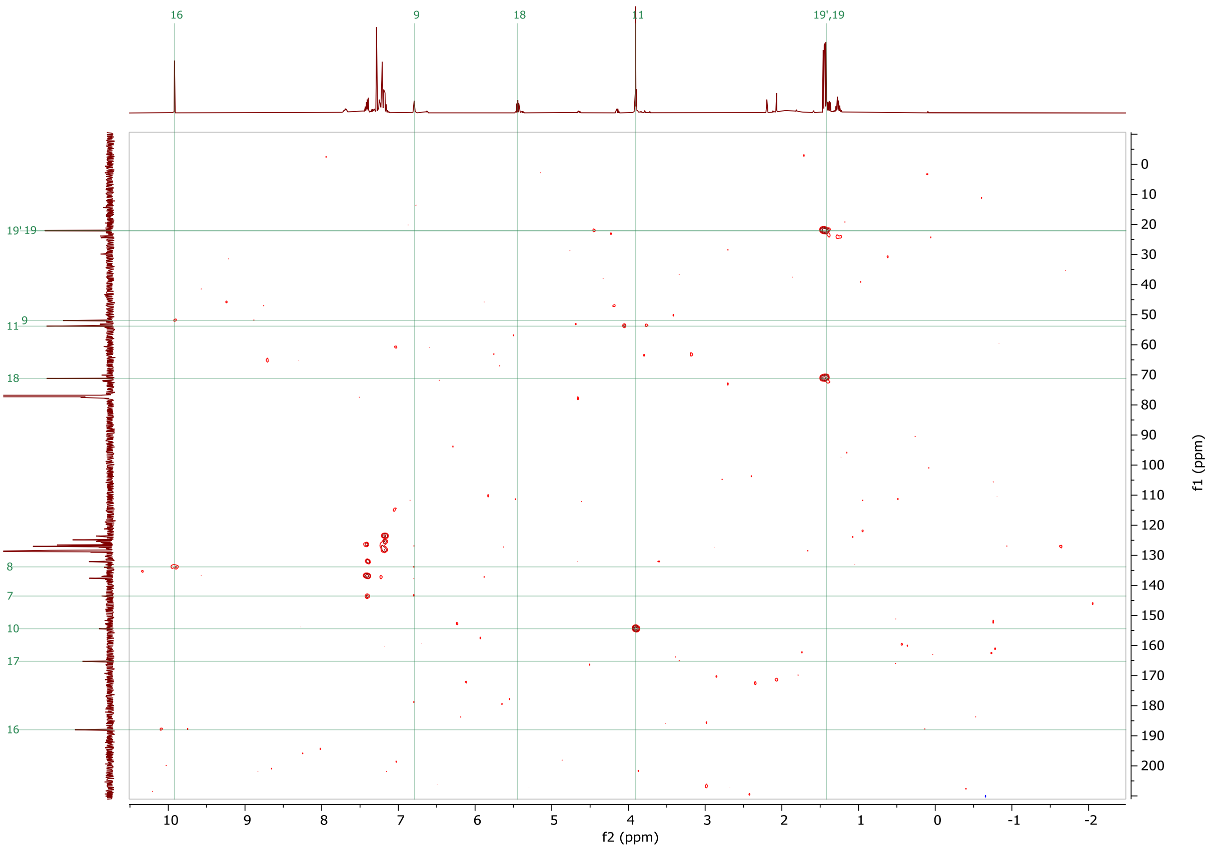

1-(*tert*-Butyl) 4-isopropyl (*R*)-3-formyl-2-phenylquinoline-1,4(2H)-dicarboxylate (–)-18d

^{1}H NMR (500 MHz, $CDCl_3$)

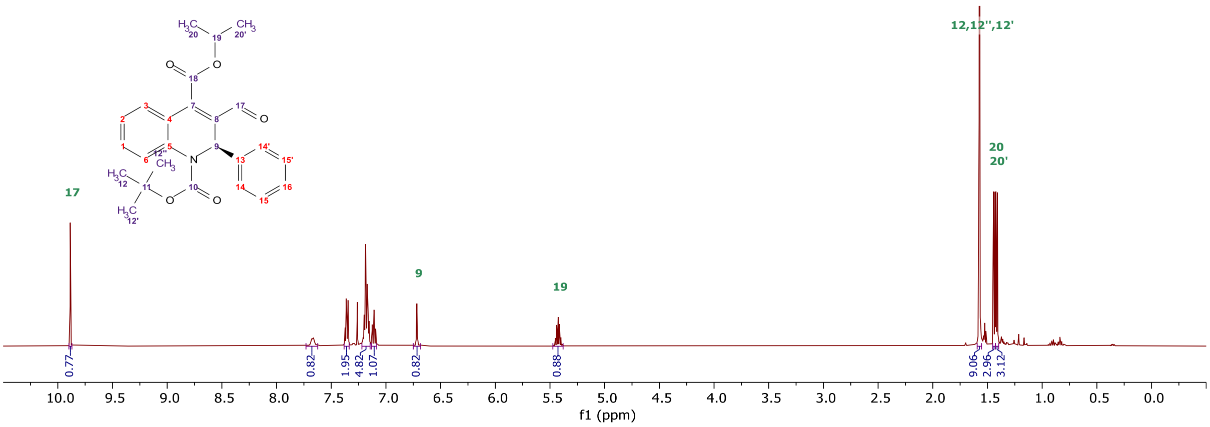

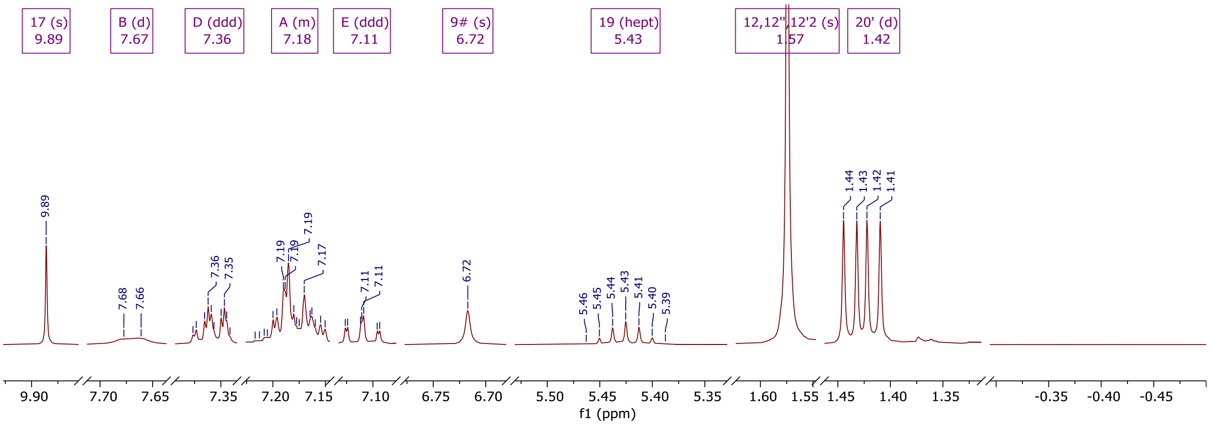

^{13}C NMR (126 MHz, $CDCl_3$)

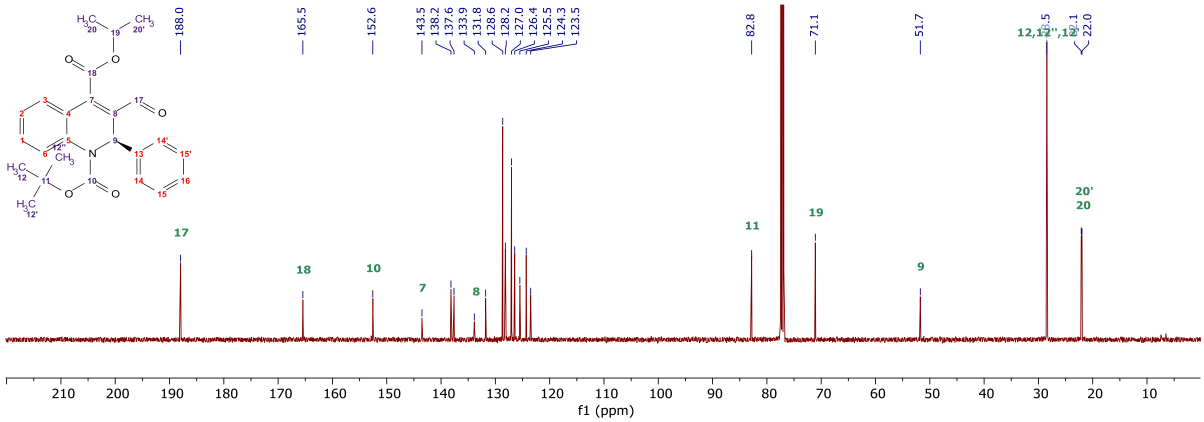

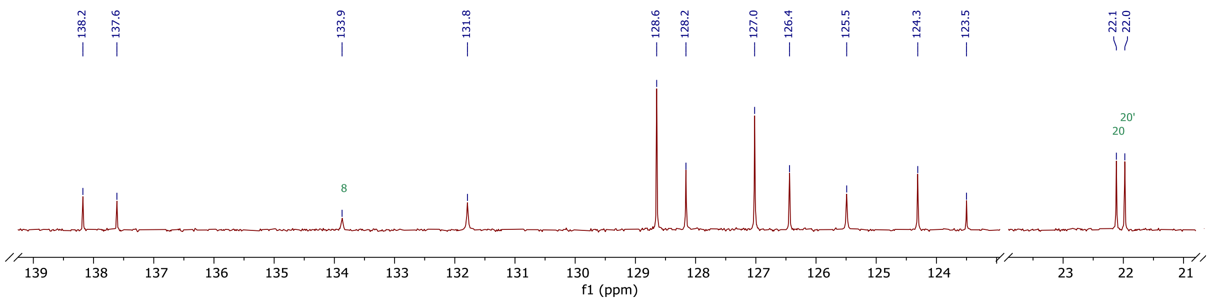

$^{1}H/^{1}H$ COSY

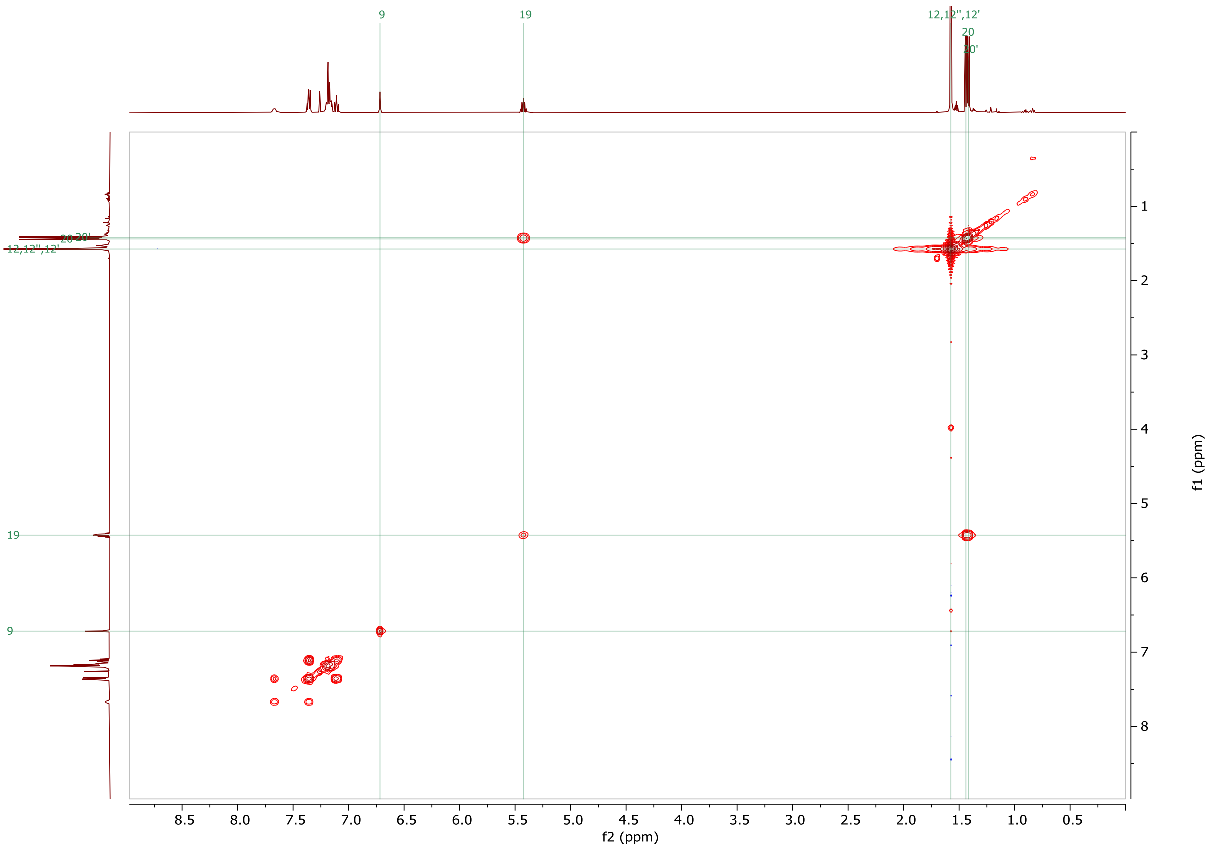

$^{1}H/^{13}C$ HSQC

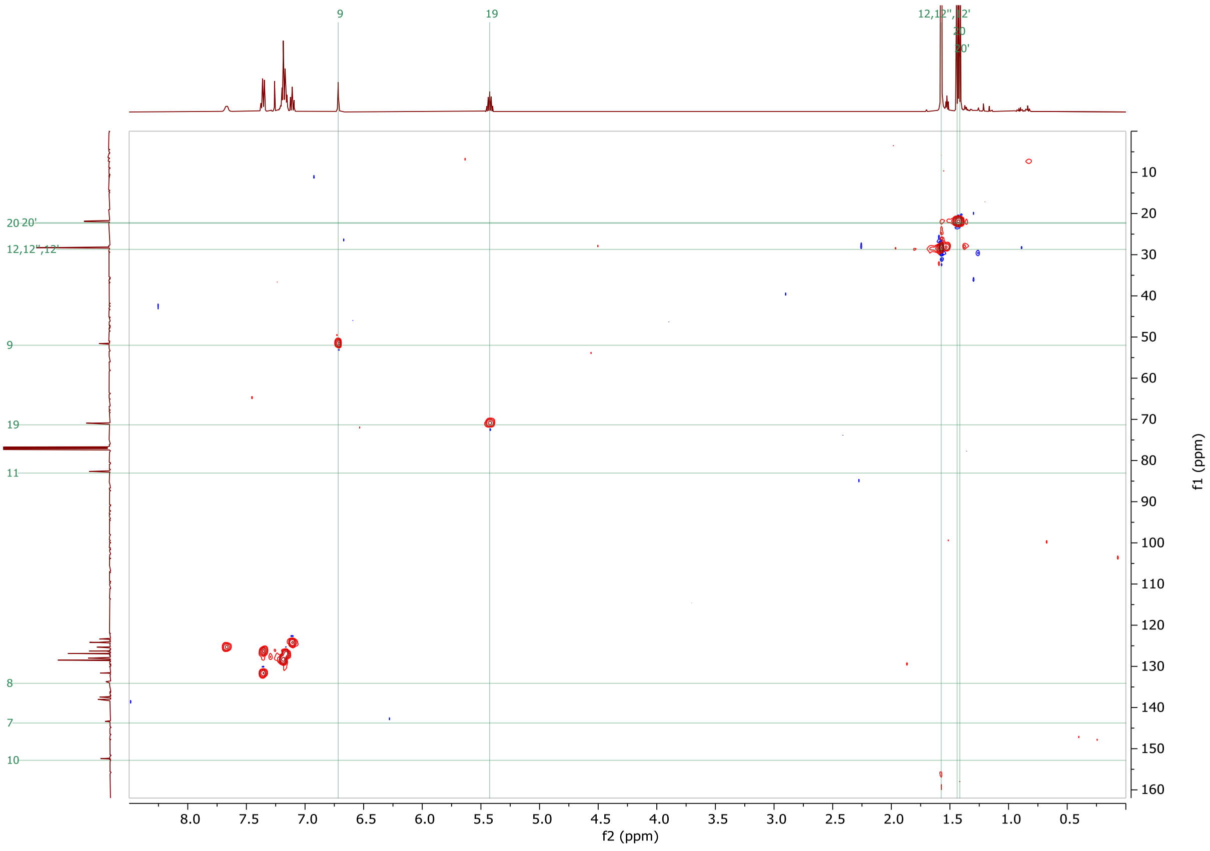

$^{1}H/^{13}C$ HMBC

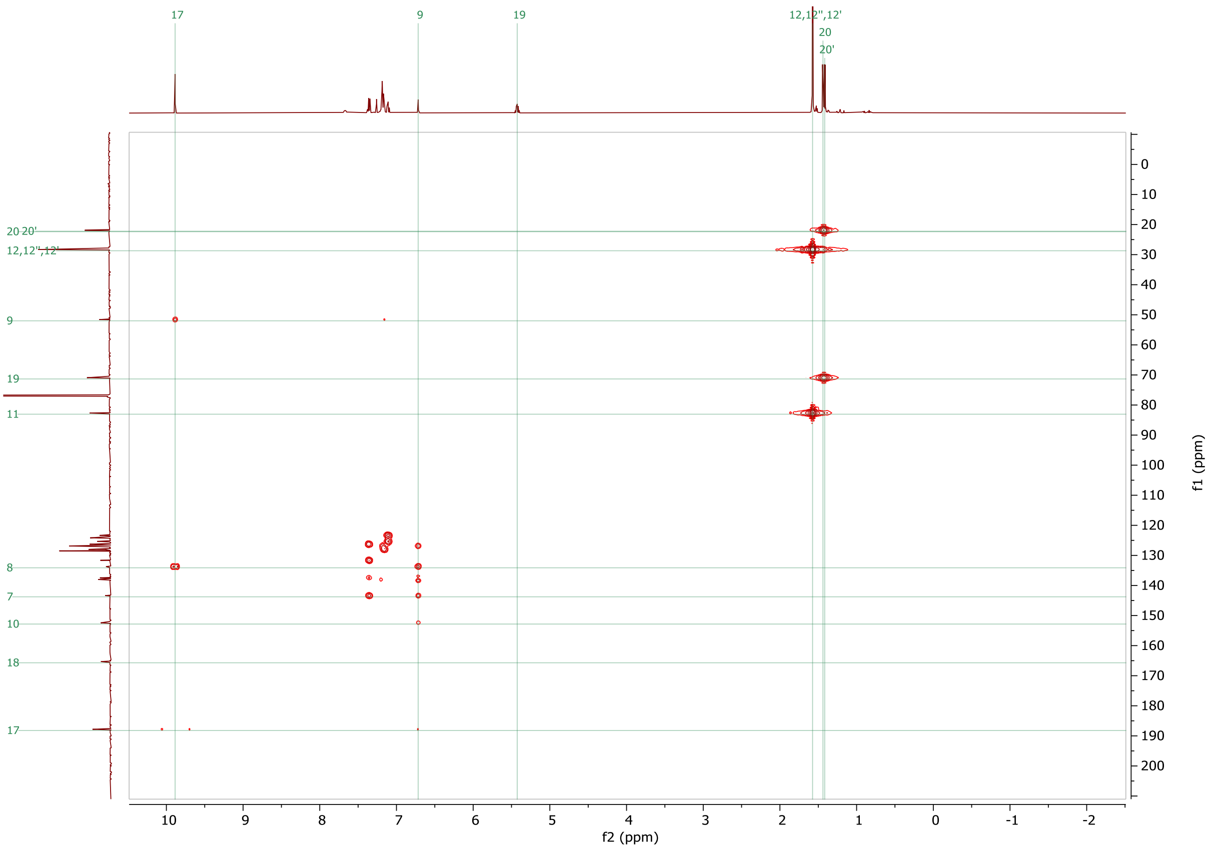

1-Benzyl 4-isopropyl (*R*)-6-chloro-3-formyl-2-phenylquinoline-1,4(2H)-dicarboxylate (–)-18e

^{1}H NMR (500 MHz, $CDCl_3$)

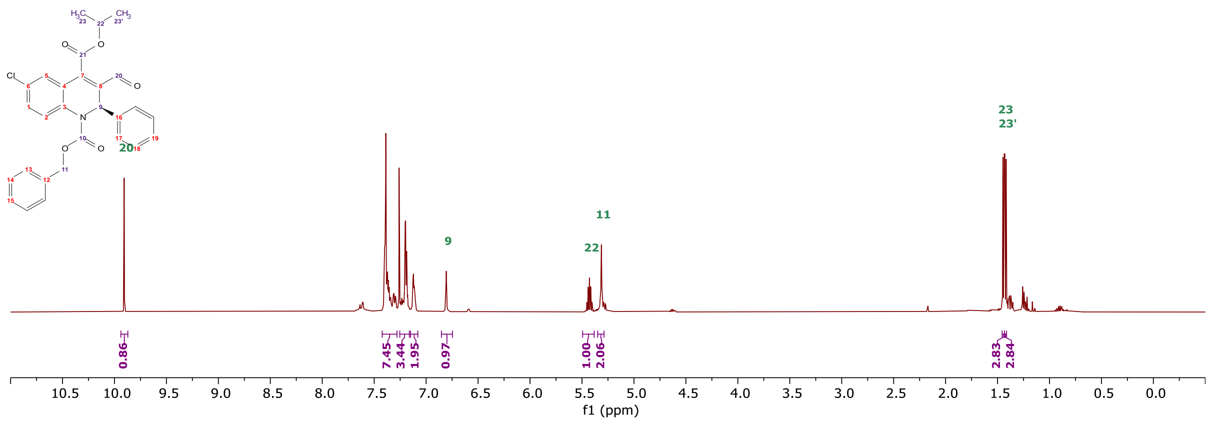

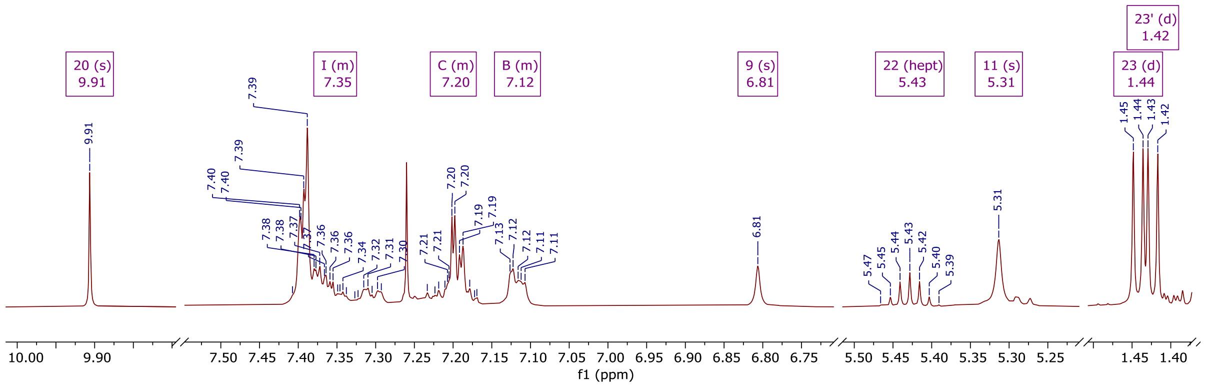

^{13}C NMR (126 MHz, $CDCl_3$)

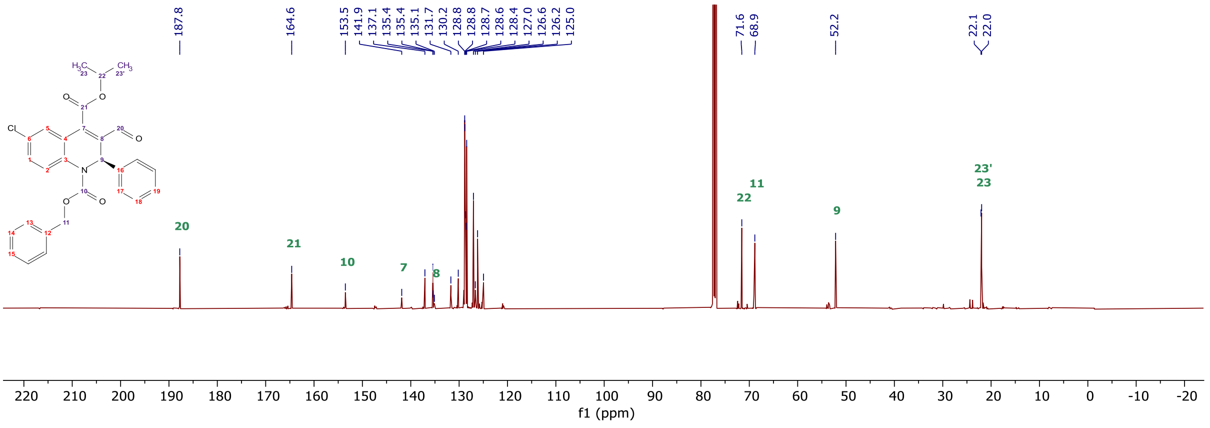

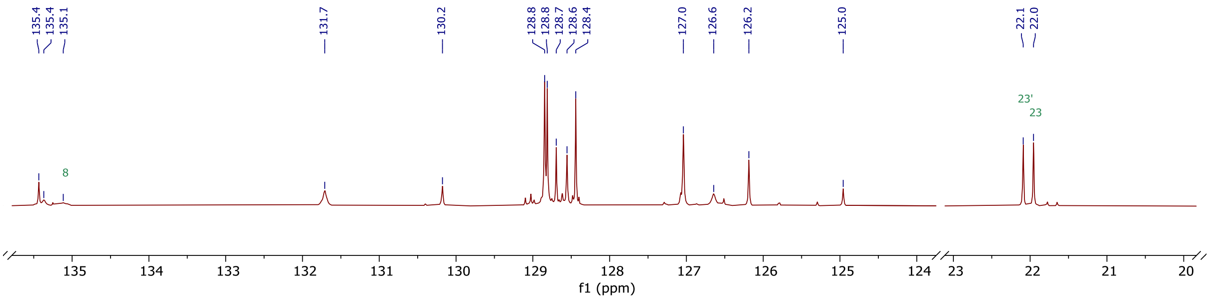

$^{1}H/^{1}H$ COSY

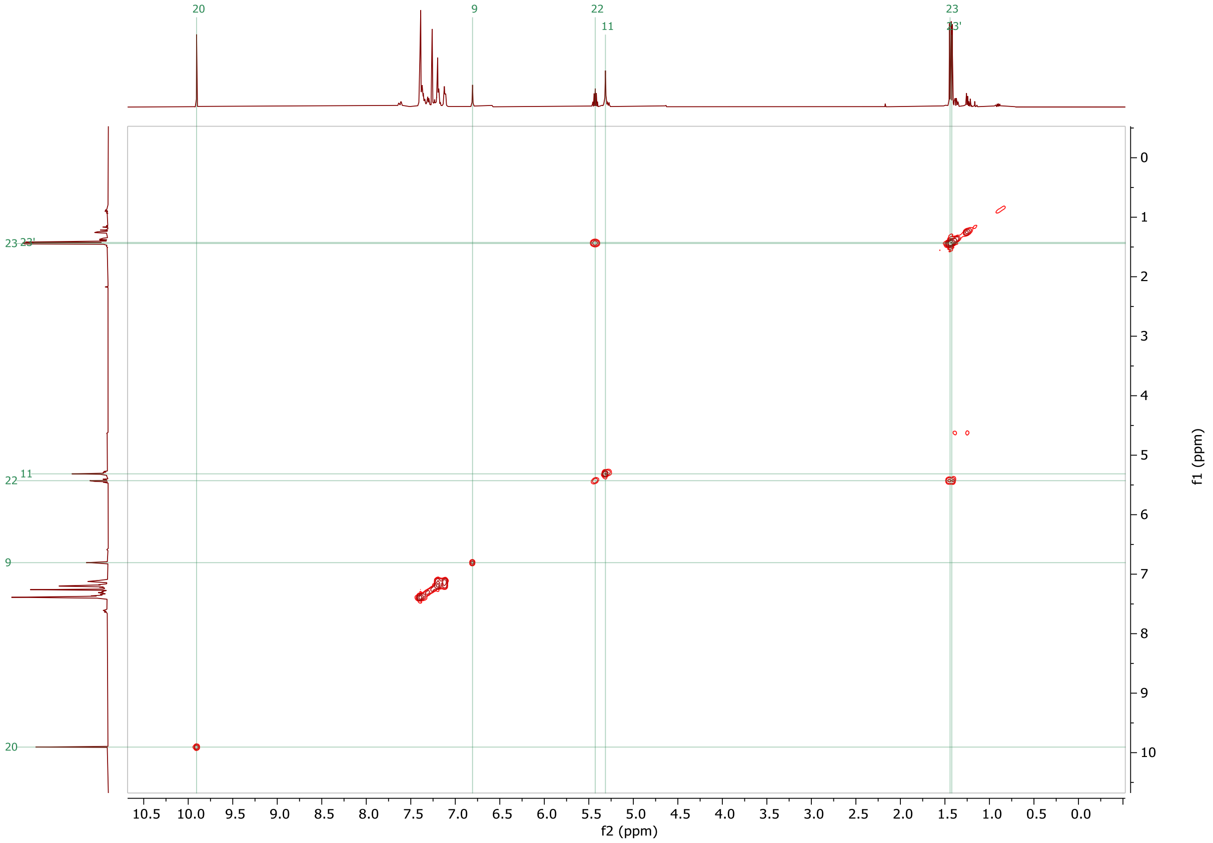

$^{1}H/^{13}C$ HSQC

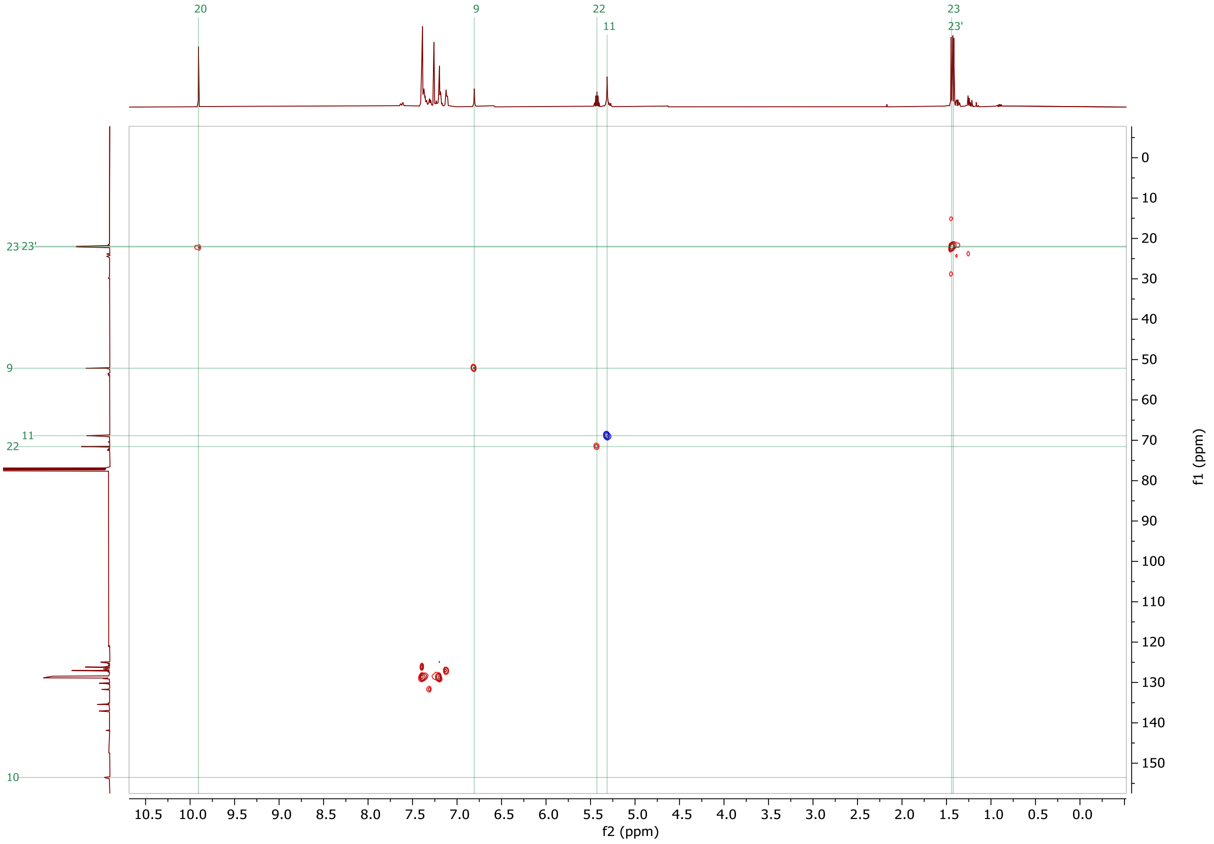

$^{1}H/^{13}C$ HMBC

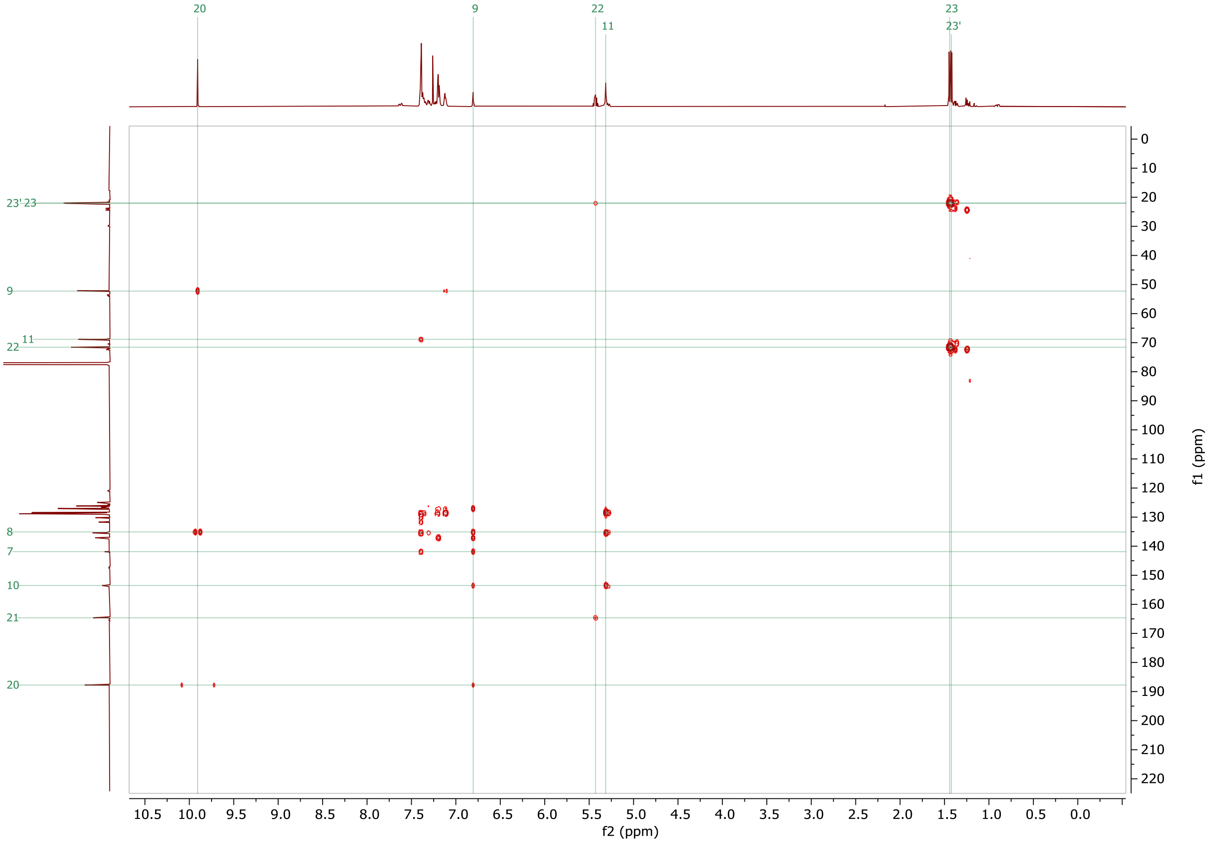

1-Benzyl 4-isopropyl (*R*)-3-formyl-6-methyl-2-phenylquinoline-1,4(2H)-dicarboxylate (–)-18f

^{1}H NMR (500 MHz, $CDCl_3$)

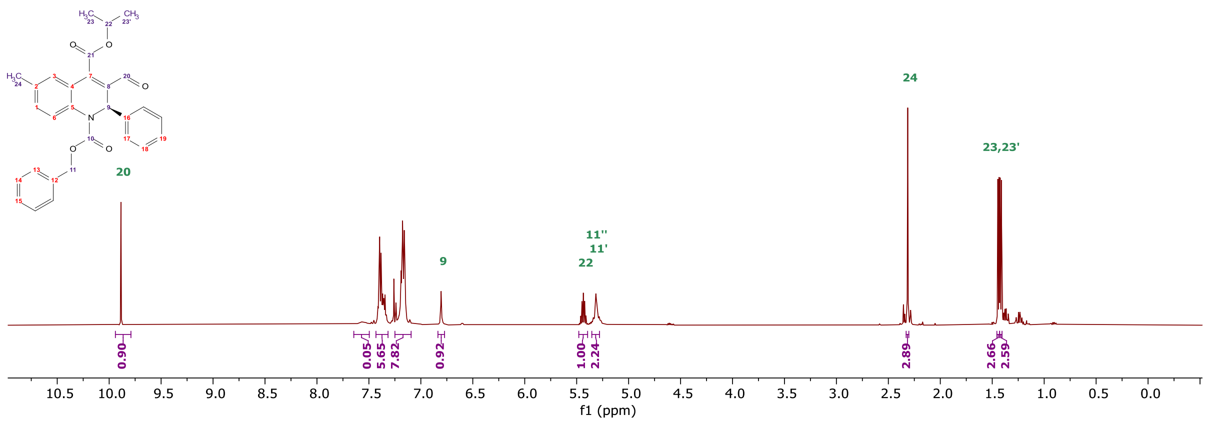

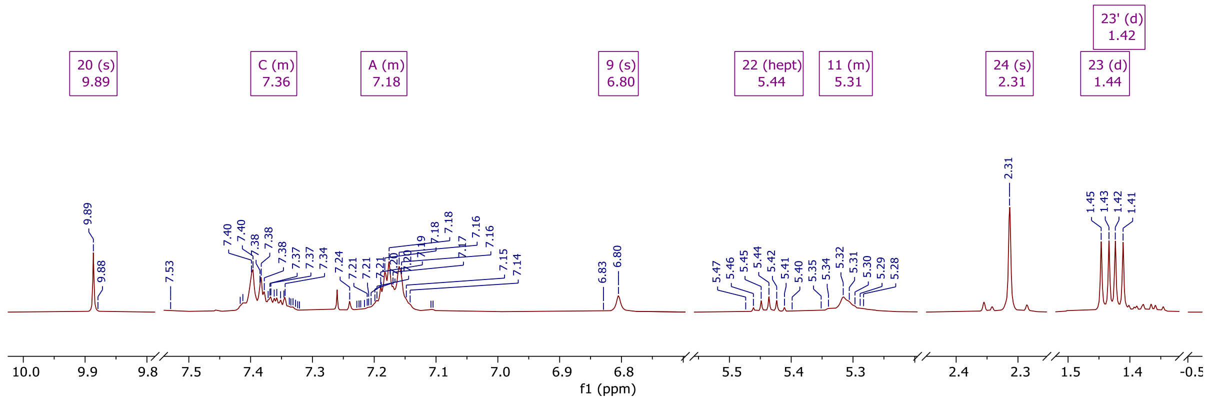

^{13}C NMR (126 MHz, $CDCl_3$)

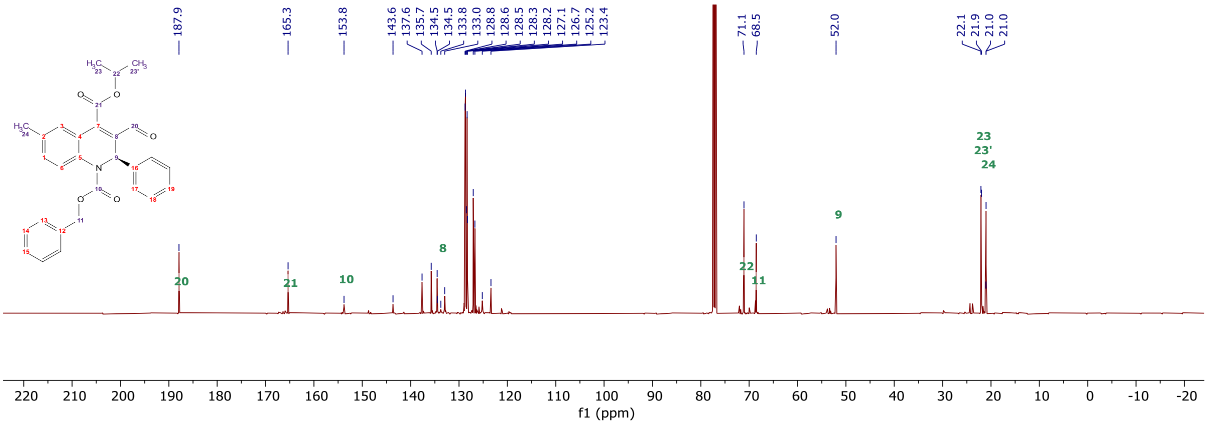

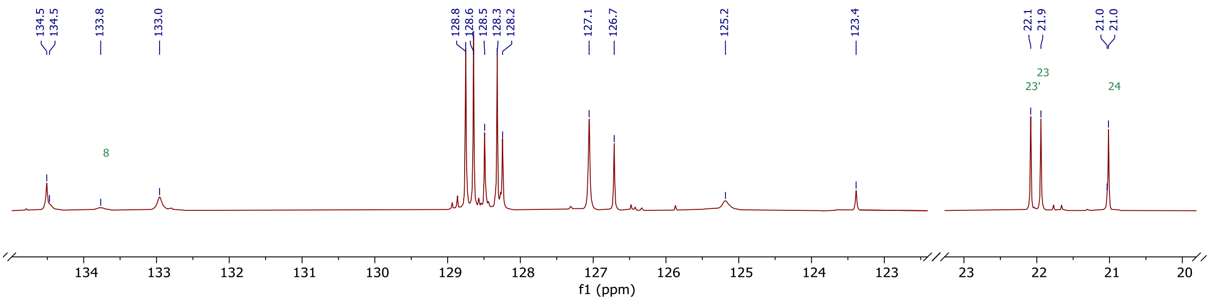

$^1H/^1H$ COSY

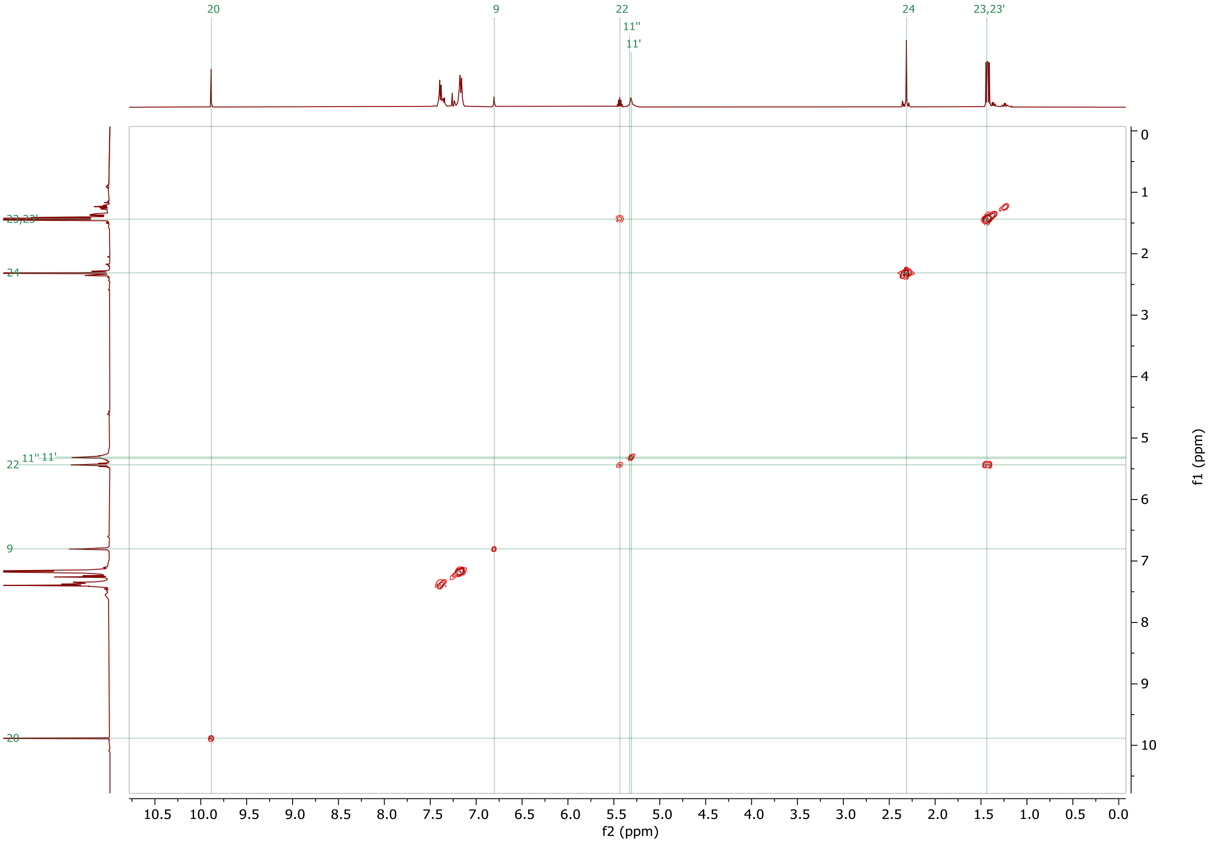

$^1H/^{13}C$ HSQC

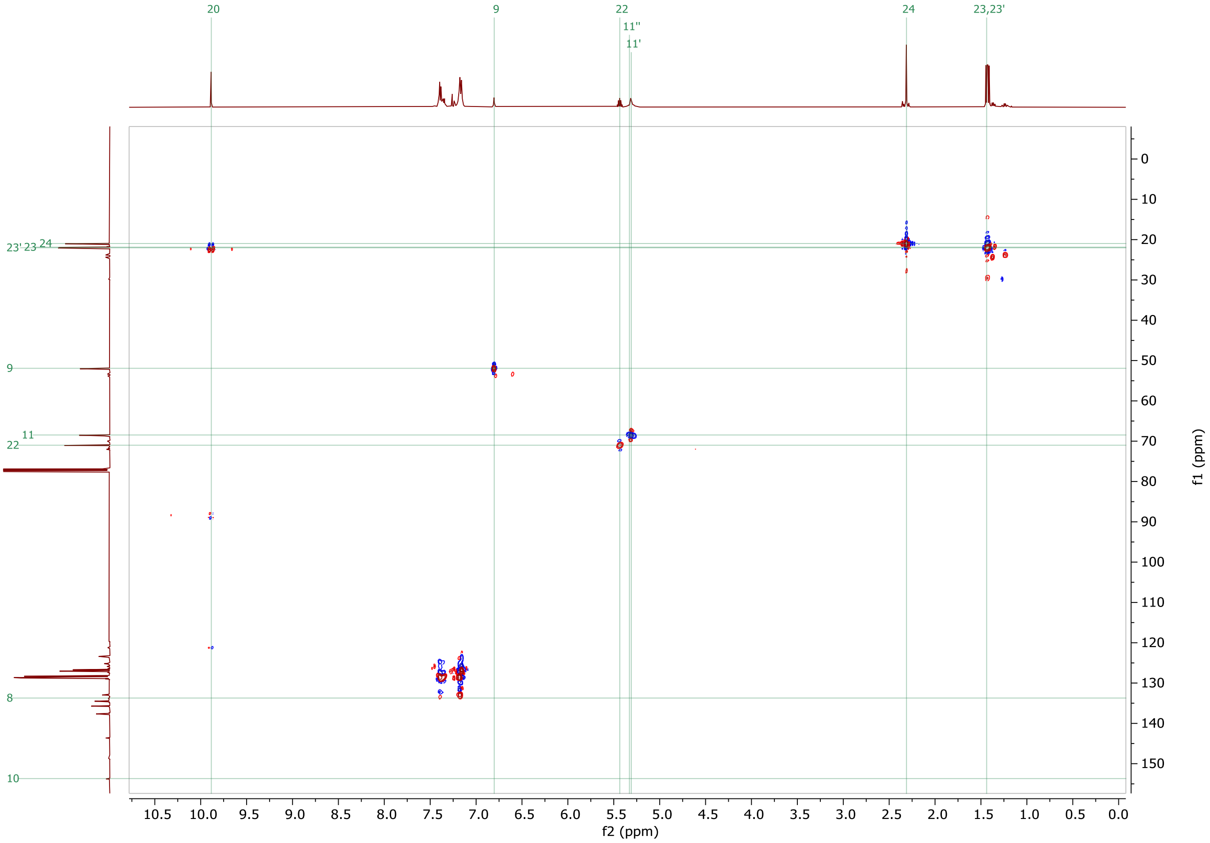

$^{1}H/^{13}C$ HMBC

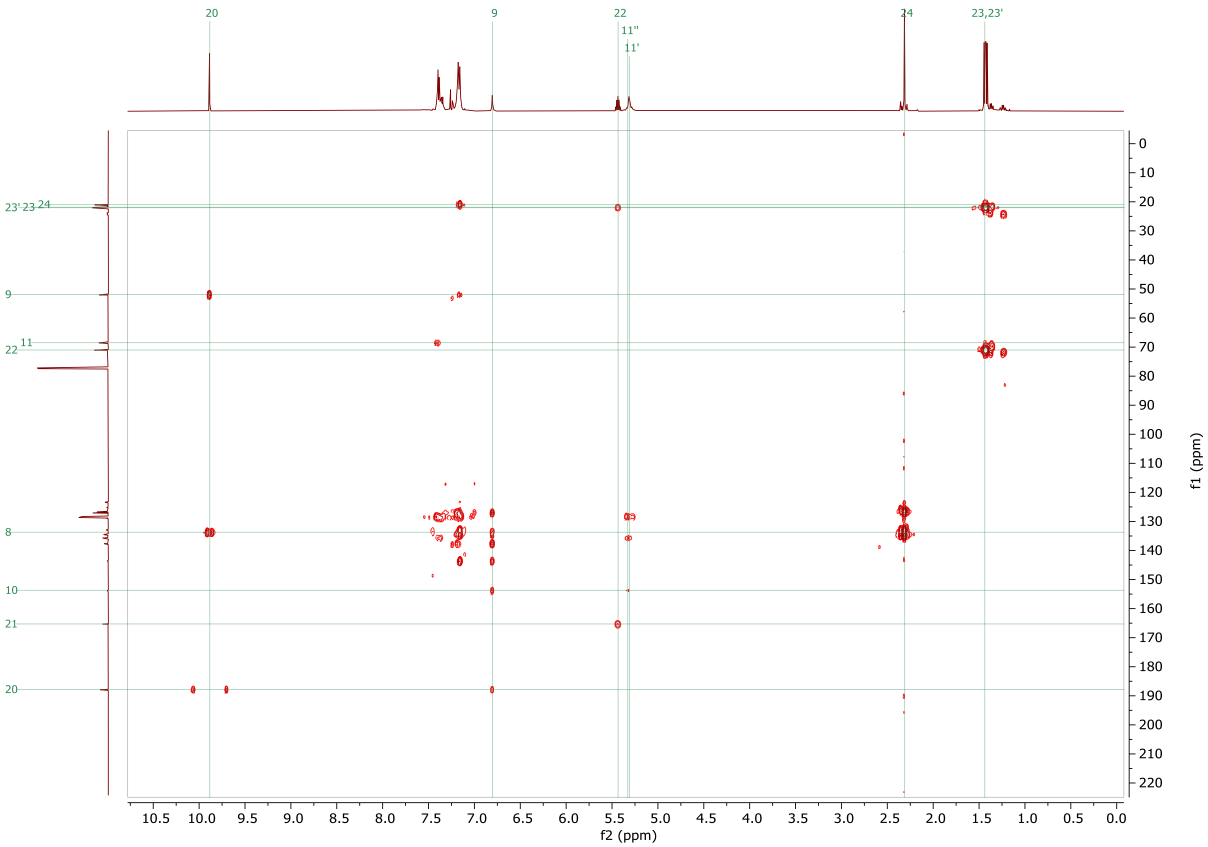

1-Benzyl 4-isopropyl (*R*)-8-fluoro-3-formyl-2-phenylquinoline-1,4(2H)-dicarboxylate (–)-18i

^{1}H NMR (500 MHz, $CDCl_3$)

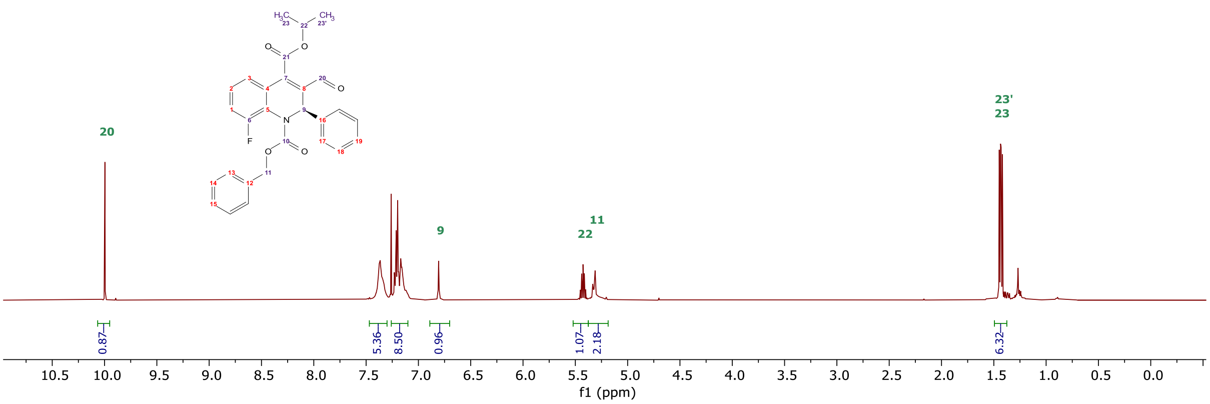

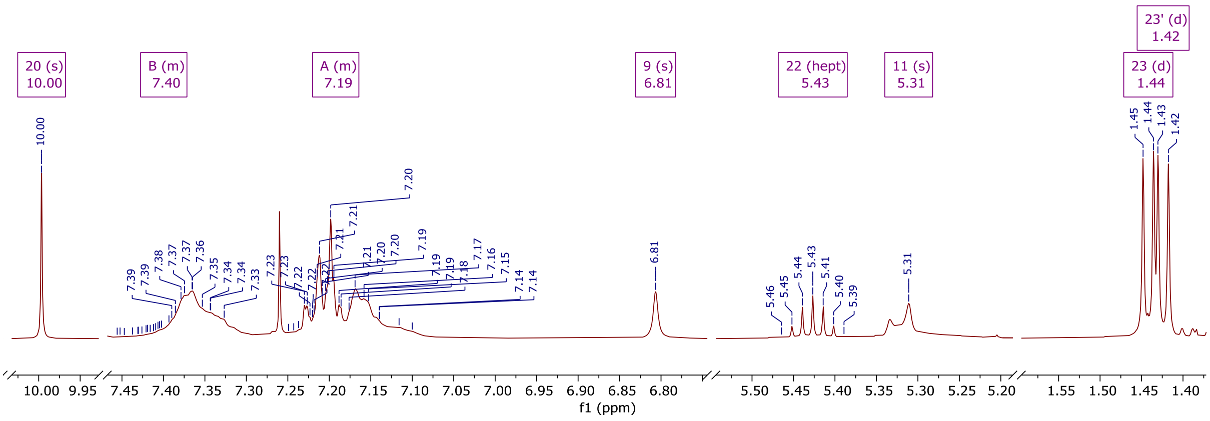

^{13}C NMR (126 MHz, $CDCl_3$)

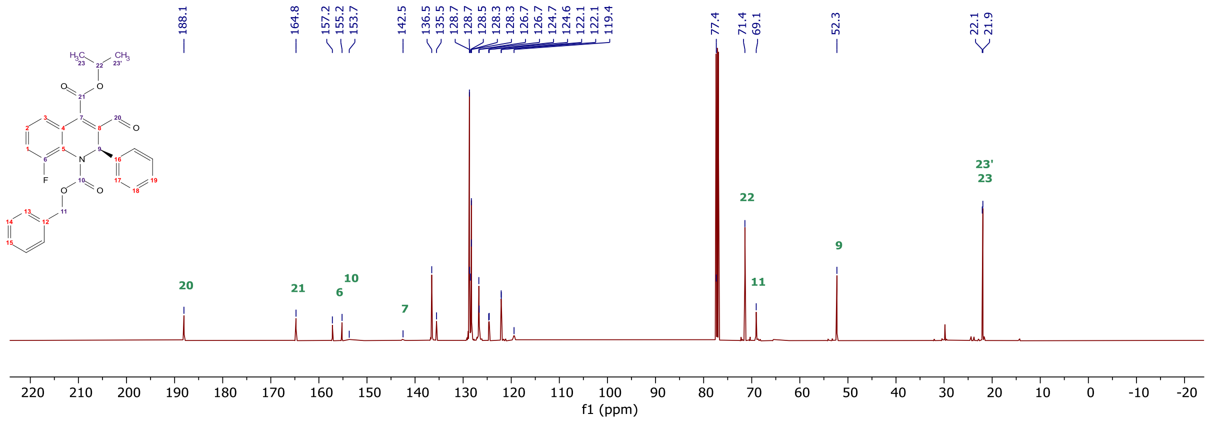

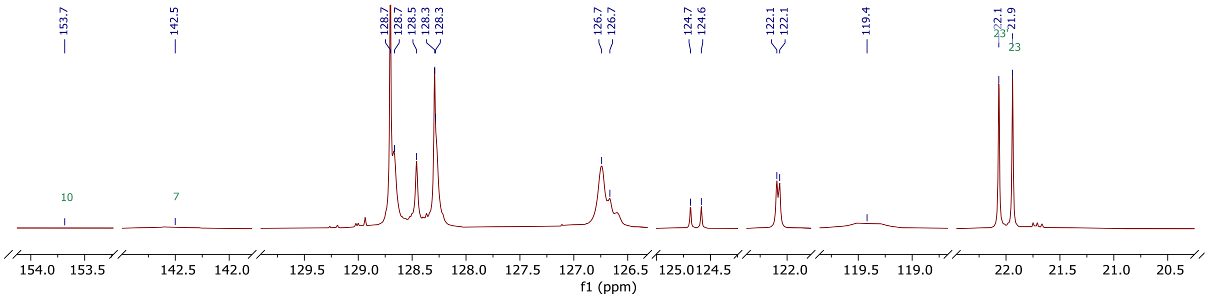

^{19}F NMR (471 MHz, $CDCl_3$)

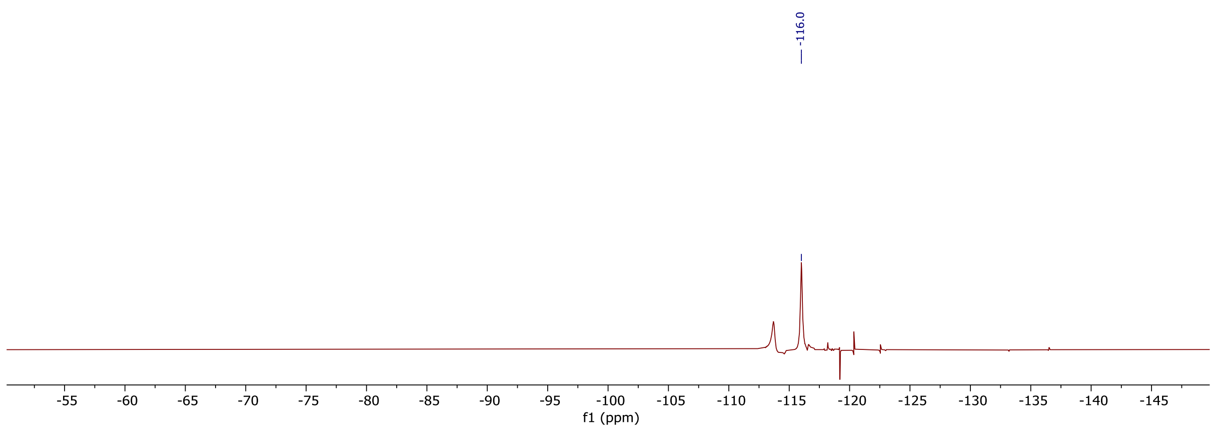

$^{1}H/^{1}H$ COSY

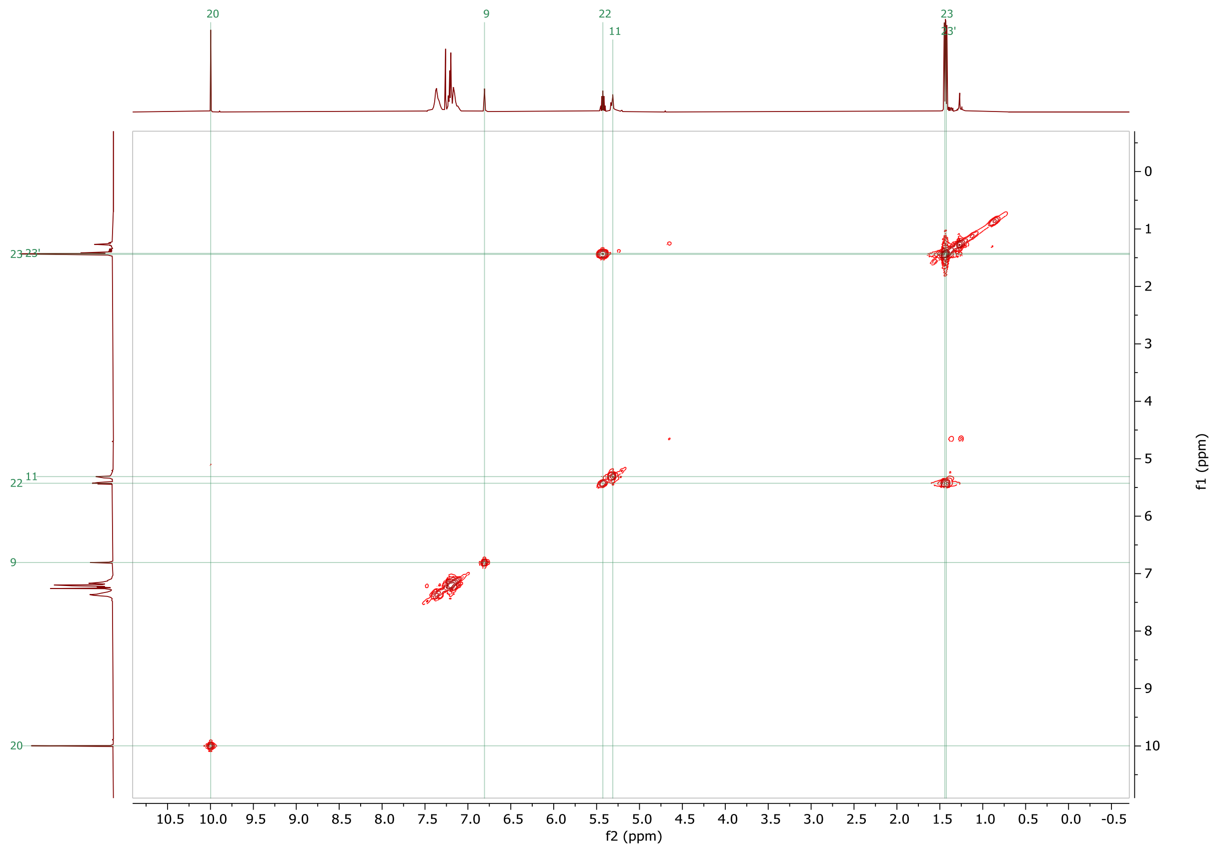

$^{1}H/^{13}C$ HSQC

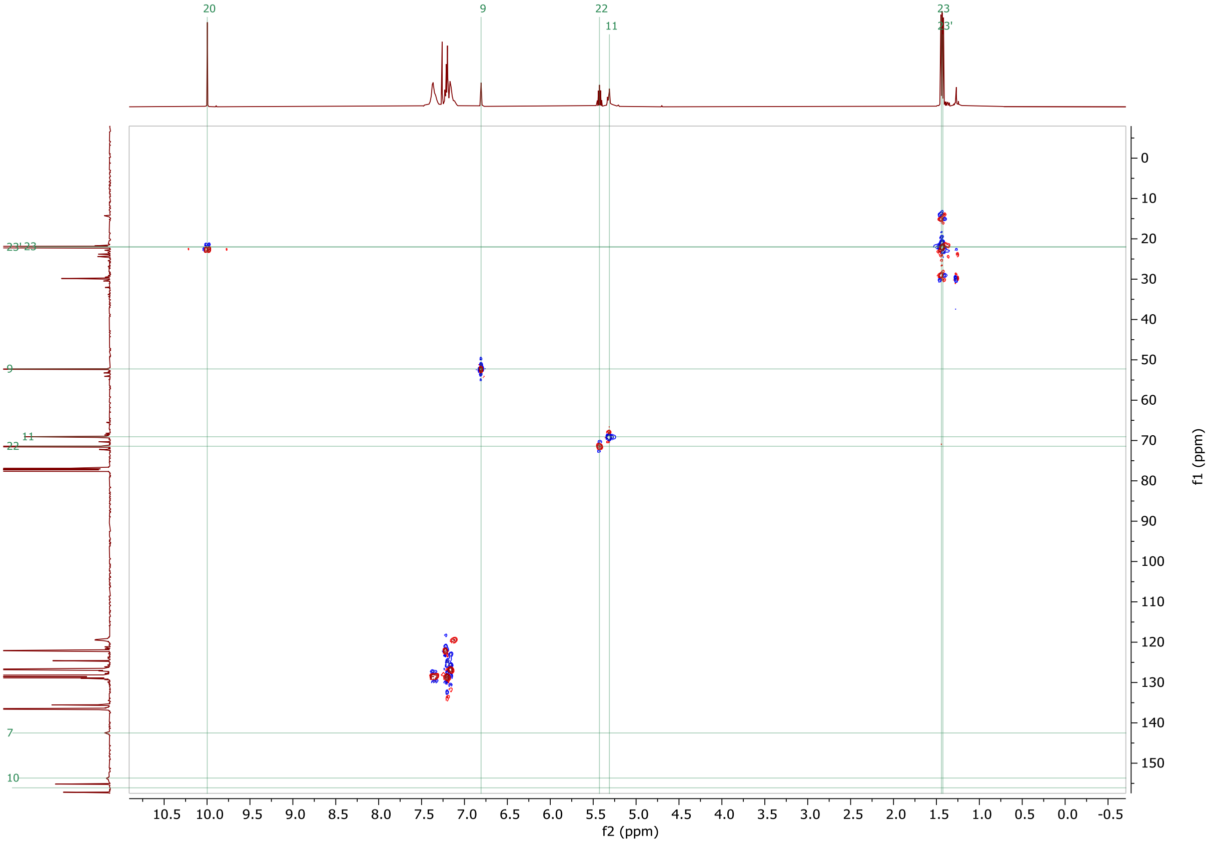

$^{1}H/^{13}C$ HMBC

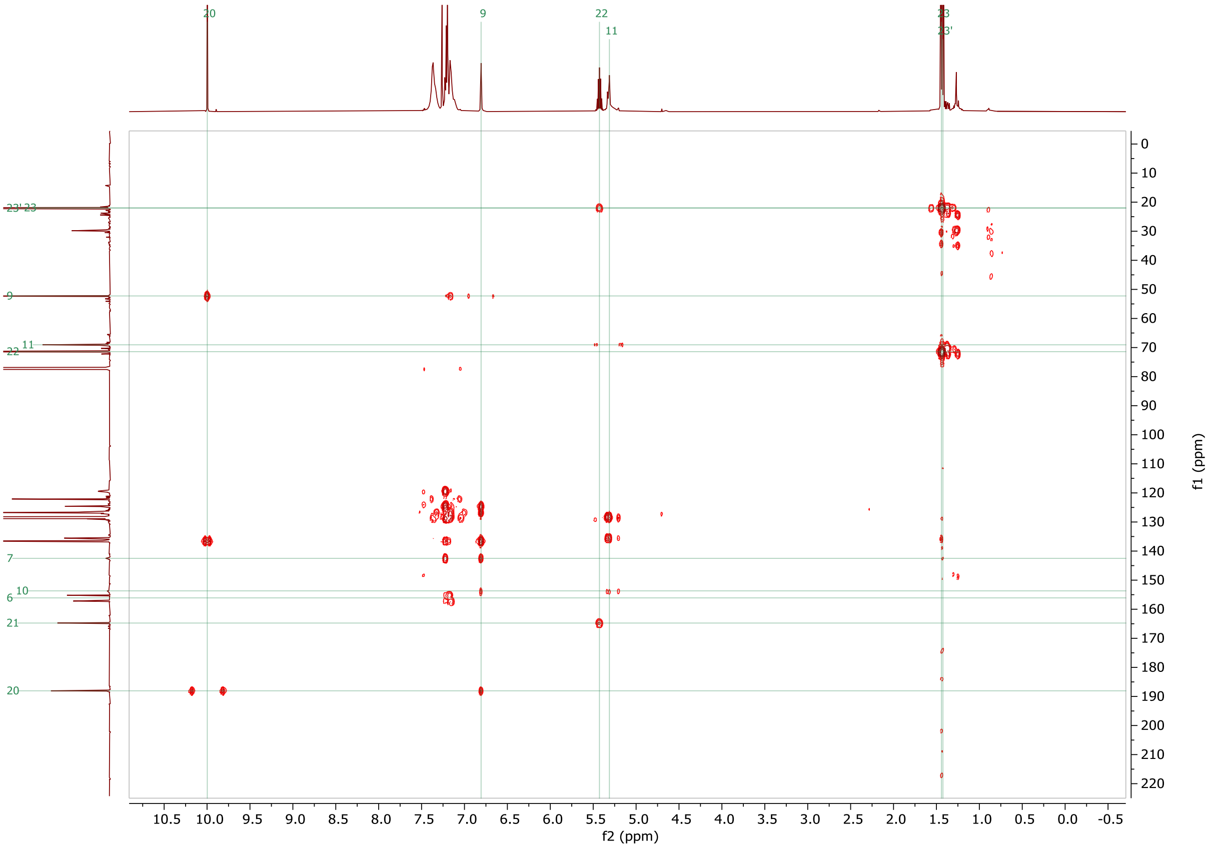

1-Benzyl 4-isopropyl (*R*)-3-formyl-6-nitro-2-phenylquinoline-1,4(2H)-dicarboxylate (–)-18g

^{1}H NMR (500 MHz, $CDCl_3$)

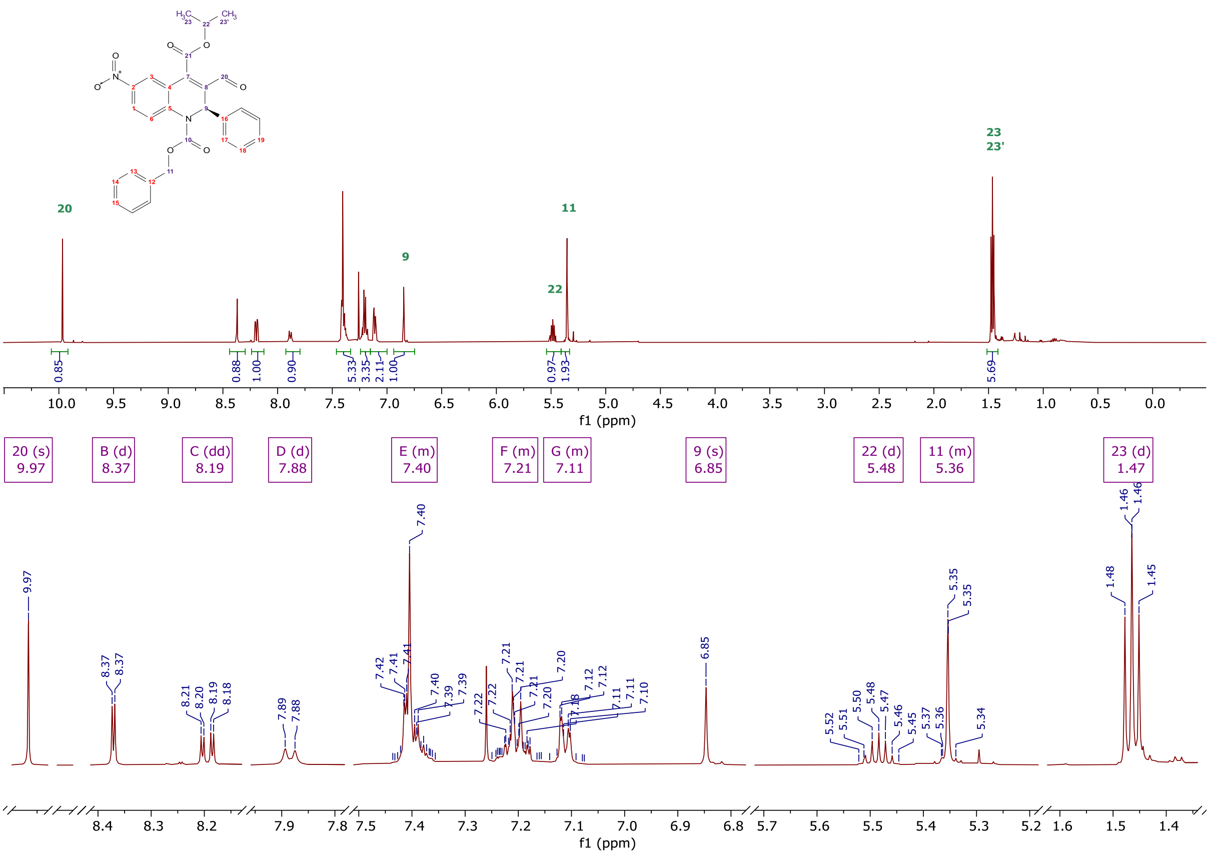

^{13}C NMR (126 MHz, $CDCl_3$)

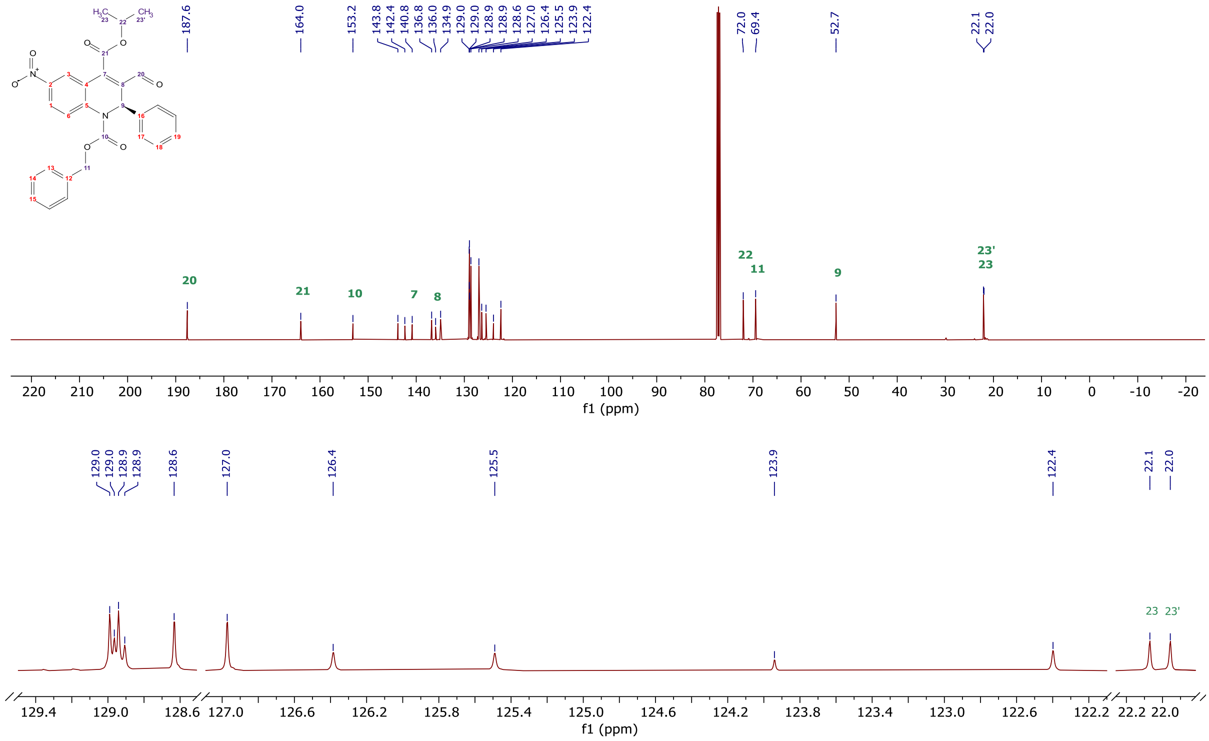

$^1H/^1H$ COSY

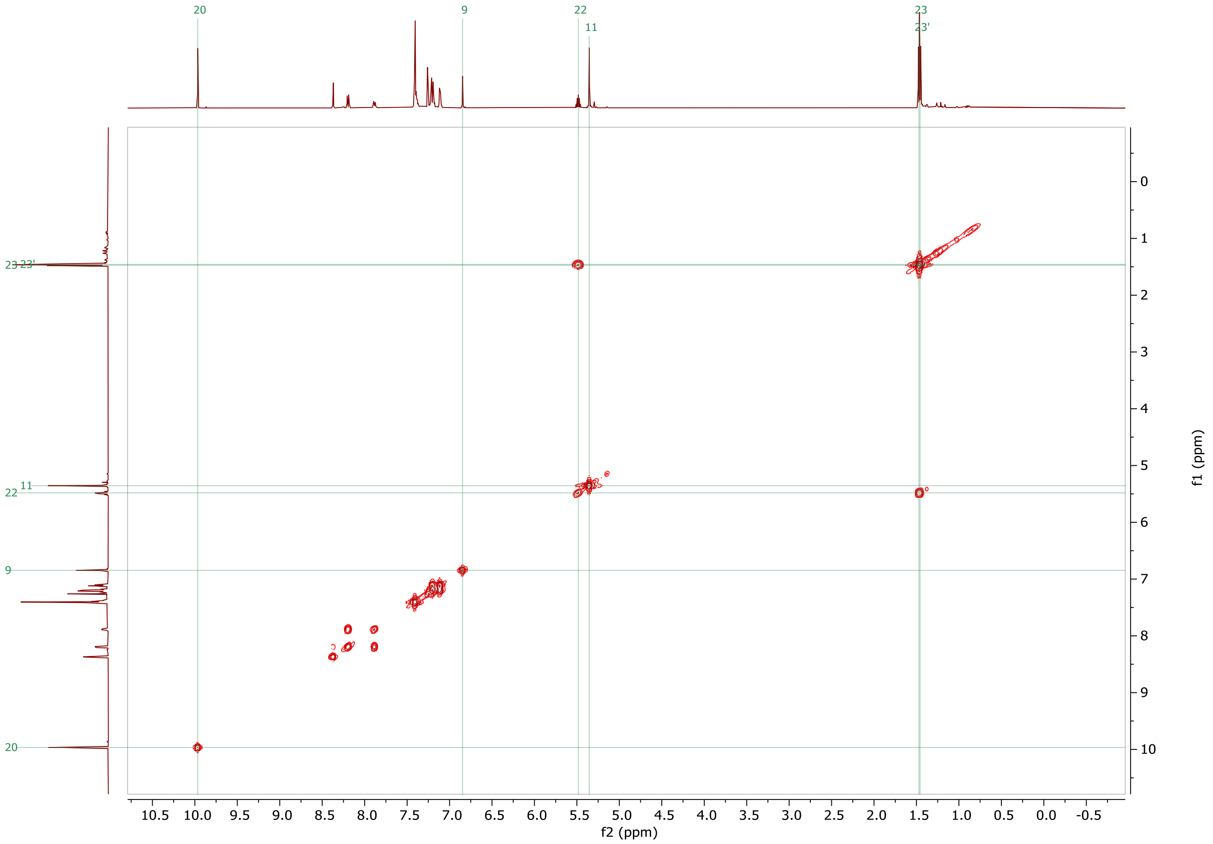

$^1H/^{13}C$ HSQC

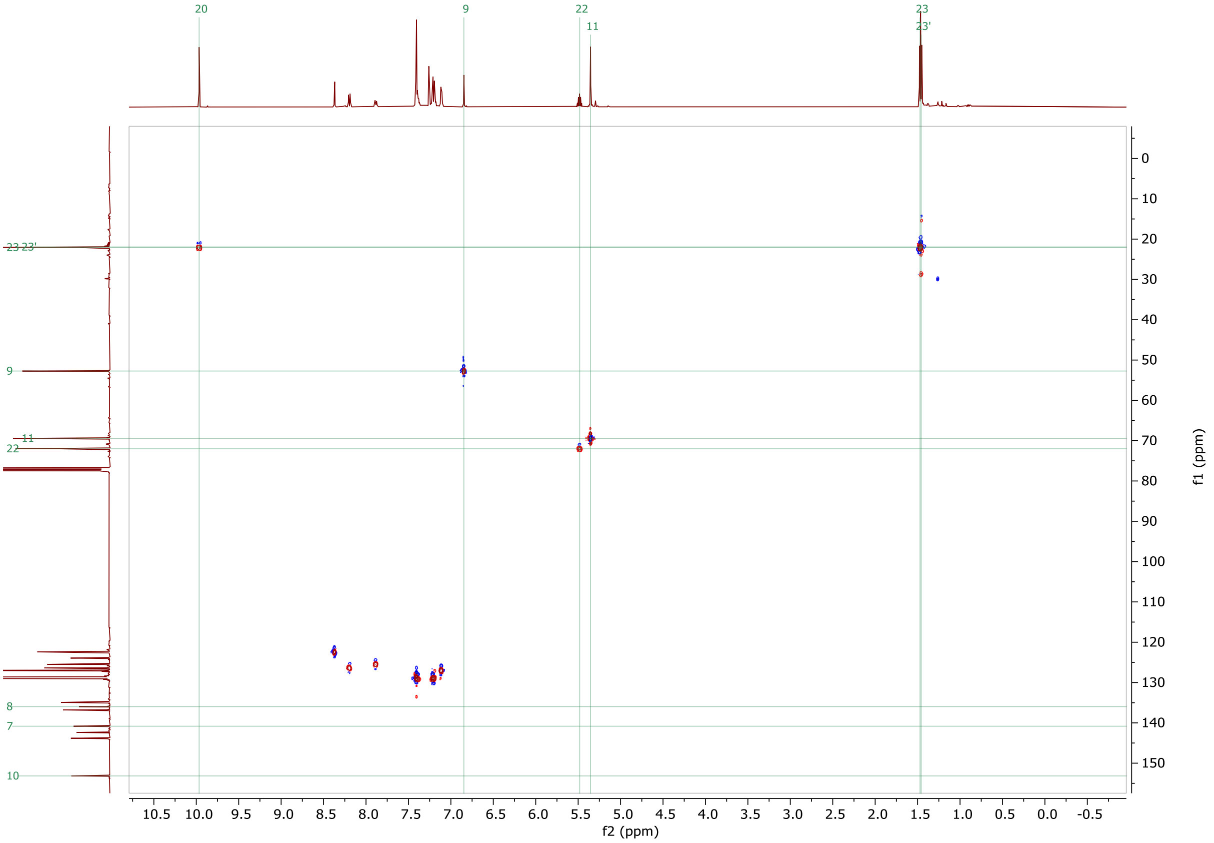

$^{1}H/^{13}C$ HMBC

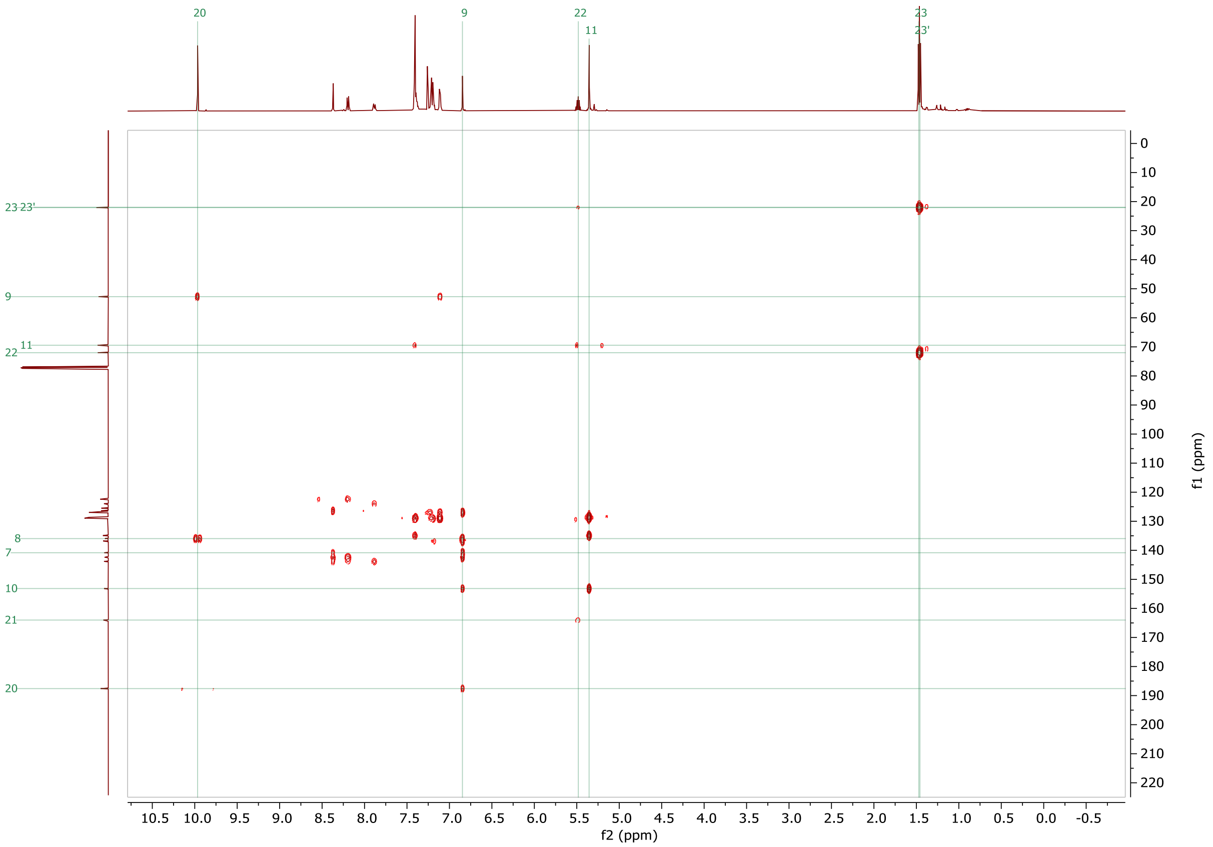

1-Benzyl 4-isopropyl (*R*)-3-formyl-6-methoxy-2-phenylquinoline-1,4(2H)-dicarboxylate (–)-18h

^{1}H NMR (500 MHz, $CDCl_3$)

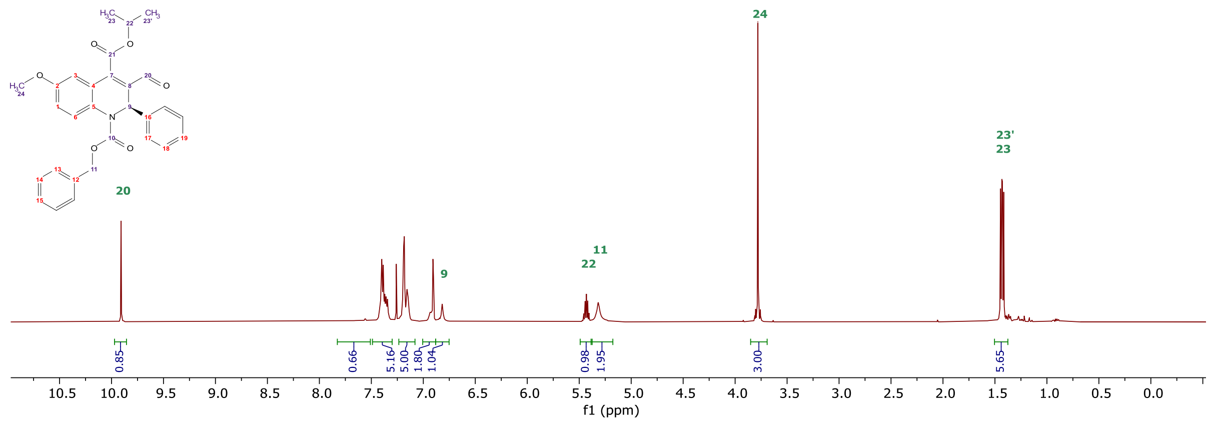

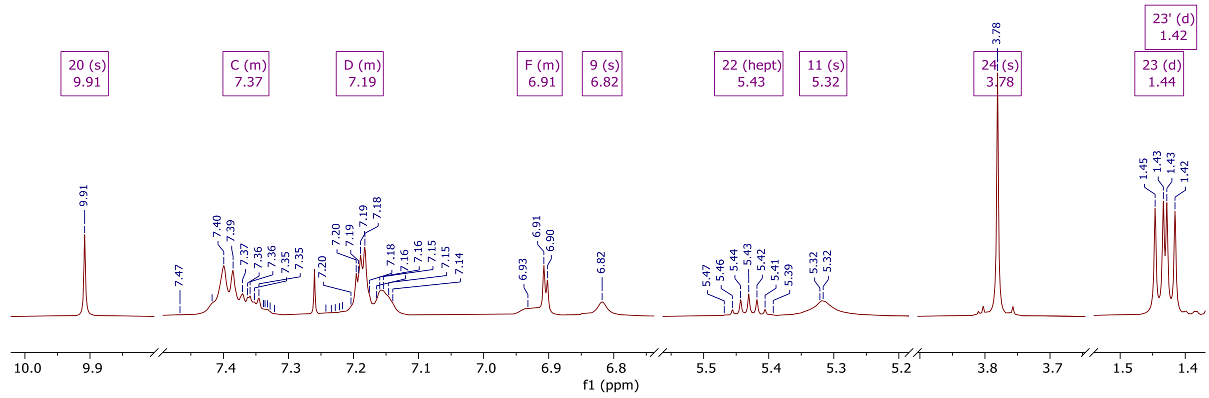

^{13}C NMR (126 MHz, $CDCl_3$)

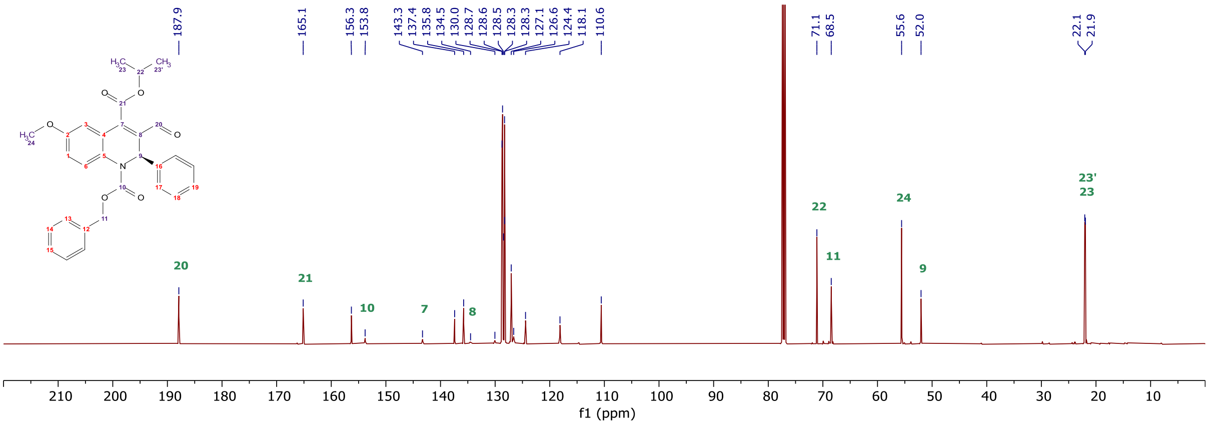

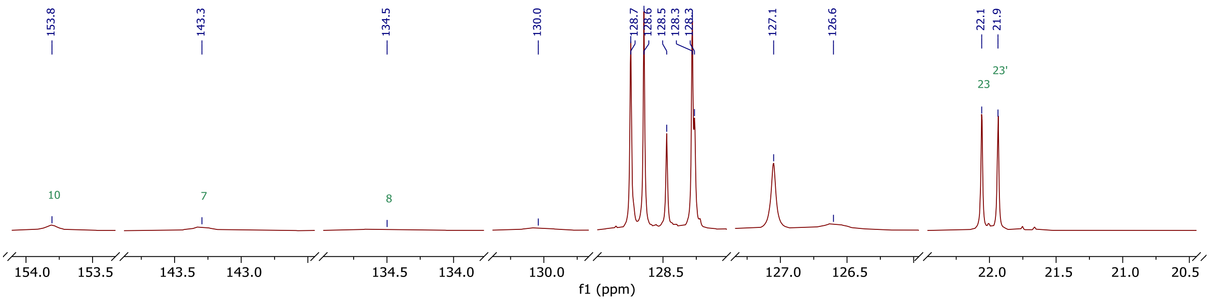

$^{1}H/^{1}H$ COSY

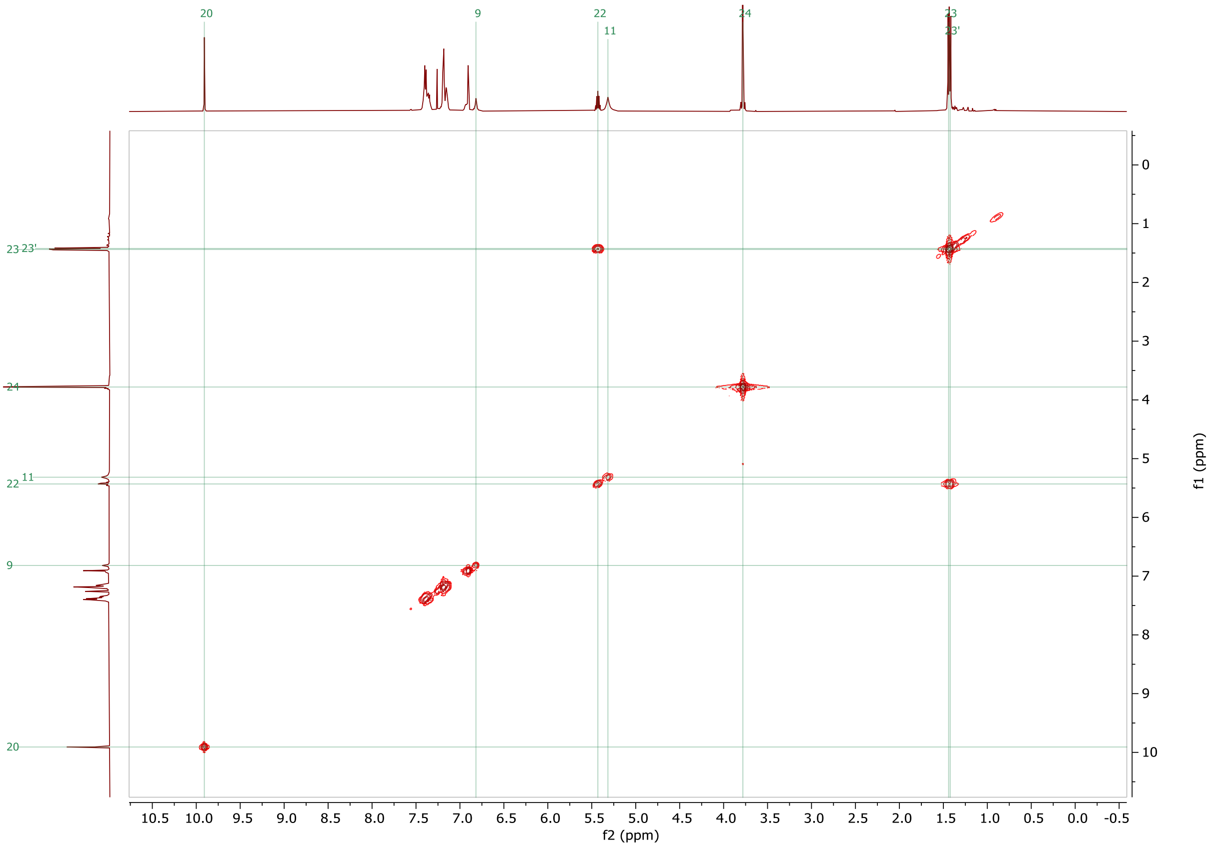

$^{1}H/^{13}C$ HSQC

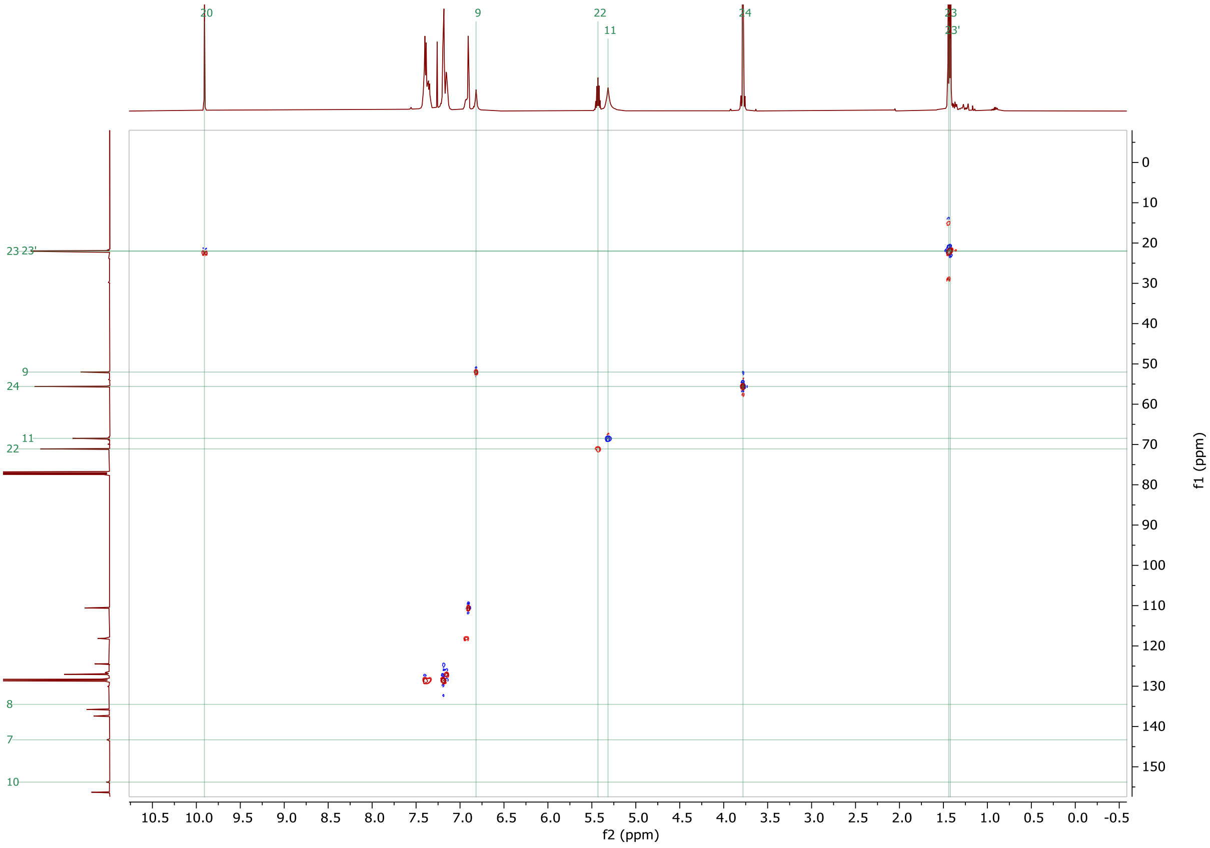

$^{1}H/^{13}C$ HMBC

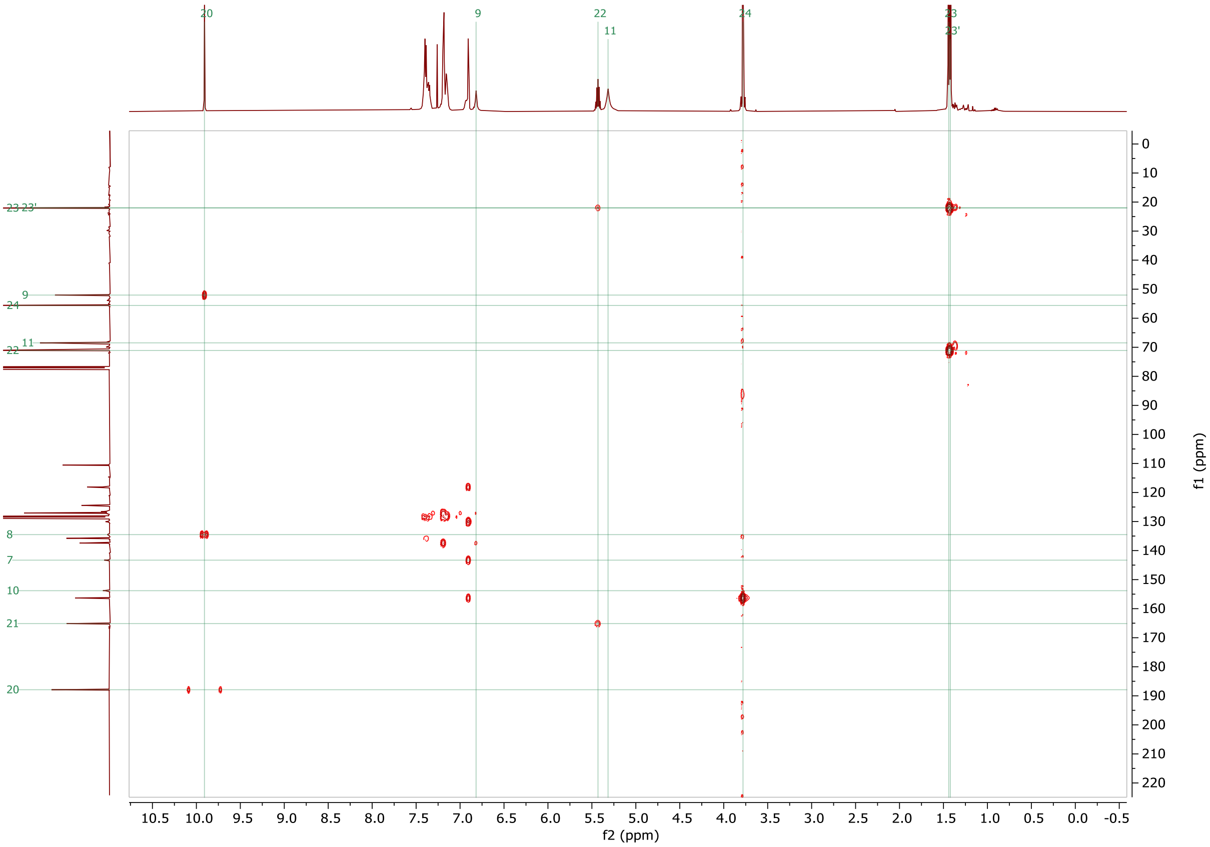

1-Benzyl 4-isopropyl (*R*)-5,7-dichloro-3-formyl-2-phenylquinoline-1,4(2H)-dicarboxylate (–)-18j

^{1}H NMR (500 MHz, $CDCl_3$)

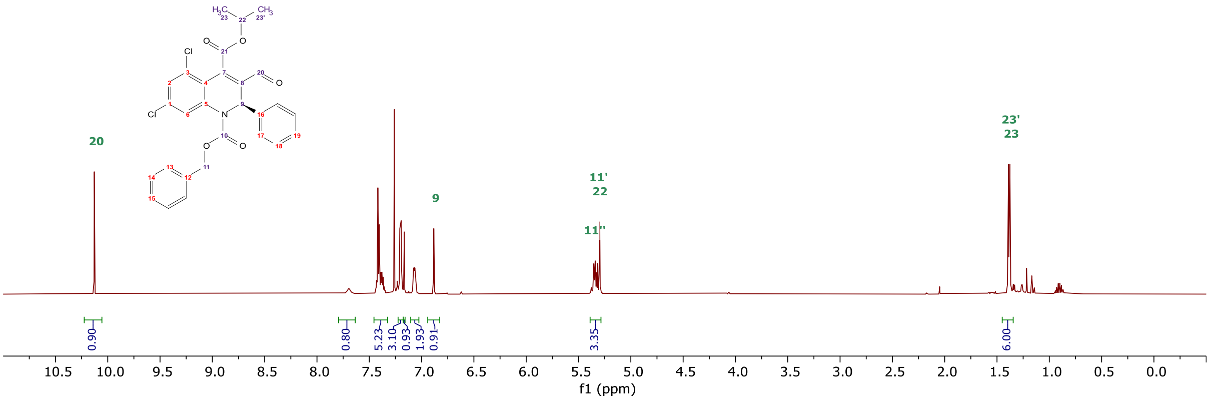

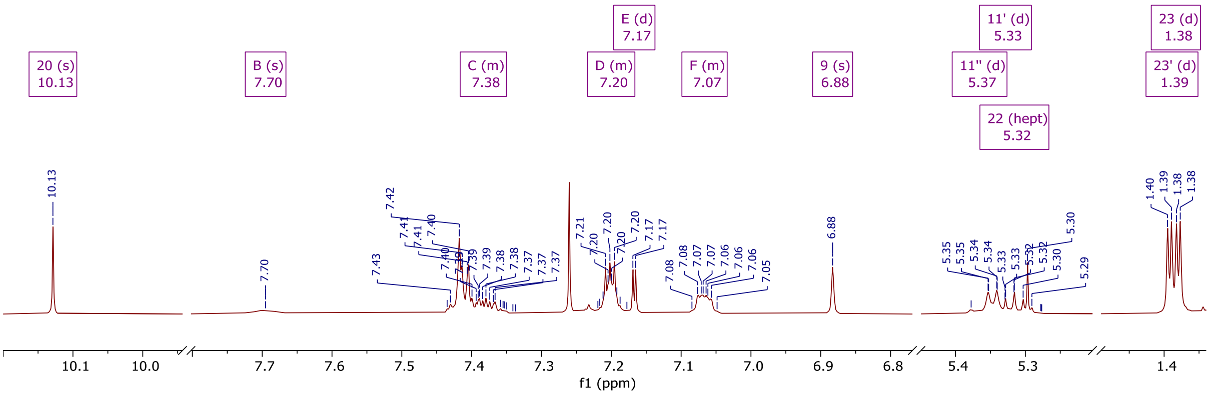

^{13}C NMR (126 MHz, $CDCl_3$)

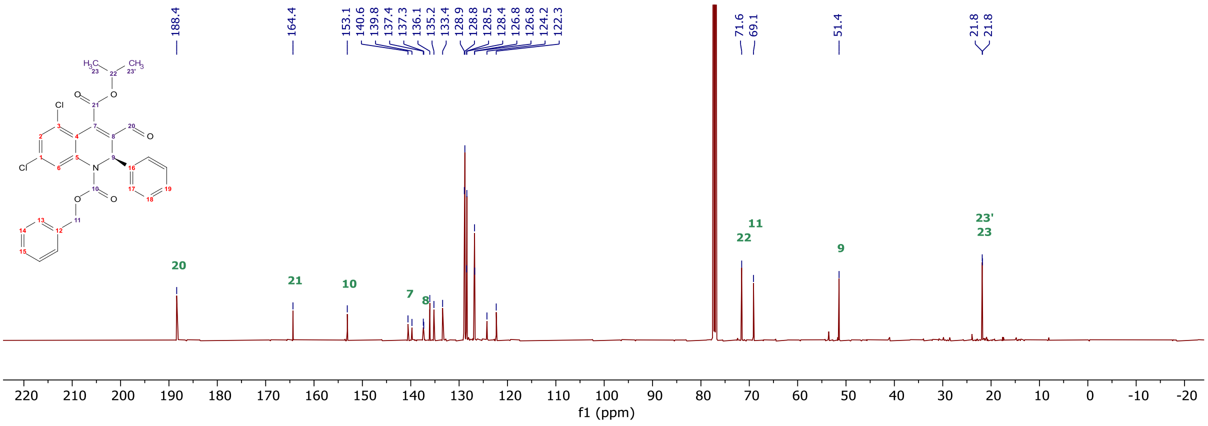

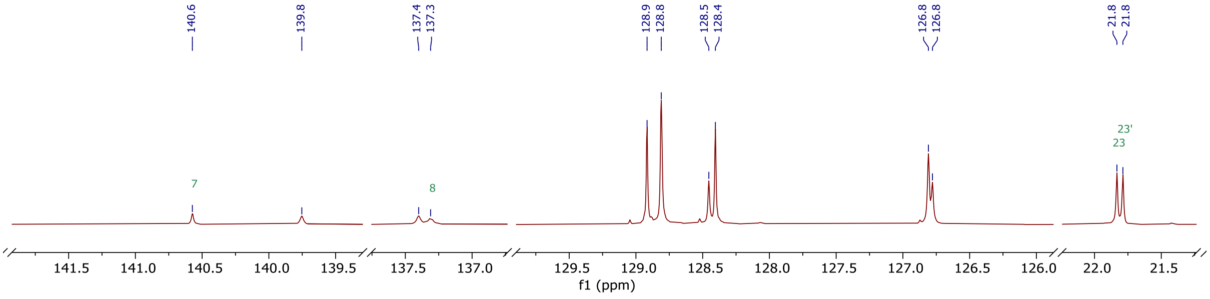

$^{1}H/^{1}H$ COSY

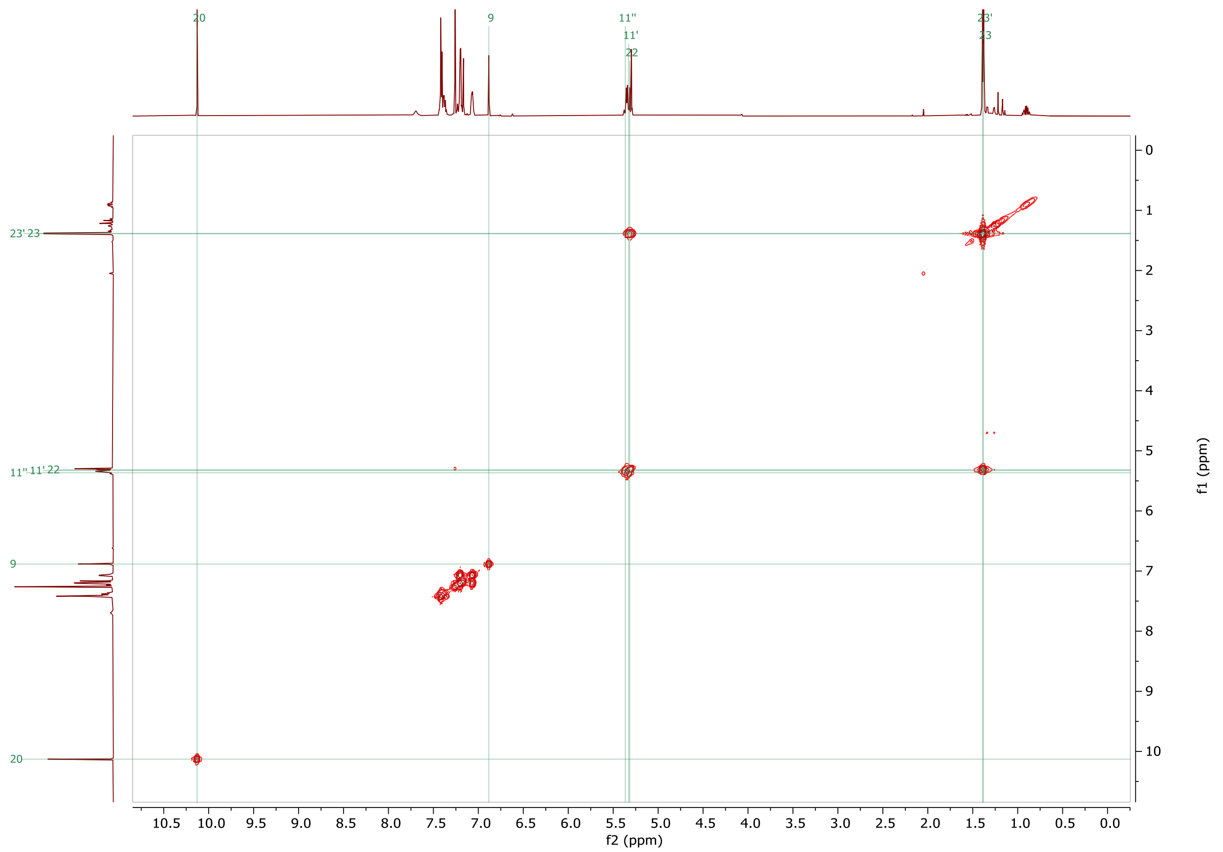

$^{1}H/^{13}C$ HSQC

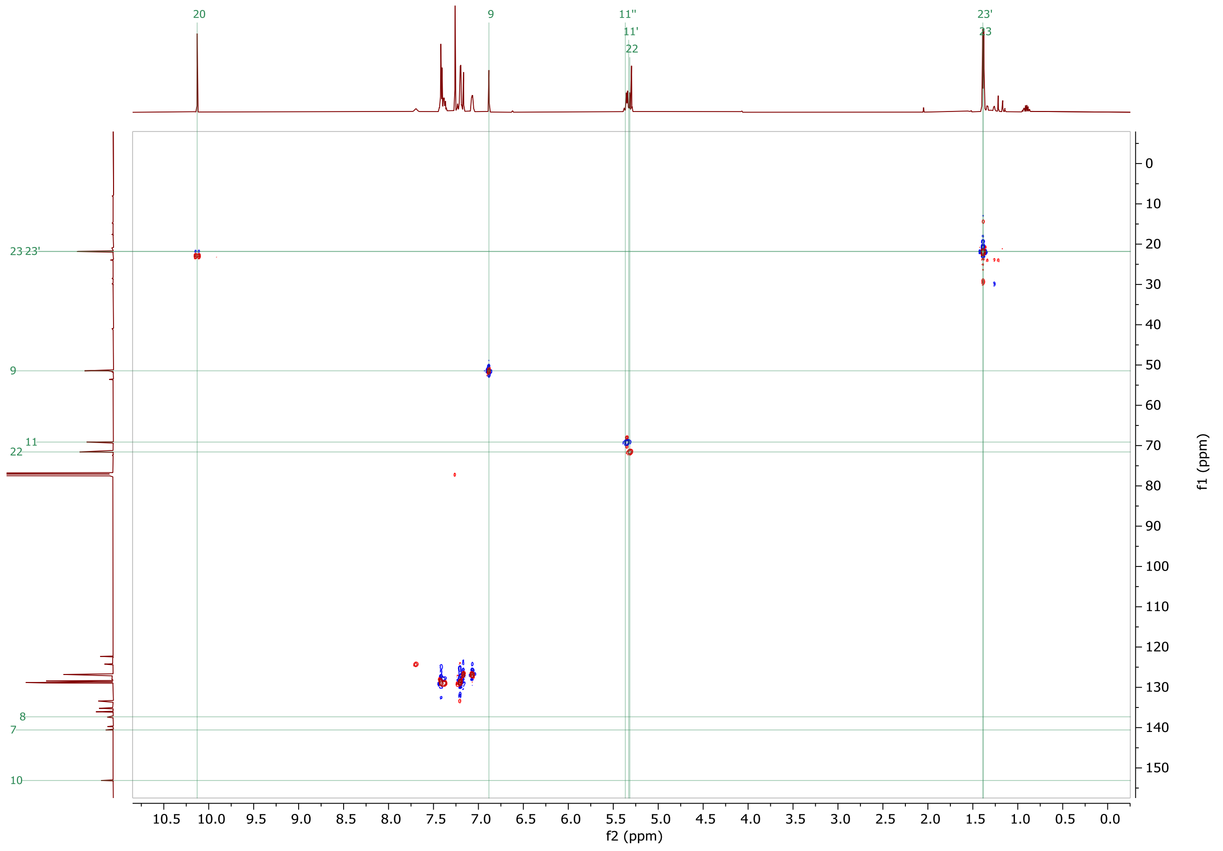

$^{1}H/^{13}C$ HMBC

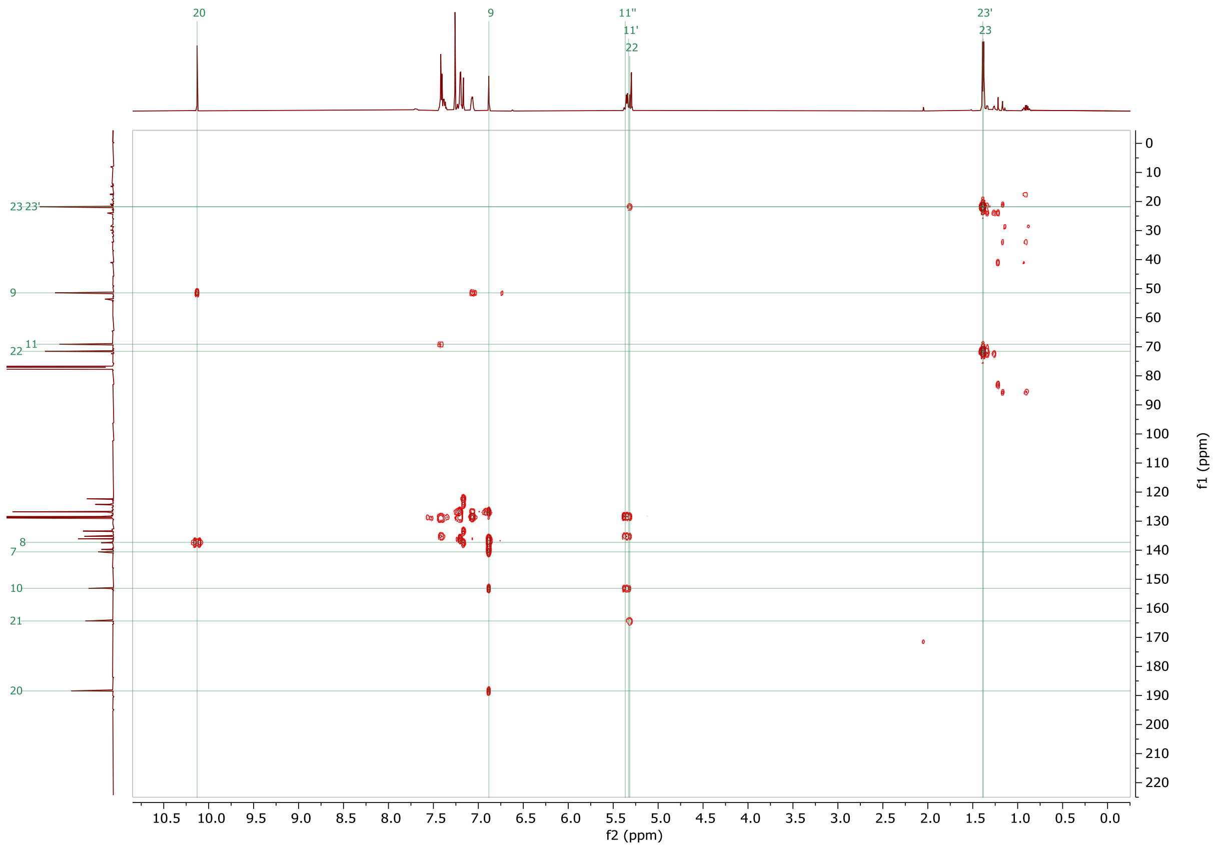

1-Benzyl 4-isopropyl (*R*)-2-(4-chlorophenyl)-3-formylquinoline-1,4(2H)-dicarboxylate (–)-18k

^{1}H NMR (500 MHz, $CDCl_3$)

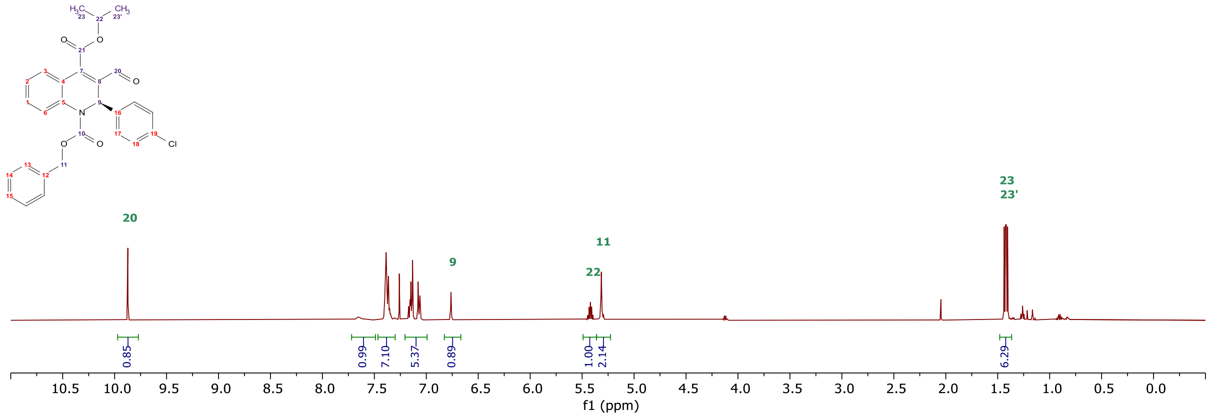

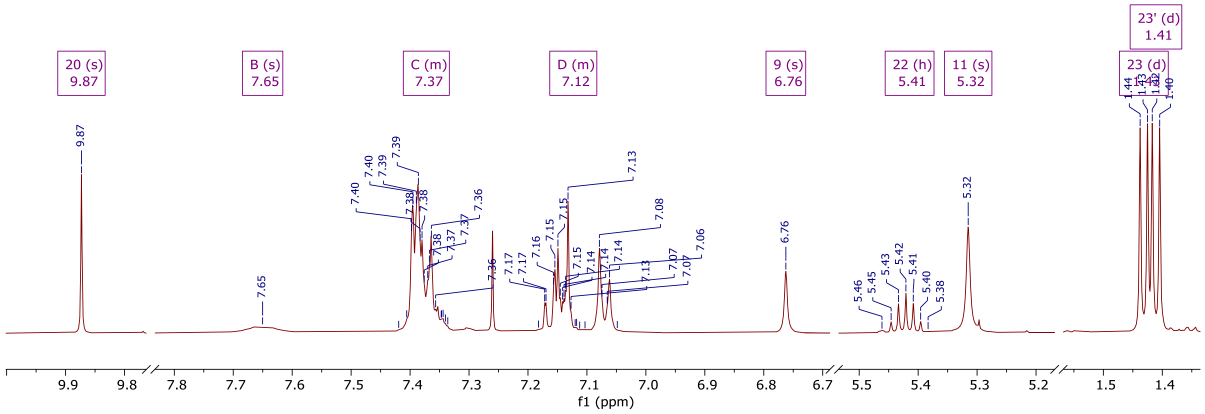

^{13}C NMR (126 MHz, $CDCl_3$)

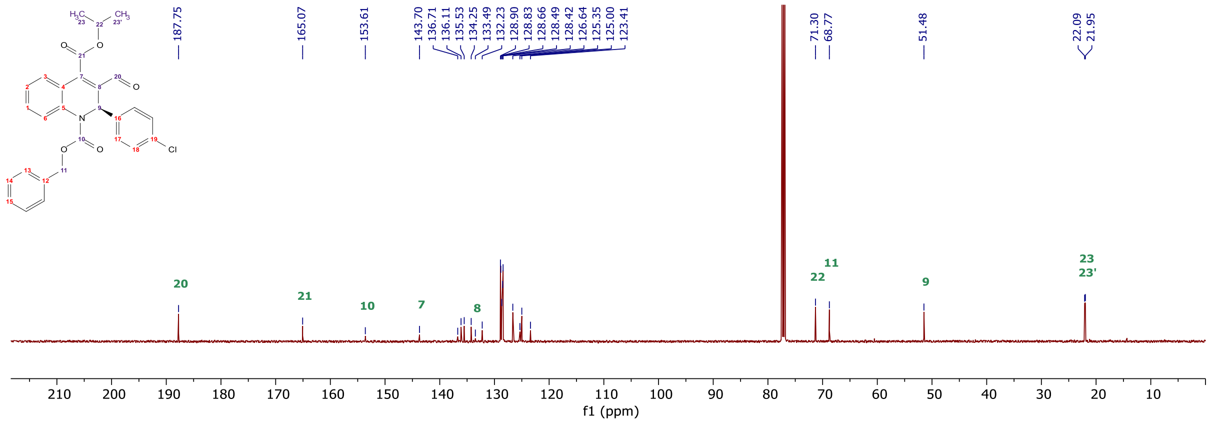

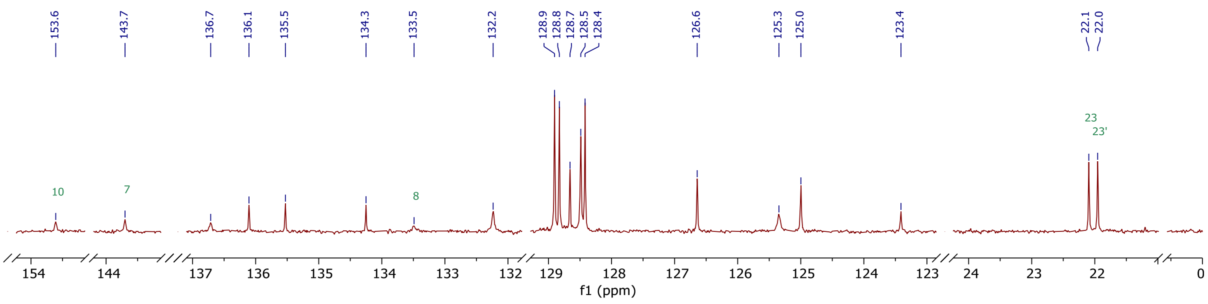

$^1H/^1H$ COSY

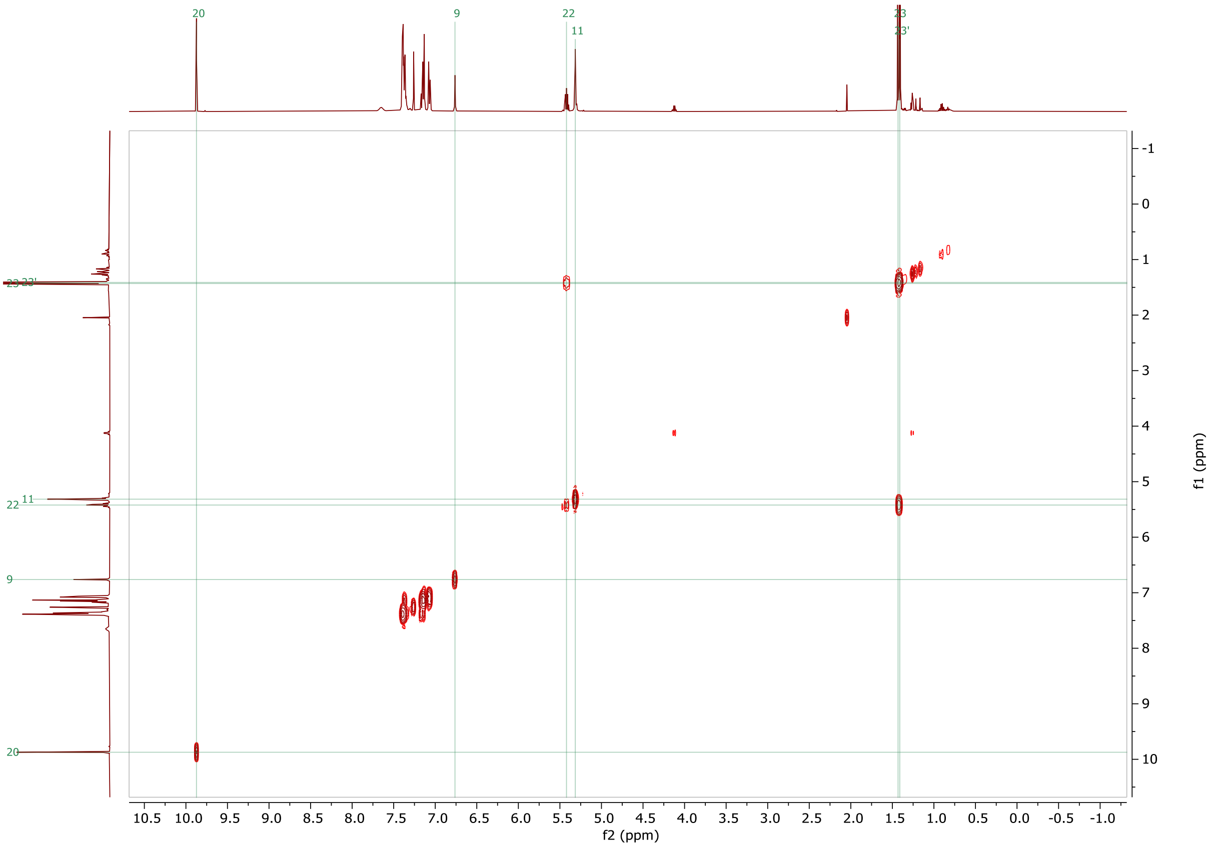

$^1H/^{13}C$ HSQC

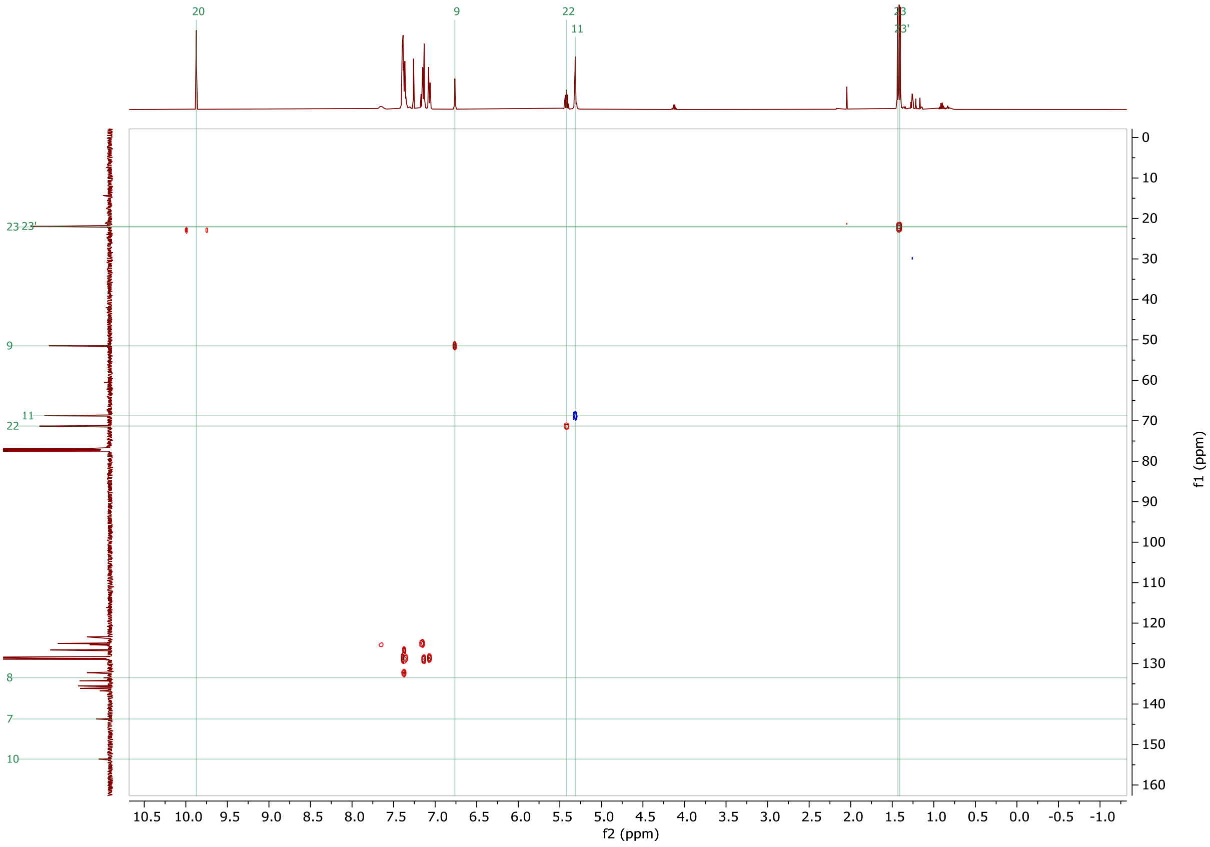

$^1H/^{13}C$ HMBC

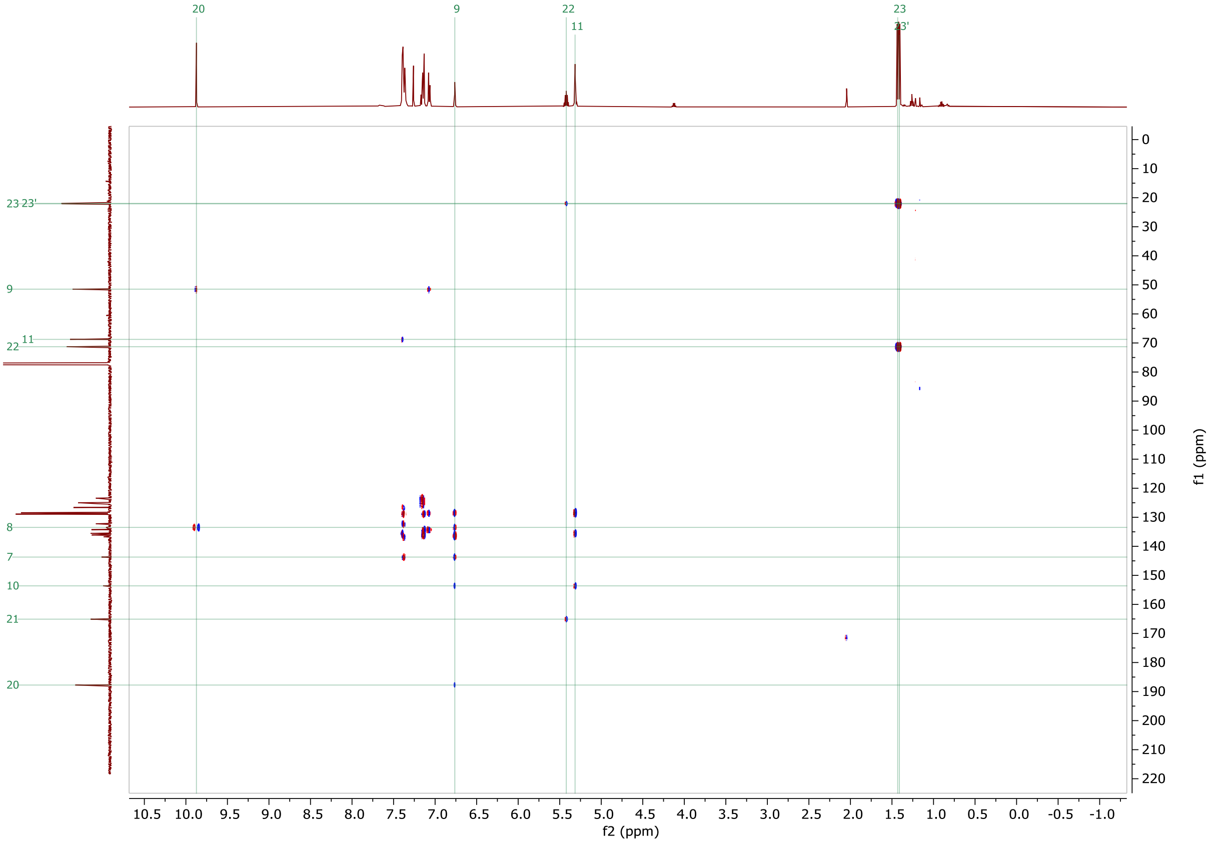
20
9
22
11
23
23'
23 23'
9
22
11
8
7
10
21
20
0
10
20
30
40
50
60
70
80
90
100
110
120
130
140
150
160
170
180
190
200
210
220
f1 (ppm)
10.5
10.0
9.5
9.0
8.5
8.0
7.5
7.0
6.5
6.0
5.5
5.0
4.5
4.0
3.5
3.0
2.5
2.0
1.5
1.0
0.5
0.0
-0.5
-1.0
f2 (ppm)

1-Benzyl 4-isopropyl (*R*)-2-(4-acetoxy-3-methoxyphenyl)-3-formylquinoline-1,4(2H)-dicarboxylate (–)-18n

^{1}H NMR (500 MHz, $CDCl_3$)

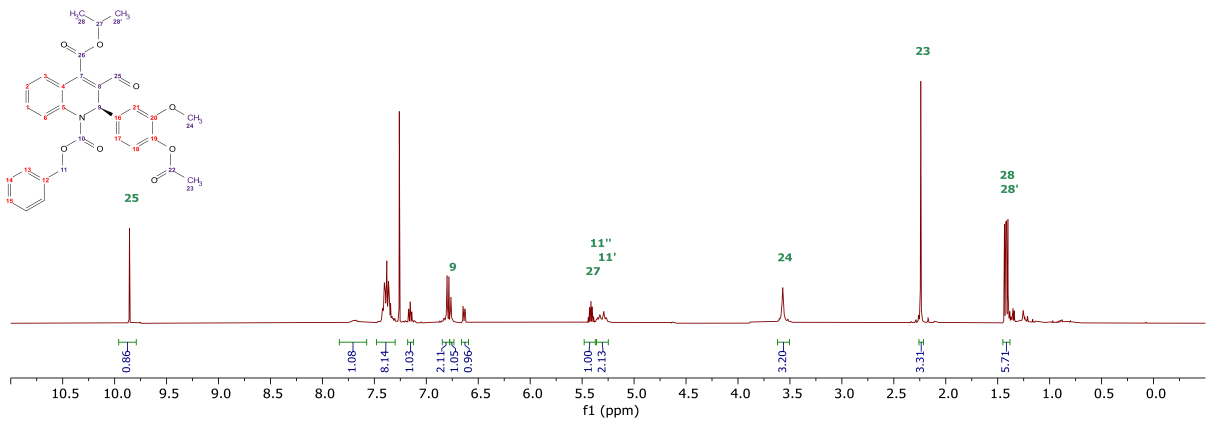

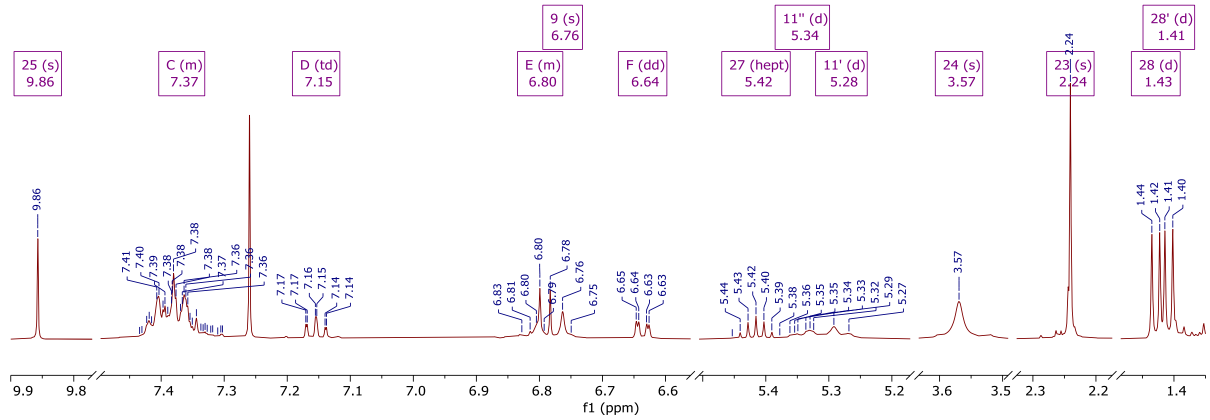

^{13}C NMR (126 MHz, $CDCl_3$)

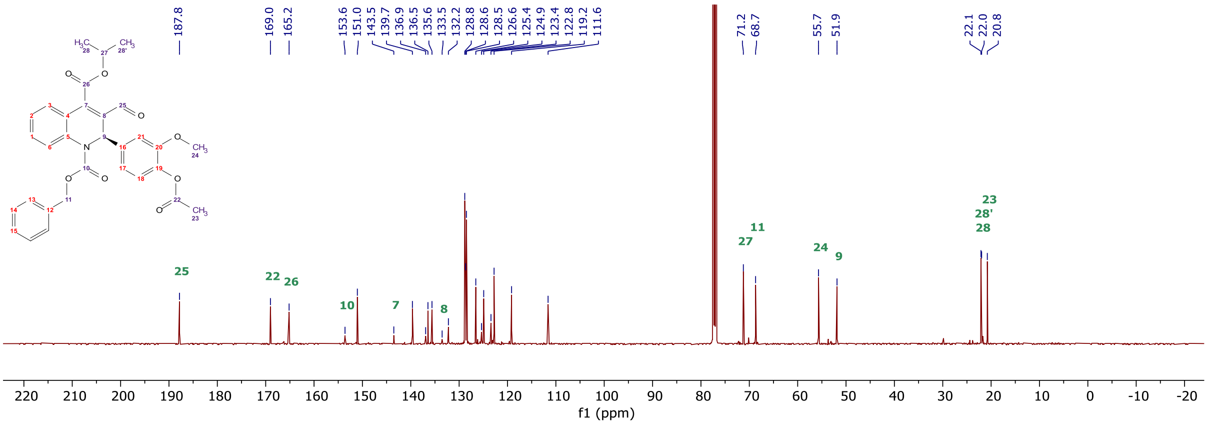

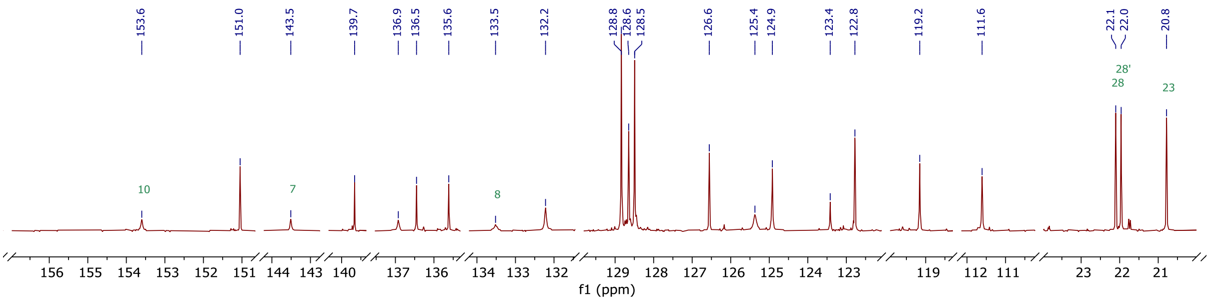

$^{1}H/^{1}H$ COSY

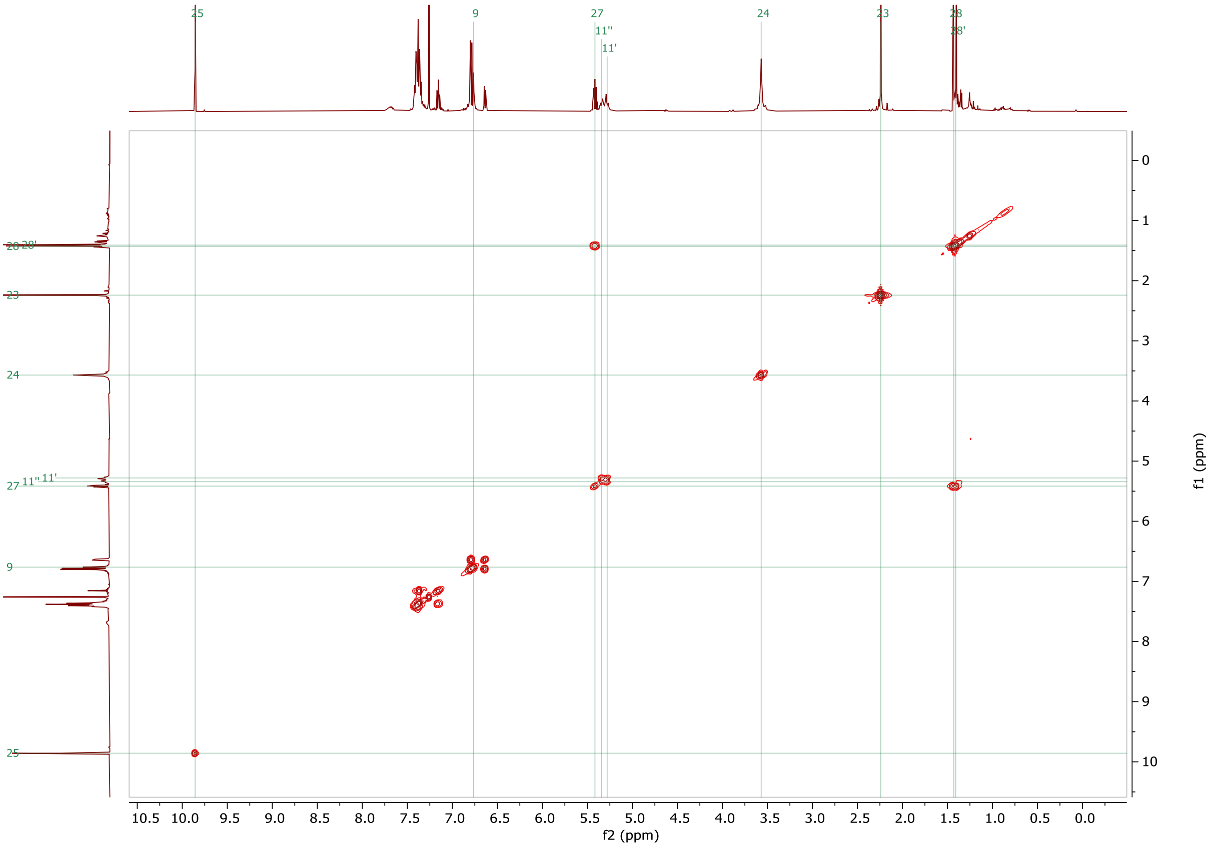

$^{1}H/^{13}C$ HSQC

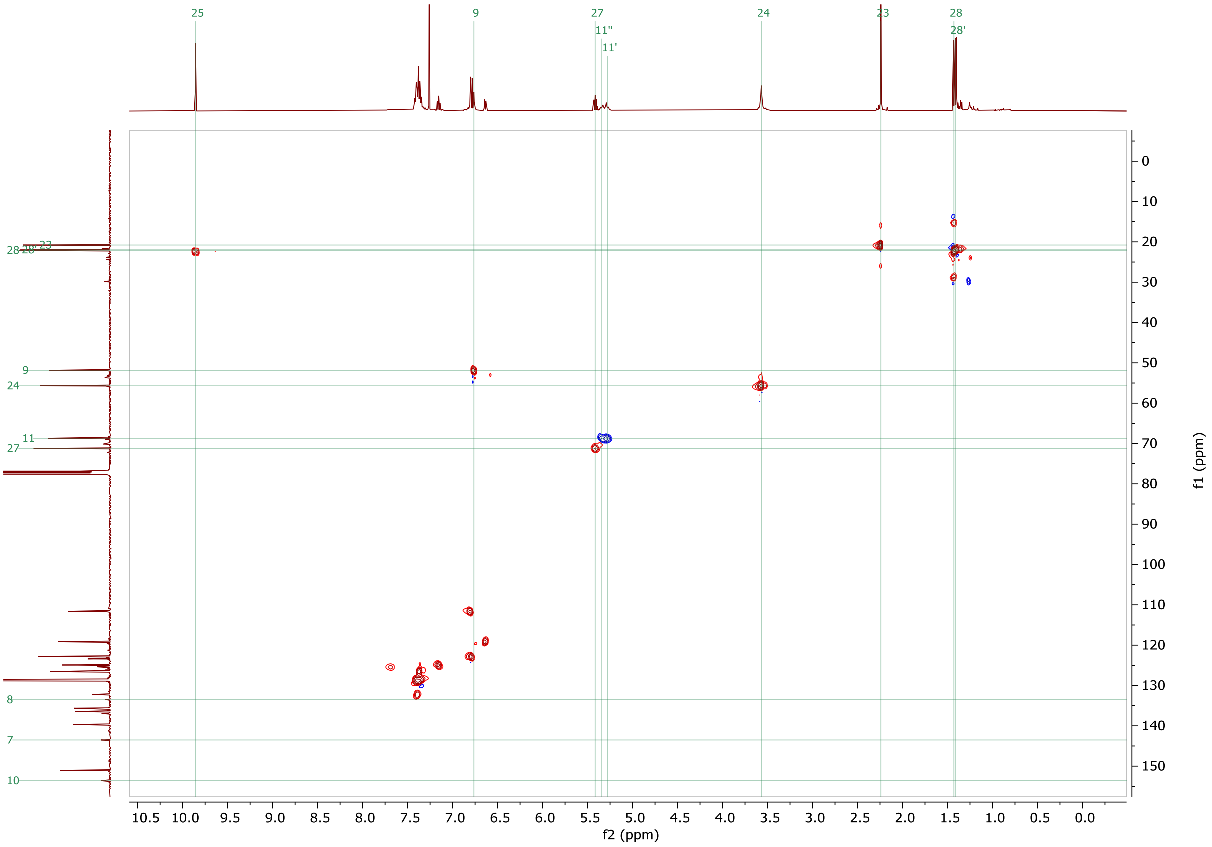

$^{1}H/^{13}C$ HMBC

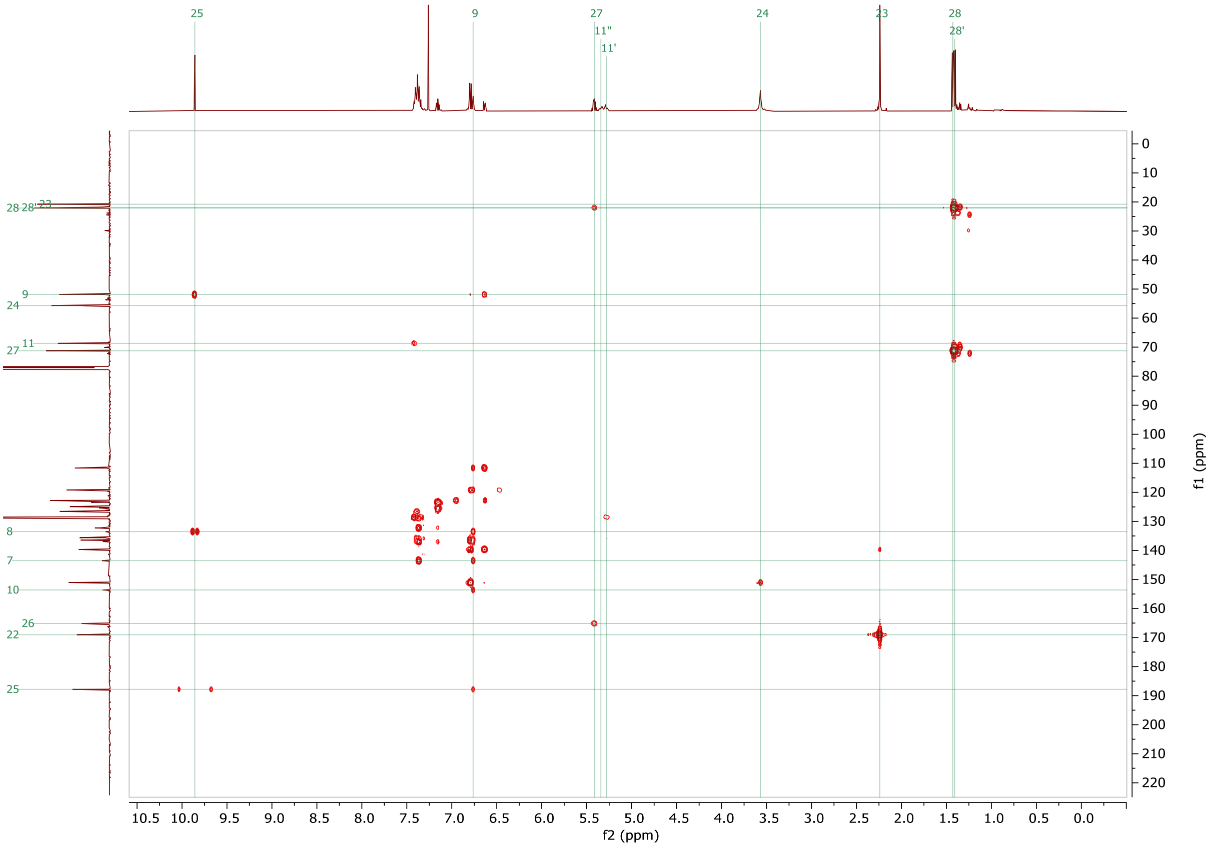

1-Benzyl 4-isopropyl (*R*)-3-formyl-2-(4-nitrophenyl)quinoline-1,4(2H)-dicarboxylate 18m

^{1}H NMR (500 MHz, $CDCl_3$)

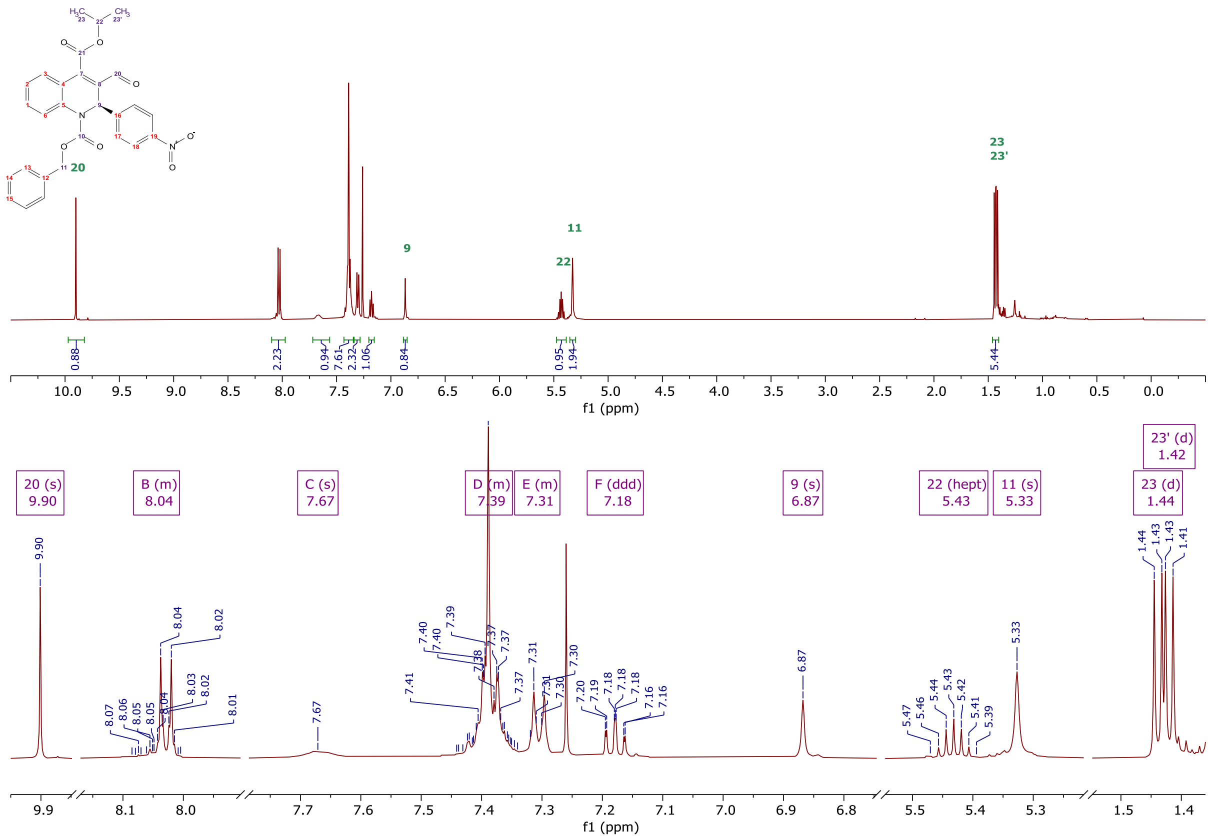

^{13}C NMR (126 MHz, $CDCl_3$)

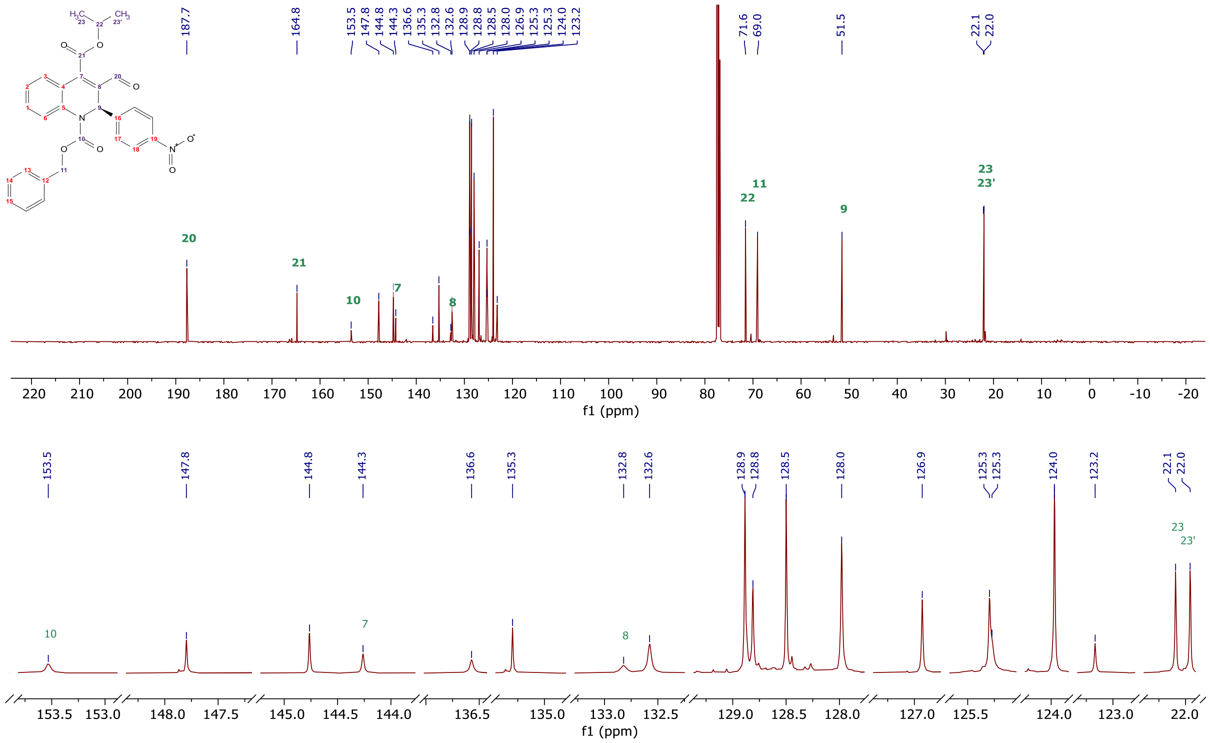

$^{1}H/^{1}H$ COSY

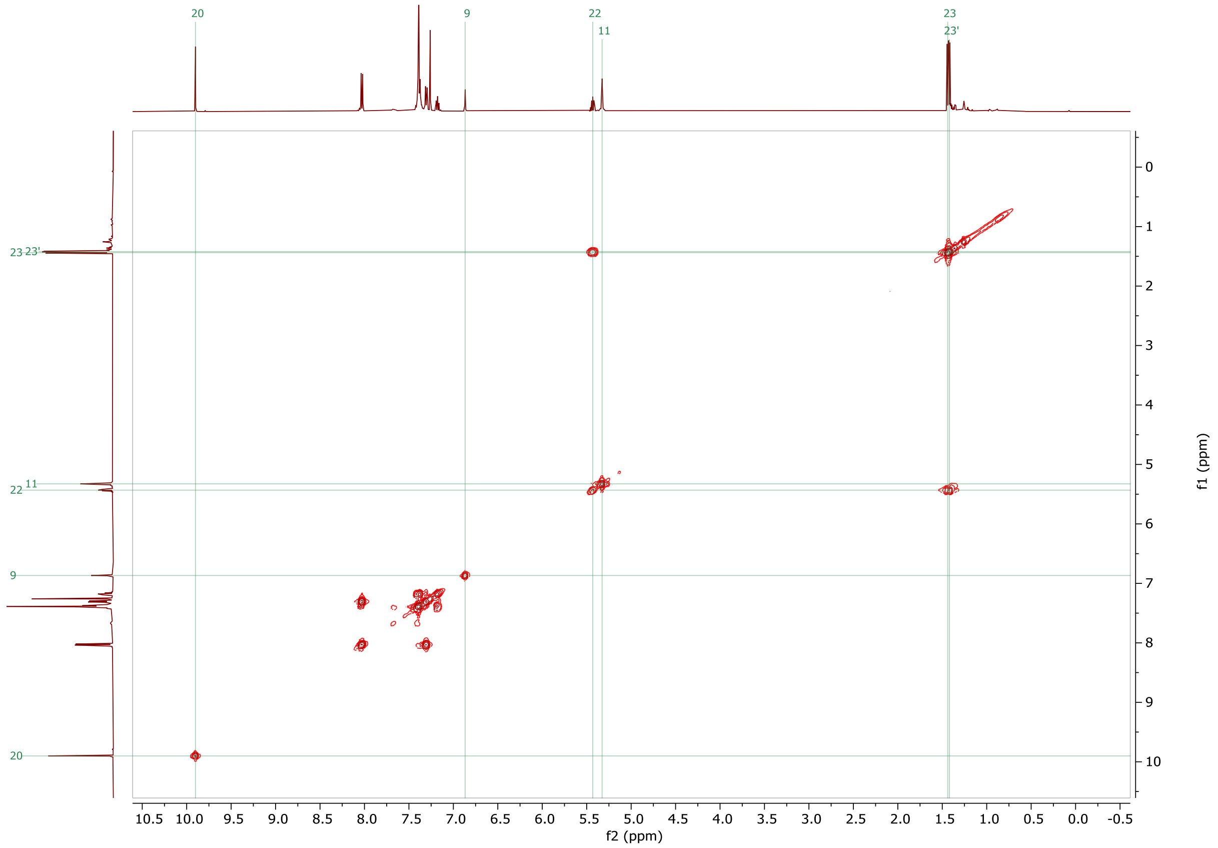

$^{1}H/^{13}C$ HSQC

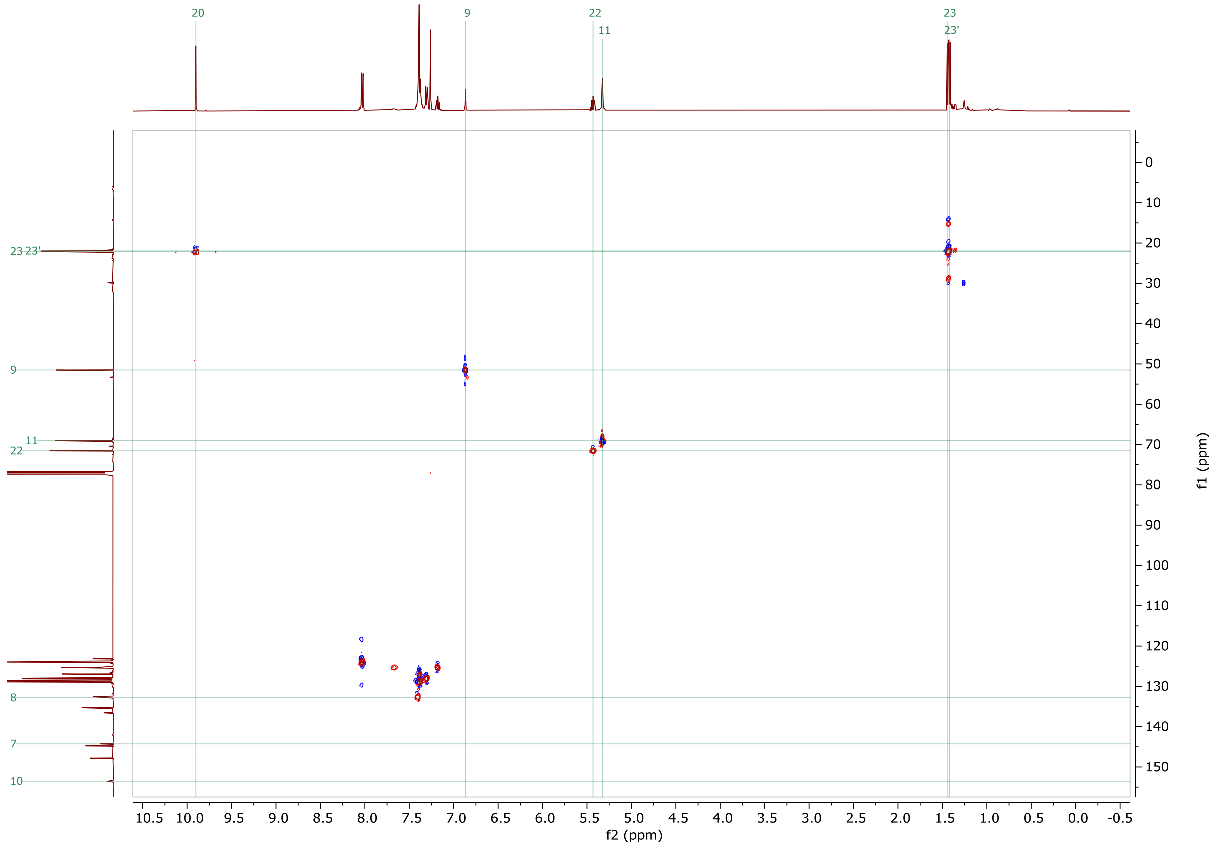

$^{1}H/^{13}C$ HMBC

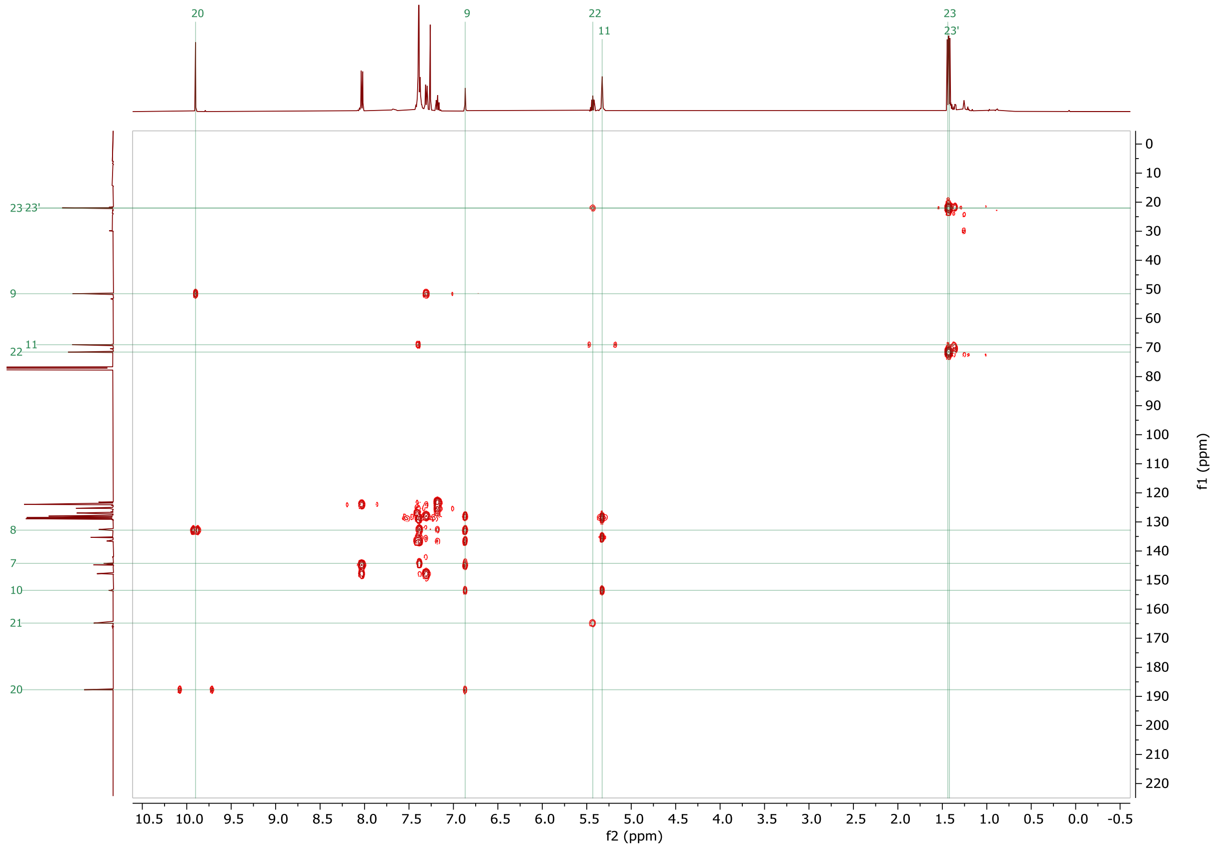

1-Benzyl 4-isopropyl (*R*)-3-formyl-2-(2-nitrophenyl)quinoline-1,4(2H)-dicarboxylate (–)-18o

^{1}H NMR (500 MHz, $CDCl_3$)

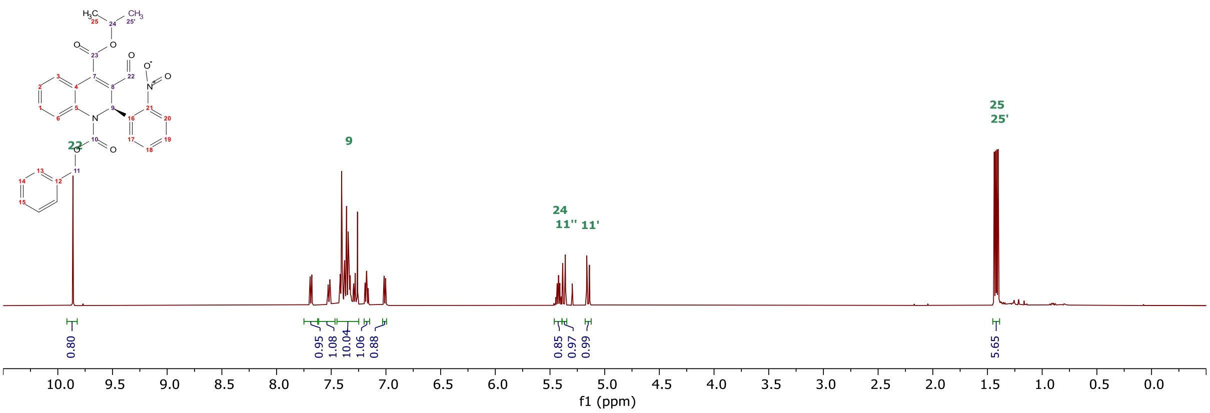

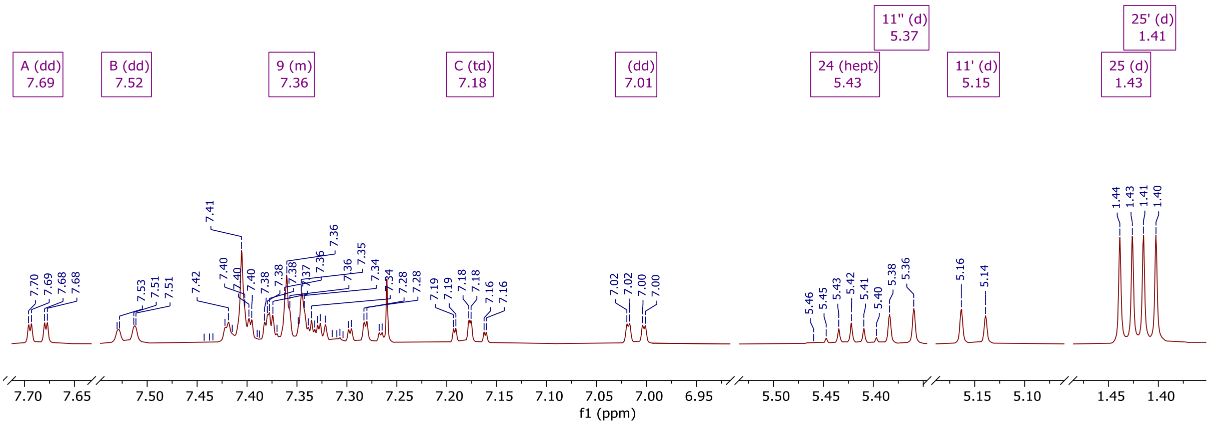

^{13}C NMR (126 MHz, $CDCl_3$)

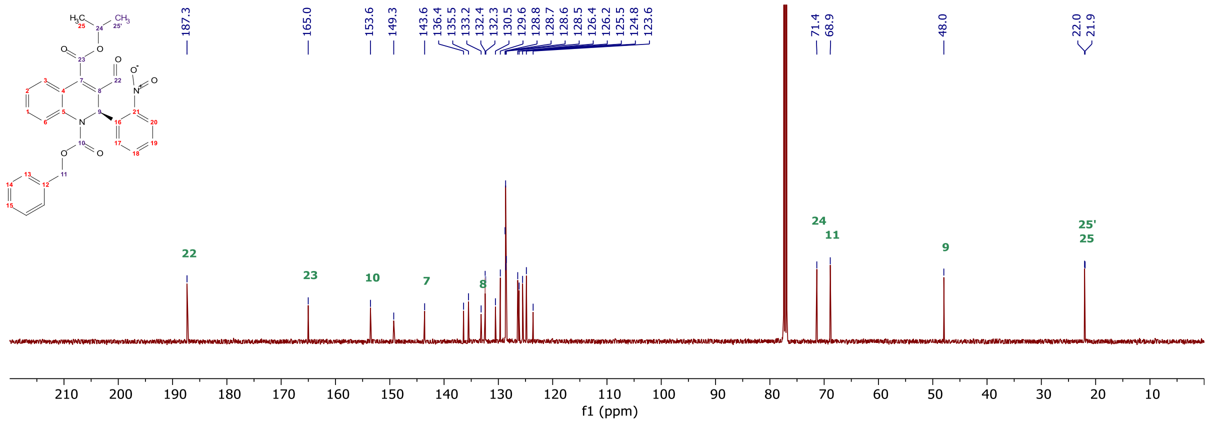

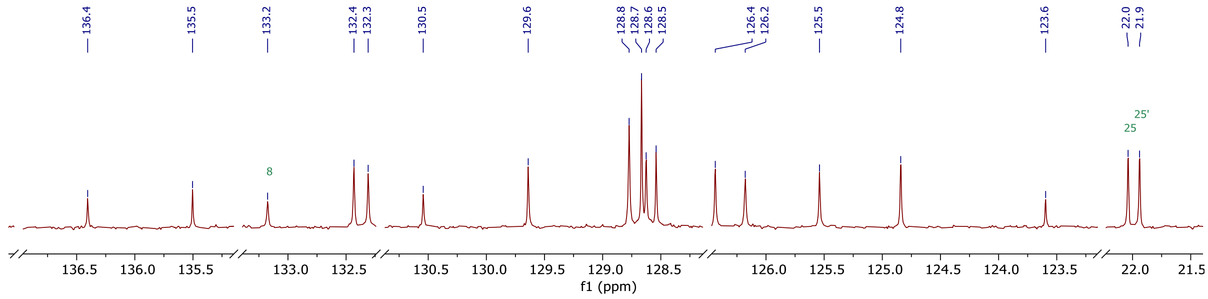

$^{1}H/^{1}H$ COSY

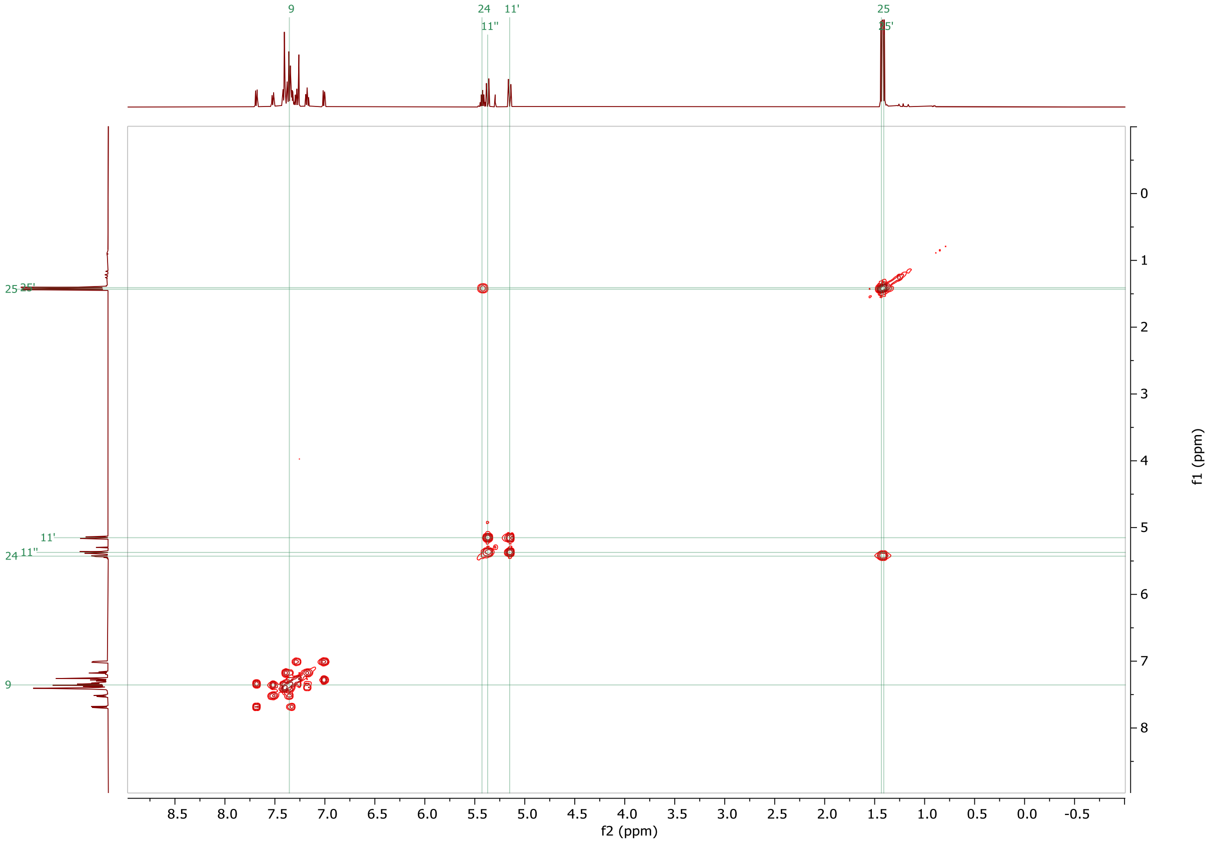

$^{1}H/^{13}C$ HSQC

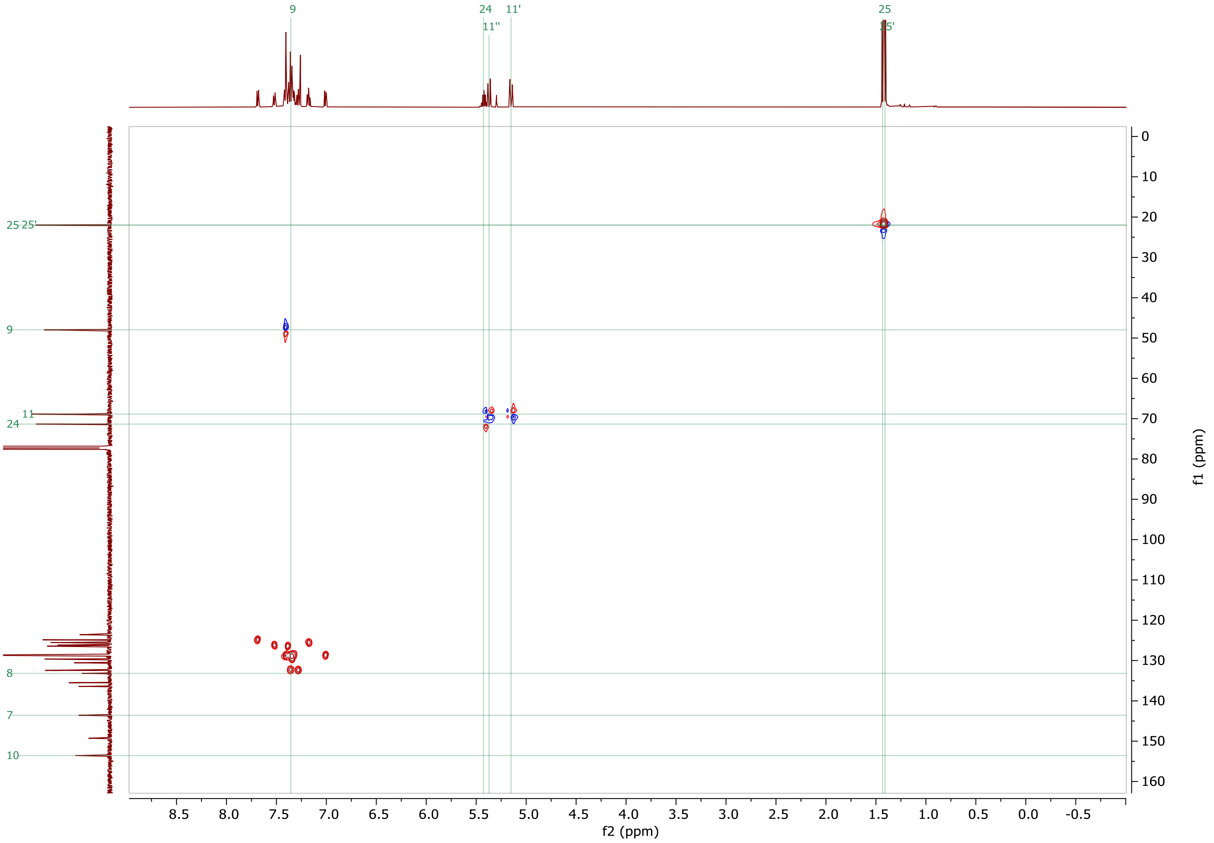

$^{1}H/^{13}C$ HMBC

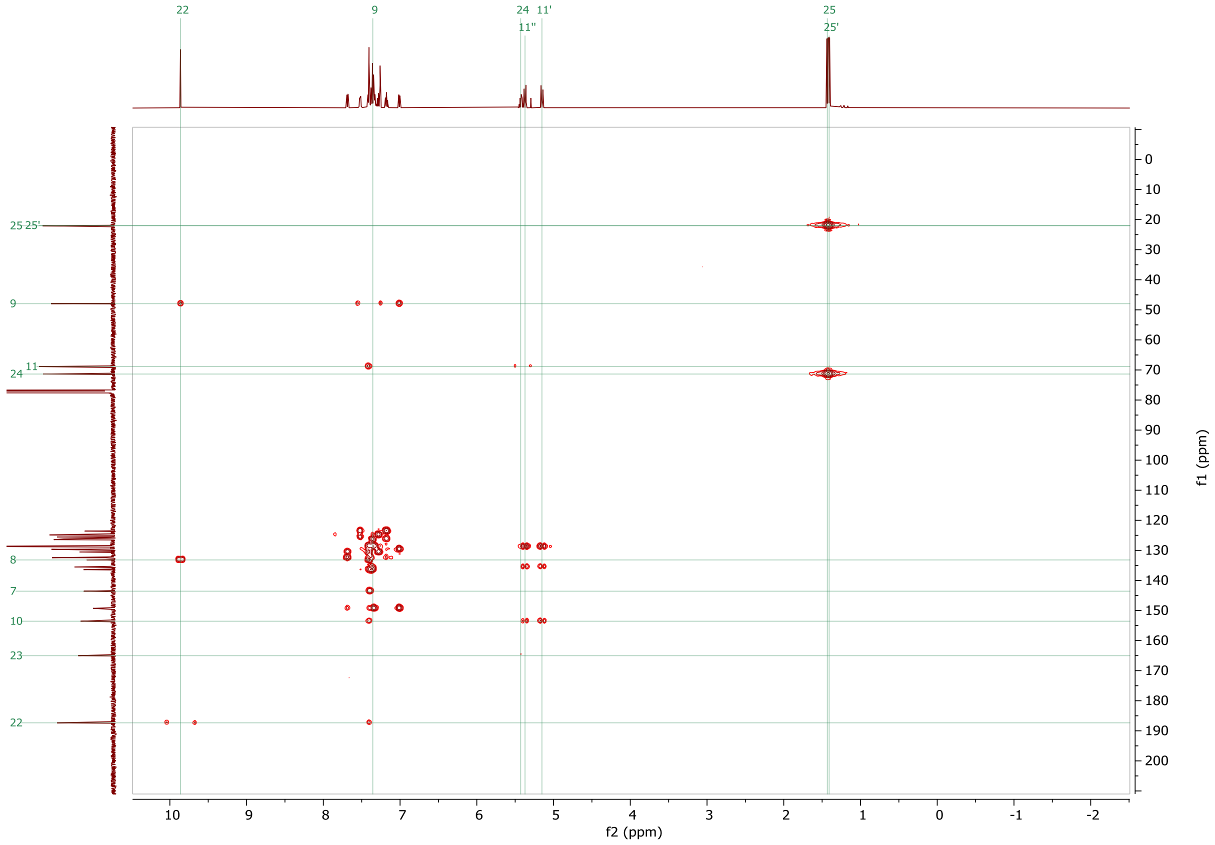

1-Benzyl 4-isopropyl (*R*)-3-formyl-2-(4-methoxyphenyl)quinoline-1,4(2H)-dicarboxylate (–)-18l

^{1}H NMR (500 MHz, $CDCl_3$)

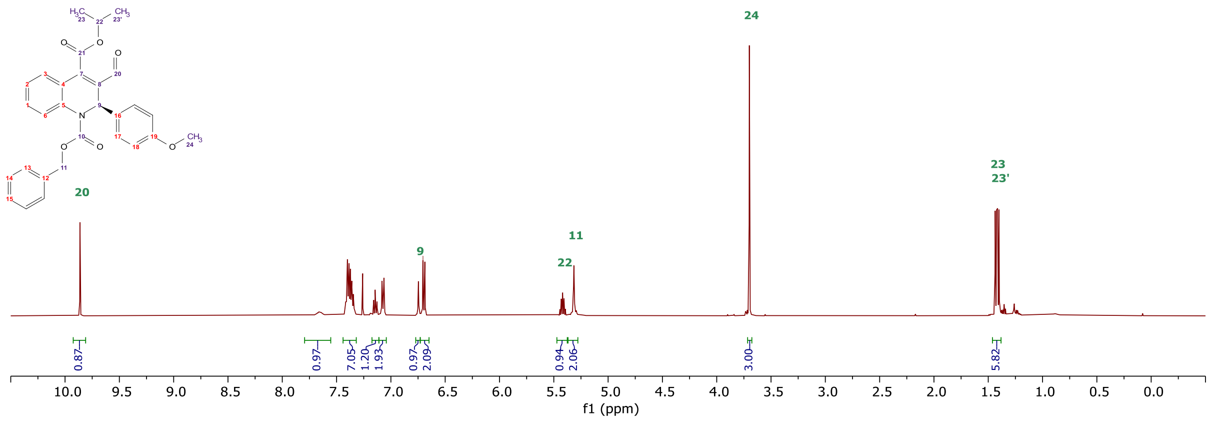

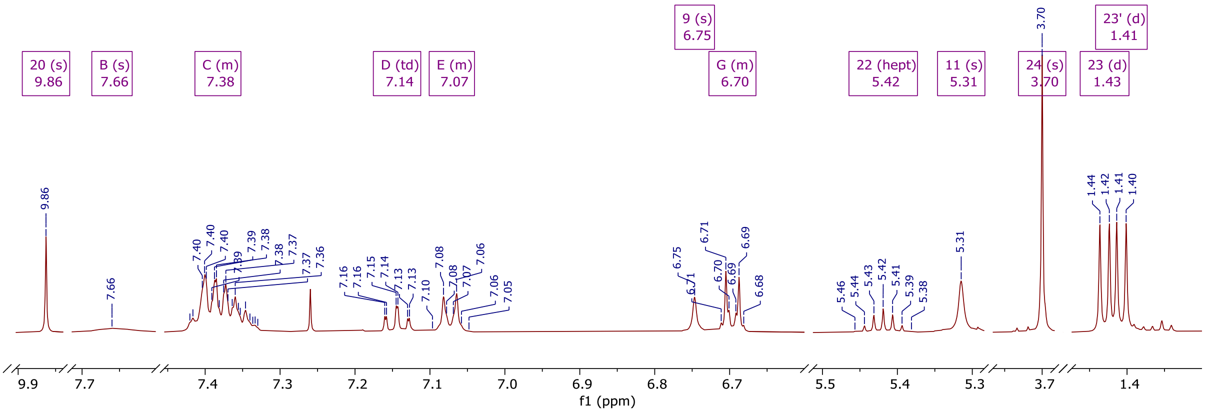

^{13}C NMR (126 MHz, $CDCl_3$)

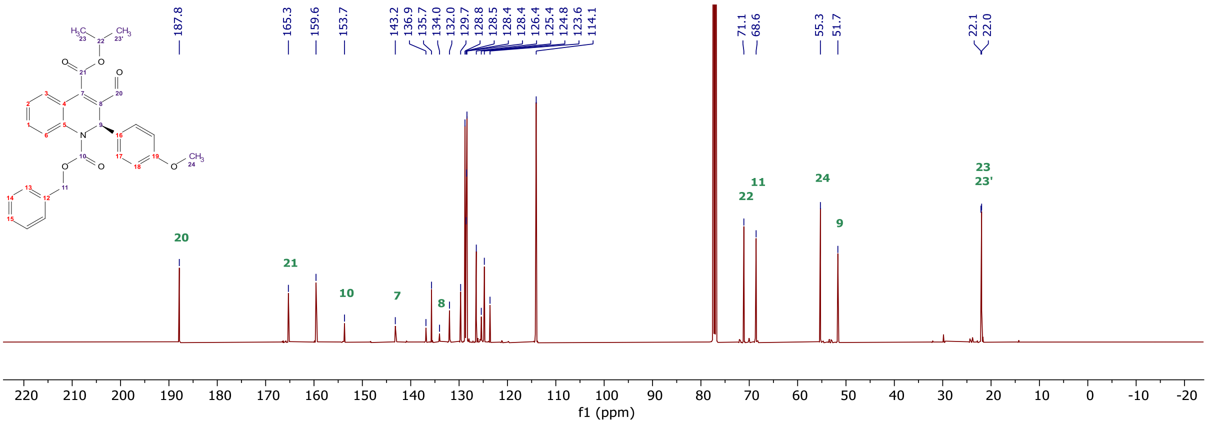

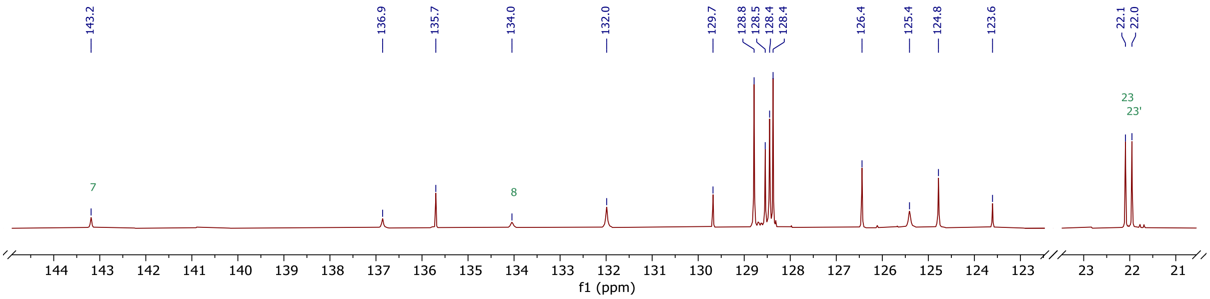

$^1H/^1H$ COSY

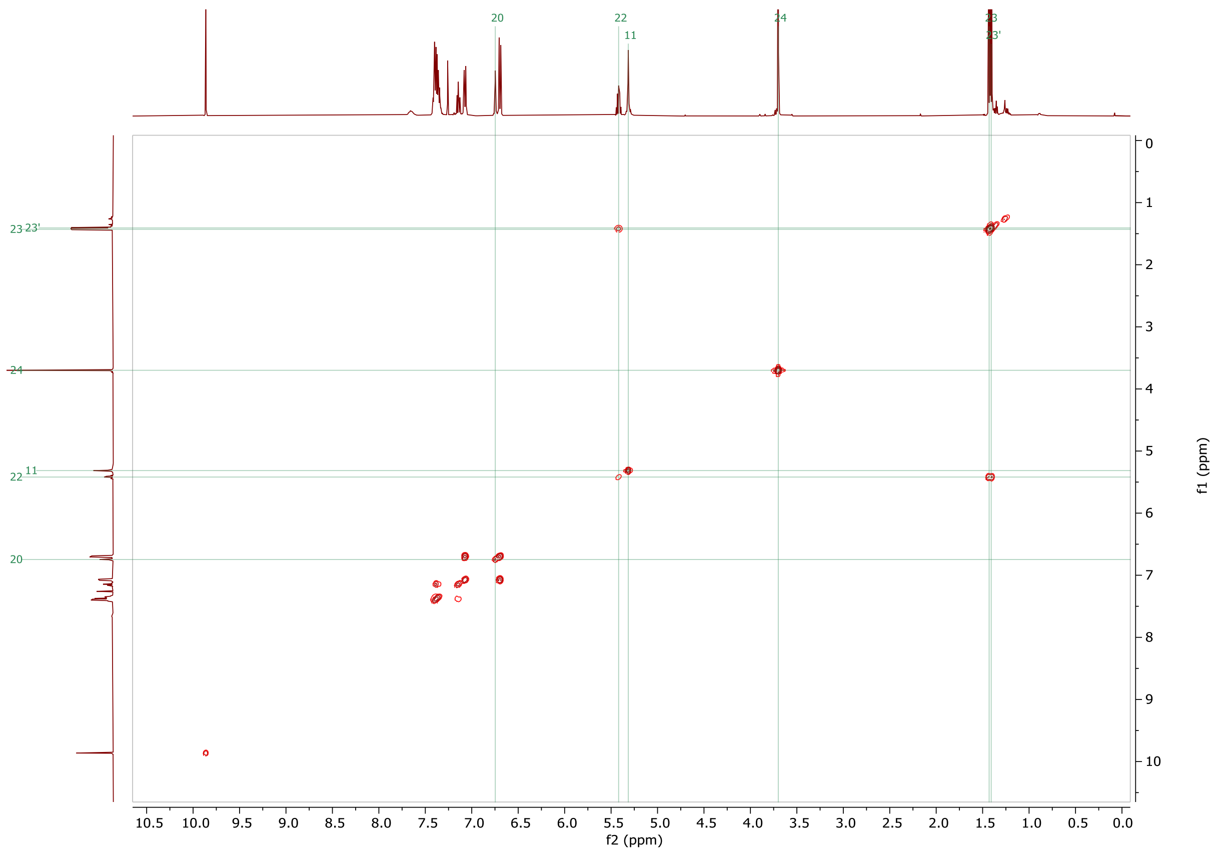

$^1H/^{13}C$ HSQC

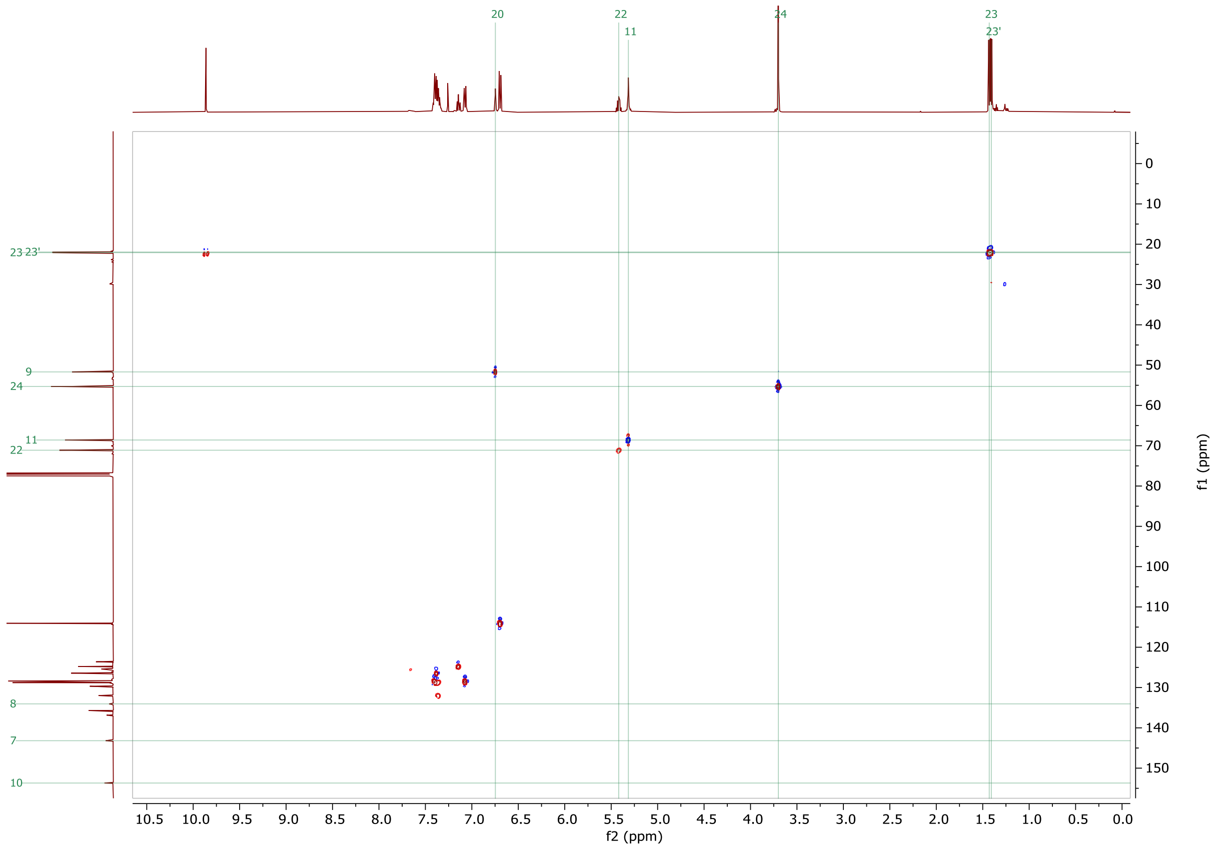

$^1H/^{13}C$ HMBC

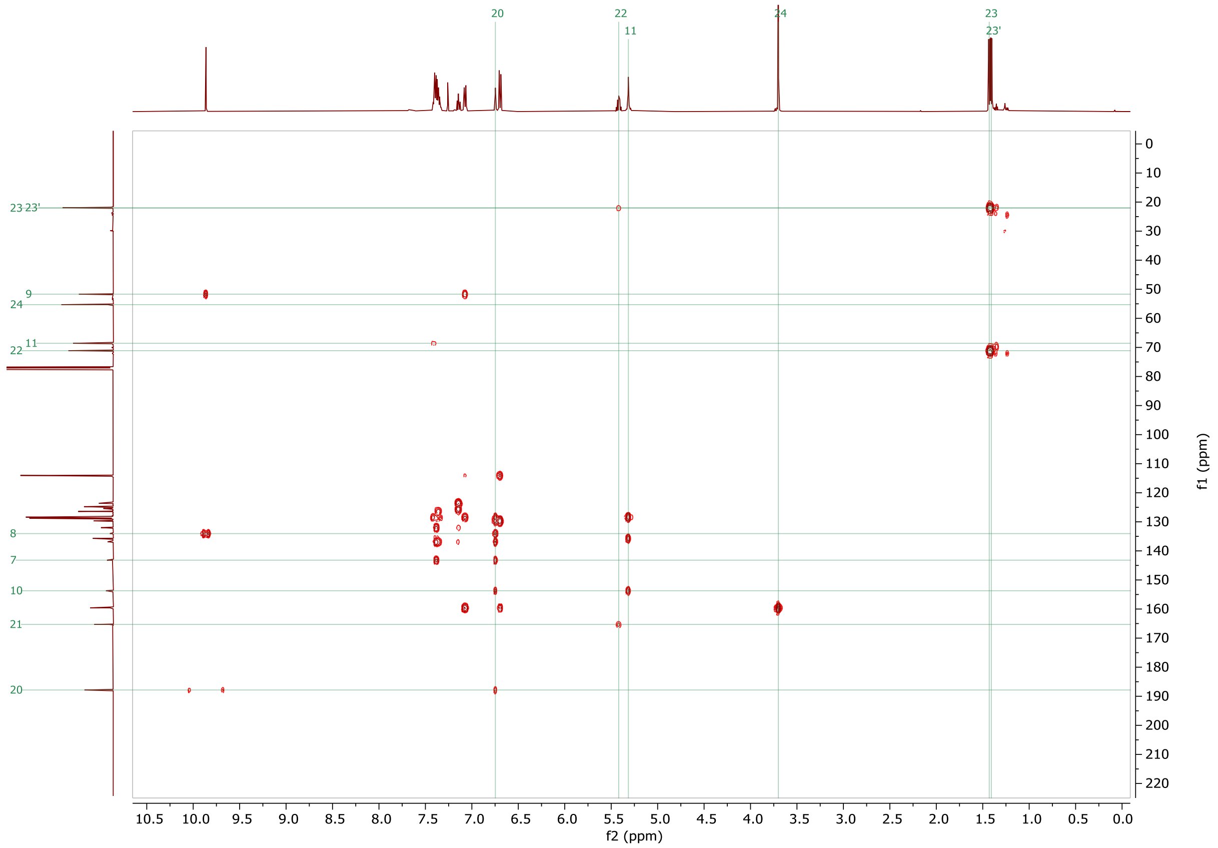

1-Benzyl 4-isopropyl (*R*)-3-formyl-2-(2-methoxyphenyl)quinoline-1,4(2H)-dicarboxylate (–)-18p

^{1}H NMR (500 MHz, $CDCl_3$)

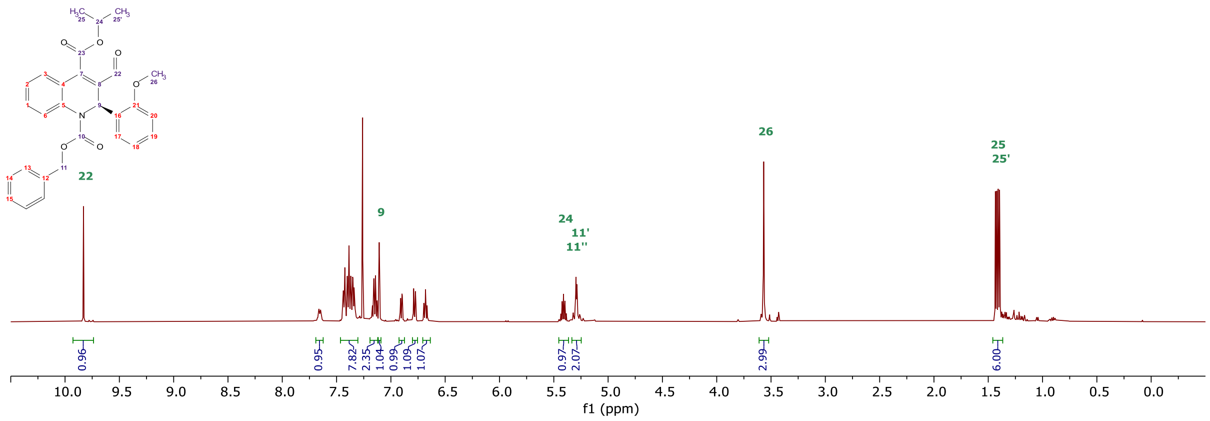

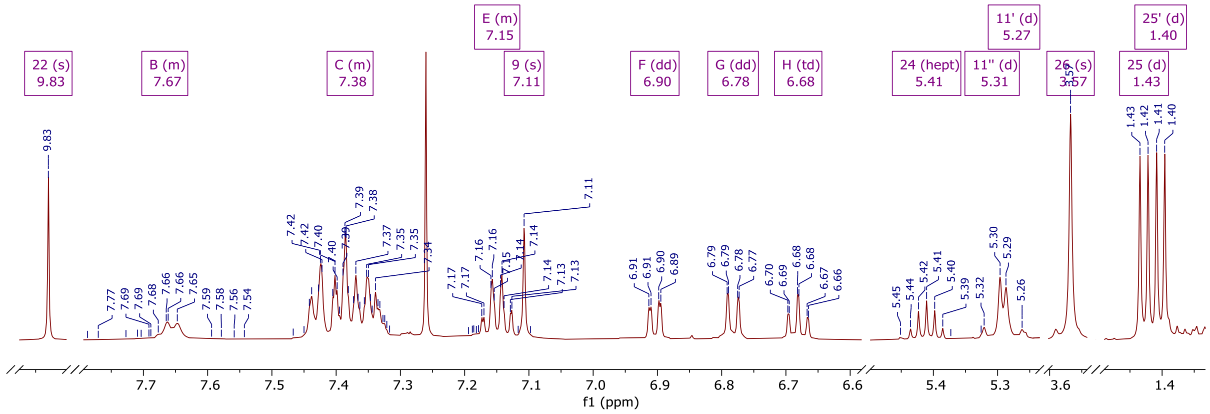

^{13}C NMR (126 MHz, $CDCl_3$)

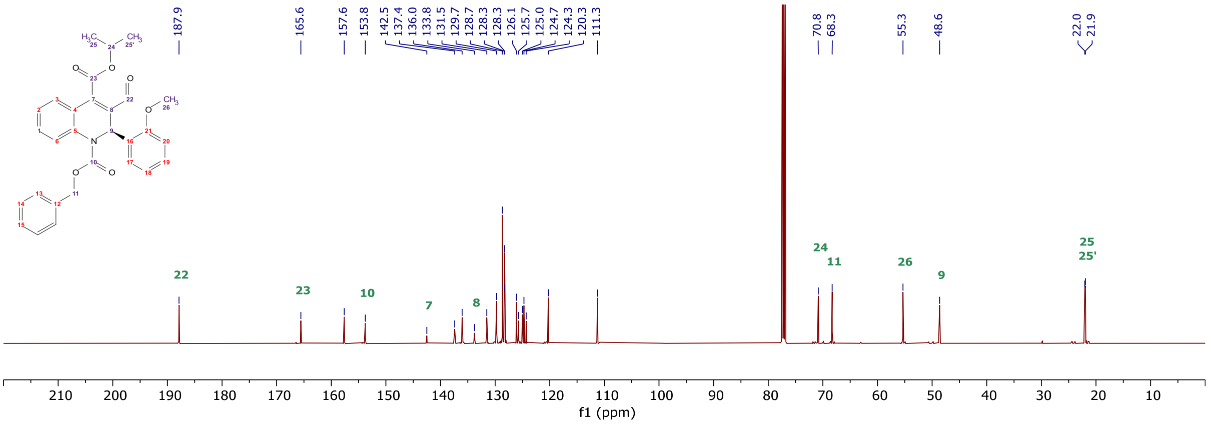

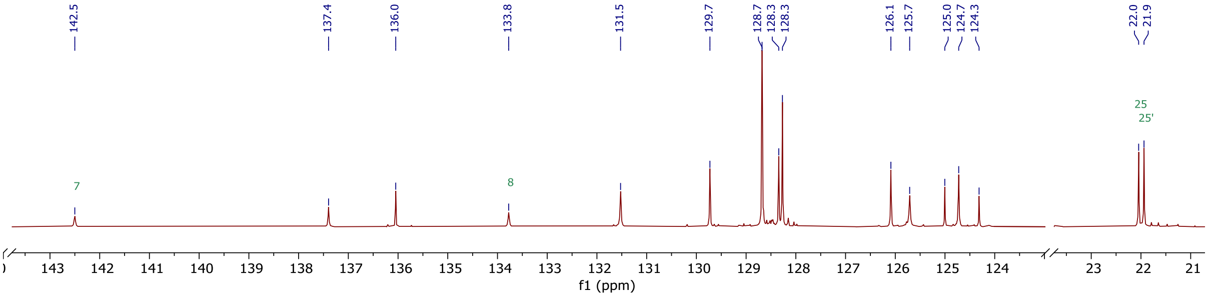

$^{1}H/^{1}H$ COSY

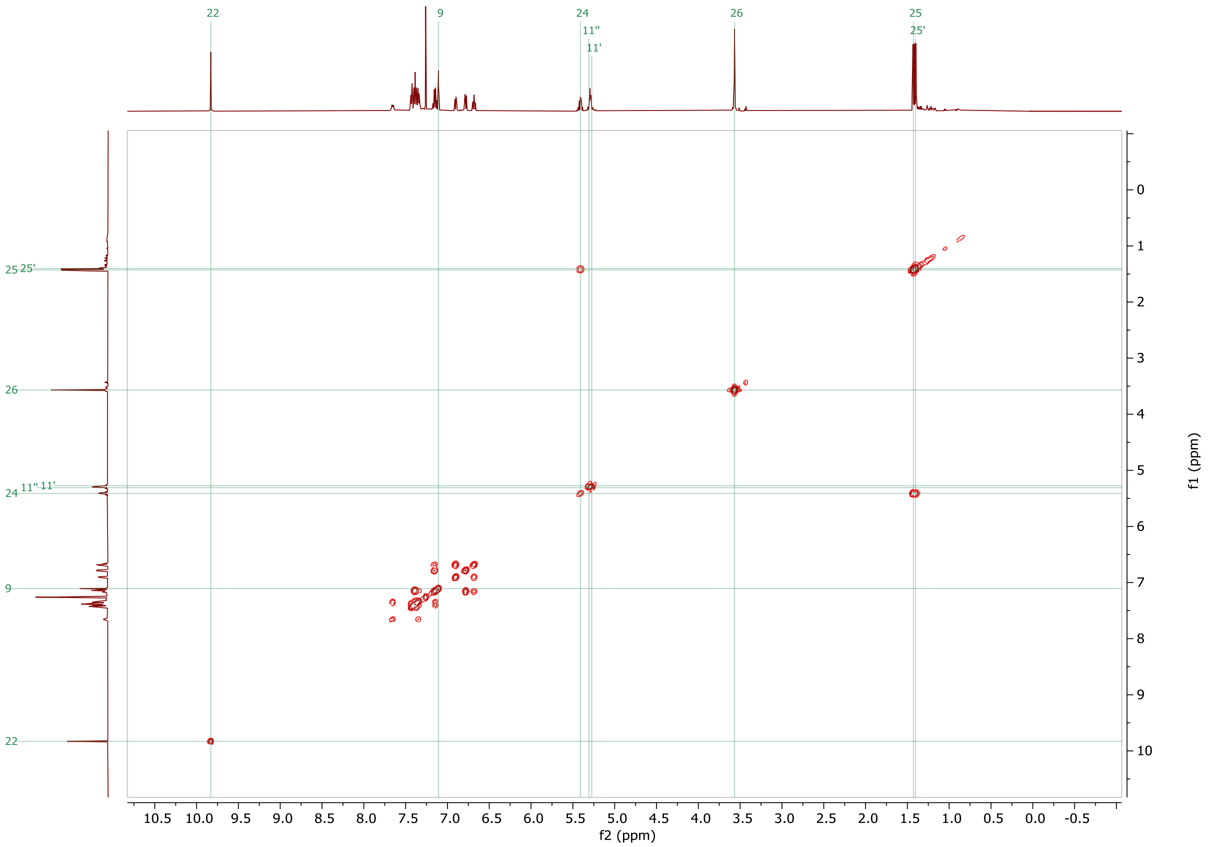

$^{1}H/^{13}C$ HSQC

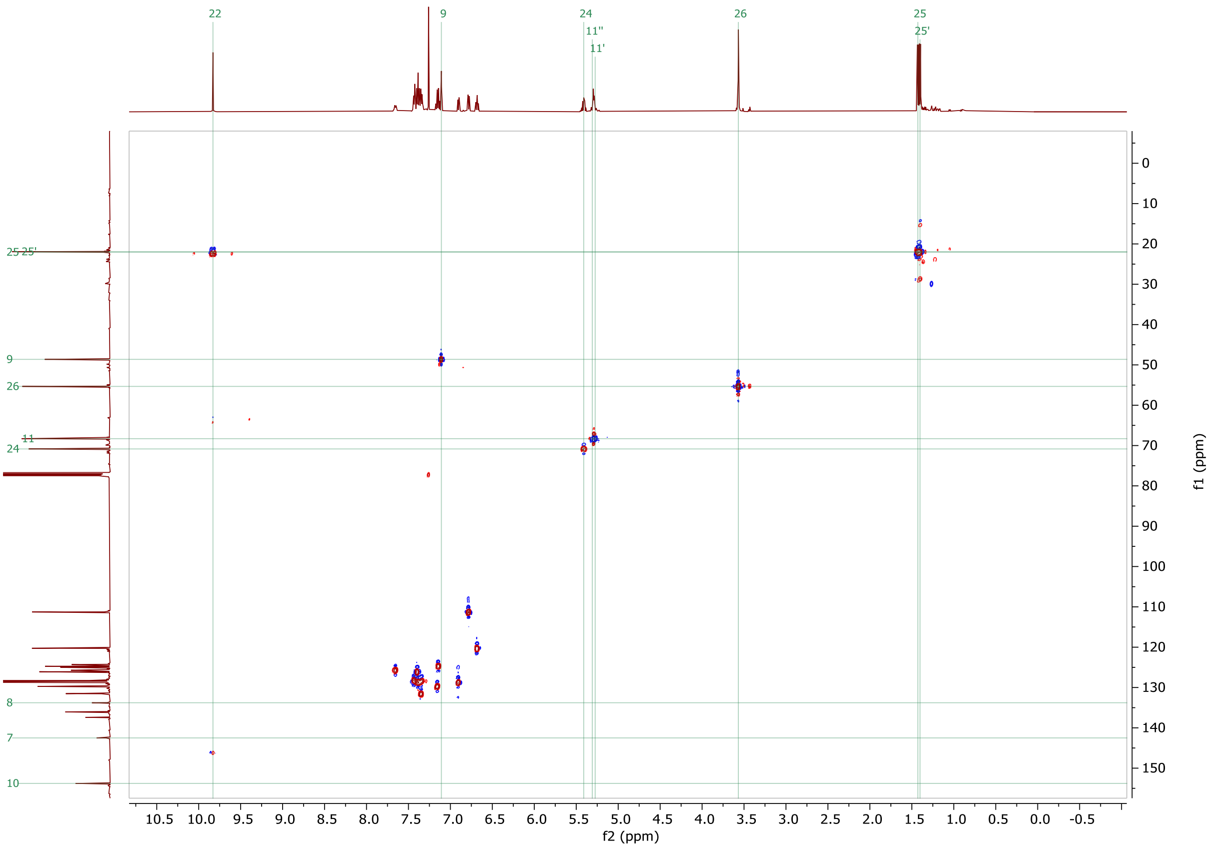

$^{1}H/^{13}C$ HMBC

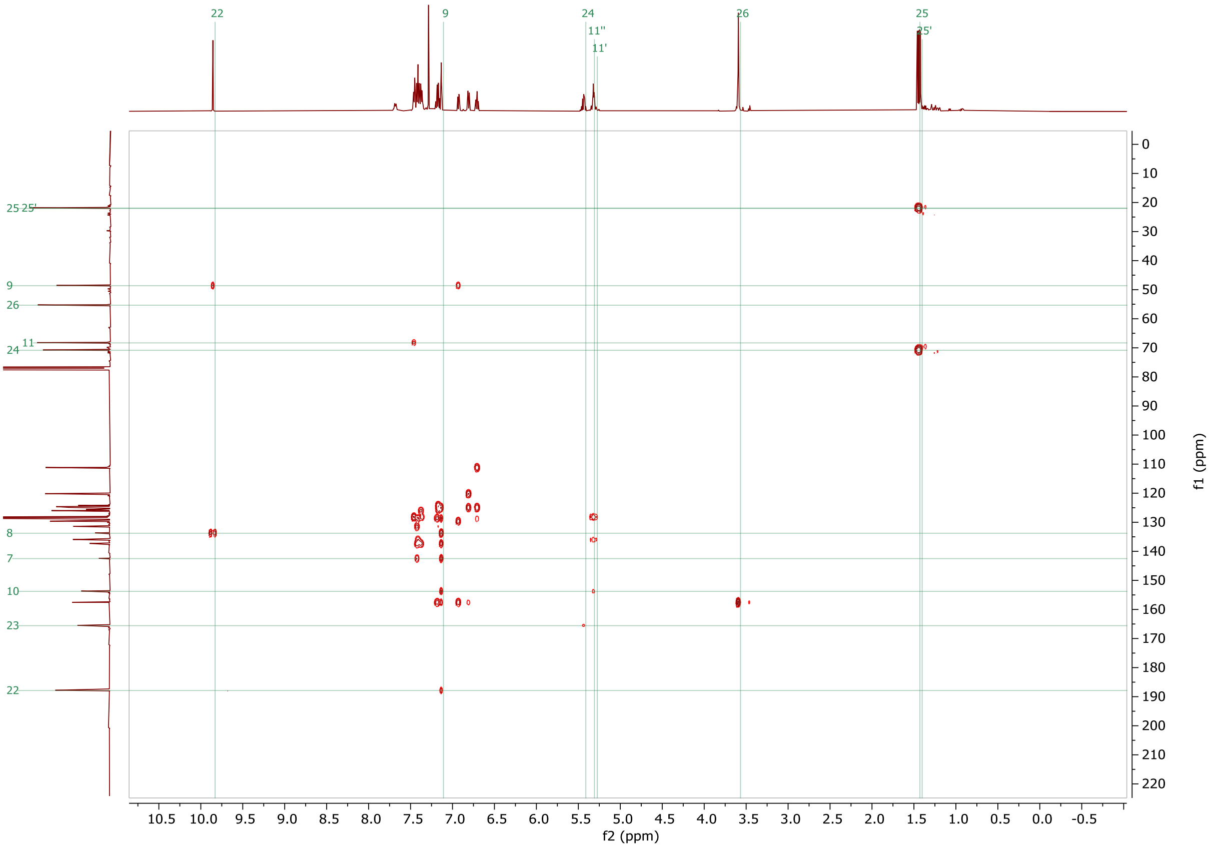

1-Benzyl 4-isopropyl (*S*)-2-(2-bromophenyl)-3-formylquinoline-1,4(2H)-dicarboxylate (–)-18q

^{1}H NMR (500 MHz, $CDCl_3$)

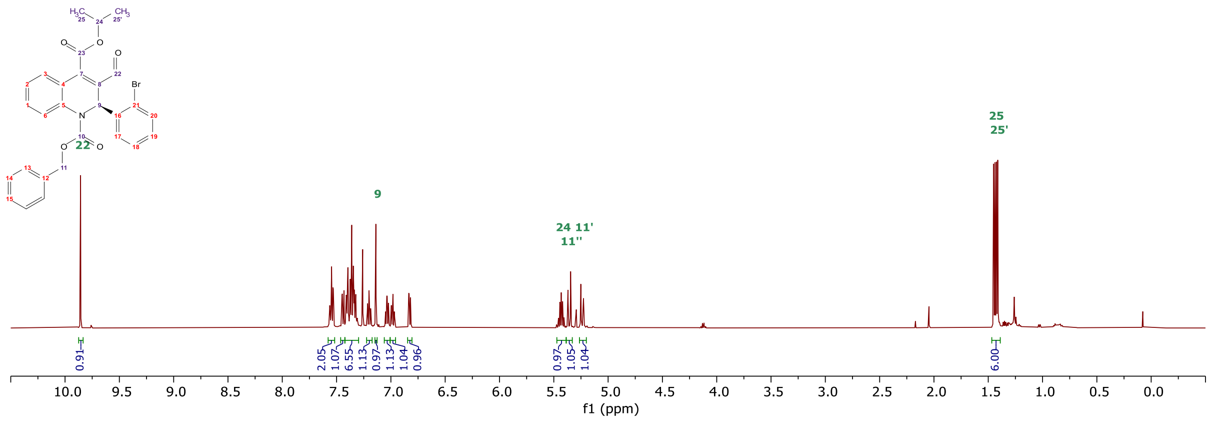

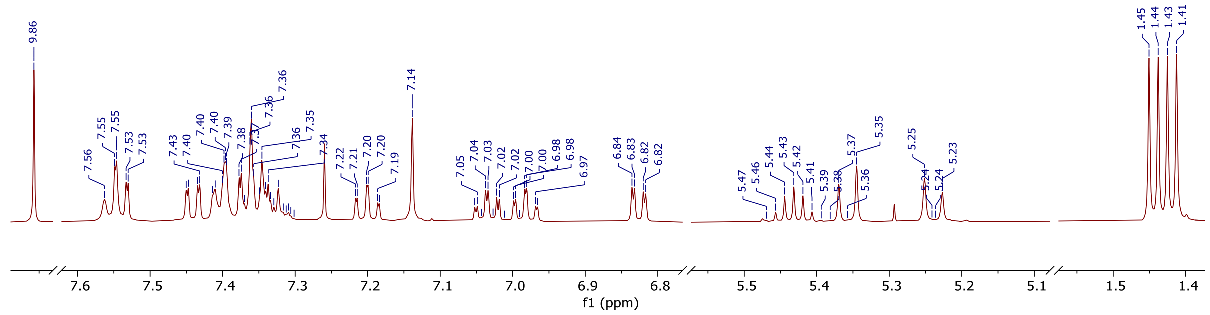

^{13}C NMR (126 MHz, $CDCl_3$)

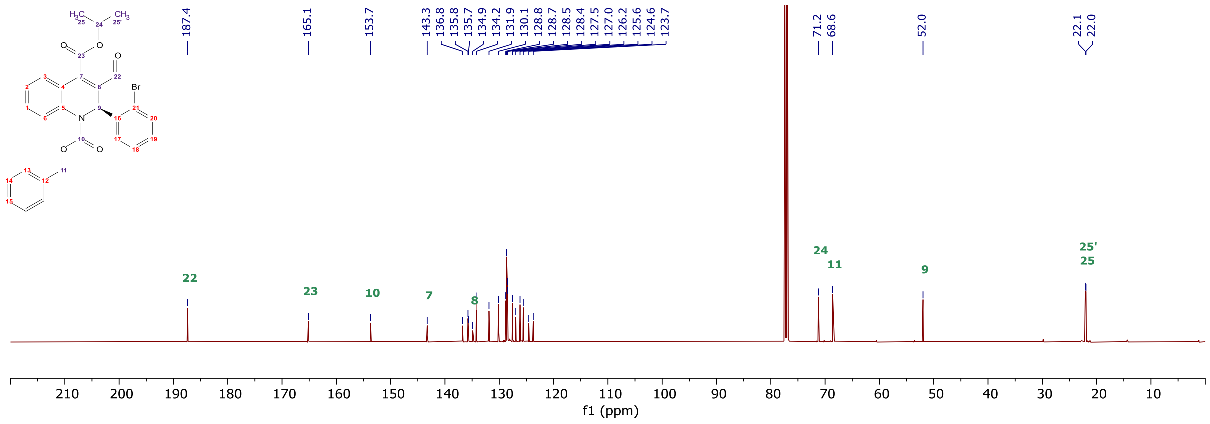

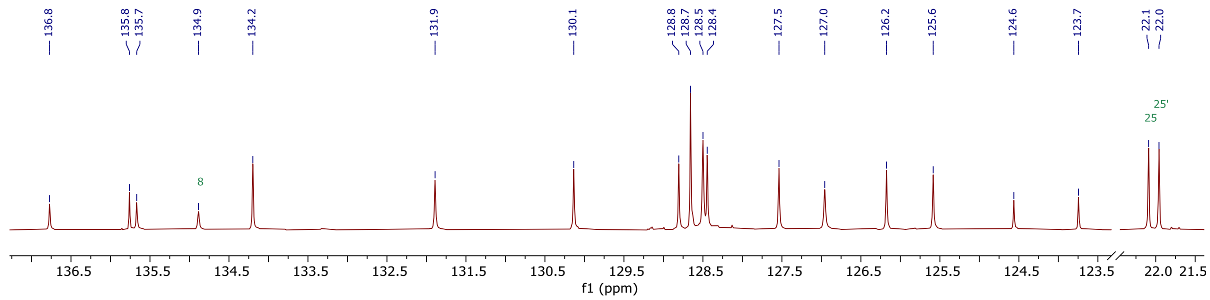

$^{1}H/^{1}H$ COSY

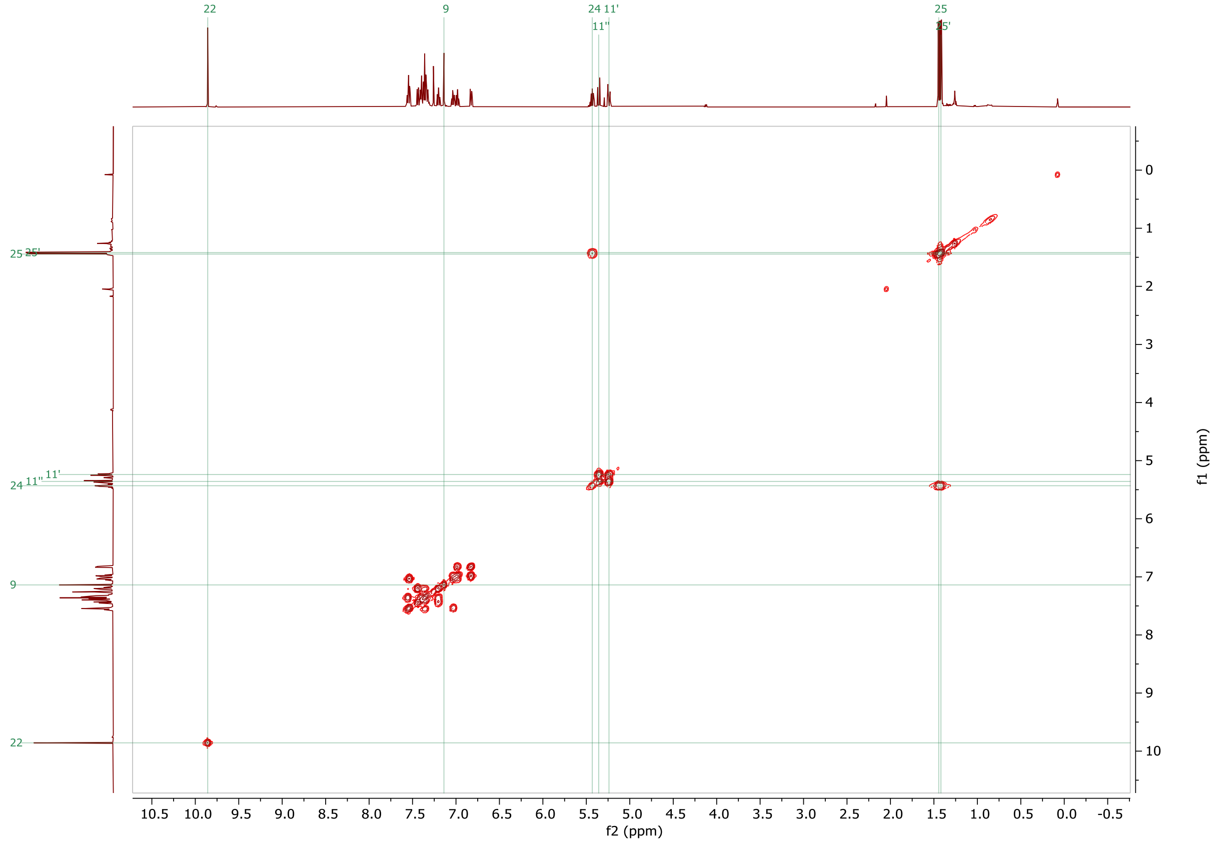

$^{1}H/^{13}C$ HSQC

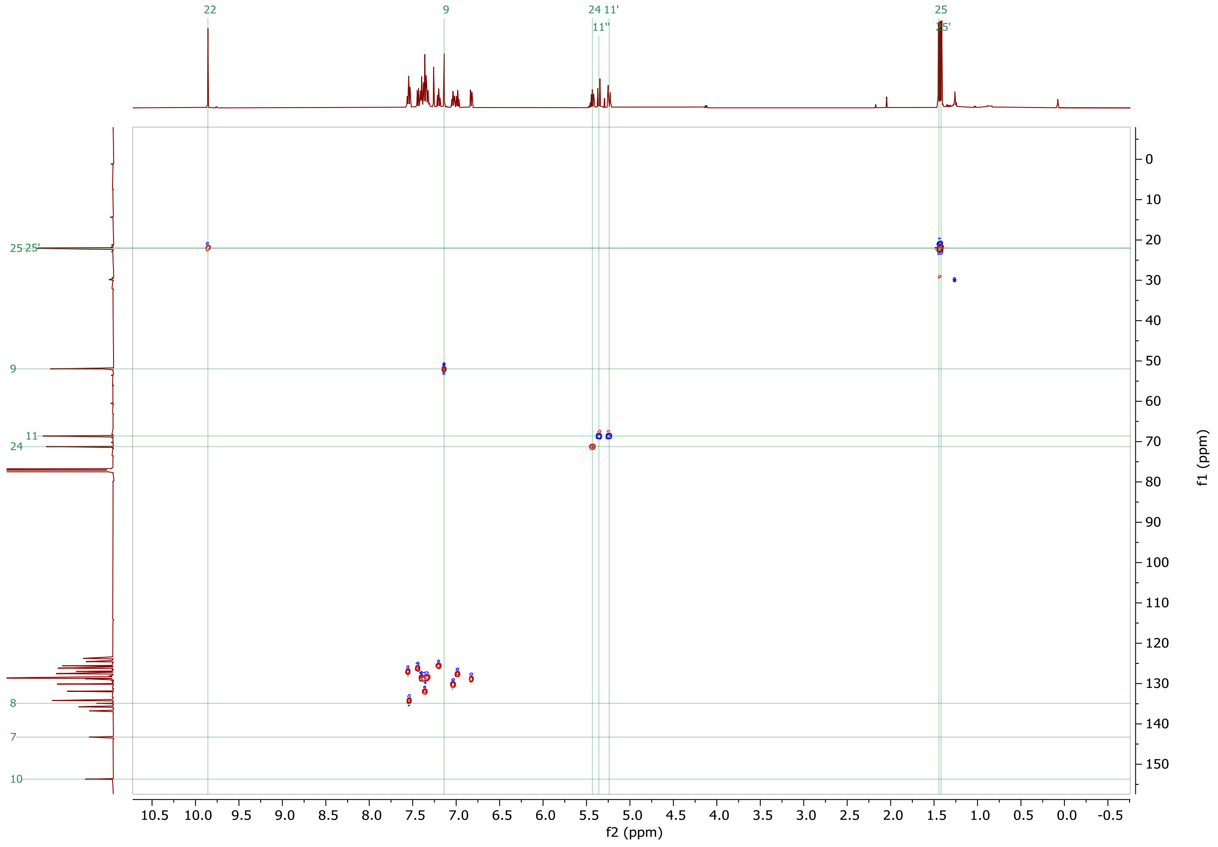

$^1H/^{13}C$ HMBC

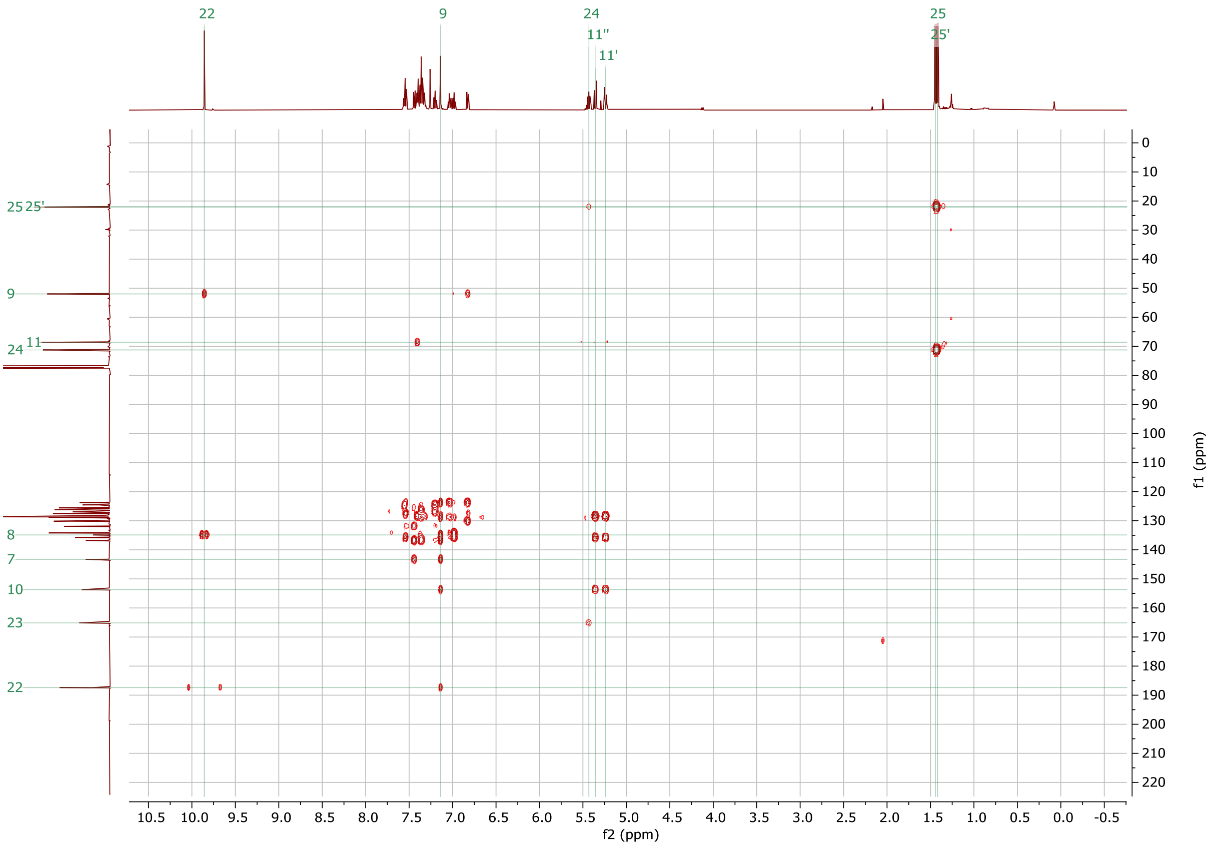

Total Synthesis of Sealutomicin C

1-Benzyl 4-isopropyl (*R*)-2-(2-bromophenyl)-3-formyl-6-methoxyquinoline-1,4(2*H*)-dicarboxylate (+)-18r

^{1}H NMR (500 MHz, $CDCl_3$)

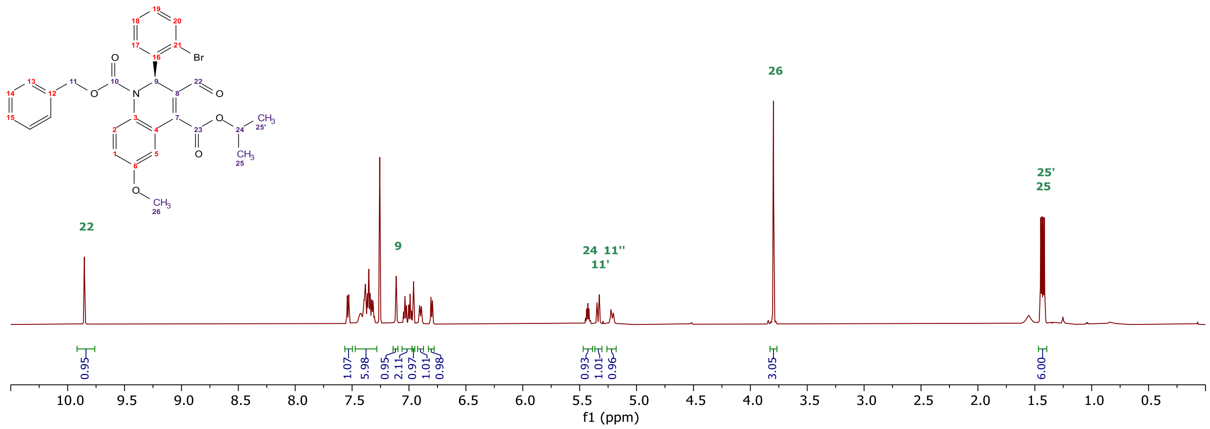

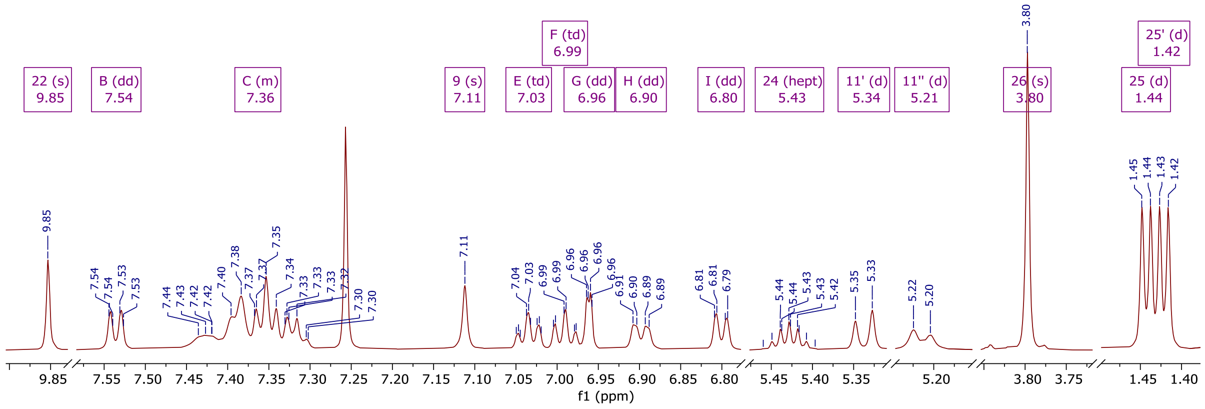

^{13}C NMR (126 MHz, $CDCl_3$)

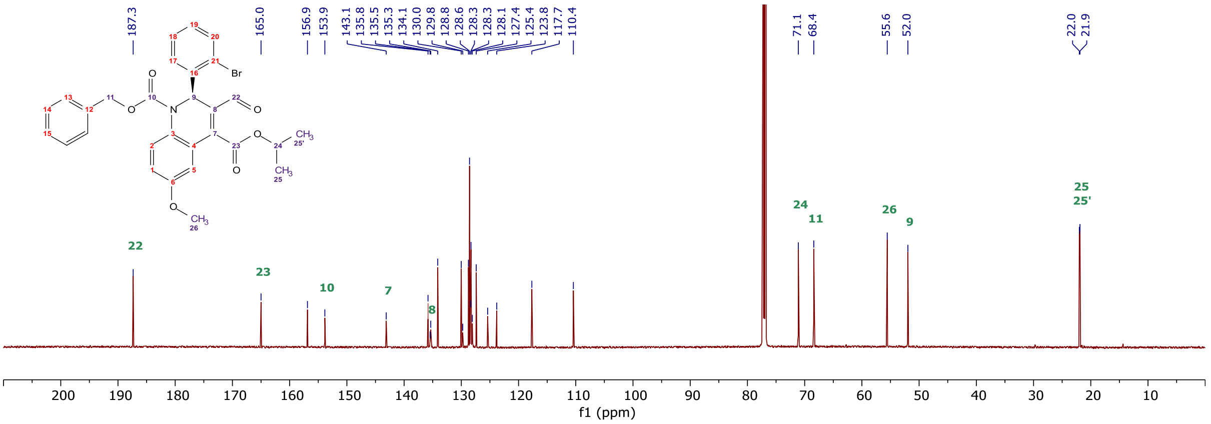

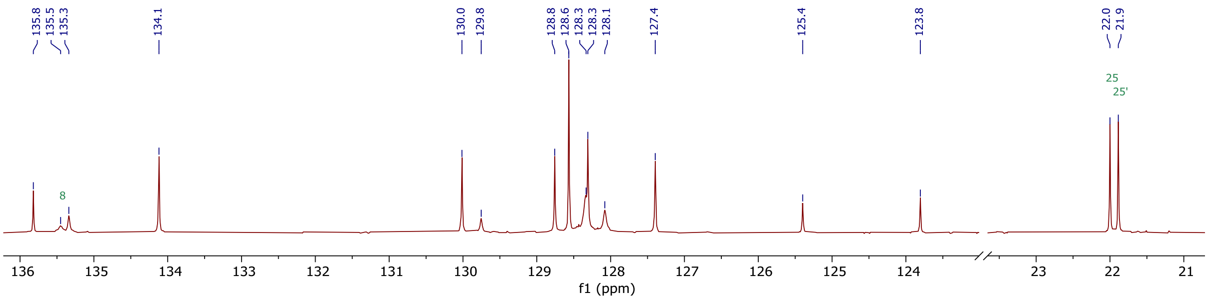

$^{1}H/^{1}H$ COSY

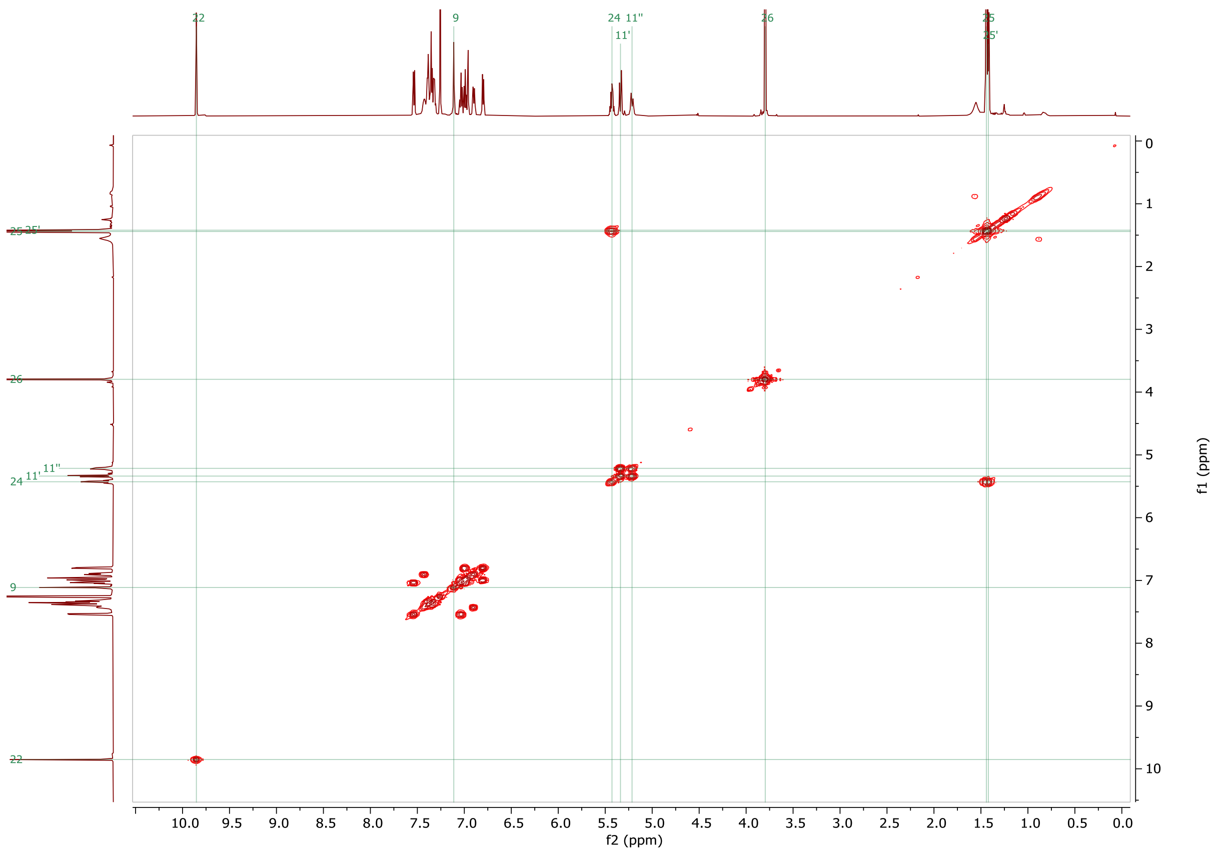

$^{1}H/^{13}C$ HSQC

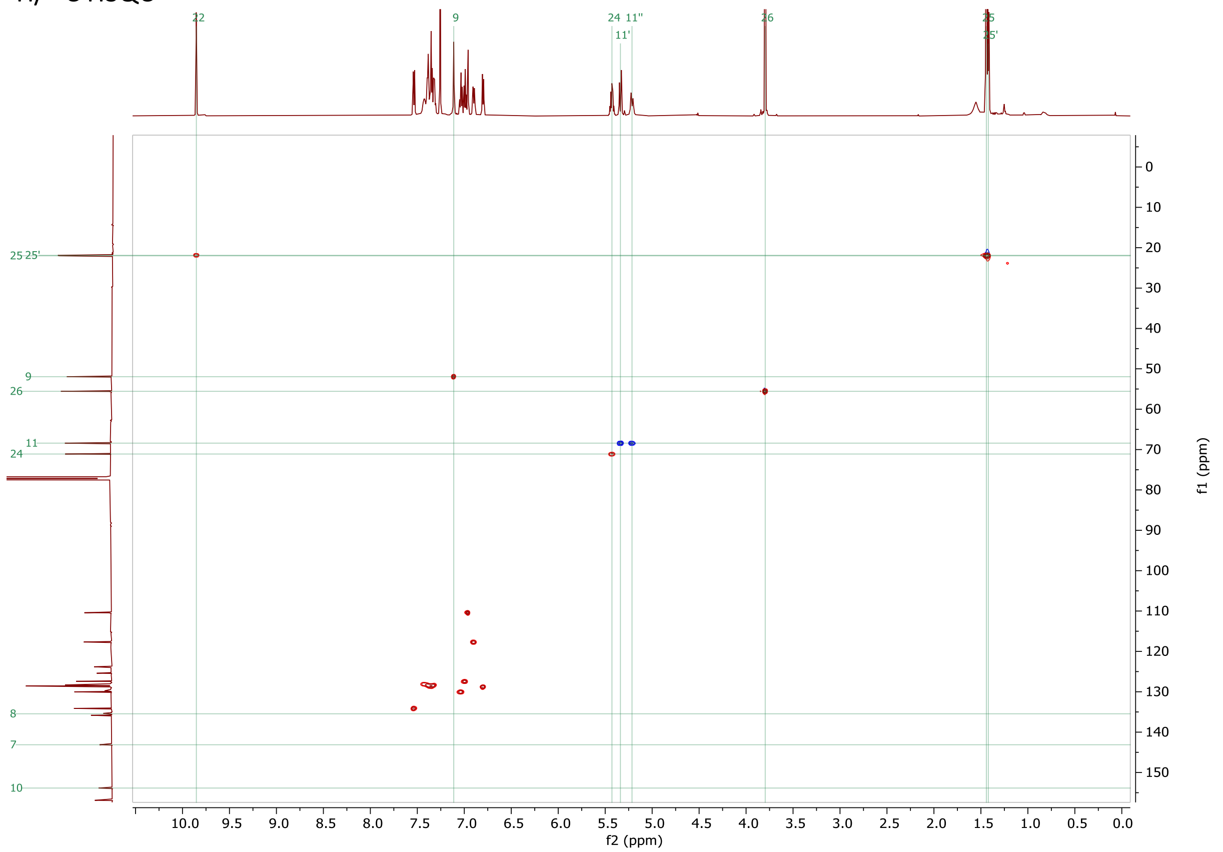

$^{1}H/^{13}C$ HMBC

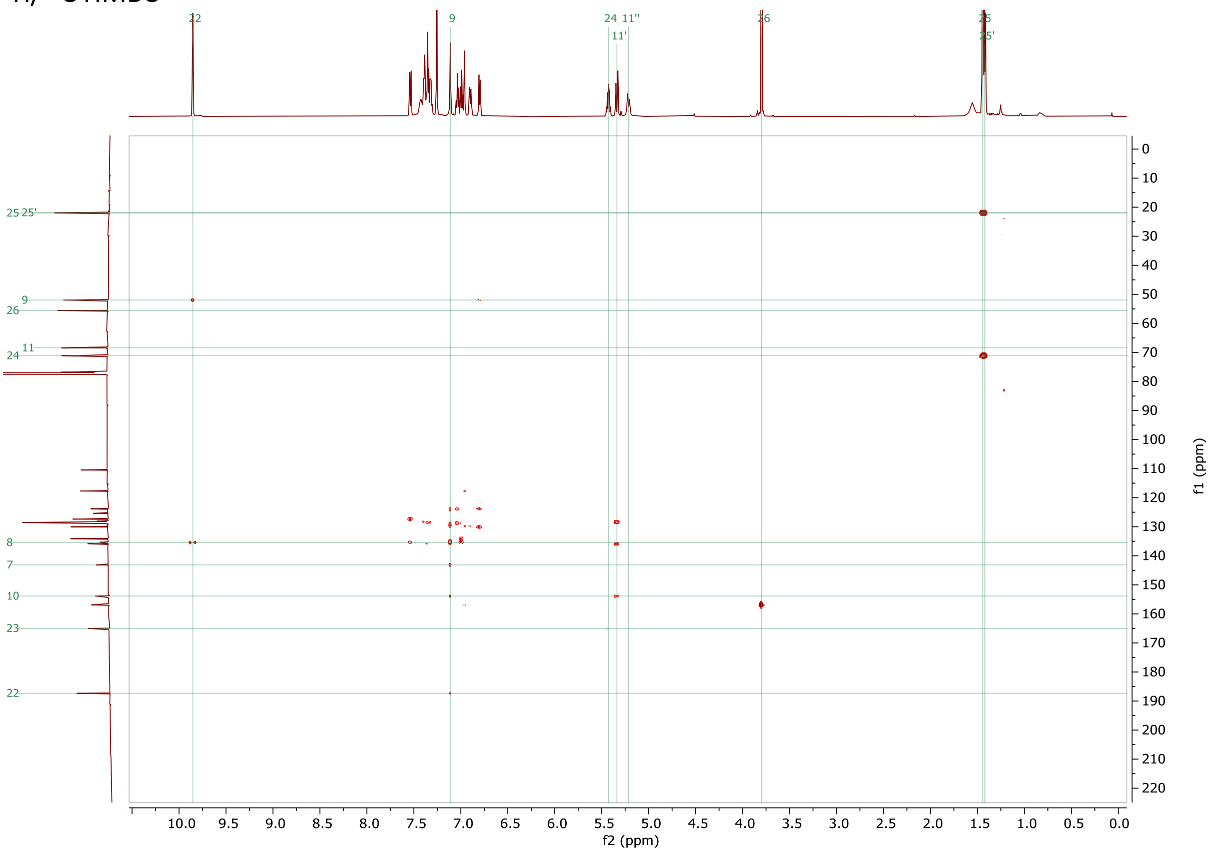

Benzyl (*R*)-4-(2-bromophenyl)-8-methoxy-1-oxo-1,4-dihydrofuro[3,4-c]quinoline-5(3*H*)-carboxylate 23

^{1}H NMR (500 MHz, CDCl$_3$)

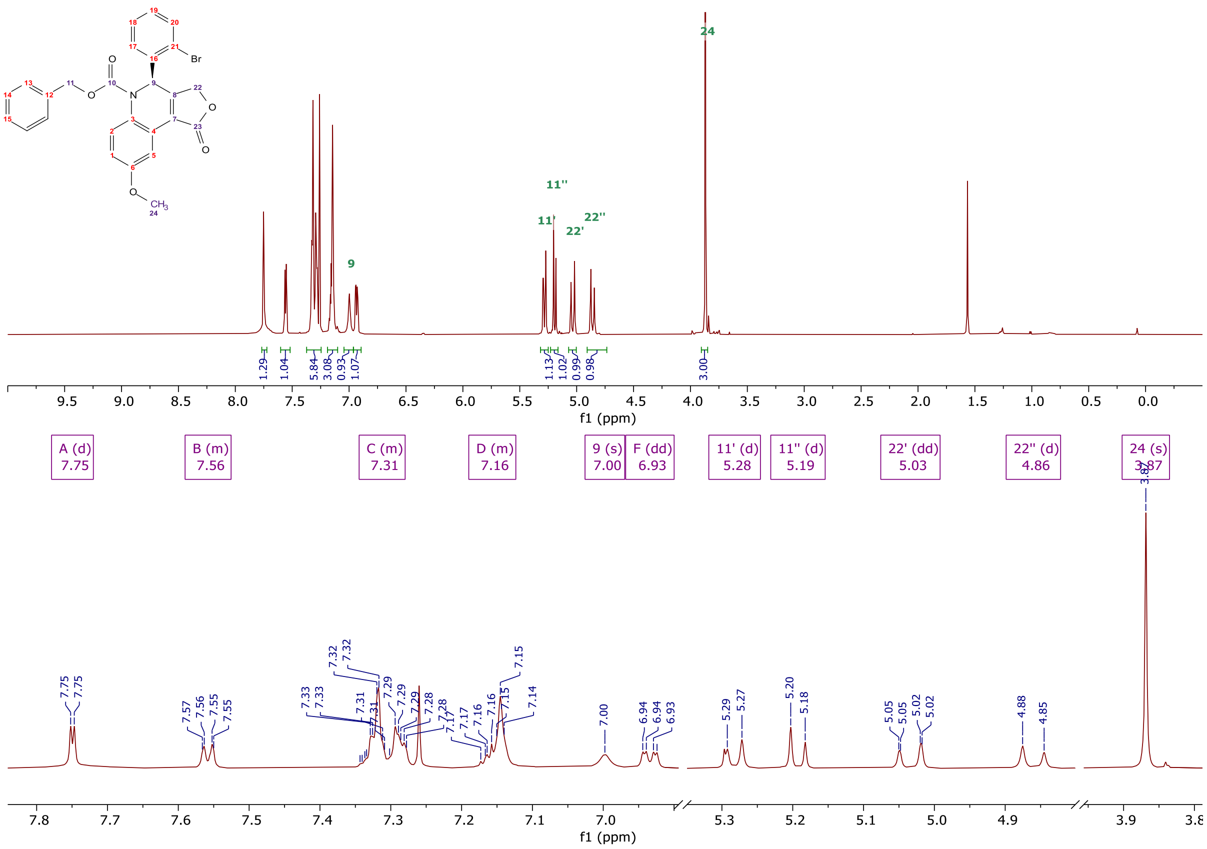

^{13}C NMR (126 MHz, CDCl$_3$)

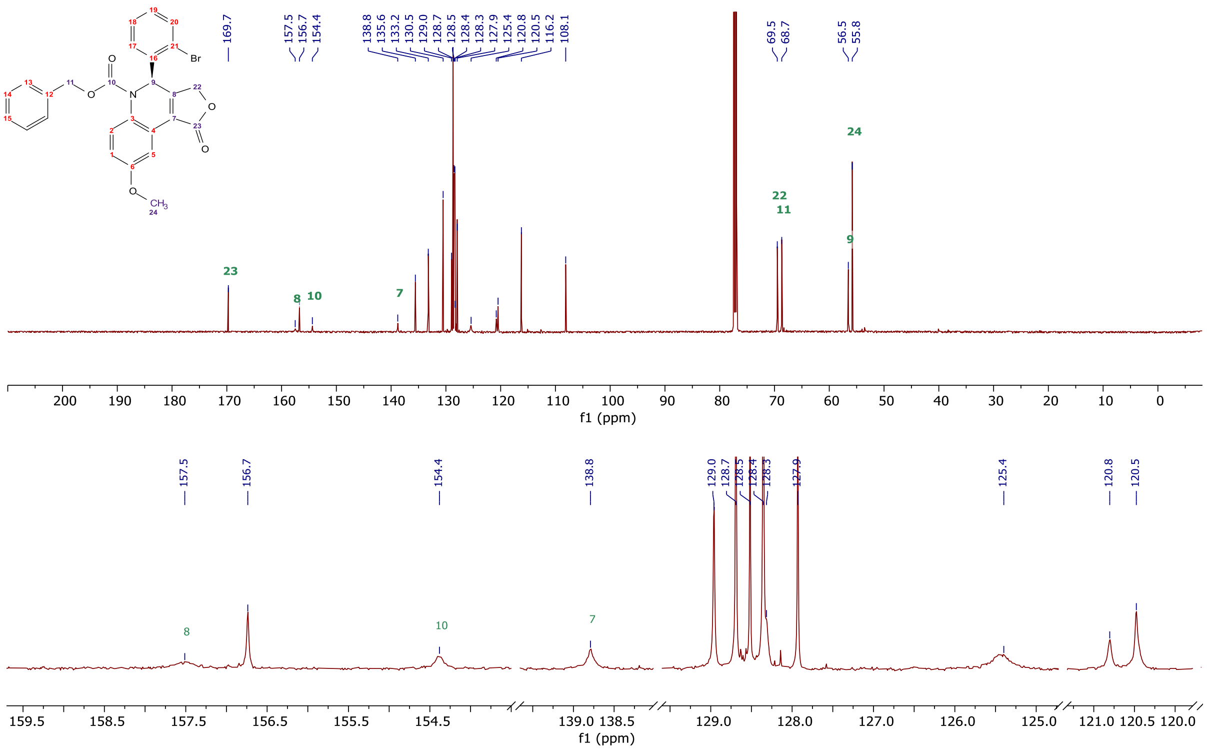

$^1H/^1H$ COSY

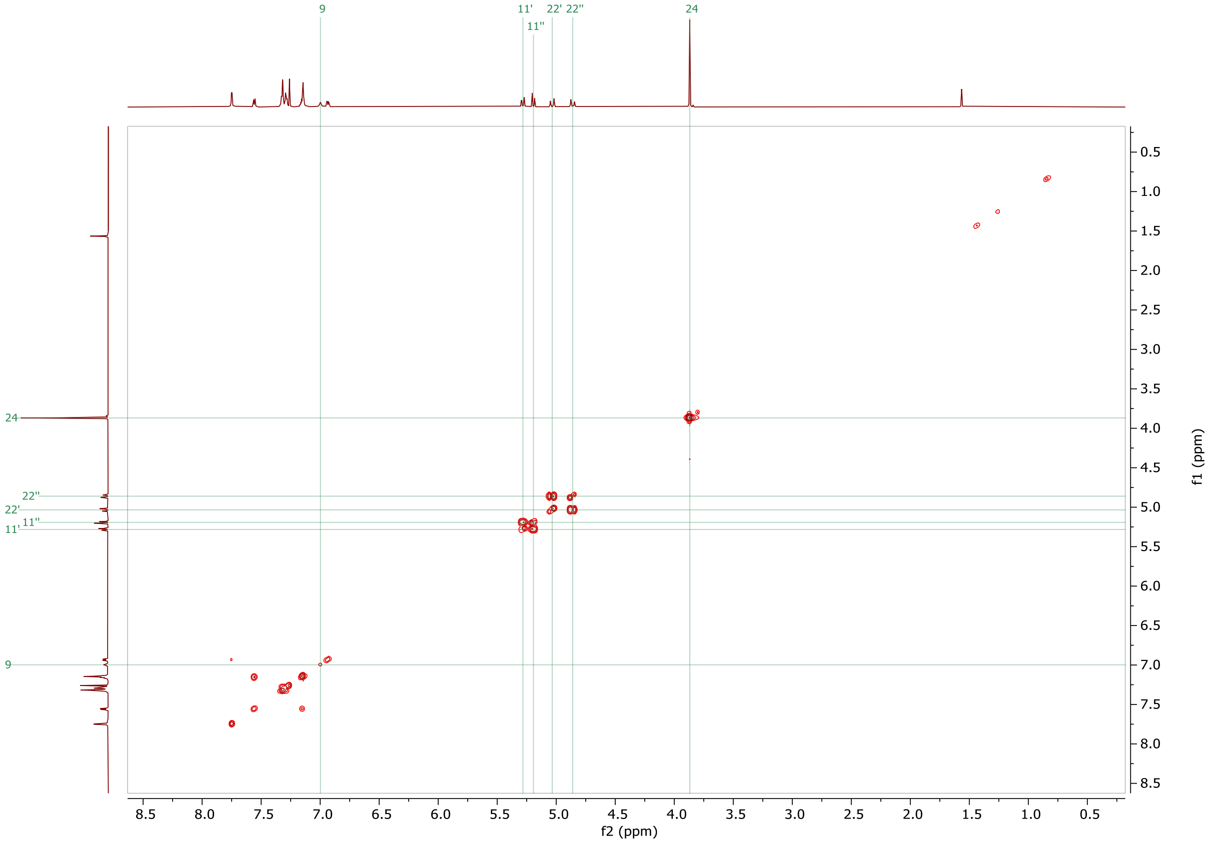

$^1H/^{13}C$ HSQC

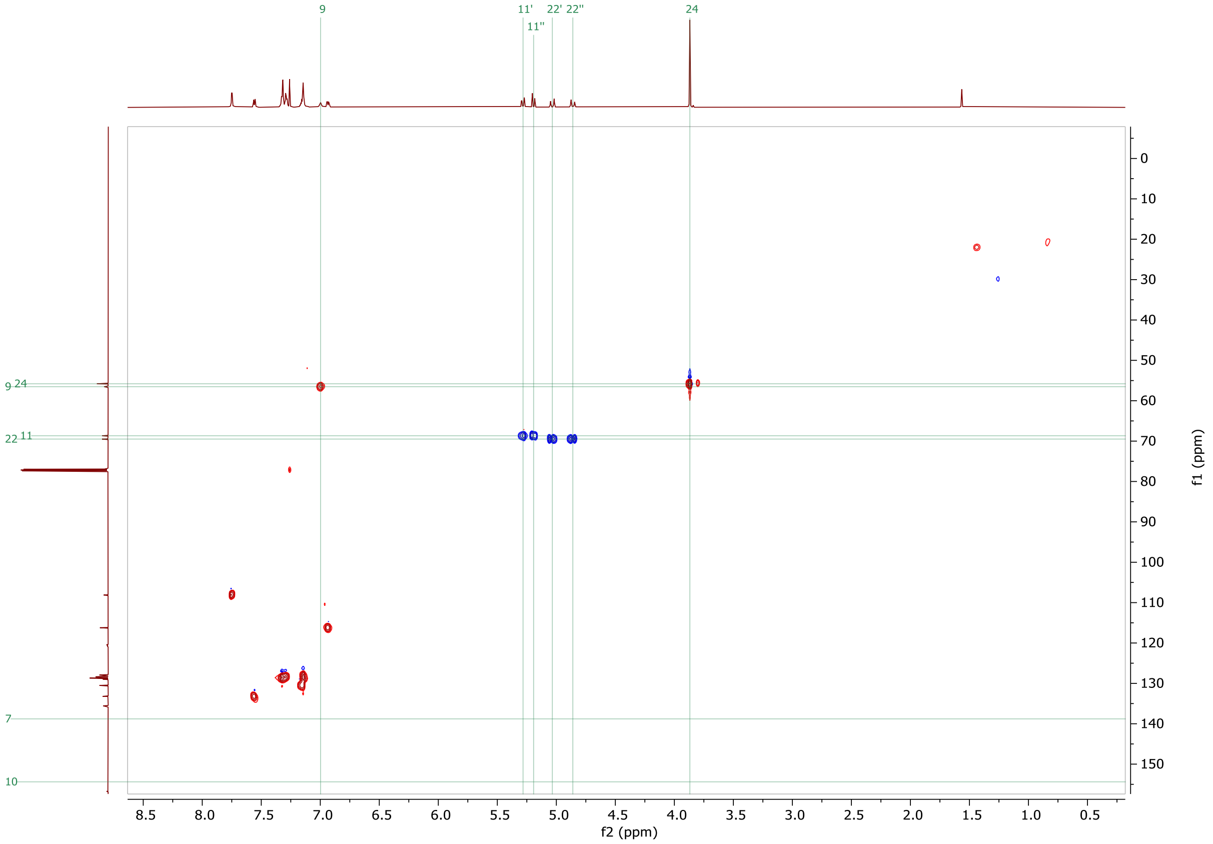

$^{1}H/^{13}C$ HMBC

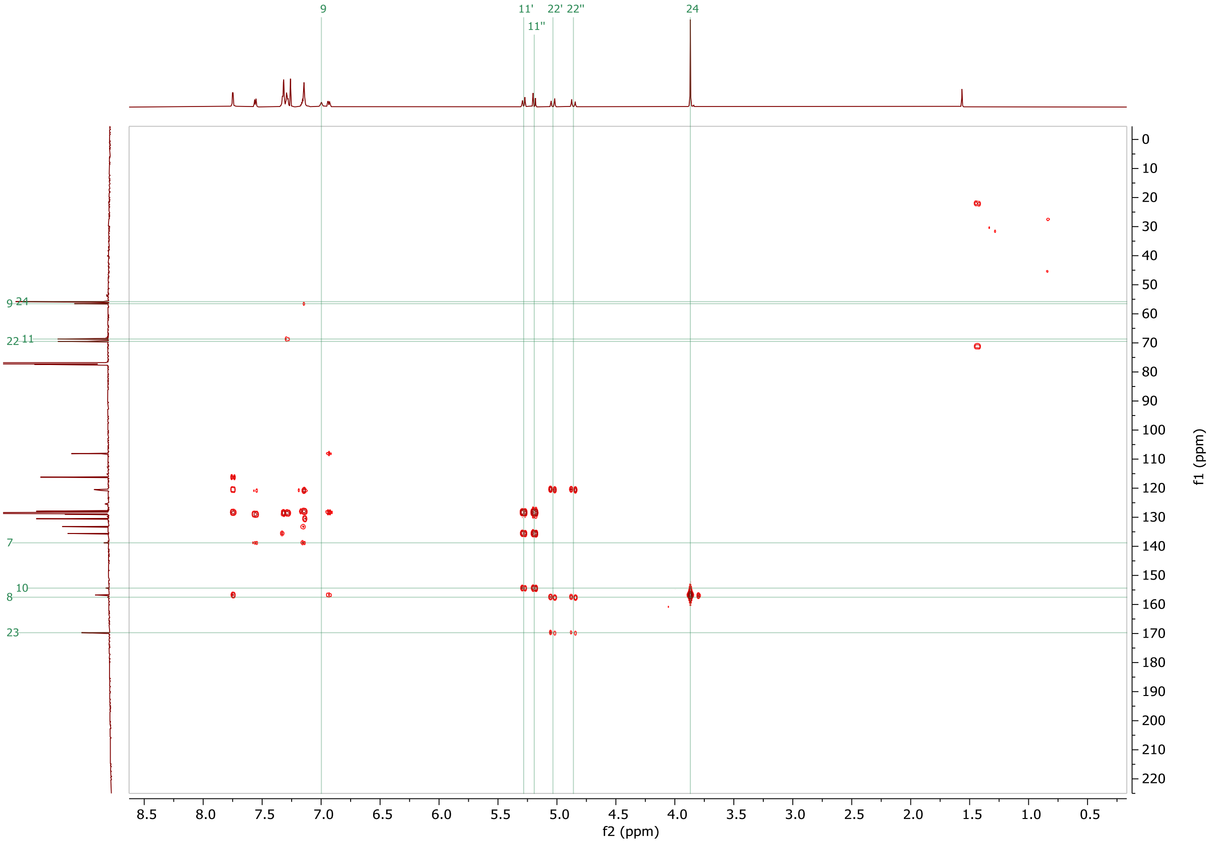

9
11'
11''
22'
22''
24
9
24
22
11
7
10
8
23
0
10
20
30
40
50
60
70
80
90
100
110
120
130
140
150
160
170
180
190
200
210
220
f1 (ppm)
8.5
8.0
7.5
7.0
6.5
6.0
5.5
5.0
4.5
4.0
3.5
3.0
2.5
2.0
1.5
1.0
0.5
f2 (ppm)

Benzyl (3a*R*,4*S*,9b*R*)-4-(2-bromophenyl)-3a,9b-dihydroxy-8-methoxy-1-oxo-1,3a,4,9b-tetrahydrofuro[3,4-c]quinoline-5(3*H*)-carboxylate 24

^{1}H NMR (500 MHz, $CDCl_3$)

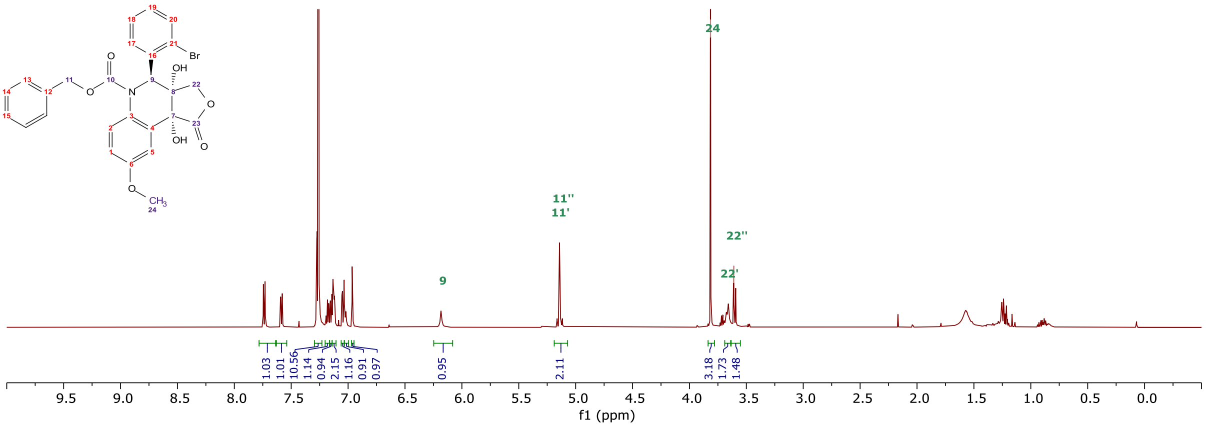

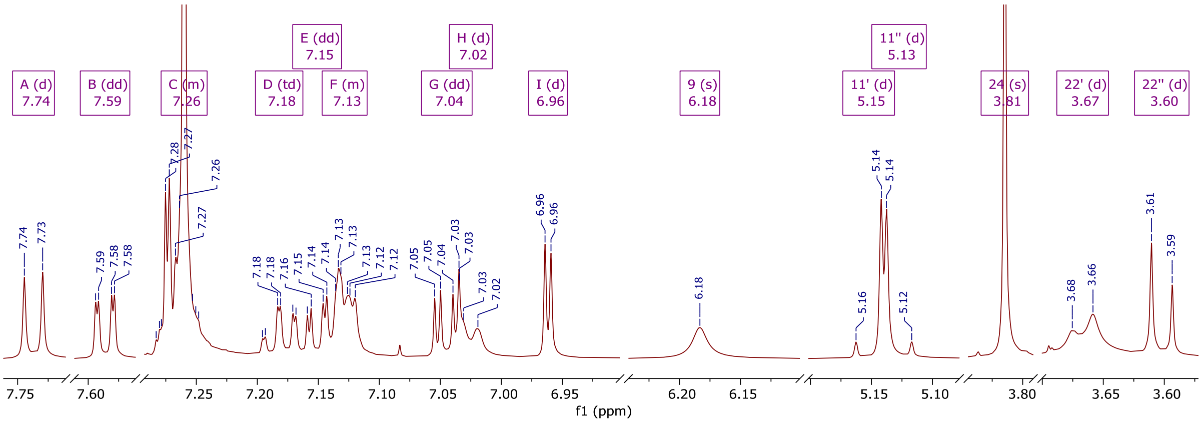

^{13}C NMR (126 MHz, $CDCl_3$)

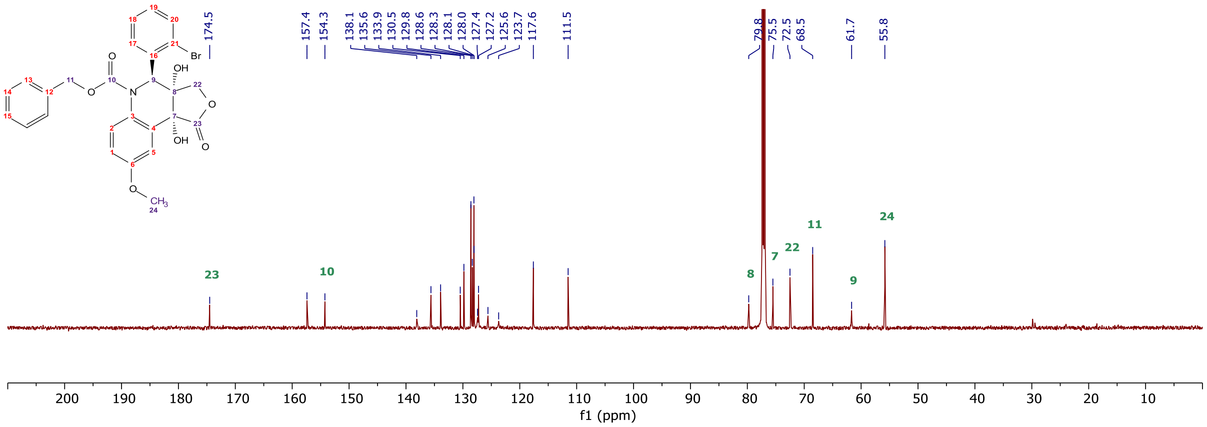

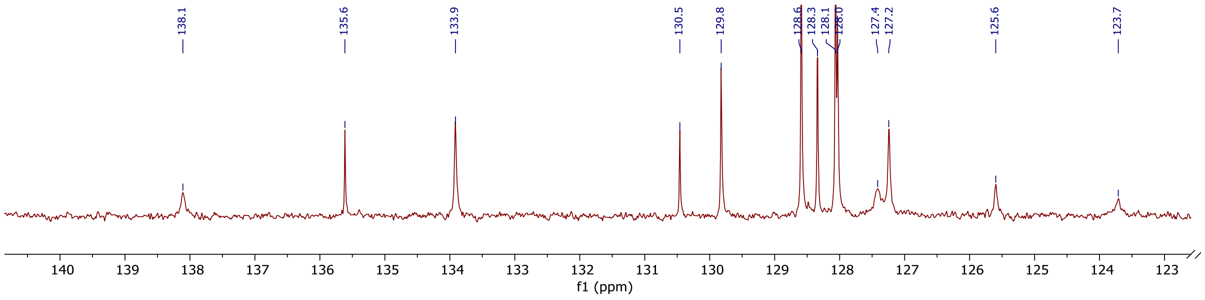

$^{1}H/^{1}H$ COSY

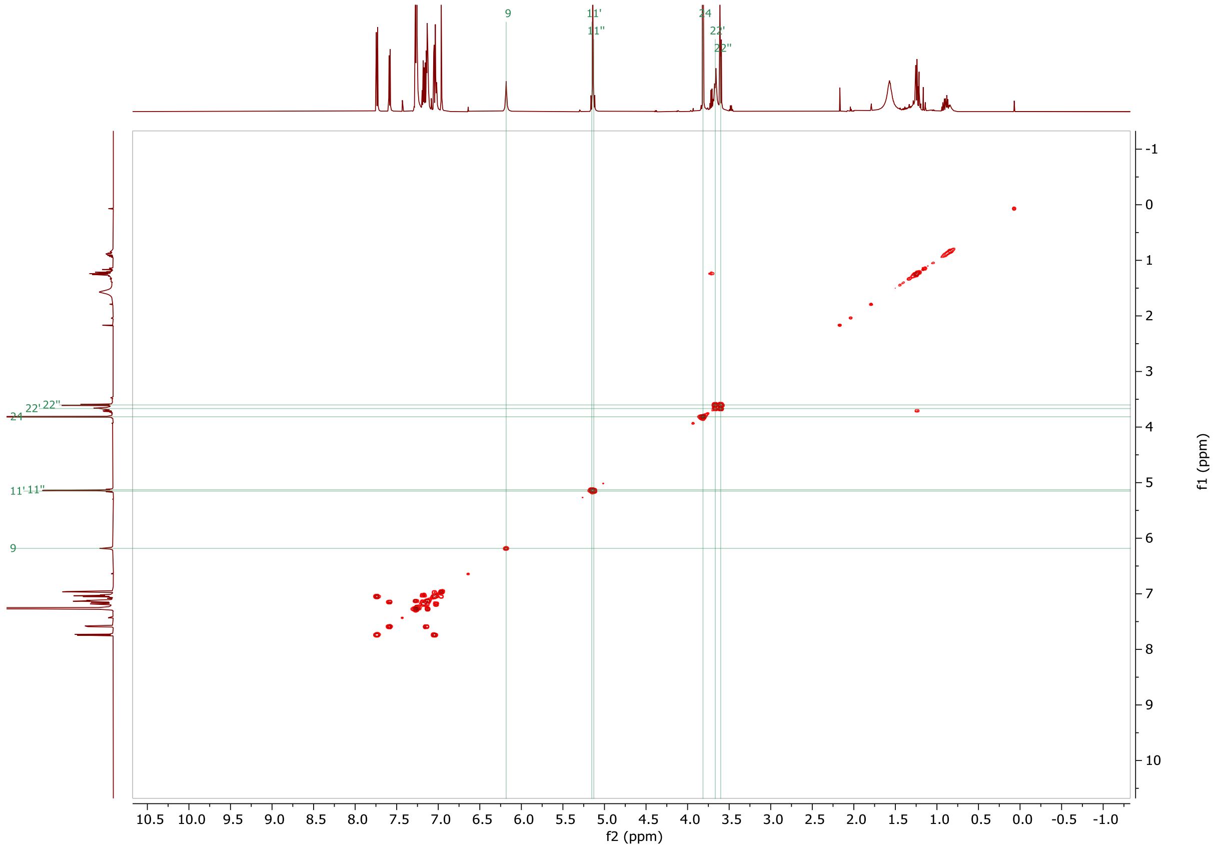

$^{1}H/^{13}C$ HSQC

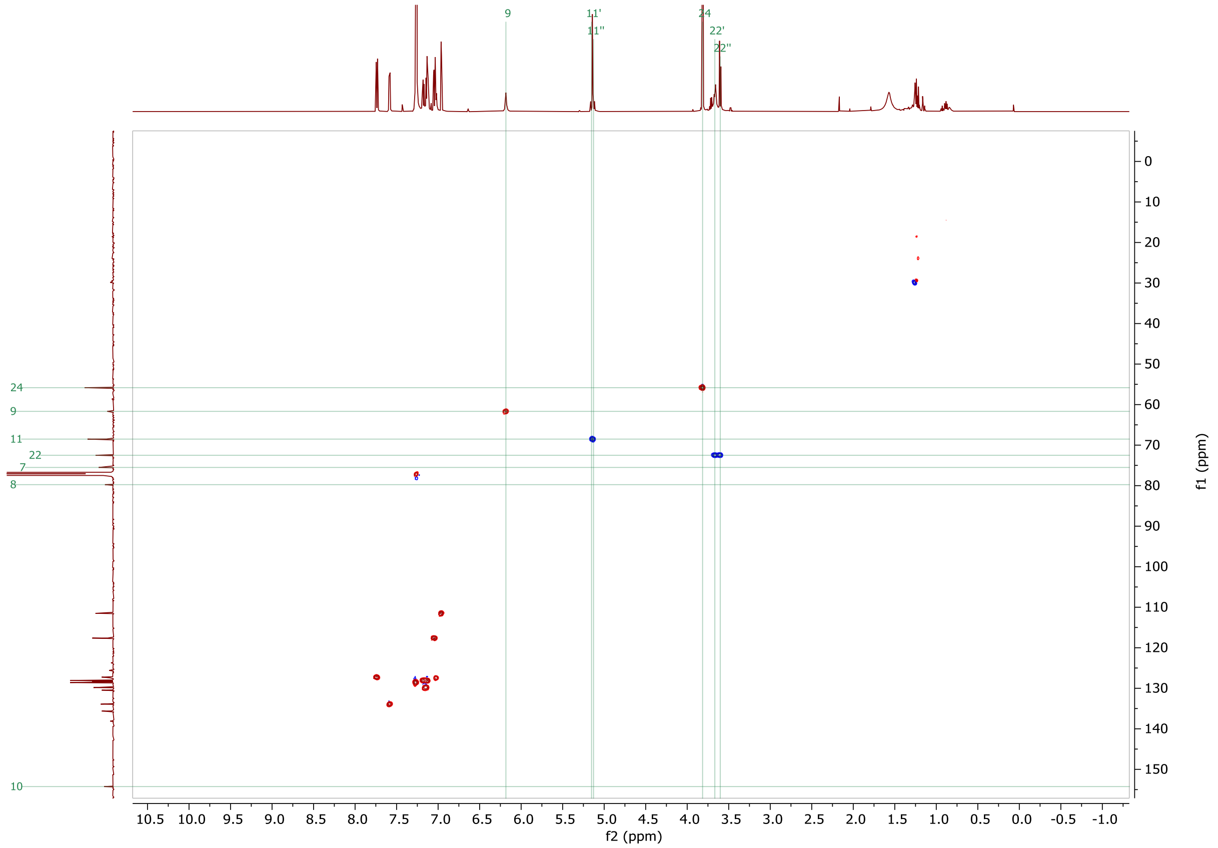

$^{1}H/^{13}C$ HMBC

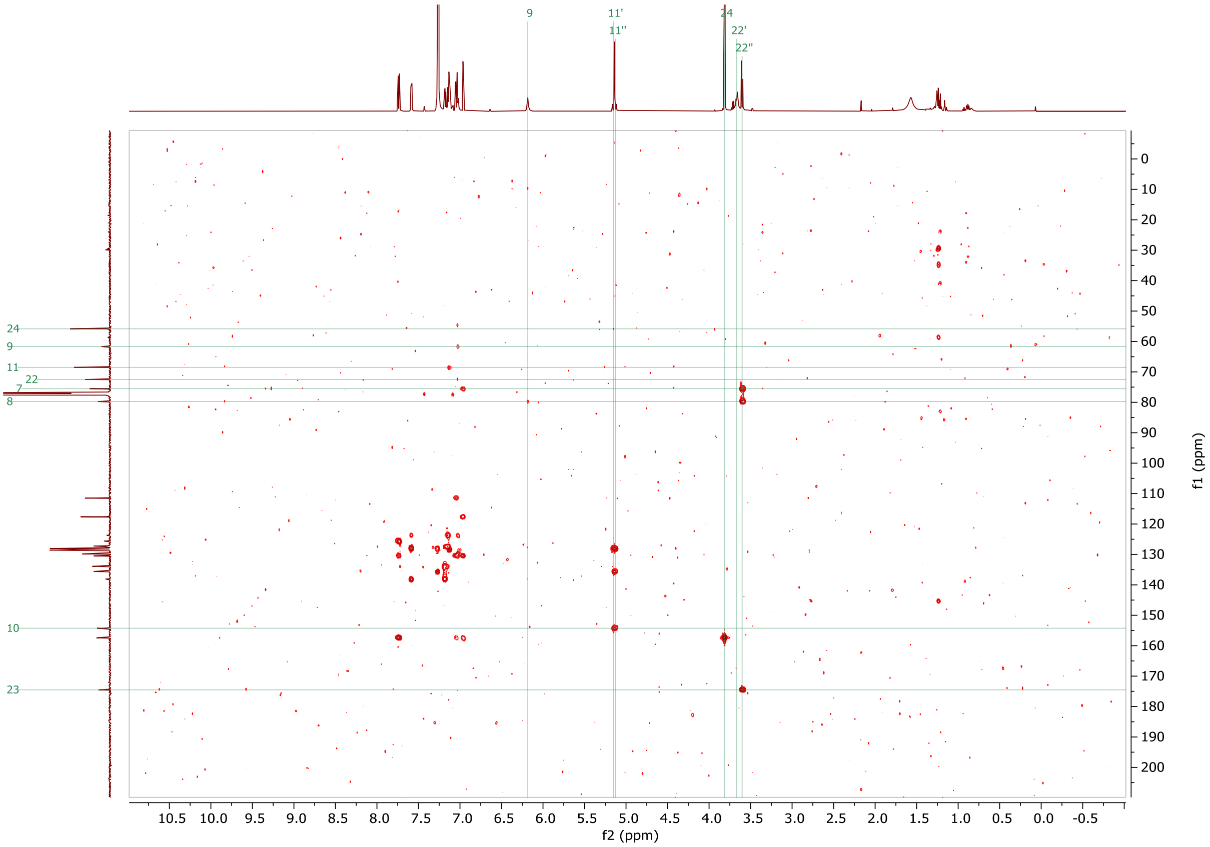

Benzyl (3a*R*,4*S*,9b*R*)-4-(2-bromophenyl)-8-methoxy-3a,9b-bis((4-methoxybenzyl)oxy)-1-oxo-1,3a,4,9b-tetrahydrofuro[3,4-c]quinoline-5(3*H*)-carboxylate 25

^{1}H NMR (500 MHz, $CDCl_3$)

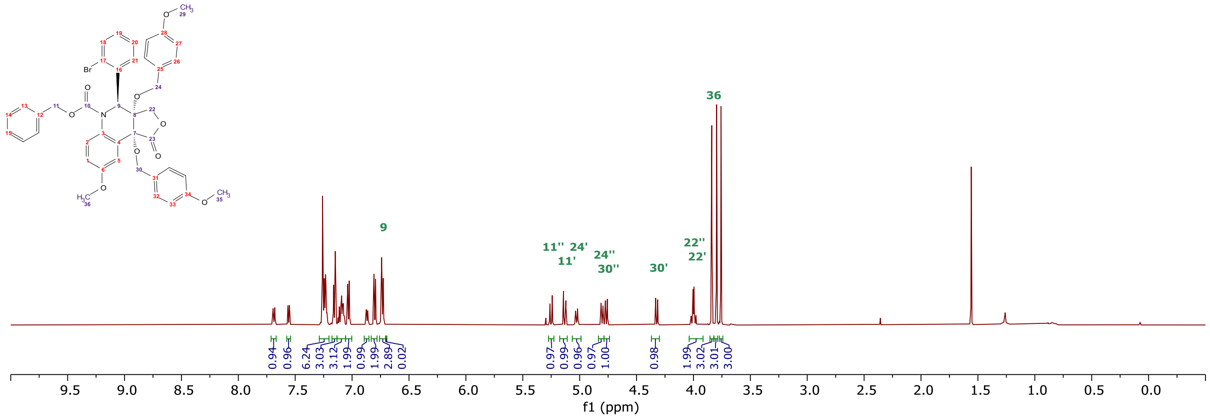

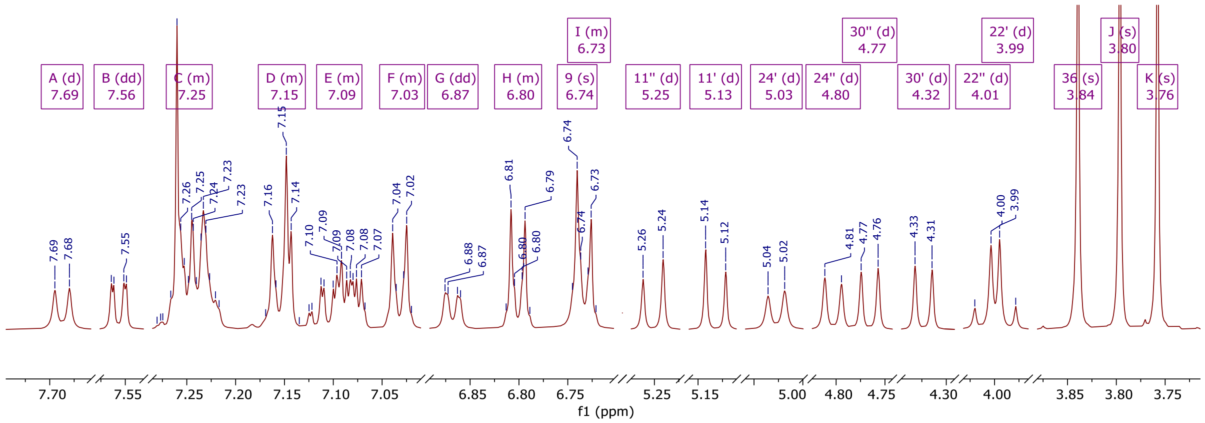

^{13}C NMR (126 MHz, $CDCl_3$)

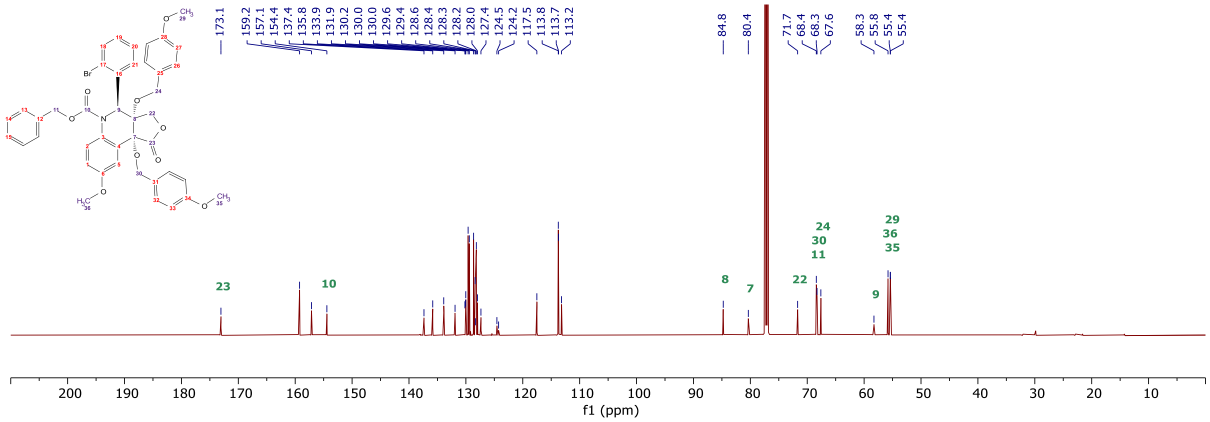

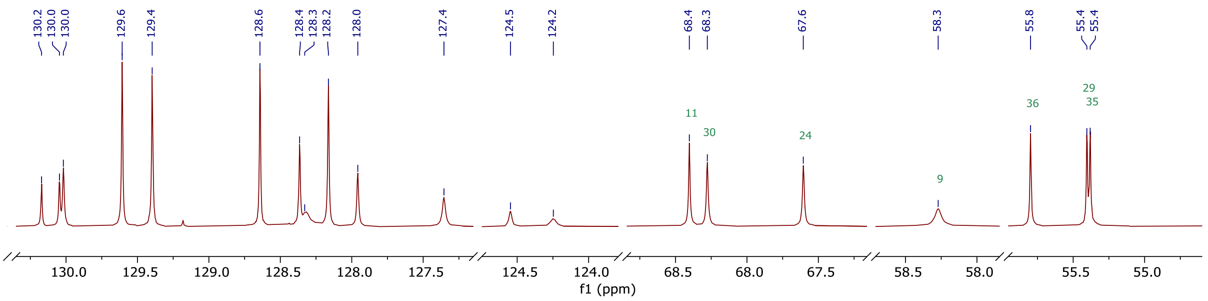

$^1H/^1H$ COSY

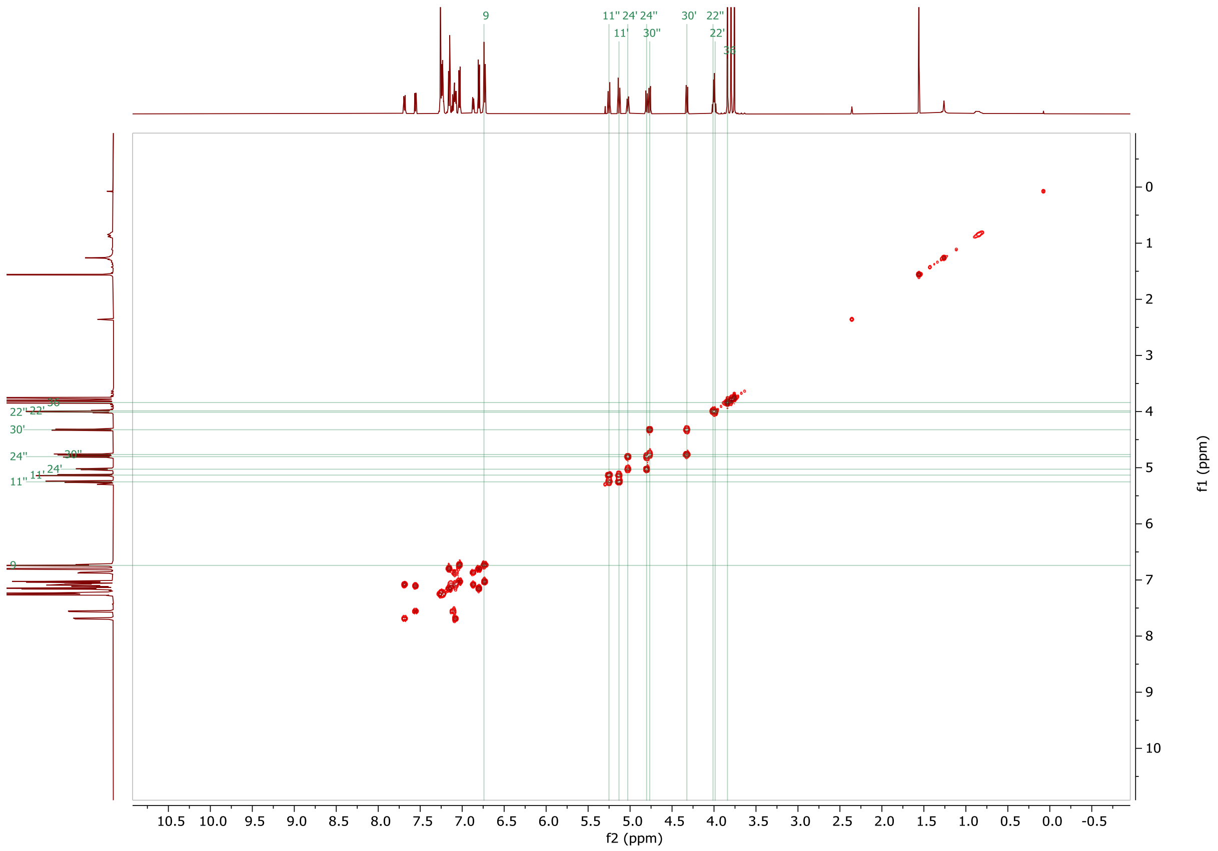

$^1H/^{13}C$ HSQC

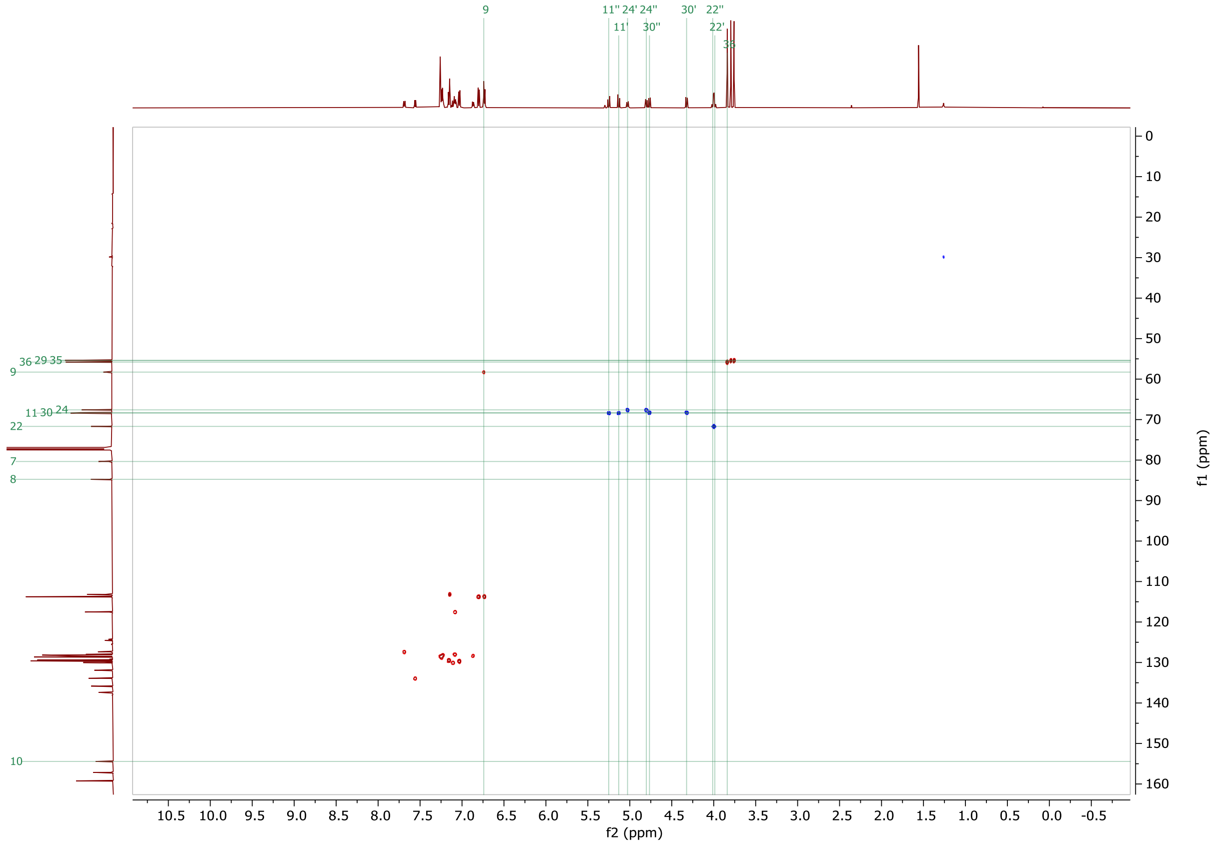

$^{1}H/^{13}C$ HMBC

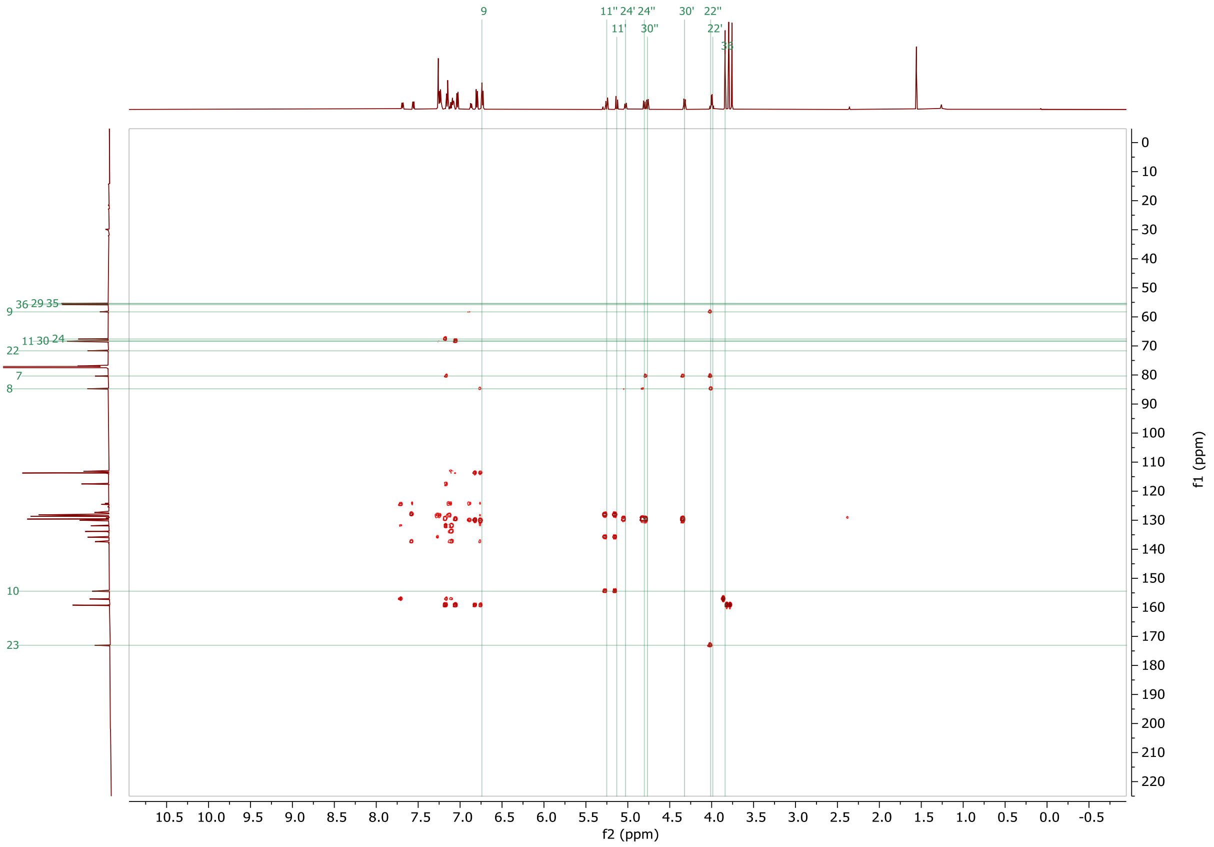

9
11'' 24' 24''
11' 30''
30' 22''
22'
35
36 29 35
9
11 30 24
22
7
8
10
23
f1 (ppm)
f2 (ppm)

Benzyl (6*S*,12*R*,13*R*)-13-(hydroxymethyl)-2-methoxy-12,13-bis((4-methoxybenzyl)oxy)-11-oxo-11,12-dihydro-6,12-methanodibenzo[*b*,*f*]azocine-5(6*H*)-carboxylate 26

^{1}H NMR (500 MHz, DMSO-d_6, 378 K)

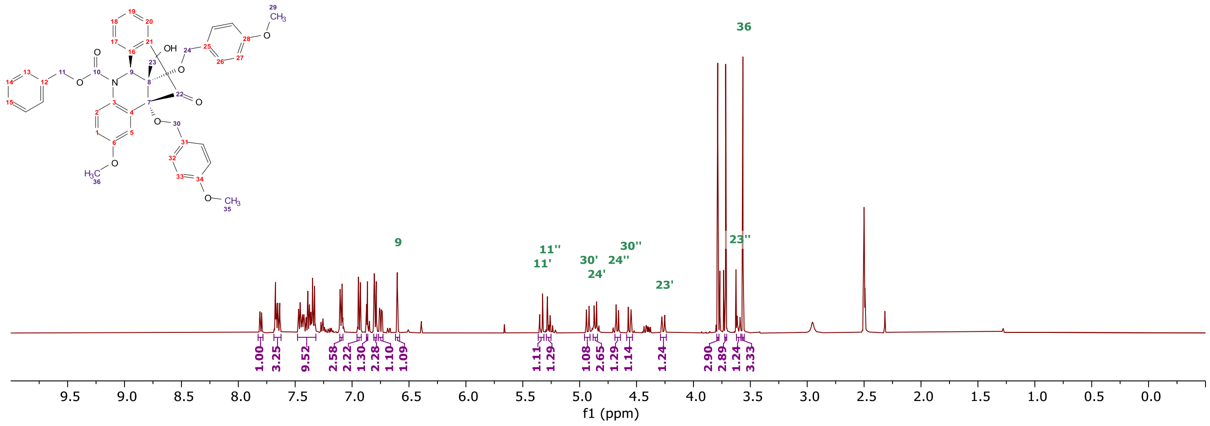

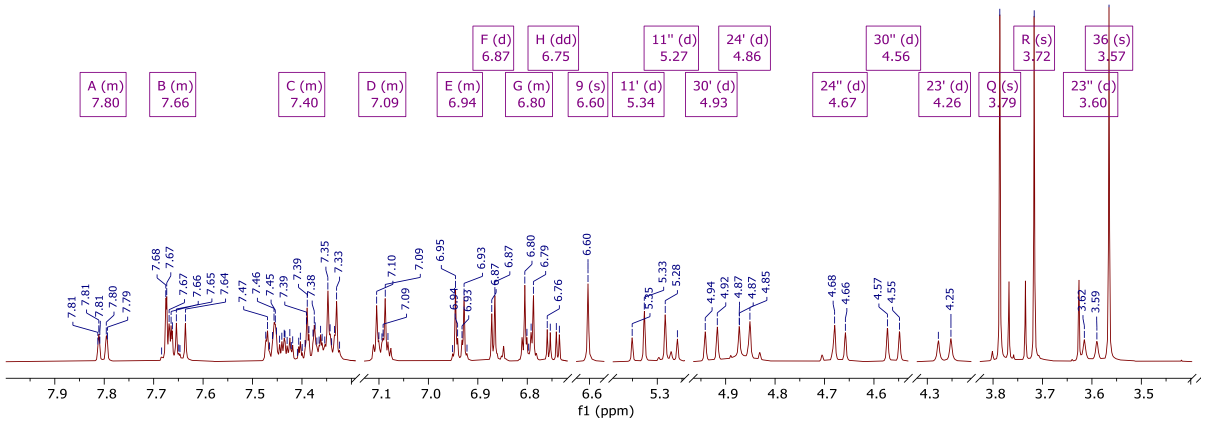

^{13}C NMR (126 MHz, DMSO-d_6, 378 K)

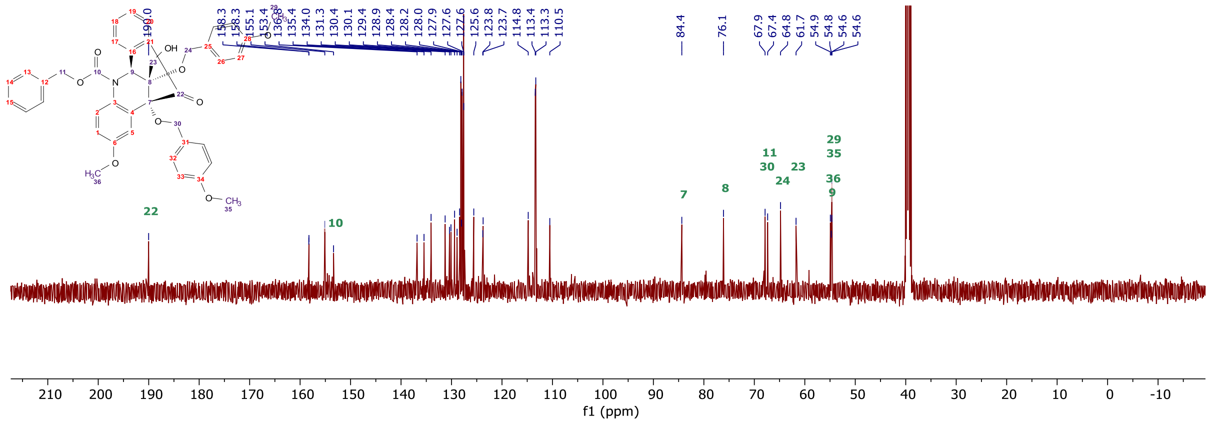

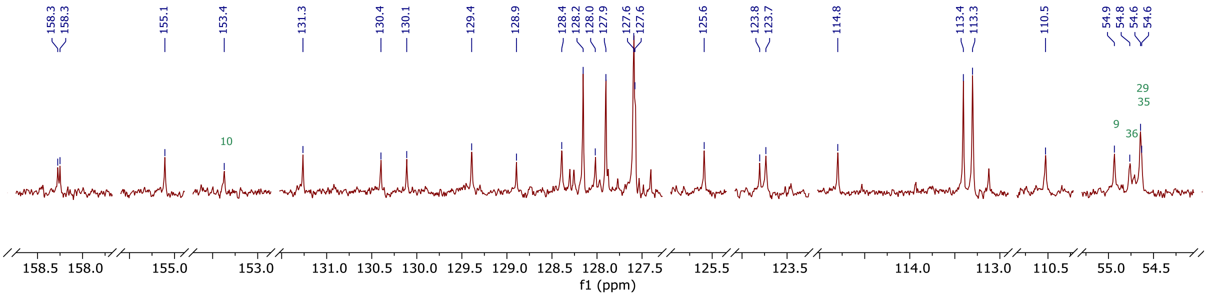

$^1H/^1H$ COSY (378 K)

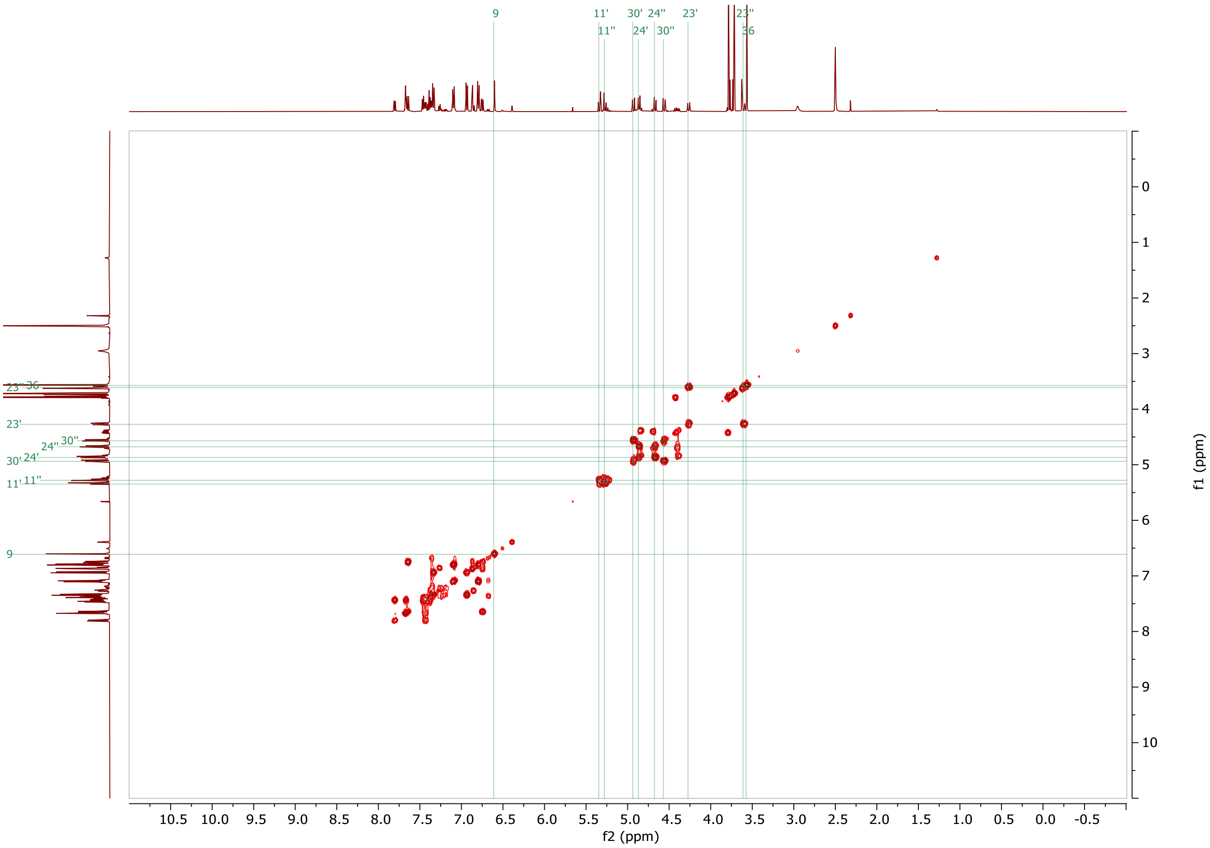

$^1H/^{13}C$ HSQC (378 K)

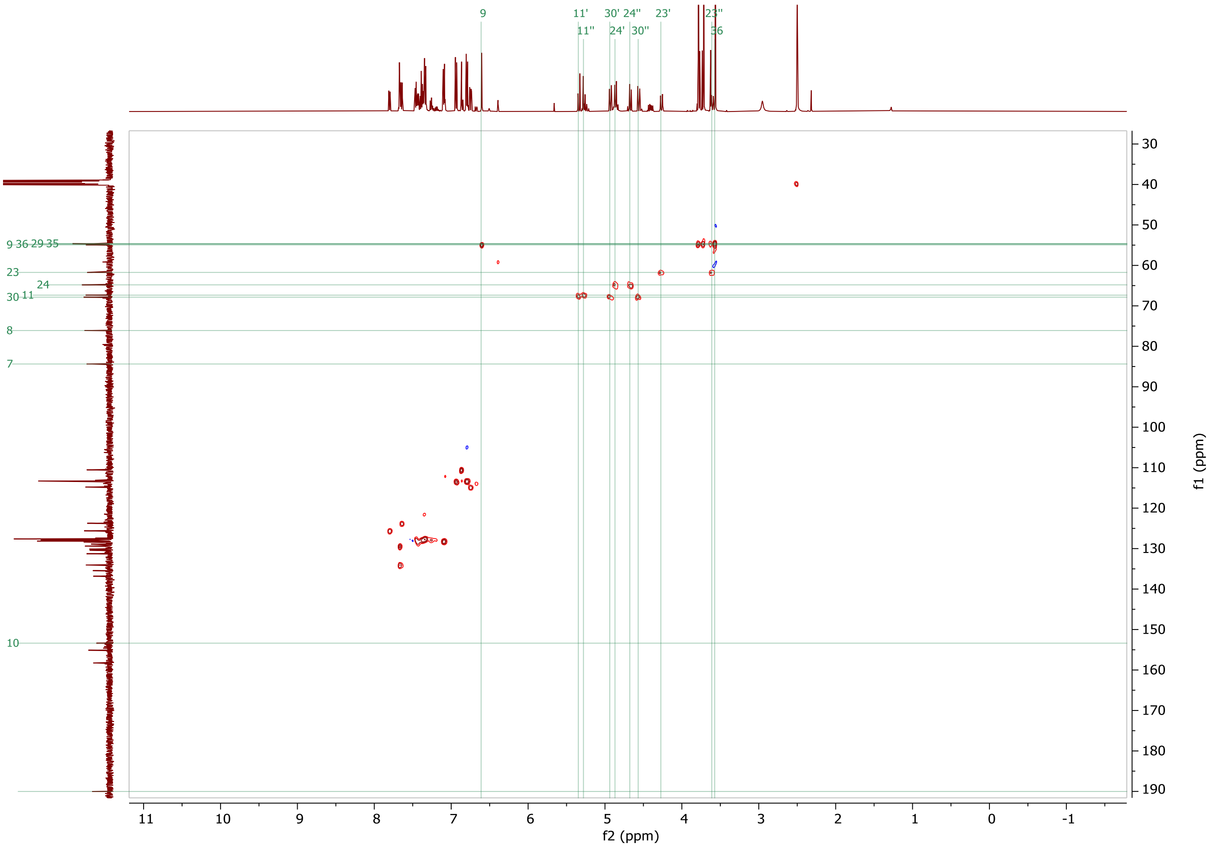

$^1H/^{13}C$ HMBC (378 K)

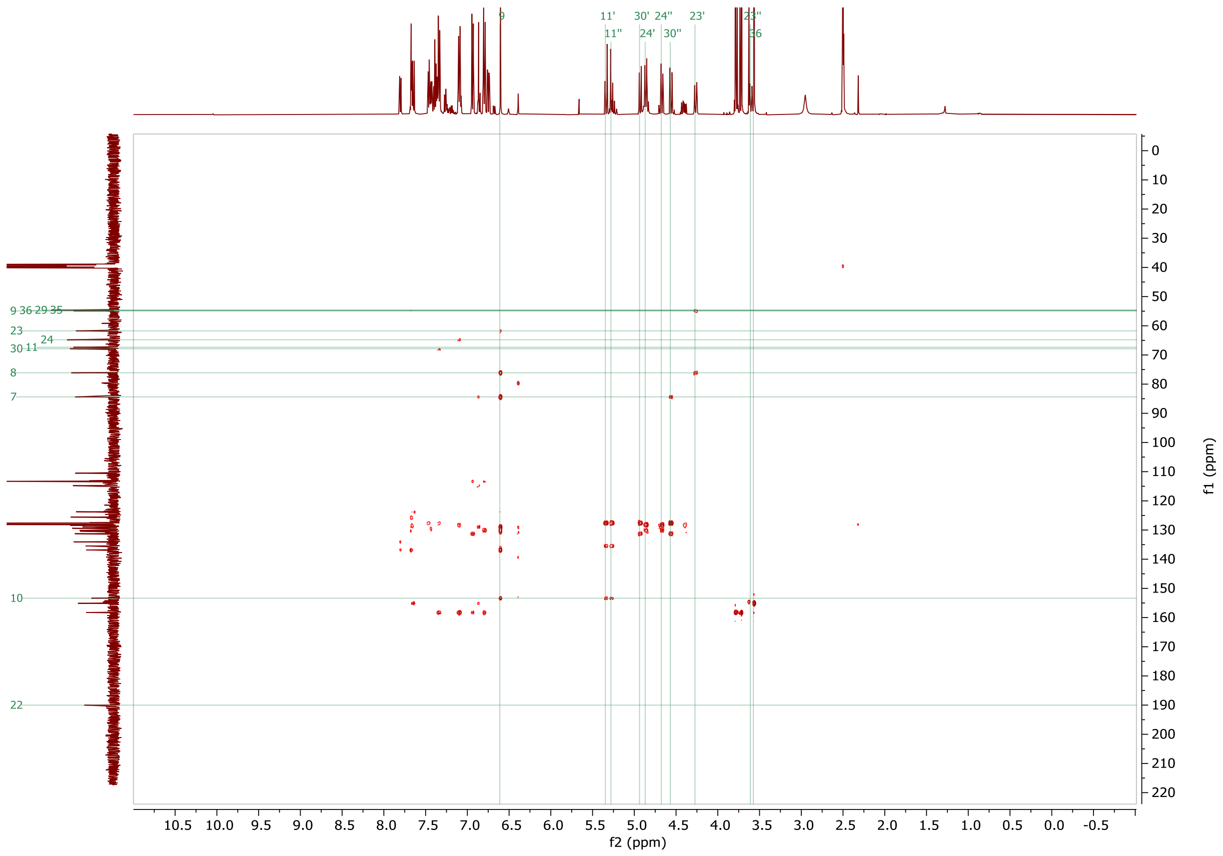

Benzyl (6S,12R,13S)-13-formyl-2-methoxy-12,13-bis((4-methoxybenzyl)oxy)-11-oxo-11,12-dihydro-6,12-methanodibenzo[*b*,*f*]azocine-5(6*H*)-carboxylate 27

^{1}H NMR (500 MHz, CDCl$_3$)

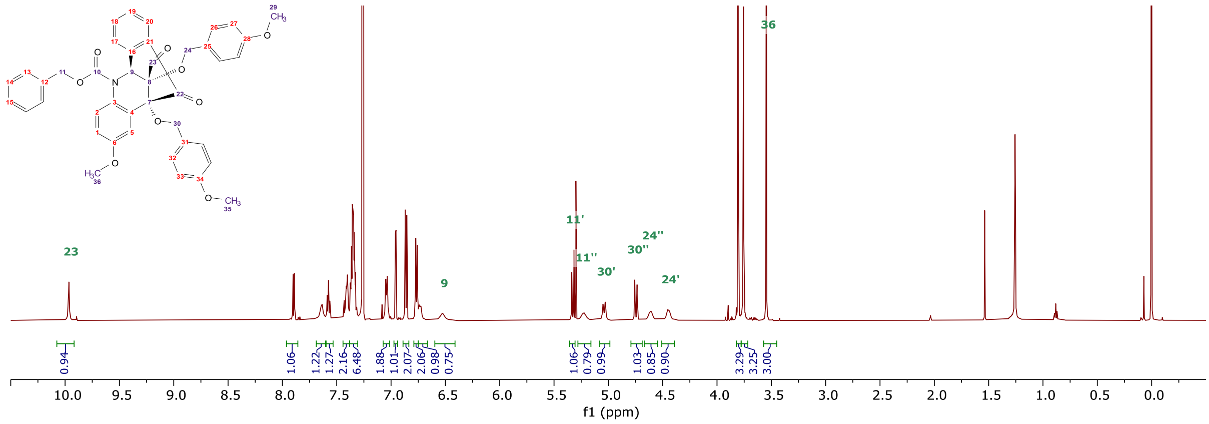

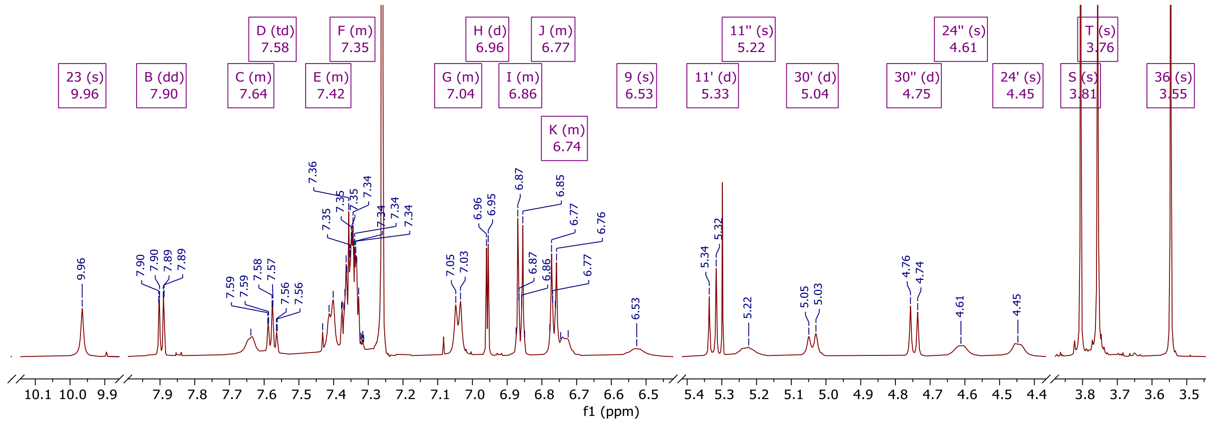

^{13}C NMR (126 MHz, CDCl$_3$)

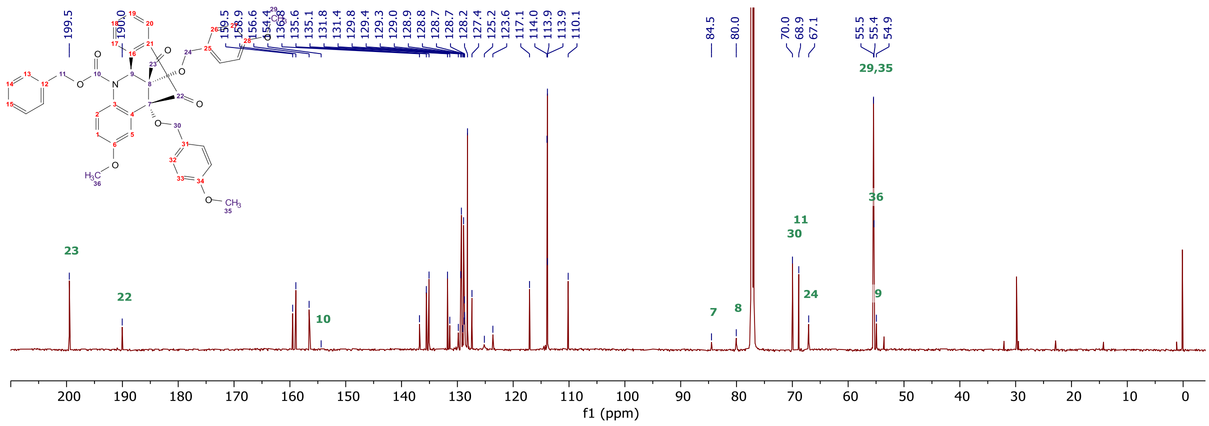

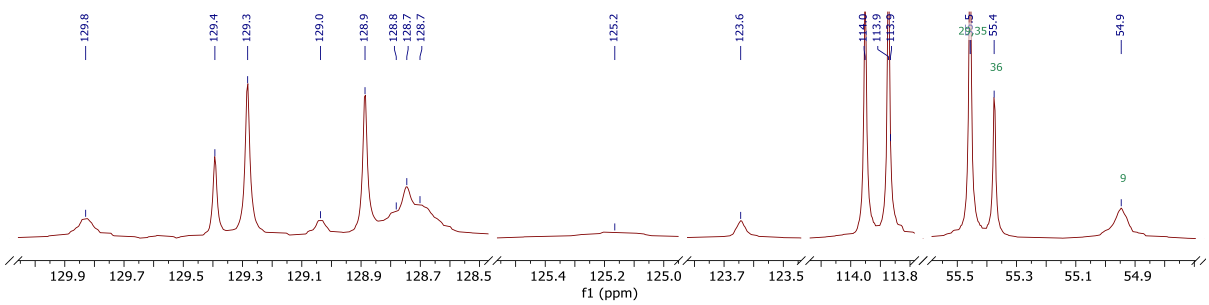

^{1}H/^{1}H COSY

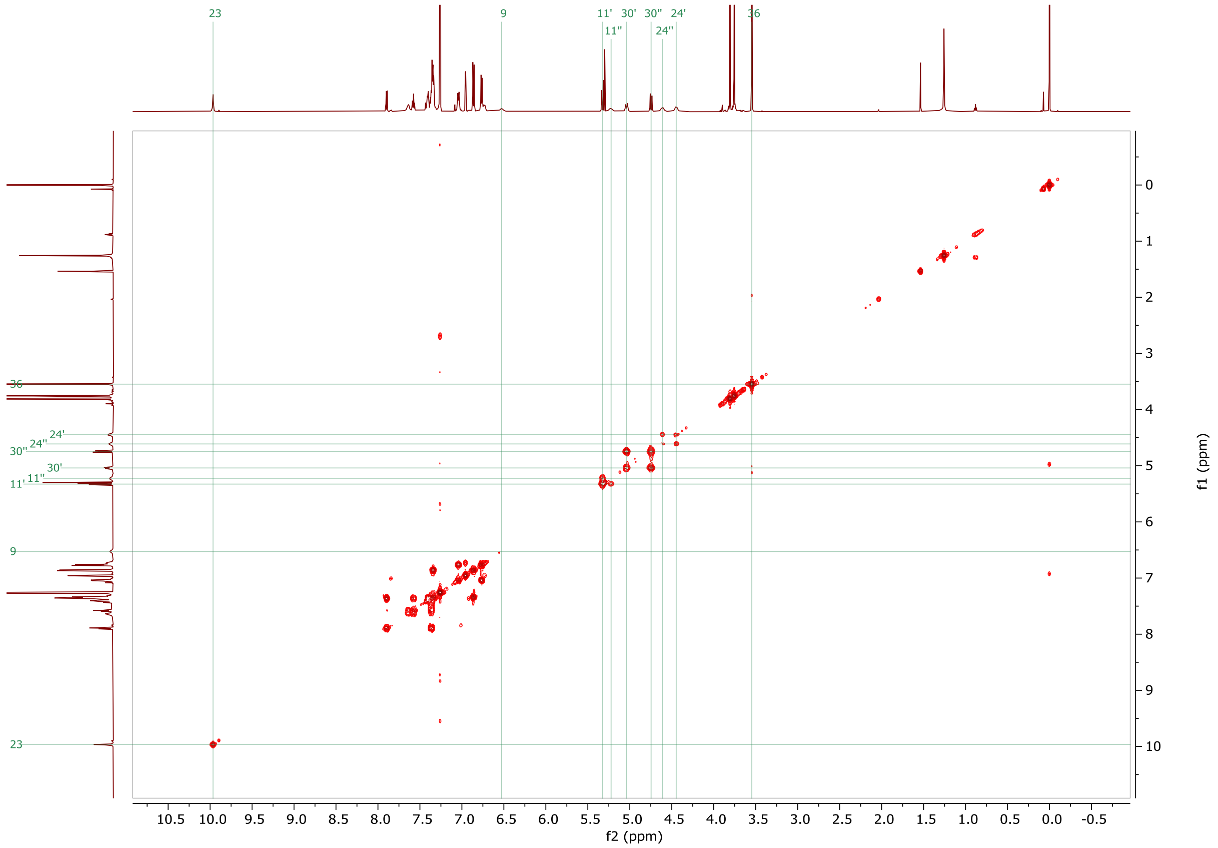

^{1}H/^{13}C HSQC

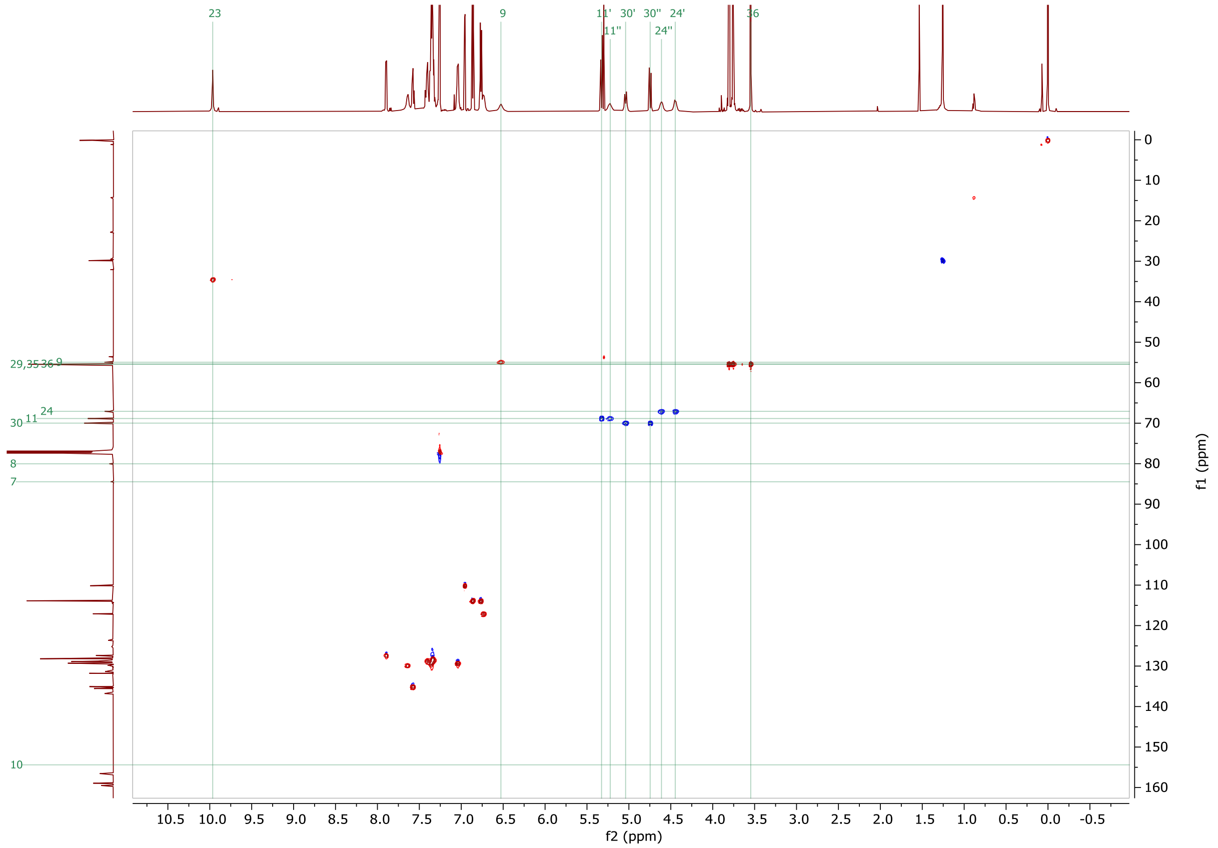

$^{1}H/^{13}C$ HMBC

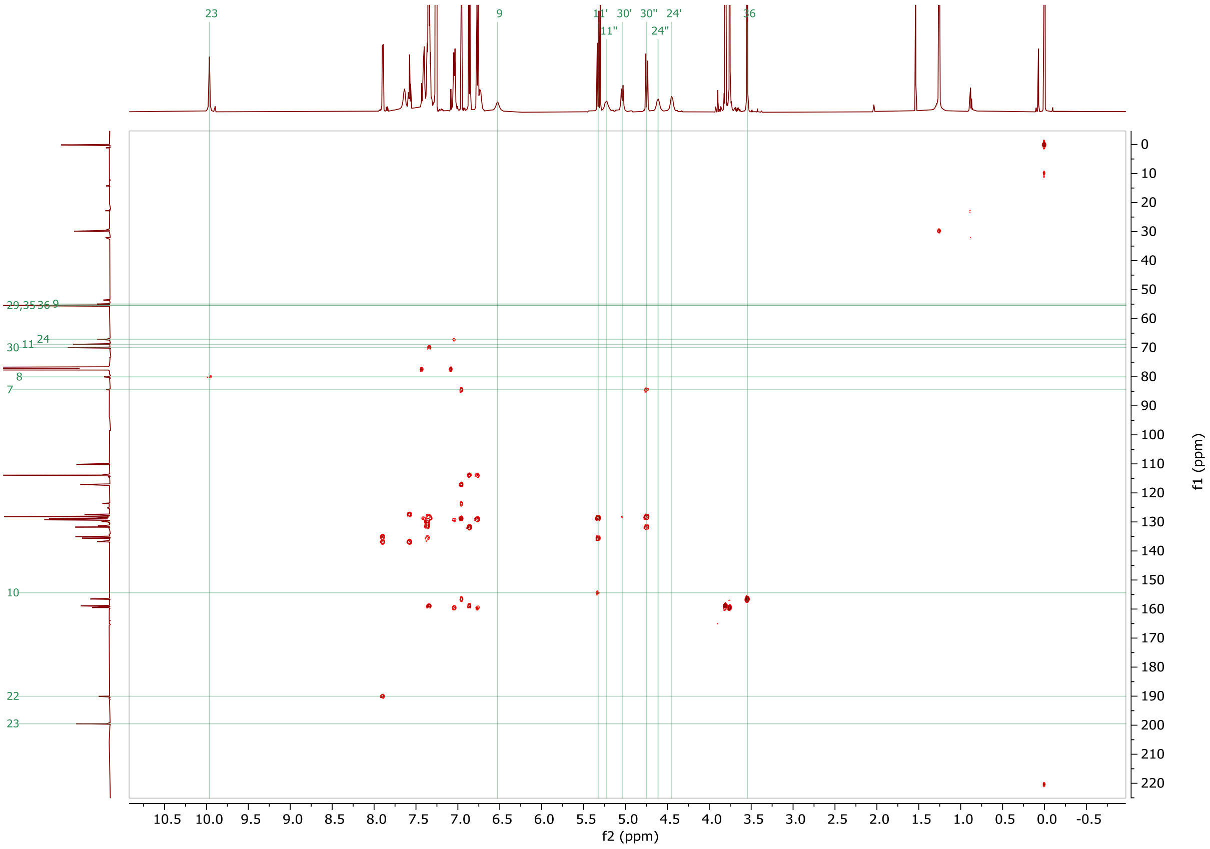

Benzyl (6*S*,12*R*,13*S*)-13-ethynyl-2-methoxy-12,13-bis((4-methoxybenzyl)oxy)-11-oxo-11,12-dihydro-6,12-methanodibenzo[*b*,*f*]azocine-5(6*H*)-carboxylate 28

^{1}H NMR (500 MHz, DMSO-d_6)

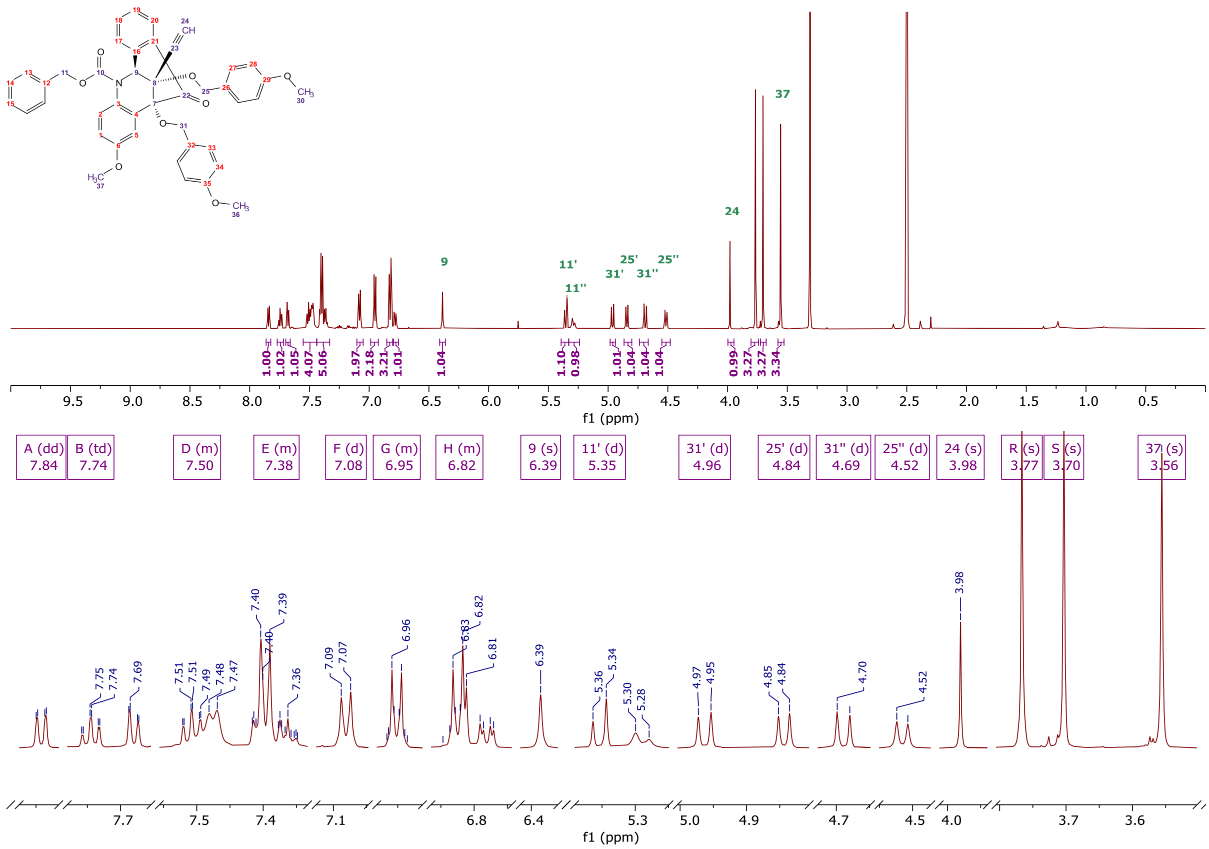

^{13}C NMR (126 MHz, DMSO-d_6)

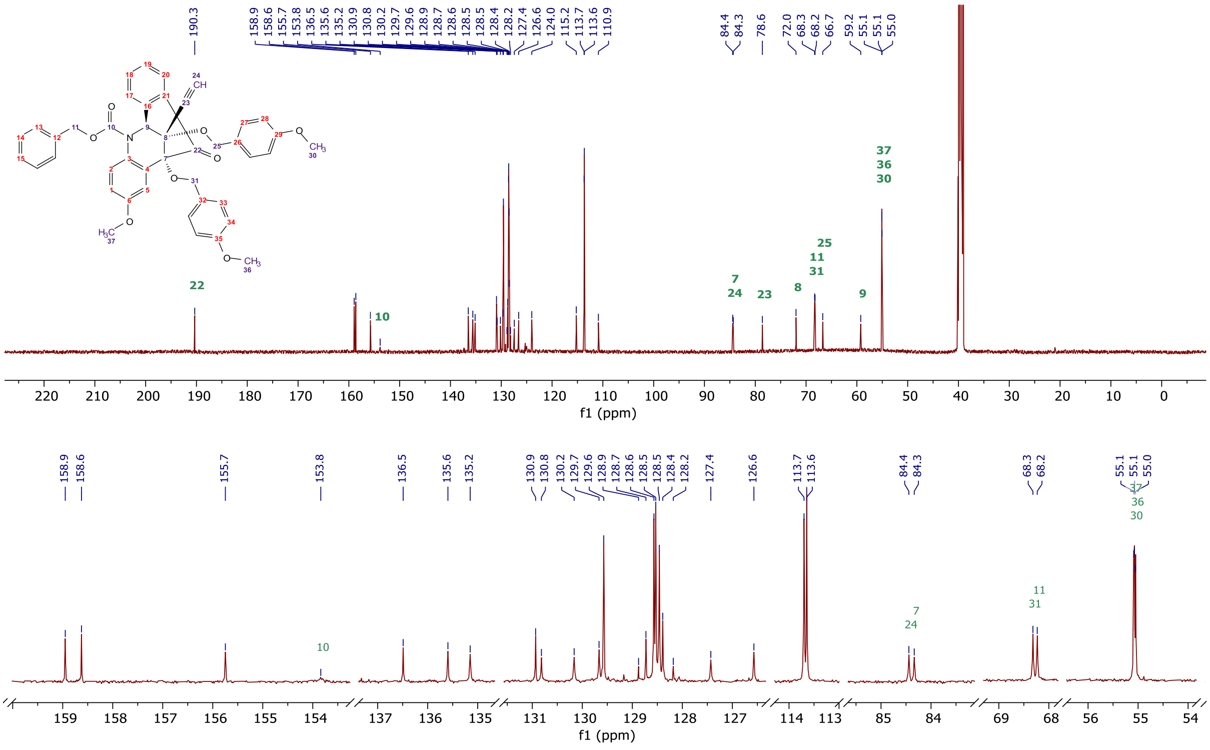

$^{1}H/^{1}H$ COSY

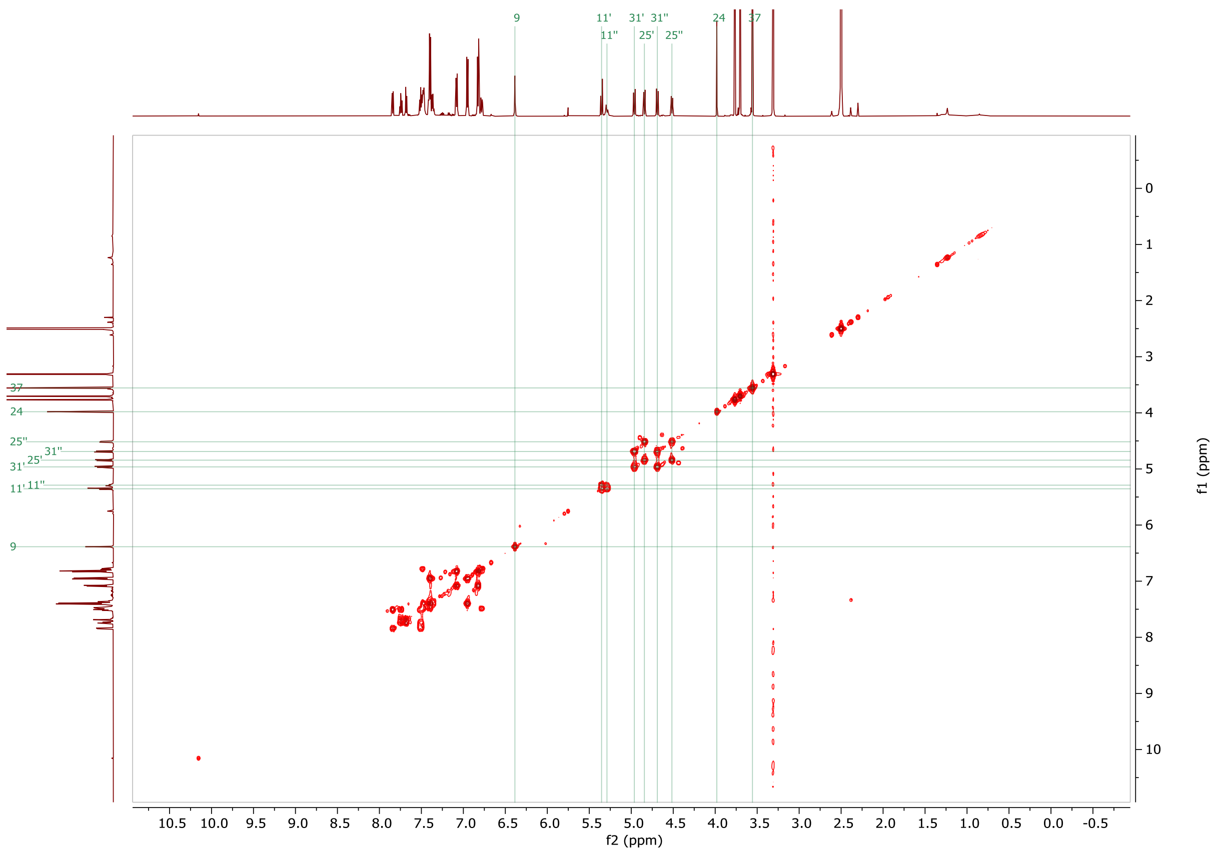

$^{1}H/^{13}C$ HSQC

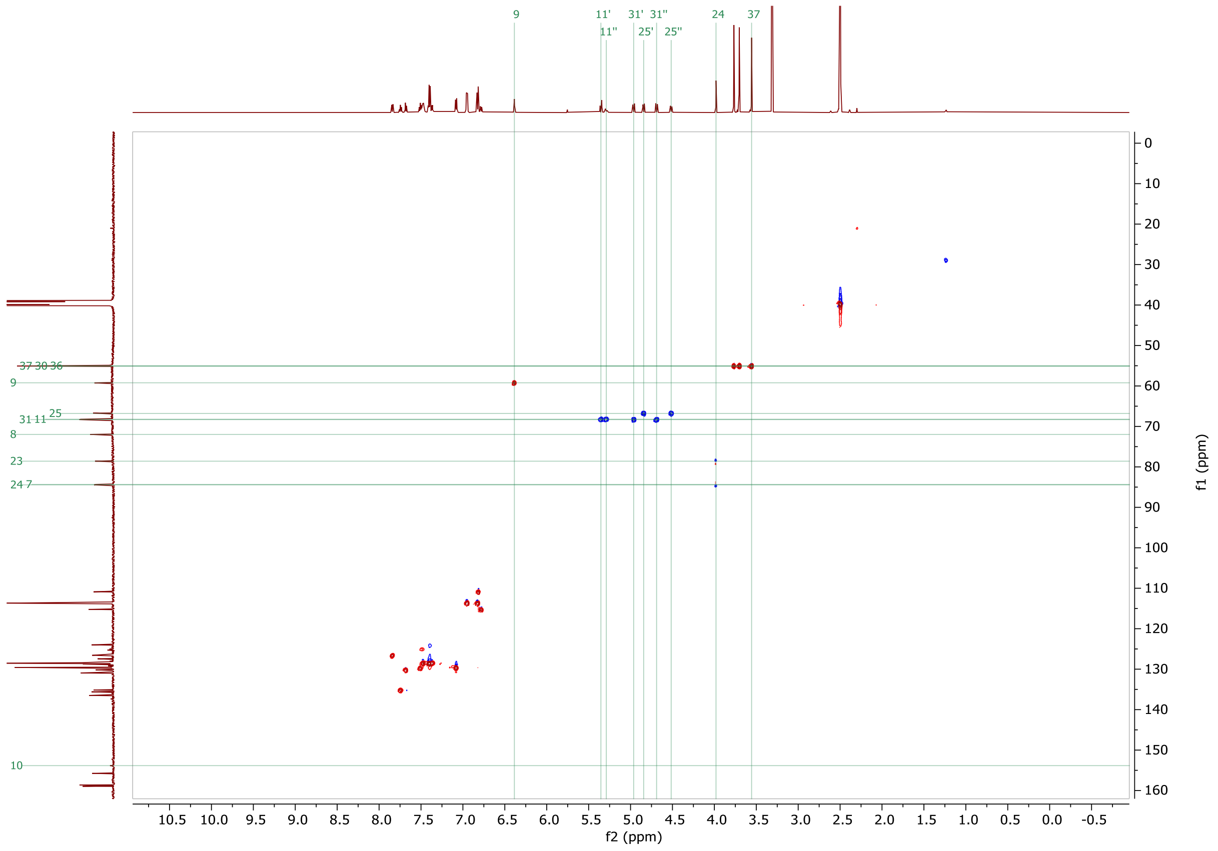

$^{1}H/^{13}C$ HMBC

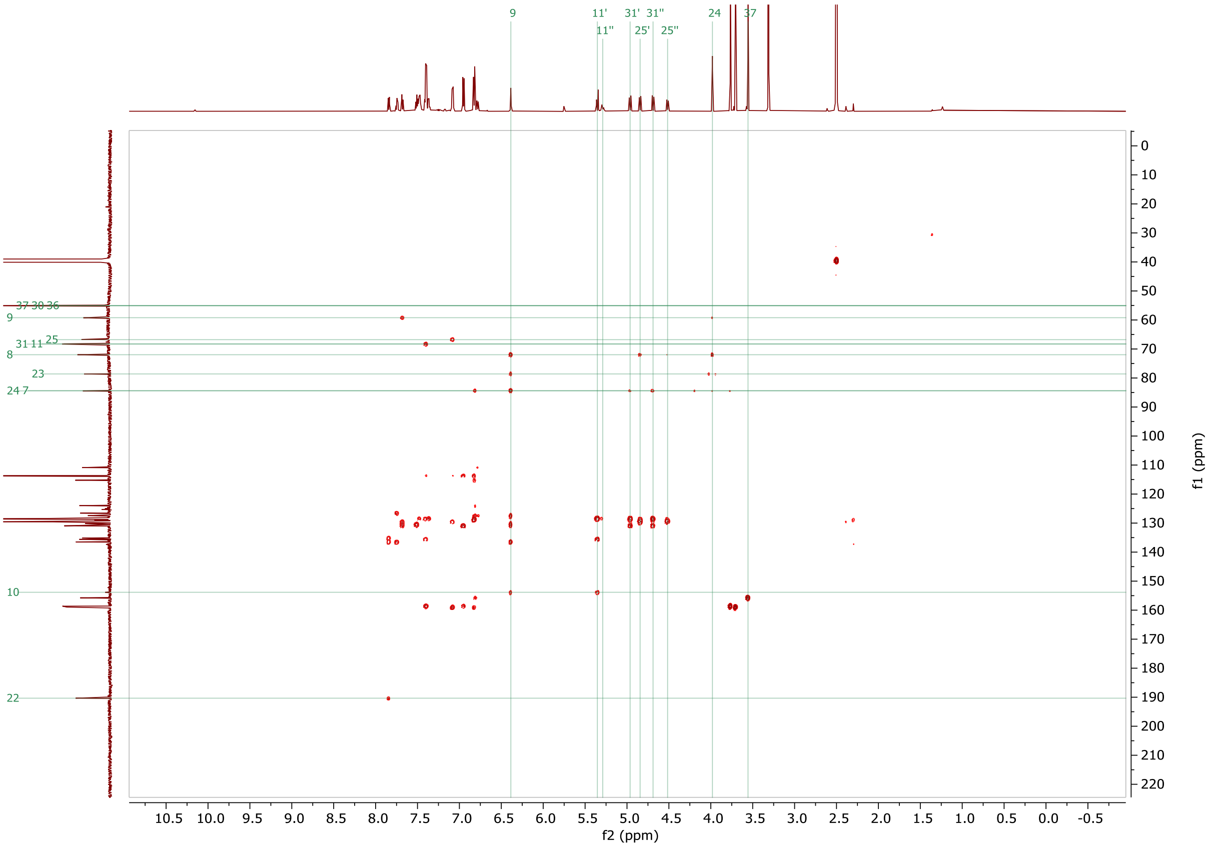
9
11'
11''
31'
25'
31''
25''
24
37
37 30 36
9
31 11
25
8
23
24 7
10
22
f1 (ppm)
f2 (ppm)

Benzyl (6*S*,11*R*,12*S*,13*S*)-13-ethynyl-11-hydroxy-2-methoxy-12,13-bis((4-methoxybenzyl)oxy)-11,12-dihydro-6,12-methanodibenzo[*b*,*f*]azocine-5(6*H*)-carboxylate 29

^{1}H NMR (500 MHz, DMSO-d_6)

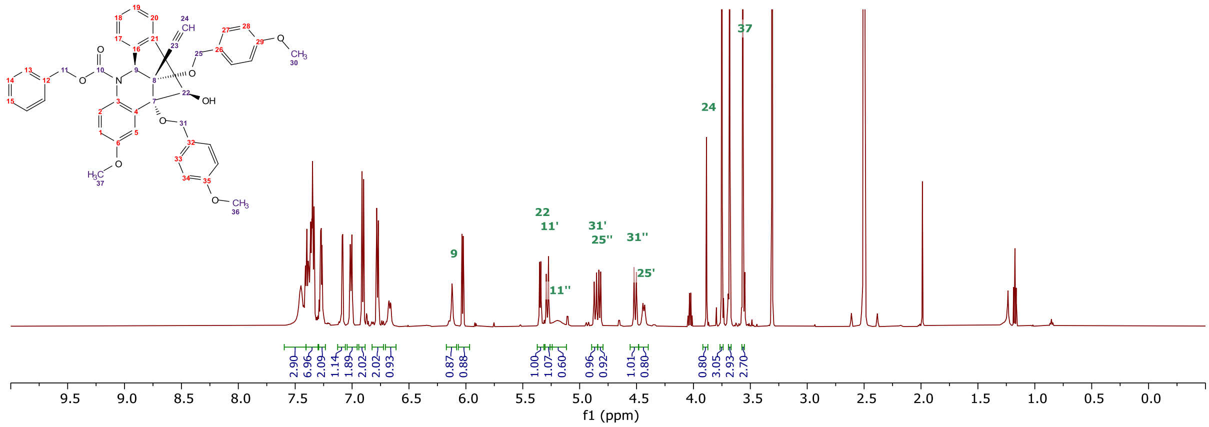

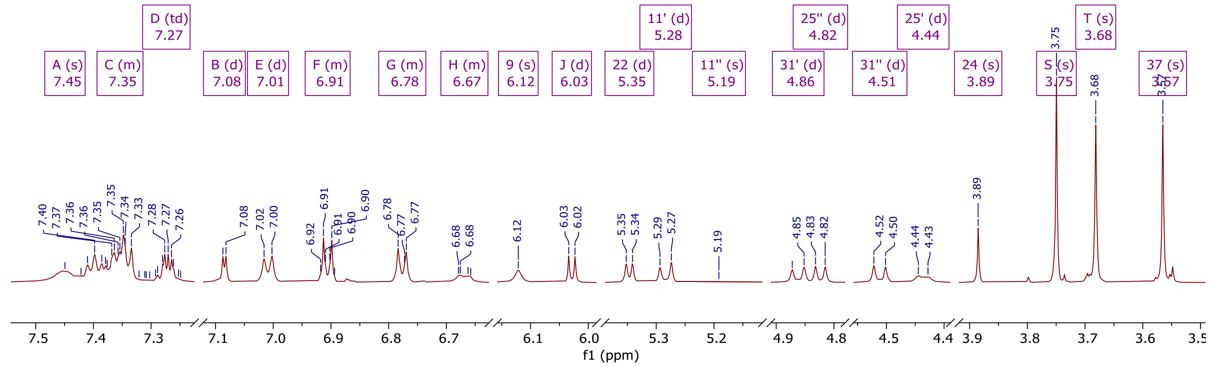

^{13}C NMR (126 MHz, DMSO-d_6)

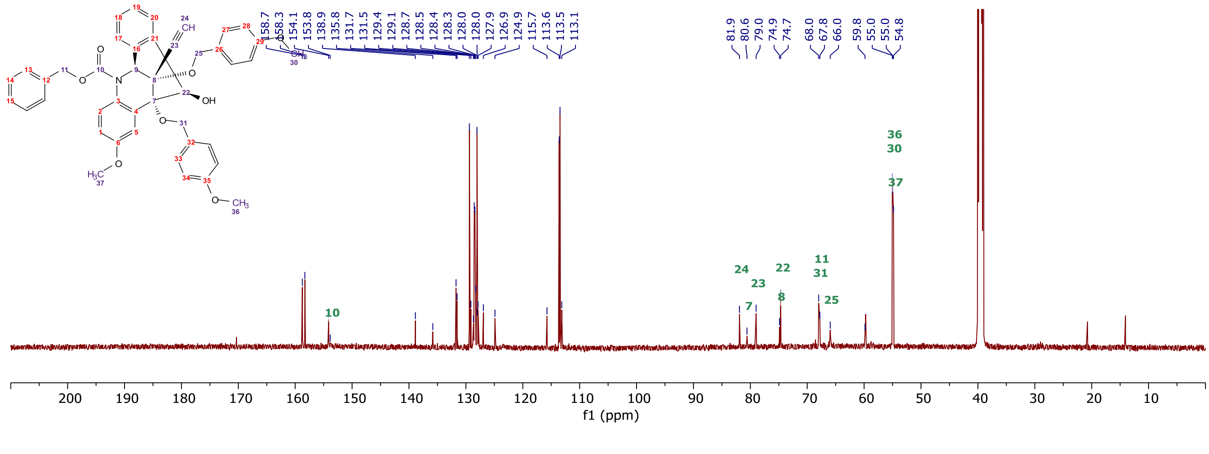

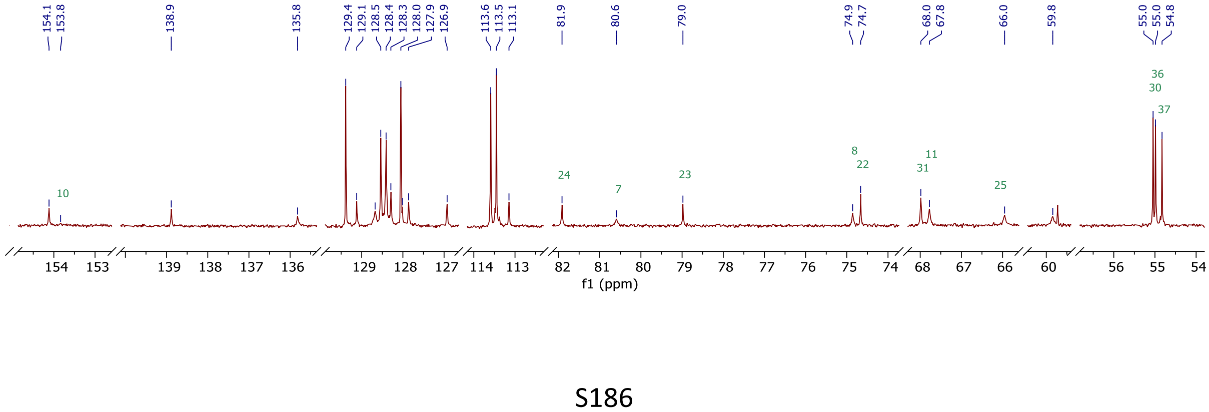

$^{1}H/^{1}H$ COSY

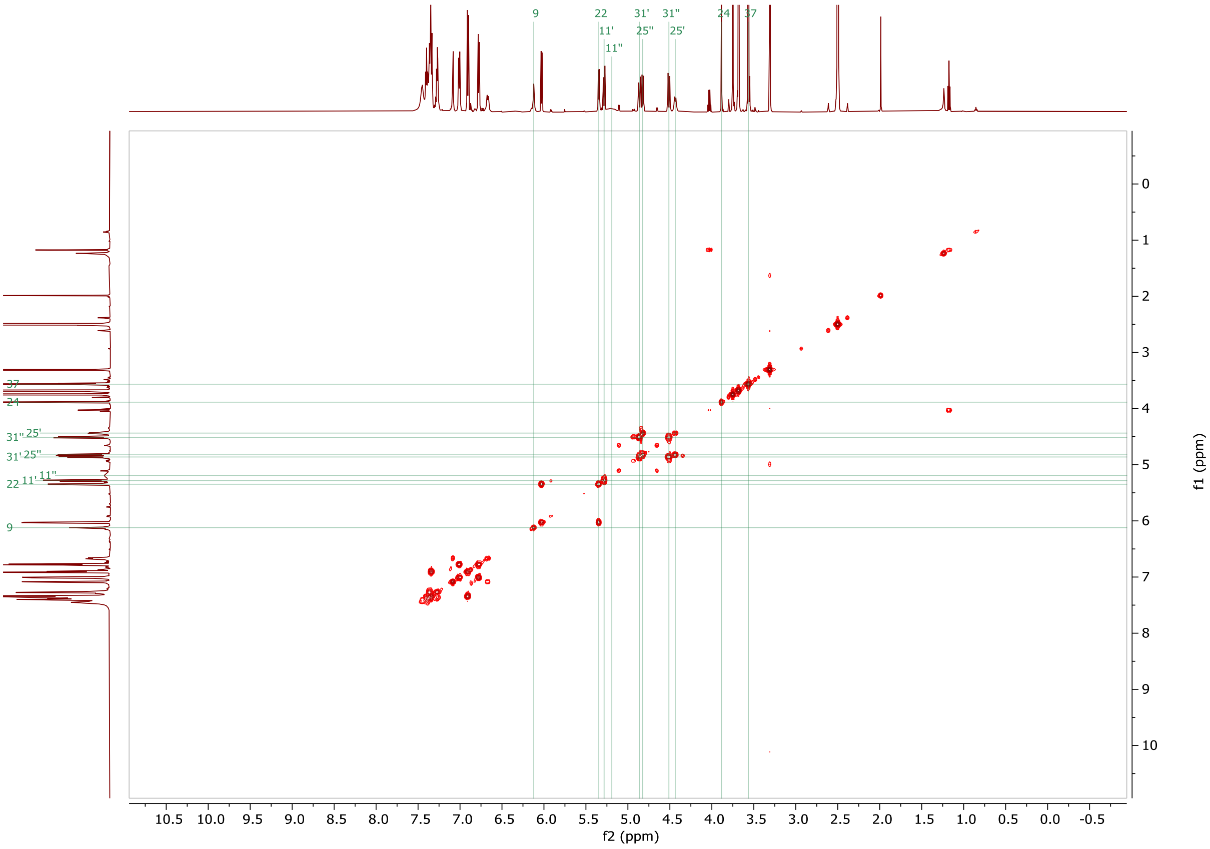

$^{1}H/^{13}C$ HSQC

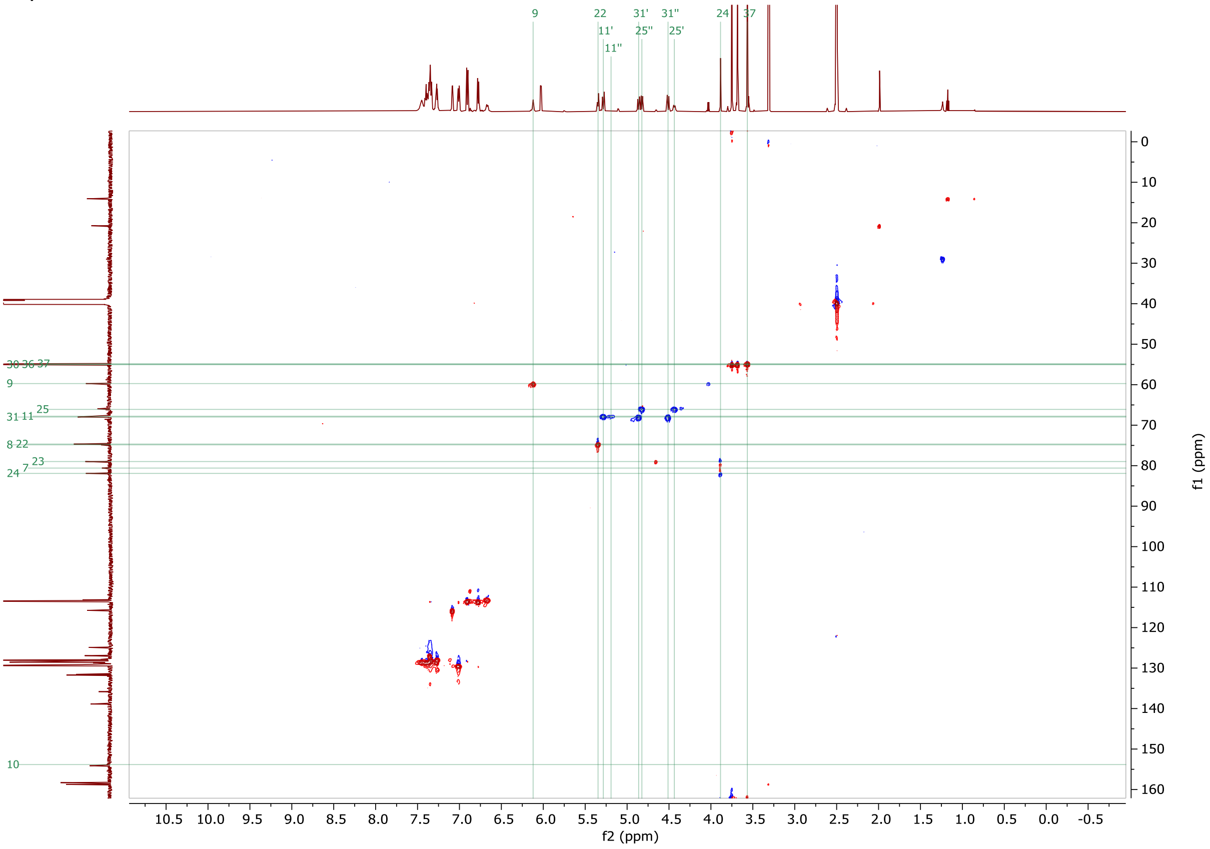

$^{1}H/^{13}C$ HMBC

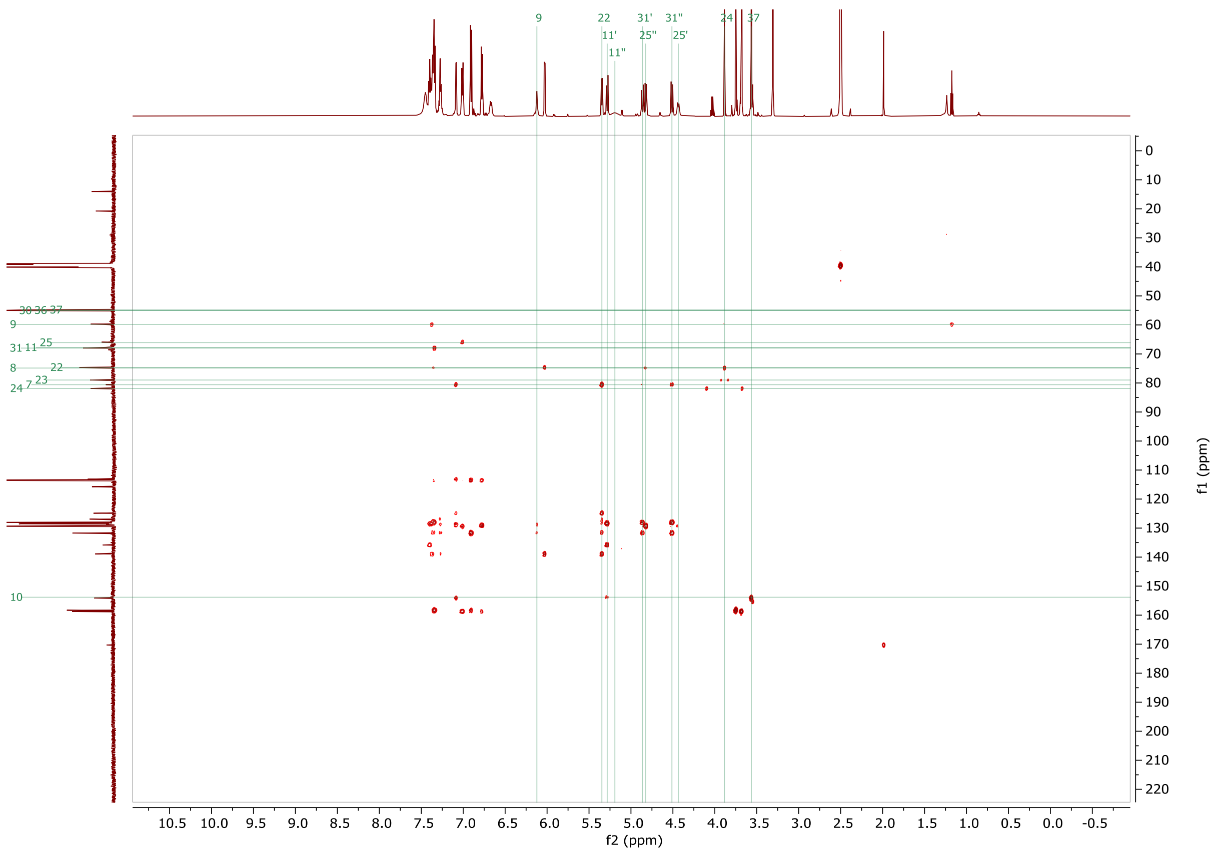

9
22
11'
11''
31'
25''
31''
25'
24
37
30 36 37
9
31 11 25
8
22
24 7 23
10
f1 (ppm)
f2 (ppm)

Benzyl (6*S*,11*R*,12*S*,13*S*)-13-ethynyl-2-methoxy-11,12,13-tris((4-methoxybenzyl)oxy)-11,12-dihydro-6,12-methanodibenzo[*b*,*f*]azocine-5(6*H*)-carboxylate S6

^{1}H NMR (500 MHz, DMSO-d_6)

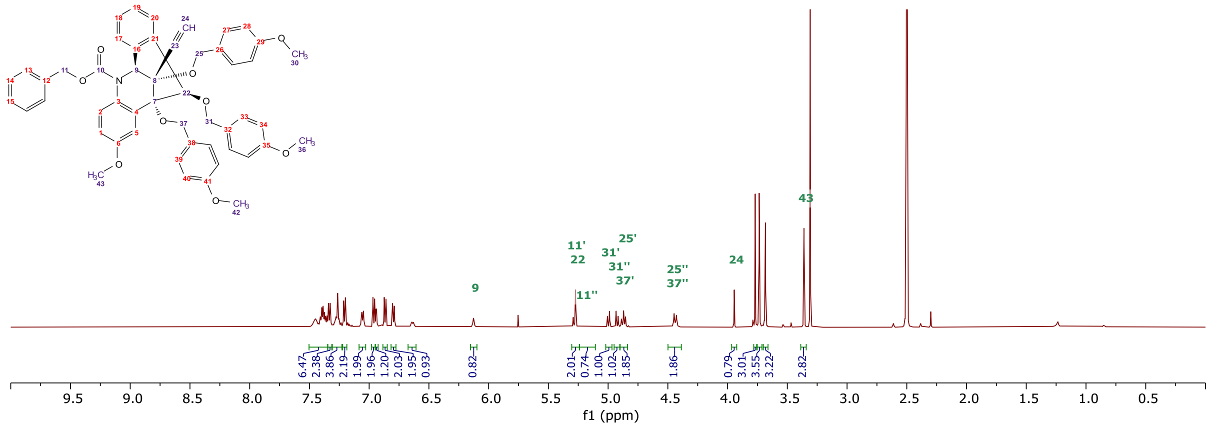

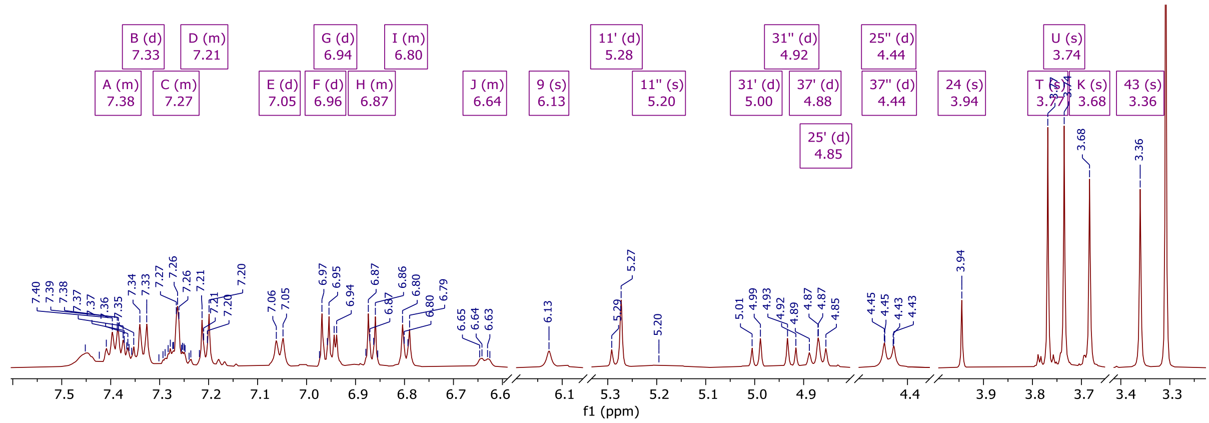

^{13}C NMR (126 MHz, DMSO-d_6)

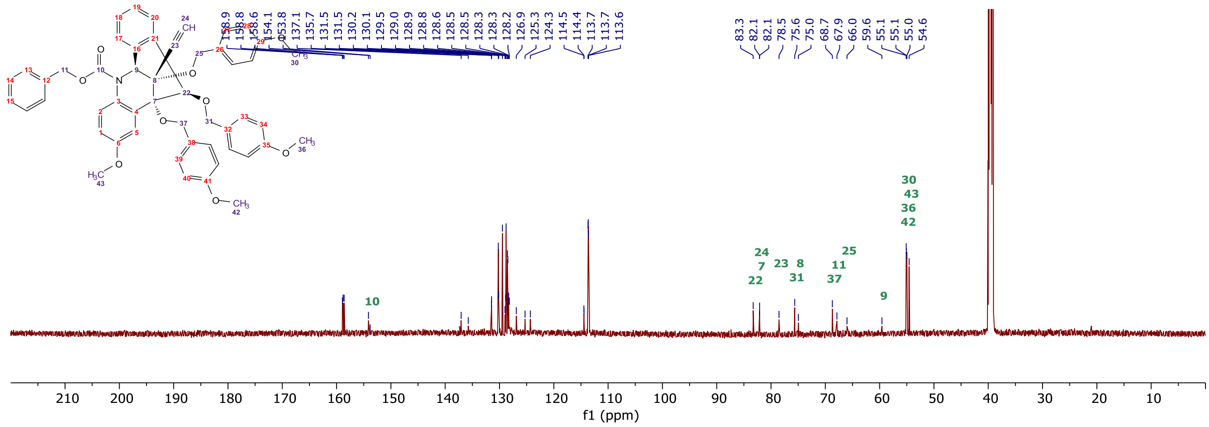

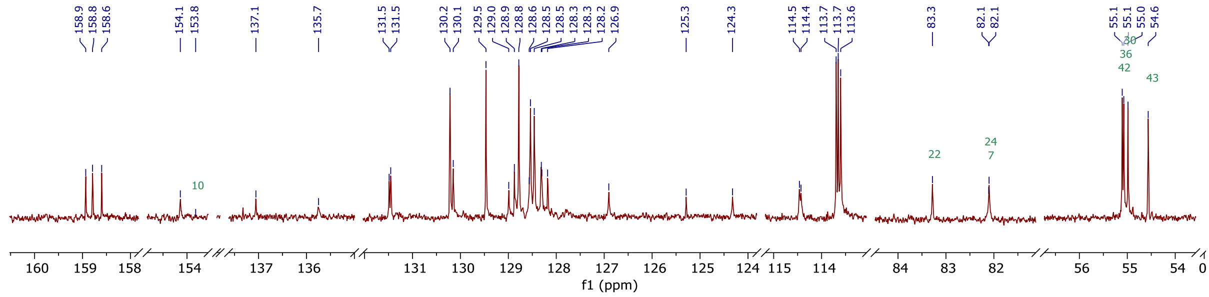

$^{1}H/^{1}H$ COSY

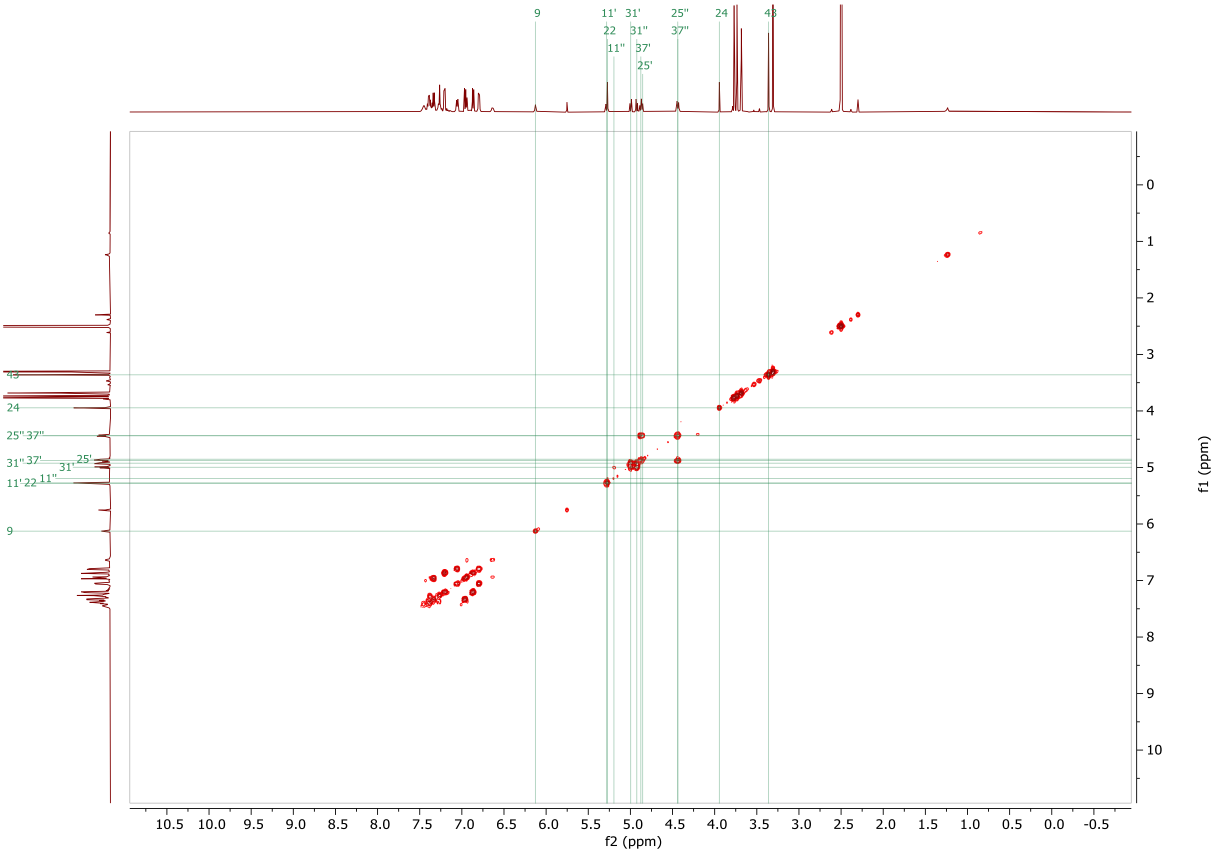

$^{1}H/^{13}C$ HSQC

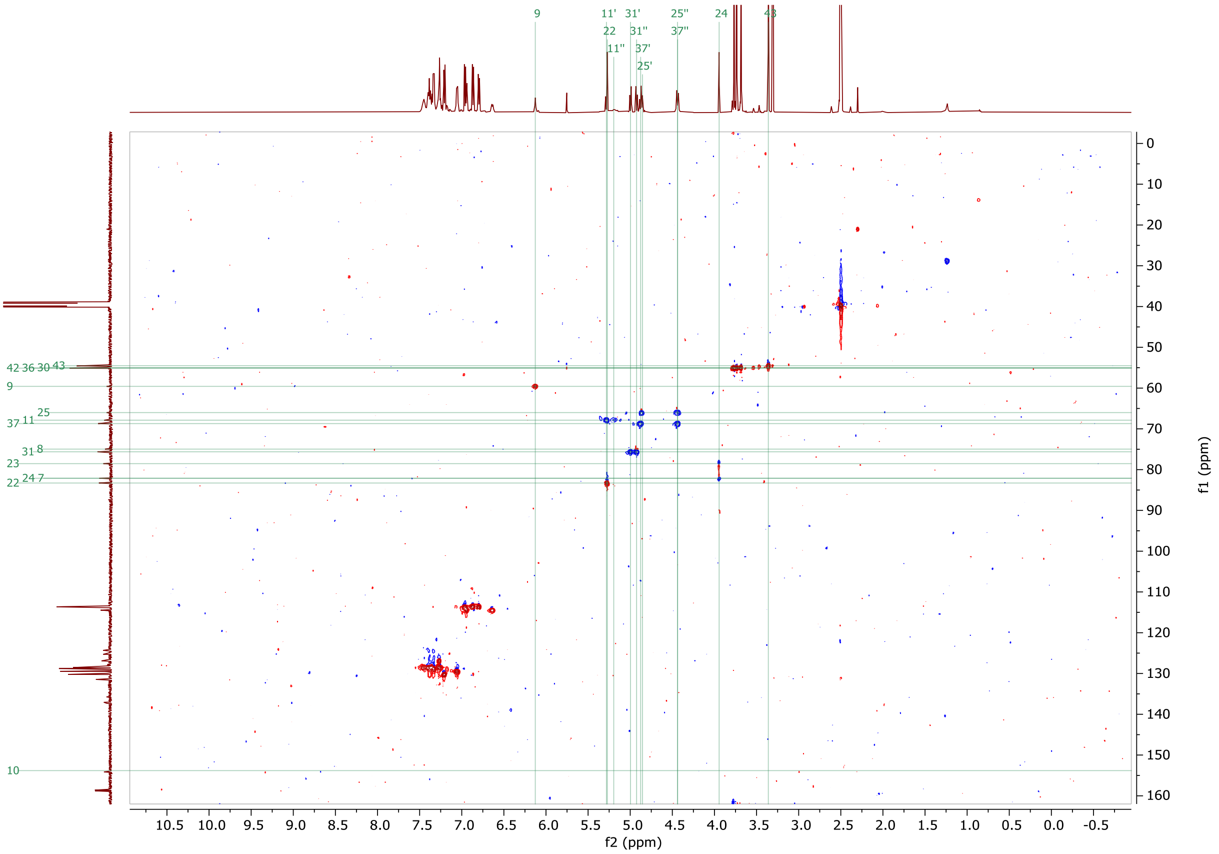

$^{1}H/^{13}C$ HMBC

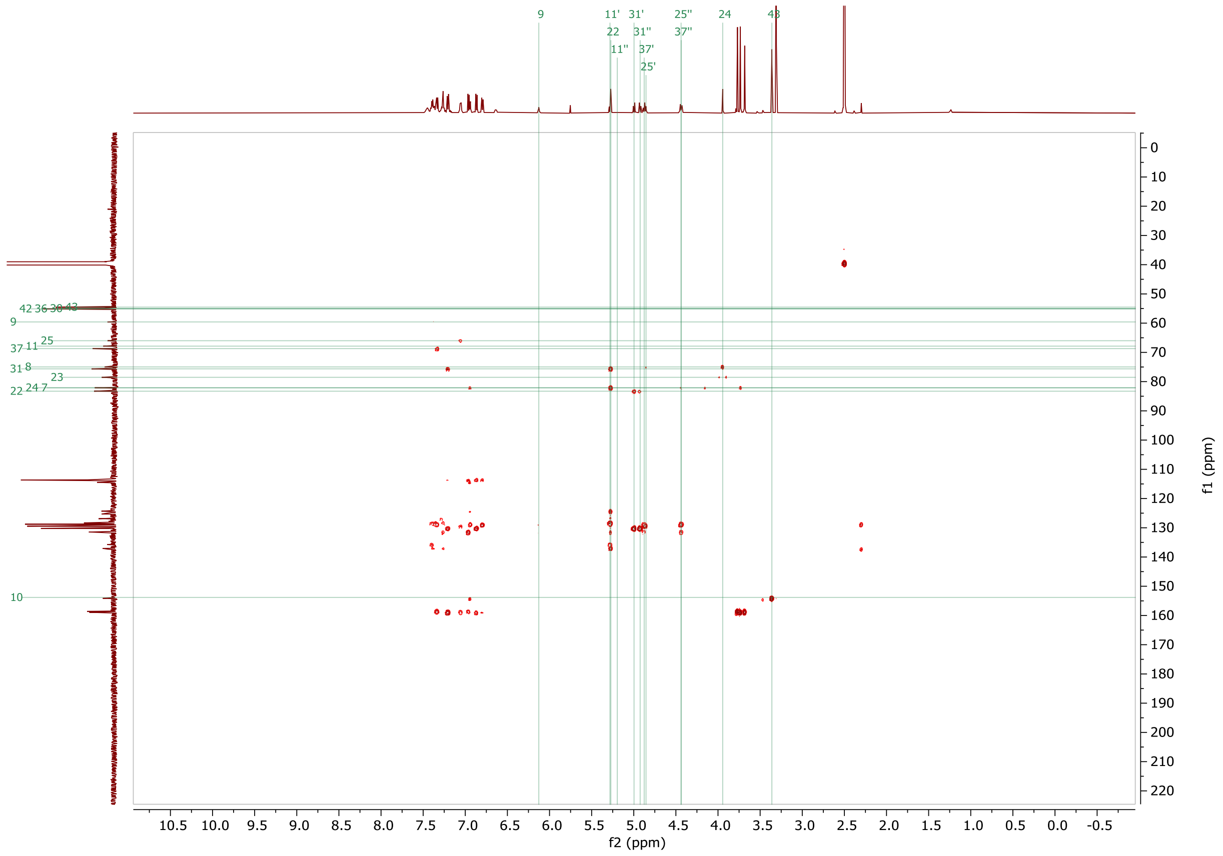
9
11'
22
11"
31'
31"
37'
25'
25"
37"
24
43
42 36 30 42
9
37 11 25
31 8
23
22 24 7
10
f1 (ppm)
f2 (ppm)

Benzyl (6*S*,11*R*,12*S*,13*S*)-2-methoxy-13-(3-methoxy-3-oxoprop-1-yn-1-yl)-11,12,13-tris((4-methoxybenzyl)oxy)-11,12-dihydro-6,12-methanodibenzo[*b*,*f*]azocine-5(6*H*)-carboxylate 30

^{1}H NMR (500 MHz, DMSO-d_6)

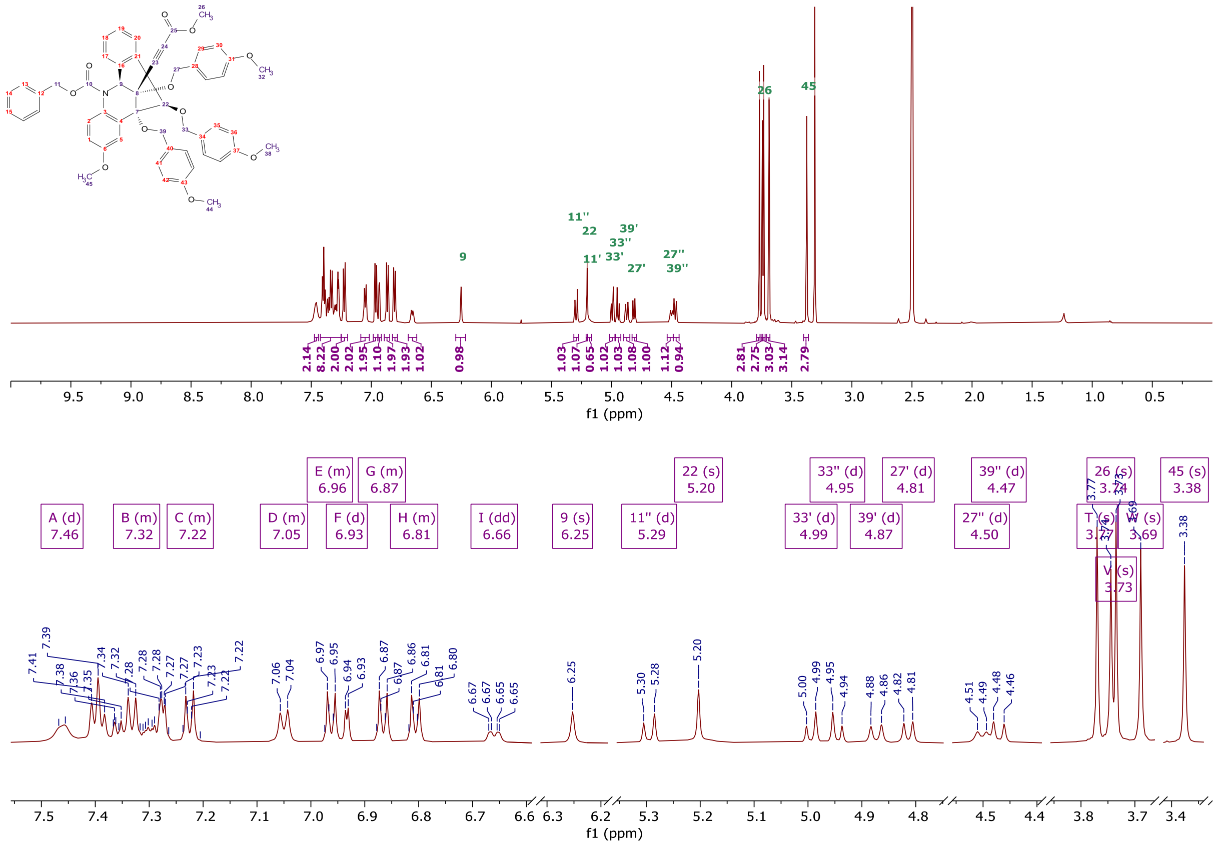

^{13}C NMR (126 MHz, DMSO-d_6)

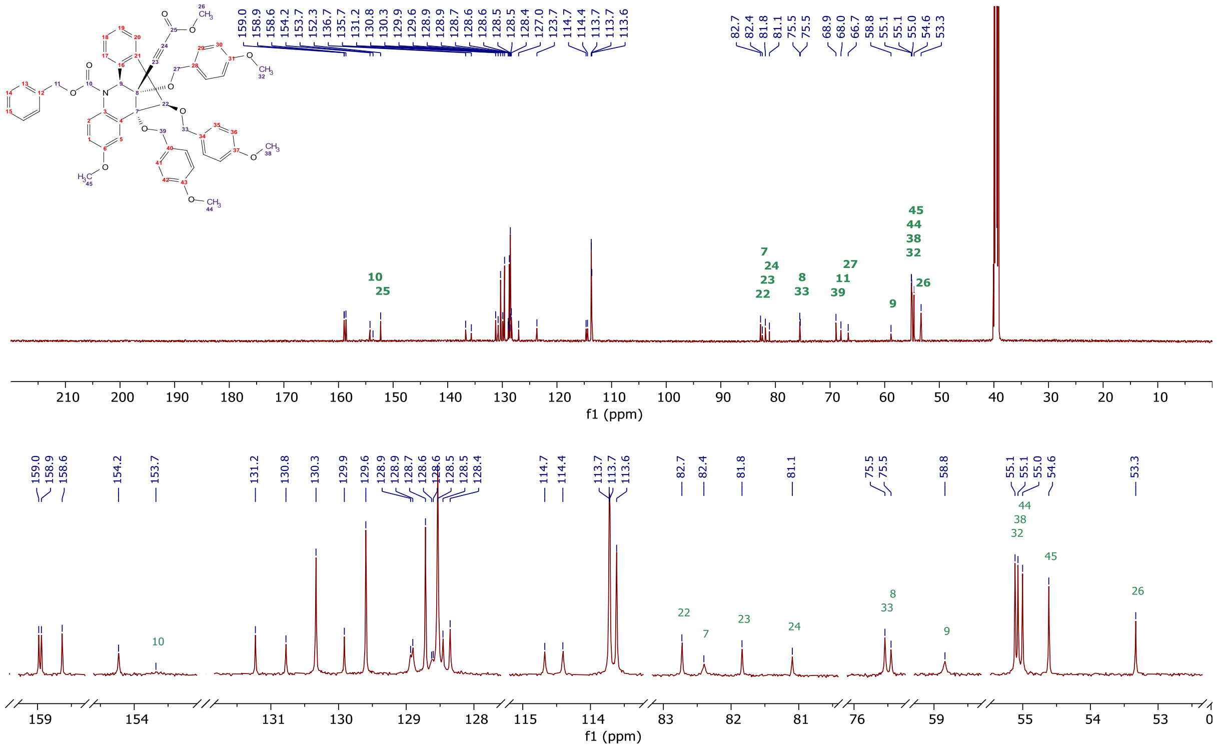

$^{1}H/^{1}H$ COSY

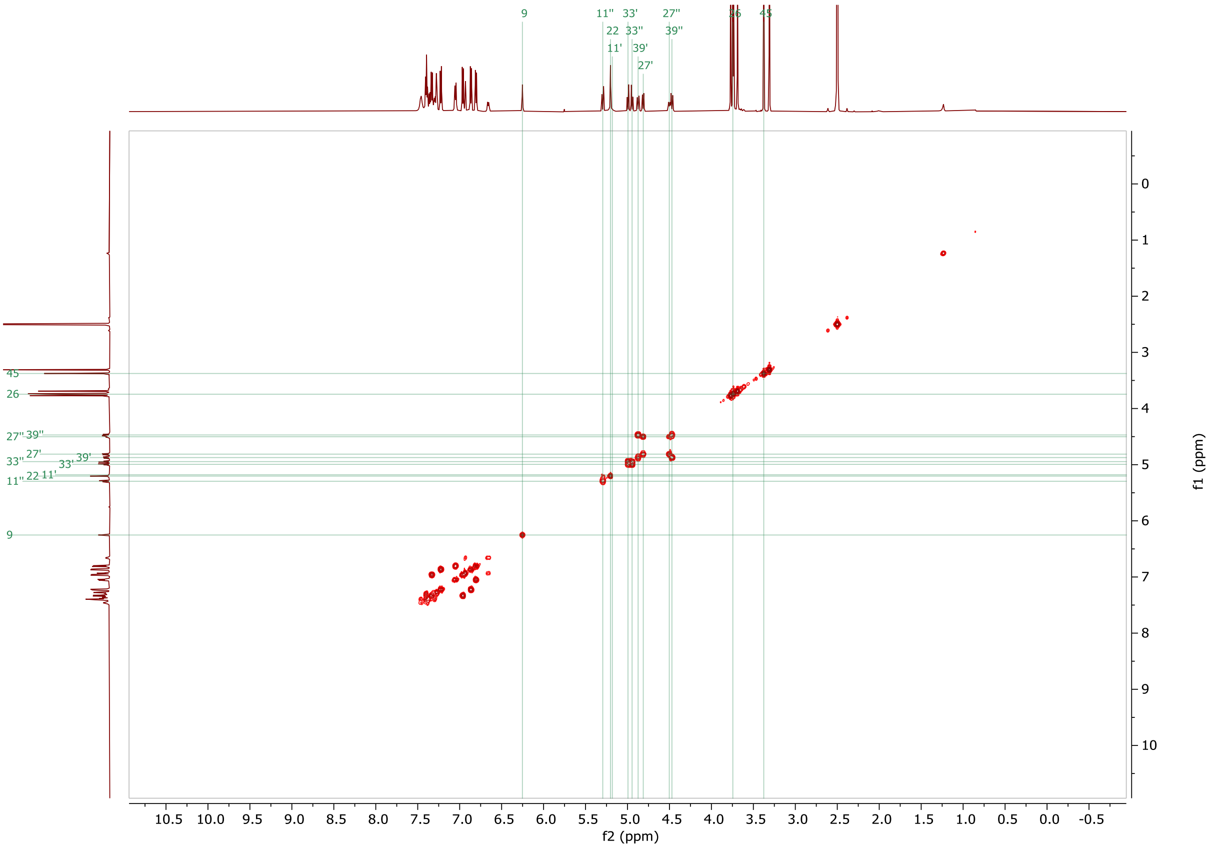

$^{1}H/^{13}C$ HSQC

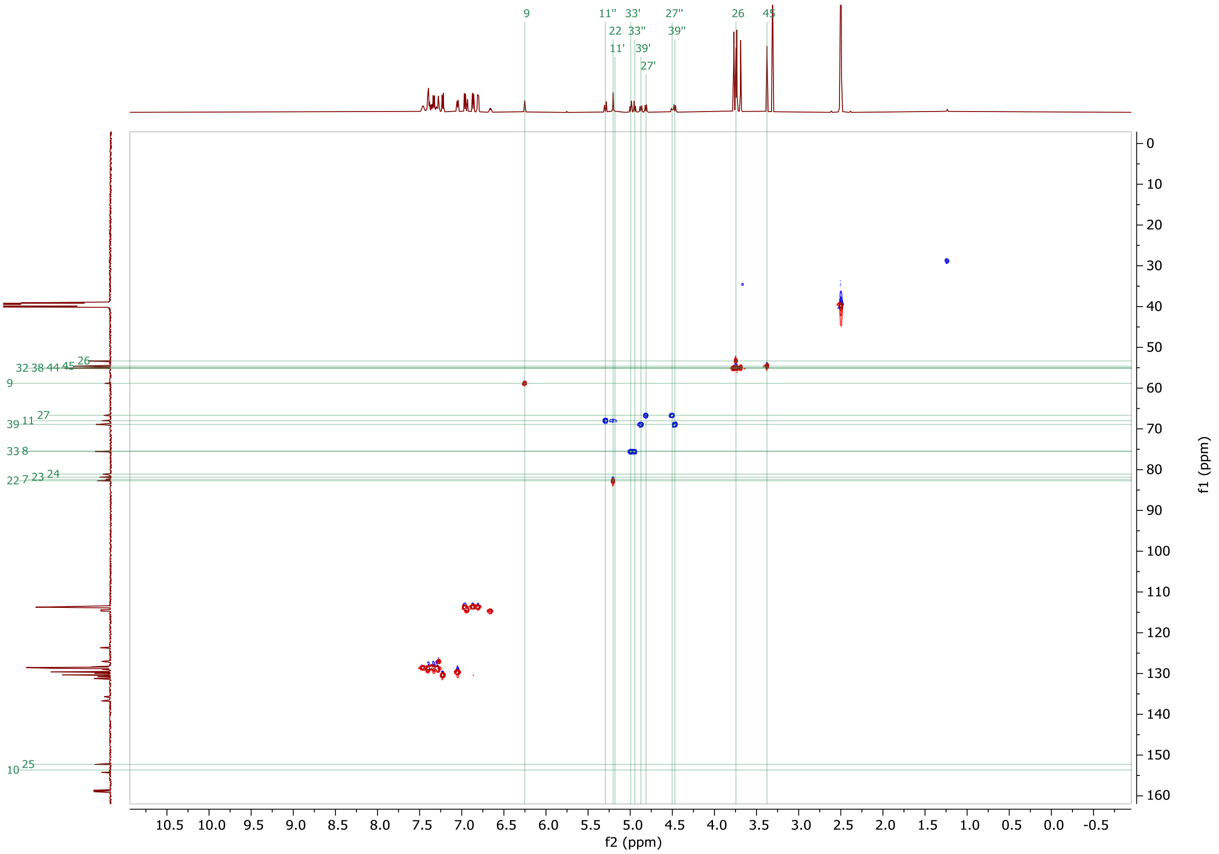

$^{1}H/^{13}C$ HMBC

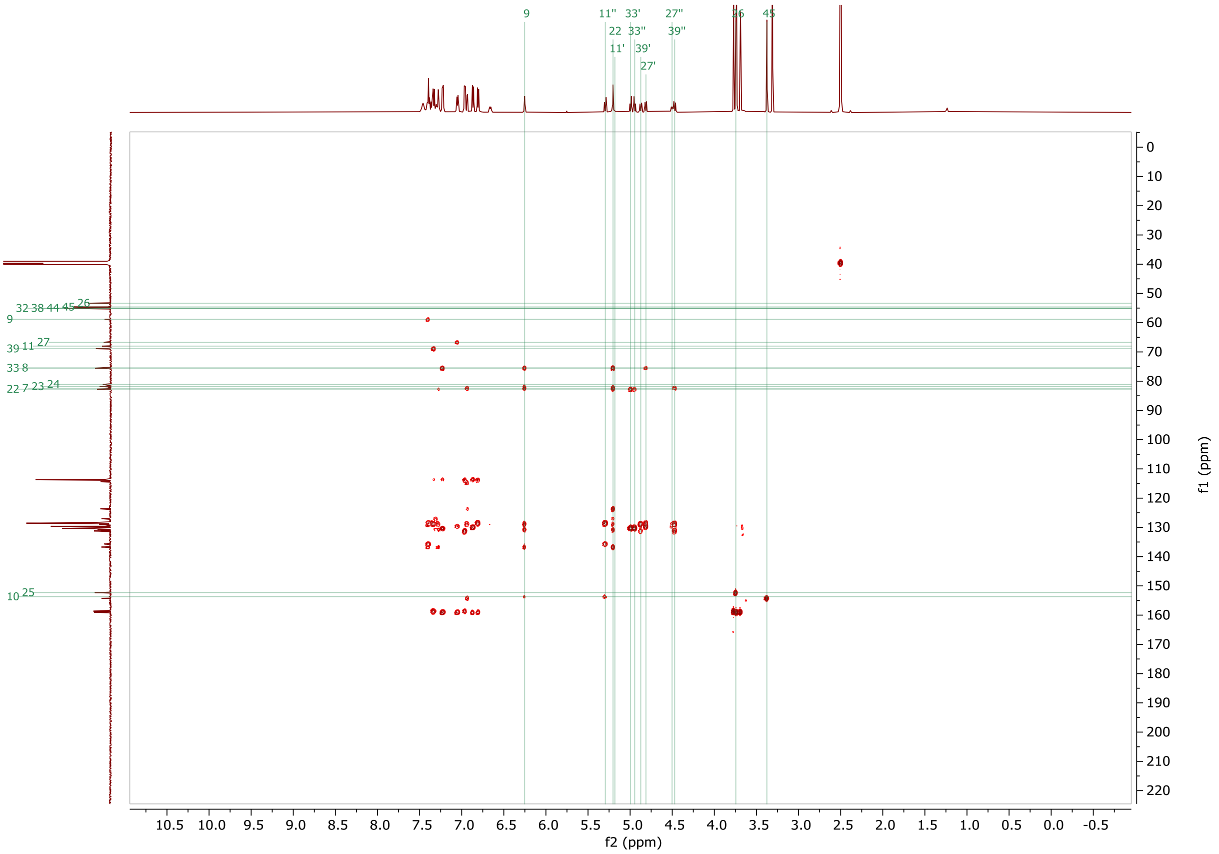

9
11''
22
11'
33'
33''
39'
27'
27''
39''
36
45
32 38 44 45 26
9
39 11 27
33 8
22 7 23 24
10 25
f1 (ppm)
f2 (ppm)

Benzyl (6*S*,11*R*,12*S*,13*S*)-2-methoxy-13-((*E*)-4-methoxy-4-oxobut-2-en-2-yl)-11,12,13-tris((4-methoxybenzyl)oxy)-11,12-dihydro-6,12-methanodibenzo[*b*,*f*]azocine-5(6*H*)-carboxylate 31

^{1}H NMR (500 MHz, DMSO-d_6)

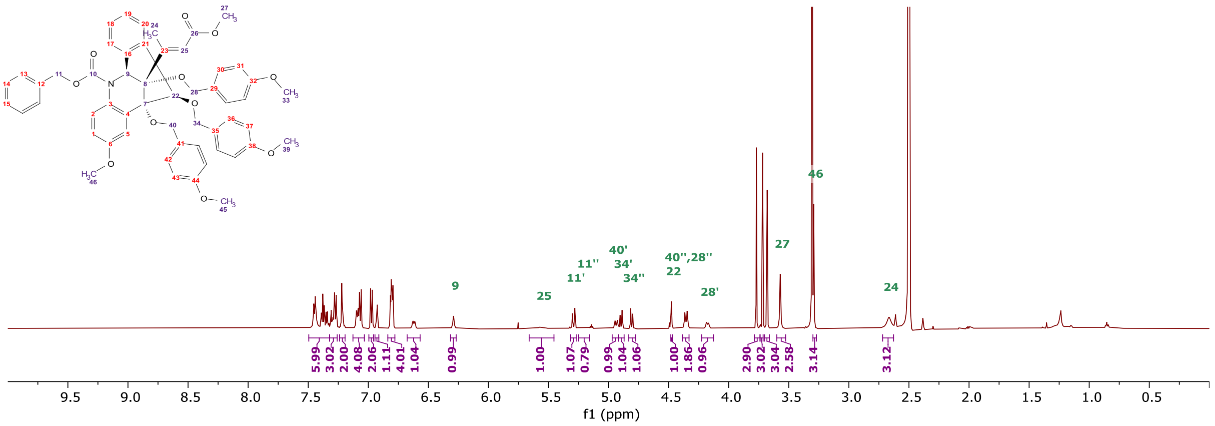

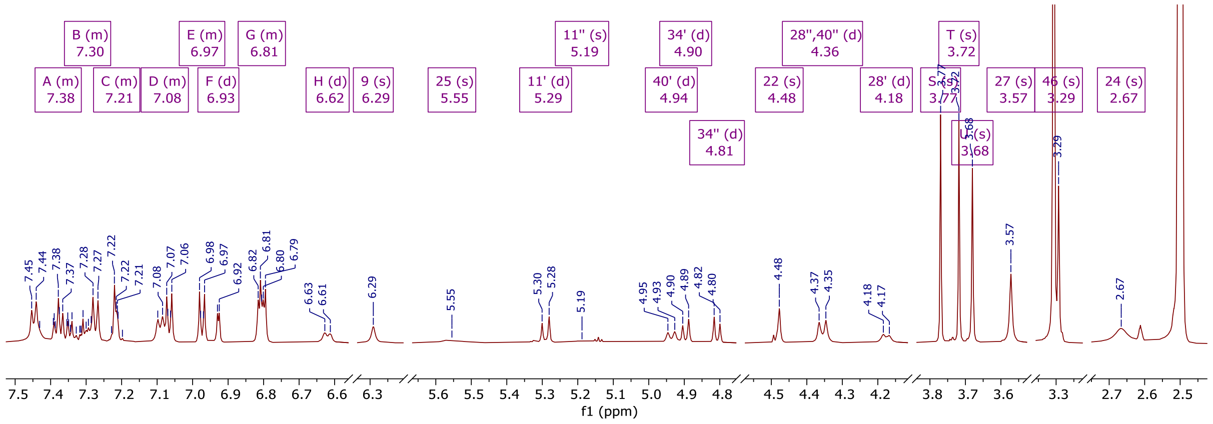

^{13}C NMR (126 MHz, DMSO-d_6)

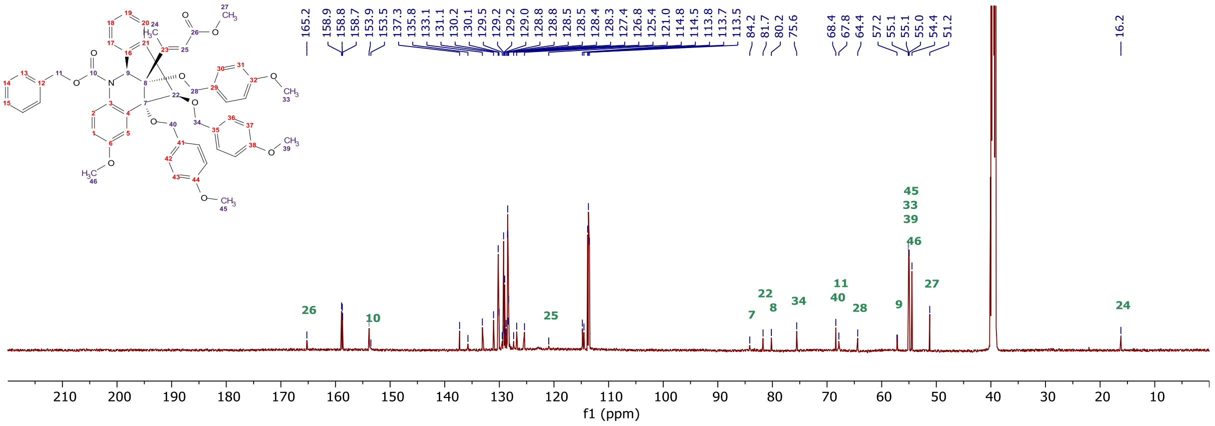

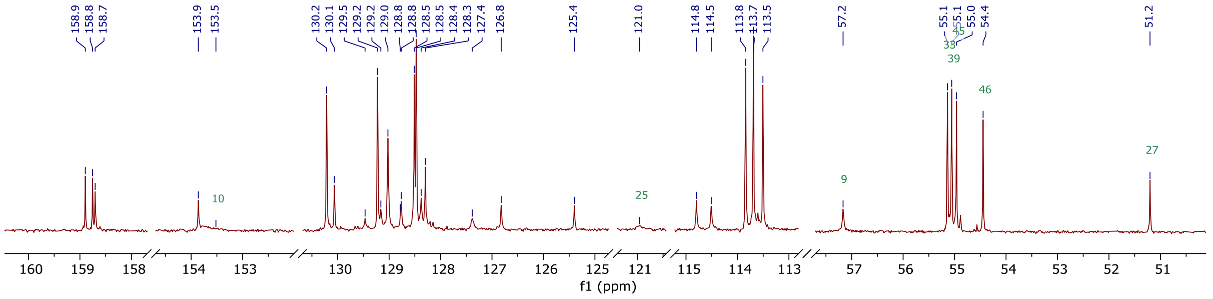

$^{1}H/^{1}H$ COSY

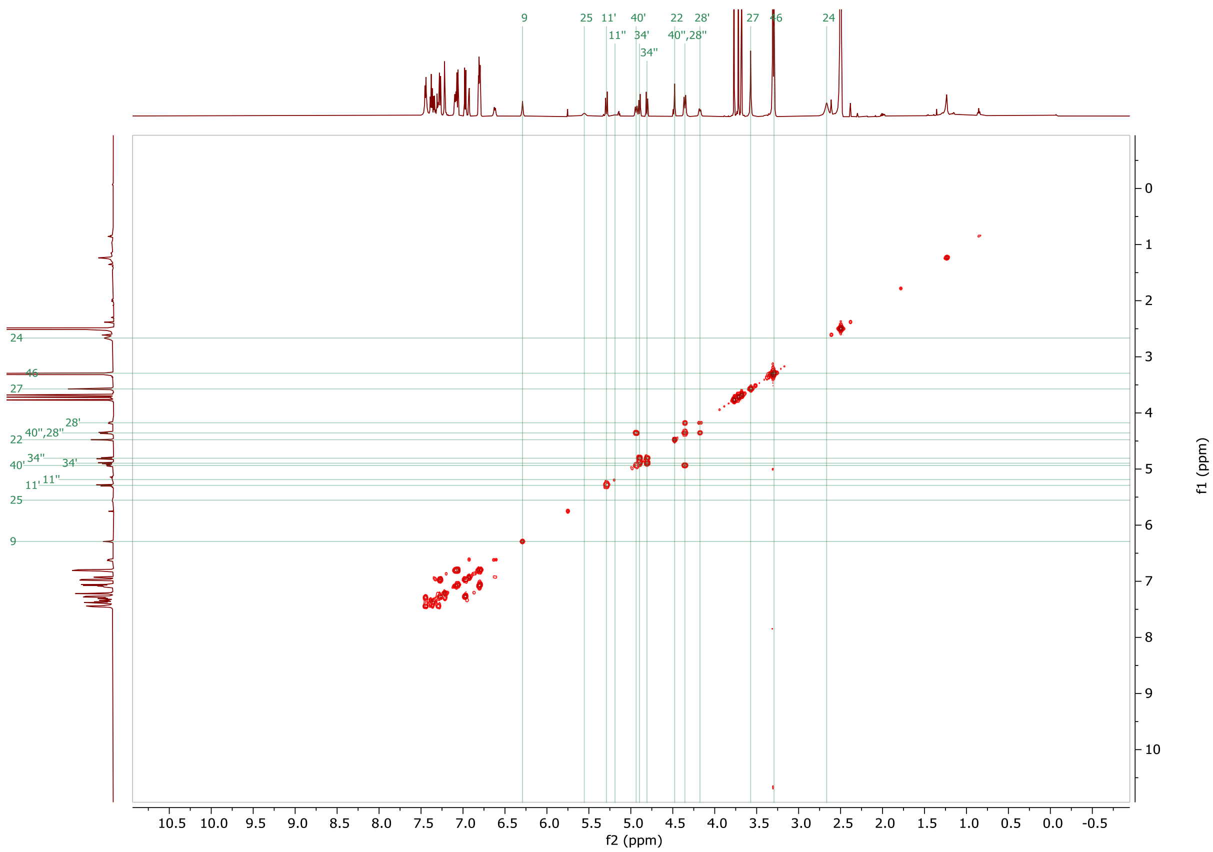

$^{1}H/^{13}C$ HSQC

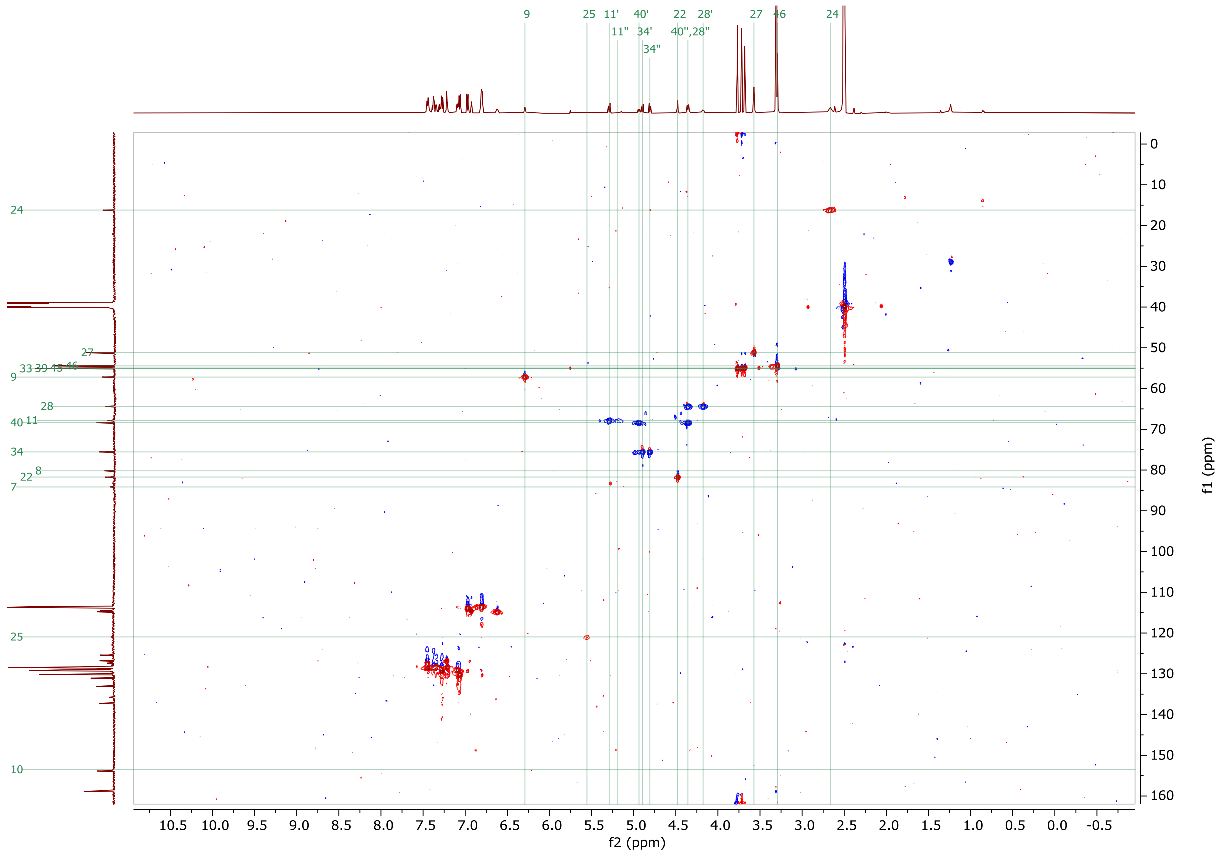

$^{1}H/^{13}C$ HMBC

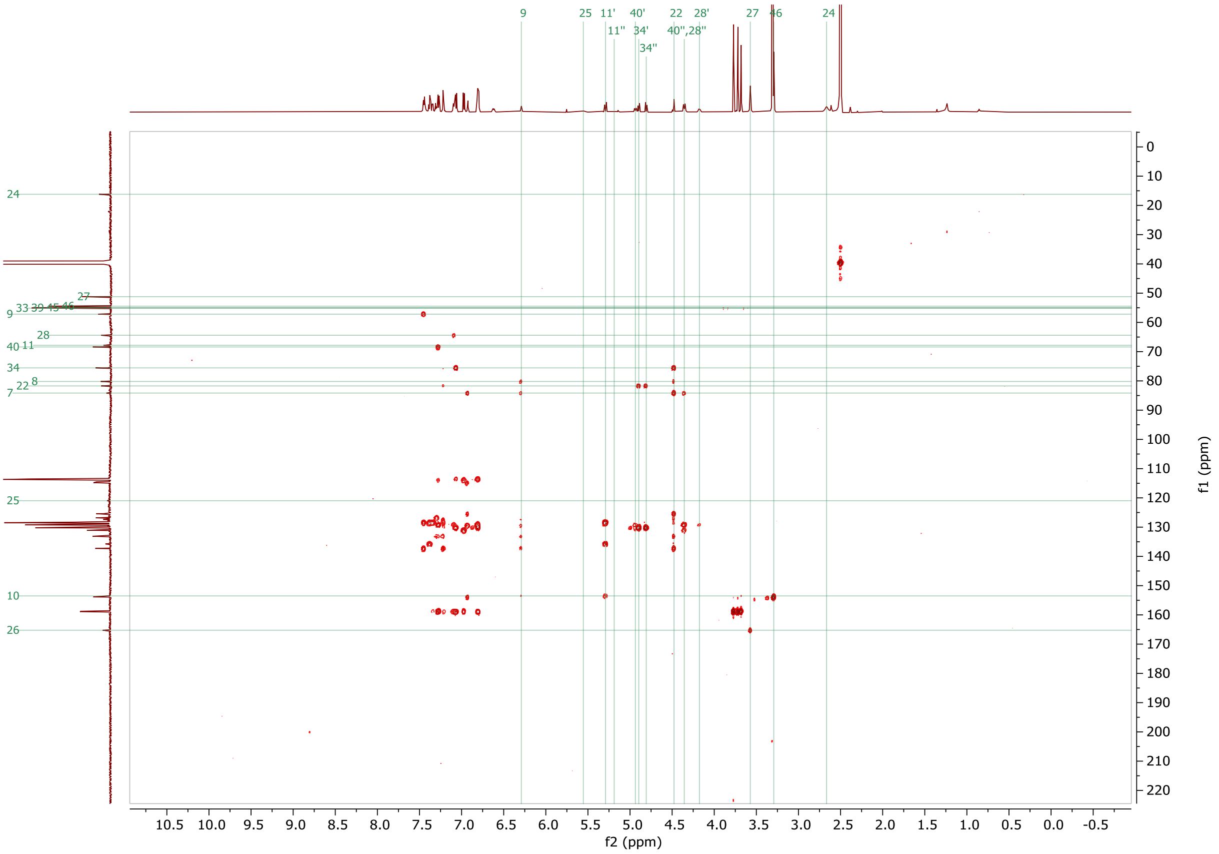
9
25
11'
11''
40'
34'
34''
22
40'',28''
28'
27
46
24
24
27
33 39 45 46
9
28
40 11
34
22 8
7
25
10
26
f2 (ppm)
f1 (ppm)

Methyl (*E*)-3-((6*S*,11*R*,12*S*,13*S*)-2-methoxy-11,12,13-tris((4-methoxybenzyl)oxy)-5,6,11,12-tetrahydro-6,12-methanodibenzo[*b*,*f*]azocin-13-yl)but-2-enoate S7

^{1}H NMR (500 MHz, DMSO-d_6)

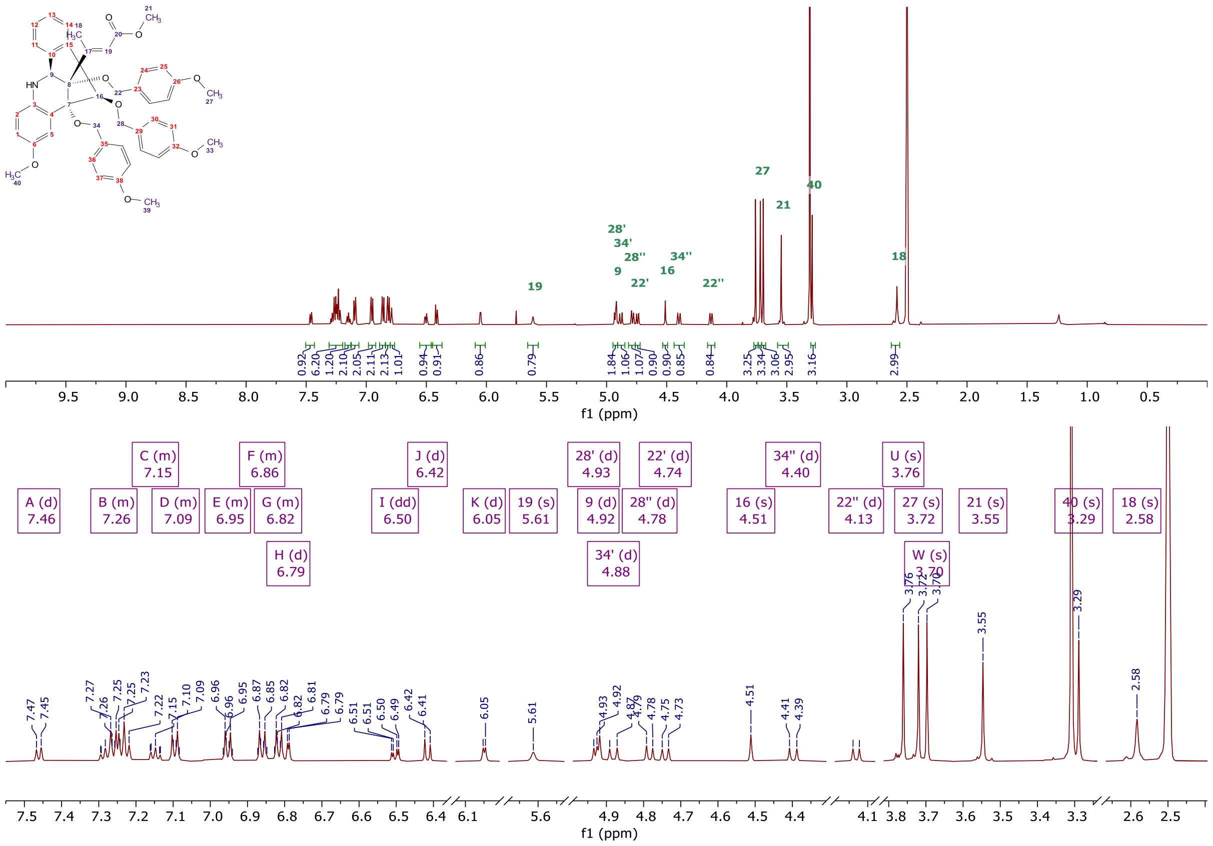

^{13}C NMR (126 MHz, DMSO-d_6)

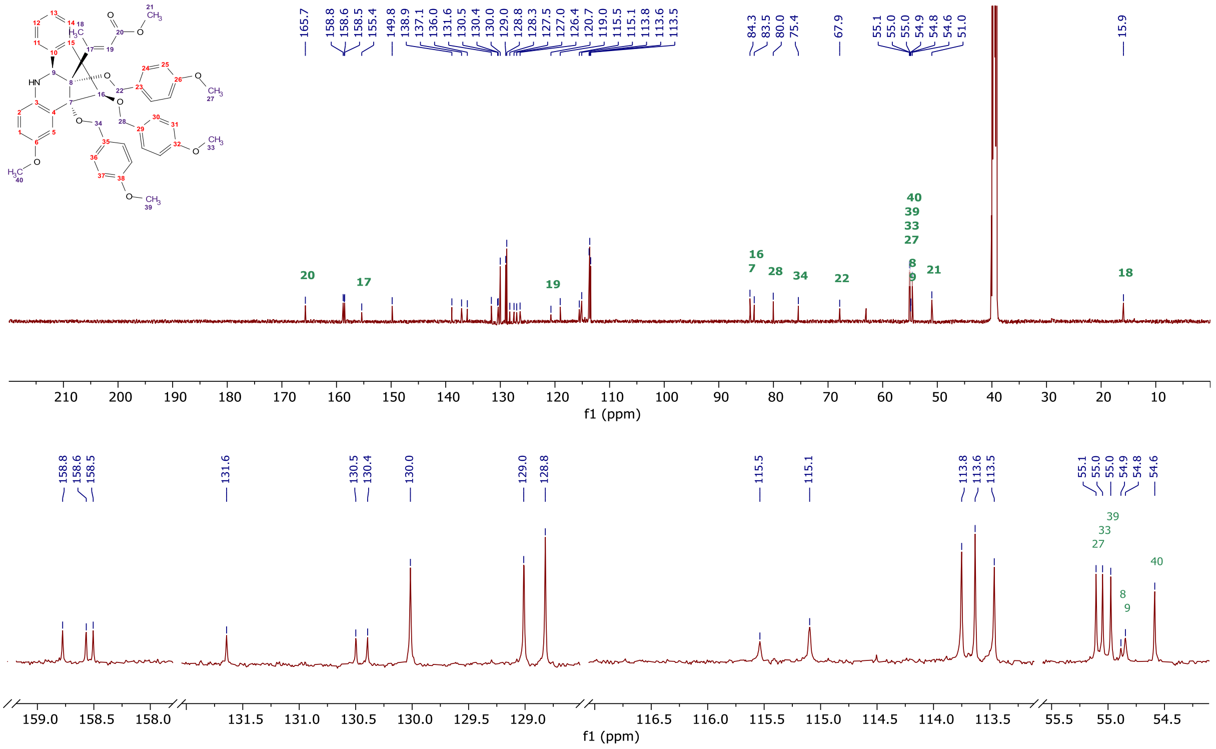

$^{1}H/^{1}H$ COSY

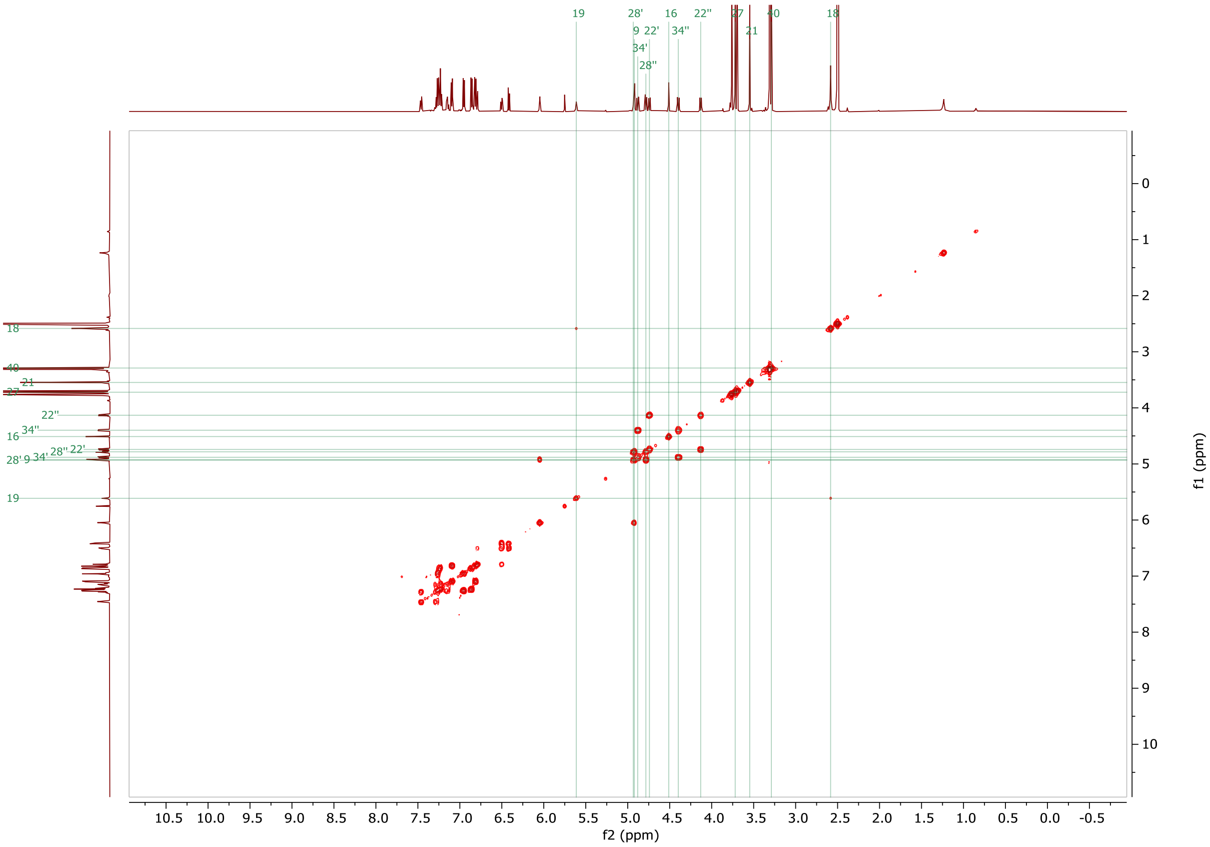

$^{1}H/^{13}C$ HSQC

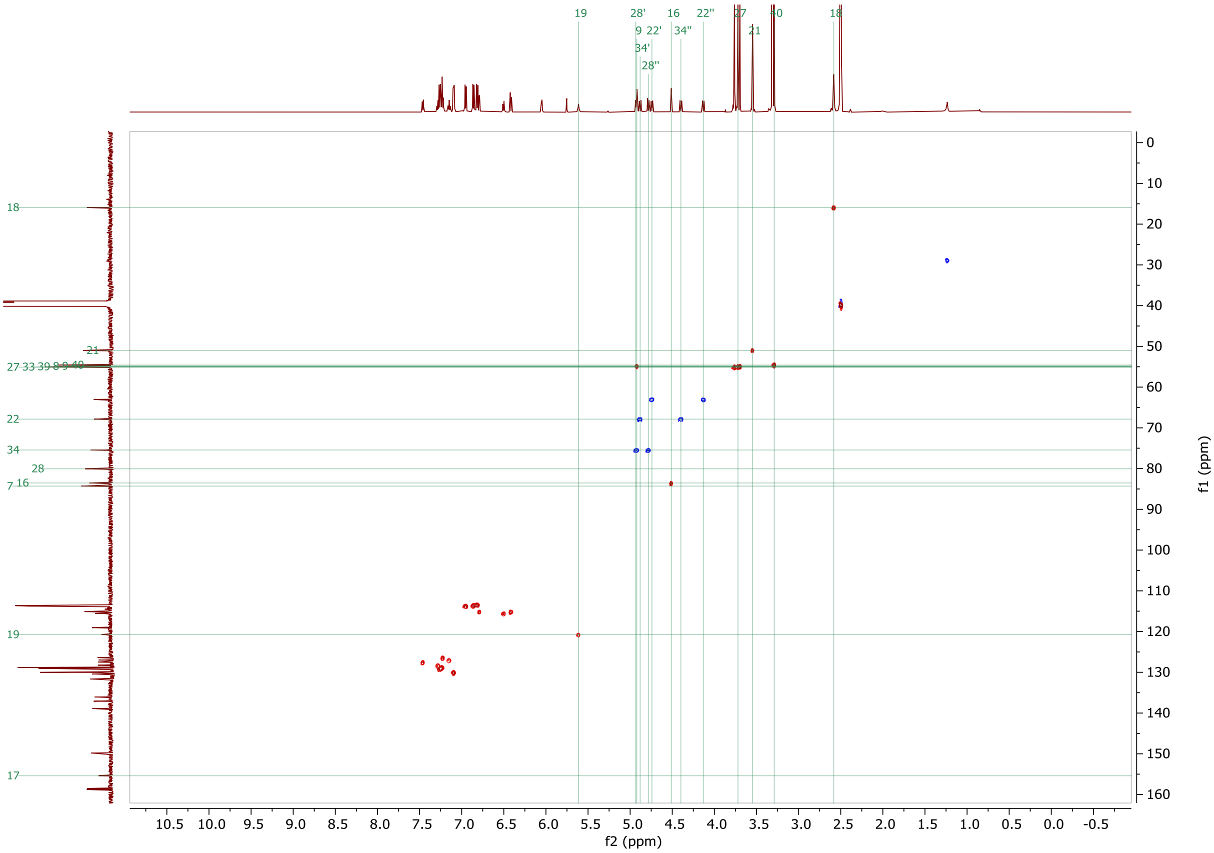

$^{1}H/^{13}C$ HMBC

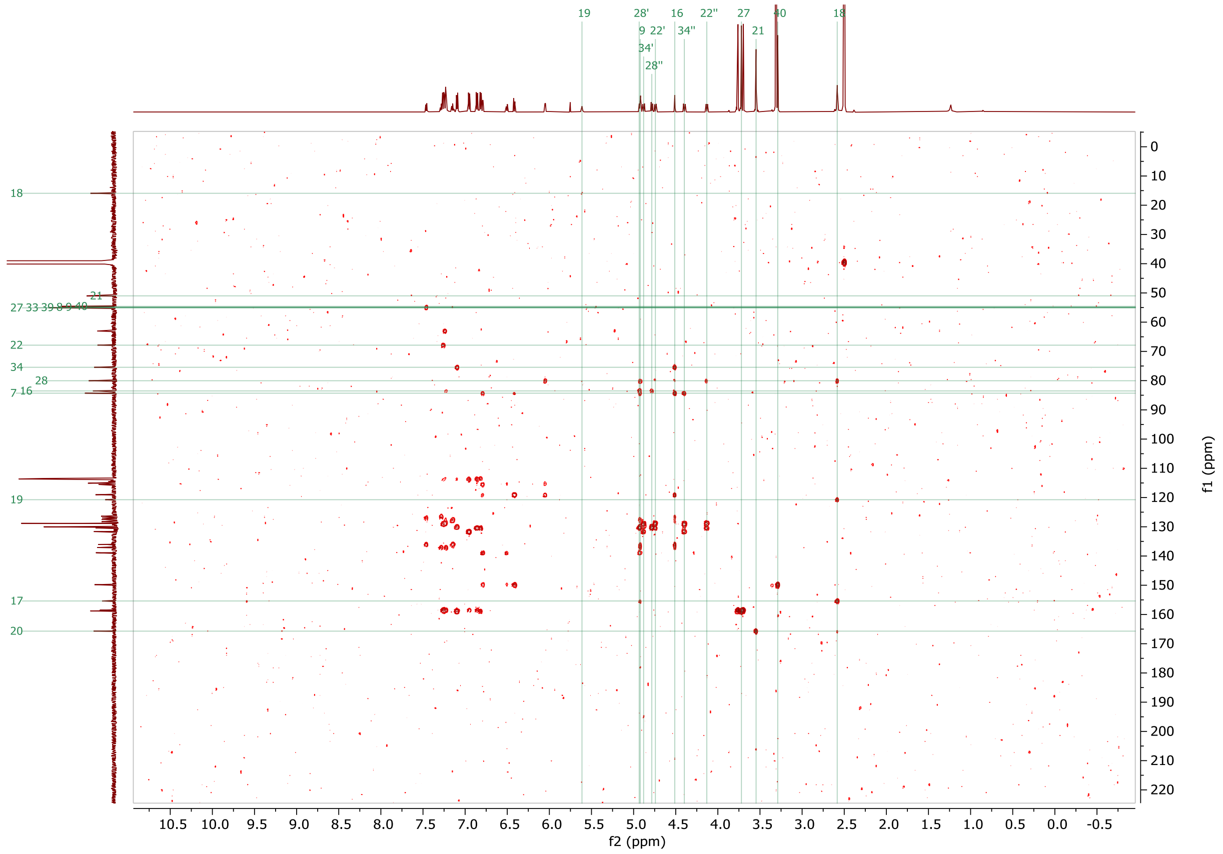
19
28'
9
22'
34'
28''
16
34''
22''
27
21
40
18
18
21
27 33 39 8 9 40
22
34
28
7 16
19
17
20
f2 (ppm)
f1 (ppm)

Methyl (E)-3-((6*S*,11*R*,12*S*,13*S*)-11,12,13-tris((4-methoxybenzyl)oxy)-2-oxo-2,6,11,12-tetrahydro-6,12-methanodibenzo[*b*,*f*]azocin-13-yl)but-2-enoate 32

^{1}H NMR (500 MHz, DMSO-d_6)

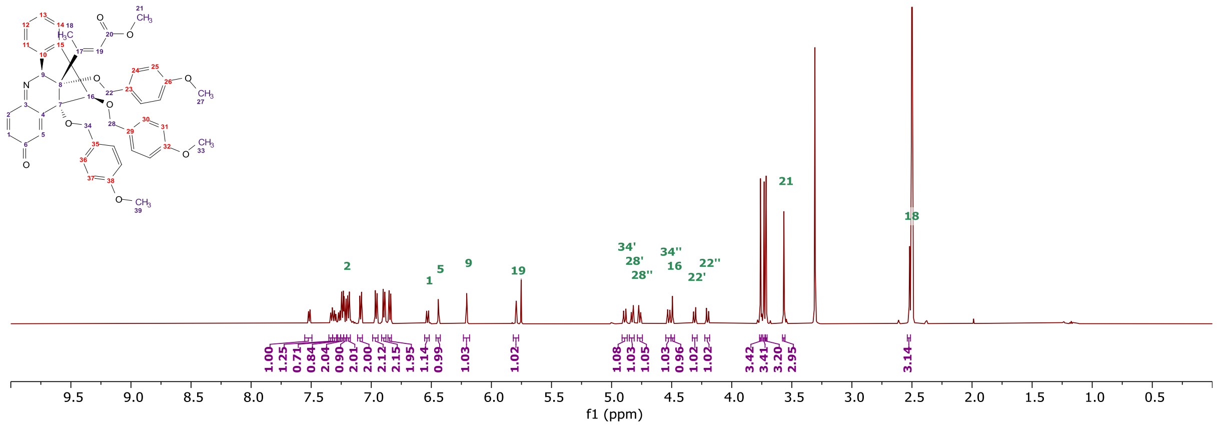

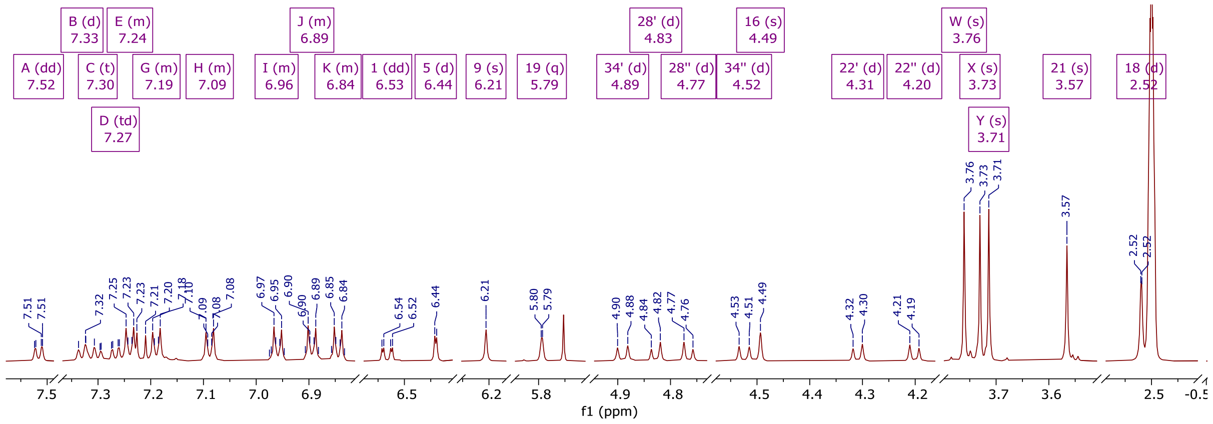

^{13}C NMR (126 MHz, DMSO-d_6)

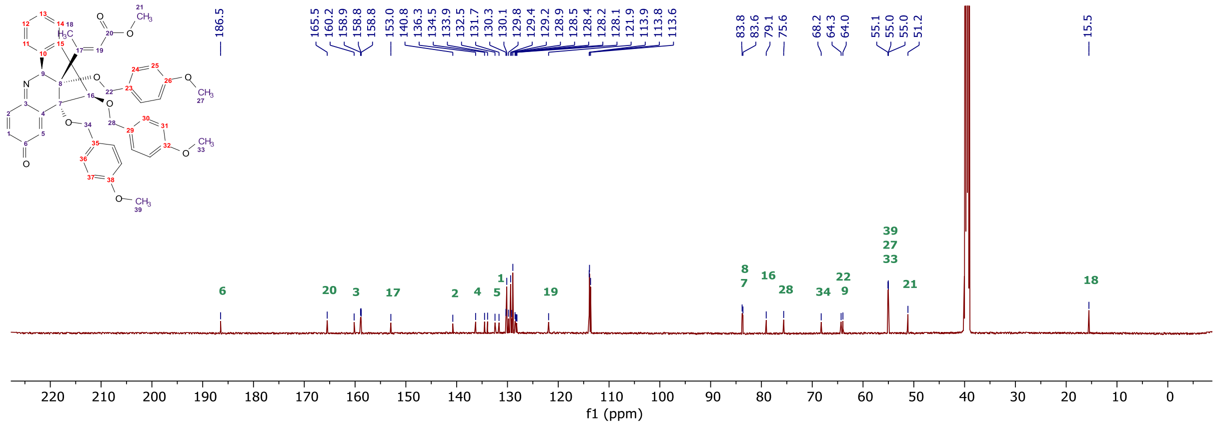

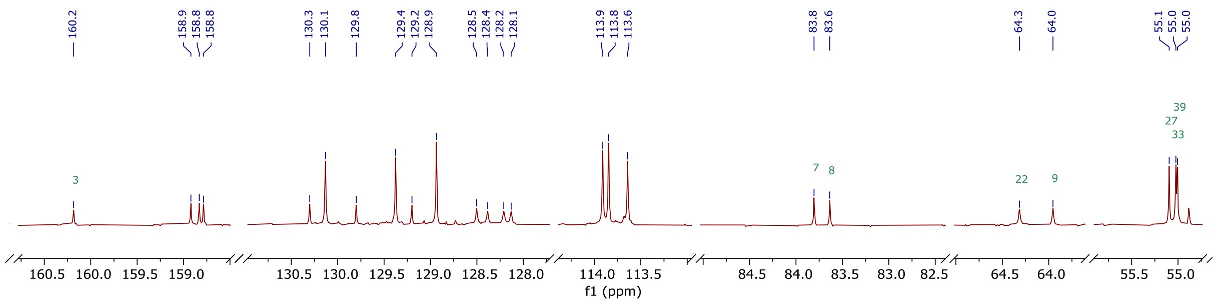

$^{1}H/^{1}H$ COSY

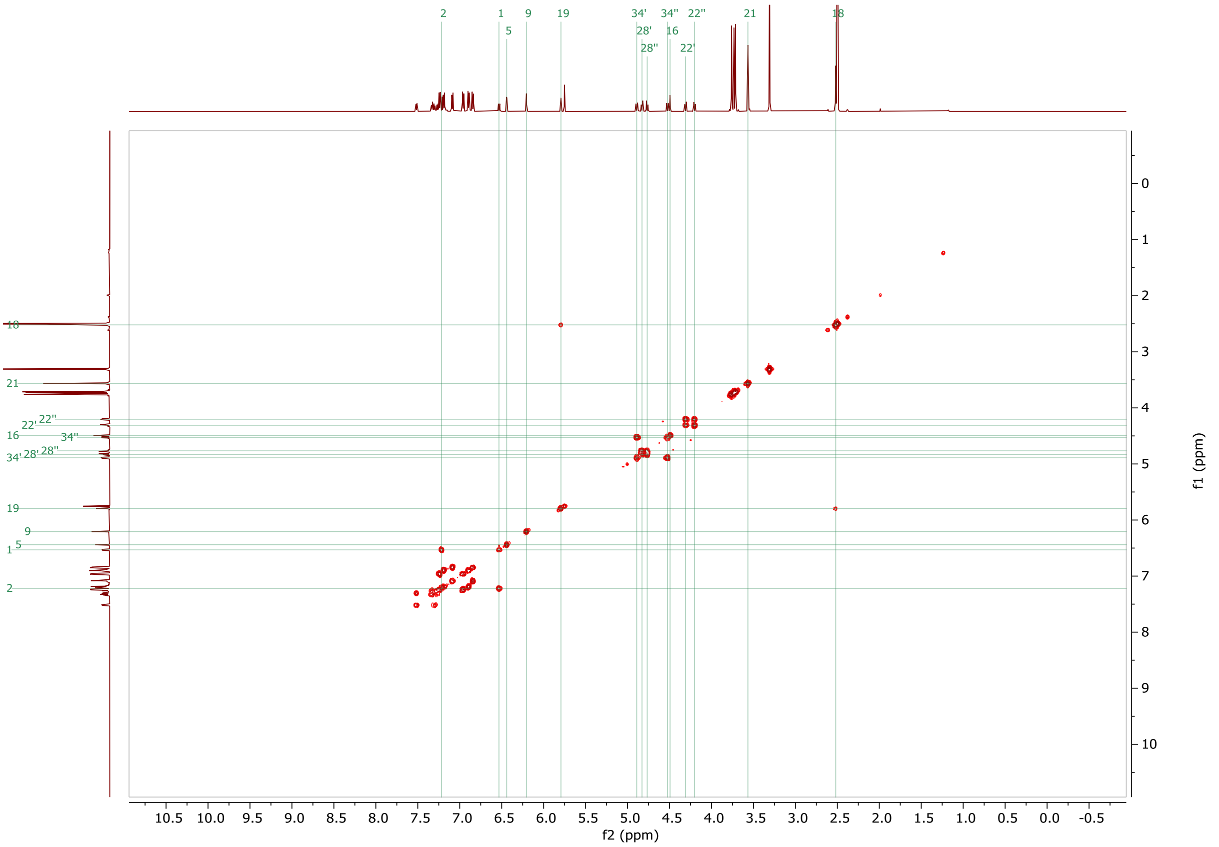

$^{1}H/^{13}C$ HSQC

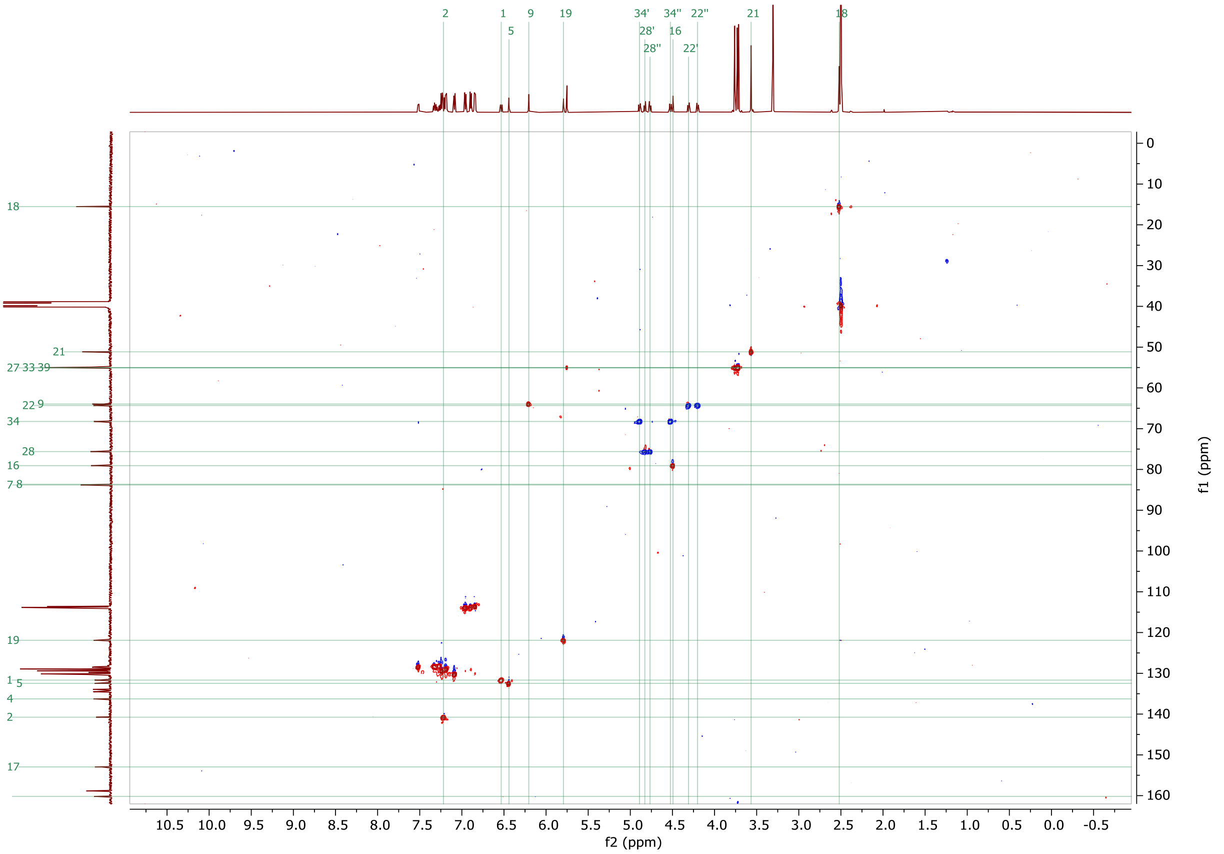

$^{1}H/^{13}C$ HMBC

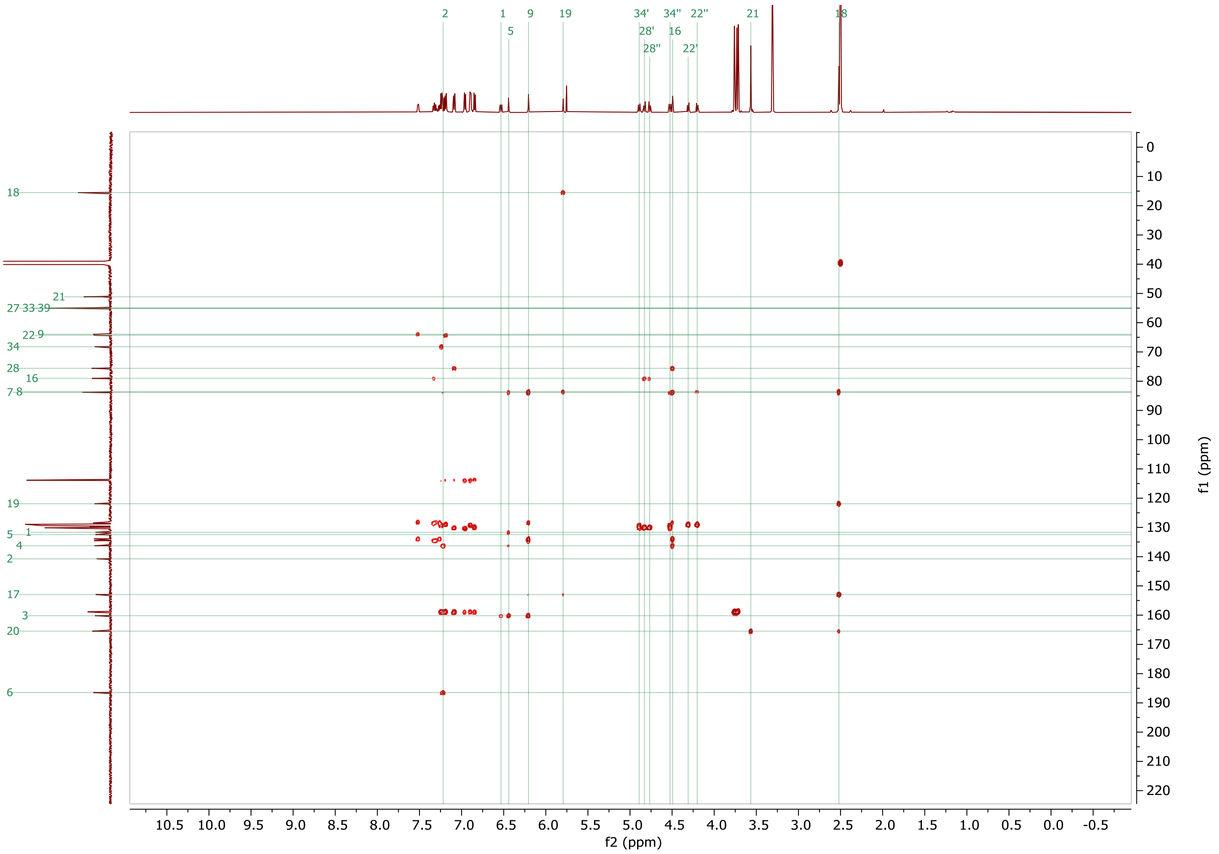
2
1
5
9
19
34'
28'
28''
34''
16
22''
22'
21
18
27 33 39
22 9
34
28
16
7 8
19
5
1
4
2
17
3
20
6
f2 (ppm)
f1 (ppm)

Methyl (*E*)-3-((8*S*,9*R*,14*S*,17*S*)-6-hydroxy-8,9,17-tris((4-methoxybenzyl)oxy)-5,16-dioxo-5,8,9,14,15,16-hexahydro-8,14-methanoanthra[1,2-b]benzo[*f*]azocin-17-yl)but-2-enoate 33

^{1}H NMR (500 MHz, DMSO-d_6)

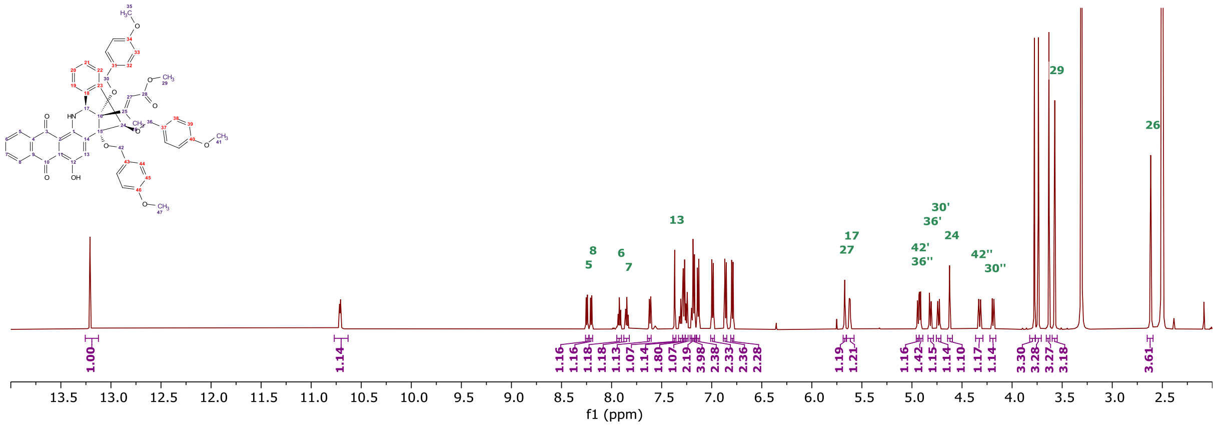

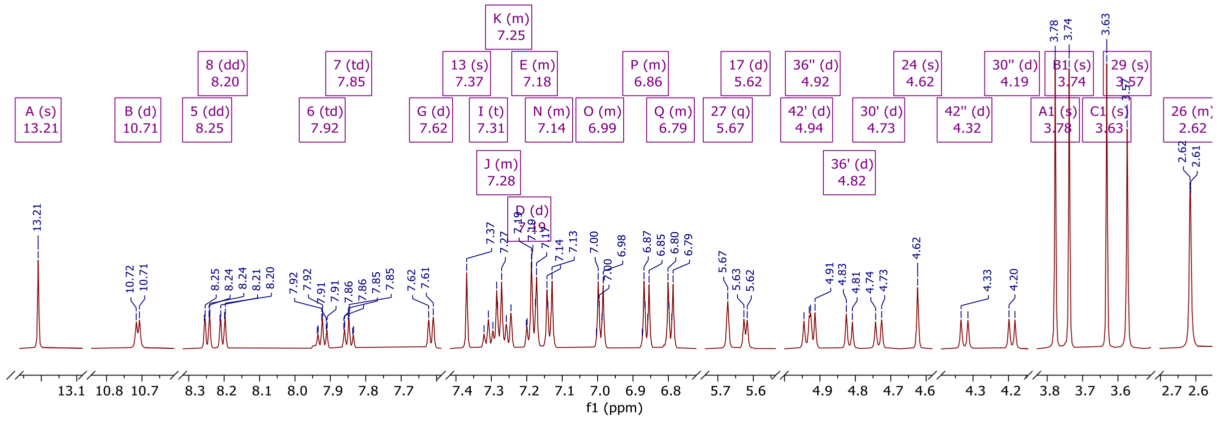

^{13}C NMR (126 MHz, DMSO-d_6)

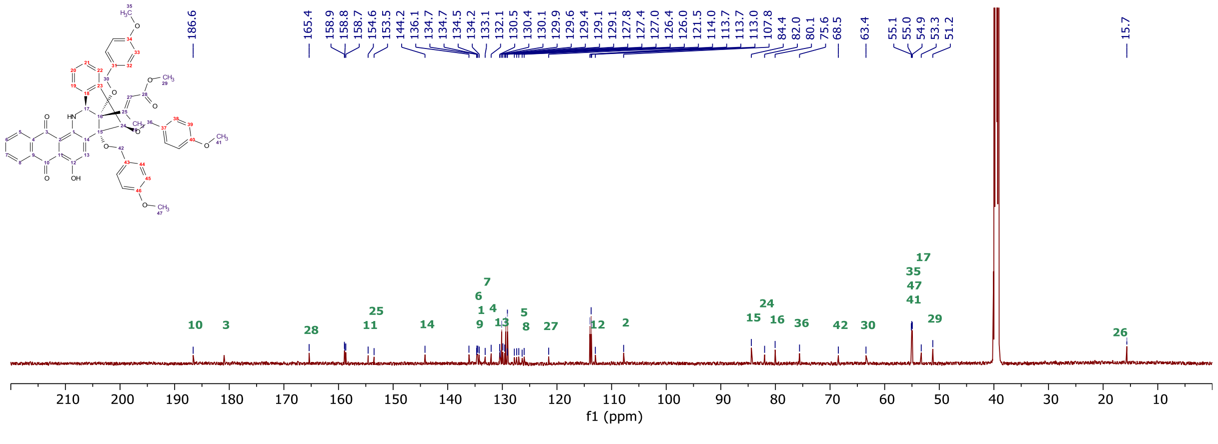

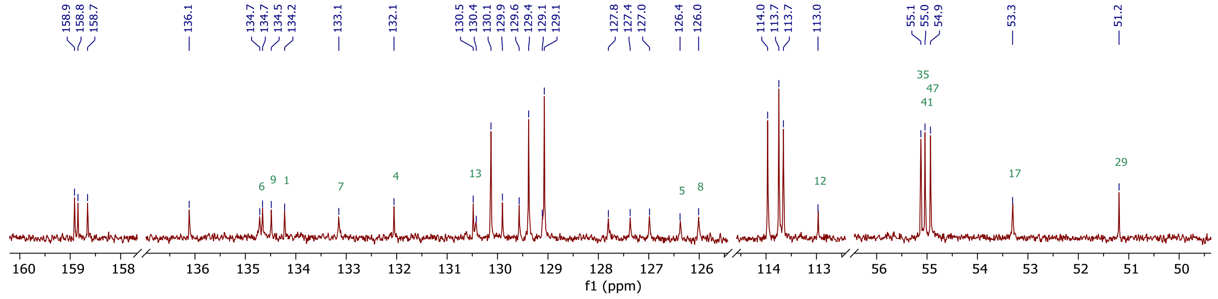

$^{1}H/^{1}H$ COSY

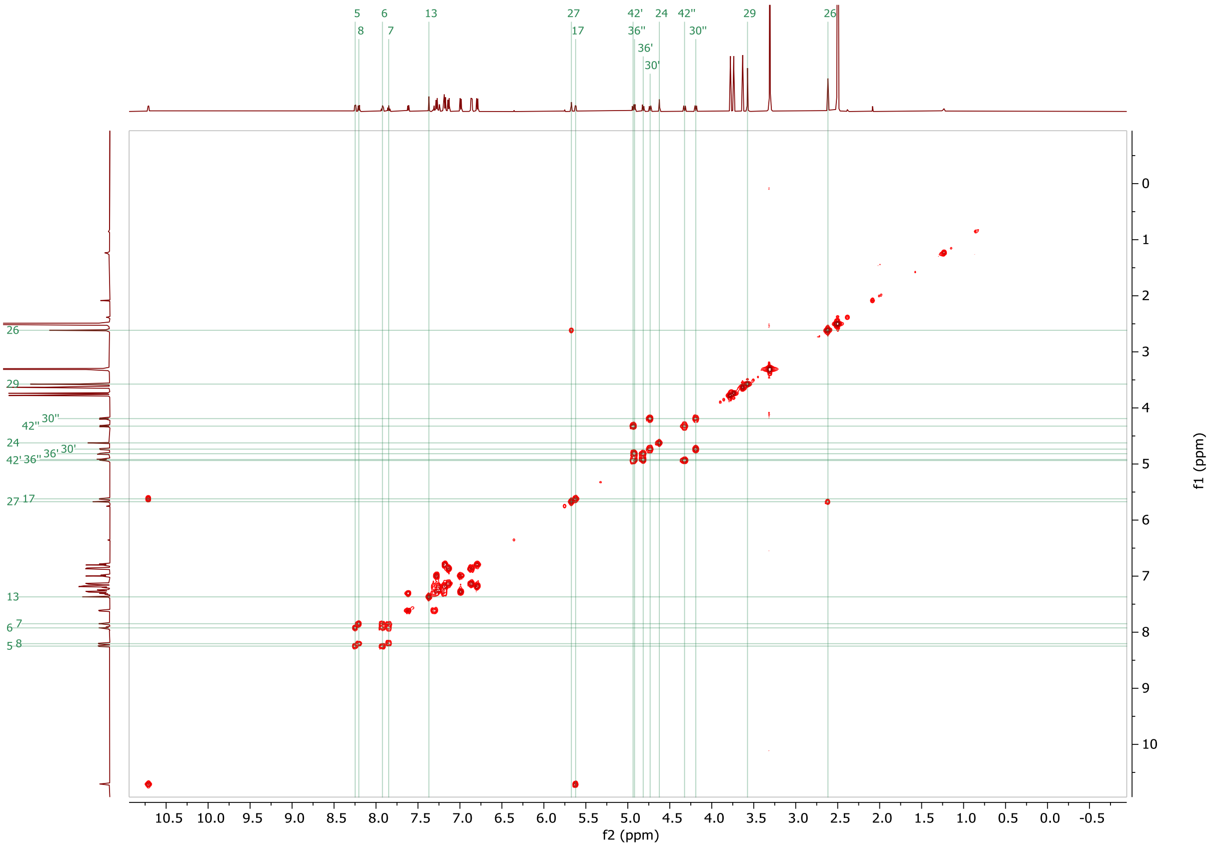

$^{1}H/^{13}C$ HSQC

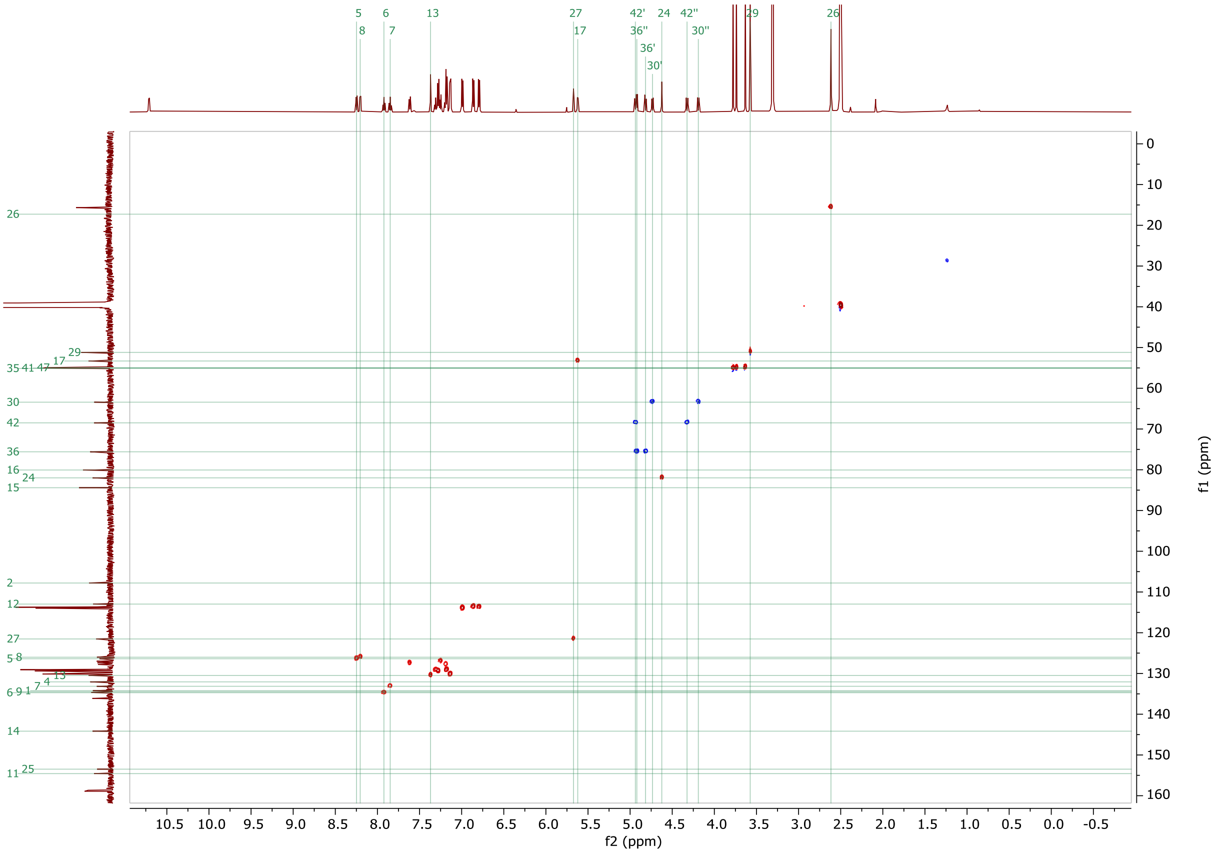

$^{1}H/^{13}C$ HMBC

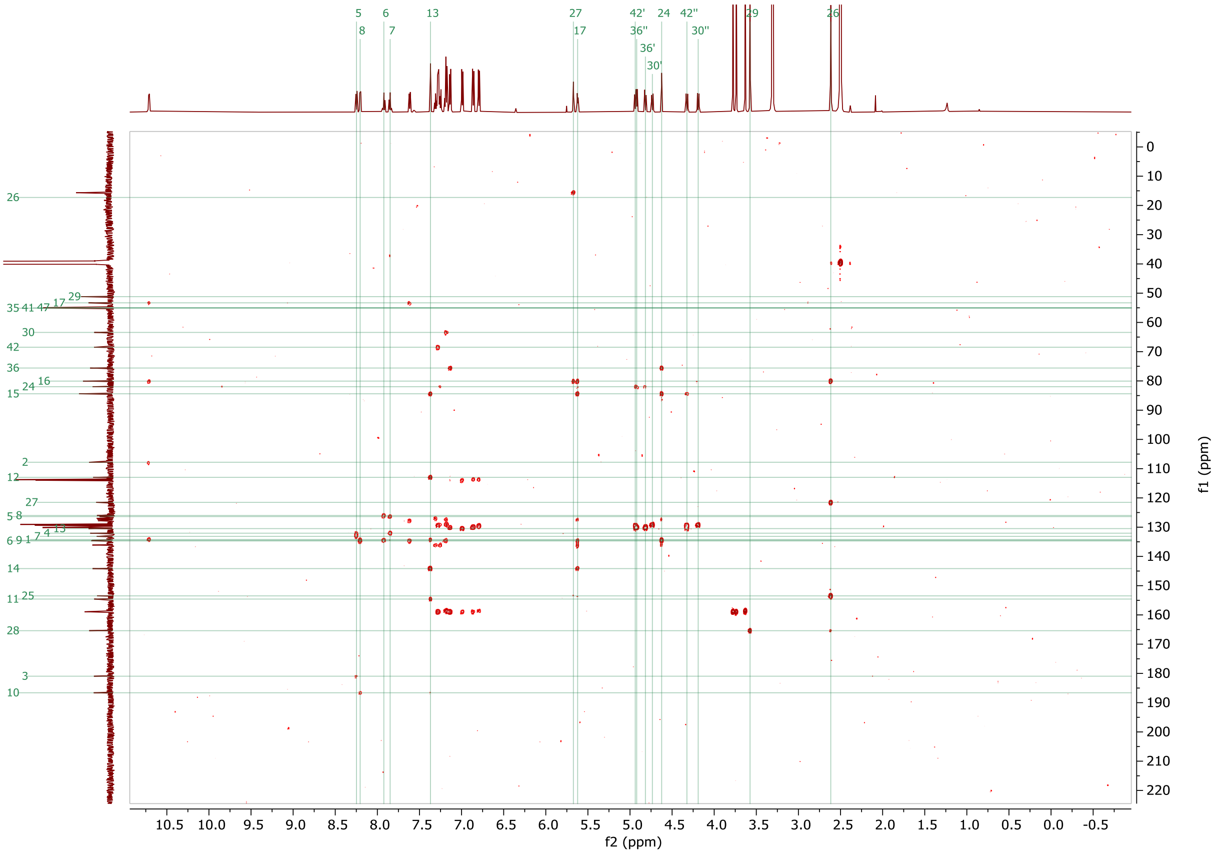

$^{1}H/^{1}H$ ROESY

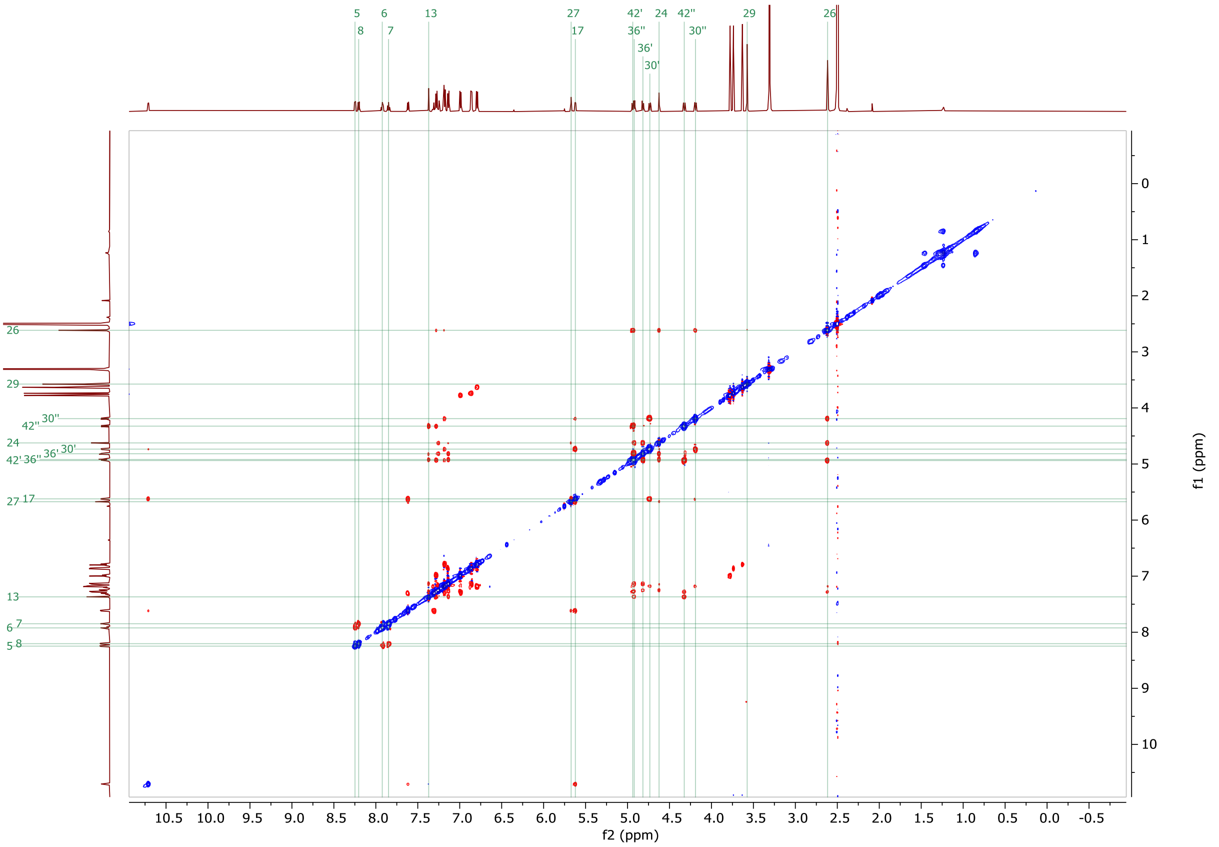

Methyl (*E*)-3-((8*S*,9*R*,14*S*,17*S*)-6,8,9,17-tetrahydroxy-5,16-dioxo-5,8,9,14,15,16-hexahydro-8,14-methanoanthra[1,2-b]benzo[*f*]azocin-17-yl)but-2-enoate – Sealutomicin C 3

^{1}H NMR (600 MHz, DMSO-d_6)

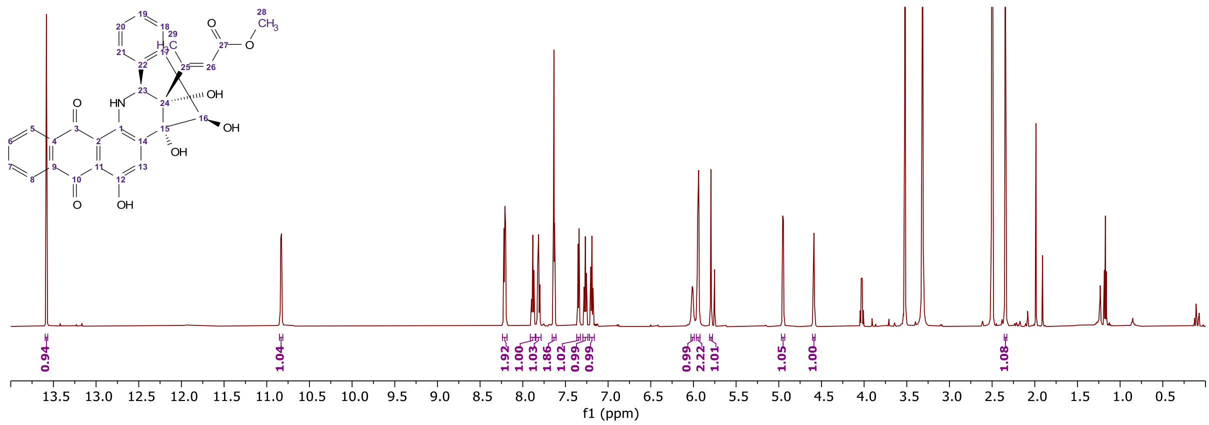

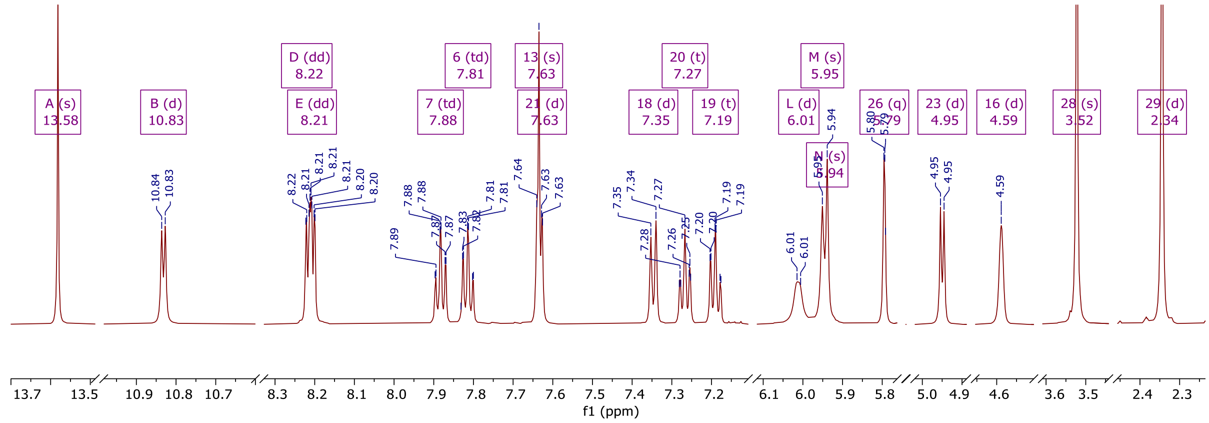

^{13}C NMR (150 MHz, DMSO-d_6)

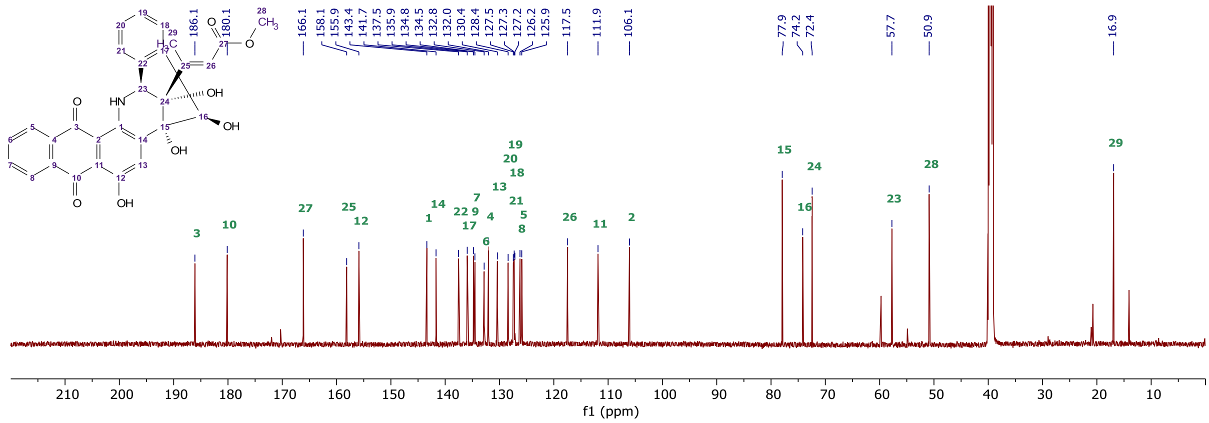

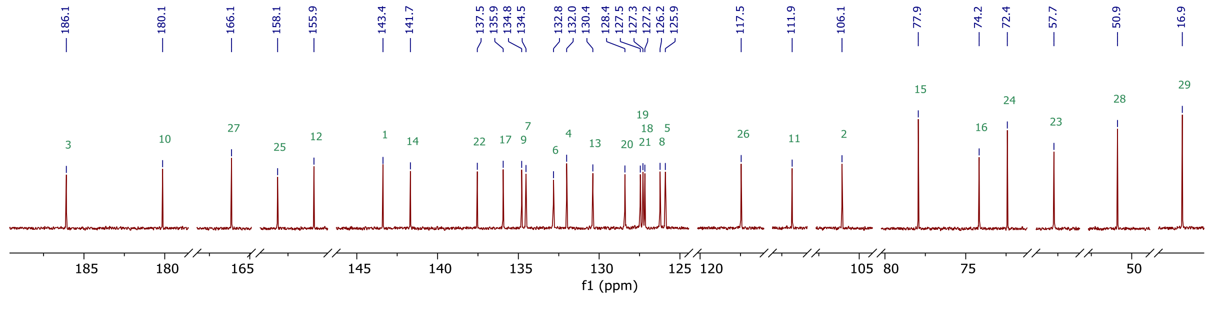

$^{1}H/^{1}H$ COSY (DMSO-d_6)

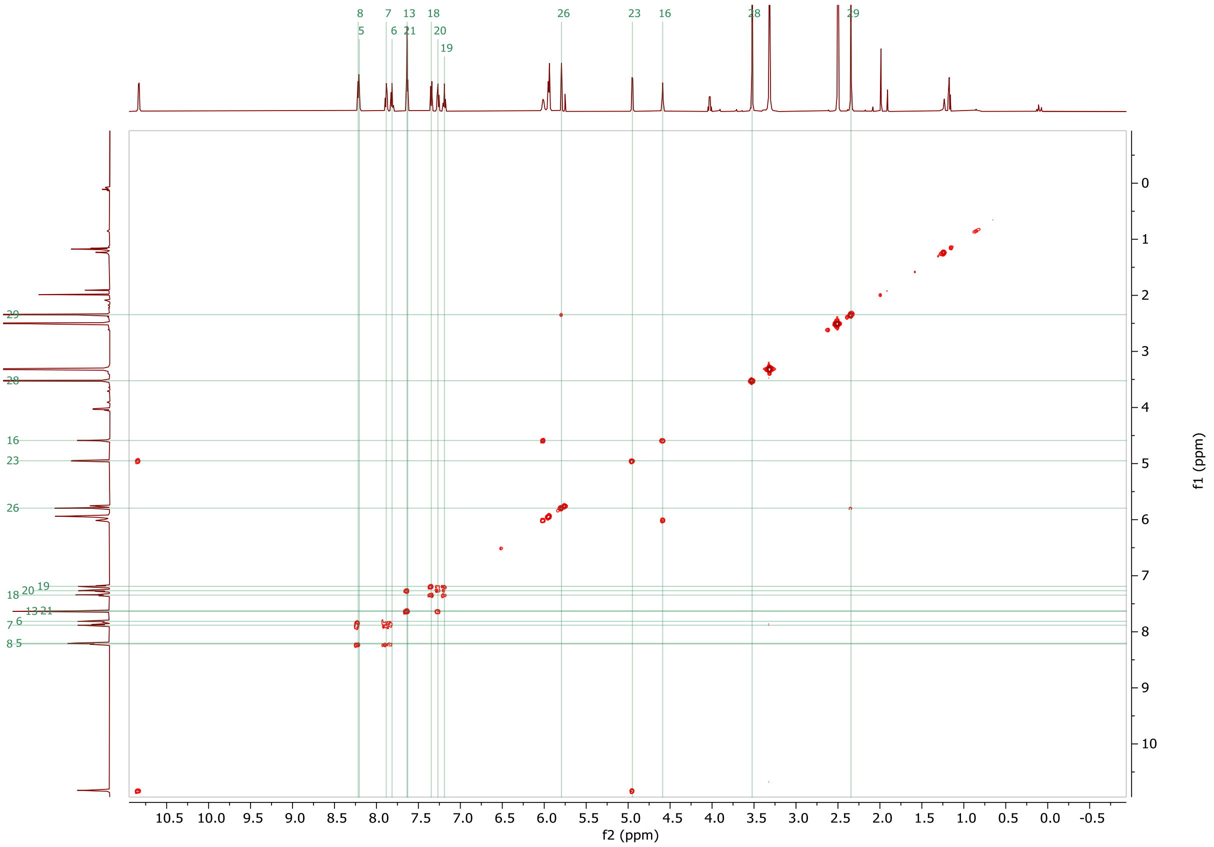

$^{1}H/^{13}C$ HSQC (DMSO-d_6)

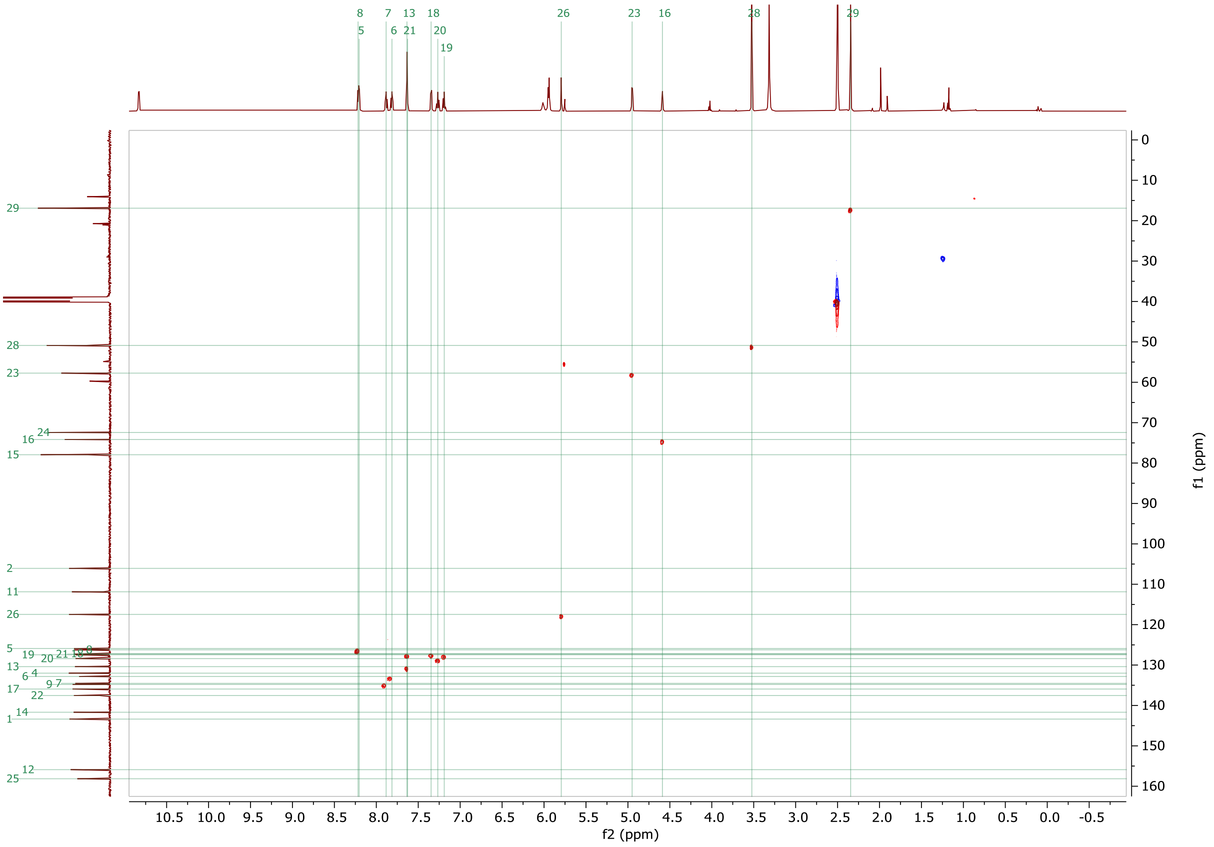

$^1H/^{13}C$ HMBC (DMSO-d_6)

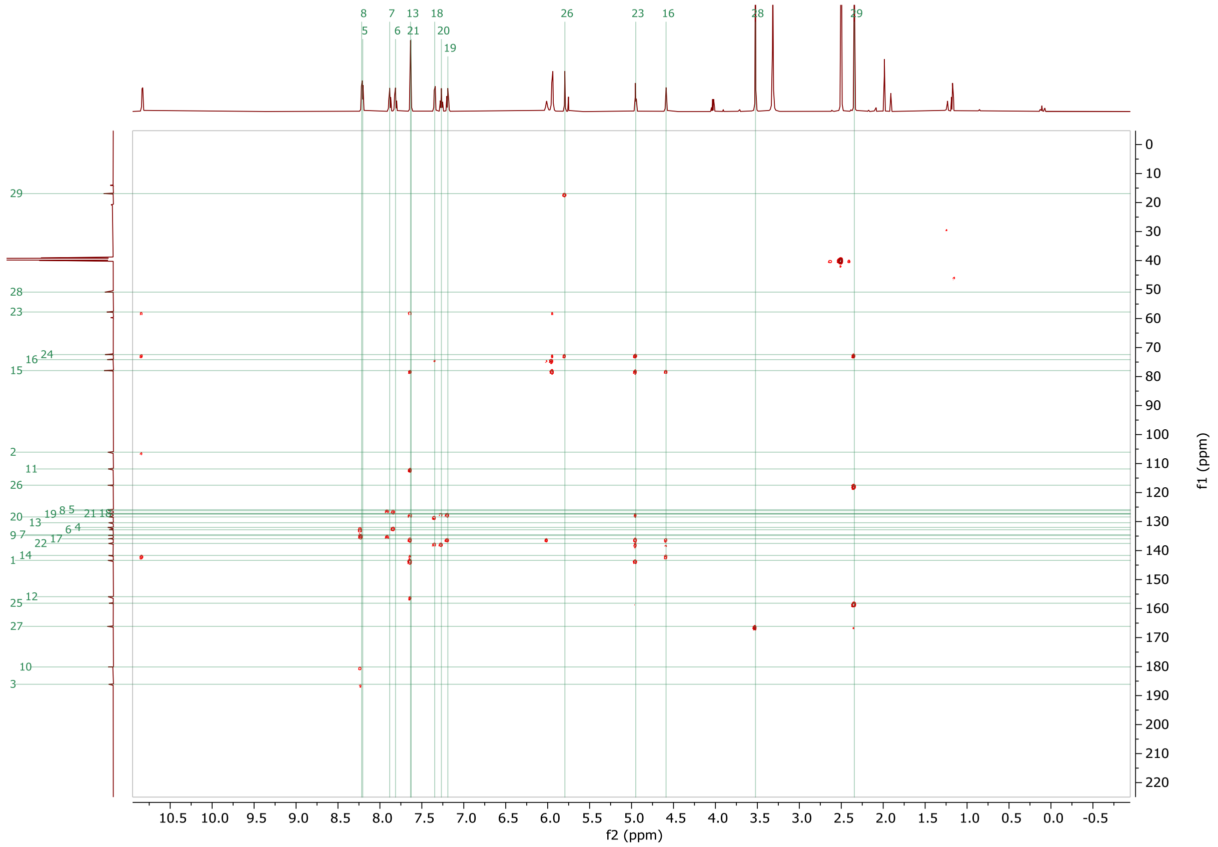

Methyl (*E*)-3-((8*S*,9*R*,14*S*,17*S*)-6,8,9,17-tetrahydroxy-5,16-dioxo-5,8,9,14,15,16-hexahydro-8,14-methanoanthra[1,2-b]benzo[*f*]azocin-17-yl)but-2-enoate – Sealutomicin C 3

^{1}H NMR (600 MHz, Acetone-d_6)

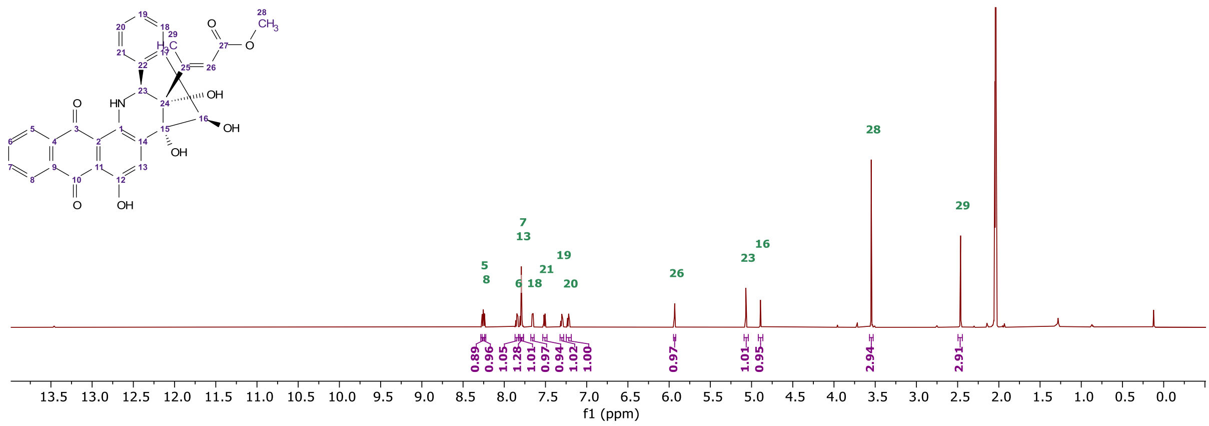

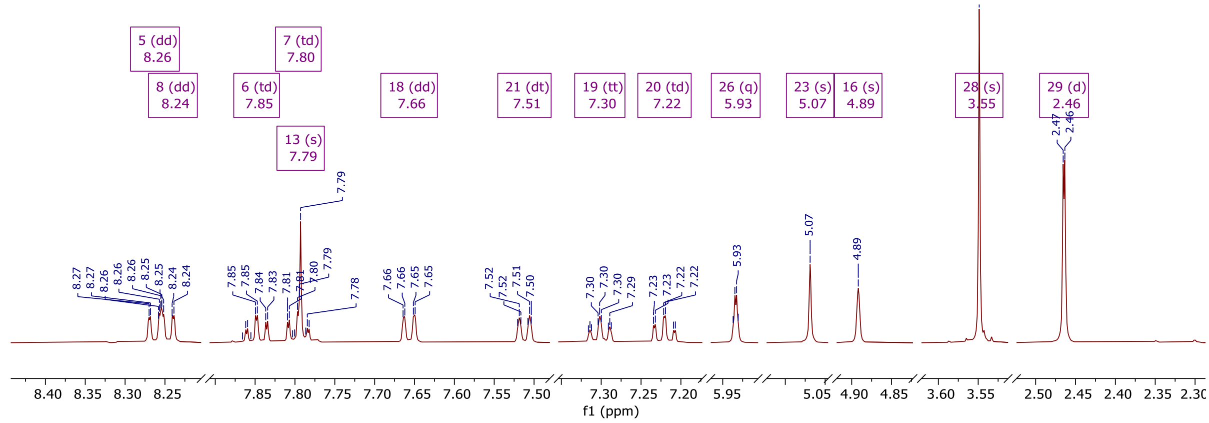

^{13}C NMR (150 MHz, Acetone-d_6)

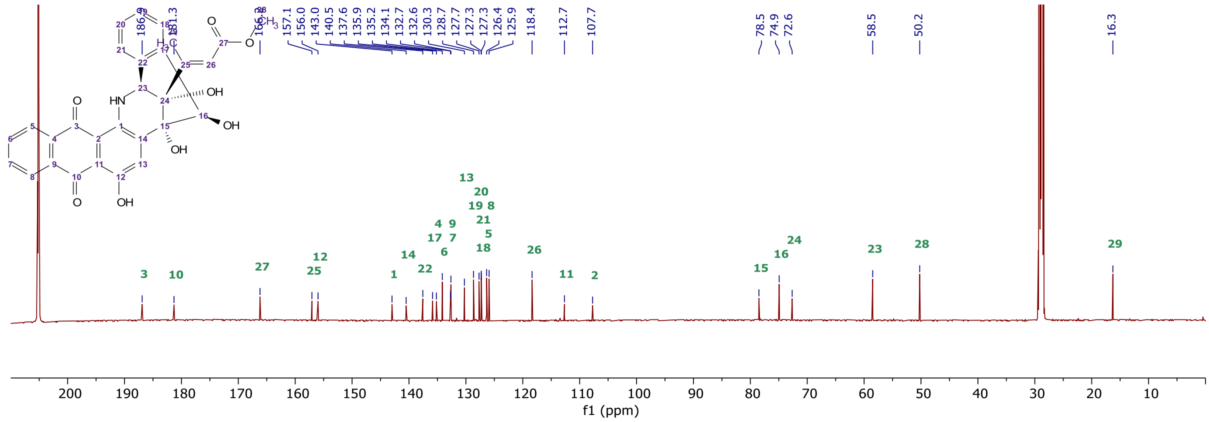

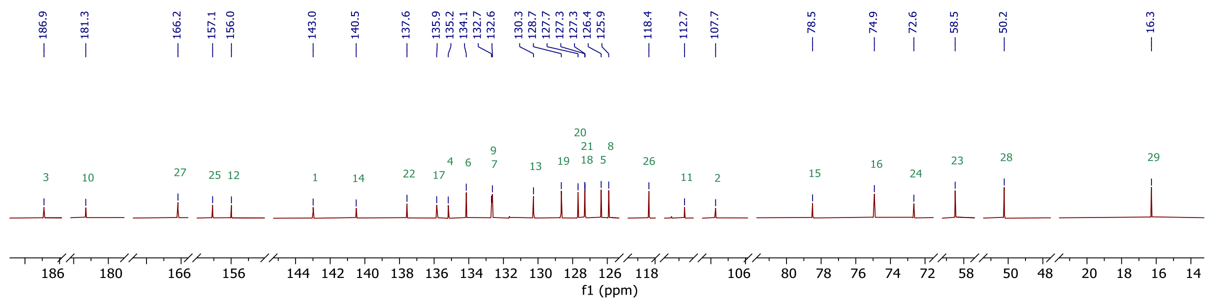

$^{1}H/^{1}H$ COSY (Acetone-d_6)

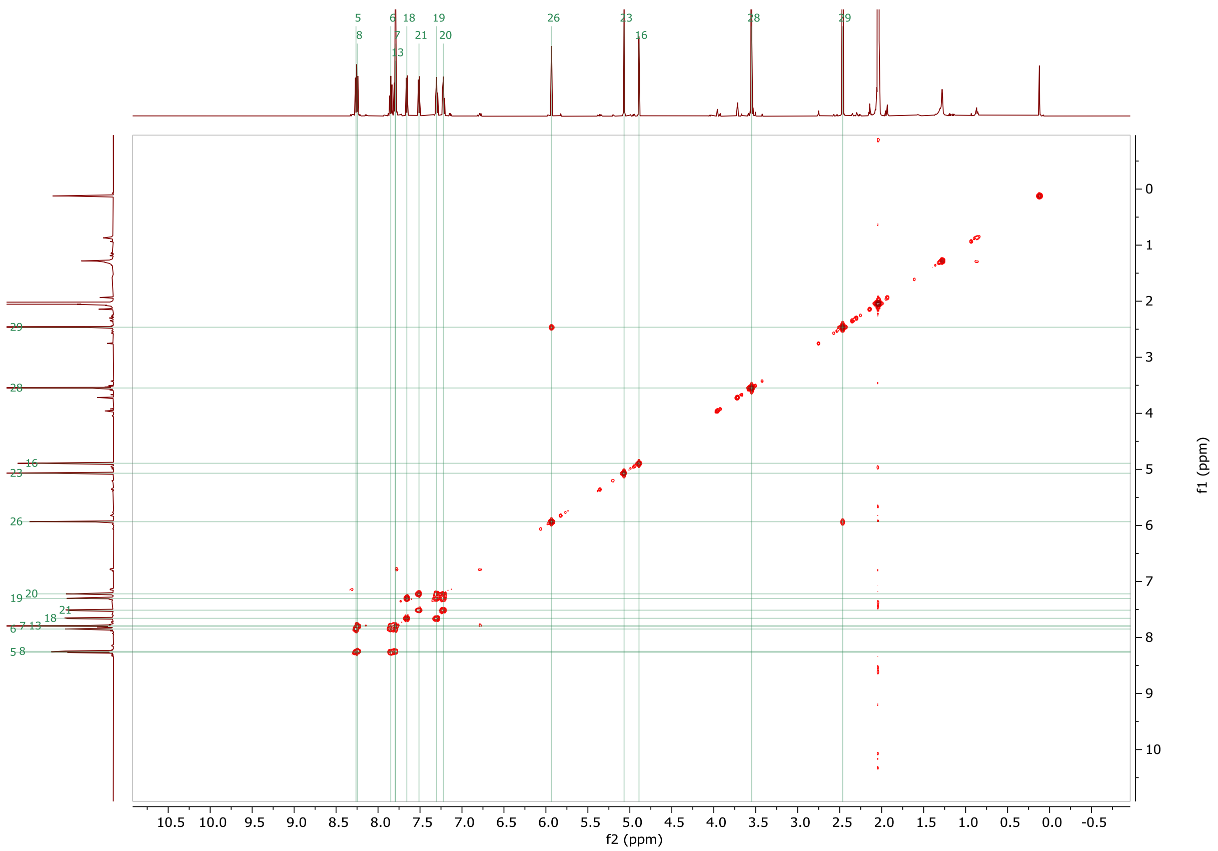

$^{1}H/^{13}C$ HSQC (Acetone-d_6)

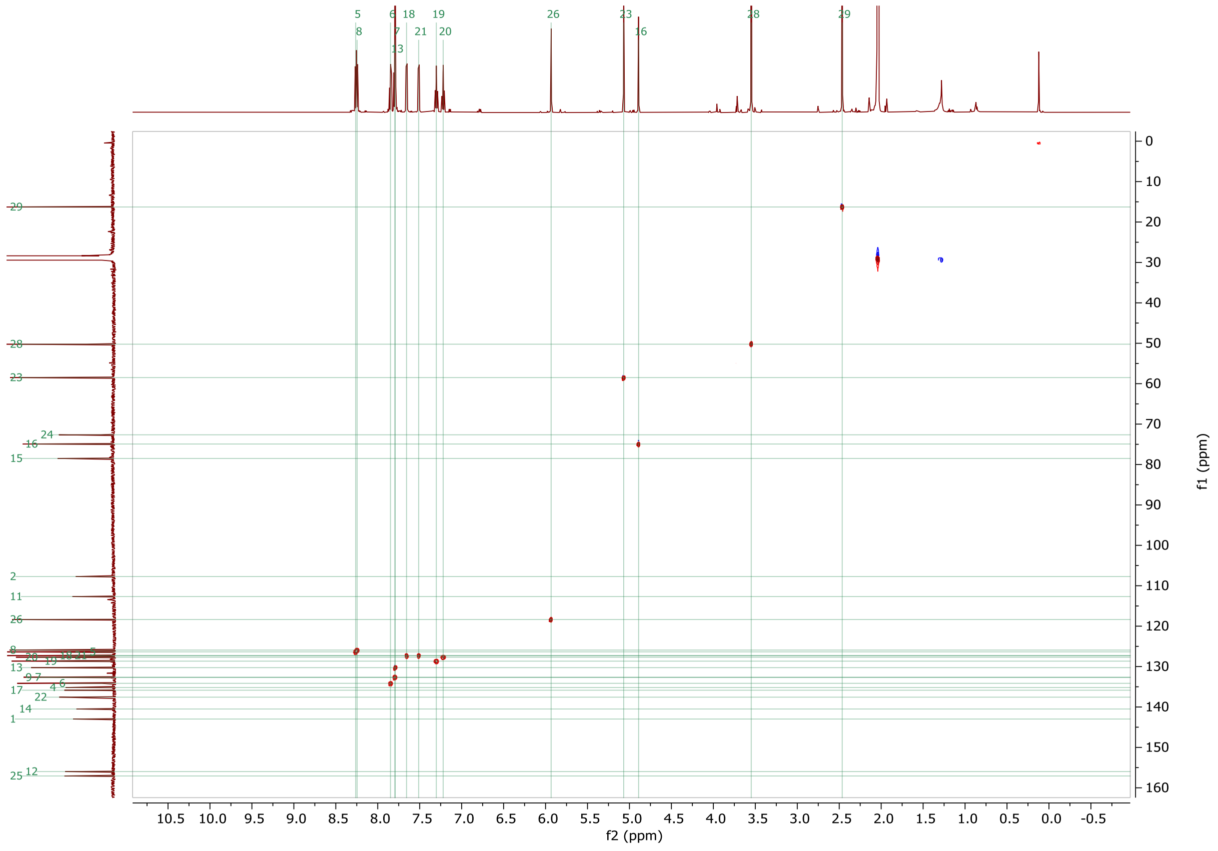

$^{1}H/^{13}C$ HMBC (Acetone-d_6)

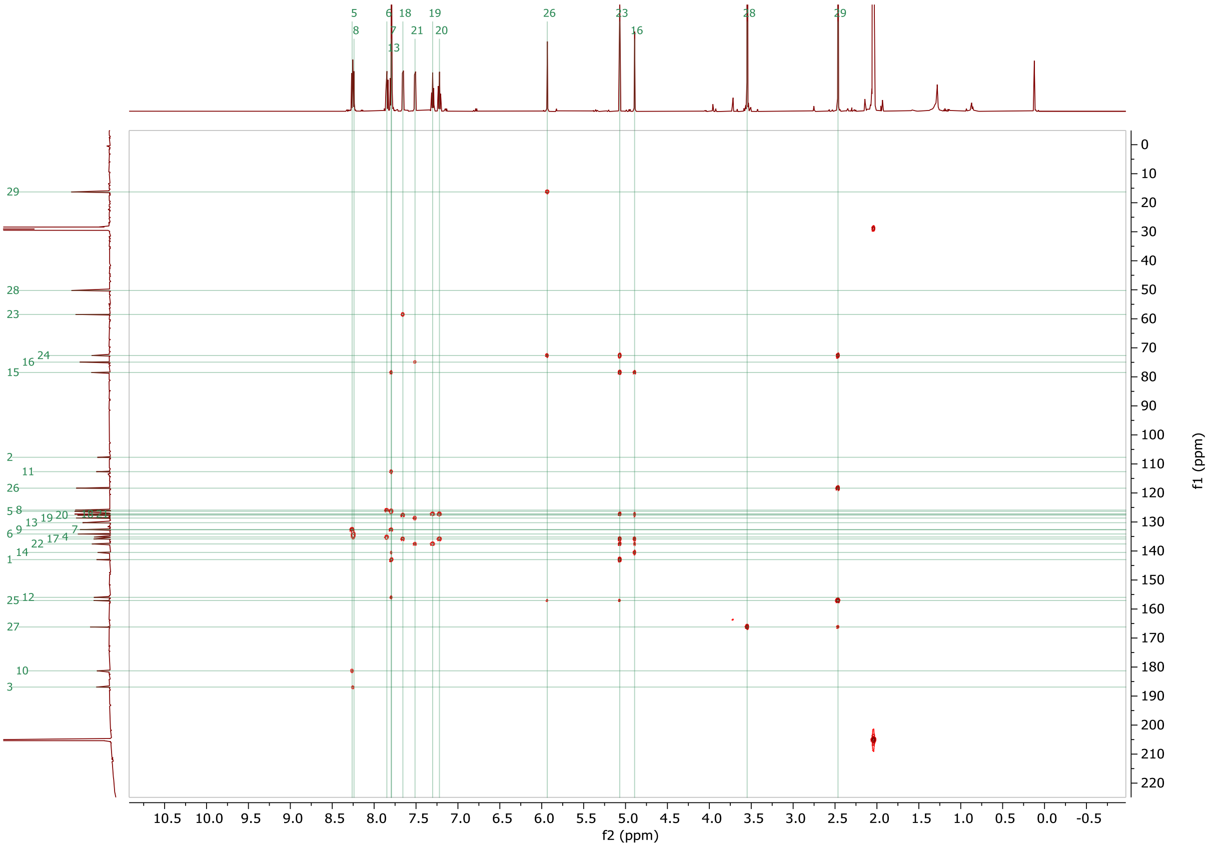

HPLC Data

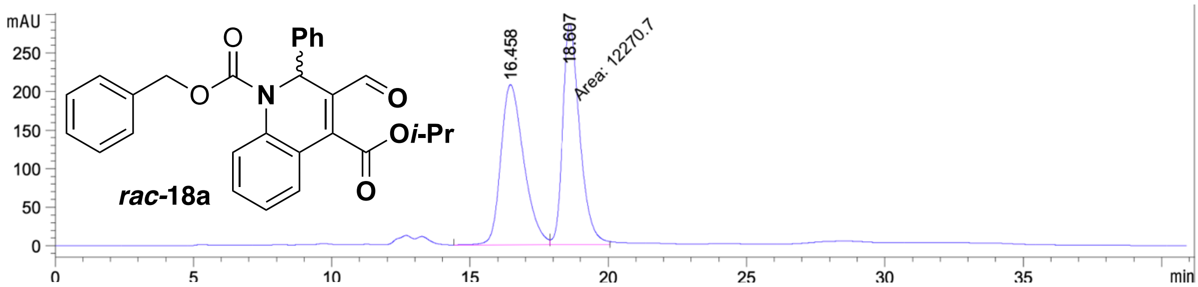

Peak #	RetTime [min]	Type	Width [min]	Area [mAU*s]	Height [mAU]	Area %
1	16.458	BV	0.8838	1.19935e4	207.65910	49.4286
2	18.607	MF	0.7141	1.22707e4	286.37646	50.5714

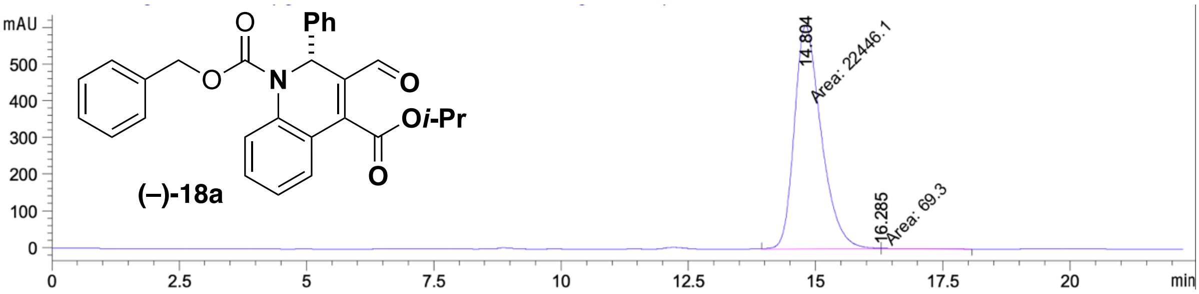

Peak #	RetTime [min]	Type	Width [min]	Area [mAU*s]	Height [mAU]	Area %
1	14.804	MM	0.6149	2.24461e4	608.42542	99.6922
2	16.285	MM	0.9932	69.30003	8.32548e-1	0.3078

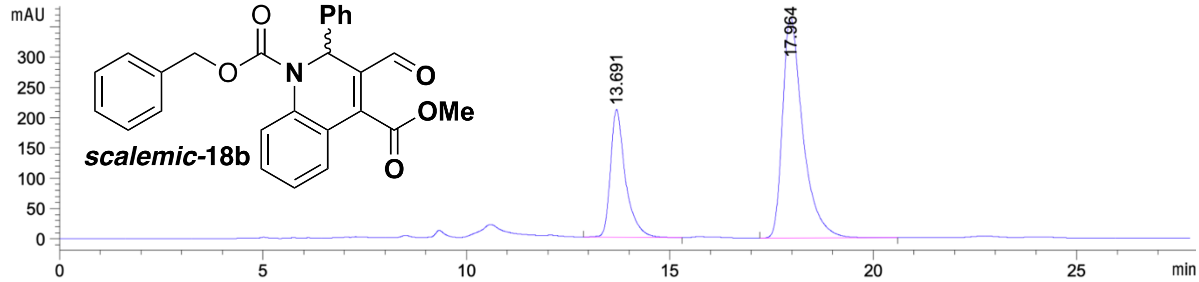

Peak #	RetTime [min]	Type	Width [min]	Area [mAU*s]	Height [mAU]	Area %
1	13.691	BB	0.3802	5428.18945	210.92751	29.9585
2	17.964	BB	0.5174	1.26908e4	365.21381	70.0415

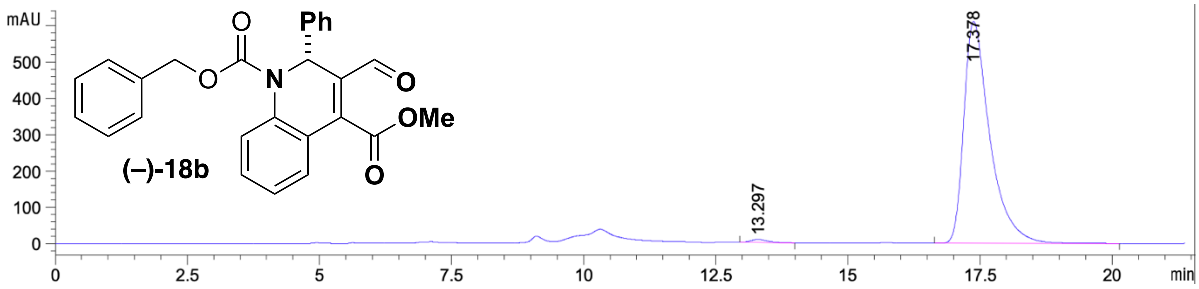

Peak #	RetTime [min]	Type	Width [min]	Area [mAU*s]	Height [mAU]	Area %
1	13.297	BB	0.3351	180.63298	8.19638	0.8547
2	17.378	BB	0.5120	2.09536e4	611.11902	99.1453

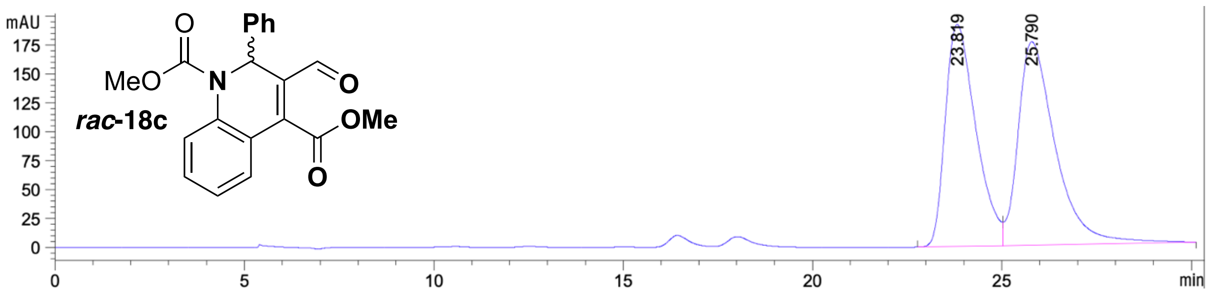

Peak #	RetTime [min]	Type	Width [min]	Area [mAU*s]	Height [mAU]	Area %
1	23.819	BV	0.8565	1.07775e4	192.10706	47.2008
2	25.790	VBA	1.0290	1.20558e4	176.04239	52.7992

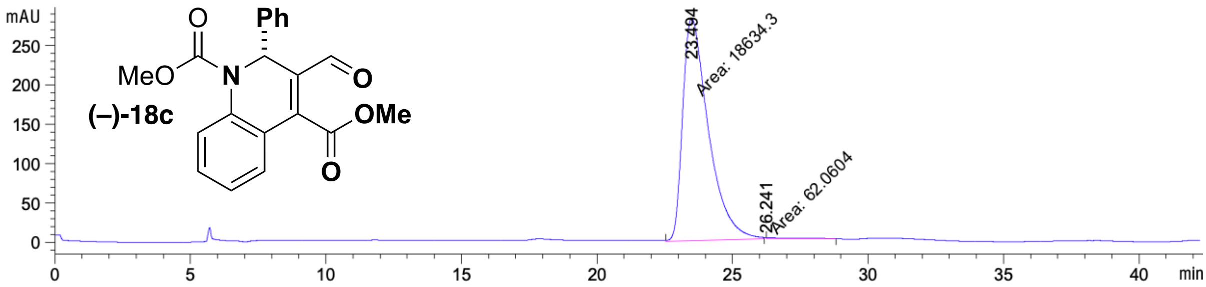

Peak #	RetTime [min]	Type	Width [min]	Area [mAU*s]	Height [mAU]	Area %
1	23.494	MM T	1.0987	1.86343e4	282.68207	99.6681
2	26.241	MM	0.6335	62.06044	1.14675	0.3319

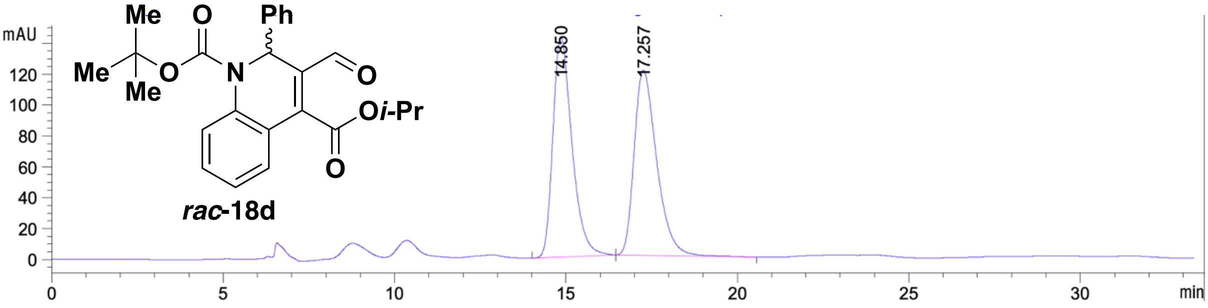

Peak #	RetTime [min]	Type	Width [min]	Area [mAU*s]	Height [mAU]	Area %
1	14.850	BB	0.5882	5511.17529	142.81035	50.0966
2	17.257	BB	0.6999	5489.91406	119.97408	49.9034

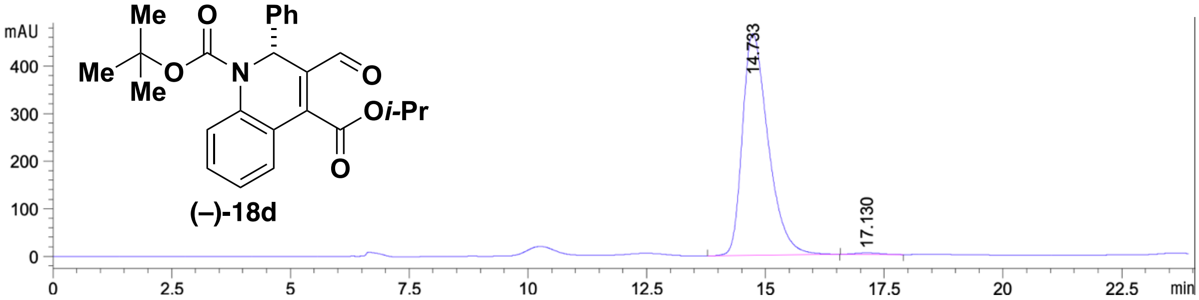

Peak #	RetTime [min]	Type	Width [min]	Area [mAU*s]	Height [mAU]	Area %
1	14.733	BB	0.5756	1.75591e4	465.96680	99.3540
2	17.130	BB	0.5459	114.16874	3.15676	0.6460

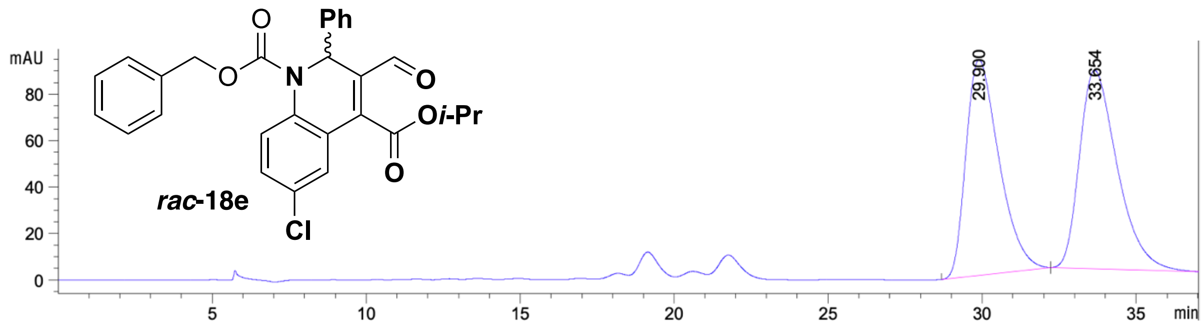

Peak #	RetTime [min]	Type	Width [min]	Area [mAU*s]	Height [mAU]	Area %
1	29.900	BB	1.1303	6933.05029	92.94095	48.7469
2	33.654	BBA	1.3024	7289.50928	85.52449	51.2531

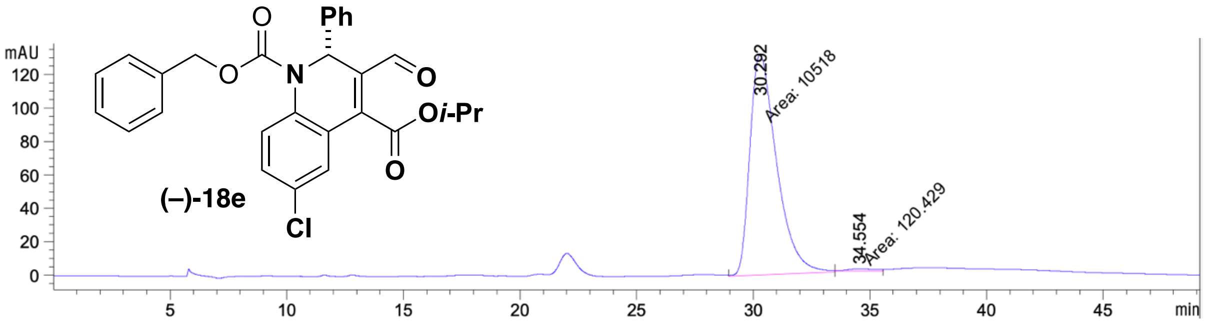

Peak #	RetTime [min]	Type	Width [min]	Area [mAU*s]	Height [mAU]	Area %
1	30.292	MM	1.3292	1.05180e4	131.88789	98.8680
2	34.554	MM	1.4507	120.42887	1.38359	1.1320

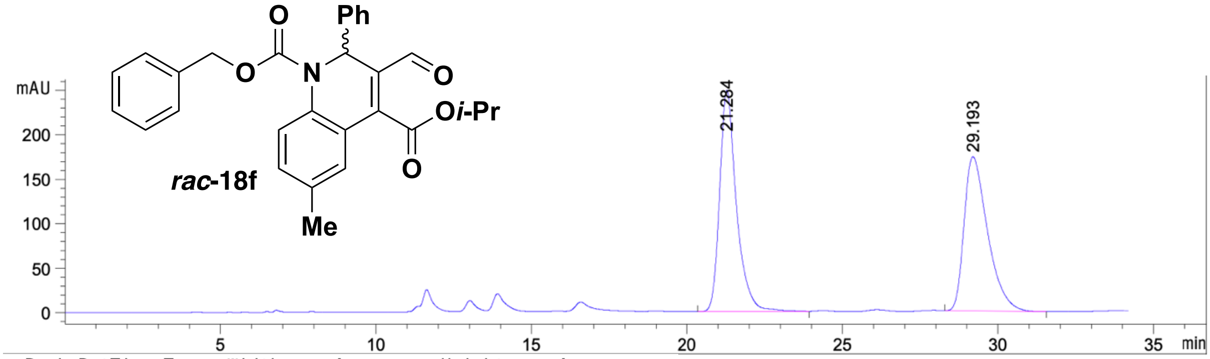

Peak #	RetTime [min]	Type	Width [min]	Area [mAU*s]	Height [mAU]	Area %
1	21.284	BB	0.5616	9376.41113	248.85080	50.6561
2	29.193	BB	0.8025	9133.53320	173.40965	49.3439

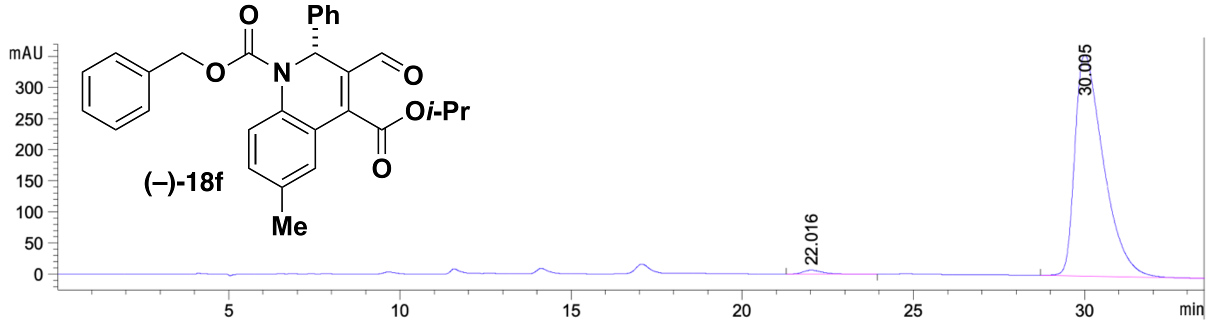

Peak #	RetTime [min]	Type	Width [min]	Area [mAU*s]	Height [mAU]	Area %
1	22.016	BB	0.5797	260.22263	6.57626	1.2405
2	30.005	BBA	0.8835	2.07167e4	355.67810	98.7595

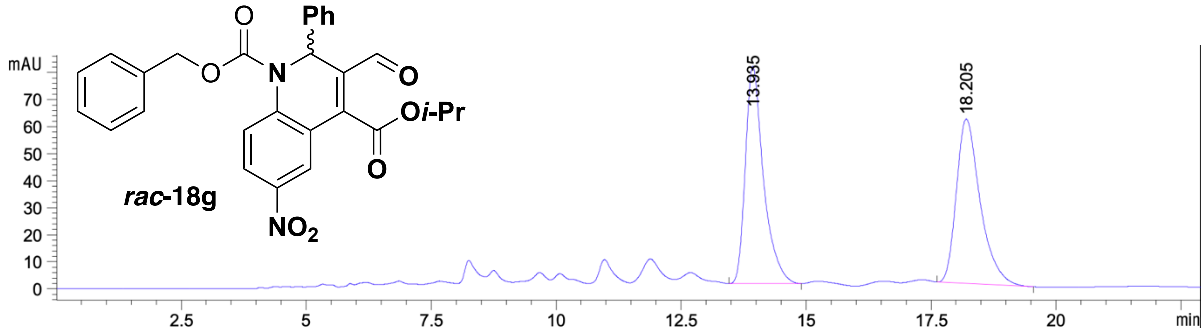

Peak #	RetTime [min]	Type	Width [min]	Area [mAU*s]	Height [mAU]	Area %
1	13.935	BB	0.3765	2032.07092	80.51197	50.0987
2	18.205	BB	0.5020	2024.06689	60.86510	49.9013

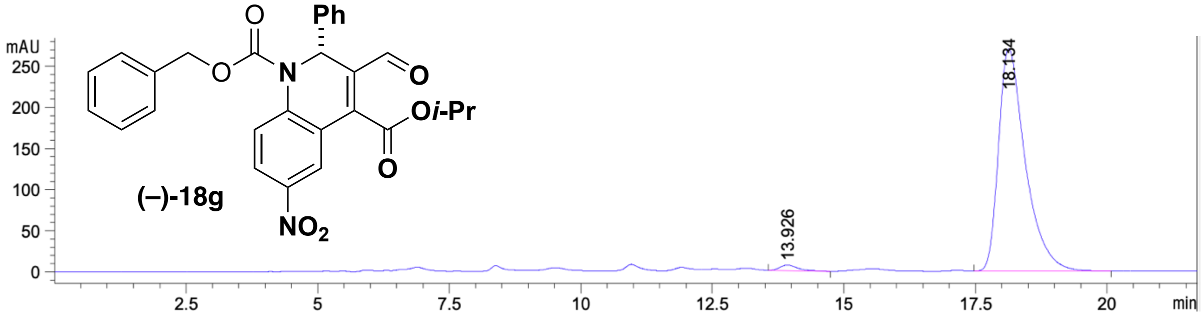

Peak #	RetTime [min]	Type	Width [min]	Area [mAU*s]	Height [mAU]	Area %
1	13.926	BB	0.3548	160.25716	6.85480	1.6496
2	18.134	BB	0.5309	9554.56445	269.94986	98.3504

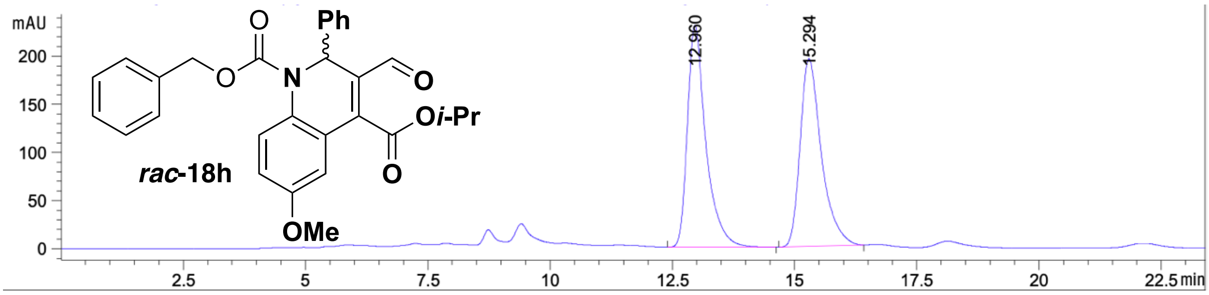

Peak #	RetTime [min]	Type	Width [min]	Area [mAU*s]	Height [mAU]	Area %
1	12.960	BB	0.3899	6047.50586	230.67813	50.6508
2	15.294	BB	0.4546	5892.10010	194.93600	49.3492

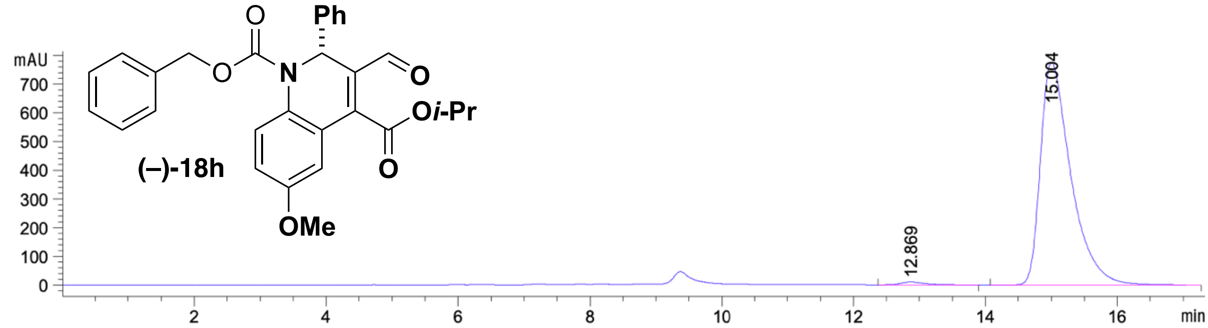

Peak #	RetTime [min]	Type	Width [min]	Area [mAU*s]	Height [mAU]	Area %
1	12.869	BB	0.3792	253.72581	9.89353	1.0114
2	15.004	BB	0.4830	2.48321e4	776.68793	98.9886

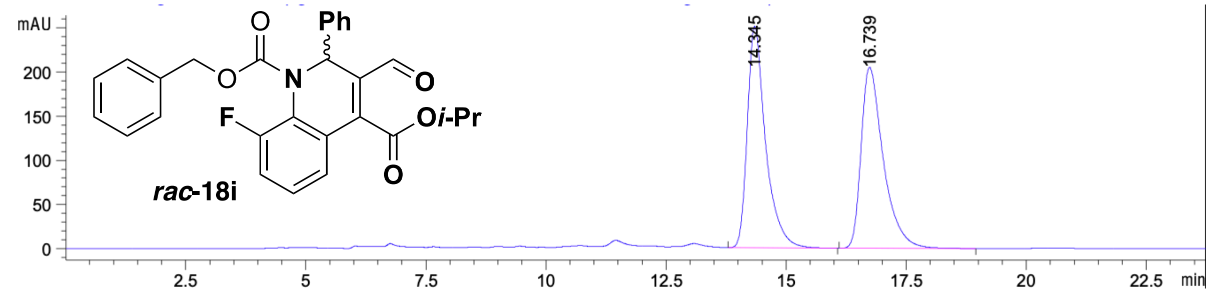

Peak #	RetTime [min]	Type	Width [min]	Area [mAU*s]	Height [mAU]	Area %
1	14.345	BB	0.3930	6644.85449	250.85956	50.2295
2	16.739	BB	0.4826	6584.13379	205.04614	49.7705

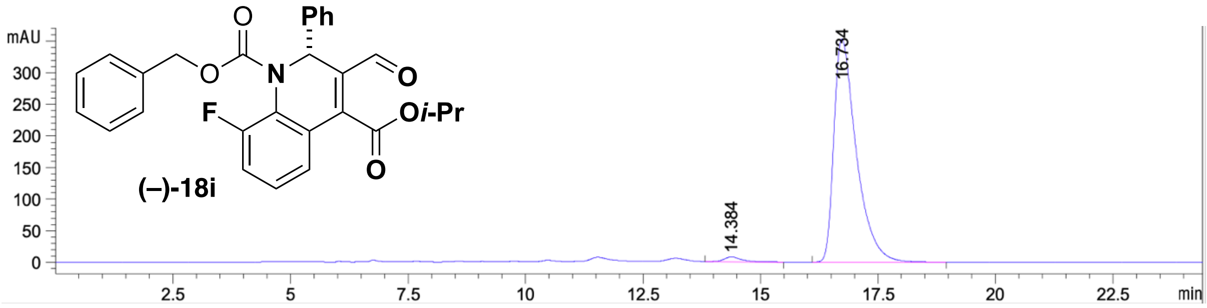

Peak #	RetTime [min]	Type	Width [min]	Area [mAU*s]	Height [mAU]	Area %
1	14.384	BB	0.3780	192.21030	7.62855	1.6100
2	16.734	BB	0.4999	1.17463e4	353.30325	98.3900

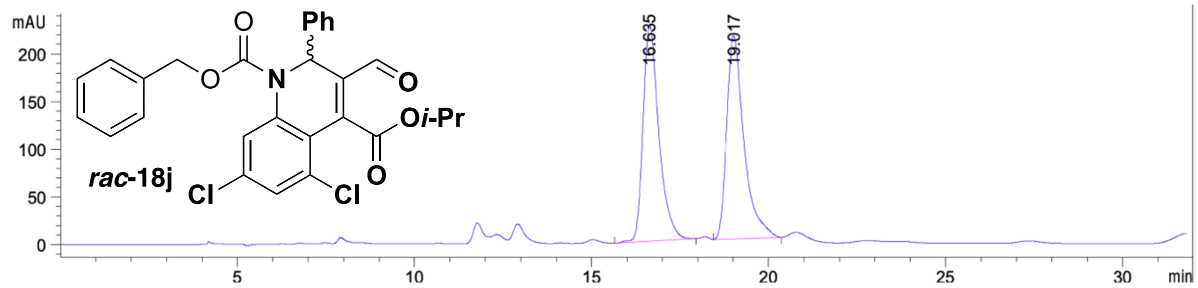

Peak #	RetTime [min]	Type	Width [min]	Area [mAU*s]	Height [mAU]	Area %
1	16.635	BB	0.4688	7081.02441	227.68582	49.3453
2	19.017	BB	0.5104	7268.92725	212.83656	50.6547

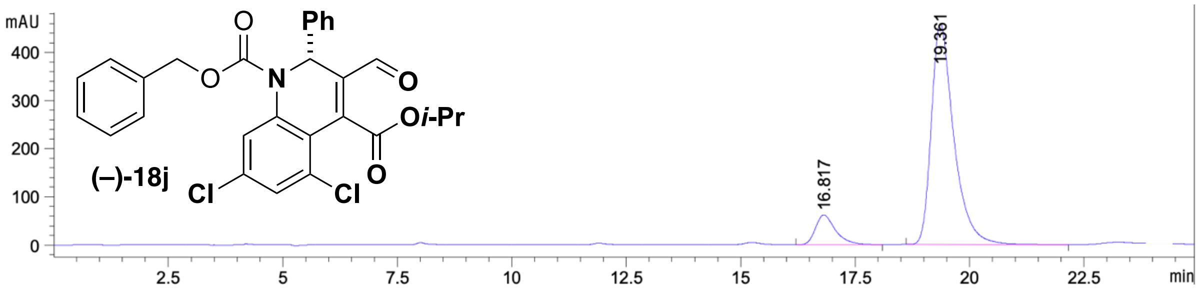

Peak #	RetTime [min]	Type	Width [min]	Area [mAU*s]	Height [mAU]	Area %
1	16.817	BB	0.4572	1886.89575	61.97211	10.7355
2	19.361	BB	0.5129	1.56894e4	458.92136	89.2645

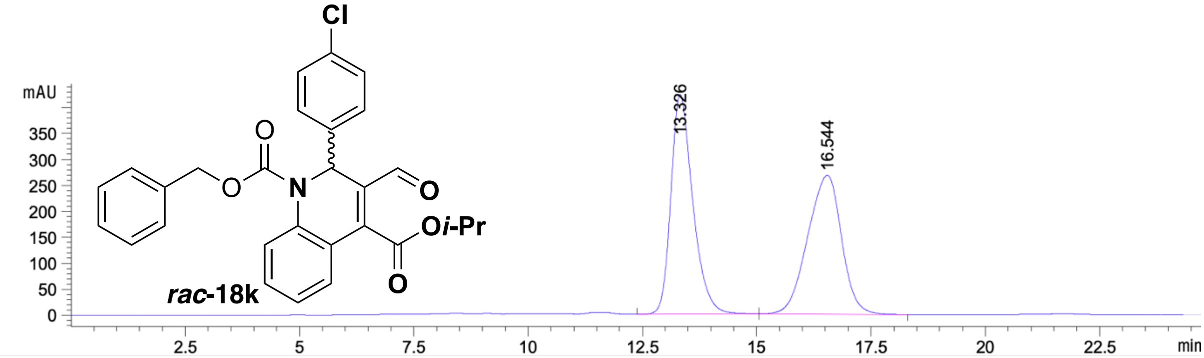

Peak #	RetTime [min]	Type	Width [min]	Area [mAU*s]	Height [mAU]	Area %
1	13.326	BB	0.5052	1.41146e4	423.10132	50.6610
2	16.544	BB	0.8175	1.37463e4	267.50024	49.3390

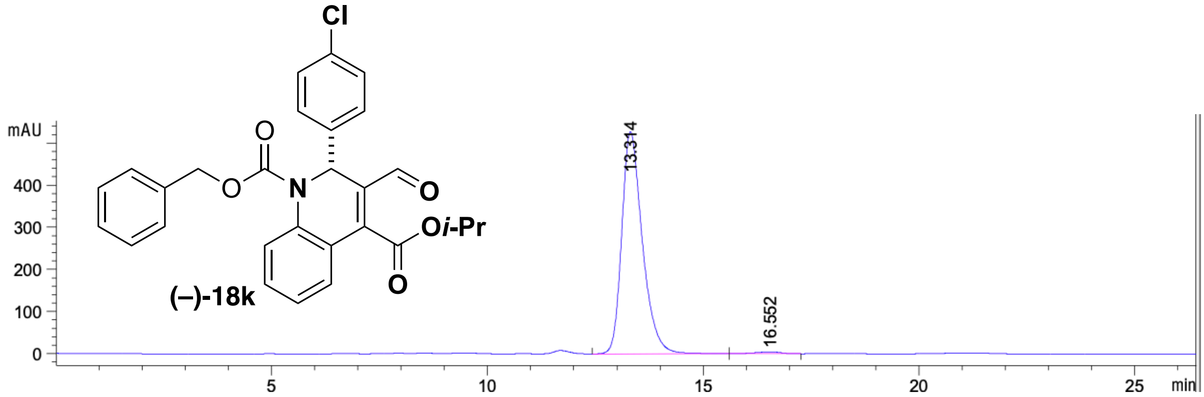

Peak #	RetTime [min]	Type	Width [min]	Area [mAU*s]	Height [mAU]	Area %
1	13.314	BB	0.5007	1.74899e4	527.67499	99.0058
2	16.552	BB	0.5804	175.63057	4.48961	0.9942

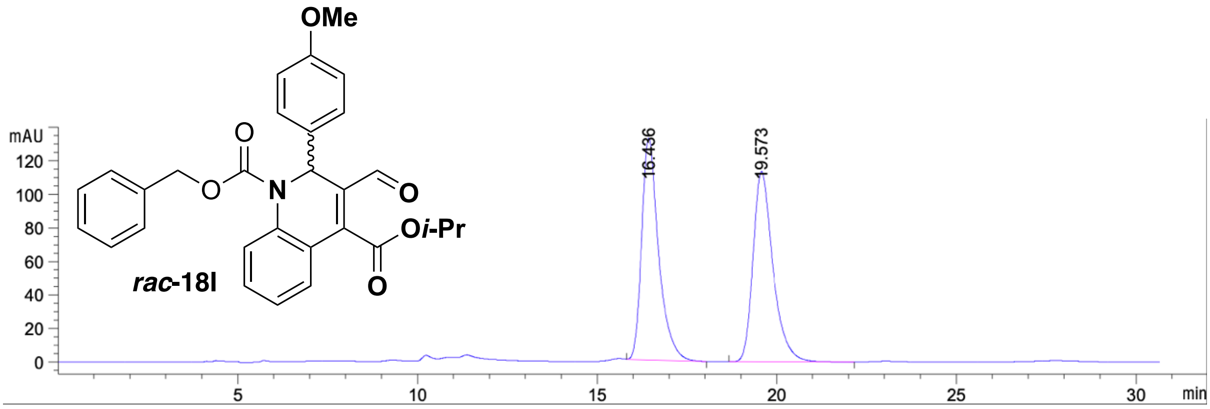

Peak #	RetTime [min]	Type	Width [min]	Area [mAU*s]	Height [mAU]	Area %
1	16.436	BB	0.4882	4301.23389	131.97121	49.4618
2	19.573	BB	0.5842	4394.83838	113.37889	50.5382

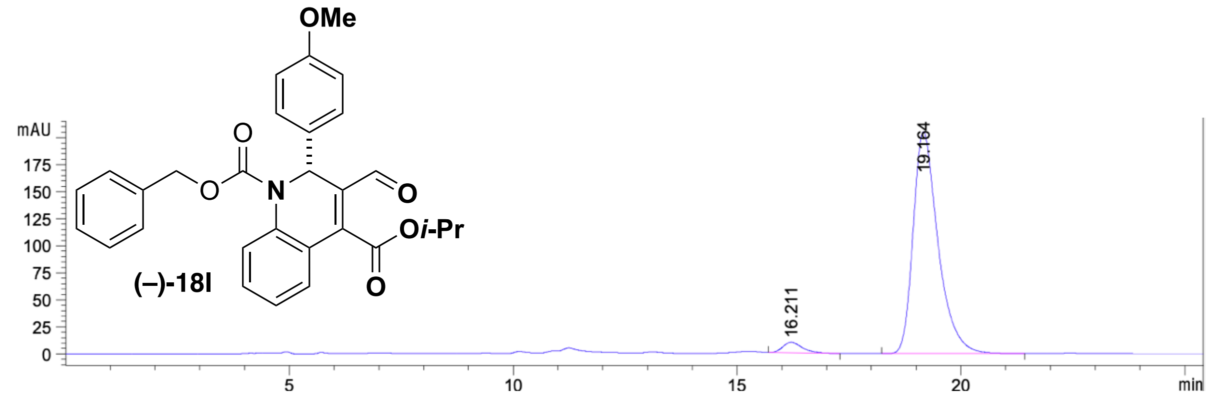

Peak #	RetTime [min]	Type	Width [min]	Area [mAU*s]	Height [mAU]	Area %
1	16.211	BB	0.4580	301.64279	9.88410	3.6648
2	19.164	BB	0.5849	7929.26514	205.18085	96.3352

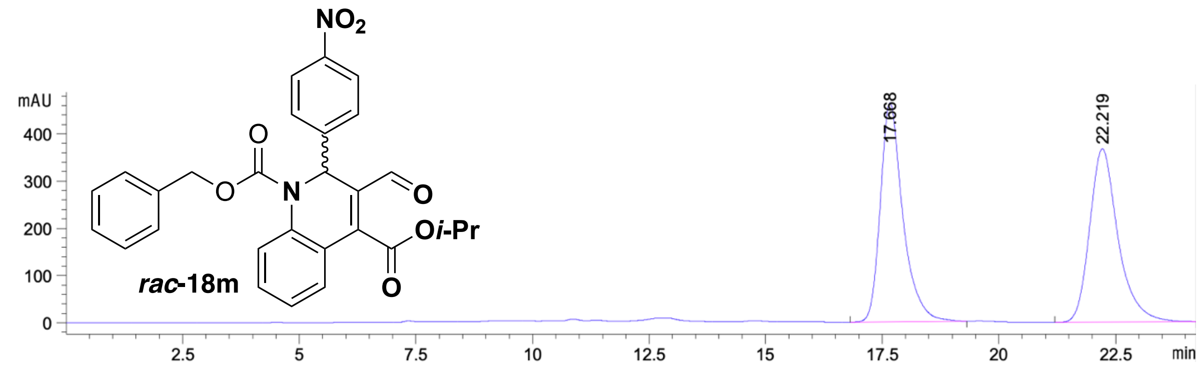

Peak #	RetTime [min]	Type	Width [min]	Area [mAU*s]	Height [mAU]	Area %
1	17.668	BB	0.5014	1.55566e4	463.71231	49.9668
2	22.219	BBA	0.6391	1.55773e4	367.03885	50.0332

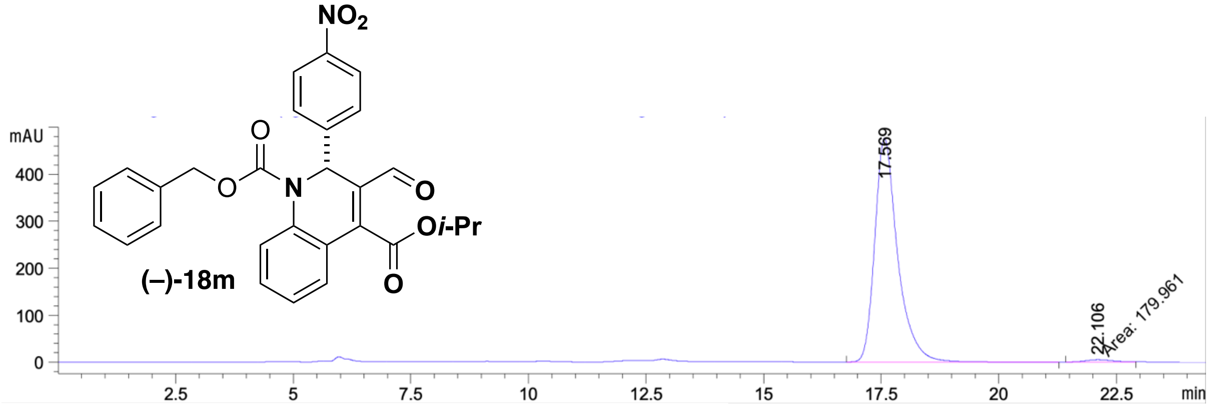

Peak #	RetTime [min]	Type	Width [min]	Area [mAU*s]	Height [mAU]	Area %
1	17.569	BB	0.5036	1.60740e4	476.47421	98.8928
2	22.106	MM	0.6478	179.96133	4.63034	1.1072

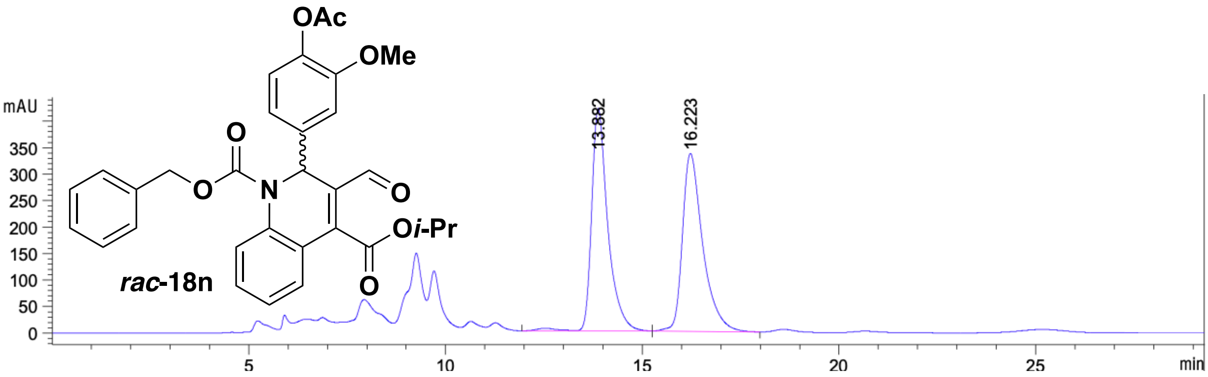

Peak #	RetTime [min]	Type	Width [min]	Area [mAU*s]	Height [mAU]	Area %
1	13.882	VB R	0.4190	1.20528e4	420.46768	50.0143
2	16.223	BB	0.5364	1.20459e4	335.91287	49.9857

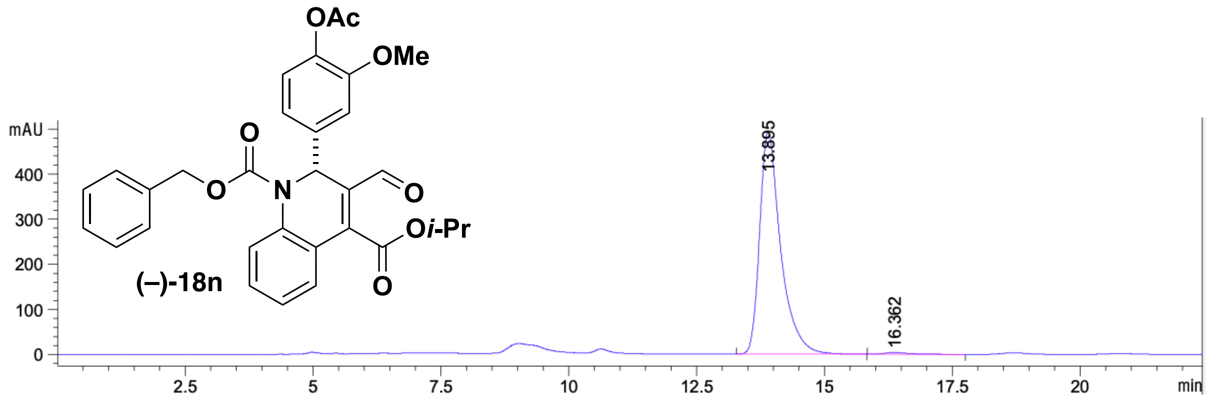

Peak #	RetTime [min]	Type	Width [min]	Area [mAU*s]	Height [mAU]	Area %
1	13.895	BB	0.4261	1.40618e4	493.93875	99.0973
2	16.362	BB	0.5192	128.08990	3.61652	0.9027

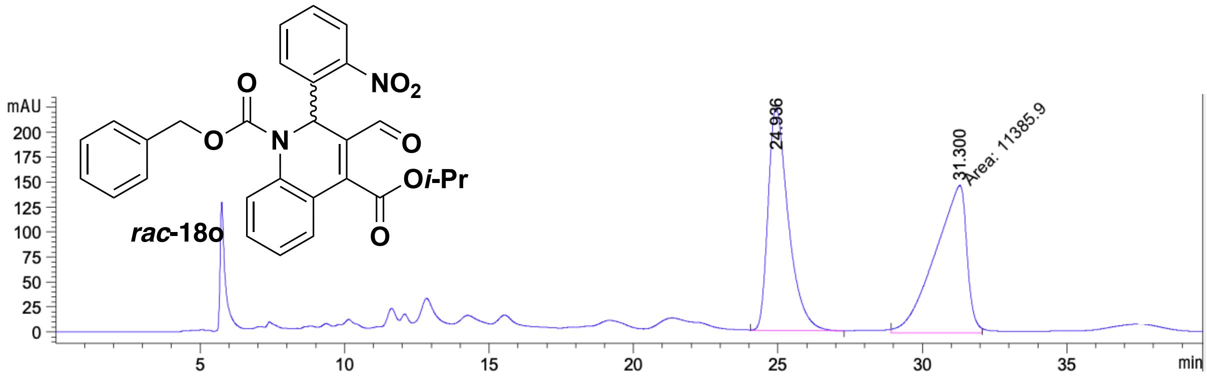

Peak #	RetTime [min]	Type	Width [min]	Area [mAU*s]	Height [mAU]	Area %
1	24.936	BB	0.7363	1.08363e4	222.44171	48.7634
2	31.300	MM	1.2834	1.13859e4	147.86238	51.2366

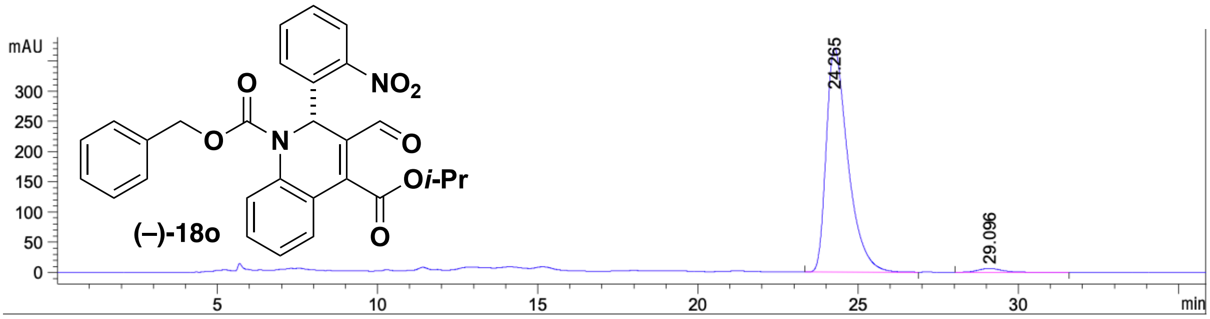

Peak #	RetTime [min]	Type	Width [min]	Area [mAU*s]	Height [mAU]	Area %
1	24.265	BB	0.7150	1.75134e4	370.80646	98.1123
2	29.096	BB	0.7727	336.96613	6.33371	1.8877

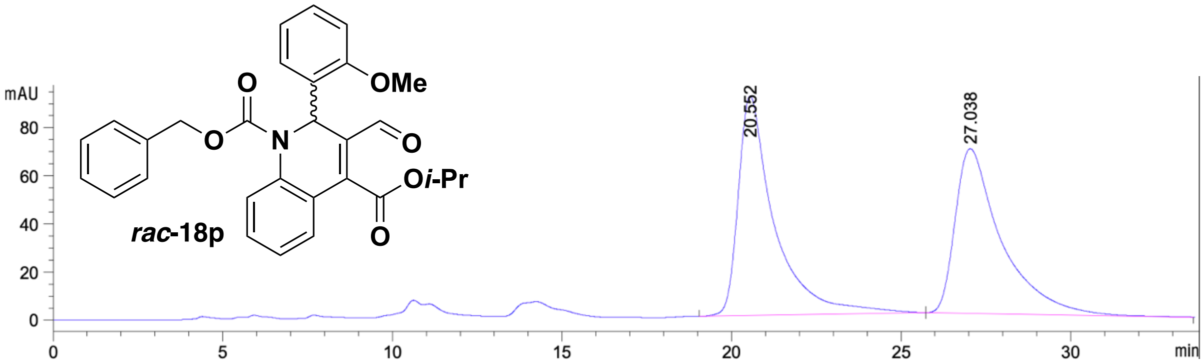

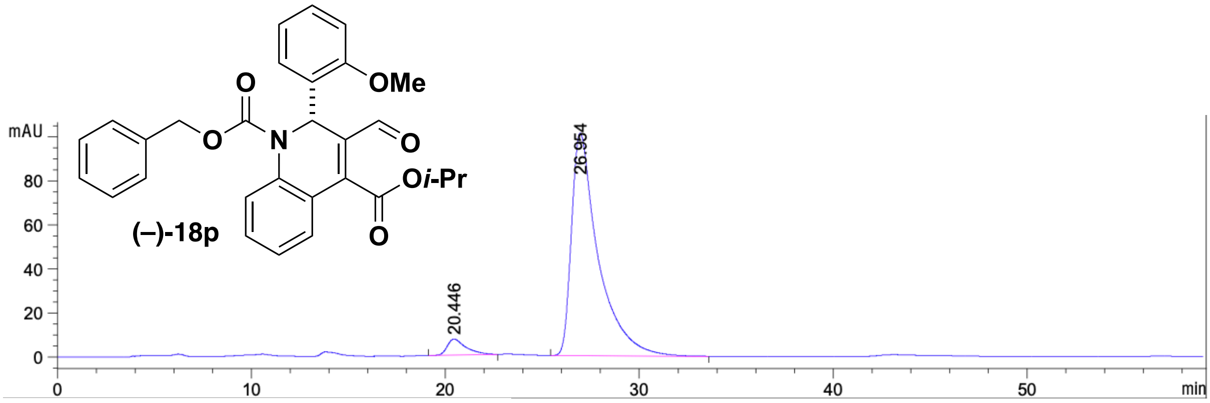

Peak #	RetTime [min]	Type	Width [min]	Area [mAU*s]	Height [mAU]	Area %
1	20.446	BB	0.9299	483.09656	7.26373	4.7587
2	26.954	BB	1.3773	9668.84277	100.96300	95.2413

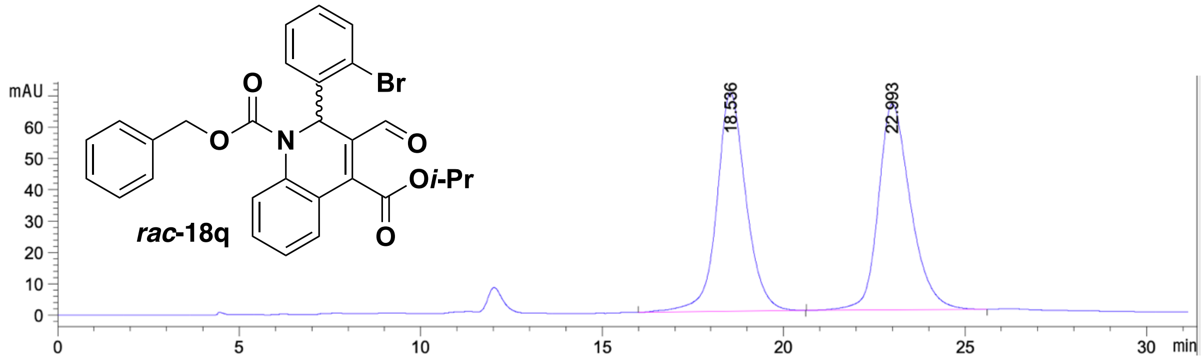

Peak #	RetTime [min]	Type	Width [min]	Area [mAU*s]	Height [mAU]	Area %
1	18.536	BB	0.8460	3926.53979	69.83287	49.1156
2	22.993	BB	0.9312	4067.94727	65.80273	50.8844

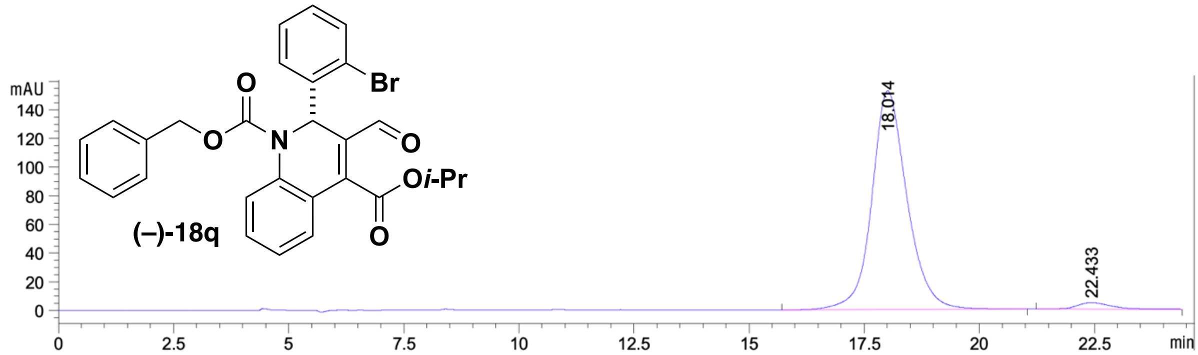

Peak #	RetTime [min]	Type	Width [min]	Area [mAU*s]	Height [mAU]	Area %
1	18.014	BB	0.7975	8138.17627	152.76128	97.2732
2	22.433	BBA	0.7123	228.13245	4.46355	2.7268

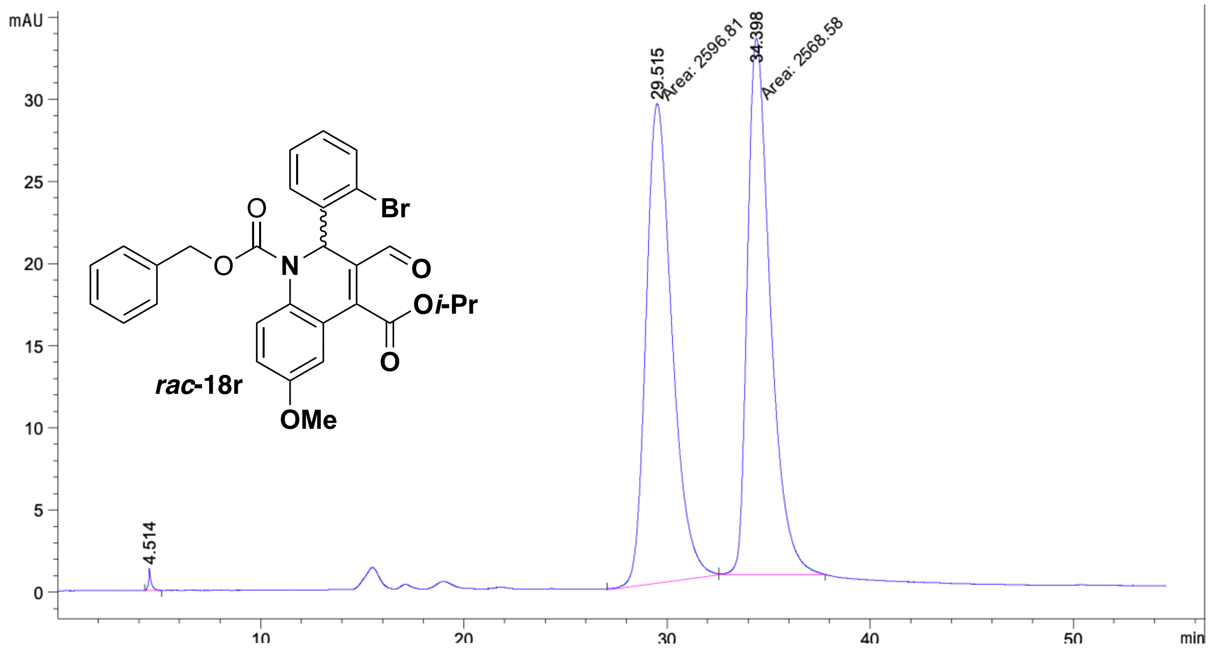

Peak #	RetTime [min]	Type	Width [min]	Area [mAU*s]	Height [mAU]	Area %
1	4.514	BB	0.1165	11.80692	1.34380	0.2281
2	29.515	MM	1.4833	2596.80713	29.17749	50.1586
3	34.398	MM	1.3113	2568.57715	32.64579	49.6133

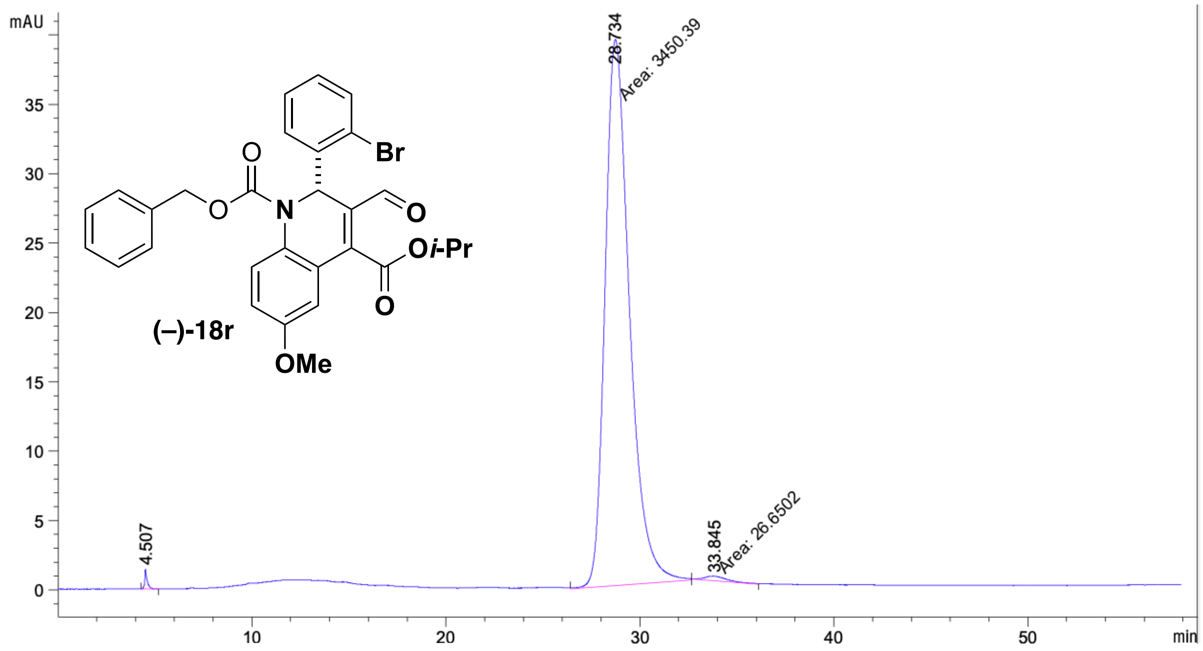

Peak #	RetTime [min]	Type	Width [min]	Area [mAU*s]	Height [mAU]	Area %
1	4.507	BB	0.1164	12.44926	1.39071	0.3568
2	28.734	MM	1.4609	3450.38574	39.36285	98.8795
3	33.845	MM	1.3311	26.65024	3.33696e-1	0.7637

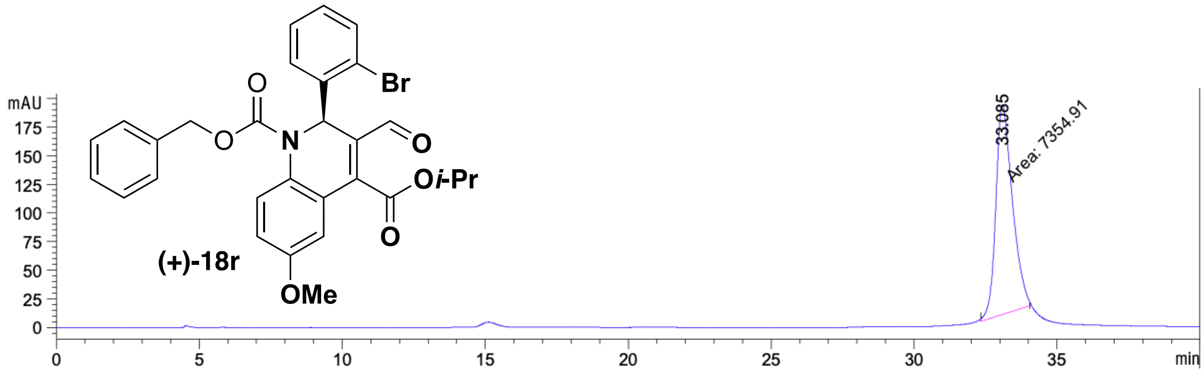

Signal 1: DAD1 A, Sig=254,4 Ref=off

Peak #	RetTime [min]	Type	Width [min]	Area [mAU*s]	Height [mAU]	Area %
1	33.085	MM	0.6697	7354.91016	183.04808	100.0000
